# Funding and services needed to achieve universal health coverage: applications of global, regional, and national estimates of utilisation of outpatient visits and inpatient admissions from 1990 to 2016, and unit costs from 1995 to 2016

**DOI:** 10.1016/S2468-2667(18)30213-5

**Published:** 2018-12-12

**Authors:** Mark W Moses, Paola Pedroza, Ranju Baral, Sabina Bloom, Jonathan Brown, Abby Chapin, Kelly Compton, Erika Eldrenkamp, Nancy Fullman, John Everett Mumford, Vishnu Nandakumar, Katherine Rosettie, Nafis Sadat, Tom Shonka, Abraham Flaxman, Theo Vos, Chris J L Murray, Marcia R Weaver

**Affiliations:** a Institute for Health Metrics and Evaluation, University of Washington, Seattle, WA, USA; b PATH, Seattle, WA, USA

## Abstract

**Background:**

To inform plans to achieve universal health coverage (UHC), we estimated utilisation and unit cost of outpatient visits and inpatient admissions, did a decomposition analysis of utilisation, and estimated additional services and funds needed to meet a UHC standard for utilisation.

**Methods:**

We collated 1175 country-years of outpatient data on utilisation from 130 countries and 2068 country-years of inpatient data from 128 countries. We did meta-regression analyses of annual visits and admissions per capita by sex, age, location, and year with DisMod-MR, a Bayesian meta-regression tool. We decomposed changes in total number of services from 1990 to 2016. We used data from 795 National Health Accounts to estimate shares of outpatient and inpatient services in total health expenditure by location and year and estimated unit costs as expenditure divided by utilisation. We identified standards of utilisation per disability-adjusted life-year and estimated additional services and funds needed.

**Findings:**

In 2016, the global age-standardised outpatient utilisation rate was 5·42 visits (95% uncertainty interval [UI] 4·88–5·99) per capita and the inpatient utilisation rate was 0·10 admissions (0·09–0·11) per capita. Globally, 39·35 billion (95% UI 35·38–43·58) visits and 0·71 billion (0·65–0·77) admissions were provided in 2016. Of the 58·65% increase in visits since 1990, population growth accounted for 42·95%, population ageing for 8·09%, and higher utilisation rates for 7·63%; results for the 67·96% increase in admissions were 44·33% from population growth, 9·99% from population ageing, and 13·55% from increases in utilisation rates. 2016 unit cost estimates (in 2017 international dollars [I$]) ranged from I$2 to I$478 for visits and from I$87 to I$22 543 for admissions. The annual cost of 8·20 billion (6·24–9·95) additional visits and 0·28 billion (0·25–0·30) admissions in low-income and lower-middle income countries in 2016 was I$503·12 billion (404·35–605·98) or US$158·10 billion (126·58–189·67).

**Interpretation:**

UHC plans can be based on utilisation and unit costs of current health systems and guided by standards of utilisation of outpatient visits and inpatient admissions that achieve the highest coverage of personal health services at the lowest cost.

**Funding:**

Bill & Melinda Gates Foundation.

## Introduction

Universal health coverage (UHC) is a global priority. It is one of three strategic priorities of WHO's General Programme of Work for 2019–23.^1^ It is also target 3.8 of the Sustainable Development Goals aimed at achieving “financial risk protection, access to quality essential health-care services, and access to safe, effective, quality and affordable essential medicines and vaccines for all”. Meeting the target will require improvements in population-level interventions, and personal health services to promote health and provide preventive and curative care.^2^ Indicator 3.8.1 on coverage of essential health services and indicator 3.8.2 on financial risk protection will monitor progress towards the target. Researchers have proposed indices of essential health service coverage.^3^, ^4^ The 2016 Sustainable Development Goal Collaborators for the Global Burden of Diseases, Injuries, and Risk Factors Study (GBD) calculated a UHC index of personal health services with 41 items, including coverage of nine tracer interventions and mortality from 32 causes that are amenable to care.^3^ The items represented essential services such as reproductive, maternal, newborn, and child health care, and access to care for infectious diseases, non-communicable diseases, and injuries. More research is needed, however, on utilisation and unit costs of personal health services in the health systems that will expand coverage over the next 12 years.

**Research in context**

**Evidence before this study**

Prospects of expanding access to quality essential health services are improving, as WHO seeks to expand health coverage to 1 billion people by 2023 and countries prepare to meet this target of the Sustainable Development Goals by 2030. Researchers have made progress towards measuring universal health coverage (UHC), but far less is known about the utilisation and unit cost of services of health systems that will expand coverage. We searched the PubMed database on July 3, 2017, for utilisation estimates with the Medical Subject Heading term “global health” and terms for health-care outputs such as “ambulatory care” and “inpatient” as well as the websites for the Organisation of Economic Co-operation and Development, World Bank, and WHO with no date or language restrictions. Utilisation of outpatient visits and inpatient admissions has not been estimated globally, and existing global estimates of unit costs are 10 years old. Researchers have used different methods to estimate the cost of UHC for selected countries only (ie, not globally), and the additional services needed have not been quantified.

**Added value of this study**

We generated a time series of global health-care utilisation, and updated unit cost estimates. We also quantified the volume of services needed to expand access for a given population, and the costs to supply those services. Building on the strengths of methods from the Global Burden of Diseases, Injuries, and Risk Factors Study (GBD) that account for age, sex, spatial, and temporal patterns in health outcomes, and adjusting for differences across heterogeneous data sources, we produced estimates of utilisation per person for visits and admissions by age and sex for 195 countries from 1990 to 2016. We also decomposed changes in the volume of services over time into changes in population size, age and sex structure, and utilisation rates for every location. We estimated the share of total health expenditure on each service using mutually exclusive and collectively exhaustive National Health Account data and the cost per outpatient visit and per inpatient admission for 188 countries from 1995 to 2016. Our macro-costing approach reflected current expenditures and efficiency. We also created UHC standards of utilisation per disability-adjusted life-year based on existing health systems rather than ideals to estimate the additional services and funding needed annually to expand health coverage in 2016 for 188 countries, with results similar to previous research using different methods and groups of countries.

**Implications of all the available evidence**

Globally, outpatient visits have increased by more than half and inpatient admissions have increased by more than two-thirds since 1990. In countries such as China, Indonesia, Thailand, and Turkey, policies to expand coverage are associated with increased utilisation rates. Meanwhile, in several countries in the sub-Saharan Africa super-region with low scores on the GBD's UHC index of personal health services, most of the increases in volume of services have been from population growth rather than increases in utilisation rates. Using the Netherlands as a UHC standard, although both primary and specialty services are essential, the gap in services that countries must overcome to achieve UHC is larger for admissions than for visits. We also identified intermediate UHC standards for utilisation, such as Portugal, for which a smaller increase in admissions would expand coverage initially as a step towards achieving UHC in the future.

Although previous researchers have reported on utilisation for multiple countries, none reported on all countries over time. The Organisation of Economic Co-operation and Development (OECD) reports the annual number of outpatient visits per person and inpatient discharges for 35 member countries (with the exception of inpatient admissions for Canada and the USA), and five non-member countries for selected years. The probabilities of having general practitioner and specialist doctor visits in the past year were estimated for 18 OECD countries using the European Health Interview Survey or the most recent national health survey.^5^ The number of outpatient visits in the past 4 weeks and inpatient admissions in the past year were estimated for 39 countries outside of OECD using World Health Survey data.^6^ Systematic estimates have not been reported for more than half of countries globally, most of which have low scores on the UHC indices.

WHO's Choosing Interventions that are Cost-Effective (WHO-CHOICE) researchers estimated unit costs of outpatient visits and inpatient bed-days for 191 countries in 2007 and 2008 using facility-level data from 30 countries.^7^ Although the WHO-CHOICE estimates were standardised to reflect health systems performing at high levels of efficiency, they have been used extensively in cost-effectiveness analyses when more exact micro-costing estimates were neither practical nor appropriate.^8^, ^9^ The estimates are due for an update, based on nationally representative samples, and bounded by a national health expenditure envelope.

The aim of this study was to support progress towards UHC. The objectives were to produce global estimates of outpatient visits and inpatient admissions by age and sex for 27 years and unit costs for these services for 22 years, and to demonstrate two applications of the estimates to inform expansion of coverage of essential personal health services. We decomposed changes in volume of services by location from 1990 to 2016 into changes in utilisation rates, population size, and age and sex structure of the population to show the role of each factor in every country over time. We estimated the services and funding needed to expand utilisation for the 2016 population size and structure to meet a UHC standard for utilisation per disability-adjusted life-year (DALY) using counterfactual DALY estimates from GBD 2016.

## Methods

### Definition of utilisation

We defined outpatient utilisation rate as the annual number of visits per capita to a health facility that did not result in admission and defined inpatient utilisation rate as the annual number of admissions per capita for one night or more into a health facility. We included preventive, rehabilitative, and curative care, and adhered as closely as possible to the International Classification for Health Accounts' categories for Health-Care Functions (HC) so that the utilisation rate would be consistent with expenditure data based on the System of Health Accounts 2011.^10^ For outpatient visits, our definition mapped to four categories: outpatient curative (HC 1.3) and rehabilitative (HC 2.3) care, facility-based preventive maternal and child care (HC 6.4), and vaccinations (HC 6.2). For inpatient admissions, our definition mapped to two categories: inpatient curative (HC 1.1) and rehabilitative (HC 2.1) care. Our estimates excluded day-patient admissions (HC 1.2 and HC 2.2), and long-term care (HC 3) because data on their utilisation and expenditures were not available globally.

### Data sources for utilisation estimates

We compiled data sources from a systematic review of surveys and administrative data within the Global Health Data Exchange. All data sources were nationally or subnationally representative. In compliance with the Guidelines for Accurate and Transparent Health Estimates Reporting,^11^ we documented the methods of the systematic review, data sources for each country, data processing, and estimation (appendix p 6).

We compiled outpatient utilisation data from 130 countries, spanning 1175 country-years, and inpatient data from 128 unique countries, spanning 2068 country-years (appendix p 11). Administrative sources contributed 59·1% of outpatient country-years and 80·3% of inpatient country-years. More data were available from administrative records in the high-income and central Europe, eastern Europe, and central Asia super-regions due to their well established reporting systems. More than half of the data sources were surveys for the other super-regions, except for inpatient data for north Africa and the Middle East.

### Methods for utilisation estimates

The unit of analysis was average utilisation by sex and age categories, where the 23 age categories were early neonatal (0–6 days), neonatal (7–27 days), infants (28–364 days), 1–4 years, followed by 5-year intervals from 5–9 years to 90–94 years, and finally 95 years or older. We estimated the age-sex-specific rates of utilisation for visits and admissions with DisMod-MR, version 2.1. DisMod-MR is a Bayesian hierarchical meta-regression method and an established method to estimate age-sex-specific incidence and prevalence rates of diseases by location and year.^12^, ^13^

Measures of utilisation and recall periods were not consistent across surveys (appendix p 19), and we used two methods to adjust for inconsistencies. When inconsistencies across data sources did not differ by age category, we included dichotomous covariates in the DisMod-MR models. The reference category was annually reported, administrative records from either national sources or facility-level health information system data. For the outpatient utilisation model, we created four covariates for recall periods and two for inconsistent phrasing of the utilisation questions. For the inpatient utilisation model, we created two covariates for survey series such as the World Health Survey. When inconsistencies differed by age category such as 1-year recall of inpatient admissions, we used age-spline regressions to adjust for the differences before estimating the DisMod-MR models (appendix pp 23–27).

Additional covariates were the Socio-demographic Index—a summary development indicator of income per capita, years of schooling, and total fertility rate—in the outpatient model and hospital beds per 1000 population in the inpatient model. The rationale for including each covariate, their definitions, and estimated coefficients are reported in the appendix (pp 28–29). To account for geographical variation, we used random effects to nest GBD super-regions, regions, and countries (appendix pp 13–18).

### Decomposition of changes in utilisation

The total volume of outpatient visits and inpatient admissions was calculated by multiplying age-sex-specific utilisation rates for each location by the population for each category sourced from GBD 2016 national estimates.^14^ Age-sex-specific utilisation rates by GBD super-region are in the appendix (pp 34–36). We decomposed changes in total volume of services from 1990 to 2016 into changes in four factors: utilisation rates by age and sex, population growth, population ageing, and sex composition. Decomposition of these factors followed the method in Das Gupta^15^ to estimate the average marginal effect of changing one factor across all combinations of changes in the other factors.

### Unit cost estimates

We estimated unit costs as expenditure per capita on each service divided by utilisation per capita. Expenditure per capita was the product of total health expenditures (THE) per capita in 2017 international dollars and the share of outpatient services in THE for visits or share of inpatient services for admissions. THE estimates^16^ from 1995 to 2015 and projections for 2016^17^ were available for 188 countries. The shares were estimated with 795 country-years of National Health Accounts data, which provided a mutually exclusive and collectively exhaustive account of the flow of THE through a health system (appendix pp 51–52). The sample represented 105 (56%) of 188 countries but fewer than half of the countries in three super-regions: southeast Asia, east Asia, and Oceania; Latin America and the Caribbean; and north Africa and the Middle East. Outpatient spending was estimated as the share of outpatient curative (HC 1.3) and rehabilitative (HC 2.3) care and inpatient spending was estimated as the share of inpatient curative (HC 1.1) and rehabilitative (HC 2.1) care.

### Cost estimates to meet a UHC standard for utilisation

We estimated the additional services and funds needed to meet a UHC standard for utilisation. The metric for the units of service was the 2016 volume of services per DALY, using a counterfactual estimate of a DALY based on GBD 2016 data. A country's 2016 burden of disease was endogenous to its current health service utilisation, meaning that improved access and quality of services affected the burden. We standardised the burden of disease across countries by removing the effects of access and quality of services using age-specific estimates of the GBD 2016 Healthcare Access and Quality (HAQ) index.^18^ We regressed 2016 DALYs for each age and sex category on the Socio-demographic Index and HAQ index; we predicted the counterfactual DALYs by setting the HAQ index to zero and thus removing the effects of access and quality (appendix pp 61–63).

Our UHC standard for utilisation—ie, services per counterfactual DALY—was based on an existing health system rather than an ideal. For each country, we calculated the additional units of service needed to meet the UHC standard and multiplied the total by the unit cost of service in that country. Units of service needed was the difference between the standard for each age-sex combination and the country's 2016 volume per counterfactual DALY for that age-sex combination; this was then multiplied by those DALYs to obtain an estimate given in units of service. The national total was the sum of units of service across age-sex categories.

To identify the UHC standard for utilisation, we set each country in turn as the standard and calculated the global cost to reach that standard (see appendix p 71 for the 188 global estimates). Several countries formed a frontier, with high value on the GBD 2016 UHC index and lowest global cost for their value. Among countries on the frontier, we selected one from each of the top two quintiles of the Socio-demographic Index to serve as standards: from the first quintile we chose the Netherlands, ranked ninth on the UHC index, as the standard for the main analysis and from the second quintile we chose Portugal, ranked 34th on the UHC index, as an intermediate UHC standard for a sensitivity analysis. The aggregate ratio of total volume to counterfactual DALYs was 7·25 for visits and 0·17 for admissions for the Netherlands (see appendix pp 74–75 for the age-sex-specific ratios that were the standards used to calculate units of service needed) and 7·01 for visits and 0·14 for admissions for Portugal.

Health systems differed in the quality and type of services they provided, as well as in the volume of services. We estimated that the unit costs in the Netherlands were 28% higher for visits than predicted by cost-of-living differences in gross domestic product per capita and 24% higher for admissions (appendix p 85). We did a sensitivity analysis with unit costs increased by these percentages as a measure of improvements.

### Uncertainty

We captured and propagated uncertainty in the analysis, including all three steps of the utilisation estimates: sampling uncertainty from extracted data, uncertainty from adjustments to inconsistently reported data, and uncertainty estimated as part of DisMod-MR. For all reported estimates, we took 1000 draws from the posterior distributions. The mean of the 1000 draws was the point estimate and the 2·5th and 97·5th percentile of the draws defined the uncertainty interval (UI). Applications using modelled outputs were done at the draw level.

### Role of the funding source

The funder of the study had no role in study design, data collection, data analysis, data interpretation, or writing of the report. The corresponding author had complete access to all the data in the study and had final responsibility for the decision to submit for publication.

## Results

**Figure 1:**
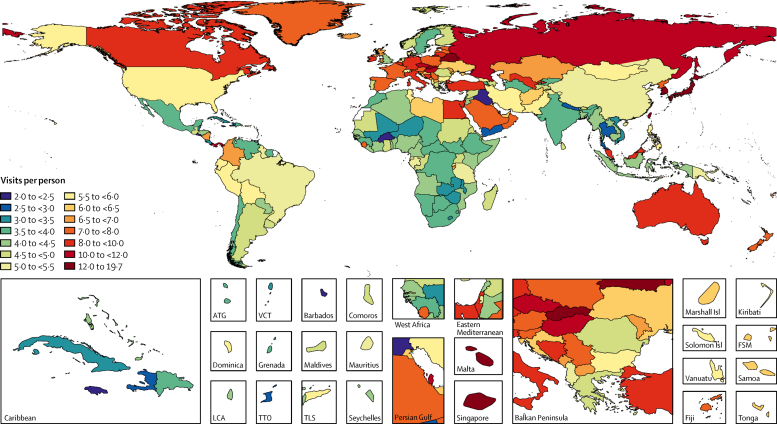
Annual outpatient visits per capita, age-standardised and for both sexes combined, by location, 2016 Map displays the age-standardised estimated annual number of outpatient visits per capita in 2016 for all ages and both sexes combined. The rate ranged from 2·5 to 7·0 visits per person for the majority of countries, and the key shows 0·5 visit increments in this range to present differences among these countries. ATG=Antigua and Barbuda. FSM=Federated States of Micronesia. Isl=Islands. LCA=Saint Lucia. TTO=Trinidad and Tobago. TLS=Timor-Leste. VCT=Saint Vincent and the Grenadines.

**Figure 2:**
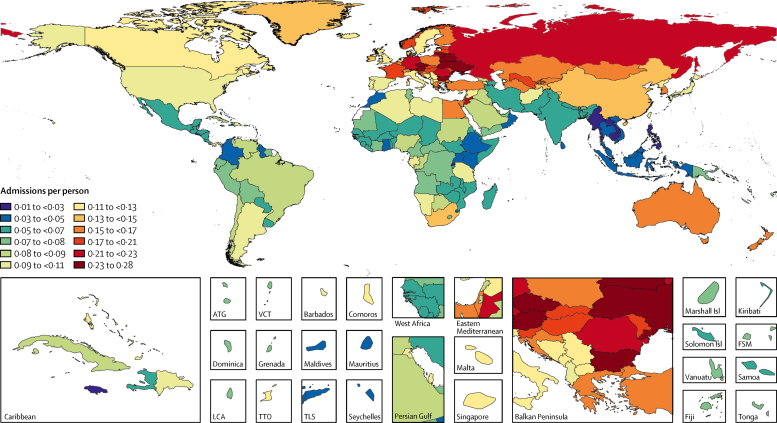
Annual inpatient admissions per capita, age-standardised and for both sexes combined, by location, 2016 Map displays the age-standardised estimated annual number of inpatient admissions per capita in 2016 for all ages and both sexes combined. ATG=Antigua and Barbuda. FSM=Federated States of Micronesia. Isl=Islands. LCA=Saint Lucia. TTO=Trinidad and Tobago. TLS=Timor-Leste. VCT=Saint Vincent and the Grenadines.

The global age-standardised utilisation rates were 5·42 outpatient visits (95% UI 4·88–5·98) and 0·10 inpatient admissions (0·09–0·11) per capita in 2016. The age-standardised utilisation rate for outpatient visits was highest in the high-income Asia-Pacific (15·46, 95% UI 14·02–17·06) and eastern European (10·29, 9·78–10·79) regions, and lowest in southern sub-Saharan Africa (3·53, 3·03–4·08) and the Caribbean (3·37, 2·89–3·88; figure 1; appendix pp 37–43). Taiwan (province of China) had the highest outpatient utilisation rate (19·61, 17·04–22·44) and Burkina Faso had the lowest (2·00, 1·17–2·32). The age-standardised utilisation rates for inpatient admissions were highest in the eastern Europe (0·23, 0·22–0·24) and central Europe (0·18, 0·17–0·20) regions and lowest in southeast Asia (0·03, 0·02–0·04) and eastern sub-Saharan Africa (0·05, 0·05–0·06; figure 2; appendix pp 44–50). Bulgaria had the highest inpatient utilisation rate (0·27, 0·26–0·28) and Cambodia had the lowest (0·02, 0·02–0·03).

Many countries were exceptions to the regional patterns, and the range of estimates within some regions was broad. In western Europe where the age-standardised outpatient rate was 7·33 (95% UI 6·68–8·12), the rates were below the global average in Scandinavia, England, Greece, the Netherlands, and Portugal. In central Latin America where the outpatient rate was 4·60 (3·99–5·27), the rates were above the global average in Colombia, Nicaragua, and Panama.

**Figure 3:**
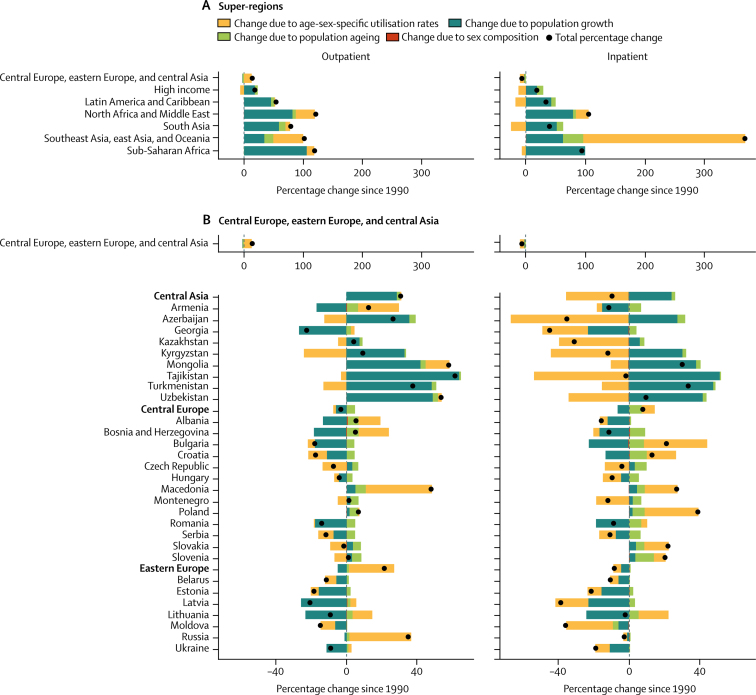

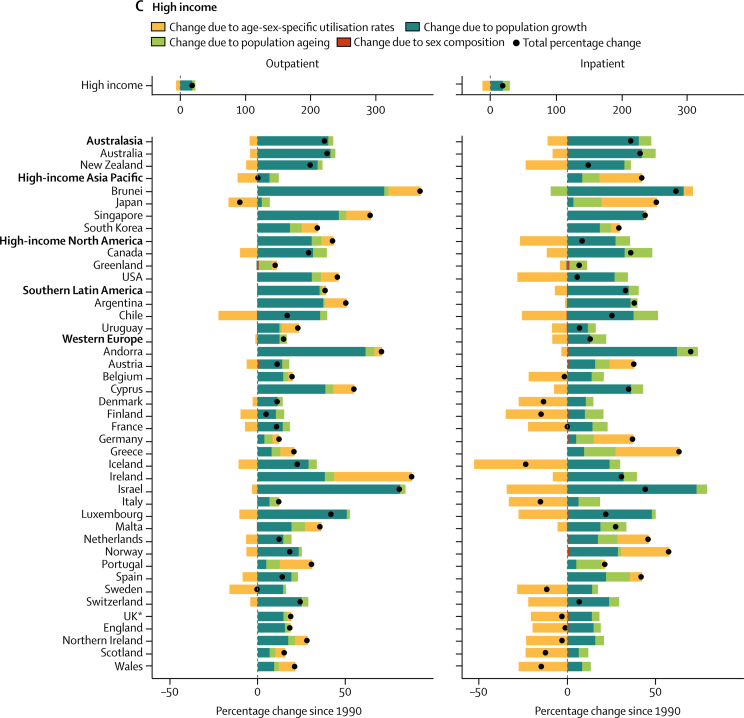

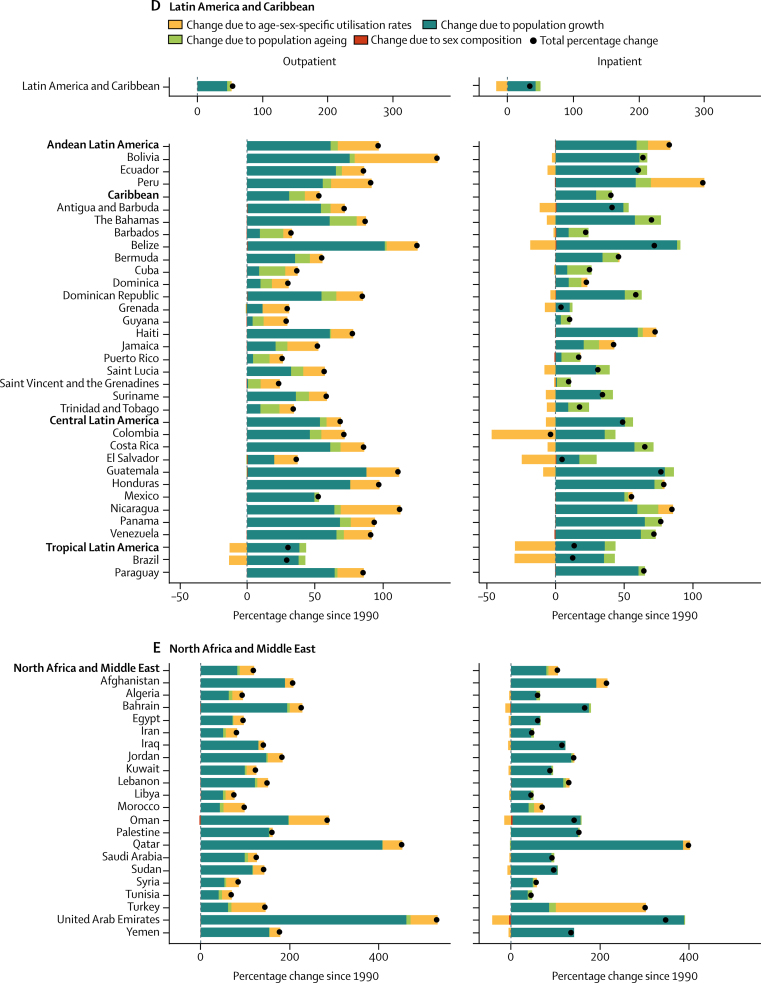

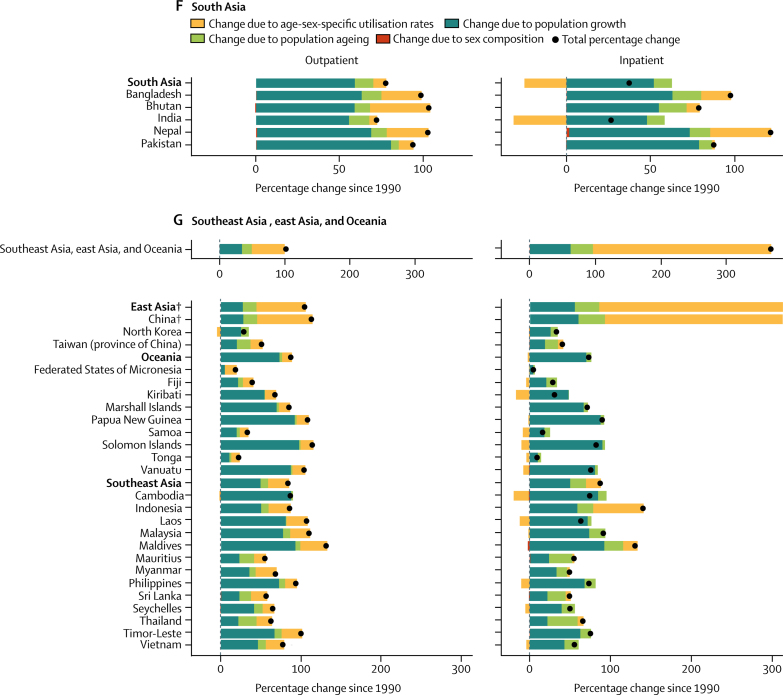

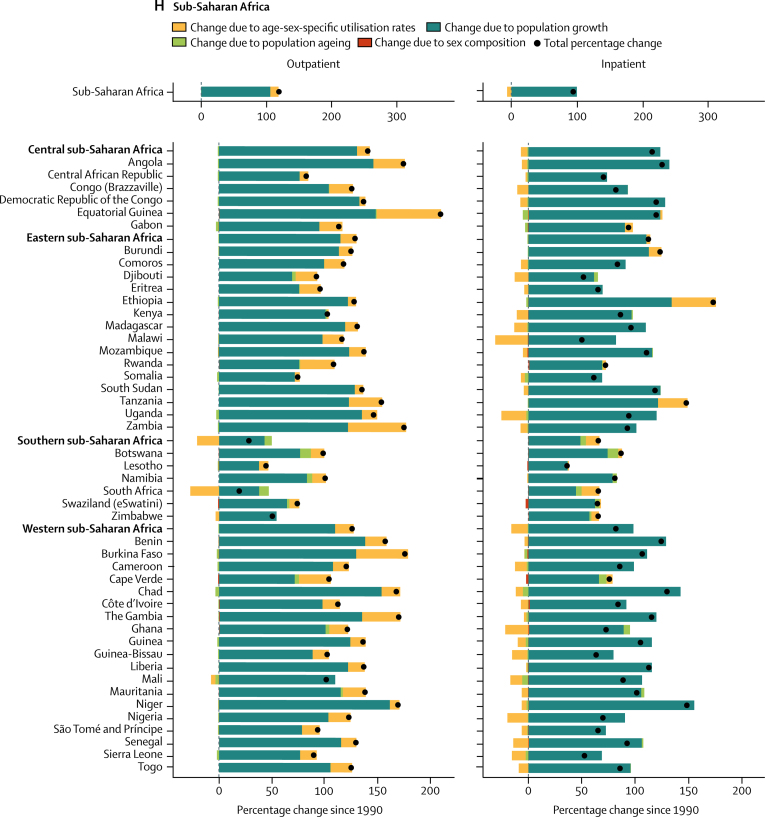
Decomposition of the percentage change in volume of outpatient visits and inpatient admissions for all ages and both sexes summarised by GBD super-region and by region and country, 1990–2016 Changes in the volume of outpatient visits and inpatient admissions from 1990 to 2016 were decomposed into changes in four factors: age-sex-specific utilisation rates, total population, the share of the population in each age category, and the share of the population of each sex within each age category. The black dots represent the overall percentage change in volume of each service. Colours represent the percentage that each factor contributed to overall percentage change. Bars to the left of zero show that the factor contributed to a decrease and bars to the right show an increase. GBD=Global Burden of Diseases, Injuries, and Risk Factors Study. *UK data are an aggregate of the data from the four constituent countries displayed below. †Results extend off the scale. For east Asia, change due to age-sex utilisation rates is 357%, contributing to a total percentage change of 443%. For China, change due to age-sex utilisation rates is 404%, contributing to a total percentage change of 497%.

From 1990 to 2016, outpatient volume increased from 24·80 billion (95% UI 21·81–28·17) to 39·35 billion (35·38–43·58) visits globally. Of this 58·65% increase in visits, 42·95% was from population growth, 8·09% from population ageing, and 7·63% from increases in utilisation rates; small changes in the sex composition of the population (ie, if utilisation rates differ between the sexes) account for the difference between the total change from 1990 to 2016 and the sum of three factors reported here. Changes over time in the age-sex-specific outpatient utilisation rates increased volume in six super-regions (figure 3A), with the exception of the high-income region. Inpatient volume increased from 0·42 billion (0·38–0·47) to 0·71 billion (0·65–0·77) admissions. Of this 67·96% increase in admissions, 44·33% was from population growth, 9·99% from population ageing, and 13·55% from increases in utilisation rates. Changes in inpatient utilisation rates decreased volume in five super-regions, with the exception of southeast Asia, east Asia, and Oceania and north Africa and the Middle East.

Increases in China's age-sex-specific utilisation rates accounted for most of their sizable increase in volume of services from 1990 to 2016 (figure 3G; inpatient results for China extend off the scale). The 114·41% increase in outpatient visits decomposed into a 69·13% increase from utilisation rates, 27·94% from population growth, and 17·26% from population ageing. The 497·00% increase in inpatient admissions decomposed into a 403·85% increase from utilisation rates, 59·80% from population growth, and 32·73% from population ageing. Increases in age-sex-specific utilisation rates also accounted for large increases in outpatient visits in Thailand (19·44% of a 63·85% increase; figure 3G) and inpatient admissions in Indonesia (62·35 % of a 141·01% increase; figure 3G) and Turkey (202·22% of a 302·87% increase; figure 3E).

Central Europe, eastern Europe, and central Asia was the only super-region with a decrease, albeit small, in the volume of inpatient admissions (figure 3B). In the central Asia region, the 9·00% decrease in inpatient admissions decomposed into a 35·30% decrease from utilisation rates, offset by a 24·24% increase from population growth and 2·00% increase from population ageing. In eastern Europe, the 7·96% decrease in inpatient admissions decomposed into a 4·40% decrease from utilisation rates and 4·44% from population decline, offset by a 0·66% increase from population ageing.

In 2016, the cost per outpatient visit (in 2017 international dollars [I$]) ranged from I$2 (in Burundi, Eritrea, and Central African Republic) to I$478 (USA; table). The cost per inpatient admission ranged from I$87 (Central African Republic) to I$22 543 (USA; table). Unit cost estimates generally followed patterns of THE per capita. Spearman rank correlation coefficients for THE per capita were 0·93 for outpatient costs and 0·89 for inpatient costs. Correlation coefficients for share of expenditure were 0·39 for outpatient costs and 0·67 for inpatient costs whereas those for utilisation per capita were 0·26 for outpatient costs and 0·25 for inpatient costs.

**Table:** National unit costs of outpatient visits and inpatient admissions, utilisation per counterfactual DALY, and additional visits, admissions, and funds needed to achieve a UHC standard for utilisation in 2016

		**UHC index, unscaled**	**Outpatient visits**			**Inpatient admissions**			**Total additional cost to meet UHC standard (I$, millions)**	**Total additional cost as percentage of GDP**
			Unit cost per outpatient visit (I$)	Ratio of total outpatient visits to counterfactual DALYs	Additional outpatient visits to meet UHC standard (thousands)	Unit cost per inpatient admission (I$)	Ratio of total inpatient admissions to counterfactual DALYs	Additional inpatient admissions to meet UHC standard (thousands)		
**Global**		**..**	**..**	**6·7 (6·04 to 7·41)**	**10 415 092 (7 809 849 to 12 742 483)**	**..**	**0·12 (0·11 to 0·13)**	**348 465 (306 581 to 383 242)**	**1 177 689 (896 052 to 1 456 564)**	**..**
Low income		..	..	..	1 956 058 (1 552 914 to 2 280 749)	..	..	61 106 (54 415 to 66 067)	47 089 (37 379 to 56 079)	4·03% (3·2 to 4·8)
Lower-middle income		..	..	..	6 224 613 (4 643 059 to 7 683 756)	..	..	214 966 (194 755 to 231 539)	456 036 (365 535 to 550 888)	2·20% (1·77 to 2·66)
Upper-middle income		..	..	..	2 023 229 (1 418 055 to 2 614 556)	..	..	59 265 (46 625 to 70 696)	408 357 (314 283 to 500 039)	0·92% (0·71 to 1·12)
High income		..	..	..	211 192 (83 151 to 330 668)	..	..	13 128 (6499 to 18 933)	266 208 (152 214 to 380 707)	0·47% (0·27 to 0·68)
**Central Europe, eastern Europe, and central Asia**		**..**	**..**	**10·82 (10·37 to 11·31)**	**116 530 (86 387 to 145 714)**	**..**	**0·24 (0·23 to 0·25)**	**4372 (2820 to 6017)**	**13 979 (10 538 to 17 605)**	**..**
Central Asia		..	..	8·25 (7·95 to 8·54)	54 694 (48 267 to 61 347)	..	0·17 (0·17 to 0·18)	1745 (1556 to 1936)	6596 (5829 to 7359)	..
	Armenia	0·65 (0·63 to 0·67)	48 (40 to 57)	4·86 (4·67 to 5·05)	5691 (5076 to 6307)	1710 (1426 to 2019)	0·16 (0·16 to 0·17)	63 (51 to 75)	386 (307 to 468)	1·37% (1·09 to 1·66)
	Azerbaijan	0·58 (0·54 to 0·61)	62 (54 to 70)	5·8 (5·53 to 6·08)	9910 (8076 to 11 755)	4938 (4342 to 5613)	0·08 (0·08 to 0·09)	581 (539 to 621)	3512 (2985 to 4077)	1·98% (1·68 to 2·3)
	Georgia	0·59 (0·56 to 0·62)	31 (26 to 38)	7·13 (6·13 to 8·43)	1736 (359 to 3125)	2408 (2128 to 2720)	0·11 (0·1 to 0·11)	209 (185 to 232)	565 (454 to 688)	1·35% (1·08 to 1·64)
	Kazakhstan	0·63 (0·6 to 0·66)	39 (35 to 43)	8·27 (7·91 to 8·65)	2937 (1224 to 4814)	1872 (1699 to 2046)	0·19 (0·18 to 0·2)	217 (163 to 277)	523 (382 to 693)	0·11% (0·08 to 0·15)
	Kyrgyzstan	0·59 (0·58 to 0·61)	18 (16 to 20)	4·06 (3·91 to 4·22)	14 137 (12 960 to 15 301)	525 (472 to 595)	0·17 (0·17 to 0·18)	83 (60 to 103)	294 (255 to 343)	1·35% (1·17 to 1·57)
	Mongolia	0·59 (0·56 to 0·62)	22 (18 to 26)	6·39 (5·38 to 7·61)	1669 (388 to 2868)	907 (717 to 1126)	0·18 (0·15 to 0·22)	32 (14 to 48)	67 (34 to 105)	0·18% (0·09 to 0·27)
	Tajikistan	0·56 (0·53 to 0·58)	9 (8 to 10)	5·69 (5·44 to 5·96)	8781 (7148 to 10 447)	410 (368 to 459)	0·15 (0·15 to 0·16)	162 (130 to 192)	143 (117 to 173)	0·55% (0·44 to 0·66)
	Turkmenistan	0·56 (0·54 to 0·58)	78 (68 to 89)	4·81 (4·6 to 5·02)	9620 (8440 to 10 757)	2182 (1706 to 2668)	0·2 (0·16 to 0·24)	44 (16 to 66)	851 (694 to 1026)	0·79% (0·64 to 0·95)
	Uzbekistan	0·6 (0·57 to 0·63)	10 (9 to 11)	11·85 (11·37 to 12·32)	212 (−318 to 764)	717 (648 to 790)	0·21 (0·2 to 0·22)	353 (275 to 431)	255 (193 to 320)	0·11% (0·08 to 0·14)
Central Europe		..	..	10·21 (9·83 to 10·6)	25 786 (19 317 to 32 104)	..	0·25 (0·23 to 0·27)	737 (344 to 1109)	3593 (2377 to 4878)	..
	Albania	0·67 (0·64 to 0·69)	36 (30 to 44)	6·98 (5·92 to 8·13)	1189 (−163 to 2405)	2546 (2275 to 2879)	0·12 (0·11 to 0·12)	129 (110 to 147)	378 (282 to 474)	1·05% (0·79 to 1·32)
	Bosnia and Herzegovina	0·65 (0·62 to 0·68)	25 (21 to 29)	12·28 (10·83 to 14·43)	0 (0 to 0)	2550 (2104 to 3087)	0·14 (0·12 to 0·16)	115 (36 to 185)	305 (82 to 562)	0·68% (0·18 to 1·25)
	Bulgaria	0·63 (0·61 to 0·66)	68 (63 to 74)	7·17 (6·7 to 7·63)	3478 (1720 to 5113)	1592 (1460 to 1728)	0·35 (0·33 to 0·37)	0 (0 to 0)	239 (118 to 358)	0·16% (0·08 to 0·24)
	Croatia	0·72 (0·71 to 0·75)	69 (63 to 76)	8·06 (7·74 to 8·42)	965 (417 to 1483)	2753 (2489 to 3067)	0·23 (0·21 to 0·24)	41 (18 to 64)	180 (86 to 277)	0·18% (0·08 to 0·27)
	Czech Republic	0·76 (0·75 to 0·78)	64 (59 to 69)	14·1 (13·48 to 14·84)	0 (0 to 0)	3035 (2546 to 3547)	0·32 (0·28 to 0·38)	0 (0 to 0)	0 (0 to 0)	0·00% (0·00 to 0·00)
	Hungary	0·7 (0·68 to 0·72)	42 (38 to 45)	15·6 (15·03 to 16·24)	0 (0 to 0)	2764 (2528 to 3013)	0·26 (0·24 to 0·28)	24 (−35 to 81)	68 (−92 to 229)	0·02% (−0·03 to 0·08)
	Macedonia	0·64 (0·62 to 0·65)	33 (28 to 38)	8·32 (7·93 to 8·72)	469 (321 to 613)	2081 (1800 to 2521)	0·15 (0·14 to 0·16)	57 (38 to 74)	135 (87 to 188)	0·44% (0·28 to 0·6)
	Montenegro	0·69 (0·67 to 0·71)	33 (30 to 36)	9·39 (8·79 to 9·95)	20 (−21 to 60)	2264 (2067 to 2469)	0·16 (0·15 to 0·17)	14 (10 to 17)	32 (22 to 42)	0·30% (0·2 to 0·39)
	Poland	0·72 (0·7 to 0·74)	62 (57 to 67)	10·21 (9·78 to 10·63)	0 (0 to 0)	2940 (2679 to 3219)	0·24 (0·22 to 0·25)	77 (−95 to 246)	229 (−267 to 730)	0·02% (−0·02 to 0·07)
	Romania	0·66 (0·64 to 0·68)	58 (53 to 65)	6·11 (5·83 to 6·38)	19 296 (15 002 to 23 587)	1393 (1231 to 1568)	0·29 (0·27 to 0·32)	10 (−60 to 74)	1149 (830 to 1481)	0·25% (0·18 to 0·32)
	Serbia	0·65 (0·63 to 0·67)	44 (40 to 48)	9·3 (8·93 to 9·65)	368 (−307 to 987)	3155 (2844 to 3495)	0·15 (0·14 to 0·16)	263 (204 to 320)	852 (639 to 1091)	0·64% (0·48 to 0·82)
	Slovakia	0·7 (0·68 to 0·72)	47 (43 to 52)	16·97 (16·28 to 17·65)	0 (0 to 0)	3164 (2865 to 3499)	0·28 (0·26 to 0·29)	5 (−6 to 15)	15 (−20 to 48)	0·01% (−0·01 to 0·03)
	Slovenia	0·8 (0·78 to 0·82)	100 (93 to 107)	9·87 (9·49 to 10·25)	0 (0 to 0)	4347 (3986 to 4707)	0·25 (0·23 to 0·26)	3 (−3 to 8)	12 (−12 to 37)	0·02% (−0·02 to 0·05)
Eastern Europe		..	..	12·12 (11·51 to 12·8)	36 050 (12 837 to 57 240)	..	0·26 (0·25 to 0·27)	1889 (666 to 3266)	3790 (1461 to 6375)	..
	Belarus	0·7 (0·67 to 0·73)	23 (22 to 25)	14·85 (14·32 to 15·42)	0 (0 to 0)	1348 (1122 to 1629)	0·29 (0·24 to 0·35)	54 (−74 to 149)	75 (−101 to 215)	0·04% (−0·05 to 0·11)
	Estonia	0·74 (0·72 to 0·77)	66 (61 to 72)	10·29 (9·92 to 10·66)	0 (0 to 0)	3052 (2793 to 3329)	0·24 (0·23 to 0·26)	11 (5 to 17)	32 (14 to 52)	0·08% (0·04 to 0·13)
	Latvia	0·69 (0·67 to 0·72)	62 (57 to 69)	8·75 (8·34 to 9·17)	737 (485 to 985)	2578 (2346 to 2813)	0·24 (0·23 to 0·25)	16 (6 to 27)	87 (48 to 128)	0·17% (0·09 to 0·24)
	Lithuania	0·68 (0·66 to 0·69)	53 (49 to 56)	12·3 (11·85 to 12·79)	0 (0 to 0)	2186 (1984 to 2401)	0·33 (0·31 to 0·35)	1 (−1 to 2)	2 (−1 to 5)	0·00% (−0·0 to 0·01)
	Moldova	0·64 (0·62 to 0·66)	17 (15 to 19)	7·79 (7·48 to 8·11)	1723 (1241 to 2196)	855 (760 to 966)	0·2 (0·19 to 0·21)	70 (56 to 84)	90 (71 to 112)	0·41% (0·32 to 0·51)
	Russia	0·63 (0·58 to 0·67)	33 (31 to 36)	13·75 (13·08 to 14·58)	0 (0 to 0)	1941 (1808 to 2089)	0·26 (0·25 to 0·27)	1237 (248 to 2429)	2403 (470 to 4834)	0·06% (0·01 to 0·12)
	Ukraine	0·63 (0·58 to 0·67)	22 (18 to 26)	7·02 (6·07 to 8·37)	33 590 (10 504 to 54 226)	694 (626 to 768)	0·26 (0·24 to 0·27)	501 (143 to 896)	1102 (431 to 1851)	0·25% (0·1 to 0·43)
**High income**		**..**	**..**	**11·79 (10·78 to 12·88)**	**249 199 (94 015 to 391 261)**	**..**	**0·2 (0·18 to 0·21)**	**12 493 (5931 to 18 151)**	**251 662 (141 111 to 362 659)**	**..**
Australasia		..	..	11·79 (11·34 to 12·25)	0 (0 to 0)	..	0·24 (0·22 to 0·26)	45 (6 to 78)	298 (36 to 531)	..
	Australia	0·82 (0·8 to 0·83)	154 (148 to 162)	11·95 (11·49 to 12·43)	0 (0 to 0)	7334 (6669 to 8087)	0·24 (0·22 to 0·26)	4 (−31 to 33)	31 (−205 to 248)	0·00% (−0·02 to 0·02)
	New Zealand	0·78 (0·76 to 0·8)	132 (120 to 146)	10·93 (10·16 to 11·63)	0 (0 to 0)	6479 (5973 to 7007)	0·21 (0·21 to 0·22)	41 (28 to 55)	267 (172 to 354)	0·15% (0·1 to 0·2)
High-income Asia-Pacific		..	..	22·92 (20·6 to 24·92)	0 (0 to 0)	..	0·18 (0·16 to 0·21)	3195 (626 to 5512)	34 384 (5618 to 66 955)	..
	Brunei	0·64 (0·62 to 0·68)	68 (54 to 83)	15·62 (13·29 to 19·64)	0 (0 to 0)	5260 (4199 to 6551)	0·19 (0·15 to 0·22)	3 (0 to 5)	15 (−2 to 32)	0·05% (−0·01 to 0·1)
	Japan	0·83 (0·81 to 0·84)	76 (67 to 88)	22·42 (19·46 to 24·99)	0 (0 to 0)	10 335 (8572 to 12 472)	0·16 (0·13 to 0·19)	3169 (642 to 5453)	34 143 (5602 to 66 285)	0·65% (0·11 to 1·27)
	Singapore	0·81 (0·78 to 0·84)	79 (65 to 95)	22·68 (19·12 to 27·38)	0 (0 to 0)	9132 (7516 to 11 044)	0·18 (0·15 to 0·21)	23 (−22 to 60)	226 (−194 to 657)	0·07% (−0·06 to 0·19)
	South Korea	0·81 (0·77 to 0·85)	50 (47 to 53)	24·33 (23·24 to 25·44)	0 (0 to 0)	4878 (4492 to 5261)	0·24 (0·23 to 0·26)	0 (0 to 0)	0 (0 to 0)	0·00% (0·00 to 0·00)
High-income North America		..	..	8·79 (7·79 to 9·87)	119 433 (27 473 to 197 248)	..	0·17 (0·15 to 0·18)	5014 (2949 to 6919)	166 930 (92 597 to 241 261)	..
	Canada	0·79 (0·78 to 0·81)	157 (148 to 167)	12·99 (12·23 to 13·69)	0 (0 to 0)	10 103 (8448 to 12 088)	0·19 (0·16 to 0·23)	302 (97 to 465)	3097 (615 to 5396)	0·18% (0·04 to 0·31)
	Greenland	0·64 (0·61 to 0·67)	..	8·7 (7·52 to 10·28)	15 (−4 to 29)	..	0·15 (0·12 to 0·18)	1 (1 to 1)	0 (0 to 0)	..
	USA	0·73 (0·71 to 0·74)	478 (415 to 548)	8·33 (7·25 to 9·52)	119 418 (27 474 to 197 217)	22 543 (20 547 to 24 392)	0·17 (0·15 to 0·18)	4710 (2677 to 6590)	163 833 (90 562 to 237 439)	0·86% (0·47 to 1·24)
Southern Latin America		..	..	5·92 (5·32 to 6·71)	77 103 (38 426 to 104 635)	..	0·12 (0·1 to 0·14)	2402 (1510 to 3199)	20 776 (13 868 to 27 692)	..
	Argentina	0·61 (0·6 to 0·63)	86 (71 to 101)	6·1 (5·28 to 7·18)	47 282 (12 243 to 72 301)	4211 (3507 to 5052)	0·12 (0·1 to 0·14)	1529 (822 to 2167)	10 799 (5745 to 16 146)	1·17% (0·62 to 1·76)
	Chile	0·71 (0·67 to 0·75)	131 (125 to 138)	5·48 (5·31 to 5·68)	26 005 (22 813 to 29 141)	5956 (5252 to 6721)	0·12 (0·11 to 0·14)	618 (397 to 806)	7178 (5414 to 8956)	1·60% (1·2 to 1·99)
	Uruguay	0·65 (0·63 to 0·66)	118 (100 to 139)	6·08 (5·29 to 7·06)	3816 (1301 to 5880)	9102 (7505 to 10 776)	0·08 (0·07 to 0·09)	255 (216 to 288)	2798 (1958 to 3614)	3·64% (2·55 to 4·7)
Western Europe		..	..	10·65 (9·86 to 11·58)	52 663 (6711 to 97 539)	..	0·24 (0·22 to 0·25)	1837 (233 to 3139)	29 275 (10 340 to 49 105)	..
	Andorra	0·81 (0·78 to 0·85)	367 (311 to 429)	10·72 (9·35 to 12·3)	0 (0 to 0)	15 054 (12 308 to 18 392)	0·24 (0·2 to 0·29)	0 (0 to 0)	0 (0 to 0)	0·00% (0·00 to 0·00)
	Austria	0·82 (0·8 to 0·84)	187 (179 to 196)	11·15 (10·71 to 11·61)	29 (−257 to 308)	5357 (5044 to 5663)	0·37 (0·35 to 0·39)	0 (0 to 0)	7 (−57 to 69)	0·00% (−0·01 to 0·02)
	Belgium	0·8 (0·78 to 0·82)	147 (131 to 162)	13·54 (12·39 to 15·04)	0 (0 to 0)	7869 (7298 to 8435)	0·24 (0·22 to 0·26)	5 (−15 to 22)	43 (−115 to 174)	0·01% (−0·02 to 0·03)
	Cyprus	0·78 (0·76 to 0·79)	110 (93 to 130)	8·51 (7·29 to 9·82)	246 (−39 to 524)	5938 (4956 to 7154)	0·15 (0·13 to 0·18)	16 (−1 to 31)	127 (17 to 248)	0·41% (0·05 to 0·79)
	Denmark	0·79 (0·77 to 0·82)	267 (256 to 281)	7·82 (7·55 to 8·09)	885 (280 to 1498)	9136 (8556 to 9721)	0·22 (0·21 to 0·23)	2 (−5 to 9)	250 (48 to 462)	0·09% (0·02 to 0·16)
	Finland	0·85 (0·84 to 0·87)	229 (218 to 240)	7·16 (6·84 to 7·47)	1777 (908 to 2645)	6627 (6175 to 7104)	0·24 (0·22 to 0·25)	0 (−1 to 1)	417 (208 to 641)	0·17% (0·09 to 0·26)
	France	0·81 (0·79 to 0·82)	170 (163 to 178)	11·44 (11·01 to 11·89)	0 (0 to 0)	6576 (6049 to 7063)	0·28 (0·26 to 0·31)	0 (0 to 0)	0 (0 to 0)	0·00% (0·00 to 0·00)
	Germany	0·79 (0·77 to 0·81)	159 (143 to 175)	13·86 (12·63 to 15·39)	0 (0 to 0)	6050 (5674 to 6445)	0·34 (0·33 to 0·36)	0 (0 to 0)	0 (0 to 0)	0·00% (0·00 to 0·00)
	Greece	0·79 (0·77 to 0·8)	122 (102 to 146)	6·75 (5·79 to 7·93)	7123 (1258 to 12 279)	3176 (2623 to 3857)	0·25 (0·21 to 0·3)	0 (0 to 0)	908 (132 to 1755)	0·30% (0·04 to 0·58)
	Iceland	0·85 (0·83 to 0·87)	180 (171 to 193)	10·14 (9·69 to 10·57)	0 (0 to 0)	9402 (8699 to 10 124)	0·18 (0·17 to 0·2)	2 (1 to 3)	19 (9 to 30)	0·11% (0·05 to 0·18)
	Ireland	0·8 (0·78 to 0·82)	170 (153 to 189)	13·09 (12·21 to 13·88)	0 (0 to 0)	10 189 (9131 to 11 362)	0·21 (0·19 to 0·22)	0 (−2 to 3)	4 (−25 to 31)	0·00% (−0·01 to 0·01)
	Israel	0·76 (0·73 to 0·8)	86 (73 to 99)	12·32 (10·77 to 14·4)	0 (0 to 0)	4183 (3879 to 4499)	0·24 (0·23 to 0·26)	0 (0 to 0)	0 (0 to 0)	0·00% (0·00 to 0·00)
	Italy	0·81 (0·79 to 0·83)	103 (91 to 115)	12·73 (11·46 to 14·42)	0 (0 to 0)	7259 (6625 to 7897)	0·17 (0·16 to 0·19)	546 (−33 to 1077)	4026 (−126 to 8322)	0·17% (−0·01 to 0·35)
	Luxembourg	0·83 (0·8 to 0·85)	274 (253 to 299)	10·79 (10·34 to 11·26)	0 (0 to 0)	12 262 (11 100 to 13 522)	0·22 (0·21 to 0·24)	0 (0 to 0)	0 (−1 to 1)	0·00% (0·00 to 0·00)
	Malta	0·77 (0·74 to 0·8)	76 (66 to 90)	18·82 (16·05 to 21·55)	0 (0 to 0)	7422 (6179 to 8997)	0·19 (0·15 to 0·23)	1 (−3 to 4)	6 (−22 to 30)	0·04% (−0·13 to 0·18)
	Netherlands	0·82 (0·8 to 0·84)	312 (282 to 343)	7·25 (6·67 to 7·89)	0 (0 to 0)	12 313 (10 701 to 14 114)	0·17 (0·15 to 0·2)	0 (0 to 0)	0 (0 to 0)	0·00% (0·00 to 0·00)
	Norway	0·84 (0·82 to 0·86)	413 (388 to 438)	7·21 (6·92 to 7·51)	1275 (374 to 2084)	10 060 (9375 to 10 820)	0·27 (0·26 to 0·29)	0 (−2 to 3)	540 (209 to 897)	0·14% (0·06 to 0·24)
	Portugal	0·77 (0·75 to 0·78)	139 (128 to 155)	7·01 (6·43 to 7·52)	4178 (1393 to 7291)	6610 (6121 to 7147)	0·14 (0·14 to 0·15)	291 (215 to 372)	2528 (1773 to 3340)	0·78% (0·55 to 1·04)
	Spain	0·83 (0·81 to 0·84)	124 (110 to 140)	10·6 (9·45 to 11·9)	0 (0 to 0)	8027 (7567 to 8480)	0·16 (0·15 to 0·17)	602 (366 to 842)	4841 (2829 to 7006)	0·27% (0·16 to 0·4)
	Sweden	0·83 (0·81 to 0·85)	402 (343 to 467)	5·7 (4·87 to 6·63)	11 494 (4594 to 17 687)	11 249 (9420 to 13 328)	0·19 (0·16 to 0·23)	61 (−91 to 179)	5473 (1771 to 9328)	1·08% (0·35 to 1·83)
	Switzerland	0·86 (0·82 to 0·89)	398 (369 to 428)	7·88 (7·36 to 8·41)	2280 (458 to 3955)	12 421 (11 587 to 13 301)	0·24 (0·22 to 0·25)	0 (0 to 0)	919 (197 to 1634)	0·17% (0·04 to 0·31)
	UK	0·77 (0·76 to 0·78)	243 (210 to 284)	7·01 (6·1 to 8·07)	23 375 (−7052 to 52 671)	9037 (7699 to 10 398)	0·18 (0·16 to 0·21)	311 (−547 to 945)	9166 (−1545 to 21 429)	0·32% (−0·05 to 0·74)
**Latin America and Caribbean**		**..**	**..**	**6·47 (5·79 to 7·22)**	**615 322 (410 489 to 803 049)**	**..**	**0·1 (0·09 to 0·11)**	**28 433 (23 133 to 32 514)**	**160 844 (121 779 to 198 897)**	**..**
Andean Latin America		..	..	7·78 (6·65 to 9·5)	11 992 (−10962 to 30 524)	..	0·1 (0·09 to 0·11)	2869 (2402 to 3267)	7866 (5691 to 9988)	..
	Bolivia	0·52 (0·49 to 0·56)	25 (19 to 30)	7·42 (6·21 to 9·26)	4481 (−1078 to 9010)	1576 (1264 to 1899)	0·1 (0·08 to 0·12)	542 (432 to 638)	976 (632 to 1321)	1·21% (0·78 to 1·64)
	Ecuador	0·61 (0·59 to 0·63)	54 (43 to 67)	7·86 (6·57 to 9·5)	2906 (−4405 to 8852)	3297 (2890 to 3777)	0·1 (0·1 to 0·11)	810 (759 to 863)	2857 (2265 to 3463)	1·49% (1·18 to 1·81)
	Peru	0·66 (0·63 to 0·69)	38 (29 to 45)	7·86 (6·72 to 9·76)	4605 (−8218 to 15 468)	2499 (2011 to 3035)	0·09 (0·08 to 0·11)	1518 (1145 to 1831)	4033 (2502 to 5623)	0·95% (0·59 to 1·33)
Caribbean		..	..	4·26 (3·66 to 4·9)	113 711 (93 323 to 132 567)	..	0·1 (0·08 to 0·11)	2427 (1909 to 2836)	12 309 (9126 to 15 807)	..
	Antigua and Barbuda	0·63 (0·6 to 0·65)	111 (92 to 136)	5·29 (4·47 to 6·18)	157 (99 to 209)	4667 (3697 to 5695)	0·1 (0·08 to 0·12)	4 (3 to 5)	38 (25 to 52)	1·71% (1·13 to 2·32)
	The Bahamas	0·6 (0·58 to 0·63)	136 (112 to 167)	6·13 (5·18 to 7·21)	423 (194 to 609)	4931 (3892 to 6170)	0·13 (0·11 to 0·16)	12 (8 to 16)	122 (68 to 172)	1·24% (0·7 to 1·75)
	Barbados	0·63 (0·6 to 0·65)	164 (137 to 197)	3·34 (2·85 to 3·86)	887 (770 to 997)	2790 (2236 to 3425)	0·15 (0·13 to 0·18)	6 (2 to 10)	165 (121 to 213)	3·45% (2·52 to 4·44)
	Belize	0·56 (0·52 to 0·58)	55 (44 to 67)	3·81 (3·19 to 4·5)	996 (784 to 1183)	2368 (1883 to 2884)	0·07 (0·06 to 0·09)	25 (20 to 28)	114 (83 to 145)	3·49% (2·54 to 4·44)
	Bermuda	0·73 (0·71 to 0·75)	..	5·96 (5·06 to 6·97)	88 (49 to 124)	..	0·13 (0·11 to 0·15)	2 (1 to 3)	0 (0 to 0)	..
	Cuba	0·67 (0·66 to 0·69)	90 (72 to 113)	4·29 (3·65 to 4·91)	27 201 (21 141 to 33 629)	2760 (2160 to 3595)	0·11 (0·09 to 0·13)	527 (327 to 704)	3992 (2639 to 5602)	3·71% (2·45 to 5·2)
	Dominica	0·57 (0·54 to 0·59)	33 (27 to 40)	7·58 (6·41 to 8·85)	54 (22 to 86)	1995 (1587 to 2447)	0·1 (0·09 to 0·12)	4 (3 to 5)	9 (6 to 13)	1·11% (0·71 to 1·57)
	Dominican Republic	0·62 (0·59 to 0·65)	92 (77 to 112)	4·5 (3·84 to 5·26)	24 490 (18 527 to 29 940)	2760 (2180 to 3365)	0·12 (0·1 to 0·14)	388 (255 to 494)	3393 (2292 to 4591)	2·04% (1·38 to 2·75)
	Grenada	0·55 (0·52 to 0·57)	66 (54 to 81)	4·55 (3·88 to 5·35)	248 (185 to 303)	2435 (1949 to 2959)	0·1 (0·08 to 0·12)	6 (4 to 7)	31 (22 to 41)	2·07% (1·46 to 2·73)
	Guyana	0·51 (0·48 to 0·53)	20 (16 to 25)	6·09 (5·08 to 7·13)	1012 (517 to 1479)	1796 (1393 to 2278)	0·06 (0·05 to 0·07)	62 (55 to 68)	133 (92 to 177)	2·45% (1·7 to 3·24)
	Haiti	0·4 (0·37 to 0·44)	14 (11 to 17)	2·95 (2·46 to 3·44)	40 783 (35 773 to 45 780)	558 (447 to 679)	0·07 (0·06 to 0·08)	882 (747 to 981)	1065 (818 to 1324)	5·32% (4·08 to 6·61)
	Jamaica	0·61 (0·58 to 0·64)	72 (59 to 87)	2·75 (2·34 to 3·18)	10 051 (9014 to 11 074)	4186 (3349 to 5145)	0·04 (0·03 to 0·05)	281 (261 to 300)	1920 (1554 to 2315)	7·52% (6·09 to 9·07)
	Puerto Rico	0·67 (0·65 to 0·69)	..	8·71 (7·46 to 10·12)	1528 (−178 to 2836)	..	0·12 (0·1 to 0·14)	153 (96 to 202)	0 (0 to 0)	..
	Saint Lucia	0·59 (0·57 to 0·62)	53 (43 to 64)	5·98 (5·08 to 6·96)	265 (153 to 368)	2565 (2036 to 3158)	0·1 (0·08 to 0·12)	9 (7 to 12)	39 (26 to 53)	1·75% (1·14 to 2·37)
	Saint Vincent and the Grenadines	0·55 (0·53 to 0·57)	49 (41 to 60)	4·24 (3·62 to 4·96)	275 (212 to 329)	1799 (1457 to 2211)	0·09 (0·08 to 0·11)	6 (5 to 8)	25 (18 to 33)	1·99% (1·4 to 2·6)
	Suriname	0·56 (0·53 to 0·58)	83 (68 to 101)	4·49 (3·81 to 5·21)	1226 (918 to 1518)	3128 (2467 to 3886)	0·09 (0·08 to 0·11)	29 (20 to 35)	194 (133 to 257)	2·43% (1·66 to 3·22)
	Trinidad and Tobago	0·58 (0·55 to 0·61)	239 (197 to 288)	3·73 (3·18 to 4·36)	3899 (3215 to 4531)	4557 (3604 to 5575)	0·15 (0·12 to 0·18)	27 (10 to 40)	1068 (750 to 1421)	2·44% (1·72 to 3·25)
	Virgin Islands	0·62 (0·59 to 0·65)	..	5·98 (5·03 to 7·05)	127 (60 to 184)	..	0·14 (0·11 to 0·16)	4 (2 to 6)	0 (0 to 0)	..
Central Latin America		..	..	5·94 (5·09 to 6·85)	380 121 (252 615 to 495 549)	..	0·08 (0·07 to 0·09)	16 581 (14 173 to 18 455)	104 914 (81 488 to 128 076)	..
	Colombia	0·65 (0·63 to 0·67)	41 (34 to 50)	8·86 (7·54 to 10·35)	6139 (−14333 to 25 382)	5897 (5084 to 6673)	0·05 (0·05 to 0·05)	4122 (3892 to 4313)	24 627 (20 410 to 28 306)	3·51% (2·91 to 4·03)
	Costa Rica	0·69 (0·67 to 0·71)	136 (111 to 166)	4·38 (3·71 to 5·15)	11 198 (8563 to 13 699)	6305 (5007 to 7685)	0·07 (0·06 to 0·09)	316 (258 to 359)	3556 (2600 to 4557)	4·38% (3·2 to 5·61)
	El Salvador	0·63 (0·6 to 0·65)	88 (78 to 100)	2·61 (2·38 to 2·82)	24 501 (23 066 to 25 961)	6919 (5851 to 8226)	0·03 (0·02 to 0·03)	672 (646 to 698)	6833 (5862 to 7859)	12·45% (10·68 to 14·32)
	Guatemala	0·54 (0·5 to 0·58)	32 (25 to 39)	5·98 (4·97 to 7·24)	18 928 (2626 to 31 078)	2220 (1764 to 2695)	0·07 (0·06 to 0·08)	1141 (944 to 1303)	3168 (2164 to 4267)	2·38% (1·63 to 3·21)
	Honduras	0·55 (0·5 to 0·59)	26 (21 to 32)	5·59 (4·63 to 6·59)	13 067 (6947 to 18 450)	1696 (1311 to 2134)	0·07 (0·06 to 0·09)	567 (456 to 657)	1319 (868 to 1774)	3·09% (2·03 to 4·16)
	Mexico	0·6 (0·59 to 0·62)	95 (80 to 112)	4·99 (4·29 to 5·77)	236 409 (160 140 to 305 056)	4577 (3592 to 5576)	0·08 (0·07 to 0·1)	7645 (5842 to 8954)	58 217 (40 783 to 76 009)	2·42% (1·7 to 3·16)
	Nicaragua	0·65 (0·62 to 0·68)	19 (16 to 24)	8·49 (7·13 to 9·97)	612 (−2376 to 2991)	1907 (1511 to 2342)	0·07 (0·06 to 0·09)	410 (336 to 468)	800 (518 to 1085)	2·34% (1·52 to 3·17)
	Panama	0·63 (0·6 to 0·65)	54 (44 to 65)	13·79 (11·76 to 15·98)	0 (0 to 0)	4095 (3338 to 5056)	0·14 (0·12 to 0·16)	72 (18 to 112)	303 (74 to 560)	0·32% (0·08 to 0·6)
	Venezuela	0·6 (0·57 to 0·63)	46 (37 to 58)	4·8 (4·01 to 5·65)	69 267 (52 395 to 84 376)	1717 (1376 to 2124)	0·1 (0·08 to 0·12)	1637 (1408 to 1841)	6091 (4493 to 7899)	1·40% (1·03 to 1·81)
Tropical Latin America		..	..	7·27 (6·79 to 7·79)	109 498 (63 846 to 156 000)	..	0·12 (0·11 to 0·14)	6556 (4321 to 8378)	35 756 (22 895 to 48 367)	..
	Brazil	0·62 (0·61 to 0·63)	87 (77 to 97)	7·33 (6·85 to 7·85)	98 890 (56 314 to 140 448)	4054 (3403 to 4761)	0·12 (0·11 to 0·14)	6167 (3989 to 7942)	33 942 (21 482 to 46 134)	1·05% (0·66 to 1·42)
	Paraguay	0·56 (0·54 to 0·58)	58 (47 to 71)	5·51 (4·68 to 6·51)	10 607 (5839 to 14 711)	3009 (2402 to 3707)	0·09 (0·07 to 0·1)	389 (304 to 455)	1813 (1234 to 2409)	2·80% (1·91 to 3·72)
**North Africa and Middle East**		**..**	**..**	**7·13 (6·31 to 8·23)**	**676 422 (469 769 to 838 873)**	**..**	**0·12 (0·11 to 0·14)**	**23 918 (17 677 to 28 552)**	**119 283 (79 039 to 157 729)**	**..**
	Afghanistan	0·31 (0·28 to 0·36)	6 (5 to 8)	5·61 (4·67 to 6·9)	63 000 (28 873 to 87 927)	599 (457 to 769)	0·08 (0·07 to 0·1)	2490 (1879 to 2993)	1930 (1226 to 2681)	3·59% (2·28 to 4·99)
	Algeria	0·64 (0·61 to 0·66)	70 (57 to 85)	5·61 (4·74 to 6·66)	47 746 (26 082 to 66 884)	3787 (2864 to 4793)	0·12 (0·1 to 0·16)	1108 (502 to 1609)	7783 (4037 to 11 714)	1·27% (0·66 to 1·92)
	Bahrain	0·68 (0·65 to 0·71)	103 (79 to 127)	10·69 (8·81 to 13·26)	9 (−11 to 27)	20 526 (15 697 to 25 631)	0·06 (0·05 to 0·07)	87 (72 to 98)	1809 (1134 to 2444)	2·63% (1·65 to 3·55)
	Egypt	0·61 (0·58 to 0·63)	18 (14 to 21)	9·29 (7·89 to 11·49)	1275 (−4466 to 6068)	1087 (860 to 1345)	0·18 (0·15 to 0·21)	1492 (553 to 2250)	1697 (534 to 2871)	0·16% (0·05 to 0·26)
	Iran	0·67 (0·64 to 0·71)	67 (54 to 81)	7·23 (6·21 to 8·57)	21 686 (−20514 to 53 980)	7217 (5582 to 9069)	0·08 (0·07 to 0·1)	4995 (3755 to 5906)	38 140 (22 497 to 54 220)	2·44% (1·44 to 3·46)
	Iraq	0·52 (0·49 to 0·56)	74 (58 to 94)	2·33 (1·94 to 2·77)	159 645 (142 270 to 174 339)	2387 (1762 to 3169)	0·09 (0·07 to 0·11)	2264 (1420 to 2905)	17 499 (12 521 to 22 768)	2·67% (1·91 to 3·47)
	Jordan	0·66 (0·62 to 0·7)	54 (43 to 67)	4·73 (3·97 to 5·69)	15 825 (9757 to 20 581)	1240 (962 to 1571)	0·25 (0·2 to 0·3)	28 (11 to 43)	907 (464 to 1376)	1·06% (0·54 to 1·6)
	Kuwait	0·73 (0·69 to 0·77)	149 (117 to 183)	8·64 (7·24 to 10·59)	217 (−427 to 747)	10 690 (7952 to 14 003)	0·13 (0·1 to 0·16)	69 (22 to 108)	809 (205 to 1508)	0·28% (0·07 to 0·52)
	Lebanon	0·74 (0·72 to 0·76)	85 (67 to 105)	5·16 (4·34 to 6·15)	12 180 (7591 to 15 958)	5140 (4048 to 6350)	0·1 (0·08 to 0·12)	315 (228 to 388)	2701 (1795 to 3617)	2·83% (1·88 to 3·79)
	Libya	0·65 (0·62 to 0·67)	31 (24 to 39)	7·93 (6·81 to 9·44)	1932 (−1693 to 4475)	2182 (1628 to 2884)	0·13 (0·11 to 0·16)	158 (64 to 240)	427 (140 to 751)	1·15% (0·38 to 2·03)
	Morocco	0·58 (0·56 to 0·61)	34 (27 to 42)	4·78 (3·97 to 5·83)	77 838 (52 509 to 97 910)	3715 (2898 to 4670)	0·05 (0·04 to 0·07)	2867 (2547 to 3136)	13 468 (9996 to 17 311)	4·88% (3·62 to 6·27)
	Oman	0·75 (0·73 to 0·77)	80 (62 to 101)	9·14 (7·59 to 11·27)	19 (−75 to 104)	14 217 (10 795 to 18 105)	0·06 (0·05 to 0·07)	300 (250 to 339)	4324 (2721 to 6075)	2·08% (1·31 to 2·93)
	Palestine	0·59 (0·57 to 0·61)	12 (10 to 15)	5·21 (4·37 to 6·19)	4803 (896 to 8395)	964 (721 to 1298)	0·08 (0·07 to 0·11)	278 (170 to 361)	337 (166 to 526)	1·60% (0·79 to 2·51)
	Qatar	0·77 (0·72 to 0·82)	118 (89 to 151)	14·61 (11·97 to 17·89)	0 (0 to 0)	13 771 (9948 to 18 967)	0·13 (0·1 to 0·17)	29 (5 to 47)	431 (71 to 834)	0·16% (0·03 to 0·31)
	Saudi Arabia	0·72 (0·7 to 0·74)	134 (110 to 161)	10·55 (9·09 to 12·28)	637 (−1938 to 3394)	13 062 (10 279 to 16 358)	0·12 (0·1 to 0·14)	802 (437 to 1111)	10 829 (4855 to 17 355)	0·62% (0·28 to 0·99)
	Sudan	0·47 (0·45 to 0·49)	15 (12 to 20)	5·5 (4·62 to 6·7)	73 204 (34 725 to 101 941)	1197 (898 to 1595)	0·09 (0·07 to 0·11)	2331 (1590 to 2886)	4013 (2376 to 5801)	2·22% (1·32 to 3·21)
	Syria	0·67 (0·65 to 0·7)	14 (11 to 17)	5·06 (4·27 to 6·03)	61 729 (46 076 to 74 829)	673 (521 to 866)	0·13 (0·1 to 0·15)	846 (486 to 1160)	1435 (904 to 1997)	1·51% (0·95 to 2·1)
	Tunisia	0·66 (0·63 to 0·69)	40 (32 to 48)	7·28 (6·22 to 8·71)	3098 (−1641 to 7151)	3401 (2684 to 4247)	0·1 (0·09 to 0·12)	516 (360 to 649)	1930 (1136 to 2876)	1·46% (0·86 to 2·18)
	Turkey	0·67 (0·64 to 0·7)	34 (30 to 39)	12·18 (11·65 to 12·72)	0 (0 to 0)	2190 (1886 to 2518)	0·22 (0·21 to 0·23)	358 (213 to 499)	790 (444 to 1148)	0·04% (0·02 to 0·06)
	United Arab Emirates	0·66 (0·62 to 0·7)	137 (104 to 173)	8·49 (6·81 to 10·95)	402 (−827 to 1492)	13 130 (10 010 to 16 708)	0·09 (0·08 to 0·12)	272 (124 to 384)	3766 (1358 to 6413)	0·54% (0·19 to 0·92)
	Yemen	0·44 (0·41 to 0·47)	18 (14 to 23)	2·5 (2·22 to 2·81)	131 176 (121 549 to 140 001)	804 (581 to 1108)	0·07 (0·06 to 0·09)	2312 (1786 to 2718)	4258 (3213 to 5638)	6·16% (4·65 to 8·16)
**South Asia**		**..**	**..**	**4·67 (3·93 to 5·48)**	**4 027 525 (3 113 082 to 4 896 398)**	**..**	**0·07 (0·06 to 0·08)**	**120 653 (108 685 to 130 918)**	**145 297 (113 640 to 177 819)**	**..**
	Bangladesh	0·55 (0·52 to 0·57)	5 (4 to 6)	4·39 (3·69 to 5·18)	422 808 (323 953 to 514 241)	326 (257 to 410)	0·07 (0·06 to 0·08)	12 253 (10 232 to 13 694)	6129 (4449 to 7797)	1·01% (0·73 to 1·29)
	Bhutan	0·56 (0·52 to 0·59)	14 (11 to 17)	5·91 (5·0 to 6·92)	1100 (605 to 1507)	842 (667 to 1078)	0·1 (0·08 to 0·12)	36 (24 to 45)	47 (29 to 65)	0·69% (0·42 to 0·95)
	India	0·5 (0·48 to 0·51)	14 (11 to 17)	4·59 (3·82 to 5·4)	3 217 963 (2 493 450 to 3 913 540)	875 (731 to 1048)	0·07 (0·07 to 0·08)	92 448 (84 033 to 100 097)	127 541 (100 334 to 156 296)	1·45% (1·14 to 1·77)
	Nepal	0·52 (0·49 to 0·55)	13 (11 to 16)	2·65 (2·24 to 3·07)	113 559 (102 332 to 124 285)	518 (411 to 660)	0·07 (0·06 to 0·08)	2207 (1843 to 2507)	2636 (2060 to 3285)	3·50% (2·73 to 4·36)
	Pakistan	0·43 (0·4 to 0·46)	6 (5 to 7)	5·76 (4·89 to 6·74)	272 095 (150 396 to 379 250)	525 (399 to 667)	0·07 (0·06 to 0·08)	13 709 (11 176 to 15 491)	8944 (6185 to 11 883)	0·88% (0·61 to 1·16)
**Southeast Asia, east Asia, and Oceania**		**..**	**..**	**6·69 (6·11 to 7·29)**	**1 709 453 (1 060 336 to 2 339 121)**	**..**	**0·14 (0·13 to 0·15)**	**71 154 (62 987 to 78 765)**	**366 220 (288 163 to 449 734)**	**..**
East Asia		..	..	7·1 (6·6 to 7·59)	753 139 (400 129 to 1 114 796)	..	0·18 (0·17 to 0·2)	12 175 (6354 to 17 677)	65 575 (39 749 to 93 221)	..
	China	0·69 (0·68 to 0·7)	59 (54 to 65)	6·73 (6·28 to 7·17)	751 600 (401 537 to 1 113 190)	1609 (1448 to 1790)	0·18 (0·17 to 0·2)	11 360 (5698 to 16 459)	63 976 (38 863 to 90 940)	0·29% (0·18 to 0·42)
	North Korea	0·56 (0·54 to 0·59)	5 (4 to 6)	7·98 (6·79 to 9·38)	1539 (−7288 to 7371)	247 (200 to 300)	0·14 (0·12 to 0·16)	579 (159 to 927)	157 (32 to 287)	0·32% (0·07 to 0·58)
	Taiwan (province of China)	0·72 (0·7 to 0·75)	45 (37 to 55)	31·05 (26·72 to 36·07)	0 (0 to 0)	5845 (4615 to 7279)	0·17 (0·14 to 0·2)	236 (−13 to 443)	1442 (−19 to 3069)	0·13% (−0·0 to 0·28)
Oceania		..	..	6·17 (5·19 to 7·76)	13 061 (2801 to 19 123)	..	0·07 (0·06 to 0·09)	752 (620 to 860)	598 (407 to 776)	..
	American Samoa	0·57 (0·54 to 0·6)	..	9·87 (8·28 to 11·83)	2 (−2 to 6)	..	0·09 (0·08 to 0·11)	4 (3 to 5)	0 (0 to 0)	..
	Federated States of Micronesia	0·45 (0·41 to 0·5)	16 (13 to 19)	8·2 (6·88 to 9·65)	40 (−18 to 102)	1153 (931 to 1393)	0·09 (0·07 to 0·11)	6 (5 to 7)	8 (5 to 11)	2·32% (1·43 to 3·19)
	Fiji	0·47 (0·43 to 0·51)	19 (15 to 23)	8·78 (7·32 to 10·99)	233 (−83 to 516)	1509 (1208 to 1794)	0·08 (0·07 to 0·1)	58 (47 to 67)	93 (60 to 125)	1·13% (0·73 to 1·53)
	Guam	0·59 (0·56 to 0·62)	..	10·14 (8·73 to 11·96)	3 (−8 to 12)	..	0·1 (0·08 to 0·12)	8 (6 to 11)	0 (0 to 0)	..
	Kiribati	0·41 (0·38 to 0·44)	12 (9 to 15)	6·55 (5·43 to 7·82)	88 (11 to 160)	894 (702 to 1124)	0·07 (0·06 to 0·09)	8 (6 to 9)	8 (5 to 11)	3·75% (2·43 to 5·11)
	Marshall Islands	0·44 (0·4 to 0·48)	35 (27 to 42)	7·12 (5·89 to 8·91)	41 (−9 to 81)	2591 (2096 to 3121)	0·07 (0·06 to 0·09)	5 (4 to 6)	14 (9 to 19)	5·23% (3·41 to 7·09)
	Northern Mariana Islands	0·66 (0·63 to 0·69)	..	9·87 (8·18 to 12·47)	0 (−1 to 1)	..	0·09 (0·07 to 0·11)	6 (4 to 7)	0 (0 to 0)	..
	Papua New Guinea	0·39 (0·35 to 0·43)	8 (6 to 10)	5·68 (4·77 to 7·26)	11 686 (2535 to 16 966)	512 (419 to 626)	0·07 (0·06 to 0·09)	576 (474 to 664)	397 (263 to 530)	1·59% (1·06 to 2·13)
	Samoa	0·48 (0·45 to 0·51)	20 (16 to 25)	7·61 (6·31 to 9·23)	50 (−36 to 134)	1475 (1184 to 1787)	0·08 (0·07 to 0·1)	12 (9 to 14)	19 (12 to 26)	1·67% (1·04 to 2·34)
	Solomon Islands	0·4 (0·37 to 0·44)	9 (7 to 11)	6·18 (5·07 to 7·34)	652 (261 to 1022)	693 (549 to 851)	0·07 (0·06 to 0·08)	45 (38 to 51)	38 (26 to 50)	3·15% (2·14 to 4·18)
	Tonga	0·54 (0·51 to 0·57)	16 (13 to 19)	8·09 (6·86 to 9·81)	22 (−32 to 63)	1111 (915 to 1328)	0·09 (0·07 to 0·1)	6 (5 to 7)	7 (5 to 10)	1·26% (0·8 to 1·73)
	Vanuatu	0·39 (0·35 to 0·42)	10 (7 to 12)	6·37 (5·33 to 7·83)	244 (−5 to 403)	641 (500 to 814)	0·08 (0·06 to 0·09)	18 (14 to 21)	14 (9 to 20)	1·78% (1·12 to 2·46)
Southeast Asia		..	..	5·73 (4·82 to 6·78)	943 254 (591 879 to 1 254 765)	..	0·04 (0·03 to 0·05)	58 227 (54 094 to 61 475)	300 047 (233 607 to 370 170)	..
	Cambodia	0·5 (0·48 to 0·52)	19 (16 to 22)	5·1 (4·55 to 5·76)	27 443 (19 330 to 34 554)	2954 (2356 to 3614)	0·03 (0·02 to 0·03)	1640 (1554 to 1723)	5390 (4243 to 6752)	8·95% (7·04 to 11·21)
	Indonesia	0·5 (0·49 to 0·52)	38 (30 to 48)	5·83 (4·7 to 7·24)	295 299 (93 244 to 446 300)	3996 (3056 to 5086)	0·04 (0·03 to 0·05)	20 694 (18 605 to 22 305)	95 523 (68 862 to 127 138)	3·13% (2·26 to 4·17)
	Laos	0·44 (0·41 to 0·46)	17 (14 to 21)	4·48 (3·74 to 5·3)	12 330 (7785 to 16 551)	2485 (1989 to 3082)	0·02 (0·02 to 0·03)	728 (689 to 768)	2038 (1598 to 2559)	4·19% (3·29 to 5·27)
	Malaysia	0·64 (0·63 to 0·66)	53 (44 to 63)	12·69 (10·7 to 14·84)	0 (0 to 0)	11 232 (9156 to 13 672)	0·04 (0·04 to 0·05)	2447 (2230 to 2638)	27 741 (20 737 to 35 747)	3·24% (2·42 to 4·17)
	Maldives	0·72 (0·69 to 0·76)	174 (137 to 218)	6·01 (5·04 to 7·11)	459 (192 to 687)	20 651 (16 123 to 25 683)	0·04 (0·03 to 0·04)	33 (31 to 35)	767 (564 to 984)	12·98% (9·54 to 16·65)
	Mauritius	0·65 (0·63 to 0·67)	87 (72 to 105)	6·78 (5·73 to 7·96)	851 (−14 to 1571)	10 051 (8136 to 12 381)	0·04 (0·03 to 0·05)	121 (111 to 129)	1297 (967 to 1675)	4·78% (3·56 to 6·17)
	Myanmar	0·5 (0·47 to 0·52)	26 (21 to 32)	5·73 (4·82 to 6·77)	69 602 (33 044 to 101 798)	4124 (3287 to 5176)	0·03 (0·02 to 0·03)	5260 (4990 to 5521)	23 664 (18 140 to 30 354)	7·05% (5·4 to 9·04)
	Philippines	0·5 (0·47 to 0·52)	24 (20 to 29)	6·94 (5·83 to 8·1)	61 728 (−3412 to 114 239)	4731 (3826 to 5748)	0·03 (0·02 to 0·03)	10 025 (9471 to 10 537)	49 031 (37 770 to 61 662)	5·98% (4·61 to 7·52)
	Sri Lanka	0·69 (0·65 to 0·72)	28 (23 to 33)	6·38 (5·41 to 7·46)	19 598 (7286 to 30 838)	3268 (2639 to 3977)	0·04 (0·03 to 0·05)	1979 (1825 to 2115)	7054 (5317 to 8942)	2·72% (2·05 to 3·45)
	Seychelles	0·59 (0·57 to 0·62)	99 (81 to 120)	5·22 (4·5 to 5·95)	141 (84 to 196)	9062 (7008 to 11 218)	0·04 (0·03 to 0·05)	9 (8 to 10)	96 (72 to 123)	3·40% (2·54 to 4·33)
	Thailand	0·68 (0·66 to 0·7)	85 (71 to 102)	3·7 (3·13 to 4·29)	194 354 (161 103 to 226 590)	5170 (4124 to 6381)	0·04 (0·04 to 0·05)	6255 (5714 to 6751)	49 533 (38 549 to 61 666)	4·32% (3·36 to 5·37)
	Timor-Leste	0·46 (0·42 to 0·51)	7 (6 to 9)	6·53 (5·49 to 7·85)	946 (44 to 1620)	1114 (874 to 1424)	0·03 (0·03 to 0·04)	111 (103 to 118)	133 (99 to 173)	3·40% (2·53 to 4·43)
	Vietnam	0·61 (0·59 to 0·64)	41 (35 to 50)	3·77 (3·17 to 4·38)	260 504 (214 653 to 306 356)	2981 (2422 to 3641)	0·04 (0·03 to 0·05)	8926 (8260 to 9545)	37 782 (29 957 to 46 284)	6·10% (4·83 to 7·47)
**Sub-Saharan Africa**		**..**	**..**	**3·77 (3·22 to 4·42)**	**3 020 640 (2 414 437 to 3 539 543)**	**..**	**0·06 (0·05 to 0·07)**	**87 442 (76 512 to 95 734)**	**120 403 (92 668 to 146 043)**	**..**
Central sub-Saharan Africa		..	..	3·5 (2·92 to 4·23)	393 978 (301 686 to 465 275)	..	0·07 (0·06 to 0·09)	9590 (7859 to 11 045)	4960 (3496 to 6437)	..
	Angola	0·45 (0·4 to 0·49)	17 (13 to 21)	4·07 (3·36 to 4·76)	63 953 (46 328 to 81 532)	676 (529 to 864)	0·08 (0·06 to 0·09)	1717 (1302 to 2081)	2278 (1559 to 3097)	1·19% (0·81 to 1·62)
	Central African Republic	0·3 (0·26 to 0·35)	2 (2 to 3)	2·87 (2·36 to 3·66)	25 402 (20 393 to 28 715)	87 (68 to 106)	0·06 (0·05 to 0·07)	589 (507 to 653)	104 (78 to 130)	2·99% (2·25 to 3·72)
	Congo (Brazzaville)	0·47 (0·43 to 0·51)	13 (10 to 16)	4·88 (4·12 to 5·96)	10 362 (6201 to 13 219)	552 (437 to 683)	0·09 (0·08 to 0·11)	282 (203 to 349)	297 (193 to 396)	1·07% (0·69 to 1·42)
	Democratic Republic of the Congo	0·43 (0·41 to 0·46)	3 (2 to 4)	3·26 (2·68 to 4·0)	290 433 (227 403 to 340 120)	128 (101 to 158)	0·07 (0·06 to 0·08)	6881 (5634 to 7924)	1785 (1297 to 2272)	2·24% (1·63 to 2·85)
	Equatorial Guinea	0·52 (0·45 to 0·6)	81 (64 to 105)	5·67 (4·75 to 6·85)	1471 (691 to 2070)	3478 (2649 to 4457)	0·1 (0·08 to 0·11)	47 (34 to 59)	293 (181 to 416)	1·05% (0·65 to 1·49)
	Gabon	0·5 (0·46 to 0·53)	38 (30 to 46)	5·75 (4·79 to 7·02)	2358 (798 to 3489)	1447 (1170 to 1784)	0·11 (0·09 to 0·13)	75 (43 to 100)	203 (109 to 294)	0·64% (0·34 to 0·93)
Eastern sub-Saharan Africa		..	..	4·28 (3·67 to 5·01)	996 828 (743 228 to 1 207 013)	..	0·05 (0·05 to 0·06)	36 696 (33 170 to 39 410)	33 354 (26 174 to 40 169)	..
	Burundi	0·43 (0·41 to 0·46)	2 (2 to 3)	5·98 (5·02 to 7·3)	11 871 (3913 to 18 542)	249 (199 to 308)	0·05 (0·04 to 0·06)	1158 (1015 to 1274)	321 (231 to 419)	3·46% (2·48 to 4·51)
	Comoros	0·45 (0·42 to 0·48)	8 (6 to 9)	5·91 (4·89 to 7·38)	955 (243 to 1488)	265 (207 to 329)	0·15 (0·12 to 0·18)	15 (4 to 23)	12 (5 to 18)	0·95% (0·39 to 1·49)
	Djibouti	0·46 (0·42 to 0·51)	10 (8 to 12)	4·89 (4·06 to 6·02)	1304 (408 to 1940)	622 (497 to 754)	0·06 (0·05 to 0·08)	70 (57 to 79)	57 (39 to 76)	1·63% (1·1 to 2·18)
	Eritrea	0·39 (0·37 to 0·42)	2 (2 to 3)	5·08 (4·22 to 6·0)	9630 (5819 to 13 329)	170 (131 to 216)	0·06 (0·05 to 0·08)	408 (344 to 462)	94 (64 to 127)	1·36% (0·92 to 1·82)
	Ethiopia	0·4 (0·37 to 0·43)	6 (5 to 8)	3·94 (3·28 to 4·61)	273 222 (206 661 to 334 516)	667 (531 to 851)	0·03 (0·03 to 0·04)	10 888 (10 182 to 11 519)	9137 (6908 to 11 609)	4·93% (3·73 to 6·26)
	Kenya	0·55 (0·52 to 0·58)	14 (11 to 16)	4·98 (4·24 to 5·94)	98 404 (62 012 to 127 388)	1048 (846 to 1245)	0·05 (0·04 to 0·06)	4086 (3634 to 4445)	5644 (4194 to 7015)	3·66% (2·72 to 4·55)
	Madagascar	0·39 (0·36 to 0·42)	5 (4 to 6)	4·19 (3·44 to 5·3)	60 315 (32 355 to 79 371)	386 (308 to 490)	0·05 (0·04 to 0·06)	2491 (2225 to 2719)	1290 (925 to 1685)	3·46% (2·48 to 4·52)
	Malawi	0·49 (0·46 to 0·53)	11 (10 to 14)	2·91 (2·39 to 3·48)	80 086 (68 235 to 90 326)	601 (489 to 710)	0·05 (0·04 to 0·06)	2061 (1859 to 2214)	2175 (1721 to 2623)	10·66% (8·44 to 12·86)
	Mozambique	0·46 (0·43 to 0·49)	6 (5 to 7)	3·53 (2·93 to 4·27)	111 554 (86 778 to 131 250)	386 (309 to 472)	0·04 (0·04 to 0·05)	3467 (3119 to 3768)	1993 (1525 to 2463)	5·44% (4·16 to 6·72)
	Rwanda	0·52 (0·49 to 0·54)	8 (7 to 8)	6·41 (5·86 to 7·0)	9032 (3988 to 13 555)	1378 (1106 to 1718)	0·03 (0·02 to 0·04)	1267 (1188 to 1341)	1830 (1389 to 2351)	7·73% (5·86 to 9·93)
	Somalia	0·27 (0·24 to 0·3)	3 (2 to 4)	4·21 (3·49 to 5·13)	31 363 (22 963 to 38 164)	202 (158 to 251)	0·05 (0·04 to 0·07)	1007 (886 to 1104)	298 (221 to 378)	5·29% (3·92 to 6·7)
	South Sudan	0·35 (0·31 to 0·4)	9 (7 to 10)	2·81 (2·34 to 3·47)	53 687 (42 287 to 62 028)	439 (348 to 537)	0·04 (0·04 to 0·06)	1594 (1410 to 1743)	1168 (886 to 1439)	2·69% (2·04 to 3·32)
	Tanzania	0·49 (0·46 to 0·51)	9 (8 to 11)	5·46 (4·54 to 6·56)	82 667 (28 247 to 127 002)	415 (328 to 515)	0·1 (0·08 to 0·12)	2717 (1706 to 3547)	1949 (1020 to 2817)	1·24% (0·65 to 1·79)
	Uganda	0·44 (0·41 to 0·46)	11 (10 to 12)	4·17 (3·85 to 4·51)	104 655 (89 660 to 119 680)	780 (658 to 909)	0·05 (0·04 to 0·06)	4041 (3717 to 4317)	4312 (3555 to 5105)	5·07% (4·18 to 6·0)
	Zambia	0·45 (0·41 to 0·49)	26 (20 to 32)	2·97 (2·42 to 3·68)	68 083 (55 857 to 77 098)	904 (721 to 1103)	0·07 (0·06 to 0·08)	1426 (1181 to 1625)	3072 (2292 to 3875)	4·55% (3·4 to 5·74)
Southern sub-Saharan Africa		..	..	3·32 (2·82 to 3·9)	315 824 (272 681 to 351 952)	..	0·13 (0·1 to 0·16)	3230 (1217 to 4639)	34 970 (25 375 to 45 095)	..
	Botswana	0·57 (0·51 to 0·68)	109 (89 to 134)	3·92 (3·27 to 4·69)	6989 (5418 to 8325)	3777 (2935 to 4698)	0·09 (0·07 to 0·1)	144 (109 to 174)	1330 (929 to 1738)	3·30% (2·3 to 4·31)
	Lesotho	0·42 (0·38 to 0·47)	28 (22 to 34)	2·42 (2·0 to 2·92)	14 410 (13 144 to 15 473)	974 (787 to 1203)	0·06 (0·05 to 0·07)	287 (255 to 315)	686 (547 to 835)	10·02% (7·99 to 12·19)
	Namibia	0·55 (0·51 to 0·62)	115 (93 to 139)	3·57 (3·01 to 4·26)	8217 (6723 to 9470)	2997 (2395 to 3697)	0·1 (0·09 to 0·13)	120 (79 to 155)	1318 (917 to 1724)	4·54% (3·16 to 5·93)
	South Africa	0·53 (0·51 to 0·55)	118 (98 to 140)	3·53 (2·99 to 4·17)	212 016 (181 378 to 239 044)	2060 (1513 to 2739)	0·15 (0·12 to 0·2)	1078 (−660 to 2294)	27 705 (19 342 to 36 532)	3·89% (2·72 to 5·13)
	Swaziland (eSwatini)	0·5 (0·44 to 0·57)	80 (65 to 100)	2·93 (2·43 to 3·53)	6271 (5439 to 6994)	5141 (4059 to 6549)	0·04 (0·03 to 0·04)	171 (159 to 181)	1397 (1098 to 1744)	11·47% (9·01 to 14·32)
	Zimbabwe	0·46 (0·42 to 0·49)	21 (17 to 25)	2·73 (2·28 to 3·31)	67 921 (59 244 to 75 421)	769 (611 to 951)	0·06 (0·05 to 0·08)	1431 (1214 to 1613)	2533 (1922 to 3110)	7·48% (5·67 to 9·18)
Western sub-Saharan Africa		..	..	3·49 (2·96 to 4·14)	1 314 010 (1 052 581 to 1 531 034)	..	0·06 (0·05 to 0·07)	37 925 (33 376 to 41 367)	47 119 (36 150 to 57 550)	..
	Benin	0·46 (0·44 to 0·48)	7 (5 to 8)	3·71 (3·13 to 4·39)	32 265 (24 674 to 38 953)	400 (320 to 481)	0·05 (0·04 to 0·06)	1073 (949 to 1179)	645 (487 to 801)	2·60% (1·96 to 3·23)
	Burkina Faso	0·46 (0·44 to 0·48)	14 (12 to 17)	1·6 (1·32 to 1·89)	107 078 (99 758 to 113 840)	402 (325 to 499)	0·05 (0·04 to 0·06)	2156 (1938 to 2353)	2390 (1956 to 2856)	7·11% (5·82 to 8·5)
	Cameroon	0·45 (0·42 to 0·49)	11 (9 to 14)	4·07 (3·32 to 5·14)	67 795 (42 948 to 85 051)	608 (489 to 735)	0·06 (0·05 to 0·07)	2145 (1835 to 2406)	2102 (1505 to 2698)	2·67% (1·91 to 3·43)
	Cape Verde	0·62 (0·59 to 0·65)	24 (19 to 28)	6·16 (5·2 to 7·44)	465 (23 to 813)	1360 (1090 to 1638)	0·09 (0·07 to 0·1)	32 (23 to 38)	55 (33 to 76)	1·46 (0·87 to 2·0)
	Chad	0·37 (0·35 to 0·39)	8 (7 to 10)	2·96 (2·47 to 3·79)	58 393 (44 688 to 67 045)	525 (412 to 659)	0·04 (0·03 to 0·05)	1769 (1601 to 1900)	1440 (1084 to 1795)	4·55% (3·42 to 5·67)
	Côte d'Ivoire	0·43 (0·41 to 0·45)	14 (11 to 17)	3·92 (3·26 to 4·72)	65 217 (46 680 to 80 859)	699 (571 to 862)	0·06 (0·05 to 0·08)	1959 (1663 to 2225)	2303 (1663 to 2966)	2·57% (1·85 to 3·31)
	The Gambia	0·51 (0·48 to 0·53)	7 (6 to 9)	5·31 (4·39 to 6·33)	2790 (1133 to 4250)	551 (438 to 669)	0·06 (0·05 to 0·07)	163 (135 to 184)	112 (77 to 149)	3·18% (2·19 to 4·23)
	Ghana	0·52 (0·5 to 0·55)	18 (16 to 20)	4·79 (4·38 to 5·18)	53 317 (44 430 to 62 866)	1635 (1311 to 1954)	0·04 (0·04 to 0·05)	2674 (2465 to 2855)	5331 (4169 to 6500)	4·37% (3·42 to 5·33)
	Guinea	0·4 (0·37 to 0·42)	5 (4 to 6)	3·45 (2·88 to 4·14)	43 783 (34 234 to 52 028)	282 (226 to 344)	0·05 (0·04 to 0·06)	1319 (1174 to 1442)	586 (447 to 723)	2·76% (2·11 to 3·41)
	Guinea-Bissau	0·38 (0·36 to 0·41)	8 (7 to 10)	3·65 (3·05 to 4·35)	5952 (4596 to 7065)	460 (366 to 571)	0·06 (0·05 to 0·07)	176 (153 to 196)	132 (99 to 167)	4·30% (3·23 to 5·43)
	Liberia	0·46 (0·44 to 0·48)	13 (10 to 16)	3·58 (2·99 to 4·36)	15 502 (11 817 to 18 396)	736 (591 to 907)	0·06 (0·05 to 0·07)	446 (388 to 493)	536 (400 to 675)	13·49% (10·07 to 16·99)
	Mali	0·44 (0·41 to 0·48)	12 (10 to 14)	2·08 (1·84 to 2·33)	96 252 (89 929 to 102 622)	680 (593 to 771)	0·03 (0·03 to 0·03)	2571 (2465 to 2671)	2889 (2525 to 3253)	8·06% (7·05 to 9·08)
	Mauritania	0·5 (0·47 to 0·55)	13 (10 to 16)	5·25 (4·36 to 6·39)	6893 (3621 to 9457)	645 (514 to 791)	0·08 (0·07 to 0·1)	231 (172 to 279)	243 (165 to 325)	1·48% (1·0 to 1·98)
	Niger	0·43 (0·4 to 0·45)	6 (5 to 7)	2·41 (1·97 to 3·0)	97 816 (83 636 to 109 861)	268 (211 to 327)	0·04 (0·04 to 0·05)	2520 (2253 to 2718)	1237 (957 to 1516)	5·94% (4·59 to 7·28)
	Nigeria	0·48 (0·45 to 0·52)	19 (15 to 24)	3·54 (2·93 to 4·3)	595 624 (450 474 to 717 983)	830 (650 to 1023)	0·06 (0·05 to 0·08)	15 940 (13 191 to 18 072)	24 775 (17 858 to 31 958)	2·24% (1·61 to 2·89)
	São Tomé and Príncipe	0·55 (0·52 to 0·58)	17 (13 to 21)	4·94 (4·12 to 6·03)	315 (156 to 439)	793 (631 to 977)	0·09 (0·07 to 0·11)	11 (7 to 13)	14 (9 to 19)	2·09% (1·27 to 2·9)
	Senegal	0·45 (0·43 to 0·47)	9 (8 to 11)	4·16 (3·51 to 4·94)	36 370 (25 259 to 45 456)	664 (531 to 811)	0·05 (0·04 to 0·06)	1383 (1233 to 1510)	1272 (973 to 1604)	3·13% (2·39 to 3·94)
	Sierra Leone	0·44 (0·41 to 0·47)	8 (6 to 9)	6·14 (5·1 to 7·57)	7602 (2229 to 11 947)	815 (639 to 1009)	0·05 (0·04 to 0·06)	722 (648 to 786)	652 (471 to 842)	6·51% (4·71 to 8·41)
	Togo	0·45 (0·43 to 0·48)	7 (6 to 8)	4·08 (3·43 to 4·93)	20 583 (14 857 to 25 208)	400 (325 to 493)	0·06 (0·05 to 0·07)	637 (539 to 714)	405 (295 to 518)	3·47% (2·53 to 4·43)

Data in parentheses are 95% uncertainty intervals. Global data are broken down into World Bank income groups. Costs are given in in 2017 international dollars (I$). Table displays four sets of national estimates organised by GBD region: (1) cost per outpatient visit and inpatient admission by country; (2) aggregate ratio of total outpatient visits and inpatient admissions to counterfactual DALYs, where the counterfactual DALYs standardised the burden of disease across countries by removing the effects of access and quality of health care; (3) estimates of additional services needed to achieve the UHC standard for utilisation calculated by age and sex category; and (4) total cost of additional services in 2017 international dollars and as a percentage of 2016 GDP (cost estimates are presented in US$ in the appendix, pp 78–84). The cost of scaling up to the UHC utilisation standard was done at the age-sex level, leading some countries to have a cost of UHC scale-up greater than zero despite their aggregate utilisation per counterfactual DALY being greater than the standard set by the Netherlands. DALY=disability-adjusted-life-year. GBD=Global Burden of Diseases, Injuries, and Risk Factors Study. GDP=gross domestic product. UHC=universal health coverage.

**Figure 4:**
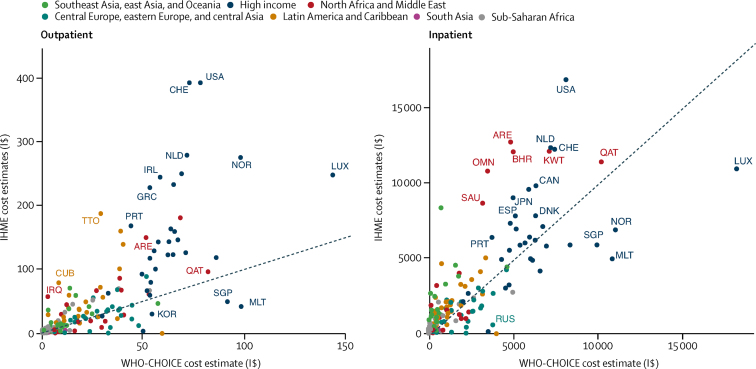
Comparison of 2008 IHME unit cost estimates to 2008 WHO-CHOICE estimates for outpatients and inpatients The figure shows scatter plots of the unit costs by country, where the horizontal axis reports the WHO-CHOICE estimates and the vertical axis reports our estimates. The diagonal line represents where the points would lie if the two estimates were identical. For outpatient visits, most points were higher and to the left of the diagonal line, showing that our estimates were higher. All unit costs are in 2010 international dollars (I$). ARE=United Arab Emirates. BHR=Bahrain. CAN=Canada. CHE=Switzerland. CUB=Cuba. DNK=Denmark. ESP=Spain. GRC=Greece. IRL=Ireland. IRQ=Iraq. JPN=Japan. KOR=Korea. KWT=Kuwait. LUX=Luxembourg. MLT=Malta. NLD=Netherlands. NOR=Norway. OMN=Oman. PRT=Portugal. QAT=Qatar. RUS=Russia. SAU=Saudi Arabia. SGP=Singapore. TTO=Trinidad and Tobago. USA=United States. IHME=Institute for Health Metrics and Evaluation. WHO-CHOICE=WHO Choosing Interventions that are Cost-Effective.

We compared our unit cost estimates to the WHO-CHOICE estimates in 2008, the year of their most recent estimates. Our estimates were generally higher (figure 4); cost per outpatient visit at any health facility was 102·67% higher on average than the WHO-CHOICE estimates for secondary hospitals, and cost per admission to any hospital was 2·64% higher on average than WHO-CHOICE estimates for teaching hospitals (appendix p 53).

Globally, 10·42 billion (95% UI 7·81–12·74) additional outpatient visits per year in 161 countries at a cost of (in 2017 I$) I$361·84 billion (212·28–526·66) were needed in 2016 to meet the UHC standard for utilisation, and 0·35 billion (0·31–0·38) additional inpatient admissions were needed in 184 countries at a cost of I$815·85 billion (583·87–1056·03), leading to a total additional cost of I$1177·69 billion (896·05–1456·56). The global gap in inpatient services was larger than in outpatient services, with a 49·20% increase required in admissions and a 26·57% increase required in visits to meet the UHC standard. This additional cost of each service for specific countries can be calculated with the results for unit cost and additional services in the table; note that the cost of scaling up to the UHC utilisation standard was done at the age-sex level, leading some countries to have a cost of UHC scale-up greater than zero despite their aggregate utilisation per counterfactual DALY being greater than the standard set by the Netherlands. Of the total additional cost required to reach the UHC standard, low-income countries required 4·00% (I$47·09 billion, 95% UI 37·38–56·08), lower-middle countries required 38·72% (I$456·04 billion, 365·53–550·89), upper-middle income countries required 34·67% (I$408·36 billion, 314·28–500·04), and high-income countries required 22·60% (I$266·21 billion, 152·21–380·71).

Four of 21 regions each accounted for 10% or more of the additional cost of reaching the UHC standard for utilisation: southeast Asia accounted for 25·48% (I$300·05 billion, 95% UI 233·61–370·17), high-income North America for 14·17% (I$166·93 billion, 92·60–241·26), south Asia for 12·34% (I$145·30 billion, 113·64–177·82), and north Africa and the Middle East for 10·13% (I$119·28 billion, 79·04–157·73; table). Much of the additional cost in southeast Asia was due to low inpatient utilisation per counterfactual DALY and high inpatient costs in Indonesia and the Philippines, as well as their large populations. In south Asia it was due to the low utilisation and large population in India, whereas in high-income North America it was due to high unit costs and large population in the USA. For north Africa and the Middle East, much of the additional cost was driven by Iran's low inpatient utilisation, high inpatient costs, and large population. The share of high-income North America increased to 28·97% (US$166·72 billion [95% UI 92·51–240·97] of US$575·57 billion [413·34–739·59]) in the US dollar estimates (appendix pp 78–84).

In sensitivity analyses using Portugal as an intermediate UHC standard for utilisation, the additional cost to meet this standard was 63·31% (I$745·58 billion, 95% UI 556·42–932·09) of the full standard (appendix pp 86–92). Under the intermediate UHC standard, the gap in admissions was 32·97% (0·23 billion of 0·71 billion admissions) and the gap in visits was 19·51% (7·67 billion of 39·35 billion visits; appendix pp 86–92). When adjusting for quality and types of service, the cost to reach the full standard increased by 25·23% to I$1474·81 billion (95% UI 1120·78–1824·45), with higher unit costs to reflect the cost of these improvements in the Netherlands (appendix pp 93–99).

## Discussion

We have reported the first global estimates of utilisation of outpatient visits and inpatient admissions, and unit costs for these services where the cost estimates were based on expenditures from the National Health Accounts. In our decomposition analysis, we have highlighted examples of countries with substantial changes in utilisation rates and have shown results for countries where increased volume was driven by population growth. Using the population and age structure in 2016, we have estimated the additional services and funds needed to meet a UHC standard for utilisation.

The decomposition analysis captures the effects of known trends in UHC, as well as other changes in health systems since 1990. China's increase in visits and admissions due to changes in utilisation rates was consistent with the expansion of insurance coverage to hospital services in 2003 and comprehensive care in 2008.^19^ Similarly, the increase in admissions in Indonesia due to changes in utilisation rates was consistent with a social security law in 2004 that included national health coverage.^20^ Although Indonesia's comprehensive health insurance scheme was not finalised until 2014, coverage of inpatient services expanded for some populations beginning in 2003. The increase in visits in Thailand due to changes in the utilisation rates was consistent with their UHC scheme that extended coverage in 2002 to the 30% of population who previously were uninsured.^21^ The increase in services in Turkey reflected the additional primary health-care teams and hospital beds from their Health Transformation Program.^22^ Age-sex-specific inpatient utilisation rates decreased in 21 of 29 countries in the central Europe, eastern Europe, and central Asia super-region, reflecting the decline in hospital beds in central Europe and eastern Europe from 1990 to 2005.^23^

The unit cost results for 188 countries were higher than the upper range of the widely referenced WHO-CHOICE estimates in 2008.^7^ The WHO-CHOICE researchers estimated cost functions with available unit cost estimates from 30 countries, where the unit of analysis was a facility-year.^7^ Our expenditure estimates included ancillary services such as diagnostic exams and medical supplies such as drugs provided during the visit or admission, consistent with the National Health Account categories,^10^ whereas the WHO-CHOICE estimates excluded them. Our unit costs estimates used utilisation as the denominator and reflected current efficiency. The WHO-CHOICE researchers sought to compare interventions across WHO locations and countries at a standard level of efficiency where all facilities operated at the 80th percentile of measured capacity. In the absence of estimates of actual unit costs, however, many researchers have relied on the WHO-CHOICE estimates as if they represented actual health systems, and thus have underestimated the cost of treatment interventions and the cost savings from preventive interventions in most countries.^8^, ^9^ Health facilities in Kenya, Uganda, and Zambia operated at 40% of capacity or less during 5-year periods that differed across countries, but were all between 2006 and 2011.^24^

To our knowledge, only two other studies have estimated comprehensive unit costs at the national level. Dieleman and colleagues^25^ reconciled the data from multiple sources with the United States' National Health Expenditure Account. Their 2013 cost per visit in the USA was 2017 US$557 compared with our estimate of US$457 (95% UI 397–525) for 2013 and cost per admission was US$18 626 compared with our estimate of US$21 000 (19 303–22 721). The Australian Independent Pricing Authority reported^26^ a 2016 cost per overnight admission in Australia of 2017 US$7429 compared with our estimate of US$8050 (7310–8865).

Our unit cost estimates were macro-costing estimates, which the second US Panel on Cost-Effectiveness and Medicine referred to as “gross costing”.^27^ Approaches to estimating unit costs ranged from our unit costs per visit and admission to micro-costing estimates that directly enumerate and cost every input, and neither approach is always more accurate or precise. The Panel recommended the macro-costing approach for some analyses because of its “simplicity, practicality, and if data are obtained broadly, robustness to geographic, institutional, and other sources of variation”.^30^ For example, a macro-costing estimate might be appropriate for an intervention that changed the quantity of services^8^, ^9^ or when its effect on the cost of services was known. Our macro-costing estimates were average costs, which would be the same as the marginal costs in stable health systems. Average costs can be less than marginal costs when initiating interventions or serving remote locations or populations. Researchers should consider the nature of the interventions, locations, and populations in their analyses and adjust the average costs as appropriate. Macro-costing estimates can be adjusted for specific diagnoses, using a country's weights for service intensity or other representative weights.^28^

The total cost of meeting a UHC standard for utilisation was 2017 I$1177·69 billion or 2017 US$575·57 billion and similar to previous UHC cost estimates for the same countries,^29^, ^30^ but our methods differed. Stenberg and colleagues^29^ estimated that progress towards UHC in 67 low-income and middle-income countries would cost 2017 US$287 billion per year by 2030 and I$391 billion for their ambitious scenario with 95% coverage of a full package of services. Our total cost for the same 67 countries was 2017 US$297·39 billion in 2016. Stenberg and colleagues used benchmarks such as the numbers of facilities and laboratories per person and human resource targets to estimate the cost of platforms, rather than the WHO-CHOICE unit costs, and added the commodity costs for 187 interventions. Jamison and colleagues^30^ estimated that a high priority package of interventions in 83 low-income and lower-middle income countries would cost 2017 US$113 billion in 2015 and US$223 billion for essential UHC. Our total cost for the same 83 countries was 2017 US$158·10 billion in 2016. Jamison and colleagues produced unit cost estimates for 218 interventions, using the best unit cost estimates in the literature with adjustments for health professional salaries across countries. Both previous estimates included the cost of population and community platforms, which they found to be 15%^29^ and 13–19%^30^ of total cost. Our estimates of the additional cost of personal health services did not include these platforms.

We report the first estimate of the additional services needed to meet a UHC standard. Equally important, our metric of utilisation per counterfactual DALY made it possible to compare health systems with different combinations of visits and admissions. Unlike diseases where zero burden is the goal, we needed to identify a goal for health services. There is both overutilisation and underutilisation of services. Some care can be provided as an outpatient or as an inpatient, with the latter being more expensive. For example, the burden could be reduced with several visits to screen for a disease and provide timely treatment, or it could be reduced with an admission for severe illness. Our frontier analysis explored different combinations of visits and admissions at the level of the health system rather than the intervention. We identified countries such as the Netherlands and Portugal whose combination achieved high values on GBD's UHC index at lower costs than for other combinations. In our sensitivity analysis using Portugal as the standard, it provided an intermediate UHC standard requiring relatively fewer admissions.

Our UHC cost estimate was based on additional services at the current quality and type of service, and was 25·23% higher in the sensitivity analysis with higher unit costs to reflect improvements, similar to the additional cost of commodities in previous estimates.^29^ Like previous estimates, ours was the starting point for national assessments that would benefit from country-specific information; quality improvement would be substantially more in some countries and minimal in others. When improvements in the quality of essential personal health services are delivered during visits and admissions, the additional cost of diagnostic exams and medical goods would be calculated using data on the country's burden of disease, current purchases, and lowest available prices, and then added to total expenditure for a service to calculate a country-specific estimate of higher unit costs.

To put our estimates in perspective, we used Dieleman and colleagues' estimate of pooled resources for health,^17^ which were prepaid revenues through government financing, social health insurance, private insurance, or development assistance for health. Pooled resources were THE minus out-of-pocket spending. In 2017 international dollars, the additional cost of reaching the UHC standard for utilisation in 2016 was 105·97% (I$71 of I$67 per capita) of pooled resources for low-income countries, 129·66% (I$153 of I$118 per capita) for lower-middle-income countries, 23·59% (I$159 of I$674 per capita) for upper-middle-income countries, and 4·66% (I$227 of I$4876 per capita) for high-income countries. As Dieleman and colleagues reported, some expansion of coverage in low-income and lower-middle-income countries might be possible with improvements in efficiency as well as additional funds.

Going forwards, health systems need to expand to accommodate population growth and ageing at the same time that they expand coverage. Population growth accounted for most of the increase in the volume of services from 1990 to 2016 globally and among four super-regions where the GBD's UHC index in many countries was low: Latin America and the Caribbean, north Africa and the Middle East, south Asia, and sub-Saharan Africa. Our cost of meeting a UHC standard for utilisation was based on population in 2016 but future estimates could include the additional cost associated with population growth and ageing.

Our methods for estimating utilisation and unit costs lend themselves to calculating the costs of future changes in health policy such as expansion in coverage. Utilisation could be forecast with global projections of population growth and age structure^31^ and forecasts of the Socio-demographic Index,^32^ which are available, as well as hospital capacity. Our analysis from 1990 to 2016 showed that hospital capacity was relatively stable over time in the absence of changes in health policy and could be forecast. Health expenditures on services could be forecast with available forecasts of THE and gross domestic product per capita^17^ and the share of expenditures on each service. Again, the shares were relatively stable and could be forecast. Estimates of the costs of changes in health policy would be modelled in this context.

This study had several limitations. A major limitation was the availability, quality, and scope of the data. Our systematic search for utilisation data revealed gaps, particularly before the year 2000 in countries outside of the high-income and central Europe, eastern Europe, and central Asia super-regions. Furthermore, the data sources for the other super-regions were primarily surveys and utilisation questionnaires were not standardised. Despite these gaps, 330 of 1175 country-years of outpatient data and 275 of 2068 country-years of inpatient data were from countries in the other super-regions, and our estimates adjusted for inconsistencies across questionnaires. In countries that continue to rely on surveys, it is important to standardise utilisation questions, as well as collect additional data (appendix p 10). A further limitation is that some of the decrease in inpatient admissions in the high-income super-region might have been associated with an increase in day hospital admissions, but these services were not included in our analysis. Utilisation and expenditure data for these services were not generally available, even though day curative (HC 1.2) and rehabilitative care (HC 2.2) were categories of the System of Health Accounts. Finally, although the utilisation estimates included facility-based preventive maternal and child care (HC 6.4), and vaccinations (HC 6.2), the expenditure shares did not because they were not reported in 649 (81·6%) of 795 of National Health Accounts. Their omission might have underestimated the unit cost of outpatient visits but the effects would have been substantial for only 16 country-years in which these categories exceeded 3% of THE.

In conclusion, plans to expand health coverage can be based on utilisation and unit costs of current health systems and guided by standards of utilisation of outpatient visits and inpatient admissions that achieve the highest coverage of personal health services at the lowest cost. Estimates of the additional services needed should direct attention towards growth of health systems in the future, and complement available research on the interventions that should be covered and their cost.

For the **Sustainable Development Goals and targets** see https://sustainabledevelopment.un.org

For **OECD statistics** see http://stats.oecd.org

For more on the **Global Health Data Exchange** see http://ghdx.healthdata.org/

## Data sharing

## References

[1] Horton R (2017). Offline: WHO—a roadmap to renewal?. Lancet.

[2] Schmidt H, Gostin LO, Emanuel EJ (2015). Public health, universal health coverage, and Sustainable Development Goals: can they coexist?. Lancet.

[3] GBD 2016 SDG Collaborators (2017). Measuring progress and projecting attainment on the basis of past trends of the health-related Sustainable Development Goals in 188 countries: an analysis from the Global Burden of Disease Study 2016. Lancet.

[4] Hogan DR, Stevens GA, Hosseinpoor AR, Boerma T (2018). Monitoring universal health coverage within the Sustainable Development Goals: development and baseline data for an index of essential health services. Lancet Glob Health.

[5] Devaux M (2015). Income-related inequalities and inequities in health care services utilisation in 18 selected OECD countries. Eur J Health Econ.

[6] Saksena P, Xu K, Elovainio R, Perrot J (2012). Utilization and expenditure at public and private facilities in 39 low-income countries. Trop Med Int Health.

[7] Stenberg K, Lauer JA, Gkountouras G, Fitzpatrick C, Stanciole A (2018). Econometric estimation of WHO-CHOICE country-specific costs for inpatient and outpatient health service delivery. Cost Eff Resour Alloc.

[8] Floyd J, Wu L, Hay Burgess D, Izadnegahdar R, Mukanga D, Ghani AC (2015). Evaluating the impact of pulse oximetry on childhood pneumonia mortality in resource-poor settings. Nature.

[9] Gu D, He J, Coxson PG (2015). The cost-effectiveness of low-cost essential antihypertensive medicines for hypertension control in China: a modelling study. PLoS Med.

[10] OECD, WHO, Eurostat (2011). A system of health accounts.

[11] Stevens GA, Alkema L, Black RE (2017). Guidelines for Accurate and Transparent Health Estimates Reporting: the GATHER statement. Epidemiol Serv Saude.

[12] Flaxman AD, Vos T, Murray CJL, Kiyono P (2015). An integrative metaregression framework for descriptive epidemiology.

[13] GBD 2016 Disease and Injury Incidence and Prevalence Collaborators (2017). Global, regional, and national incidence, prevalence, and years lived with disability for 328 diseases and injuries for 195 countries, 1990–2016: a systematic analysis for the Global Burden of Disease Study 2016. Lancet.

[14] GBD 2016 Mortality Collaborators (2017). Global, regional, and national under-5 mortality, adult mortality, age-specific mortality, and life expectancy, 1970-2016: a systematic analysis for the Global Burden of Disease Study 2016. Lancet.

[15] Das Gupta P (1991). Decomposition of the difference between two rates and its consistency when more than two populations are involved. Math Popul Stud.

[16] Dieleman JL, Haakenstad A, Micah A (2018). Spending on health and HIV/AIDS: domestic health spending and development assistance in 188 countries, 1995–2015. Lancet.

[17] Dieleman JL, Sadat N, Chang AY (2018). Trends in future health financing and coverage: future health spending and universal health coverage in 188 countries, 2016–40. Lancet.

[18] GBD 2016 Healthcare Access and Quality Collaborators (2018). Measuring performance on the Healthcare Access and Quality Index for 195 countries and territories and selected subnational locations: a systematic analysis from the Global Burden of Disease Study 2016. Lancet.

[19] Blumenthal D, Hsiao W (2015). Lessons from the east—China's rapidly evolving health care system. N Engl J Med.

[20] Pisani E, Olivier Kok M, Nugroho K (2017). Indonesia's road to universal health coverage: a political journey. Health Policy Plan.

[21] Tangcharoensathien V, Witthayapipopsakul W, Panichkriangkrai W, Patcharanarumol W, Mills A (2018). Health systems development in Thailand: a solid platform for successful implementation of universal health coverage. Lancet.

[22] Atun R (2015). Transforming Turkey's health system—lessons for universal coverage. N Engl J Med.

[23] Healy J, McKee M (2002). Implementing hospital reform in central and eastern Europe. Health Policy.

[24] Di Giorgio L, Moses MW, Fullman N (2016). The potential to expand antiretroviral therapy by improving health facility efficiency: evidence from Kenya, Uganda, and Zambia. BMC Med.

[25] Dieleman JL, Baral R, Birger M (2016). US spending on personal health care and public health, 1996–2013. JAMA.

[26] IHPA (April 23, 2018). National hospital cost data collection, public hospitals cost report, round 20 (financial year 2015–16). https://www.ihpa.gov.au/publications/national-hospital-cost-data-collection-public-hospitals-cost-report-round-20-0.

[27] Neumann PJ, Ganiats TG, Russell LB, Sanders GD, Siegel JE (2016). Cost-effectiveness in health and medicine.

[28] Reed SD, Friedman JY, Gnanasakthy A, Schulman KA (2003). Comparison of hospital costing methods in an economic evaluation of a multinational clinical trial. Int J Technol Assess Health Care.

[29] Stenberg K, Hanssen O, Edejer TT-T (2017). Financing transformative health systems towards achievement of the health Sustainable Development Goals: a model for projected resource needs in 67 low-income and middle-income countries. Lancet Glob Health.

[30] Jamison DT, Alwan A, Mock CN (2017). Universal health coverage and intersectoral action for health: key messages from Disease Control Priorities, 3rd edition. Lancet.

[31] United Nations Population Division (Jan 15, 2018). World population prospects 2017. https://esa.un.org/unpd/wpp/Download/Standard/Population/.

[32] Foreman KJ, Marquez N, Dolgert A (2018). Forecasting life expectancy, years of life lost, and all-cause and cause-specific mortality for 250 causes of death: reference and alternative scenarios for 2016–40 for 195 countries and territories. Lancet.

