# Supplementary material for: Funding and services needed to achieve universal health coverage: applications of global, regional, and national estimates of utilisation of outpatient visits and inpatient admissions from 1990 to 2016, and unit costs from 1995 to 2016
Source: Lancet Public Health. 2018 Dec 12;4(1):e49–73. doi: 10.1016/S2468-2667(18)30213-5 (PMC6323358; doi:10.1016/S2468-2667(18)30213-5)
Supplement: Supplementary appendix [file mmc1.pdf]

THE LANCET
Public Health

Supplementary appendix

This appendix formed part of the original submission and has been peer reviewed. We post it as supplied by the authors.

Supplement to: Moses M, Pedroza P, Baral R, et al. Funding and services needed to achieve universal health coverage: applications of global, regional, and national estimates of utilisation of outpatient visits and inpatient admissions from 1990 to 2016, and unit costs from 1995 to 2016. *Lancet Pubic Health 2018*; published online Dec 11. http://dx.doi.org/10.1016/S2468-2667(18)30213-5.

Supplementary Appendix

Funding and services needed to achieve universal health coverage: Applications of global, regional, and national estimates of utilisation of outpatient visits and inpatient admissions from 1990 to 2016, and unit costs from 1995 to 2016

Appendix to Funding and services needed to achieve universal health coverage: Applications of global, regional, and national estimates of utilisation of outpatient visits and inpatient admissions from 1990 to 2016, and unit costs from 1995 to 2016

Contents

Section 1. Overview 4

1.1. Introduction 4

1.2. GATHER compliance 4

1.3. List of acronyms 8

Section 2. Utilisation data sources 9

2.1. Systematic Review 9

2.2. Data Extraction 10

Section 3. Utilisation data grouping and adjustments 19

3.1. Inconsistencies across data sources 19

3.2. Assignment of reference data sources 20

3.3. Data grouping 20

3.4 Data adjustments 21

3.4.1 Inpatient data adjustments for data varying by age and sex 22

3.4.2. Outpatient data adjustments for data varying by age and sex 25

Section 4. Estimating the utilisation models 28

4.1. Introduction 28

4.2. Quantifying and adjusting for differences across data sources in DisMod-MR 28

4.3. Additional covariates and their definitions 28

4.4. Age-sex splitting 29

4.5. Raking DisMod-MR 2.1 estimates 29

4.6. Understanding the impact of missing data on DisMod-MR 2.1 estimates 30

4.7. Internal validity of results 30

4.8. Age standardisation 30

Section 5. Utilisation results 33

5.1. Age and sex specific utilisation rates 33

5.2. Country-specific utilisation rates from 1990-2016 33

Section 6. Unit cost estimates 51

6.1. Definition of unit cost 51

6.2. Data sources 51
6.3. Estimates of expenditures shares 52
6.4. Comparison to WHO-CHOICE estimates 53
6.5. Comparison to other comprehensive, national studies of the unit cost of services 53
6.6. Country-specific parameters for the calculation of 2016 unit costs 54
Section 7. Cost of universal health coverage 61
7.1. Introduction 61
7.2. DALYs in the absence of health services 61
7.3. Identifying the UHC standard for utilisation 71
7.4. Main analysis 78
7.5 Sensitivity analysis – intermediate UHC standard for utilisation 85
7.6 Sensitivity analysis – improvements in quality and types of service 85
References 100
Table S5. Data sources used in the utilisation estimates and their attributes by country and years 102

Section 1. Overview

1.1. Introduction

This appendix provides methodological detail and supplemental figures and tables. Figure S1 presents the methods for the utilisation estimates, which can be summarised in three processes, moving from left to right: 1) data extraction, 2) data adjustments, and 3) estimating the utilisation models. Each of the processes is described in Sections 2-4 below. Section 5 reports additional results for the utilisation estimates. Section 6 reports the methods and detailed results for the unit cost estimates. Section 7 reports the methods for estimating the additional cost of universal health coverage (UHC) and supplemental results.

1.2. GATHER compliance

The utilisation estimates incorporated a large number and wide variety of data sources on outpatient visits and inpatient admissions from 1990-2016. We documented the data sources and steps involved in our analytical procedures in compliance with the Guidelines for Accurate and Transparent Health Estimates Reporting (GATHER) recommendations (Table S1). The GATHER recommendations are at: http://gather-statement.org/

Figure S1. Estimation flow chart including data inputs, data adjustments, database inputs, modeling processes, and results

DisMod-MR 2.1= DisMod-Metaregression Version 2.1.

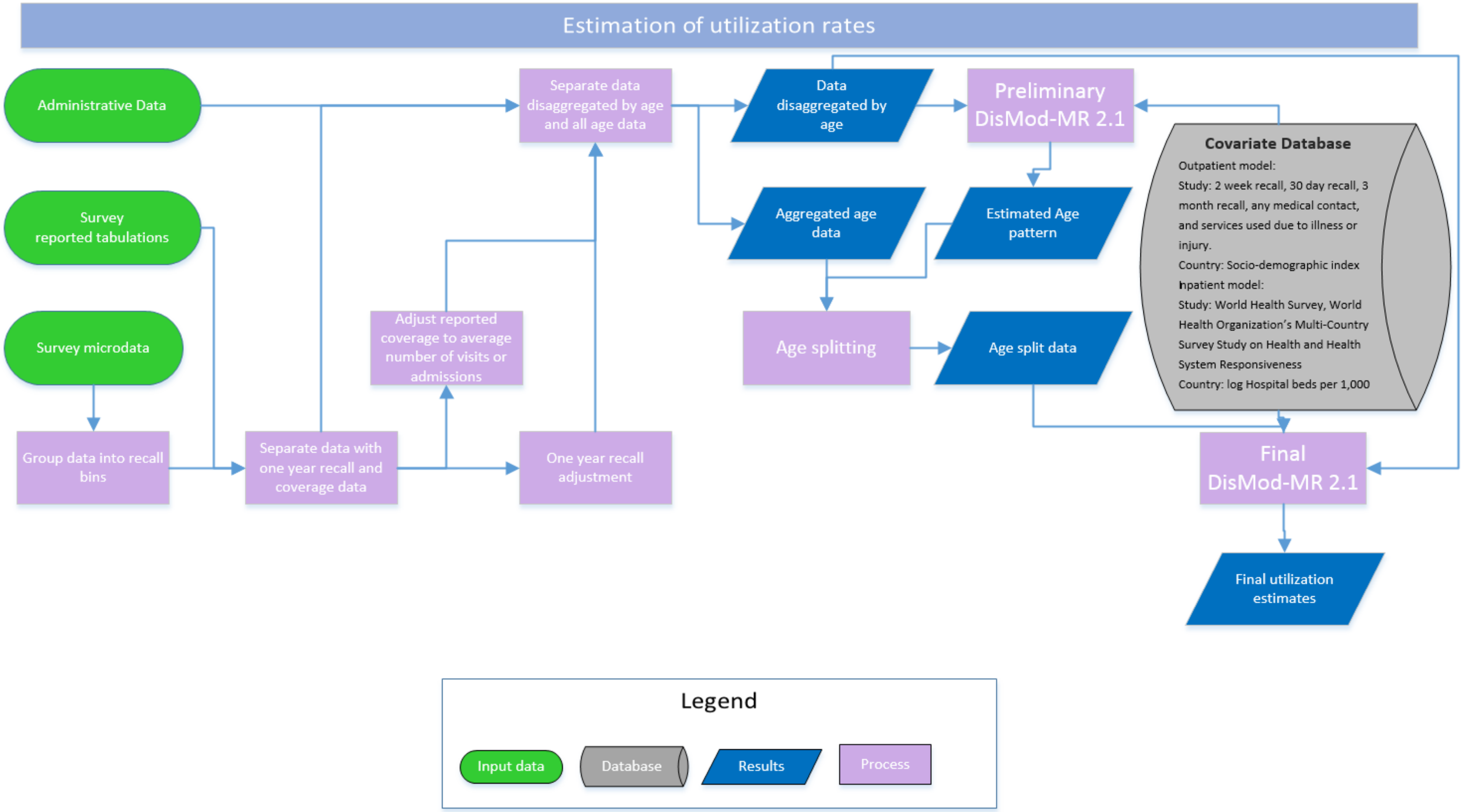

Table S1. GATHER checklist with description of compliance and location of information

#	GATHER checklist item	Description of compliance	Reference
**Objectives and funding**			
1	Define the indicators, populations, and time periods for which estimates were made.	Narrative provided in main text and supplementary appendix.	Main text (Background, Methods) and supplementary appendix (Sections 2, 4, 5; Appendix tables S3A, S3B, S5)
2	List the funding sources for the work.	Funding sources provided in main text.	Main text (Funding)
**Data Inputs**			
For all data inputs from multiple sources that are synthesised as part of the study:			
3	Describe how the data were identified and how the data were accessed.	Narrative description of data seeking methods provided in the main text and supplementary appendix.	Main text (Methods) and supplementary appendix (Section 2)
4	Specify the inclusion and exclusion criteria. Identify all ad-hoc exclusions.	Narrative about inclusion and exclusion criteria by data type provided in main text and supplementary appendix.	Main text (Methods) and supplementary appendix (Sections 2, 5)
5	Provide information on all included data sources and their main characteristics. For each data source used, report reference information or contact name/institution, population represented, data collection method, year(s) of data collection, sex and age range, diagnostic criteria or measurement method, and sample size	Attributes reported in supplementary appendix. Data sources available online through Global Health Data Exchange.	Supplementary appendix (Appendix tables S3A, S3B, S5) and online data tools (Global Health Data Exchange, http://ghdx.healthdata.org/node/380336)
6	Identify and describe any categories of input data that have potentially important biases (e.g., based on characteristics listed in item 5).	Discussion of bias provided in main text and supplementary appendix.	Main text (Methods) and supplementary appendix (Section 3)
For data inputs that contribute to the analysis but were not synthesised as part of the study:			
7	Describe and give sources for any other data inputs.	Included in supplementary appendix and online through Global Health Data Exchange	Supplementary appendix and Global Health Data Exchange, http://ghdx.healthdata.org/node/380336
For all data inputs:			
8	Provide all data inputs in a file format from which data can be efficiently extracted (e.g., a spreadsheet as opposed to a PDF), including all relevant metadata listed in item 5. For any data inputs that cannot be shared due to ethical or legal reasons, such as third-party ownership, provide a contact name or the name of the institution that retains the right to the data.	Download of input data are available through the Global Health Data Exchange	To download the data used in the analyses, please follow these links to the Global Health Data Exchange (GHDx):<br>- Utilisation of outpatient visits and inpatient admission, National Health Account expenditure shares: http://ghdx.healthdata.org/node/380336<br>- Total health expenditures: http://ghdx.healthdata.org/record/global-health-spending-1995-2015; http://ghdx.healthdata.org/record/global-expected-health-spending-2016-2040<br>- Disability-adjusted-life-years: http://ghdx.healthdata.org/record/global-burden-disease-

			study-2016-gbd-2016-disability-adjusted-life-years-and-healthy-life<br>- Population estimates: http://ghdx.healthdata.org/record/global-burden-disease-study-2016-gbd-2016-population-estimates-1950-2016
**Data analysis**			
9	Provide a conceptual overview of the data analysis method. A diagram may be helpful.	Flow diagrams of the overall methodological processes provided in the main text and supplementary appendix.	Main text (Methods) and supplementary appendix (Section 2)
10	Provide a detailed description of all steps of the analysis, including mathematical formulae. This description should cover, as relevant, data cleaning, data pre-processing, data adjustments and weighting of data sources, and mathematical or statistical model(s).	Flow diagrams and corresponding methodological write-ups provided in the main text and supplementary appendix.	Main text (Methods) and supplementary appendix (Sections 2-5)
11	Describe how candidate models were evaluated and how the final model(s) were selected.	Model evaluation provided in the main text and supplementary appendix.	Main text (Methods) and supplementary appendix (Sections 4-5)
12	Provide the results of an evaluation of model performance, if done, as well as the results of any relevant sensitivity analysis.	Performance evaluation and sensitivity analyses provided in the main text and supplementary appendix.	Main text (Methods) and supplementary appendix (Sections 4, 6, 7; Appendix tables S20, S21)
13	Describe methods for calculating uncertainty of the estimates. State which sources of uncertainty were, and were not, accounted for in the uncertainty analysis.	Description of uncertainty estimates provided in the main text and supplementary appendix.	Main text (Methods) and supplementary appendix (Sections 2-5)
14	State how analytic or statistical source code used to generate estimates can be accessed.	Code will be publicly available for access	All code used to generate estimates is available in the Global Health Data Exchange at http://ghdx.healthdata.org/node/380336
**Results and Discussion**			
15	Provide published estimates in a file format from which data can be efficiently extracted.	Download of results data available online through Global Health Data Exchange	All estimates are available in the Global Health Data Exchange at http://ghdx.healthdata.org/node/380336
16	Report a quantitative measure of the uncertainty of the estimates (e.g. uncertainty intervals).	Uncertainty intervals are provided with all results	All estimates with uncertainty intervals are available in the Global Health Data Exchange at http://ghdx.healthdata.org/node/380336
17	Interpret results in light of existing evidence. If updating a previous set of estimates, describe the reasons for changes in estimates.	Interpretation of results is provided in main text	Main text (Research in context)
18	Discuss limitations of the estimates. Include a discussion of any modelling assumptions or data limitations that affect interpretation of the estimates.	Discussion of limitations provided in the main text and supplementary appendix	Main text (Methods and Discussion) and supplementary appendix (Sections 4, 6, 7)

1.3. List of acronyms

Table S2. List of acronyms

Phrase	Acronym
Disability-adjusted-life-year	DALY
DisMod-Metaregression, version 2.1.	DisMod-MR 2.1
Global Burden of Disease	GBD
Global Health Data Exchange	GHDx
Gross Domestic Product	GDP
Guidelines for Accurate and Transparent Health Estimates Reporting	GATHER
Health Access and Quality	HAQ
Institute for Health Metrics and Evaluation	IHME
National Health Account	NHA
Primary sampling unit	PSU
Organisation for Economic Cooperation and Development	OECD
Socio-demographic Index	SDI
Standard error	SE
Spatio-Temporal Gaussian Process Regression	ST-GPR
Total Health Expenditure	THE
Universal Health Coverage	UHC
World Health Organization	WHO

Section 2. Utilisation data sources

2.1. Systematic Review

We searched the Global Health Data Exchange (GHDx) for population surveys, administrative records, and censuses from January 1990 to September 2016. We applied five search terms, referred to as secondary data filters in GHDx: "discharge", "health facility", "nationally representative", "household", or "outpatient." We also applied ten keyword filters: "healthcare access", "health care costs", "healthcare economics", "healthcare expenditure", "healthcare services", "healthcare use", "outpatient facilities", "patient counts", "hospitals", or "length of stay". We applied no language restrictions and required all returned records to have either administrative, survey microdata, or tabulated reports.

We searched the returned records' metadata for two measures of health service contact: 1) utilisation defined as the number of units of service used within a recall period, or 2) coverage defined as the proportion of respondents who used the service within a recall period. Figure S2

Figure S2. Process of systematically reviewing data sources for inclusion and exclusion

Counts reflect number of unique GHDx identification numbers. Multiple country-years may be assigned to one identification number, which explains discrepancies between counts in the figure below and counts reported elsewhere. The majority of the 696 records that were added in consultation with experts or further review of other records within a data series were sourced from administrative databases such as the World Health Organization's Health for All Database or the European Hospital Morbidity Database.

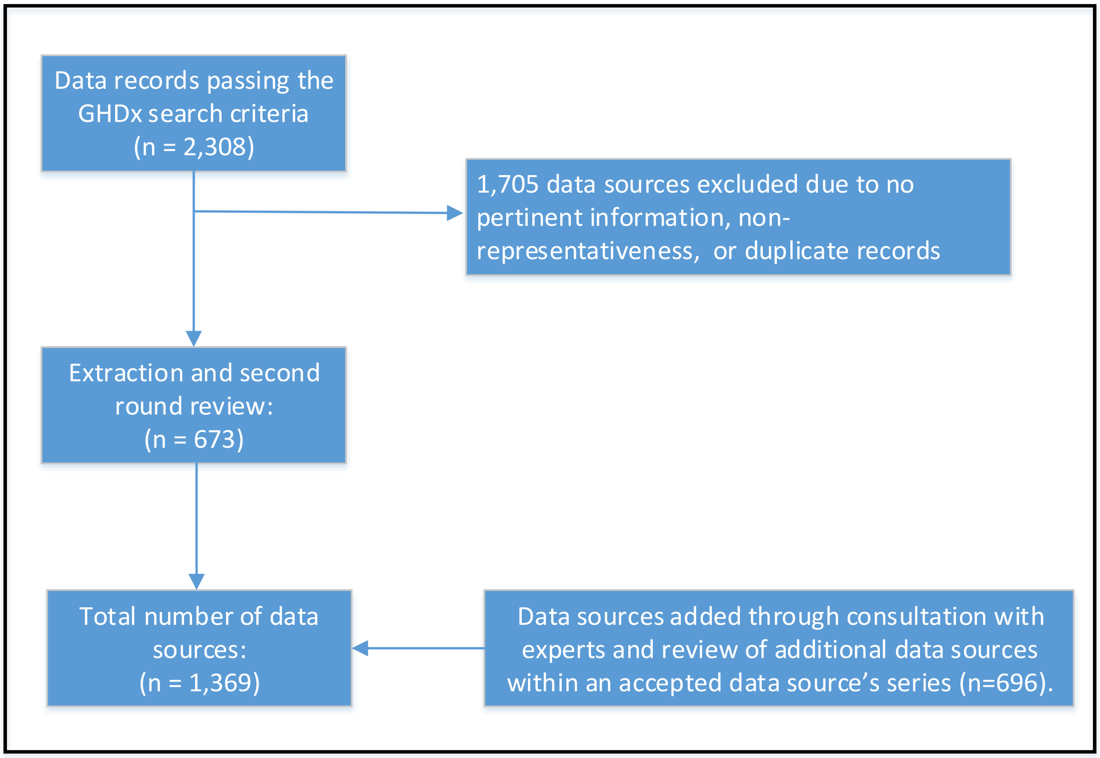

summarises the data sources that matched the search criteria, data sources that were excluded, and reasons for exclusion. We required that all measures be at the individual level and nationally or subnationally representative of outpatient visits, inpatient admissions, or general healthcare contact. If an accepted record was part of a series, we reviewed additional records in the series for inclusion, even if our initial search term did not include the record. Finally, we consulted with GBD collaborators and experts to identify relevant data sources that were either not contained within the GHDx or were excluded by our search terms.

We gathered outpatient utilisation data from 130 unique countries, spanning 1175 country-years, and inpatient data from 128 unique countries, spanning 2068 country-years. A summary of data sources by the GBD super-regions and time periods is in Table S3A, and by World Bank income categories and time periods is in Table S3B. Countries included in each GBD super-region and region are in Table S4. A list of all data sources by location and year is in Table S5, which is at the end of this document.

2.2. Data Extraction

The unit of analysis was average utilisation by sex and age categories, where the 23 age categories were: early neonatal (0-6 days), neonatal (7-27 days), infants (28-364 days), 1-4 years, and five-year intervals up to 90-94, and 95+ years. All administrative and survey microdata records were systematically extracted based on those categories, and we refer to an extracted estimate for a given age-sex-location-year-data source as a data point. For surveys with microdata, we extracted the individual level data and aggregated them in age and sex categories. For surveys without microdata, we extracted tabulated measures. When measures were reported in wide age intervals (e.g. all ages), we did age-splitting, as described in Section 4.4, Age-sex splitting. An exception was inpatient admissions for the early neonatal age category (0-6 days), for which we used the proportion of facility-based deliveries as an estimate of admissions per neonate.[1]

We used reported survey design variables such as sample weights, strata, and primary sampling units (PSUs) to calculate the mean of each data point and associated standard errors (SEs). When a specific surveys design variable was missing in greater than 10% of respondents, we dropped the survey design variable from the analysis. When neither the strata nor PSUs were available to estimate uncertainty, we used the sample size, ss, and mean, μ, of the data point to calculate SEs. When the product of the mean and sample size exceeded five we used equation (1) to calculate SEs:

$$SE = \frac{\sqrt{\mu \cdot ss}}{ss} \quad (1)$$

When the product of the mean and the sample size was less than or equal to five, we used equation (2) to calculate SEs.

$$SE = \frac{(5 - \mu \cdot ss)}{ss} + \frac{\mu \cdot ss\sqrt{\frac{5}{ss^2}}}{5} \quad (2)$$

When survey data included details on the type of provider, we excluded services provided by spiritual and religious healers. When microdata were not available we extracted reported tabulations.

We relied on the administrative and survey data sources to precisely define the units of service and to accurately count multiple units, such as visits to a general practitioner and specialist on the same day. This was important for outpatient visits, where 41.9% of the data sources were surveys, and a person could have more than one appointment at a facility each day. Some interviewer guides, such as the U.S. National Health Interview Survey's Manual, carefully defined doctor visits and gave instructions to consistently count visits when the patient sees: 1) two or more doctors, 2) a doctor and medical assistant under his/her supervision, and 3) two medical assistants supervised by different doctors.[2] We did not however, check the interviewer guide for every survey data source.

Table S3A. Descriptive statistics on administrative and survey data sources by GBD super-region and time period

Table displays counts of data sources by GBD super-region and time period, percentage of sources that were administrative data or survey data by GBD super-region, and number of countries with at least one data source or one administrative data source. GBD= Global Burden of Disease.

	Time period				Data Source as percentage of total		Number of countries with at least one data source	
	**Pre 1990**	**1990 - 2005**	**2005 - 2016**	**Total**	**Administrative data**	**Survey data**	**At least one data source (percentage)**	**At least one administrative data source (percentage)**
**Outpatient Total**	**6**	**526**	**643**	**1175**	**59.1%**	**40.9%**	**130 (66.7%)**	**51 (26.2 %)**
Central Europe, Eastern Europe, and Central Asia	0	176	246	422	85.1%	14.9%	27 (93.1%)	24 (82.8%)
High-income	4	168	249	421	60.6%	39.4%	30 (85.7%)	21 (61.8%)
Latin America and Caribbean	2	59	48	109	26.6%	73.4%	22 (68.8%)	2 (6.3%)
North Africa and Middle East	0	24	19	43	34.9%	65.1%	17 (81.0%)	1 (4.8%)
South Asia	0	9	3	12	0.0%	100.0%	4 (80.0 %)	0 (0.0 %)
Southeast Asia, East Asia, and Oceania	0	46	31	77	29.9%	70.1%	11 (39.3%)	2 (7.1%)
Sub-Saharan Africa	0	44	47	91	14.3%	85.7%	19 (41.3%)	1 (2.2%)
**Inpatient Total**	**369**	**992**	**707**	**2068**	**80.3%**	**19.7%**	**125 (64.1%)**	**60 (30.8%)**
Central Europe, Eastern Europe, and Central Asia	189	416	271	876	90.9%	9.1%	27 (93.1%)	25 (86.2%)
High-income	167	410	327	904	82.5%	17.5%	30 (85.7%)	27 (79.4%)
Latin America and Caribbean	2	58	41	101	42.6%	57.4%	18 (56.3%)	3 (9.4%)
North Africa and Middle East	10	30	13	53	66.0%	34.0%	13 (61.9%)	1 (4.8%)
South Asia	0	8	2	10	0.0%	100.0%	4 (80.0%)	0 (0.0%)
Southeast Asia, East Asia, and Oceania	1	46	26	73	35.6%	64.4%	12 (42.9%)	1 (3.6%)
Sub-Saharan Africa	0	24	27	51	29.4%	70.6%	21 (45.7%)	3 (6.5%)

Table S3B. Descriptive statistics on administrative and survey data sources by World Bank income categories and time period
Table displays counts of data sources by GBD super-region and time period, percentage of sources that were administrative data or survey data by World Bank income categories, and number of countries with at least one data source or one administrative data source.

	Time Period				Data Source as percentage of total		Number of countries with at least one data source	
	**Pre 1990**	**1990 - 2005**	**2005 - 2016**	**Total**	**Administrative data**	**Survey data**	**At least one data source (percentage)**	**At least one administrative data source (percentage)**
**Outpatient Total**	**6**	**526**	**643**	**1175**	**59.1%**	**40.9%**	**130 (66.7%)**	**51 (26.2%)**
Low income	0	19	17	36	0.0%	100.0%	14 (45.2%)	0 (0.0%)
Lower middle income	0	93	97	190	46.3%	53.7%	32 (61.5%)	5 (9.6%)
Upper middle income	2	167	185	354	59.6%	40.4%	36 (66.7%)	16 (29.6%)
High income	4	247	344	595	66.4%	33.6%	48 (82.8%)	30 (51.7%)
**Inpatient Total**	**369**	**992**	**707**	**2068**	**80.3%**	**19.7%**	**125 (64.1%)**	**60 (30.8%)**
Low income	0	11	12	23	21.7%	78.3%	14 (45.2%)	1 (3.2%)
Lower middle income	64	175	122	361	75.6%	24.4%	34 (65.4%)	9 (17.3%)
Upper middle income	76	258	167	501	77.6%	22.4%	34 (63.0%)	15 (27.8%)
High income	229	548	406	1183	84.0%	16.0%	43 (74.1%)	35 (60.3%)

Table S4. Locations characterised by GBD super-region and region

Location	ISO Code	Region	Super region
Afghanistan	AFG	North Africa and Middle East	North Africa and Middle East
Albania	ALB	Central Europe	Central Europe, Eastern Europe, and Central Asia
Algeria	DZA	North Africa and Middle East	North Africa and Middle East
American Samoa	ASM	Oceania	Southeast Asia, East Asia, and Oceania
Andorra	AND	Western Europe	High-income
Angola	AGO	Central sub-Saharan Africa	Sub-Saharan Africa
Antigua and Barbuda	ATG	Caribbean	Latin America and Caribbean
Argentina	ARG	Southern Latin America	High-income
Armenia	ARM	Central Asia	Central Europe, Eastern Europe, and Central Asia
Australia	AUS	Australasia	High-income
Austria	AUT	Western Europe	High-income
Azerbaijan	AZE	Central Asia	Central Europe, Eastern Europe, and Central Asia
Bahrain	BHR	North Africa and Middle East	North Africa and Middle East
Bangladesh	BGD	South Asia	South Asia
Barbados	BRB	Caribbean	Latin America and Caribbean
Belarus	BLR	Eastern Europe	Central Europe, Eastern Europe, and Central Asia
Belgium	BEL	Western Europe	High-income
Belize	BLZ	Caribbean	Latin America and Caribbean
Benin	BEN	Western sub-Saharan Africa	Sub-Saharan Africa
Bermuda	BMU	Caribbean	Latin America and Caribbean
Bhutan	BTN	South Asia	South Asia
Bolivia	BOL	Andean Latin America	Latin America and Caribbean
Bosnia and Herzegovina	BIH	Central Europe	Central Europe, Eastern Europe, and Central Asia
Botswana	BWA	Southern sub-Saharan Africa	Sub-Saharan Africa
Brazil	BRA	Tropical Latin America	Latin America and Caribbean
Brunei	BRN	High-income Asia Pacific	High-income
Bulgaria	BGR	Central Europe	Central Europe, Eastern Europe, and Central Asia
Burkina Faso	BFA	Western sub-Saharan Africa	Sub-Saharan Africa
Burundi	BDI	Eastern sub-Saharan Africa	Sub-Saharan Africa
Cambodia	KHM	Southeast Asia	Southeast Asia, East Asia, and Oceania
Cameroon	CMR	Western sub-Saharan Africa	Sub-Saharan Africa

Canada	CAN	High-income North America	High-income
Cape Verde	CPV	Western sub-Saharan Africa	Sub-Saharan Africa
Central African Republic	CAF	Central sub-Saharan Africa	Sub-Saharan Africa
Chad	TCD	Western sub-Saharan Africa	Sub-Saharan Africa
Chile	CHL	Southern Latin America	High-income
China	CHN	East Asia	Southeast Asia, East Asia, and Oceania
Colombia	COL	Central Latin America	Latin America and Caribbean
Comoros	COM	Eastern sub-Saharan Africa	Sub-Saharan Africa
Congo	COG	Central sub-Saharan Africa	Sub-Saharan Africa
Costa Rica	CRI	Central Latin America	Latin America and Caribbean
Cote d'Ivoire	CIV	Western sub-Saharan Africa	Sub-Saharan Africa
Croatia	HRV	Central Europe	Central Europe, Eastern Europe, and Central Asia
Cuba	CUB	Caribbean	Latin America and Caribbean
Cyprus	CYP	Western Europe	High-income
Czech Republic	CZE	Central Europe	Central Europe, Eastern Europe, and Central Asia
DR Congo	COD	Central sub-Saharan Africa	Sub-Saharan Africa
Denmark	DNK	Western Europe	High-income
Djibouti	DJI	Eastern sub-Saharan Africa	Sub-Saharan Africa
Dominica	DMA	Caribbean	Latin America and Caribbean
Dominican Republic	DOM	Caribbean	Latin America and Caribbean
Ecuador	ECU	Andean Latin America	Latin America and Caribbean
Egypt	EGY	North Africa and Middle East	North Africa and Middle East
El Salvador	SLV	Central Latin America	Latin America and Caribbean
England	ENG	Western Europe	High-income
Equatorial Guinea	GNQ	Central sub-Saharan Africa	Sub-Saharan Africa
Eritrea	ERI	Eastern sub-Saharan Africa	Sub-Saharan Africa
Estonia	EST	Eastern Europe	Central Europe, Eastern Europe, and Central Asia
Ethiopia	ETH	Eastern sub-Saharan Africa	Sub-Saharan Africa
Federated States of Micronesia	FSM	Oceania	Southeast Asia, East Asia, and Oceania
Fiji	FJI	Oceania	Southeast Asia, East Asia, and Oceania
Finland	FIN	Western Europe	High-income
France	FRA	Western Europe	High-income
Gabon	GAB	Central sub-Saharan Africa	Sub-Saharan Africa
Georgia	GEO	Central Asia	Central Europe, Eastern Europe, and Central Asia
Germany	DEU	Western Europe	High-income

Ghana	GHA	Western sub-Saharan Africa	Sub-Saharan Africa
Greece	GRC	Western Europe	High-income
Greenland	GRL	High-income North America	High-income
Grenada	GRD	Caribbean	Latin America and Caribbean
Guam	GUM	Oceania	Southeast Asia, East Asia, and Oceania
Guatemala	GTM	Central Latin America	Latin America and Caribbean
Guinea	GIN	Western sub-Saharan Africa	Sub-Saharan Africa
Guinea-Bissau	GNB	Western sub-Saharan Africa	Sub-Saharan Africa
Guyana	GUY	Caribbean	Latin America and Caribbean
Haiti	HTI	Caribbean	Latin America and Caribbean
Honduras	HND	Central Latin America	Latin America and Caribbean
Hungary	HUN	Central Europe	Central Europe, Eastern Europe, and Central Asia
Iceland	ISL	Western Europe	High-income
India	IND	South Asia	South Asia
Indonesia	IDN	Southeast Asia	Southeast Asia, East Asia, and Oceania
Iran	IRN	North Africa and Middle East	North Africa and Middle East
Iraq	IRQ	North Africa and Middle East	North Africa and Middle East
Ireland	IRL	Western Europe	High-income
Israel	ISR	Western Europe	High-income
Italy	ITA	Western Europe	High-income
Jamaica	JAM	Caribbean	Latin America and Caribbean
Japan	JPN	High-income Asia Pacific	High-income
Jordan	JOR	North Africa and Middle East	North Africa and Middle East
Kazakhstan	KAZ	Central Asia	Central Europe, Eastern Europe, and Central Asia
Kenya	KEN	Eastern sub-Saharan Africa	Sub-Saharan Africa
Kiribati	KIR	Oceania	Southeast Asia, East Asia, and Oceania
Kuwait	KWT	North Africa and Middle East	North Africa and Middle East
Kyrgyzstan	KGZ	Central Asia	Central Europe, Eastern Europe, and Central Asia
Laos	LAO	Southeast Asia	Southeast Asia, East Asia, and Oceania
Latvia	LVA	Eastern Europe	Central Europe, Eastern Europe, and Central Asia
Lebanon	LBN	North Africa and Middle East	North Africa and Middle East
Lesotho	LSO	Southern sub-Saharan Africa	Sub-Saharan Africa
Liberia	LBR	Western sub-Saharan Africa	Sub-Saharan Africa
Libya	LBY	North Africa and Middle East	North Africa and Middle East
Lithuania	LTU	Eastern Europe	Central Europe, Eastern Europe, and Central Asia
Luxembourg	LUX	Western Europe	High-income

Macedonia	MKD	Central Europe	Central Europe, Eastern Europe, and Central Asia
Madagascar	MDG	Eastern sub-Saharan Africa	Sub-Saharan Africa
Malawi	MWI	Eastern sub-Saharan Africa	Sub-Saharan Africa
Malaysia	MYS	Southeast Asia	Southeast Asia, East Asia, and Oceania
Maldives	MDV	Southeast Asia	Southeast Asia, East Asia, and Oceania
Mali	MLI	Western sub-Saharan Africa	Sub-Saharan Africa
Malta	MLT	Western Europe	High-income
Marshall Islands	MHL	Oceania	Southeast Asia, East Asia, and Oceania
Mauritania	MRT	Western sub-Saharan Africa	Sub-Saharan Africa
Mauritius	MUS	Southeast Asia	Southeast Asia, East Asia, and Oceania
Mexico	MEX	Central Latin America	Latin America and Caribbean
Moldova	MDA	Eastern Europe	Central Europe, Eastern Europe, and Central Asia
Mongolia	MNG	Central Asia	Central Europe, Eastern Europe, and Central Asia
Montenegro	MNE	Central Europe	Central Europe, Eastern Europe, and Central Asia
Morocco	MAR	North Africa and Middle East	North Africa and Middle East
Mozambique	MOZ	Eastern sub-Saharan Africa	Sub-Saharan Africa
Myanmar	MMR	Southeast Asia	Southeast Asia, East Asia, and Oceania
Namibia	NAM	Southern sub-Saharan Africa	Sub-Saharan Africa
Nepal	NPL	South Asia	South Asia
Netherlands	NLD	Western Europe	High-income
New Zealand	NZL	Australasia	High-income
Nicaragua	NIC	Central Latin America	Latin America and Caribbean
Niger	NER	Western sub-Saharan Africa	Sub-Saharan Africa
Nigeria	NGA	Western sub-Saharan Africa	Sub-Saharan Africa
North Korea	PRK	East Asia	Southeast Asia, East Asia, and Oceania
Northern Ireland	NIR	Western Europe	High-income
Northern Mariana Islands	MNP	Oceania	Southeast Asia, East Asia, and Oceania
Norway	NOR	Western Europe	High-income
Oman	OMN	North Africa and Middle East	North Africa and Middle East
Pakistan	PAK	South Asia	South Asia
Palestine	PSE	North Africa and Middle East	North Africa and Middle East
Panama	PAN	Central Latin America	Latin America and Caribbean
Papua New Guinea	PNG	Oceania	Southeast Asia, East Asia, and Oceania
Paraguay	PRY	Tropical Latin America	Latin America and Caribbean
Peru	PER	Andean Latin America	Latin America and Caribbean
Philippines	PHL	Southeast Asia	Southeast Asia, East Asia, and Oceania

Poland	POL	Central Europe	Central Europe, Eastern Europe, and Central Asia
Portugal	PRT	Western Europe	High-income
Puerto Rico	PRI	Caribbean	Latin America and Caribbean
Qatar	QAT	North Africa and Middle East	North Africa and Middle East
Romania	ROU	Central Europe	Central Europe, Eastern Europe, and Central Asia
Russia	RUS	Eastern Europe	Central Europe, Eastern Europe, and Central Asia
Rwanda	RWA	Eastern sub-Saharan Africa	Sub-Saharan Africa
Saint Lucia	LCA	Caribbean	Latin America and Caribbean
Saint Vincent and the Grenadines	VCT	Caribbean	Latin America and Caribbean
Samoa	WSM	Oceania	Southeast Asia, East Asia, and Oceania
Sao Tome and Principe	STP	Western sub-Saharan Africa	Sub-Saharan Africa
Saudi Arabia	SAU	North Africa and Middle East	North Africa and Middle East
Scotland	SCT	Western Europe	High-income
Senegal	SEN	Western sub-Saharan Africa	Sub-Saharan Africa
Serbia	SRB	Central Europe	Central Europe, Eastern Europe, and Central Asia
Seychelles	SYC	Southeast Asia	Southeast Asia, East Asia, and Oceania
Sierra Leone	SLE	Western sub-Saharan Africa	Sub-Saharan Africa
Singapore	SGP	High-income Asia Pacific	High-income
Slovakia	SVK	Central Europe	Central Europe, Eastern Europe, and Central Asia
Slovenia	SVN	Central Europe	Central Europe, Eastern Europe, and Central Asia
Solomon Islands	SLB	Oceania	Southeast Asia, East Asia, and Oceania
Somalia	SOM	Eastern sub-Saharan Africa	Sub-Saharan Africa
South Africa	ZAF	Southern sub-Saharan Africa	Sub-Saharan Africa
South Korea	KOR	High-income Asia Pacific	High-income
South Sudan	SSD	Eastern sub-Saharan Africa	Sub-Saharan Africa
Spain	ESP	Western Europe	High-income
Sri Lanka	LKA	Southeast Asia	Southeast Asia, East Asia, and Oceania
Sudan	SDN	North Africa and Middle East	North Africa and Middle East
Suriname	SUR	Caribbean	Latin America and Caribbean
Swaziland	SWZ	Southern sub-Saharan Africa	Sub-Saharan Africa
Sweden	SWE	Western Europe	High-income
Switzerland	CHE	Western Europe	High-income
Syria	SYR	North Africa and Middle East	North Africa and Middle East
Taiwan	TWN	East Asia	Southeast Asia, East Asia, and Oceania
Tajikistan	TJK	Central Asia	Central Europe, Eastern Europe, and Central Asia

Tanzania	TZA	Eastern sub-Saharan Africa	Sub-Saharan Africa
Thailand	THA	Southeast Asia	Southeast Asia, East Asia, and Oceania
The Bahamas	BHS	Caribbean	Latin America and Caribbean
The Gambia	GMB	Western sub-Saharan Africa	Sub-Saharan Africa
Timor-Leste	TLS	Southeast Asia	Southeast Asia, East Asia, and Oceania
Togo	TGO	Western sub-Saharan Africa	Sub-Saharan Africa
Tonga	TON	Oceania	Southeast Asia, East Asia, and Oceania
Trinidad and Tobago	TTO	Caribbean	Latin America and Caribbean
Tunisia	TUN	North Africa and Middle East	North Africa and Middle East
Turkey	TUR	North Africa and Middle East	North Africa and Middle East
Turkmenistan	TKM	Central Asia	Central Europe, Eastern Europe, and Central Asia
Uganda	UGA	Eastern sub-Saharan Africa	Sub-Saharan Africa
Ukraine	UKR	Eastern Europe	Central Europe, Eastern Europe, and Central Asia
United Arab Emirates	ARE	North Africa and Middle East	North Africa and Middle East
United States	USA	High-income North America	High-income
Uruguay	URY	Southern Latin America	High-income
Uzbekistan	UZB	Central Asia	Central Europe, Eastern Europe, and Central Asia
Vanuatu	VUT	Oceania	Southeast Asia, East Asia, and Oceania
Venezuela	VEN	Central Latin America	Latin America and Caribbean
Vietnam	VNM	Southeast Asia	Southeast Asia, East Asia, and Oceania
Virgin Islands, U.S.	VIR	Caribbean	Latin America and Caribbean
Wales	WLS	Western Europe	High-income
Yemen	YEM	North Africa and Middle East	North Africa and Middle East
Zambia	ZMB	Eastern sub-Saharan Africa	Sub-Saharan Africa
Zimbabwe	ZWE	Southern sub-Saharan Africa	Sub-Saharan Africa

Section 3. Utilisation data grouping and adjustments

3.1. Inconsistencies across data sources

Measures of utilisation and recall periods were not consistent across surveys (Table S6). As noted above, health service contacts were reported as either utilisation or coverage in the available data. Among survey data, utilisation was measured in 196 (40.7%) of surveys with outpatient data and 117 (28.7%) with inpatient data. In querying respondents, 186 (38.7%) surveys did not distinguish the type of service (outpatient or inpatient care) and were analysed with the outpatient data. Among outpatient surveys, 138 (28.7%) restricted utilisation questions to people who had been ill. Further, the recall period of questions varied across

Table S6. Descriptive statistics on survey by recall period, health services questions, and units of service

Table displays number (percentage) of surveys gathered by recall period, phrasing of the questions, and service measure (utilisation or coverage).

	Outpatient number	Outpatient percentage	Inpatient number	Inpatient percentage
**Total number of surveys**	**481**		**407**	
**Recall period**				
2 weeks	69	14.3%	10	2.5%
2.1 weeks (15 days)	3	0.6%	..	..
4 weeks	84	17.5%	29	7.1%
4.3 weeks	131	27.2%	84	20.6%
8.6 weeks (2 months)	8	1.7%	1	0.2%
12.9 weeks (3 months)	39	8.1%	29	7.1%
17 weeks (4 months)	4	0.8%	2	0.5%
26 weeks (6 months)	9	1.9%	3	0.7%
One Year	134	27.9%	249	61.2%
**Question specifies outpatient and inpatient services**				
Use of specific service	295	61.3%	407	100.0%
Use of any services, without distinguishing outpatient or inpatient	186	38.7%	..	..
**Question specifies recent illness**				
Use of services regardless of health status, i.e. may include preventive care	343	71.3%	407	100.0%
Specifies person in household with a recent illness or injury	138	28.7%	..	..
**Question on utilisation vs coverage**				
Utilisation, defined as a number of units of service used within a recall period	196	40.7%	117	28.7%
Coverage, defined as a proportion of people who used service within recall period	285	59.3%	290	71.3%

surveys, such as two weeks, 15 days, one month, 30 days, or two months. Longer recall periods have been reported to be biased.[3–5]

We sought to produce valid and consistent estimates while making use of all available information. To accomplish this, we reconciled inconsistent measures in three steps: 1) assigned a reference data sources that most closely matched our case definition, 2) grouped reported measures into consistent recall bins, and 3) quantified and adjusted for differences across data sources.

3.2. Assignment of reference data sources

Our case definition was nationally representative, annual utilisation rate per capita. See the Definition of utilisation section of the main text for the corresponding functional categories of the National Health Accounts (NHA). The data sources that most closely matched this definition were annually reported, administrative records from either national sources or facility-level health information system data, and free of recall bias. Examples are the World Health Organization's European Hospital Morbidity Database, and Health for All database, the Organisation for Economic Cooperation and Development database, United States' Healthcare Cost and Utilisation Project data, and the Institute for Health Metrics and Evaluation's (IHME's) Access, Bottlenecks, Costs, and Equity survey. When data were reported by more than one source, such as the European Hospital Morbidity Database and Health for All database, we used only the most detailed one. All of the administrative sources reported in Table S3 were reference data, and all reference source are noted in Table S5.

3.3. Data grouping

Survey recall periods varied substantially (Table S6). We sought to improve the uniformity of the data by grouping data from population-based surveys into bins based on the recall period. We grouped outpatient data into four recall bins of: two weeks, 30 days, three months, and one year. We grouped inpatient data into two recall bins of: 30 days, and one year. Other recall periods were grouped into a bins selected on the basis of breaks in the frequency of recall periods. Assignments of surveys into bins was based on proximity to the nearest bin (Table S7).

For surveys reporting utilisation rates in recall periods not exactly matching the assigned recall bin such as 15 days instead of two weeks, we multiplied the data point mean by a constant, k, where $k = \frac{t_{new}}{t_{old}}$ and t_{old} represents the survey reported recall period and t_{new} represents the recall period of the assigned bin. Coefficients of variation were used to calculate new standard errors for the adjusted data points.

Table S7. Grouping of survey recall periods

Table displays how survey recalls periods were grouped into bins to improve the consistency of the data.

Model	Survey recall bins	Survey recall period
**Outpatient**	2 weeks	2 weeks, 2.1 weeks (15 days)
	30 days (4.3 weeks)	4 weeks, 30 days, one month, 2 months (8.6 weeks)
	3 months (12.9 weeks)	3 months, 4 months (17.2 weeks), 6 months (26 weeks)
	One year (52 weeks)	One year
**Inpatient**	30 days	2 weeks, 4 weeks, 30 days, 1 month, 2 months, 3 months, 4 months, 6 months
	One year (52 weeks)	One year

For surveys reporting proportions who used services in recall periods that didn't exactly match the assigned recall bins, the adjustment assumed an exponential decline in recall. To adjust reported proportions and propagate uncertainty, we took draws from a normal distribution where the mean was the logit transformed reported proportion, p_{old}, and the SE was the SE of p_{old} converted to logit space by way of the delta method. These draws were then back transformed to a proportion. This procedure was taken to generate draws of p_{old} bounded between zero and one that matched the reported standard error. The draws of p_{old} were then converted to rates, r, by $r = \frac{-\ln(1-p_{old})}{t_{old}}$. Using the rates and the recall period of the assigned recall bin, t_{new}, we calculated a new proportion, p_{new}, by $p_{new} = 1 - e^{-r \cdot t_{new}}$. The mean of the draws was the point estimate, and the 95% uncertainty interval was calculated from the draws.

3.4 Data adjustments

We accounted for discrepancies between reference data sources and survey data types. A survey data type was defined by service (outpatient or inpatient), reported measure (utilisation or coverage), and assigned recall bin (2 weeks, 30-day, three months, one year). We used two methods to adjust for survey data types that differed from reference data types: dichotomous covariates, and age-spline adjustments. When survey data types differed systematically and multiplicatively from reference data sources, we adjusted for the difference with dichotomous covariates within the DisMod-MR 2.1 model. More details on this procedure may be found in Section 4.2. When survey data types didn't differ systematically and multiplicatively from reference data types, we used age-spline regressions to adjust for the differences as described in the succeeding Section 3.4.1. For example, the number of inpatient admissions reported with one-year recall showed over-reporting for the youngest and oldest age categories, and under-reporting for the middle age categories relative to the reference data. We documented the process of adjusting each survey data type to the reference data type (Table S8). All analyses and adjustments were done with Python, version 2.7. (Python Software Foundation. Available at http://www.python.org.)

We used visual inspection to determine whether or not the survey data differed systematically and multiplicatively from the reference data sources. We estimated the DisMod-MR regression model with covariates for each data adjustment (See Section 4.2), and we created a figure to compare the utilisation estimates by age with the data points. If the estimates for an adjustment weren't consistently above or below the data points for any age categories, we concluded that the inconsistencies did not differ by age category.

The age-spline adjustments addressed differences across data sources by age, but not location. As reported in Table S8, the reference data were used in the age-spline adjustments for inpatient utilisation with recall for 1-year, inpatient coverage with recall for 1-year and 1-month, and outpatient coverage with recall for 1-year. For inpatient utilisation with 1-year recall, the adjustment assumed that relationship of the respondents' recall and patient's age is the same across super-regions. For the coverage data, the adjustment added the assumption that the relationship between utilisation and coverage across age groups is the same across super-regions. For example, the adjustment assumed then when a child who used a service during the recall period, the number of units of service was the same across super-regions.

The metaregression analysis adjusted for recall and location, but not the interaction between them. The reference data were also used in the metaregression analysis to adjust utilisation rates reported in surveys compared to administrative sources. The adjustment assumed that the relationship between survey responses and administrative data is the same across super-regions.

Table S8. Survey data types and methods of adjustment

Table displays reported survey data type (recall period and reported measure), the method of adjustment, and the equivalent data type after adjustment. All reported measures were adjusted to the reference data in the final DisMod-MR estimates.

Model	Recall period	Reported measure	Method of adjustment	Equivalent data type after adjustment
**Outpatient**	Two weeks	Utilisation	Dichotomous covariate	Reference data source
	Two weeks	Proportion who used service	Age-spline adjustment	Two-week utilisation recall
	30 days	Utilisation	Dichotomous covariate	Reference data source
	30 days	Proportion who used service	Age-spline adjustment	30 day utilisation recall
	Three months	Utilisation	Dichotomous covariate	Reference data source
	Three months	Proportion who used service	Age-spline adjustment	Three-month utilisation recall
	One-year	Utilisation	Dichotomous covariate	Reference data source
	One-year	Proportion who used service	Age-spline adjustment	Reference data source
**Inpatient**	30 days	Proportion who used service	Age-spline adjustment	Reference data source
	One-year	Utilisation	Age-spline adjustment	Reference data source
	One-year	Proportion who used service	Age-spline adjustment	Reference data source

3.4.1 Inpatient data adjustments for data varying by age and sex

To adjust for inconsistent data that differed by age, such as the one-year recall biases for inpatient admissions, we estimated adjustment factors with penalised spline regressions. The penalised spline regression produced flexible and non-linear estimates across age categories. We calculated the ratio of the point estimate from the reference data, μ_{ref}, to the survey data, μ_s. We fit these ratios with a penalised spline regression shown in equation (3):

$$\ln\left(\frac{\mu_{ref,i}}{\mu_{s,i}}\right) = \beta_0 + h(age_i) + \varepsilon_i \quad (3)$$

Where i denotes a given matched observation, $h(age_i)$ represents a basis function which estimated a cross-validated penalised spline over the population weighted mean age of the age category, and ε represents the residual. To adjust survey data to reference data, we multiplied survey data by the exponentiated predictions from respective penalised spline regressions (adjustment factor).

We accounted for uncertainty from the adjustments for using equation (4):

$$se_a = \sqrt{se_m^2 \cdot se_s^2 + se_m^2 \cdot \mu_s^2 + se_s^2 \cdot \mu_m^2} \quad (4)$$

Where $se_a, se_{m,}$ and se_s are the SE of the adjusted survey data point, the adjustment factor, and the survey data point, respectively. μ_s and μ_m are the means of the survey data point and the adjustment factor.

We present the predicted adjustment factors by age for inpatient utilisation rates with one-year recall (Figure S3), for inpatient coverage with one-year recall (Figure S4), and inpatient coverage with one-month recall (Figure S5). The adjustment factors for inpatient coverage with one-year recall were higher than inpatient utilisation with the same recall, because they adjust for both recall and reported measures.

Figure S3. Global age-sex specific adjustment factors applied to surveys reporting one-year recall of inpatient utilisation rate

Adjustment factors were estimated from cross-validated age-spline regressions where the dependent variable was the ratio of annual inpatient utilisation rates reported by reference data sources to survey data sources reporting one-year recall of inpatient utilisation rates, matched on age, sex, location, and year. Estimated adjustment factors are denoted by the blue lines along with their associated predictions intervals shaded in grey. Data points (ratios) in which the adjustment factors were estimated on are displayed as black circles.

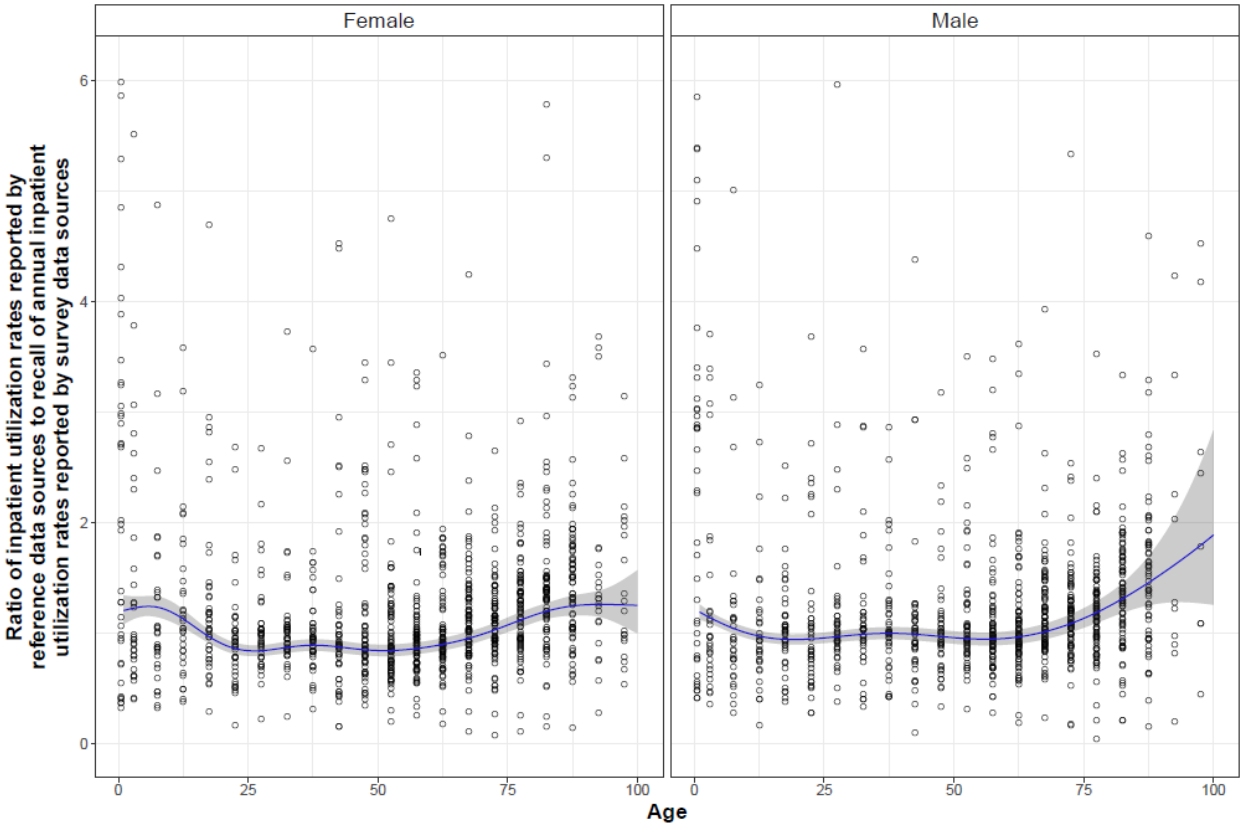

Figure S4. Global age-sex specific adjustment factors applied to surveys reporting coverage of hospital admissions in the past year

Adjustment factors were estimated from cross-validated age-spline regressions where the dependent variable was the ratio of annual inpatient utilisation rates reported by reference data sources to survey data sources reporting proportion of respondents admitted into hospital in the past year, matched on age, sex, location, and year. Estimated adjustment factors are denoted by the blue lines along with their associated predictions intervals shaded in grey. Data points (ratios) in which the adjustment factors were estimated on are displayed as black circles.

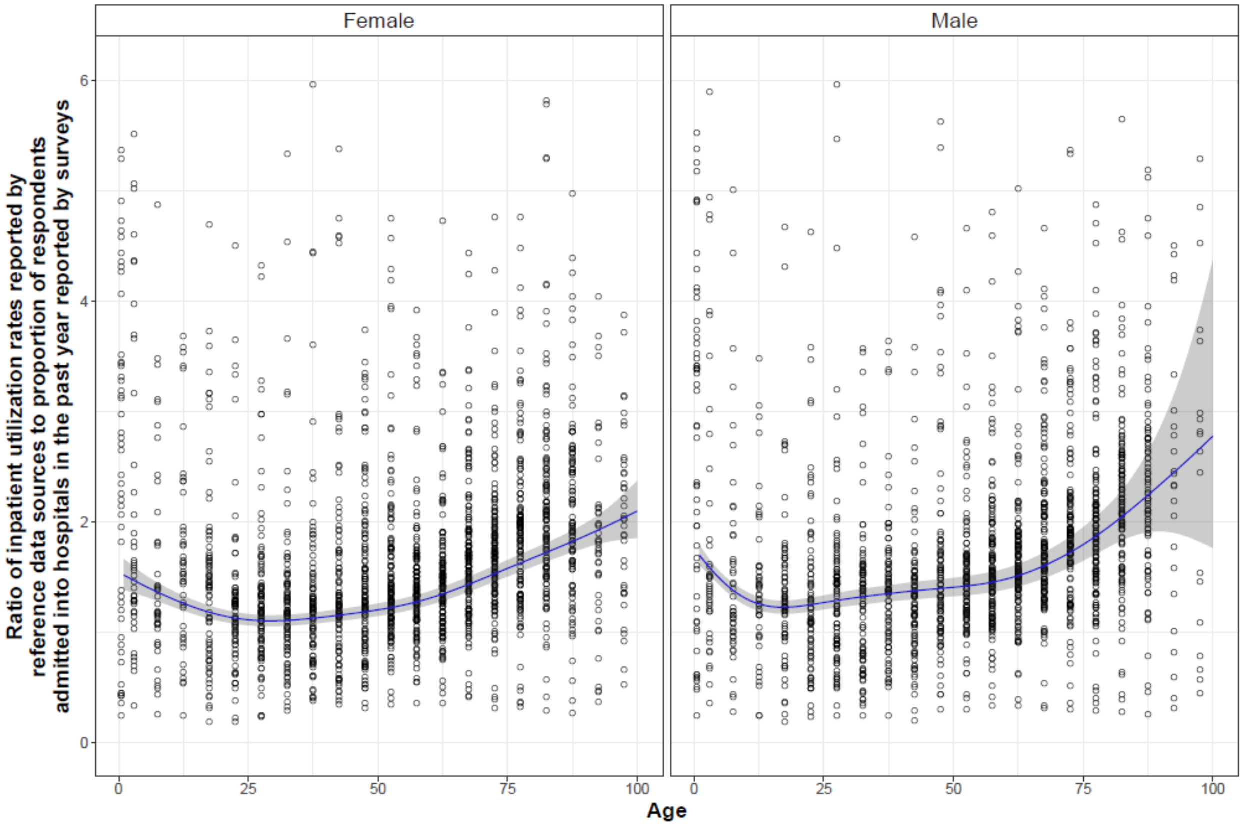

Figure S5. Global age-sex specific adjustment factors applied to surveys reporting coverage of hospital admissions in the past month

Adjustment factors were estimated from cross-validated age-spline regressions where the dependent variable was the ratio of annual inpatient utilisation rates reported by reference data sources to survey data sources reporting proportion of respondents admitted into hospital in the past month, matched on age, sex, location, and year. Estimated adjustment factors are denoted by the blue lines along with their associated predictions intervals shaded in grey. Data points (ratios) in which the adjustment factors were estimated on are displayed as black circles.

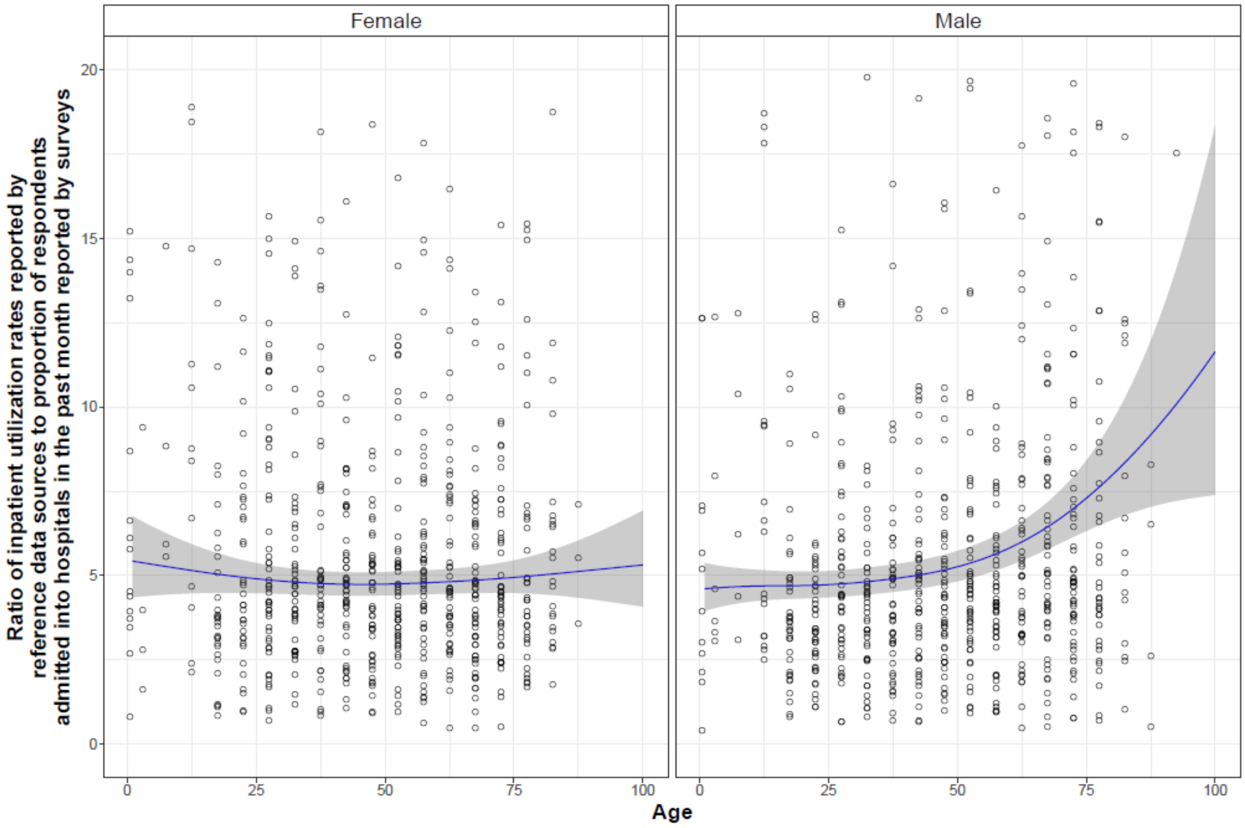

3.4.2. Outpatient data adjustments for data varying by age and sex

For outpatient coverage data with a one-year recall (Figure S6), we estimated adjustment factors using the reference data and penalised spline regression as described in Section 3.4.1 on inpatient data adjustments for data varying by age and sex above.

For outpatient data on coverage with shorter recall (i.e. two weeks, 30 days, and three months), we did not directly adjust these data to reference data. For each coverage recall period, we looked for overlap with a reliable utilisation data source on the basis of age, sex, location, and year. We adjusted the proportions with any use of service to number of visits per persons using surveys with similar recall periods. We ran sex specific penalised-spline regressions on all outpatient data with a recall of less than one year and used dichotomous covariates to denote surveys with a two-week and three-month recall as shown in equation (5):

$$\ln\left(\frac{\mu_{rate,i}}{\mu_{proportion,i}}\right) = \beta_0 + h(age_i) + \beta_1 x_{2week,i} + \beta_2 x_{3\,month,i} + \varepsilon_i \ (5)$$

Figure S6. Global age-sex specific adjustment factors applied to surveys reporting coverage of outpatient services in the past year

Adjustment factors were estimated from cross-validated age-spline regressions where the dependent variable was the ratio of annual outpatient utilisation rates reported by reference data sources to survey data sources reporting proportions of respondents who used outpatient services in the past year, matched on age, sex, location, and year. Estimated adjustment factors are denoted by the blue lines along with their associated predictions intervals shaded in grey. Data points (ratios) in which the adjustment factors were estimated on are displayed as black circles.

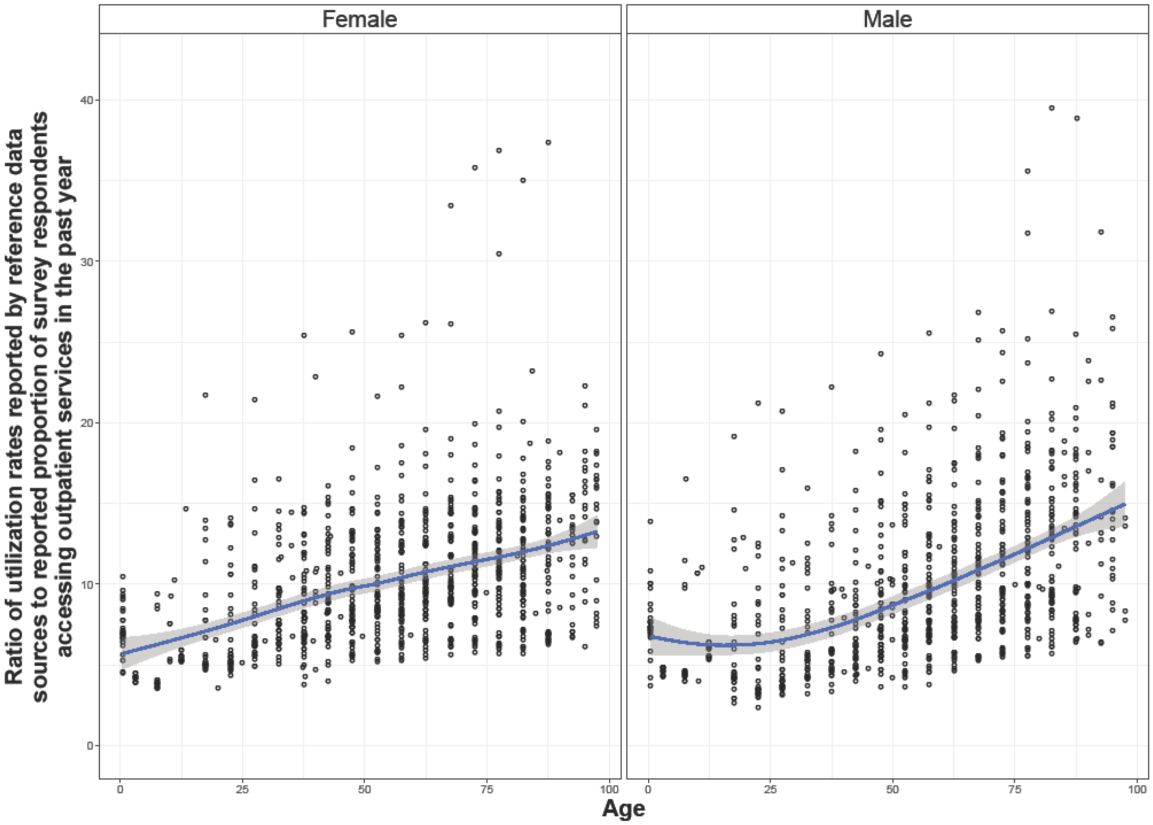

Where i denotes a given matched observation, $h(age_i)$ represents a basis function that estimated a cross-validated penalised spline over the population weighted mean age of the age category, μ_{rate} and $\mu_{proportion}$ are the point estimates of survey data reporting utilisation rates and proportions respectively, x_{2week} and $x_{3\ month}$ are dichotomous covariates denoting surveys with a two-week or three-month recall, and ε represents the residual. This adjustment assumed that surveys with a two-week and three-month recall systematically differed from surveys with 30-day recall, as estimated by β_1 and β_2 in equation (5). Exponentiated predictions from these regressions were multiplied by reported survey proportions and errors were propagated with equation (4). We display the predicted regressions fits (Figure S7). The adjusted data were treated as survey reported utilisation rates with a two week, 30-day, or three-month recall.

Figure S7. Global age-sex specific adjustment factors applied to surveys reporting coverage of outpatient services in the past two weeks, 30 days, and three months

Adjustment factors were estimated from cross-validated age-spline regressions where the dependent variable was the ratio of outpatient utilisation rates reported by surveys to proportion of people who used outpatient services reported by surveys in the past two weeks, 30 days, or three months, matched on recall period, age, sex, location, and year. Estimated adjustment factors are denoted by the blue lines along with their associated predictions intervals shaded in grey. Data points (ratios) in which the adjustment factors were estimated on are displayed as black circles.

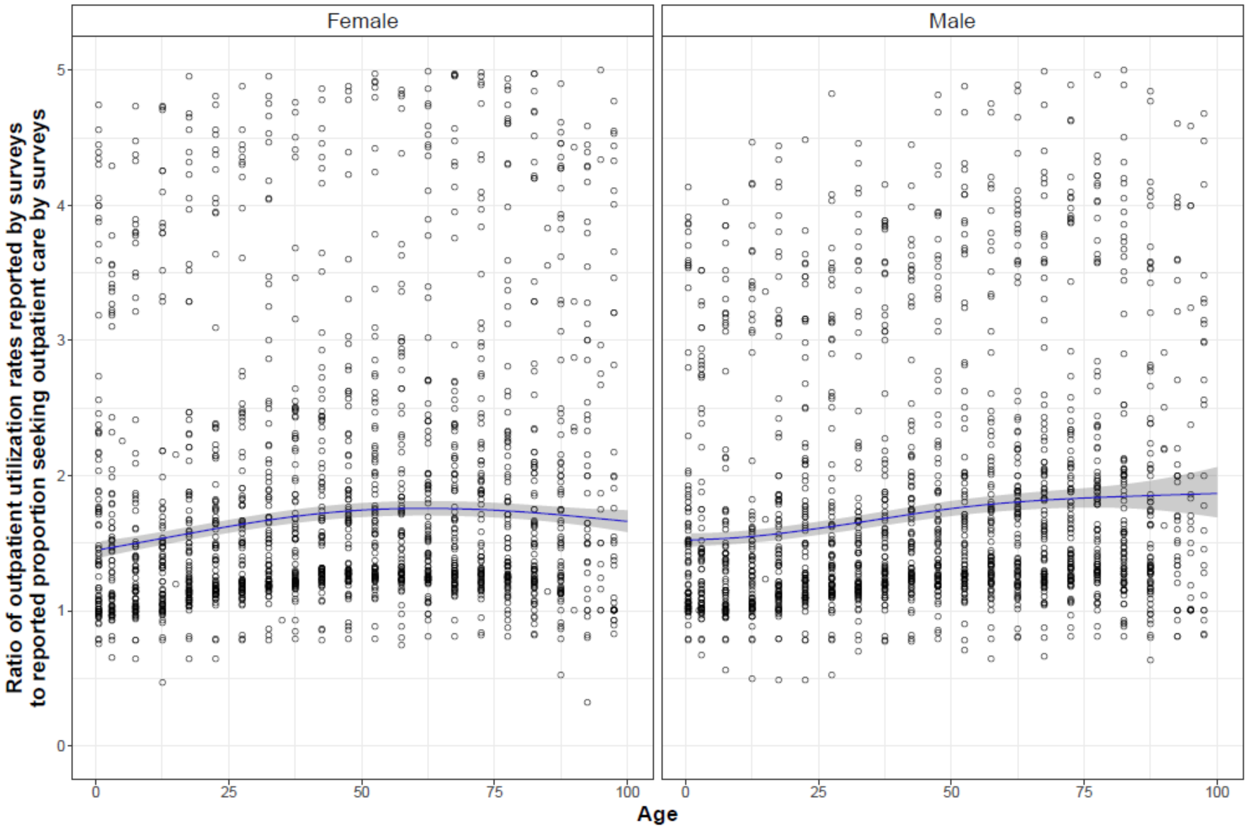

Section 4. Estimating the utilisation models

4.1. Introduction

We applied a Bayesian hierarchal metaregression method referred to as DisMod- MR-2.1 to analyse the extracted data and estimate age-sex-year-location specific rates of utilisation.[6,7] DisMod-MR offers two key advantages over other multiple regression methods: 1) it incorporates priors from a geographic hierarchy to improve estimates in countries with little to no data, and 2) it leverages age splines to draw power from neighbouring age categories and estimates age-sex specific patterns. Data points from a few data sources (n=42) were outliered when they systematically differed from neighbouring data points.

4.2. Quantifying and adjusting for differences across data sources in DisMod-MR

When the survey data systematically and multiplicatively differed from reference data points, we adjusted the data using dichotomous covariates within DisMod-MR 2.1. For the outpatient utilisation model, we created four covariates for survey recall periods: two-week, 30-day, three-month recall, and one year (Table S9). We also created two covariates for special phrasing of the utilisation questions that: 1) did not distinguish outpatient and inpatient services, and 2) asked only about utilisation due to illness or injury. For the inpatient utilisation model, we created covariates for two survey series: 1) World Health Survey, and 2) WHO's Multi-country Survey Study on Health and Health System Responsiveness.

The exponentiated beta value of the coefficient was the adjustment applied to the survey data type across age and sex categories. For example, the result for surveys reporting two-week recall was 0.036 (95%UI 0.035-0.037), and meant that reported utilisation during a two-week time period would, on average, be adjusted by 1/0.036 or a factor 27.78 to represent annual utilisation. Final posterior estimates were made with dichotomous covariates assuming the value of zero.

4.3. Additional covariates and their definitions

The DisMod-MR models also included Socio-demographic Index (SDI) as a covariate for outpatient utilisation, and hospital beds per 1,000 population for inpatient utilisation. Utilisation of health services is positively, and statistically significantly related to income at the national level in global estimates,[8] and to education.[9] In global data at the country level however, income, education, and fertility were highly correlated, which creates multi-collinearity when all three variables are included in regression analyses. SDI is a single variable that summarises the information, and produces stable regression estimates. In the estimate of utilisation of outpatient visits, the coefficient for SDI was statistically significant.

The GBD 2016 SDI was calculated from three variables: lag-distributed income, where income is Gross National Product per capita, educational attainment among people over 15 years of age, and total fertility rate.[11]Lag-distributed income is gross national product per capita in international dollars distributed over ten years, with larger weights for more recent years. Each variable was scaled from zero to one where zero represented the lowest observed value of lagged distributed income and education, and the highest observed value for total fertility rate for a country between 1990 to 2016; one represented the other end of the range. SDI was the geometric mean of the rescaled values.

Hospital beds per 1,000 population measured of the capacity associated with greater utilisation and resources. In the estimate of utilisation of inpatient admissions, its coefficient was statistically significant. The relationship between utilisation of inpatient admissions and SDI was less clear. Demand for inpatient care is less responsive (elastic) to income. Also, maternity care wa a large share of inpatient admissions, meaning that admissions were positively related to the total fertility rate, instead of the negative relationship measured by SDI.

Hospital beds per 1,000 was based on World Development Indicator data.[12] To create a complete time-series of hospital beds per 1,000 for all locations, we used Spatiotemporal Gaussian Process Regression (ST-GPR) to interpolate data for missing locations and years. The ST-GPR is a data synthesis method widely used in GBD.[13] Briefly, ST-GPR is a three-step model. In the first step of the modeling process we run a linear regression with a set of explanatory variables to predict general trends in the data. In the second step, we smooth over the residuals

Table S9. Estimated coefficients of the outpatient and inpatient utilisation model

Study-level covariates denote global dichotomous covariates that adjusted corresponding data points.

Model	Covariate name	Covariate type	Beta value (95% UI)	Exponentiated beta value (95% UI)
**Outpatient utilisation**	Surveys reporting two-week recall	Study-level	-3.32 (-3.34 — -3.31)	0.036 (0.035 — 0.037)
	Surveys reporting 30 day recall	Study-level	-2.77 (-2.78 — -2.76)	0.063 (0.062 — 0.063)
	Surveys reporting three-month recall	Study-level	-1.68 (-1.72 — -1.65)	0.19 (0.18 — 0.19)
	Surveys reporting one-year recall	Study-level	-0.53 (-0.54 — -0.51)	0.59 (0.58 — 0.60)
	Survey reports utilisation measure only when person in household had a illness or injury	Study-level	-0.15 (-0.16 — -0.14)	0.86 (0.85 — 0.87)
	Surveys reports utilisation measure without distinguishing outpatient or inpatient	Study-level	0.081 (0.064 — 0.10)	1.08 (1.07 — 1.11)
	Socio-demographic Index	Country-level	0.83 (0.78 — 0.87)	2.28 (2.18 — 2.39)
**Inpatient utilisation**	World Health Survey	Study-level	0.44 (0.39 — 0.48)	1.55 (1.48 — 1.61)
	World Health Organization's Multi-country Survey Study on Health and Responsiveness	Study-level	0.49 (0.42 — 0.55)	1.63 (1.52 — 1.73)
	log hospital beds per 1,000	Country-level	0.17 (0.16 — 0.19)	1.19 (1.17 — 1.20)

of the linear regression to account for variation across time and space. These smoothed residuals were subsequently added to the predictions of the linear regression estimated in the first step to create a spatiotemporal prediction. In the third step, we passed the spatiotemporal predictions as a prior to GPR's mean function and an input into GPR's covariance function.

4.4. Age-sex splitting

When measures were reported in wide age intervals (e.g. all ages), we did age-splitting. DisMod-MR 2.1 is capable of conducting age-integration but its performance degrades while integrating across wide age categories. To remedy this issue, we ran a preliminary DisMod-MR 2.1 model with data that were disaggregated by age to estimate countries' age-pattern and then we applied the estimated age-pattern to split aggregated all age data. This procedure was operationalised by calculating a constant, k, which was the ratio of the aggregated all age data point, $\mu_{all\ age}$, to the all age estimated utilisation rate from the DisMod-MR 2.1 model, $\widehat{\mu_d}$, as shown in equation (6):

$$k = \frac{\mu_{all\ age}}{\widehat{\mu_d}} \quad (6)$$

The constant, k, was then multiplied by age-specific utilisation rates from the DisMod-MR 2.1 model. The uncertainty from the data and the age-pattern were propagated following equation 4. The split data was then incorporated into final DisMod-MR 2.1 estimates.

4.5. Raking DisMod-MR 2.1 estimates

DisMod-MR 2.1 produced accurate estimates in locations with little to no data through leveraging the geographical hierarchy (Table S4). This process is operationalised by first fitting a model at the global level on all available data. Estimates from the global model serve as Bayesian priors to super-region models estimated on data specific to the super-region. In turn, super-region models serve as priors to regional models, regional models serve as priors to national models, and, in nations with subnational estimates, national models serve as priors to subnational models. To generate final national level estimates in nations where subnational estimates exist, by default DisMod-MR 2.1

aggregates subnational estimates to the national level. This process may lead to inconsistencies between the final aggregated national level estimate and national administrative records, especially in nations where subnational data on utilisation do not exist.

To resolve this issue, we raked (scaled) subnational estimates to match the fitted national estimates. This is in contrast to aggregating subnational estimates to replace fitted national estimates. Raking only occurred in countries where we did not have subnational level data on utilisation. For our outpatient model, we raked estimates Japan, South Africa, Kenya, England, Saudi Arabia, United States, and Sweden. In our inpatient model we raked estimates in Japan, Kenya, England, Saudi Arabia, and Sweden. The lone exception to this rule was China. We also raked China estimates, because the national estimates based on several years of data indicated a strong time trend that disappeared when subnational estimates based on a single year of data were aggregated to the national level.

4.6. Understanding the impact of missing data on DisMod-MR 2.1 estimates

Although DisMod-MR produces estimates for locations with little to no data, the impact of this method was that country-level results were similar to the regional results for those locations. When no data were available for a country, the final estimate was based on the regional prior, and covariates. When data were available, the final estimate gave more weight to the country data than the regional prior, as well as the covariates.

An example of the impact is the decomposition analysis for sub-Saharan Africa, where results were more similar across countries than other super-regions. We had 91 country-years of outpatient data and 51 country-years of inpatient data for 55 countries. Results for countries with more data differed from the other countries in the region. For example, in the Southern Africa region, we had 32 country-years of outpatient data and three country-years of inpatient data from South Africa, one country-year of inpatient data Namibia, Swaziland, and Zimbabwe, and no data from Botswana and Lesotho. The South African estimates showed a substantial decrease in the outpatient utilisation rate, but not the other countries' estimates. Note that the final regional estimate differed from the regional prior (not shown), because it included the country-level South African results.

4.7. Internal validity of results

To examine the internal validity of the estimates we compared utilisation rates estimated with all available data to rates from reference data sources (Table S5). This internal validation exercise was limited to country-years that had both reference data and survey data. This validation process identified the problem with the subnational estimates in countries that did not have subnational utilisation data described in Section 4.5 above. Outpatient (Figure S8) and inpatient (Figure S9) utilisation per capita from the final DisMod 2.1 estimates with all data sources, including raked estimates for eight countries, by country-year, were compared to the reference the reference data. The comparison showed good agreement between the final estimated rates and the reference data.

4.8. Age standardisation

To facilitate comparisons across countries with different age structures in Figures 1 and 2, we estimated age-standardised utilisation rates. Age-standardisation was the process of applying age-specific rates for each location to a population with a constant global age structure and aggregating the estimate to a national level. We used the constant global age structure developed by the GBD 2013 collaborators, which reflected the percentage of the population in each age category based on the United Nations Population Division's World Population Prospects for all countries for the years 2010-2035.[14]

Figure S8. Internal validation of outpatient utilisation rates reported by reference group compared to estimated outpatient utilisation rate

Displayed utilisation rates were not age-standardised, because the comparison is between country-years.

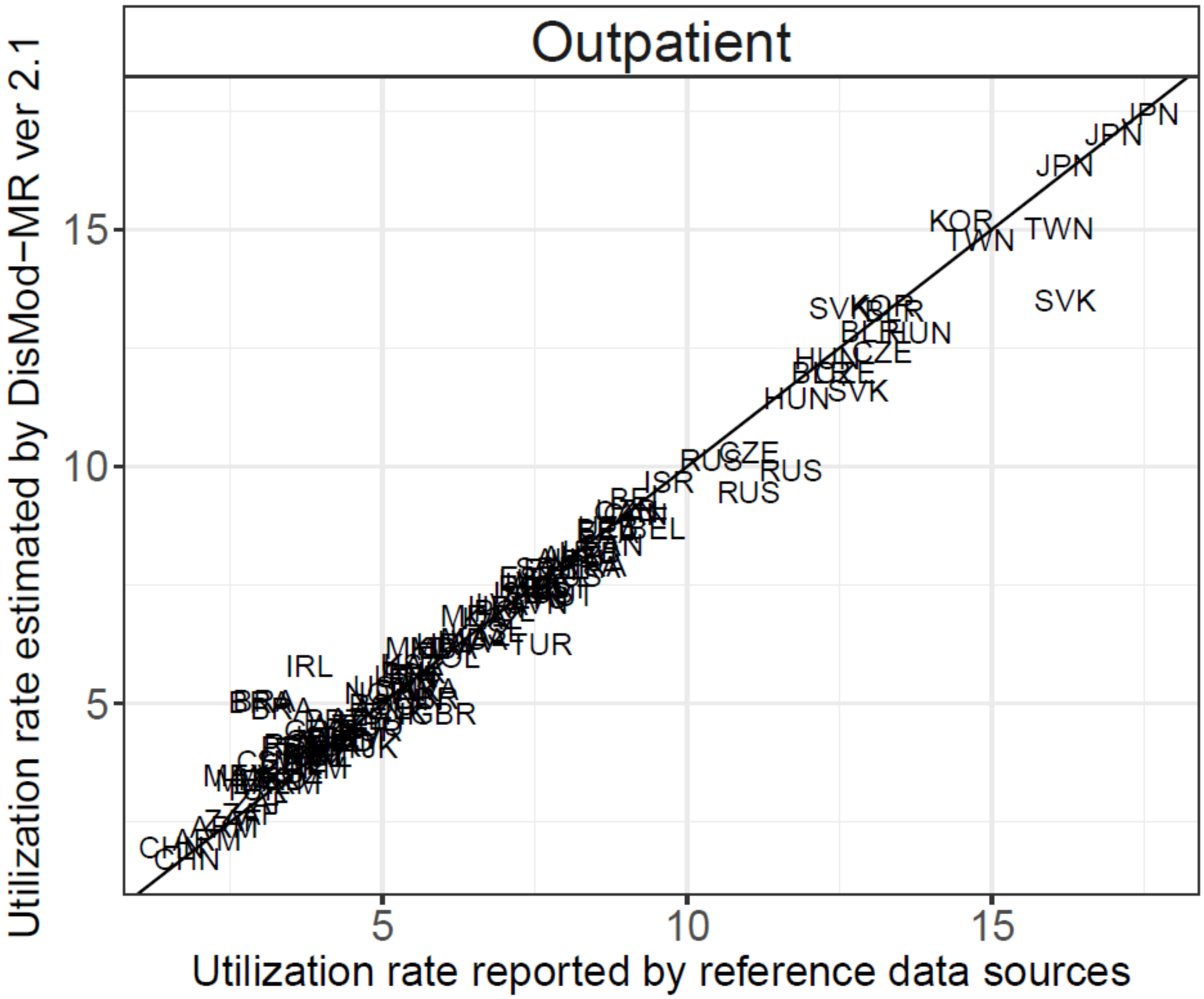

Figure S9. Internal validation of inpatient utilisation rates reported by reference group compared to estimated inpatient utilisation rate.

Displayed utilisation rates were not age-standardised, because the comparison is between country-years.

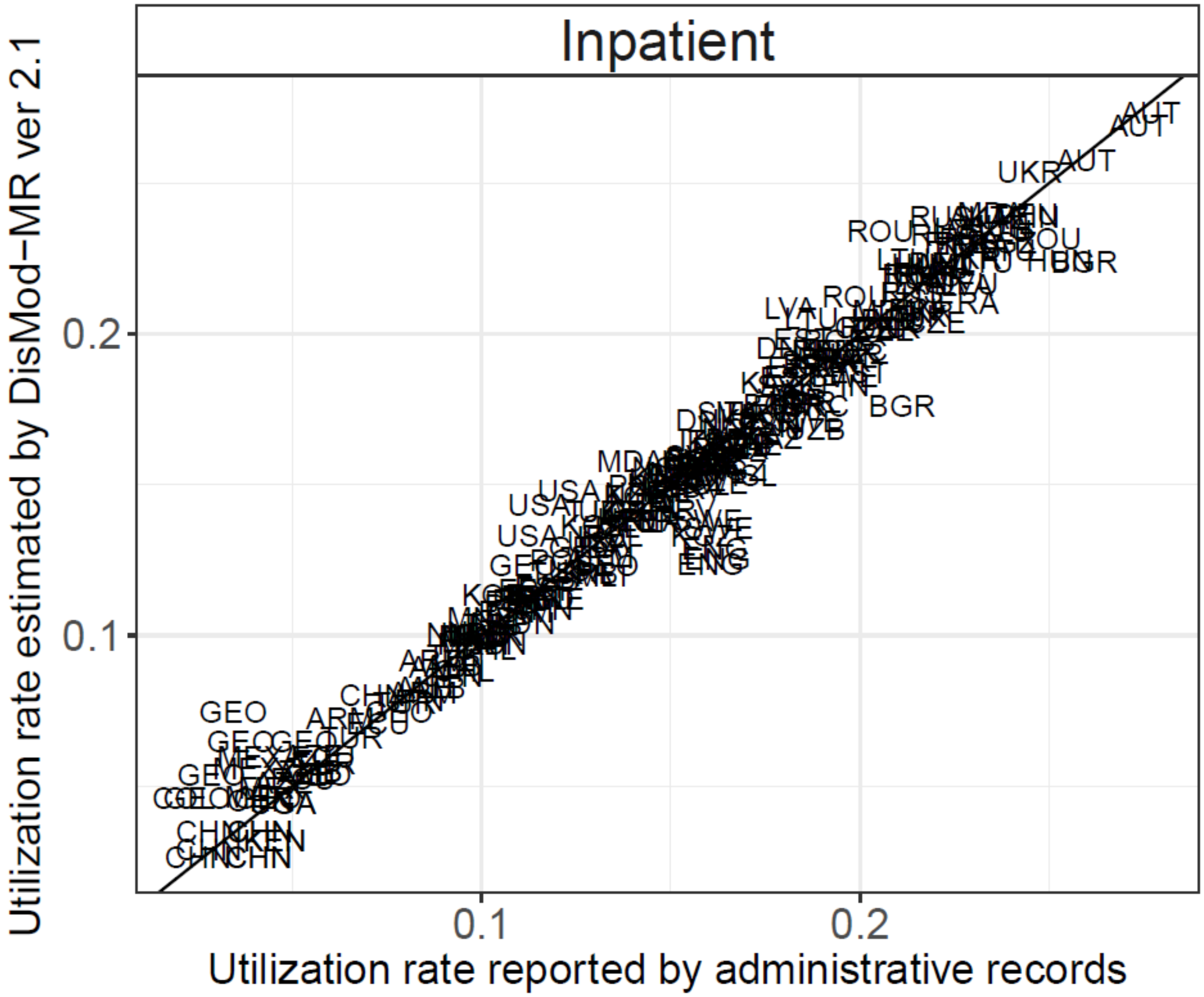

Section 5. Utilisation results

5.1. Age and sex specific utilisation rates

In all super-regions in 2016, outpatient utilisation rates increased with age beginning in early adulthood (Figure S10). The increase was steepest among men in South Asia, and less pronounced in Latin America and the Caribbean and in sub-Saharan Africa. Inpatient utilisation rates increased among women during reproductive age categories (10-54 years), and then increased with age among women and men in later adulthood (Figure S11). Utilisation rates were higher for women than men in reproductive age categories, and similar in younger and older age categories (Figure S12).

5.2. Country-specific utilisation rates from 1990-2016

Figure S10. Global and GBD super-region estimates of outpatient utilisation rate in 2016 by age and sex

Bold lines represent the estimated mean number of outpatient visits per person from 1,000 draws, and the dashed lines in the same colour are the uncertainty intervals defined as the 2.5th and 97.5th percentile of draws. GBD= Global Burden of Disease

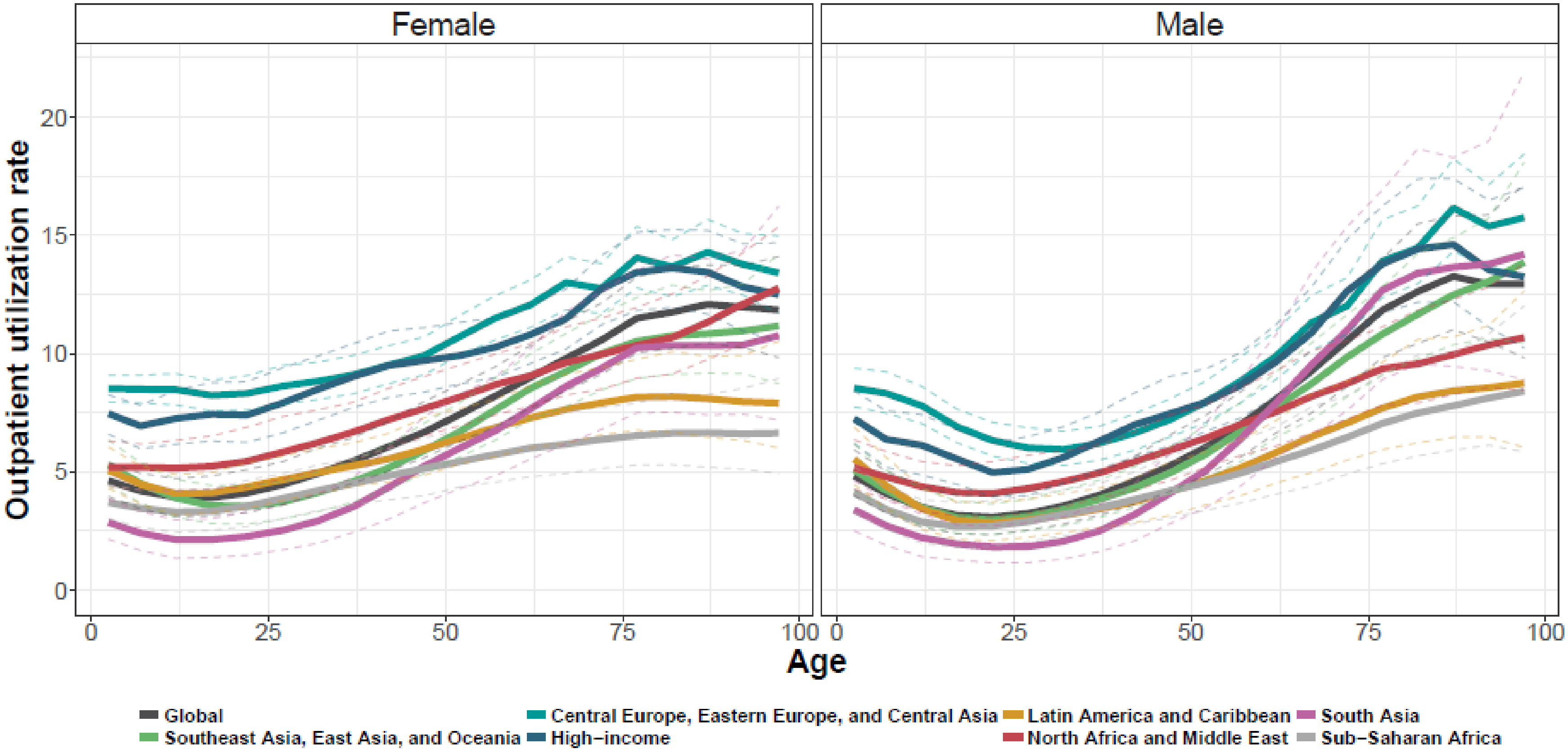

Figure S11. Global and GBD super-region estimates of inpatient utilisation rate in 2016 by age and sex

Bold lines represent the estimated mean number of inpatient admissions per person from 1,000 draws, and the dashed lines in the same colour are the uncertainty intervals defined as the 2.5th and 97.5th percentile of draws. GBD= Global Burden of Disease

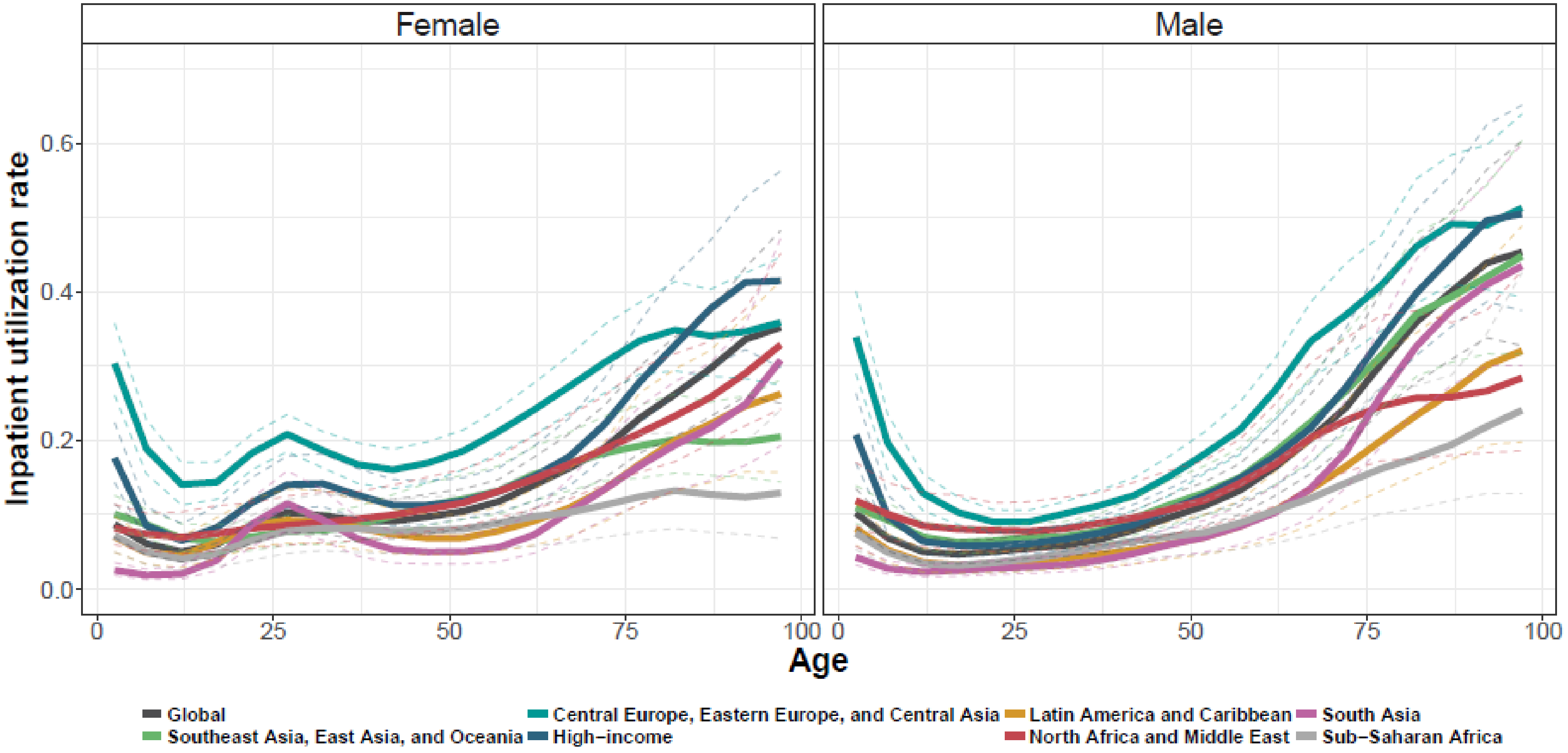

Figure S12. Ratio of female to male global and GBD super-region estimates of outpatient and inpatient utilisation rate in 2016 by age

Bold lines represent the estimated mean of the ratio of female utilisation rate to the male utilisation rate from 1,000 draws, and the dashed lines in the same colour are the uncertainty intervals defined as the 2.5th and 97.5th percentile of draws. The horizontal line at one shows where female and male utilisation rates would be the same. GBD= Global Burden of Disease

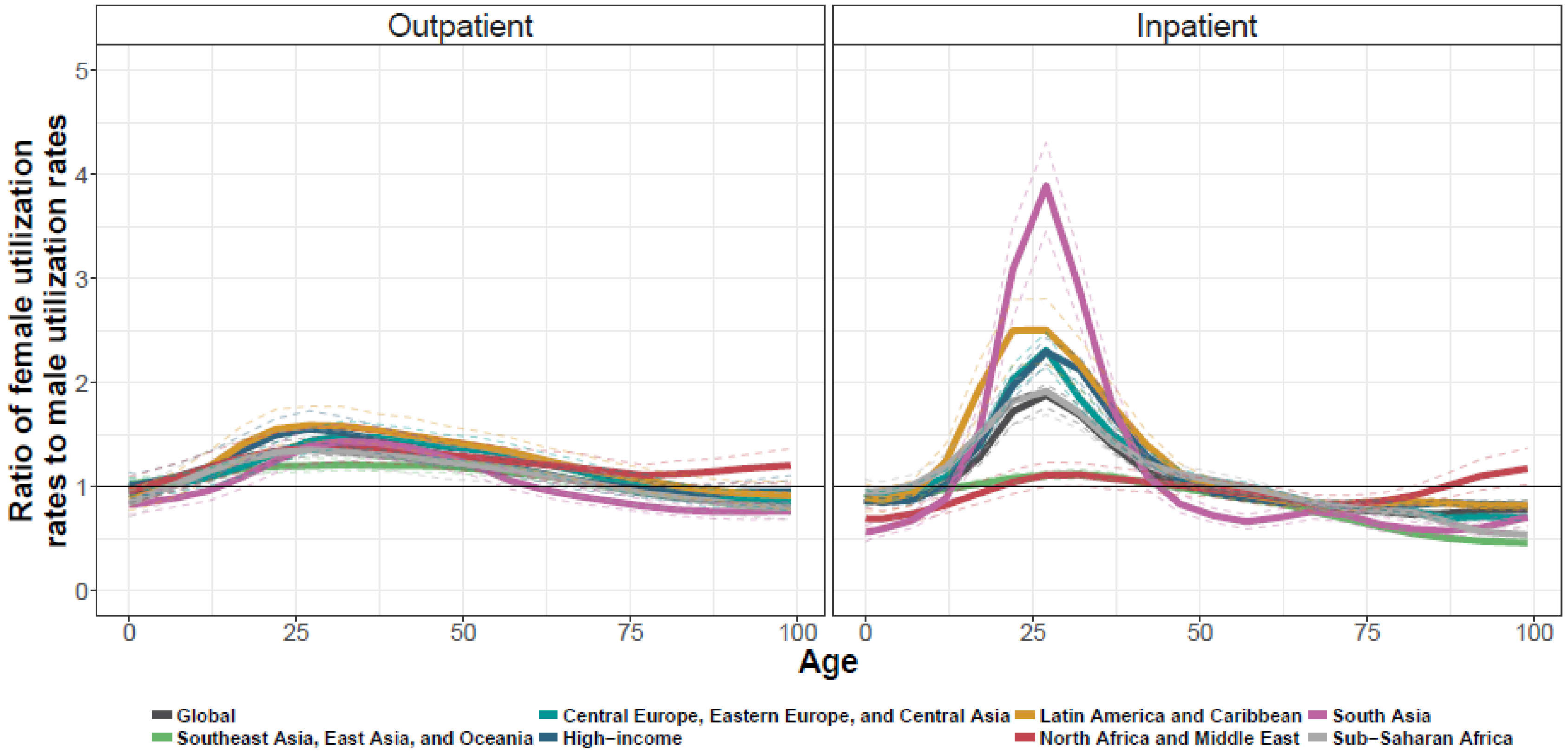

Table S10. Global, GBD super-region, regional, and national age-standardised outpatient utilisation rates and annualised rate of change 1990 to 2016, and volume of outpatient visits in 2016 and percentage change in volume from 1990 to 2016, both sexes combined

Table displays four sets of results: age-standardised outpatient utilisation rate per person defined as the number of outpatient visits per person per year, the annualised rate of change in the age-standardised outpatient utilisation rate, volume of outpatient visits in 2016, and the percentage change in volume from 1990 to 2016. For each result, the mean of 1,000 draws is reported, and in parenthesis the uncertainty interval defined as the 2.5th and 97.5th percentile of draws. GBD= Global Burden of Disease.

Table S10: Global, GBD super-region, regional, and national age-standardised outpatient utilisation rates and annualised rate of change 1990 to 2016, and volume of outpatient visits in 2016 and percentage change in volume from 1990 to 2016, both sexes combined

	Age standardised for international comparisons							Estimates based on national age structure	
	Age standardised outpatient utilisation rate (per capita)						Annual rate of change in age-standardised rate (as percentage)	Volume of outpatient visits per year (in thousands)	Total percent age change (Figure 3)
	1990	1995	2000	2005	2010	2016 (Figure 1a)	1990 - 2016	2016	1990 - 2016
Global	5·06<br>(4·5 – 5·69)	4·6<br>(4·16 – 5·04)	4·58<br>(4·14 – 5·04)	4·7<br>(4·32 – 5·08)	4·8<br>(4·38 – 5·22)	5·42<br>(4·88 – 5·98)	0·26<br>(0·17 – 0·39)	39 349 571<br>(35 381 140 – 43 581 880)	58·65
Central Europe, Eastern Europe, and Central Asia	7·85<br>(7·04 – 8·76)	7·16<br>(6·72 – 7·59)	7·64<br>(7·34 – 7·96)	8·13<br>(7·84 – 8·43)	8·06<br>(7·77 – 8·33)	8·74<br>(8·37 – 9·12)	0·41<br>(0·13 – 0·7)	3 754 868<br>(3 598 848 – 3 910 868)	15·81
Central Asia	6·76<br>(5·8 – 7·82)	5·98<br>(5·59 – 6·39)	6·2<br>(6·02 – 6·39)	6·49<br>(6·32 – 6·66)	6·65<br>(6·48 – 6·81)	6·84<br>(6·62 – 7·06)	0·04<br>(-0·42 – 0·56)	589 912<br>(570 621 – 610 569)	31·18
Armenia	3·03<br>(2·56 – 3·48)	2·48<br>(2·34 – 2·63)	2·14<br>(2·09 – 2·19)	2·41<br>(2·34 – 2·49)	3·31<br>(3·25 – 3·38)	3·78<br>(3·66 – 3·9)	0·86<br>(0·35 – 1·43)	11 874<br>(11 480 – 12 250)	12·95
Azerbaijan	5·24<br>(4·49 – 6·1)	5·49<br>(5·13 – 5·84)	5·07<br>(4·92 – 5·21)	4·81<br>(4·7 – 4·93)	4·65<br>(4·54 – 4·76)	4·7<br>(4·54 – 4·87)	-0·42<br>(-0·96 – 0·16)	45 896<br>(44 265 – 47 685)	26·85
Georgia	5·73<br>(4·88 – 6·86)	5·62<br>(4·79 – 6·49)	5·55<br>(4·75 – 6·47)	5·57<br>(4·76 – 6·59)	5·71<br>(4·96 – 6·72)	5·86<br>(5·05 – 6·96)	0·09<br>(-0·18 – 0·4)	24 457<br>(21 169 – 28 907)	-22·21
Kazakhstan	7·05<br>(6·06 – 8·22)	6·07<br>(5·66 – 6·46)	5·95<br>(5·78 – 6·11)	6·58<br>(6·41 – 6·73)	6·92<br>(6·77 – 7·09)	6·76<br>(6·54 – 6·99)	-0·16<br>(-0·7 – 0·4)	119 225<br>(115 221 – 123 445)	4·5
Kyrgyzstan	4·53<br>(3·96 – 5·24)	5·15<br>(4·85 – 5·43)	4·57<br>(4·46 – 4·67)	4·11<br>(4·04 – 4·19)	3·72<br>(3·65 – 3·8)	3·63<br>(3·53 – 3·73)	-0·85<br>(-1·38 to - 0·38)	20 820<br>(20 236 – 21 441)	9·78
Mongolia	4·98<br>(4·2 – 5·76)	5·17<br>(4·39 – 6·03)	5·32<br>(4·54 – 6·25)	5·34<br>(4·57 – 6·36)	5·41<br>(4·59 – 6·25)	5·51<br>(4·72 – 6·47)	0·4<br>(0·12 – 0·67)	16 220<br>(13 805 – 19 244)	58·44
Tajikistan	4·88<br>(4·09 – 5·82)	3·92<br>(3·66 – 4·22)	4·33<br>(4·19 – 4·46)	4·41<br>(4·29 – 4·53)	4·38<br>(4·26 – 4·49)	4·8<br>(4·64 – 4·96)	-0·06<br>(-0·69 – 0·55)	39 062<br>(37 635 – 40 584)	62·02
Turkmenistan	4·09<br>(3·48 – 4·9)	4·19<br>(3·53 – 4·95)	4·51<br>(4·36 – 4·66)	4·15<br>(4·05 – 4·25)	3·83<br>(3·74 – 3·92)	3·68<br>(3·56 – 3·81)	-0·4<br>(-1·06 – 0·19)	19 458<br>(18 739 – 20 162)	38·04
Uzbekistan	9·76<br>(8·2 – 11·41)	7·88<br>(7·44 – 8·32)	8·71<br>(8·52 – 8·91)	9·27<br>(9·08 – 9·46)	9·53<br>(9·35 – 9·72)	9·99<br>(9·71 – 10·28)	0·09<br>(-0·45 – 0·71)	292 900<br>(284 110 – 301 880)	54·12
Central Europe	7·65<br>(6·61 – 8·87)	6·78<br>(6·38 – 7·2)	7·03<br>(6·79 – 7·27)	7·42<br>(7·26 – 7·6)	7·48<br>(7·29 – 7·67)	7·52<br>(7·23 – 7·81)	-0·06<br>(-0·52 – 0·43)	920 135<br>(886 691 – 952 737)	-3·01
Albania	4·79<br>(4·11 – 5·56)	4·9<br>(4·21 – 5·79)	4·46<br>(3·92 – 5·13)	4·25<br>(3·82 – 4·81)	5·3<br>(4·53 – 6·12)	5·44<br>(4·63 – 6·31)	0·48<br>(0·16 – 0·85)	16 081<br>(13 638 – 18 757)	5·58
Bosnia and Herzegovina	8·22<br>(7·08 – 9·63)	8·33<br>(7·09 – 9·56)	9·56<br>(8·38 – 10·83)	7·16<br>(6·28 – 8·52)	9·66<br>(8·42 – 11·32)	9·71<br>(8·54 – 11·43)	0·64<br>(0·23 – 0·99)	38 642<br>(34 174 – 45 107)	5·26
Bulgaria	5·87<br>(5·07 – 7·16)	4·55<br>(4·19 – 4·97)	4·91<br>(4·59 – 5·23)	5·27<br>(4·91 – 5·62)	5·94<br>(5·7 – 6·17)	5·58<br>(5·23 – 5·92)	-0·19<br>(-0·8 – 0·26)	43 761<br>(41 097 – 46 378)	-17·84
Croatia	6·81<br>(5·72 – 7·9)	6·77<br>(5·77 – 7·95)	6·8<br>(6·54 – 7·05)	6·71<br>(6·58 – 6·85)	5·85<br>(5·73 – 5·96)	6·01<br>(5·81 – 6·22)	-0·48<br>(-1·04 – 0·14)	27 452<br>(26 523 – 28 425)	-17·38
Czech Republic	11·81<br>(10·12 – 13·36)	12·12<br>(11·55 – 12·7)	11·8<br>(11·52 – 12·07)	12·06<br>(11·82 – 12·32)	9·98<br>(9·65 – 10·3)	10·35<br>(9·9 – 10·86)	-0·51<br>(-0·87 – 0·01)	114 798<br>(110 551 – 119 834)	-7·22
Hungary	12·36<br>(10·74 – 14·4)	10·41<br>(9·9 – 10·92)	11·09<br>(10·6 – 11·55)	12·3<br>(12·06 – 12·54)	11·78<br>(11·54 – 12·03)	11·94<br>(11·56 – 12·33)	-0·13<br>(-0·68 – 0·33)	125 899<br>(121 841 – 129 909)	-3·94
Macedonia	4·8<br>(4·09 – 5·56)	3·32<br>(3·11 – 3·57)	3·41<br>(3·33 – 3·49)	4·19<br>(4·05 – 4·34)	6·07<br>(5·91 – 6·23)	6·53<br>(6·27 – 6·79)	1·18<br>(0·67 – 1·68)	13 993<br>(13 415 – 14 569)	48·25
Montenegro	7·36<br>(6·3 – 8·66)	7·33<br>(6·37 – 8·74)	7·34<br>(6·29 – 8·68)	7·48<br>(6·36 – 8·89)	7·59<br>(6·49 – 8·72)	6·98<br>(6·57 – 7·37)	-0·2<br>(-0·73 – 0·25)	4556<br>(4305 – 4812)	1·57
Poland	7·01<br>(5·89 – 8·31)	5·45<br>(5·18 – 5·72)	5·89<br>(5·79 – 6·01)	6·78<br>(6·64 – 6·91)	7·17<br>(7·02 – 7·31)	7·03<br>(6·77 – 7·3)	0·01<br>(-0·6 – 0·62)	285 941<br>(276 324 – 296 022)	6·94
Romania	4·74<br>(4·04 – 5·79)	4·02<br>(3·79 – 4·27)	4·01<br>(3·9 – 4·12)	4·4<br>(4·32 – 4·49)	4·75<br>(4·66 – 4·85)	4·68<br>(4·48 – 4·87)	-0·05<br>(-0·74 – 0·51)	96 637<br>(92 871 – 100 207)	-13·9
Serbia	8·06<br>(6·87 – 9·43)	8·12<br>(6·92 – 9·41)	7·96<br>(7·28 – 8·62)	7·46<br>(7·19 – 7·74)	7·28<br>(7·12 – 7·47)	7·3<br>(7·06 – 7·53)	-0·38<br>(-0·93 – 0·21)	68 195<br>(65 925 – 70 283)	-11·48
Slovakia	13·24<br>(11·41 – 15·33)	12·75<br>(12·31 – 13·18)	13·45<br>(13·0 – 13·84)	13·08<br>(12·83 – 13·33)	11·36<br>(11·0 – 11·73)	12·02<br>(11·61 – 12·42)	-0·37<br>(-0·9 – 0·18)	68 753<br>(66 438 – 70 949)	-1·36
Slovenia	7·52<br>(6·55 – 8·97)	7·4<br>(6·43 – 8·53)	7·55<br>(6·44 – 8·84)	7·44<br>(7·2 – 7·69)	6·82<br>(6·67 – 6·98)	7·0<br>(6·78 – 7·22)	-0·27<br>(-0·94 – 0·22)	15 426<br>(14 964 – 15 875)	1·28
Eastern Europe	8·38<br>(7·76 – 9·1)	7·87<br>(7·38 – 8·34)	8·58<br>(8·17 – 9·03)	9·24<br>(8·81 – 9·69)	9·01<br>(8·6 – 9·41)	10·29<br>(9·78 – 10·79)	0·79<br>(0·61 – 0·95)	2 244 820<br>(2 135 045 – 2 355 315)	21·75
Belarus	13·53<br>(12·05 – 15·31)	12·0<br>(11·41 – 12·67)	11·95<br>(11·7 – 12·19)	12·76<br>(12·54 – 12·99)	13·18<br>(12·94 – 13·39)	12·66<br>(12·3 – 13·01)	-0·26<br>(-0·68 – 0·15)	122 488<br>(119 061 – 125 736)	-11·25
Estonia	8·0<br>(7·01 – 9·35)	6·52<br>(6·2 – 6·9)	7·36<br>(7·18 – 7·56)	7·75<br>(7·6 – 7·9)	7·57<br>(7·42 – 7·72)	7·6<br>(7·38 – 7·82)	-0·2<br>(-0·77 – 0·27)	10 320<br>(10 044 – 10 587)	-18·29
Latvia	6·77<br>(5·82 – 7·76)	4·91<br>(4·64 – 5·2)	5·35<br>(5·24 – 5·47)	6·3<br>(6·18 – 6·42)	7·0<br>(6·85 – 7·16)	7·02<br>(6·71 – 7·33)	0·14<br>(-0·33 – 0·62)	14 286<br>(13 705 – 14 868)	-20·56
Lithuania	8·31<br>(7·12 – 9·8)	7·13<br>(6·79 – 7·46)	7·2<br>(7·03 – 7·36)	7·88<br>(7·74 – 8·03)	7·96<br>(7·78 – 8·14)	9·29<br>(9·02 – 9·57)	0·43<br>(-0·19 – 0·98)	28 035<br>(27 254 – 28 859)	-8·99

Moldova	7·28 (6·16 – 8·38)	8·29 (7·85 – 8·74)	6·89 (6·74 – 7·03)	6·23 (6·12 – 6·34)	6·43 (6·3 – 6·55)	6·65 (6·44 – 6·86)	-0·35 (-0·85 – 0·22)	26 964 (26 123 – 27 827)	-14·66
Russia	8·88 (8·34 – 9·44)	8·1 (7·64 – 8·55)	9·61 (9·19 – 10·05)	10·23 (9·82 – 10·64)	9·81 (9·43 – 10·18)	11·61 (11·1 – 12·14)	1·03 (0·84 – 1·23)	1 755 031 (1 677 137 – 1 832 763)	35·28
Ukraine	6·09 (5·2 – 7·13)	6·57 (5·66 – 7·4)	5·29 (4·57 – 6·27)	6·07 (5·27 – 7·45)	6·17 (5·25 – 7·25)	6·19 (5·38 – 7·31)	0·07 (-0·17 – 0·33)	287 696 (250 635 – 342 033)	-8·8
High-income	8·63 (7·53 – 9·78)	8·59 (7·68 – 9·5)	8·02 (7·3 – 8·78)	7·78 (7·18 – 8·41)	7·68 (7·09 – 8·26)	7·97 (7·26 – 8·8)	-0·31 (-0·44 to - 0·11)	9 167 921 (8 403 482 – 10 011 034)	16·66
Australasia	8·53 (7·3 – 10·09)	11·16 (9·22 – 12·85)	7·31 (7·06 – 7·55)	7·59 (7·42 – 7·76)	7·62 (7·45 – 7·77)	8·22 (7·94 – 8·48)	-0·14 (-0·73 – 0·43)	240 993 (233 044 – 248 421)	38·45
Australia	8·59 (7·32 – 10·21)	11·71 (9·56 – 13·51)	7·32 (7·14 – 7·51)	7·43 (7·28 – 7·58)	7·55 (7·4 – 7·68)	8·3 (8·05 – 8·54)	-0·13 (-0·75 – 0·46)	204 734 (198 709 – 210 632)	39·97
New Zealand	8·23 (7·07 – 9·58)	8·46 (7·23 – 10·02)	7·25 (6·61 – 8·07)	8·38 (8·02 – 8·71)	7·99 (7·63 – 8·34)	7·77 (7·25 – 8·24)	-0·22 (-0·68 – 0·23)	36 259 (33 862 – 38 389)	30·42
High-income Asia-Pacific	17·36 (15·18 – 19·61)	17·41 (15·47 – 19·38)	15·8 (14·0 – 17·8)	15·4 (13·97 – 16·89)	15·26 (13·82 – 16·53)	15·46 (14·02 – 17·06)	-0·45 (-0·56 to - 0·28)	2 960 975 (2 664 263 – 3 216 790)	0·67
Brunei	8·35 (7·36 – 9·86)	8·67 (7·48 – 10·24)	7·87 (7·46 – 8·32)	9·1 (7·86 – 11·17)	9·29 (8·13 – 11·07)	9·48 (8·1 – 11·64)	0·49 (0·12 – 0·76)	3972 (3392 – 4991)	92·7
Japan	18·35 (16·03 – 20·71)	18·33 (16·2 – 20·57)	16·92 (14·93 – 18·97)	16·25 (14·33 – 18·36)	15·42 (13·48 – 17·25)	15·24 (13·26 – 17·47)	-0·71 (-0·84 to - 0·59)	2 058 990 (1 785 566 – 2 288 948)	-9·62
Singapore	12·88 (11·57 – 14·62)	13·18 (11·27 – 14·77)	13·62 (11·76 – 15·47)	13·81 (12·0 – 15·72)	14·11 (11·93 – 16·51)	14·36 (12·25 – 17·43)	0·42 (-0·03 – 0·77)	57 248 (48 863 – 69 706)	64·39
South Korea	14·91 (12·92 – 16·98)	15·33 (13·63 – 17·27)	13·06 (11·72 – 15·35)	13·56 (13·15 – 13·98)	15·15 (14·79 – 15·48)	16·27 (15·69 – 16·79)	0·33 (-0·12 – 0·85)	840 765 (811 979 – 867 526)	34·24
High-income North America	5·7 (4·98 – 6·58)	5·58 (4·89 – 6·43)	5·5 (4·85 – 6·23)	5·66 (5·03 – 6·37)	5·77 (5·14 – 6·49)	5·94 (5·25 – 6·71)	0·16 (-0·03 – 0·31)	2 306 572 (2 043 005 – 2 587 508)	43·11
Canada	9·41 (8·24 – 10·75)	9·66 (8·26 – 11·18)	8·25 (7·84 – 8·62)	8·7 (8·5 – 8·9)	8·53 (8·33 – 8·73)	8·6 (8·2 – 9·0)	-0·35 (-0·78 – 0·09)	337 164 (320 280 – 352 540)	29·39
Greenland	6·98 (6·02 – 8·05)	6·96 (5·87 – 8·21)	6·96 (6·02 – 8·21)	7·1 (6·16 – 8·31)	7·19 (6·19 – 8·32)	7·22 (6·26 – 8·45)	0·13 (-0·18 – 0·39)	361 (313 – 425)	10·41
United States	5·3 (4·59 – 6·13)	5·13 (4·5 – 5·89)	5·21 (4·53 – 5·99)	5·33 (4·66 – 6·12)	5·48 (4·77 – 6·27)	5·65 (4·94 – 6·49)	0·25 (0·06 – 0·39)	1 968 253 (1 712 250 – 2 237 653)	45·78
Southern Latin America	4·31 (3·81 – 4·79)	4·37 (3·92 – 4·85)	4·13 (3·72 – 4·56)	3·71 (3·5 – 3·94)	3·82 (3·61 – 4·08)	4·38 (3·93 – 4·95)	0·06 (-0·17 – 0·27)	287 249 (257 292 – 324 392)	38·8
Argentina	4·08 (3·47 – 4·73)	4·19 (3·61 – 4·82)	4·19 (3·66 – 4·77)	3·59 (3·34 – 3·91)	3·71 (3·46 – 4·05)	4·52 (3·89 – 5·31)	0·39 (0·05 – 0·7)	197 665 (170 214 – 232 432)	50·58
Chile	4·9 (4·67 – 5·14)	4·86 (4·72 – 4·99)	3·92 (3·8 – 4·04)	3·84 (3·76 – 3·91)	3·93 (3·83 – 4·04)	4·0 (3·91 – 4·09)	-0·78 (-0·92 to - 0·63)	73 361 (71 809 – 75 050)	17·33
Uruguay	4·23 (3·61 – 4·81)	4·28 (3·62 – 4·99)	4·41 (3·84 – 5·06)	4·44 (3·8 – 5·22)	4·51 (3·86 – 5·23)	4·61 (3·99 – 5·36)	0·33 (0·03 – 0·61)	16 209 (14 128 – 18 753)	23·24
Western Europe	7·42 (6·41 – 8·58)	7·4 (6·74 – 8·02)	7·21 (6·7 – 7·73)	6·9 (6·49 – 7·37)	6·75 (6·36 – 7·19)	7·33 (6·68 – 8·12)	-0·04 (-0·26 – 0·22)	3 372 132 (3 117 281 – 3 663 638)	15·3
Andorra	6·87 (6·02 – 8·01)	7·04 (6·07 – 8·16)	7·01 (5·96 – 8·36)	7·1 (6·06 – 8·46)	7·11 (6·08 – 8·43)	7·07 (6·18 – 8·11)	0·11 (-0·26 – 0·44)	603 (528 – 690)	70·83
Austria	8·07 (6·92 – 9·36)	6·44 (6·1 – 6·81)	6·96 (6·79 – 7·12)	7·58 (7·44 – 7·74)	7·54 (7·39 – 7·69)	7·51 (7·26 – 7·78)	-0·27 (-0·78 – 0·29)	71 220 (69 125 – 73 423)	11·77
Belgium	9·1 (7·86 – 10·6)	7·56 (7·21 – 7·91)	8·32 (8·14 – 8·49)	8·76 (8·58 – 8·92)	8·04 (7·78 – 8·32)	9·43 (8·47 – 10·74)	0·13 (-0·24 – 0·47)	112 672 (103 231 – 124 882)	19·96
Cyprus	5·11 (4·44 – 5·91)	5·21 (4·44 – 6·27)	5·2 (4·54 – 6·02)	5·56 (4·74 – 6·45)	5·55 (4·78 – 6·44)	5·61 (4·75 – 6·47)	0·36 (0·07 – 0·72)	5292 (4516 – 6084)	54·96
Denmark	5·47 (4·63 – 6·43)	4·18 (3·98 – 4·38)	4·61 (4·52 – 4·71)	5·02 (4·93 – 5·12)	5·27 (5·18 – 5·36)	5·24 (5·11 – 5·36)	-0·16 (-0·75 – 0·44)	32 599 (31 861 – 33 332)	11·49
Finland	5·52 (4·78 – 6·44)	4·22 (4·0 – 4·43)	4·93 (4·81 – 5·05)	5·23 (5·12 – 5·33)	5·17 (5·07 – 5·26)	4·97 (4·8 – 5·14)	-0·4 (-0·93 – 0·11)	29 798 (28 798 – 30 750)	5·41
France	8·16 (6·97 – 9·74)	6·54 (6·23 – 6·87)	7·58 (7·39 – 7·78)	8·19 (8·04 – 8·33)	7·36 (7·2 – 7·52)	7·53 (7·28 – 7·79)	-0·31 (-0·96 – 0·26)	528 113 (513 364 – 543 914)	11·17
Germany	9·14 (7·83 – 10·52)	10·72 (9·67 – 11·39)	9·8 (8·93 – 10·46)	8·04 (7·2 – 8·95)	7·78 (7·28 – 8·41)	9·68 (8·6 – 10·96)	0·22 (-0·09 – 0·54)	850 500 (773 673 – 936 425)	12·34
Greece	4·49 (3·86 – 5·33)	4·07 (3·83 – 4·3)	3·96 (3·88 – 4·05)	4·05 (3·92 – 4·18)	4·39 (3·99 – 4·83)	4·82 (4·08 – 5·83)	0·27 (-0·03 – 0·59)	56 668 (48 650 – 66 797)	20·95
Iceland	7·57 (6·47 – 9·09)	5·4 (5·1 – 5·79)	6·47 (6·28 – 6·65)	7·32 (7·17 – 7·48)	7·14 (6·98 – 7·3)	6·86 (6·62 – 7·1)	-0·38 (-1·01 – 0·13)	2365 (2283 – 2447)	22·78
Ireland	6·56 (5·51 – 7·67)	6·71 (5·79 – 7·8)	6·07 (5·39 – 7·17)	5·16 (4·87 – 5·47)	5·63 (5·38 – 5·85)	9·02 (8·48 – 9·51)	1·22 (0·67 – 1·72)	43 470 (40 961 – 45 746)	87·83
Israel	8·77 (7·52 – 10·29)	8·91 (7·63 – 10·12)	9·77 (9·19 – 10·3)	9·23 (8·27 – 10·34)	7·37 (6·91 – 7·88)	8·52 (7·43 – 9·96)	-0·11 (-0·59 – 0·29)	70 402 (61 775 – 81 819)	81·02
Italy	8·87 (7·63 – 10·15)	9·02 (7·82 – 10·15)	8·11 (7·16 – 9·27)	8·6 (7·8 – 9·58)	9·06 (8·13 – 10·09)	8·94 (7·95 – 10·44)	0·03 (-0·3 – 0·34)	585 977 (529 305 – 662 681)	12·35
Luxembourg	8·02 (6·95 – 9·19)	8·04 (6·94 – 9·16)	8·12 (7·52 – 8·66)	7·2 (7·01 – 7·38)	7·24 (7·1 – 7·39)	7·31 (7·07 – 7·55)	-0·36 (-0·82 – 0·16)	4481 (4338 – 4628)	42·13
Malta	11·93 (10·27 – 14·09)	12·12 (10·49 – 13·75)	11·93 (10·83 – 13·28)	12·59 (10·86 – 14·58)	12·57 (10·76 – 14·53)	12·86 (11·08 – 14·77)	0·29 (-0·11 – 0·61)	5790 (4934 – 6641)	35·91
Netherlands	5·36 (4·6 – 6·5)	5·47 (4·64 – 6·4)	5·12 (4·8 – 5·43)	4·77 (4·58 – 4·97)	4·85 (4·64 – 5·06)	5·06 (4·64 – 5·58)	-0·22 (-0·69 – 0·19)	92 091 (85 339 – 99 650)	12·69
Norway	5·24 (4·37 – 6·24)	5·34 (4·58 – 6·34)	5·4 (4·61 – 6·42)	4·44 (4·28 – 4·6)	4·9 (4·8 – 5·01)	4·91 (4·75 – 5·08)	-0·25 (-0·85 – 0·38)	27 348 (26 448 – 28 256)	18·77
Portugal	4·27 (3·66 – 5·04)	3·33 (3·13 – 3·52)	3·79 (3·62 – 3·98)	3·64 (3·57 – 3·72)	4·22 (4·08 – 4·35)	4·85 (4·45 – 5·2)	0·49 (0·03 – 0·92)	57 470 (52 897 – 61 515)	31

Spain	7·99 (6·89 – 9·2)	8·09 (6·95 – 9·37)	8·23 (7·52 – 8·92)	6·98 (6·59 – 7·39)	6·7 (6·3 – 7·14)	7·61 (6·65 – 8·68)	-0·19 (-0·55 – 0·17)	364 830 (326 079 – 410 417)	14·48
Sweden	4·6 (4·0 – 5·33)	4·64 (3·99 – 5·41)	3·94 (3·4 – 4·62)	3·57 (3·06 – 4·08)	3·96 (3·32 – 4·64)	3·96 (3·36 – 4·69)	-0·58 (-0·83 to -0·29)	41 650 (35 544 – 48 616)	0·18
Switzerland	5·56 (4·74 – 6·58)	5·58 (4·77 – 6·42)	4·47 (4·16 – 4·91)	4·7 (4·5 – 4·88)	5·09 (4·87 – 5·29)	5·3 (4·93 – 5·69)	-0·18 (-0·65 – 0·3)	48 220 (45 373 – 51 332)	24·67
United Kingdom	4·8 (4·17 – 5·57)	4·83 (4·18 – 5·61)	4·58 (3·96 – 5·29)	4·9 (4·24 – 5·69)	4·89 (4·22 – 5·69)	4·85 (4·2 – 5·62)	0·04 (-0·0 – 0·1)	337 223 (292 471 – 384 746)	18·99
Latin America and Caribbean	4·76 (4·17 – 5·47)	4·71 (4·14 – 5·37)	4·68 (4·18 – 5·17)	4·69 (4·25 – 5·13)	4·63 (4·25 – 5·02)	4·84 (4·34 – 5·37)	0·06 (-0·11 – 0·22)	2 716 242 (2 434 008 – 3 032 214)	52·6
Andean Latin America	4·81 (4·35 – 5·3)	4·51 (4·14 – 4·91)	4·5 (4·17 – 4·83)	5·31 (4·96 – 5·71)	5·92 (5·55 – 6·29)	5·88 (5·04 – 7·12)	0·78 (0·44 – 1·21)	341 584 (290 621 – 416 754)	96·56
Bolivia	3·96 (3·67 – 4·27)	3·98 (3·72 – 4·24)	3·99 (3·76 – 4·22)	4·36 (4·14 – 4·58)	4·8 (4·35 – 5·29)	5·57 (4·75 – 6·81)	1·31 (0·82 – 2·0)	59 337 (49 875 – 73 913)	139·69
Ecuador	5·26 (4·46 – 6·23)	5·37 (4·53 – 6·39)	5·51 (4·7 – 6·44)	5·6 (4·7 – 6·81)	5·76 (4·9 – 6·72)	5·91 (5·0 – 7·02)	0·45 (0·03 – 0·83)	95 235 (79 805 – 114 457)	85·82
Peru	4·85 (4·42 – 5·31)	4·27 (3·99 – 4·54)	4·18 (4·0 – 4·37)	5·5 (5·31 – 5·7)	6·39 (6·2 – 6·58)	5·97 (5·11 – 7·28)	0·8 (0·37 – 1·35)	187 011 (159 602 – 230 504)	91·27
Caribbean	3·09 (2·67 – 3·54)	3·14 (2·72 – 3·58)	3·2 (2·77 – 3·64)	3·26 (2·82 – 3·74)	3·3 (2·84 – 3·78)	3·37 (2·89 – 3·88)	0·33 (0·22 – 0·42)	152 895 (131 201 – 175 715)	53·13
Antigua and Barbuda	3·49 (2·98 – 4·05)	3·55 (3·02 – 4·1)	3·63 (3·1 – 4·22)	3·64 (3·14 – 4·24)	3·73 (3·17 – 4·34)	3·78 (3·22 – 4·38)	0·31 (0·08 – 0·54)	338 (285 – 394)	71·67
The Bahamas	4·29 (3·62 – 5·02)	4·29 (3·65 – 5·01)	4·09 (3·73 – 4·46)	4·46 (3·78 – 5·17)	4·48 (3·8 – 5·2)	4·49 (3·83 – 5·26)	0·18 (-0·05 – 0·38)	1775 (1509 – 2075)	87·05
Barbados	2·14 (1·82 – 2·44)	2·17 (1·85 – 2·5)	2·2 (1·92 – 2·49)	2·23 (1·9 – 2·57)	2·24 (1·9 – 2·6)	2·26 (1·93 – 2·61)	0·21 (0·01 – 0·42)	706 (602 – 813)	32·71
Belize	2·87 (2·43 – 3·36)	3·05 (2·6 – 3·52)	3·13 (2·66 – 3·64)	3·2 (2·73 – 3·71)	3·25 (2·78 – 3·8)	3·33 (2·84 – 3·84)	0·57 (0·35 – 0·79)	1089 (915 – 1284)	125·02
Bermuda	3·63 (3·1 – 4·19)	3·69 (3·14 – 4·24)	3·74 (3·2 – 4·36)	3·81 (3·23 – 4·4)	3·86 (3·3 – 4·46)	3·91 (3·34 – 4·53)	0·28 (0·09 – 0·49)	270 (230 – 315)	55·22
Cuba	2·94 (2·51 – 3·38)	2·96 (2·55 – 3·41)	2·98 (2·59 – 3·4)	3·05 (2·61 – 3·51)	3·08 (2·61 – 3·57)	3·17 (2·7 – 3·65)	0·29 (0·08 – 0·52)	40 426 (34 600 – 46 347)	36·97
Dominica	5·06 (4·31 – 5·91)	5·22 (4·42 – 6·15)	5·26 (4·54 – 6·12)	5·45 (4·63 – 6·43)	5·57 (4·73 – 6·52)	5·64 (4·79 – 6·54)	0·41 (0·22 – 0·6)	412 (348 – 481)	30·46
Dominican Republic	3·06 (2·6 – 3·57)	3·13 (2·66 – 3·59)	3·23 (2·73 – 3·74)	3·31 (2·83 – 3·81)	3·4 (2·89 – 3·96)	3·52 (3·02 – 4·08)	0·55 (0·34 – 0·78)	34 633 (29 530 – 40 524)	84·98
Grenada	3·0 (2·53 – 3·48)	3·12 (2·67 – 3·59)	3·23 (2·77 – 3·73)	3·36 (2·88 – 3·85)	3·45 (2·94 – 3·99)	3·54 (3·03 – 4·13)	0·64 (0·44 – 0·86)	351 (299 – 412)	29·94
Guyana	4·32 (3·9 – 4·77)	4·38 (3·93 – 4·82)	4·82 (4·13 – 5·6)	4·84 (4·13 – 5·67)	4·95 (4·22 – 5·78)	5·11 (4·36 – 5·91)	0·64 (0·33 – 0·99)	3611 (3009 – 4203)	29·19
Haiti	2·45 (2·09 – 2·83)	2·52 (2·14 – 2·89)	2·55 (2·19 – 2·92)	2·6 (2·23 – 3·01)	2·68 (2·28 – 3·08)	2·75 (2·34 – 3·16)	0·45 (0·24 – 0·64)	27 119 (22 792 – 31 526)	77·69
Jamaica	1·8 (1·69 – 1·9)	1·72 (1·64 – 1·8)	1·87 (1·75 – 2·0)	2·09 (1·78 – 2·4)	2·12 (1·79 – 2·43)	2·14 (1·83 – 2·47)	0·67 (0·16 – 1·11)	6026 (5154 – 6945)	52·07
Puerto Rico	5·22 (4·46 – 6·08)	5·37 (4·57 – 6·23)	5·49 (4·66 – 6·41)	5·59 (4·87 – 6·41)	5·67 (4·9 – 6·52)	5·68 (4·84 – 6·6)	0·33 (0·08 – 0·57)	22 677 (19 425 – 26 331)	26·2
Saint Lucia	3·79 (3·26 – 4·39)	3·92 (3·33 – 4·52)	3·78 (3·46 – 4·09)	3·88 (3·56 – 4·2)	3·89 (3·57 – 4·2)	4·3 (3·67 – 4·99)	0·48 (0·2 – 0·7)	773 (657 – 899)	57·02
Saint Vincent and the Grenadines	3·03 (2·61 – 3·49)	3·13 (2·67 – 3·63)	3·22 (2·77 – 3·72)	3·3 (2·83 – 3·82)	3·37 (2·88 – 3·95)	3·43 (2·94 – 3·99)	0·48 (0·26 – 0·7)	366 (313 – 429)	23·69
Suriname	3·17 (2·71 – 3·63)	3·2 (2·72 – 3·71)	3·24 (2·78 – 3·76)	3·32 (2·82 – 3·87)	3·41 (2·9 – 3·92)	3·51 (3·01 – 4·05)	0·4 (0·18 – 0·61)	1831 (1557 – 2117)	58·55
Trinidad and Tobago	2·57 (2·18 – 2·98)	2·61 (2·23 – 3·02)	2·53 (2·22 – 2·83)	2·72 (2·32 – 3·15)	2·78 (2·37 – 3·16)	2·81 (2·4 – 3·26)	0·35 (0·15 – 0·53)	3825 (3270 – 4466)	34·37
Virgin Islands, U·S·	3·79 (3·24 – 4·38)	3·88 (3·29 – 4·53)	3·96 (3·4 – 4·58)	4·02 (3·41 – 4·64)	4·09 (3·49 – 4·76)	4·13 (3·52 – 4·82)	0·34 (0·09 – 0·55)	505 (427 – 595)	··
Central Latin America	4·28 (3·68 – 4·98)	4·33 (3·69 – 5·07)	4·53 (3·98 – 5·1)	4·36 (3·95 – 4·74)	4·24 (3·79 – 4·72)	4·6 (3·99 – 5·27)	0·28 (0·14 – 0·4)	1 128 647 (970 071 – 1 298 259)	68·75
Colombia	5·99 (5·2 – 6·9)	6·16 (5·24 – 7·31)	7·14 (6·32 – 7·78)	6·33 (5·91 – 6·79)	5·74 (5·07 – 6·63)	6·81 (5·85 – 7·91)	0·49 (0·22 – 0·79)	320 362 (274 546 – 373 583)	71·46
Costa Rica	2·86 (2·44 – 3·31)	2·94 (2·51 – 3·37)	3·22 (2·74 – 3·77)	2·77 (2·39 – 3·16)	3·16 (2·69 – 3·68)	3·24 (2·75 – 3·77)	0·48 (0·25 – 0·72)	15 387 (13 021 – 17 974)	85·89
El Salvador	1·86 (1·58 – 2·14)	1·92 (1·64 – 2·23)	2·0 (1·72 – 2·29)	2·06 (1·75 – 2·36)	2·0 (1·85 – 2·15)	2·07 (1·91 – 2·22)	0·42 (0·04 – 0·87)	12 372 (11 373 – 13 322)	36·57
Guatemala	4·24 (3·61 – 4·88)	4·39 (3·74 – 5·2)	4·5 (3·83 – 5·18)	4·5 (3·93 – 5·07)	4·68 (4·03 – 5·31)	4·99 (4·24 – 5·86)	0·63 (0·35 – 0·92)	75 793 (62 939 – 91 821)	111·1
Honduras	3·76 (3·18 – 4·38)	3·9 (3·33 – 4·65)	4·04 (3·4 – 4·75)	4·16 (3·5 – 4·92)	4·27 (3·57 – 5·01)	4·37 (3·68 – 5·09)	0·57 (0·32 – 0·83)	33 659 (27 975 – 39 419)	97·04
Mexico	3·88 (3·28 – 4·59)	3·92 (3·29 – 4·65)	3·81 (3·23 – 4·48)	3·69 (3·33 – 4·06)	3·67 (3·31 – 4·04)	3·8 (3·29 – 4·36)	-0·08 (-0·28 – 0·09)	476 921 (411 679 – 550 204)	52·65
Nicaragua	5·27 (4·46 – 6·14)	4·79 (4·3 – 5·48)	6·65 (5·95 – 7·35)	6·47 (5·59 – 7·35)	5·78 (5·32 – 6·25)	6·91 (5·86 – 7·91)	1·04 (0·73 – 1·37)	39 671 (33 230 – 46 376)	112·45
Panama	9·01 (7·76 – 10·6)	7·82 (7·37 – 8·43)	7·94 (7·61 – 8·3)	8·56 (8·16 – 8·96)	9·03 (8·44 – 9·67)	10·21 (8·77 – 11·73)	0·48 (0·23 – 0·77)	39 418 (33 751 – 45 502)	93·73
Venezuela	3·32 (2·81 – 3·89)	3·42 (2·92 – 3·94)	3·36 (2·91 – 3·8)	3·67 (3·12 – 4·25)	3·7 (3·12 – 4·29)	3·82 (3·24 – 4·45)	0·54 (0·33 – 0·77)	115 064 (96 988 – 135 135)	91·1
Tropical Latin America	5·64 (4·93 – 6·53)	5·51 (4·88 – 6·25)	5·24 (4·71 – 5·74)	5·23 (4·7 – 5·78)	5·0 (4·69 – 5·35)	5·12 (4·8 – 5·45)	-0·37 (-0·81 – 0·02)	1 093 117 (1 024 805 – 1 166 501)	30·51
Brazil	5·7 (4·97 – 6·59)	5·58 (4·94 – 6·34)	5·29 (4·76 – 5·8)	5·28 (4·75 – 5·85)	5·05 (4·74 – 5·41)	5·16 (4·84 – 5·48)	-0·38 (-0·84 – 0·01)	1 067 171 (1 001 689 – 1 138 268)	29·58
Paraguay	3·49 (3·0 – 4·14)	2·89 (2·63 – 3·18)	3·43 (3·15 – 3·74)	3·25 (3·04 – 3·48)	3·16 (2·98 – 3·34)	4·03 (3·44 – 4·71)	0·55 (0·29 – 0·84)	25 945 (22 035 – 30 585)	85·43

North Africa and Middle East	4·88 (4·21 – 5·64)	5·04 (4·35 – 5·84)	4·69 (4·15 – 5·28)	5·08 (4·5 – 5·8)	5·54 (4·92 – 6·22)	6·06 (5·43 – 6·88)	0·84 (0·65 – 1·0)	3 286 256 (2 916 614 – 3 798 035)	119·58
Afghanistan	5·55 (4·73 – 6·46)	5·57 (4·7 – 6·62)	5·63 (4·8 – 6·76)	5·06 (4·52 – 5·75)	6·42 (5·91 – 6·87)	6·13 (5·23 – 7·26)	0·38 (0·09 – 0·69)	186 654 (155 891 – 227 810)	207·78
Algeria	3·58 (2·98 – 4·25)	3·55 (3·09 – 4·17)	3·74 (3·17 – 4·37)	3·81 (3·26 – 4·52)	3·91 (3·33 – 4·6)	4·2 (3·6 – 4·94)	0·62 (0·27 – 0·99)	163 861 (138 980 – 193 457)	94·51
Bahrain	7·01 (5·97 – 8·2)	7·6 (7·09 – 8·12)	6·79 (5·86 – 8·09)	7·69 (6·62 – 8·99)	7·85 (6·72 – 9·34)	8·01 (6·83 – 9·55)	0·51 (0·23 – 0·82)	10 451 (8663 – 13 018)	226·91
Egypt	6·94 (5·93 – 8·1)	7·21 (6·14 – 8·36)	6·93 (6·17 – 7·89)	7·56 (6·55 – 8·83)	7·71 (6·72 – 8·81)	8·14 (6·99 – 9·79)	0·62 (0·25 – 0·97)	712 227 (605 804 – 877 997)	96·49
Iran	4·85 (4·13 – 5·76)	5·25 (4·51 – 6·1)	5·51 (4·66 – 6·58)	5·62 (4·81 – 6·73)	5·74 (4·93 – 6·74)	5·84 (5·11 – 6·76)	0·71 (0·39 – 1·02)	457 505 (394 519 – 536 110)	81·68
Iraq	2·16 (1·86 – 2·51)	2·24 (1·9 – 2·59)	2·04 (1·79 – 2·3)	2·18 (1·99 – 2·37)	2·28 (2·03 – 2·51)	2·33 (1·99 – 2·73)	0·29 (0·04 – 0·51)	84 519 (71 009 – 100 032)	141·97
Jordan	3·4 (2·88 – 4·04)	3·6 (3·04 – 4·33)	3·56 (3·16 – 4·01)	3·84 (3·23 – 4·53)	3·95 (3·38 – 4·65)	4·08 (3·48 – 4·82)	0·7 (0·41 – 1·01)	29 442 (24 705 – 35 195)	183·69
Kuwait	5·31 (4·54 – 6·41)	5·25 (4·47 – 6·37)	5·56 (4·72 – 6·5)	5·61 (4·83 – 6·62)	5·87 (4·97 – 6·9)	6·11 (5·22 – 7·29)	0·54 (0·25 – 0·84)	22 558 (19 011 – 27 657)	124·24
Lebanon	3·55 (3·03 – 4·14)	3·65 (3·11 – 4·25)	3·48 (3·11 – 3·85)	3·89 (3·33 – 4·67)	4·0 (3·39 – 4·63)	4·07 (3·45 – 4·77)	0·53 (0·24 – 0·79)	23 309 (19 682 – 27 735)	150·27
Libya	5·15 (4·37 – 6·14)	5·32 (4·57 – 6·26)	5·43 (4·68 – 6·4)	5·65 (4·77 – 6·68)	5·92 (5·08 – 6·89)	6·03 (5·23 – 7·02)	0·61 (0·28 – 0·93)	35 538 (30 645 – 42 287)	75·9
Morocco	2·66 (2·38 – 2·96)	3·16 (2·68 – 3·75)	3·36 (2·94 – 3·78)	3·43 (2·91 – 4·04)	3·54 (3·03 – 4·15)	3·66 (3·08 – 4·39)	1·23 (0·84 – 1·69)	120 089 (99 811 – 146 430)	98·48
Oman	4·79 (4·15 – 5·6)	4·74 (4·42 – 5·03)	5·42 (4·73 – 6·19)	6·19 (5·34 – 7·39)	6·71 (5·72 – 7·7)	7·15 (6·2 – 8·4)	1·54 (1·26 – 1·89)	30 916 (25 915 – 38 000)	285·18
Palestine	5·18 (4·36 – 6·01)	5·38 (4·64 – 6·36)	5·41 (4·75 – 6·1)	5·43 (4·78 – 6·34)	5·22 (4·44 – 6·05)	5·35 (4·57 – 6·2)	0·13 (-0·22 – 0·44)	25 291 (21 148 – 30 092)	161·26
Qatar	9·12 (7·88 – 10·57)	8·49 (7·9 – 9·12)	8·74 (8·18 – 9·37)	10·05 (8·67 – 11·74)	10·31 (8·76 – 12·01)	10·51 (9·11 – 12·21)	0·55 (0·19 – 0·89)	22 037 (18 192 – 26 838)	452·25
Saudi Arabia	6·4 (5·54 – 7·46)	6·14 (5·27 – 7·14)	6·66 (5·71 – 7·72)	6·9 (5·95 – 7·97)	7·06 (6·08 – 8·16)	7·25 (6·33 – 8·23)	0·48 (0·29 – 0·63)	216 427 (187 657 – 250 264)	126·11
Sudan	4·06 (3·45 – 4·81)	4·11 (3·5 – 4·89)	4·18 (3·56 – 5·04)	4·37 (3·75 – 5·27)	4·55 (3·85 – 5·41)	4·77 (4·08 – 5·67)	0·62 (0·22 – 0·92)	173 011 (145 714 – 212 388)	142·65
Syria	3·89 (3·28 – 4·56)	4·1 (3·5 – 4·78)	4·05 (3·62 – 4·58)	4·47 (3·83 – 5·23)	4·69 (3·98 – 5·46)	4·83 (4·16 – 5·66)	0·83 (0·49 – 1·13)	81 921 (69 881 – 97 794)	85·5
Tunisia	4·69 (3·97 – 5·65)	4·9 (4·2 – 5·97)	5·1 (4·3 – 5·97)	5·26 (4·52 – 6·32)	5·39 (4·61 – 6·35)	5·54 (4·77 – 6·57)	0·64 (0·28 – 0·91)	61 770 (53 049 – 73 614)	69·89
Turkey	5·54 (4·76 – 6·46)	5·66 (4·83 – 6·84)	3·34 (3·27 – 3·43)	4·63 (4·39 – 4·88)	6·48 (6·13 – 6·83)	8·95 (8·63 – 9·25)	1·85 (1·31 – 2·37)	699 558 (673 022 – 724 144)	145·43
United Arab Emirates	5·41 (4·63 – 6·52)	5·57 (5·13 – 6·03)	5·6 (4·86 – 6·64)	6·19 (5·3 – 7·32)	6·27 (5·29 – 7·4)	6·48 (5·43 – 7·89)	0·69 (0·35 – 1·02)	57 991 (46 637 – 74 231)	530·74
Yemen	2·31 (1·98 – 2·75)	2·39 (2·05 – 2·8)	2·52 (2·15 – 2·96)	2·32 (2·05 – 2·63)	2·7 (2·46 – 2·95)	2·87 (2·62 – 3·12)	0·83 (0·4 – 1·22)	67 826 (60 315 – 75 344)	178·18
South Asia	3·74 (3·18 – 4·36)	3·36 (2·99 – 3·74)	3·83 (3·22 – 4·55)	4·06 (3·59 – 4·5)	3·82 (3·29 – 4·39)	4·04 (3·42 – 4·69)	0·3 (0·18 – 0·42)	6 017 076 (5 057 230 – 7 056 139)	77·97
Bangladesh	3·5 (2·97 – 4·11)	3·66 (3·14 – 4·24)	3·75 (3·19 – 4·33)	3·84 (3·3 – 4·45)	3·99 (3·41 – 4·62)	4·13 (3·51 – 4·79)	0·64 (0·46 – 0·82)	569 520 (479 917 – 668 141)	99·06
Bhutan	3·88 (3·32 – 4·5)	4·1 (3·49 – 4·76)	3·83 (3·47 – 4·21)	4·03 (3·65 – 4·42)	4·77 (4·07 – 5·59)	4·94 (4·23 – 5·73)	0·93 (0·74 – 1·13)	3346 (2838 – 3917)	103·76
India	3·64 (3·04 – 4·31)	3·16 (2·84 – 3·49)	3·72 (3·1 – 4·46)	4·1 (3·62 – 4·56)	3·77 (3·2 – 4·38)	3·85 (3·23 – 4·49)	0·21 (0·07 – 0·36)	4 493 492 (3 766 824 – 5 296 350)	72·47
Nepal	2·16 (1·85 – 2·51)	2·28 (1·94 – 2·63)	2·22 (1·95 – 2·49)	2·31 (2·02 – 2·61)	2·52 (2·16 – 2·91)	2·58 (2·2 – 2·98)	0·68 (0·47 – 0·89)	66 347 (56 006 – 76 531)	103·2
Pakistan	5·09 (4·71 – 5·4)	4·89 (4·21 – 5·62)	5·08 (4·31 – 5·93)	4·17 (3·85 – 4·51)	4·27 (3·92 – 4·59)	5·6 (4·77 – 6·52)	0·36 (-0·09 – 0·84)	884 371 (747 031 – 1 032 304)	94·24
Southeast Asia, East Asia, and Oceania	3·57 (3·24 – 3·91)	2·66 (2·48 – 2·85)	2·81 (2·62 – 3·0)	3·18 (2·95 – 3·4)	3·67 (3·39 – 3·95)	5·12 (4·7 – 5·56)	1·39 (1·31 – 1·46)	10 736 942 (9 832 370 – 11 689 204)	100·13
East Asia	3·54 (3·22 – 3·86)	2·26 (2·14 – 2·38)	2·45 (2·31 – 2·59)	2·81 (2·62 – 3·0)	3·38 (3·08 – 3·67)	5·46 (5·12 – 5·81)	1·67 (1·51 – 1·82)	7 968 349 (7 452 086 – 8 487 849)	105·91
China	3·21 (2·94 – 3·49)	1·91 (1·8 – 2·01)	2·12 (1·99 – 2·24)	2·49 (2·32 – 2·66)	3·01 (2·76 – 3·25)	5·17 (4·87 – 5·47)	1·83 (1·66 – 1·99)	7 277 644 (6 829 769 – 7 709 671)	114·41
North Korea	7·56 (6·43 – 8·77)	7·48 (6·41 – 8·66)	7·24 (6·2 – 8·3)	7·19 (6·12 – 8·3)	7·19 (6·17 – 8·37)	7·24 (6·17 – 8·51)	-0·16 (-0·38 – 0·05)	192 421 (163 680 – 225 942)	29·97
Taiwan	17·31 (14·86 – 19·93)	16·14 (15·63 – 16·65)	15·47 (15·1 – 15·84)	15·42 (14·93 – 15·94)	19·23 (16·55 – 22·09)	19·61 (17·04 – 22·44)	0·48 (0·17 – 0·77)	498 284 (428 374 – 575 081)	52·17
Oceania	5·28 (4·52 – 6·34)	5·01 (4·49 – 5·62)	5·44 (4·69 – 6·44)	5·54 (4·74 – 6·57)	5·66 (4·85 – 6·65)	5·77 (4·94 – 7·03)	0·34 (0·15 – 0·54)	60 363 (50 770 – 75 864)	88·63
American Samoa	6·46 (5·53 – 7·69)	6·72 (5·76 – 7·9)	6·72 (5·67 – 8·2)	6·86 (5·89 – 8·0)	7·05 (5·97 – 8·54)	7·06 (6·09 – 8·24)	0·34 (0·1 – 0·58)	540 (455 – 647)	..
Federated States of Micronesia	5·58 (4·77 – 6·55)	5·92 (5·03 – 7·34)	5·99 (5·12 – 7·36)	6·23 (5·32 – 7·39)	6·39 (5·42 – 7·67)	6·38 (5·51 – 7·33)	0·52 (0·21 – 0·83)	605 (511 – 712)	19·61
Fiji	6·39 (5·42 – 7·77)	6·74 (5·71 – 8·36)	6·67 (5·78 – 7·74)	6·83 (5·86 – 8·0)	7·01 (6·05 – 8·13)	7·12 (6·07 – 8·71)	0·42 (0·13 – 0·68)	5933 (4984 – 7408)	40·49
Guam	7·23 (6·16 – 8·42)	7·61 (6·4 – 9·39)	7·42 (6·34 – 8·76)	7·53 (6·48 – 8·71)	7·65 (6·52 – 9·04)	7·61 (6·58 – 8·93)	0·2 (-0·06 – 0·49)	1325 (1144 – 1556)	..
Kiribati	4·99 (4·24 – 5·98)	5·2 (4·42 – 6·23)	5·18 (4·38 – 6·41)	5·36 (4·49 – 6·4)	5·53 (4·65 – 6·85)	5·55 (4·69 – 6·49)	0·41 (0·13 – 0·72)	590 (492 – 704)	68·86
Marshall Islands	5·64 (4·86 – 6·64)	5·85 (5·0 – 6·78)	5·81 (4·9 – 6·82)	6·0 (5·12 – 7·27)	6·11 (5·2 – 7·28)	6·23 (5·31 – 7·51)	0·38 (0·1 – 0·69)	432 (360 – 538)	86·39

Northern Mariana Islands	6·94 (5·94 – 8·26)	7·33 (6·35 – 8·74)	7·3 (6·26 – 8·59)	7·52 (6·42 – 8·8)	7·57 (6·51 – 9·09)	7·5 (6·34 – 9·0)	0·3 (0·01 – 0·63)	878 (724 – 1103)	..
Papua New Guinea	4·96 (4·21 – 6·04)	4·43 (4·06 – 4·89)	5·11 (4·35 – 6·08)	5·21 (4·41 – 6·22)	5·33 (4·52 – 6·31)	5·47 (4·64 – 6·76)	0·38 (0·11 – 0·66)	39 943 (33 442 – 51 069)	109·58
Samoa	5·61 (4·77 – 6·68)	5·88 (4·97 – 6·99)	5·87 (5·03 – 6·92)	5·95 (5·03 – 6·88)	6·05 (5·13 – 6·97)	6·17 (5·21 – 7·36)	0·37 (0·07 – 0·7)	1166 (971 – 1408)	34·39
Solomon Islands	5·06 (4·34 – 5·98)	5·29 (4·52 – 6·24)	5·26 (4·49 – 6·26)	5·3 (4·48 – 6·3)	5·46 (4·63 – 6·44)	5·6 (4·76 – 6·5)	0·39 (0·09 – 0·74)	3098 (2564 – 3673)	115·47
Tonga	5·67 (4·83 – 6·86)	5·87 (4·98 – 6·99)	5·87 (4·98 – 7·2)	6·03 (5·11 – 7·48)	6·17 (5·2 – 7·27)	6·24 (5·35 – 7·46)	0·37 (0·07 – 0·61)	638 (542 – 772)	23·51
Vanuatu	5·14 (4·36 – 6·21)	5·35 (4·53 – 6·36)	5·32 (4·54 – 6·36)	5·51 (4·63 – 6·84)	5·64 (4·81 – 6·64)	5·72 (4·86 – 6·87)	0·41 (0·14 – 0·69)	1483 (1242 – 1816)	105·22
Southeast Asia	3·66 (3·27 – 4·08)	3·7 (3·34 – 4·1)	3·63 (3·28 – 4·01)	3·98 (3·67 – 4·32)	4·27 (4·02 – 4·51)	4·35 (3·68 – 5·12)	0·66 (0·42 – 0·9)	2 708 230 (2 282 862 – 3 209 352)	85·1
Cambodia	4·5 (3·83 – 5·2)	4·59 (3·89 – 5·33)	3·85 (3·58 – 4·16)	4·23 (4·05 – 4·41)	4·61 (4·42 – 4·79)	4·38 (3·97 – 4·9)	-0·1 (-0·46 – 0·26)	62 696 (56 449 – 70 614)	88·12
Indonesia	3·56 (3·34 – 3·77)	3·44 (3·33 – 3·55)	3·09 (3·0 – 3·19)	3·87 (3·73 – 4·01)	4·52 (4·36 – 4·67)	4·16 (3·43 – 5·06)	0·6 (-0·0 – 1·25)	1 011 617 (827 894 – 1 250 320)	87·12
Laos	3·51 (2·98 – 4·12)	3·64 (3·12 – 4·23)	3·74 (3·18 – 4·34)	3·92 (3·36 – 4·58)	4·03 (3·45 – 4·64)	4·18 (3·57 – 4·91)	0·67 (0·45 – 0·86)	26 628 (22 460 – 31 447)	108·37
Malaysia	7·72 (6·58 – 9·08)	7·94 (6·78 – 9·24)	8·18 (7·02 – 9·43)	8·62 (7·38 – 10·0)	7·99 (7·4 – 8·65)	9·13 (7·76 – 10·62)	0·64 (0·44 – 0·84)	259 560 (219 501 – 304 103)	111·38
Maldives	3·9 (3·31 – 4·55)	4·08 (3·45 – 4·74)	4·23 (3·58 – 4·9)	4·47 (3·76 – 5·23)	4·65 (3·92 – 5·41)	4·82 (4·09 – 5·6)	0·81 (0·64 – 1·01)	1554 (1308 – 1840)	132·79
Mauritius	4·54 (3·83 – 5·27)	4·7 (4·01 – 5·48)	4·77 (4·04 – 5·53)	4·93 (4·18 – 5·73)	5·02 (4·3 – 5·85)	5·1 (4·34 – 5·96)	0·45 (0·24 – 0·65)	6631 (5616 – 7780)	56·31
Myanmar	3·66 (3·1 – 4·24)	3·76 (3·18 – 4·36)	3·83 (3·26 – 4·45)	4·05 (3·42 – 4·75)	4·28 (3·62 – 4·94)	4·46 (3·8 – 5·19)	0·76 (0·59 – 0·94)	225 165 (189 459 – 264 997)	69·5
Philippines	5·19 (4·44 – 6·01)	5·28 (4·52 – 6·2)	5·36 (4·54 – 6·2)	5·53 (4·73 – 6·4)	5·14 (4·76 – 5·54)	5·74 (4·87 – 6·63)	0·39 (0·18 – 0·63)	531 660 (447 361 – 620 236)	94·92
Sri Lanka	4·17 (3·54 – 4·83)	4·35 (3·71 – 5·07)	4·42 (3·77 – 5·14)	4·58 (3·88 – 5·29)	4·71 (4·03 – 5·42)	4·91 (4·17 – 5·73)	0·63 (0·43 – 0·85)	102 564 (86 441 – 119 754)	57·87
Seychelles	3·75 (3·18 – 4·35)	4·25 (3·64 – 4·92)	3·85 (3·29 – 4·48)	4·25 (3·62 – 4·91)	4·24 (3·66 – 4·87)	4·16 (3·6 – 4·71)	0·4 (0·05 – 0·66)	398 (343 – 453)	66·09
Thailand	2·37 (2·02 – 2·73)	2·47 (2·09 – 2·85)	2·64 (2·26 – 2·98)	2·41 (2·17 – 2·62)	2·77 (2·51 – 2·98)	2·75 (2·34 – 3·19)	0·57 (0·38 – 0·75)	195 444 (165 287 – 228 016)	63·85
Timor-Leste	4·69 (4·01 – 5·49)	4·85 (4·13 – 5·69)	4·48 (3·95 – 5·08)	5·1 (4·66 – 5·53)	5·3 (4·81 – 5·78)	5·58 (4·73 – 6·54)	0·67 (0·47 – 0·88)	5785 (4860 – 6959)	101·39
Vietnam	2·57 (2·19 – 2·95)	2·68 (2·26 – 3·1)	2·78 (2·38 – 3·23)	2·61 (2·49 – 2·73)	2·65 (2·46 – 2·84)	3·04 (2·57 – 3·49)	0·64 (0·43 – 0·87)	274 625 (232 238 – 317 955)	78·68
Sub-Saharan Africa	3·87 (3·35 – 4·48)	3·85 (3·34 – 4·45)	3·55 (3·14 – 4·0)	3·67 (3·29 – 4·07)	3·8 (3·38 – 4·27)	4·15 (3·59 – 4·79)	0·27 (0·19 – 0·35)	3 670 266 (3 133 629 – 4 299 669)	118·53
Central sub-Saharan Africa	3·77 (3·24 – 4·42)	3·8 (3·28 – 4·42)	3·74 (3·26 – 4·37)	3·67 (3·19 – 4·19)	3·73 (3·23 – 4·29)	4·04 (3·43 – 4·77)	0·26 (0·04 – 0·45)	430 168 (358 847 – 518 324)	141·46
Angola	3·66 (3·11 – 4·31)	3·74 (3·18 – 4·38)	3·78 (3·27 – 4·41)	3·45 (3·14 – 3·8)	3·58 (3·27 – 3·94)	4·28 (3·65 – 4·94)	0·61 (0·31 – 0·88)	99 629 (82 353 – 116 204)	174·76
Central African Republic	3·65 (3·14 – 4·27)	3·71 (3·23 – 4·27)	3·72 (3·21 – 4·3)	3·79 (3·26 – 4·43)	3·86 (3·29 – 4·5)	3·89 (3·27 – 4·73)	0·24 (-0·09 – 0·55)	17 872 (14 677 – 22 651)	83·35
Congo (Brazzaville)	4·06 (3·46 – 4·71)	4·2 (3·62 – 5·0)	4·25 (3·62 – 4·96)	4·39 (3·73 – 5·15)	4·46 (3·84 – 5·2)	4·65 (3·97 – 5·51)	0·52 (0·17 – 0·83)	20 107 (16 975 – 24 615)	126·57
Democratic Republic of the Congo	3·78 (3·22 – 4·46)	3·77 (3·23 – 4·41)	3·67 (3·17 – 4·31)	3·64 (3·11 – 4·22)	3·67 (3·12 – 4·31)	3·89 (3·29 – 4·64)	0·11 (-0·16 – 0·36)	280 282 (232 494 – 343 722)	137·28
Equatorial Guinea	3·79 (3·22 – 4·5)	3·88 (3·34 – 4·57)	4·34 (3·7 – 5·21)	4·75 (4·11 – 5·6)	5·0 (4·3 – 5·93)	5·24 (4·46 – 6·15)	1·24 (0·95 – 1·57)	4070 (3409 – 4921)	209·92
Gabon	4·34 (3·68 – 5·08)	4·5 (3·81 – 5·23)	4·63 (3·99 – 5·49)	4·71 (4·03 – 5·51)	4·85 (4·13 – 5·6)	5·01 (4·26 – 5·98)	0·56 (0·24 – 0·85)	8208 (6851 – 10 074)	113·95
Eastern sub-Saharan Africa	3·99 (3·43 – 4·65)	4·14 (3·59 – 4·76)	3·76 (3·37 – 4·21)	3·88 (3·52 – 4·28)	3·96 (3·56 – 4·44)	4·39 (3·84 – 5·02)	0·37 (0·24 – 0·5)	1 561 084 (1 339 433 – 1 820 107)	129·34
Burundi	6·43 (5·51 – 7·78)	5·49 (4·85 – 6·42)	5·96 (5·5 – 6·45)	7·0 (6·61 – 7·37)	6·68 (5·7 – 7·75)	6·95 (5·92 – 8·21)	0·3 (-0·0 – 0·67)	74 720 (62 784 – 90 857)	125·41
Comoros	4·27 (3·6 – 5·19)	4·35 (3·75 – 5·08)	4·4 (3·82 – 5·12)	4·51 (3·85 – 5·34)	4·62 (3·9 – 5·56)	4·79 (4·07 – 5·71)	0·45 (0·18 – 0·75)	3458 (2867 – 4317)	118·47
Djibouti	4·02 (3·45 – 4·81)	4·09 (3·5 – 4·81)	4·19 (3·58 – 4·96)	4·33 (3·67 – 5·07)	4·48 (3·81 – 5·27)	4·64 (3·89 – 5·62)	0·55 (0·25 – 0·87)	4245 (3513 – 5234)	92·79
Eritrea	3·94 (3·37 – 4·65)	4·09 (3·45 – 5·13)	4·27 (3·66 – 5·07)	4·36 (3·7 – 5·12)	4·44 (3·77 – 5·32)	4·52 (3·85 – 5·22)	0·53 (0·25 – 0·88)	22 143 (18 469 – 26 160)	96·1
Ethiopia	3·57 (2·98 – 4·31)	4·39 (3·63 – 5·14)	3·27 (2·92 – 3·66)	2·82 (2·45 – 3·27)	3·18 (2·76 – 3·82)	3·8 (3·23 – 4·43)	0·24 (-0·1 – 0·54)	353 712 (292 168 – 417 388)	128·37
Kenya	4·34 (3·75 – 5·05)	3·54 (3·04 – 4·14)	3·53 (3·03 – 4·12)	4·19 (3·62 – 4·9)	3·79 (3·26 – 4·43)	4·38 (3·79 – 5·09)	0·03 (-0·03 – 0·1)	186 683 (159 851 – 221 903)	103·41
Madagascar	4·19 (3·56 – 5·03)	4·24 (3·57 – 5·06)	4·26 (3·61 – 4·97)	4·3 (3·68 – 5·05)	4·36 (3·74 – 5·09)	4·51 (3·8 – 5·51)	0·28 (-0·02 – 0·56)	104 087 (86 119 – 131 593)	131·22
Malawi	3·04 (2·59 – 3·5)	3·1 (2·61 – 3·75)	3·18 (2·68 – 3·86)	3·06 (2·77 – 3·35)	2·93 (2·66 – 3·23)	3·44 (2·92 – 4·09)	0·48 (0·22 – 0·74)	56 879 (47 167 – 67 947)	116·97
Mozambique	3·85 (3·24 – 4·6)	3·86 (3·27 – 4·61)	3·94 (3·36 – 4·71)	4·02 (3·45 – 4·76)	4·11 (3·5 – 4·82)	4·23 (3·64 – 4·96)	0·36 (0·06 – 0·7)	112 921 (94 542 – 135 721)	137·88
Rwanda	4·77 (4·09 – 5·68)	4·84 (4·15 – 5·71)	5·85 (5·41 – 6·25)	5·88 (5·44 – 6·3)	4·82 (4·4 – 5·28)	5·95 (5·53 – 6·33)	0·85 (0·37 – 1·28)	66 019 (60 787 – 71 464)	109·23
Somalia	3·96 (3·35 – 4·69)	3·97 (3·4 – 4·64)	3·94 (3·35 – 4·66)	3·97 (3·4 – 4·67)	4·05 (3·5 – 4·74)	4·1 (3·51 – 4·8)	0·14 (-0·14 – 0·46)	39 150 (32 438 – 47 323)	74·64
South Sudan	3·65 (3·1 – 4·39)	3·65 (3·09 – 4·24)	3·67 (3·13 – 4·28)	3·73 (3·18 – 4·42)	3·8 (3·23 – 4·45)	3·84 (3·28 – 4·65)	0·19 (-0·11 – 0·45)	48 410 (40 492 – 59 349)	136·14

Tanzania	4·58 (4·09 – 5·13)	4·69 (4·12 – 5·4)	4·11 (3·81 – 4·48)	4·84 (4·57 – 5·12)	4·76 (4·45 – 5·05)	5·44 (4·7 – 6·35)	0·66 (0·32 – 1·03)	273 739 (227 362 – 328 590)	154·25
Uganda	4·18 (3·57 – 5·01)	4·31 (3·71 – 5·1)	3·78 (3·38 – 4·2)	3·9 (3·48 – 4·35)	4·42 (4·25 – 4·6)	4·55 (4·29 – 4·82)	0·33 (-0·27 – 0·83)	166 752 (154 154 – 179 120)	147
Zambia	2·28 (1·91 – 2·75)	2·38 (2·13 – 2·71)	2·64 (2·41 – 2·89)	2·77 (2·57 – 2·98)	3·33 (2·89 – 3·71)	3·09 (2·62 – 3·68)	1·17 (0·76 – 1·79)	47 157 (38 543 – 58 043)	175·88
Southern sub-Saharan Africa	4·24 (3·66 – 4·88)	3·39 (2·92 – 3·94)	3·08 (2·65 – 3·57)	3·15 (2·71 – 3·64)	3·3 (2·83 – 3·8)	3·53 (3·03 – 4·08)	-0·71 (-0·8 to -0·59)	237 285 (201 002 – 277 822)	28·81
Botswana	3·48 (2·98 – 4·05)	3·57 (3·03 – 4·17)	3·68 (3·11 – 4·34)	3·64 (3·12 – 4·24)	3·67 (3·14 – 4·26)	3·78 (3·21 – 4·43)	0·32 (0·05 – 0·58)	7558 (6309 – 9058)	98·99
Lesotho	3·12 (2·68 – 3·61)	3·18 (2·72 – 3·72)	3·24 (2·77 – 3·73)	3·21 (2·76 – 3·75)	3·22 (2·75 – 3·76)	3·33 (2·83 – 3·9)	0·25 (0·01 – 0·51)	5948 (4945 – 7144)	45·18
Namibia	3·26 (2·82 – 3·81)	3·34 (2·86 – 3·9)	3·46 (2·98 – 4·04)	3·44 (2·96 – 4·0)	3·42 (2·91 – 3·96)	3·57 (3·03 – 4·15)	0·35 (0·13 – 0·59)	7573 (6346 – 9033)	100·9
South Africa	4·58 (3·95 – 5·27)	3·4 (2·93 – 3·94)	2·96 (2·53 – 3·42)	3·08 (2·65 – 3·56)	3·3 (2·85 – 3·79)	3·58 (3·08 – 4·14)	-0·95 (-1·06 to -0·81)	171 156 (145 691 – 199 552)	19·64
Swaziland (eSwatini)	3·33 (2·87 – 3·88)	3·43 (2·93 – 3·99)	3·54 (3·05 – 4·05)	3·38 (2·89 – 3·93)	3·32 (2·83 – 3·87)	3·56 (2·99 – 4·14)	0·25 (0·01 – 0·48)	3908 (3248 – 4709)	74·72
Zimbabwe	3·27 (2·8 – 3·76)	3·36 (2·87 – 3·91)	3·4 (2·91 – 3·92)	3·29 (2·81 – 3·85)	3·12 (2·66 – 3·65)	3·18 (2·7 – 3·71)	-0·11 (-0·35 – 0·13)	41 142 (34 271 – 49 725)	50·94
Western sub-Saharan Africa	3·63 (3·13 – 4·21)	3·7 (3·2 – 4·33)	3·4 (3·02 – 3·87)	3·59 (3·23 – 3·99)	3·75 (3·35 – 4·2)	4·05 (3·48 – 4·72)	0·42 (0·28 – 0·55)	1 441 729 (1 219 671 – 1 712 639)	126·53
Benin	3·64 (3·13 – 4·29)	3·73 (3·19 – 4·31)	3·82 (3·24 – 4·51)	3·86 (3·27 – 4·54)	3·98 (3·42 – 4·7)	4·06 (3·49 – 4·68)	0·42 (0·09 – 0·68)	41 545 (35 084 – 49 031)	157·61
Burkina Faso	1·58 (1·35 – 1·83)	1·62 (1·37 – 1·9)	1·43 (1·22 – 1·67)	1·46 (1·24 – 1·7)	1·93 (1·66 – 2·22)	2·0 (1·71 – 2·32)	0·92 (0·63 – 1·24)	34 367 (28 439 – 40 703)	176·44
Cameroon	4·04 (3·45 – 4·83)	4·11 (3·46 – 4·81)	4·19 (3·56 – 4·89)	4·26 (3·65 – 5·03)	4·3 (3·64 – 5·03)	4·44 (3·75 – 5·29)	0·36 (0·12 – 0·65)	95 631 (78 725 – 120 746)	121·04
Cape Verde	4·42 (3·79 – 5·21)	4·56 (3·91 – 5·29)	4·73 (4·01 – 5·62)	4·44 (4·1 – 4·76)	4·64 (4·3 – 4·96)	5·39 (4·63 – 6·39)	0·76 (0·44 – 1·09)	2749 (2318 – 3302)	104·73
Chad	3·54 (3·04 – 4·18)	3·62 (3·05 – 4·36)	3·64 (3·1 – 4·27)	3·7 (3·14 – 4·26)	3·8 (3·21 – 4·56)	3·89 (3·32 – 4·71)	0·36 (0·1 – 0·62)	49 806 (41 429 – 62 991)	167·72
Cote d'Ivoire	3·82 (3·27 – 4·54)	3·95 (3·37 – 4·59)	4·07 (3·46 – 4·93)	4·09 (3·52 – 4·81)	4·15 (3·55 – 4·86)	4·26 (3·65 – 4·99)	0·42 (0·15 – 0·71)	88 637 (73 413 – 105 785)	113·5
The Gambia	4·44 (4·23 – 4·66)	4·45 (4·22 – 4·69)	5·12 (4·35 – 6·02)	5·23 (4·47 – 6·1)	5·39 (4·62 – 6·54)	5·48 (4·65 – 6·4)	0·8 (0·24 – 1·34)	9936 (8271 – 11 797)	170·81
Ghana	4·12 (3·77 – 4·49)	4·63 (4·29 – 4·93)	4·32 (4·04 – 4·6)	3·96 (3·62 – 4·32)	3·99 (3·56 – 4·43)	4·56 (4·25 – 4·87)	0·39 (0·21 – 0·59)	116 445 (106 959 – 125 550)	121·99
Guinea	3·63 (3·11 – 4·28)	3·67 (3·12 – 4·34)	3·76 (3·17 – 4·5)	3·77 (3·2 – 4·45)	3·87 (3·35 – 4·62)	3·96 (3·36 – 4·68)	0·33 (0·07 – 0·56)	45 983 (38 444 – 55 143)	136·79
Guinea-Bissau	3·63 (3·12 – 4·24)	3·7 (3·14 – 4·39)	3·77 (3·22 – 4·4)	3·82 (3·27 – 4·49)	3·92 (3·36 – 4·74)	4·01 (3·44 – 4·7)	0·38 (0·13 – 0·66)	6941 (5836 – 8290)	102·81
Liberia	3·64 (3·08 – 4·35)	3·58 (3·05 – 4·21)	3·61 (3·05 – 4·23)	3·75 (3·19 – 4·42)	3·87 (3·32 – 4·57)	4·01 (3·41 – 4·73)	0·37 (0·13 – 0·61)	16 695 (14 030 – 20 087)	137·49
Mali	3·07 (2·61 – 3·57)	3·12 (2·66 – 3·71)	3·18 (2·71 – 3·76)	3·24 (2·77 – 3·8)	3·33 (2·85 – 3·91)	3·06 (2·79 – 3·36)	-0·02 (-0·44 – 0·39)	47 373 (42 007 – 53 072)	102·1
Mauritania	3·98 (3·41 – 4·72)	4·09 (3·46 – 4·82)	4·18 (3·52 – 4·85)	4·25 (3·64 – 5·01)	4·38 (3·75 – 5·1)	4·54 (3·86 – 5·36)	0·51 (0·23 – 0·78)	16 777 (13 939 – 20 236)	138·43
Niger	3·23 (2·77 – 3·8)	3·25 (2·76 – 3·86)	3·24 (2·75 – 3·8)	3·29 (2·81 – 3·88)	3·05 (2·75 – 3·34)	3·4 (2·87 – 4·04)	0·2 (-0·04 – 0·49)	61 251 (50 092 – 75 861)	169·74
Nigeria	3·59 (3·05 – 4·18)	3·62 (3·08 – 4·29)	3·02 (2·69 – 3·41)	3·42 (3·15 – 3·7)	3·67 (3·32 – 4·04)	4·1 (3·47 – 4·88)	0·51 (0·24 – 0·76)	677 695 (557 771 – 820 354)	123·55
Sao Tome and Principe	3·88 (3·34 – 4·68)	3·97 (3·41 – 4·7)	4·06 (3·46 – 4·76)	4·14 (3·52 – 4·81)	4·24 (3·64 – 4·98)	4·36 (3·69 – 5·18)	0·45 (0·15 – 0·72)	775 (647 – 944)	94·1
Senegal	3·74 (3·19 – 4·4)	3·82 (3·22 – 4·52)	3·9 (3·34 – 4·54)	3·95 (3·39 – 4·6)	4·01 (3·39 – 4·76)	4·08 (3·48 – 4·81)	0·34 (0·01 – 0·6)	56 790 (48 057 – 67 435)	129·77
Sierra Leone	6·66 (5·67 – 7·88)	6·77 (5·78 – 8·24)	6·13 (5·73 – 6·56)	6·14 (5·83 – 6·44)	6·41 (5·96 – 6·84)	7·43 (6·39 – 8·74)	0·42 (0·13 – 0·71)	44 206 (36 773 – 54 504)	90·25
Togo	3·72 (3·16 – 4·33)	3·88 (3·26 – 4·74)	3·96 (3·36 – 4·7)	4·03 (3·41 – 4·82)	4·13 (3·52 – 4·86)	4·23 (3·63 – 4·95)	0·49 (0·21 – 0·75)	28 110 (23 676 – 34 006)	125·7

Table S11. Global, GBD super-region, regional, and national age-standardised outpatient utilisation rates and annualised rate of change 1990 to 2016, and volume of inpatient admissions in 2016, and percentage change in volume from 1990 to 2016, both sexes combined

Table displays four sets of results: age-standardised inpatient utilisation rate per person defined as the number of inpatient admissions per person per year, the annualised rate of change in the age-standardised inpatient utilisation rate, volume of inpatient admissions in 2016, and the percentage change in volume from 1990 to 2016. For each result, the mean of 1,000 draws is reported, and in parenthesis the uncertainty interval defined as the 2.5th and 97.5th percentile of draws. GBD= Global Burden of Disease.

Table S11: Global, GBD super-region, regional, and national age-standardised inpatient utilisation rates and annualised rate of change 1990 to 2016, and volume of inpatient admissions in 2016, and percentage change in volume from 1990 to 2016, both sexes combined

	Age standardised for international comparisons							Estimates based on national age structure	
	Age standardised inpatient utilisation rate (per capita)						Annual rate of change in age-standardised rate (as percentage)	Volume of inpatient admissions per year (in thousands)	Total percentage change (Figure 3)
	1990	1995	2000	2005	2010	2016 (Figure 1b)	1990 - 2016	2016	1990 - 2016
Global	0·09 (0·08 – 0·1)	0·08 (0·07 – 0·09)	0·08 (0·08 – 0·09)	0·09 (0·08 – 0·09)	0·09 (0·08 – 0·09)	0·1 (0·09 – 0·11)	0·38 (0·24 – 0·5)	709 219 (652 249 – 774 259)	67·96
Central Europe, Eastern Europe, and Central Asia	0·21 (0·2 – 0·22)	0·19 (0·18 – 0·2)	0·19 (0·18 – 0·19)	0·19 (0·19 – 0·2)	0·19 (0·19 – 0·2)	0·19 (0·19 – 0·2)	-0·32 (-0·4 to -0·24)	83 247 (79 597 – 87 098)	-4·28
Central Asia	0·21 (0·2 – 0·23)	0·16 (0·15 – 0·16)	0·13 (0·12 – 0·13)	0·14 (0·13 – 0·14)	0·15 (0·14 – 0·15)	0·15 (0·14 – 0·15)	-1·4 (-1·61 to -1·2)	12 501 (12 005 – 13 036)	-9
Armenia	0·13 (0·13 – 0·13)	0·09 (0·09 – 0·1)	0·07 (0·07 – 0·08)	0·08 (0·08 – 0·08)	0·11 (0·11 – 0·11)	0·13 (0·12 – 0·13)	-0·11 (-0·22 to -0·01)	400 (384 – 418)	-10·84
Azerbaijan	0·15 (0·14 – 0·15)	0·09 (0·08 – 0·09)	0·05 (0·05 – 0·06)	0·06 (0·06 – 0·06)	0·06 (0·06 – 0·06)	0·07 (0·06 – 0·07)	-3·02 (-3·11 to -2·93)	650 (626 – 675)	-34·53
Georgia	0·12 (0·12 – 0·13)	0·06 (0·06 – 0·07)	0·05 (0·05 – 0·05)	0·05 (0·05 – 0·06)	0·07 (0·07 – 0·07)	0·09 (0·08 – 0·09)	-1·29 (-1·39 to -1·19)	376 (361 – 392)	-44·24
Kazakhstan	0·25 (0·24 – 0·25)	0·19 (0·18 – 0·19)	0·16 (0·15 – 0·16)	0·17 (0·17 – 0·18)	0·17 (0·17 – 0·17)	0·16 (0·15 – 0·16)	-1·77 (-1·87 to -1·67)	2759 (2650 – 2862)	-30·34
Kyrgyzstan	0·26 (0·25 – 0·26)	0·18 (0·17 – 0·19)	0·15 (0·15 – 0·16)	0·15 (0·15 – 0·15)	0·16 (0·16 – 0·17)	0·16 (0·15 – 0·16)	-1·87 (-1·98 to -1·76)	894 (857 – 929)	-11·35
Mongolia	0·17 (0·15 – 0·21)	0·17 (0·14 – 0·2)	0·16 (0·14 – 0·19)	0·16 (0·13 – 0·19)	0·16 (0·13 – 0·19)	0·16 (0·13 – 0·19)	-0·34 (-0·73 – 0·07)	461 (381 – 562)	30·67
Tajikistan	0·22 (0·22 – 0·23)	0·16 (0·15 – 0·16)	0·11 (0·11 – 0·12)	0·13 (0·12 – 0·14)	0·14 (0·14 – 0·15)	0·14 (0·13 – 0·14)	-1·88 (-1·97 to -1·78)	1064 (1024 – 1108)	-1·18
Turkmenistan	0·17 (0·15 – 0·21)	0·17 (0·14 – 0·2)	0·16 (0·14 – 0·19)	0·16 (0·13 – 0·19)	0·15 (0·13 – 0·18)	0·15 (0·13 – 0·18)	-0·48 (-0·87 to -0·1)	801 (663 – 977)	34·04
Uzbekistan	0·24 (0·2 – 0·29)	0·18 (0·18 – 0·19)	0·15 (0·15 – 0·16)	0·16 (0·16 – 0·17)	0·17 (0·17 – 0·18)	0·18 (0·17 – 0·18)	-1·18 (-1·86 to -0·59)	5097 (4902 – 5291)	10·17
Central Europe	0·17 (0·16 – 0·18)	0·17 (0·16 – 0·17)	0·17 (0·16 – 0·17)	0·17 (0·16 – 0·17)	0·17 (0·16 – 0·17)	0·18 (0·17 – 0·2)	0·29 (0·16 – 0·41)	22 459 (21 089 – 24 010)	8·13
Albania	0·1 (0·09 – 0·11)	0·09 (0·09 – 0·09)	0·09 (0·09 – 0·09)	0·09 (0·09 – 0·09)	0·09 (0·09 – 0·1)	0·09 (0·09 – 0·1)	-0·17 (-0·37 – 0·02)	268 (251 – 285)	-15·31
Bosnia and Herzegovina	0·12 (0·1 – 0·14)	0·11 (0·1 – 0·13)	0·09 (0·08 – 0·1)	0·1 (0·09 – 0·12)	0·11 (0·09 – 0·13)	0·11 (0·09 – 0·13)	-0·12 (-0·51 – 0·21)	440 (368 – 519)	-11
Bulgaria	0·2 (0·19 – 0·21)	0·18 (0·17 – 0·18)	0·15 (0·15 – 0·16)	0·17 (0·16 – 0·17)	0·21 (0·2 – 0·22)	0·27 (0·26 – 0·28)	1·18 (1·03 – 1·33)	2120 (1999 – 2238)	21·48
Croatia	0·14 (0·14 – 0·15)	0·14 (0·13 – 0·14)	0·14 (0·14 – 0·15)	0·15 (0·15 – 0·15)	0·16 (0·15 – 0·16)	0·17 (0·16 – 0·17)	0·55 (0·44 – 0·67)	767 (730 – 804)	13·33
Czech Republic	0·27 (0·23 – 0·31)	0·22 (0·2 – 0·25)	0·2 (0·19 – 0·2)	0·19 (0·19 – 0·19)	0·18 (0·18 – 0·19)	0·23 (0·2 – 0·28)	-0·5 (-0·99 to -0·07)	2643 (2311 – 3092)	-3·69
Hungary	0·22 (0·21 – 0·23)	0·22 (0·21 – 0·23)	0·22 (0·22 – 0·22)	0·2 (0·2 – 0·21)	0·18 (0·18 – 0·18)	0·2 (0·19 – 0·21)	-0·42 (-0·56 to -0·29)	2089 (1974 – 2217)	-9·18
Macedonia	0·11 (0·1 – 0·11)	0·1 (0·1 – 0·11)	0·1 (0·1 – 0·11)	0·1 (0·1 – 0·11)	0·12 (0·11 – 0·13)	0·12 (0·11 – 0·13)	0·63 (0·37 – 0·92)	254 (234 – 275)	27·26
Montenegro	0·14 (0·12 – 0·17)	0·14 (0·12 – 0·17)	0·14 (0·12 – 0·17)	0·11 (0·1 – 0·11)	0·11 (0·11 – 0·12)	0·12 (0·11 – 0·13)	-0·74 (-1·3 to -0·15)	76 (72 – 80)	-11·58
Poland	0·13 (0·12 – 0·13)	0·14 (0·13 – 0·14)	0·15 (0·15 – 0·16)	0·15 (0·15 – 0·15)	0·15 (0·15 – 0·15)	0·17 (0·16 – 0·17)	1·0 (0·81 – 1·17)	6627 (6217 – 7019)	39·23
Romania	0·21 (0·21 – 0·22)	0·21 (0·2 – 0·21)	0·22 (0·22 – 0·23)	0·23 (0·23 – 0·24)	0·22 (0·21 – 0·23)	0·22 (0·21 – 0·24)	0·14 (-0·11 – 0·43)	4572 (4231 – 4968)	-8·38
Serbia	0·13 (0·11 – 0·15)	0·13 (0·11 – 0·15)	0·09 (0·07 – 0·1)	0·08 (0·07 – 0·09)	0·11 (0·11 – 0·12)	0·12 (0·11 – 0·12)	-0·42 (-0·96 – 0·17)	1096 (1041 – 1155)	-10·32
Slovakia	0·18 (0·17 – 0·18)	0·2 (0·19 – 0·2)	0·2 (0·2 – 0·21)	0·19 (0·19 – 0·2)	0·19 (0·19 – 0·2)	0·2 (0·19 – 0·21)	0·48 (0·3 – 0·66)	1121 (1056 – 1190)	22·43
Slovenia	0·16 (0·16 – 0·17)	0·16 (0·16 – 0·17)	0·17 (0·16 – 0·17)	0·17 (0·16 – 0·17)	0·16 (0·16 – 0·16)	0·18 (0·17 – 0·19)	0·37 (0·2 – 0·55)	386 (365 – 407)	20·69
Eastern Europe	0·24 (0·23 – 0·25)	0·22 (0·21 – 0·23)	0·22 (0·21 – 0·23)	0·23 (0·22 – 0·24)	0·23 (0·22 – 0·24)	0·23 (0·22 – 0·24)	-0·24 (-0·35 to -0·12)	48 288 (46 312 – 50 290)	-7·96
Belarus	0·27 (0·22 – 0·32)	0·26 (0·22 – 0·31)	0·26 (0·22 – 0·31)	0·26 (0·21 – 0·3)	0·23 (0·2 – 0·27)	0·25 (0·21 – 0·3)	-0·2 (-0·48 – 0·09)	2411 (2022 – 2863)	-10·21
Estonia	0·2 (0·19 – 0·21)	0·2 (0·19 – 0·2)	0·2 (0·2 – 0·21)	0·2 (0·19 – 0·2)	0·19 (0·18 – 0·19)	0·18 (0·17 – 0·19)	-0·33 (-0·47 to -0·19)	246 (236 – 255)	-21·01
Latvia	0·24 (0·23 – 0·24)	0·22 (0·21 – 0·22)	0·22 (0·22 – 0·22)	0·21 (0·21 – 0·21)	0·2 (0·19 – 0·2)	0·18 (0·18 – 0·19)	-0·98 (-1·07 to -0·9)	384 (372 – 398)	-38·21
Lithuania	0·21 (0·2 – 0·21)	0·23 (0·22 – 0·24)	0·23 (0·22 – 0·23)	0·22 (0·21 – 0·22)	0·22 (0·21 – 0·22)	0·24 (0·22 – 0·25)	0·53 (0·33 – 0·73)	743 (698 – 794)	-1·79
Moldova	0·24 (0·23 – 0·25)	0·21 (0·21 – 0·22)	0·17 (0·16 – 0·17)	0·15 (0·15 – 0·16)	0·17 (0·16 – 0·17)	0·17 (0·17 – 0·18)	-1·25 (-1·38 to -1·13)	676 (645 – 707)	-35·48

Russia	0·23 (0·23 – 0·24)	0·22 (0·21 – 0·23)	0·23 (0·22 – 0·24)	0·24 (0·23 – 0·25)	0·23 (0·22 – 0·24)	0·22 (0·21 – 0·23)	-0·17 (-0·3 to -0·04)	33 170 (31 916 – 34 357)	-2·35
Ukraine	0·26 (0·25 – 0·26)	0·22 (0·22 – 0·23)	0·2 (0·2 – 0·21)	0·22 (0·22 – 0·23)	0·23 (0·23 – 0·24)	0·24 (0·22 – 0·25)	-0·33 (-0·49 to -0·17)	10 657 (10 088 – 11 214)	-18·45
High-income	0·14 (0·13 – 0·16)	0·13 (0·12 – 0·15)	0·13 (0·12 – 0·14)	0·13 (0·12 – 0·13)	0·12 (0·12 – 0·13)	0·13 (0·12 – 0·14)	-0·35 (-0·5 to -0·21)	152 621 (141 609 – 164 276)	16·74
Australasia	0·18 (0·15 – 0·21)	0·15 (0·13 – 0·17)	0·16 (0·14 – 0·18)	0·15 (0·14 – 0·16)	0·16 (0·16 – 0·17)	0·16 (0·15 – 0·18)	-0·31 (-0·76 – 0·11)	4807 (4412 – 5203)	36·18
Australia	0·17 (0·15 – 0·2)	0·14 (0·12 – 0·16)	0·16 (0·13 – 0·18)	0·15 (0·14 – 0·16)	0·16 (0·16 – 0·17)	0·16 (0·15 – 0·18)	-0·2 (-0·65 – 0·23)	4096 (3716 – 4477)	41·42
New Zealand	0·19 (0·16 – 0·22)	0·18 (0·16 – 0·22)	0·15 (0·15 – 0·16)	0·15 (0·14 – 0·15)	0·15 (0·15 – 0·15)	0·15 (0·15 – 0·16)	-0·83 (-1·44 to -0·25)	712 (686 – 738)	12·22
High-income Asia-Pacific	0·1 (0·09 – 0·12)	0·09 (0·08 – 0·1)	0·1 (0·09 – 0·11)	0·11 (0·1 – 0·12)	0·12 (0·1 – 0·13)	0·12 (0·11 – 0·14)	0·69 (0·46 – 0·93)	23 322 (20 565 – 26 427)	42·27
Brunei	0·12 (0·1 – 0·14)	0·12 (0·1 – 0·14)	0·12 (0·1 – 0·14)	0·12 (0·1 – 0·14)	0·12 (0·1 – 0·14)	0·12 (0·1 – 0·14)	0·15 (-0·22 – 0·54)	47 (39 – 56)	61·57
Japan	0·08 (0·07 – 0·1)	0·09 (0·07 – 0·1)	0·1 (0·08 – 0·12)	0·1 (0·08 – 0·12)	0·1 (0·09 – 0·12)	0·1 (0·09 – 0·12)	0·99 (0·74 – 1·23)	14 500 (12 051 – 17 312)	50·59
Singapore	0·12 (0·1 – 0·15)	0·12 (0·1 – 0·15)	0·12 (0·1 – 0·14)	0·12 (0·1 – 0·15)	0·12 (0·1 – 0·14)	0·12 (0·1 – 0·15)	0·01 (-0·33 – 0·34)	459 (385 – 538)	44·63
South Korea	0·16 (0·14 – 0·19)	0·09 (0·09 – 0·1)	0·1 (0·1 – 0·11)	0·12 (0·12 – 0·13)	0·16 (0·15 – 0·16)	0·17 (0·16 – 0·18)	0·18 (-0·44 – 0·79)	8316 (7836 – 8824)	29·58
High-income North America	0·14 (0·12 – 0·16)	0·13 (0·11 – 0·16)	0·12 (0·11 – 0·14)	0·12 (0·11 – 0·13)	0·11 (0·1 – 0·12)	0·11 (0·1 – 0·12)	-0·89 (-1·28 to -0·51)	44 246 (40 504 – 48 103)	8·54
Canada	0·13 (0·11 – 0·15)	0·12 (0·11 – 0·15)	0·11 (0·1 – 0·12)	0·11 (0·1 – 0·12)	0·1 (0·09 – 0·1)	0·12 (0·1 – 0·14)	-0·39 (-0·71 to -0·08)	4999 (4175 – 5905)	36·27
Greenland	0·14 (0·11 – 0·16)	0·13 (0·11 – 0·16)	0·13 (0·11 – 0·15)	0·13 (0·11 – 0·15)	0·13 (0·11 – 0·15)	0·13 (0·11 – 0·15)	-0·1 (-0·38 – 0·2)	6 (5 – 8)	6·89
United States	0·14 (0·12 – 0·17)	0·13 (0·11 – 0·16)	0·12 (0·11 – 0·14)	0·12 (0·11 – 0·13)	0·11 (0·1 – 0·12)	0·11 (0·1 – 0·12)	-0·95 (-1·39 to -0·52)	39 225 (36 277 – 42 441)	5·81
Southern Latin America	0·09 (0·08 – 0·11)	0·09 (0·08 – 0·1)	0·09 (0·08 – 0·1)	0·09 (0·08 – 0·1)	0·09 (0·08 – 0·1)	0·09 (0·08 – 0·1)	-0·23 (-0·48 – 0·04)	5802 (5034 – 6684)	33·15
Argentina	0·09 (0·08 – 0·11)	0·09 (0·08 – 0·11)	0·09 (0·08 – 0·11)	0·09 (0·08 – 0·11)	0·09 (0·08 – 0·11)	0·09 (0·08 – 0·11)	-0·04 (-0·38 – 0·33)	3990 (3396 – 4669)	38·29
Chile	0·11 (0·1 – 0·12)	0·1 (0·1 – 0·11)	0·1 (0·1 – 0·1)	0·1 (0·1 – 0·1)	0·09 (0·09 – 0·09)	0·09 (0·08 – 0·1)	-0·84 (-1·12 to -0·59)	1601 (1427 – 1799)	25·52
Uruguay	0·06 (0·05 – 0·08)	0·06 (0·05 – 0·07)	0·06 (0·05 – 0·07)	0·06 (0·05 – 0·07)	0·06 (0·05 – 0·07)	0·06 (0·05 – 0·07)	-0·32 (-0·62 – 0·04)	211 (182 – 250)	7·24
Western Europe	0·16 (0·16 – 0·17)	0·16 (0·16 – 0·17)	0·15 (0·15 – 0·16)	0·15 (0·14 – 0·15)	0·14 (0·14 – 0·15)	0·15 (0·14 – 0·17)	-0·22 (-0·34 to -0·1)	74 445 (70 227 – 78 993)	13·32
Andorra	0·15 (0·13 – 0·18)	0·15 (0·13 – 0·18)	0·15 (0·13 – 0·18)	0·15 (0·13 – 0·18)	0·15 (0·13 – 0·18)	0·15 (0·13 – 0·18)	-0·08 (-0·43 – 0·29)	14 (11 – 16)	70·32
Austria	0·2 (0·2 – 0·2)	0·21 (0·21 – 0·22)	0·22 (0·22 – 0·23)	0·23 (0·22 – 0·23)	0·22 (0·22 – 0·22)	0·25 (0·24 – 0·26)	0·82 (0·6 – 1·02)	2355 (2243 – 2470)	37·81
Belgium	0·19 (0·16 – 0·23)	0·19 (0·16 – 0·22)	0·14 (0·14 – 0·14)	0·14 (0·14 – 0·14)	0·14 (0·14 – 0·15)	0·15 (0·14 – 0·16)	-0·87 (-1·47 to -0·28)	1999 (1884 – 2125)	-1·35
Cyprus	0·11 (0·09 – 0·12)	0·1 (0·09 – 0·12)	0·1 (0·09 – 0·12)	0·1 (0·08 – 0·12)	0·08 (0·07 – 0·09)	0·1 (0·08 – 0·12)	-0·25 (-0·6 – 0·08)	95 (79 – 112)	35·24
Denmark	0·19 (0·18 – 0·19)	0·18 (0·18 – 0·19)	0·16 (0·16 – 0·17)	0·15 (0·14 – 0·15)	0·14 (0·14 – 0·15)	0·14 (0·14 – 0·15)	-1·04 (-1·18 to -0·89)	905 (864 – 948)	-12·95
Finland	0·22 (0·21 – 0·23)	0·22 (0·21 – 0·23)	0·18 (0·17 – 0·18)	0·15 (0·15 – 0·15)	0·13 (0·13 – 0·14)	0·15 (0·14 – 0·16)	-1·42 (-1·59 to -1·25)	981 (921 – 1046)	-14·57
France	0·22 (0·21 – 0·22)	0·22 (0·21 – 0·22)	0·2 (0·19 – 0·2)	0·18 (0·17 – 0·18)	0·17 (0·17 – 0·18)	0·18 (0·17 – 0·19)	-0·74 (-0·99 to -0·47)	13 053 (12 235 – 14 041)	0·36
Germany	0·18 (0·18 – 0·19)	0·2 (0·19 – 0·2)	0·19 (0·19 – 0·2)	0·19 (0·19 – 0·19)	0·2 (0·19 – 0·2)	0·23 (0·22 – 0·24)	0·84 (0·68 – 0·99)	21 120 (20 189 – 22 079)	37·2
Greece	0·12 (0·12 – 0·13)	0·13 (0·13 – 0·14)	0·15 (0·14 – 0·15)	0·16 (0·16 – 0·17)	0·18 (0·17 – 0·18)	0·17 (0·14 – 0·19)	1·11 (0·47 – 1·73)	2134 (1759 – 2531)	63·32
Iceland	0·22 (0·21 – 0·23)	0·21 (0·2 – 0·22)	0·18 (0·17 – 0·18)	0·15 (0·14 – 0·16)	0·13 (0·12 – 0·13)	0·12 (0·11 – 0·13)	-2·29 (-2·42 to -2·16)	43 (41 – 45)	-23·03
Ireland	0·15 (0·14 – 0·15)	0·15 (0·14 – 0·15)	0·14 (0·14 – 0·14)	0·13 (0·13 – 0·13)	0·13 (0·12 – 0·13)	0·14 (0·13 – 0·15)	-0·26 (-0·46 to -0·07)	685 (643 – 730)	31·04
Israel	0·22 (0·18 – 0·26)	0·2 (0·18 – 0·23)	0·18 (0·17 – 0·18)	0·18 (0·17 – 0·18)	0·16 (0·16 – 0·17)	0·16 (0·16 – 0·17)	-1·06 (-1·65 to -0·49)	1389 (1324 – 1453)	44·59
Italy	0·16 (0·15 – 0·16)	0·16 (0·15 – 0·16)	0·14 (0·13 – 0·14)	0·12 (0·12 – 0·12)	0·11 (0·11 – 0·11)	0·11 (0·1 – 0·12)	-1·3 (-1·54 to -1·04)	7972 (7373 – 8622)	-14·73
Luxembourg	0·19 (0·16 – 0·22)	0·19 (0·16 – 0·22)	0·15 (0·15 – 0·15)	0·14 (0·14 – 0·14)	0·13 (0·13 – 0·13)	0·15 (0·14 – 0·16)	-0·94 (-1·51 to -0·33)	91 (86 – 97)	22·24
Malta	0·13 (0·11 – 0·15)	0·13 (0·11 – 0·15)	0·12 (0·1 – 0·15)	0·09 (0·09 – 0·1)	0·11 (0·1 – 0·12)	0·12 (0·1 – 0·14)	-0·21 (-0·57 – 0·11)	58 (48 – 70)	27·75
Netherlands	0·1 (0·09 – 0·1)	0·1 (0·09 – 0·1)	0·09 (0·09 – 0·1)	0·09 (0·09 – 0·1)	0·1 (0·1 – 0·1)	0·11 (0·1 – 0·13)	0·57 (0·08 – 1·05)	2203 (1945 – 2493)	45·95
Norway	0·14 (0·14 – 0·15)	0·14 (0·14 – 0·15)	0·16 (0·15 – 0·16)	0·17 (0·16 – 0·17)	0·18 (0·17 – 0·18)	0·18 (0·17 – 0·19)	0·87 (0·73 – 1·02)	1042 (991 – 1096)	57·67
Portugal	0·09 (0·09 – 0·1)	0·1 (0·1 – 0·11)	0·11 (0·1 – 0·11)	0·11 (0·1 – 0·11)	0·1 (0·1 – 0·1)	0·1 (0·09 – 0·1)	0·1 (-0·03 – 0·24)	1184 (1116 – 1249)	21·35
Spain	0·1 (0·09 – 0·1)	0·1 (0·1 – 0·11)	0·11 (0·11 – 0·11)	0·11 (0·11 – 0·11)	0·1 (0·1 – 0·11)	0·1 (0·1 – 0·11)	0·3 (0·18 – 0·44)	5440 (5216 – 5660)	42·21
Sweden	0·17 (0·14 – 0·2)	0·16 (0·13 – 0·19)	0·14 (0·12 – 0·17)	0·13 (0·11 – 0·15)	0·12 (0·11 – 0·15)	0·13 (0·11 – 0·15)	-1·07 (-1·41 to -0·75)	1412 (1193 – 1658)	-11·26
Switzerland	0·19 (0·16 – 0·22)	0·17 (0·15 – 0·18)	0·13 (0·13 – 0·14)	0·13 (0·13 – 0·14)	0·14 (0·14 – 0·14)	0·16 (0·15 – 0·17)	-0·7 (-1·26 to -0·12)	1444 (1366 – 1522)	7·04

United Kingdom	0·15 (0·12 – 0·17)	0·13 (0·11 – 0·14)	0·11 (0·1 – 0·13)	0·11 (0·1 – 0·13)	0·11 (0·1 – 0·13)	0·12 (0·11 – 0·14)	-0·7 (-0·87 to -0·52)	8752 (7572 – 10 072)	-2·77
Latin America and Caribbean	0·08 (0·07 – 0·1)	0·08 (0·07 – 0·1)	0·08 (0·07 – 0·09)	0·08 (0·07 – 0·09)	0·07 (0·06 – 0·08)	0·07 (0·07 – 0·08)	-0·53 (-0·82 to -0·25)	40 529 (35 914 – 46 741)	32·59
Andean Latin America	0·07 (0·06 – 0·08)	0·07 (0·06 – 0·07)	0·08 (0·07 – 0·08)	0·06 (0·06 – 0·06)	0·06 (0·06 – 0·07)	0·07 (0·07 – 0·08)	0·38 (0·15 – 0·62)	4235 (3790 – 4817)	83·29
Bolivia	0·07 (0·06 – 0·09)	0·07 (0·06 – 0·09)	0·07 (0·06 – 0·09)	0·07 (0·06 – 0·09)	0·07 (0·06 – 0·08)	0·07 (0·06 – 0·09)	-0·08 (-0·47 – 0·29)	761 (645 – 917)	64·12
Ecuador	0·08 (0·07 – 0·1)	0·06 (0·06 – 0·06)	0·06 (0·06 – 0·06)	0·06 (0·06 – 0·06)	0·07 (0·07 – 0·08)	0·08 (0·08 – 0·08)	-0·16 (-0·85 – 0·51)	1241 (1209 – 1276)	60·75
Peru	0·06 (0·05 – 0·07)	0·07 (0·06 – 0·08)	0·08 (0·07 – 0·09)	0·05 (0·05 – 0·05)	0·06 (0·05 – 0·06)	0·07 (0·06 – 0·08)	0·87 (0·41 – 1·24)	2233 (1889 – 2655)	107·77
Caribbean	0·08 (0·07 – 0·09)	0·08 (0·06 – 0·09)	0·07 (0·06 – 0·09)	0·08 (0·06 – 0·09)	0·08 (0·07 – 0·09)	0·08 (0·07 – 0·09)	-0·0 (-0·15 – 0·16)	3453 (2957 – 4080)	40·81
Antigua and Barbuda	0·08 (0·07 – 0·09)	0·08 (0·06 – 0·09)	0·07 (0·06 – 0·09)	0·07 (0·06 – 0·09)	0·07 (0·06 – 0·08)	0·07 (0·06 – 0·08)	-0·35 (-0·69 to -0·04)	6 (5 – 7)	41·61
The Bahamas	0·1 (0·08 – 0·12)	0·1 (0·08 – 0·12)	0·08 (0·07 – 0·08)	0·1 (0·08 – 0·11)	0·09 (0·08 – 0·11)	0·1 (0·08 – 0·11)	-0·19 (-0·53 – 0·2)	38 (31 – 46)	70·35
Barbados	0·1 (0·09 – 0·12)	0·1 (0·09 – 0·12)	0·1 (0·09 – 0·12)	0·1 (0·09 – 0·12)	0·11 (0·09 – 0·12)	0·1 (0·09 – 0·12)	-0·03 (-0·4 – 0·31)	33 (27 – 39)	22·44
Belize	0·07 (0·06 – 0·09)	0·07 (0·06 – 0·08)	0·07 (0·06 – 0·08)	0·06 (0·05 – 0·08)	0·06 (0·05 – 0·07)	0·06 (0·05 – 0·08)	-0·49 (-0·75 to -0·13)	20 (17 – 25)	72·41
Bermuda	0·08 (0·07 – 0·1)	0·08 (0·07 – 0·1)	0·08 (0·07 – 0·1)	0·08 (0·07 – 0·1)	0·08 (0·07 – 0·1)	0·08 (0·07 – 0·1)	0·02 (-0·29 – 0·35)	6 (5 – 7)	46·31
Cuba	0·08 (0·07 – 0·1)	0·08 (0·07 – 0·1)	0·08 (0·07 – 0·1)	0·08 (0·07 – 0·1)	0·08 (0·07 – 0·1)	0·08 (0·07 – 0·1)	-0·03 (-0·34 – 0·33)	1036 (868 – 1229)	25·14
Dominica	0·07 (0·06 – 0·09)	0·07 (0·06 – 0·09)	0·08 (0·06 – 0·09)	0·08 (0·06 – 0·09)	0·08 (0·06 – 0·09)	0·08 (0·06 – 0·09)	0·14 (-0·17 – 0·43)	6 (5 – 7)	22·9
Dominican Republic	0·1 (0·08 – 0·11)	0·1 (0·08 – 0·11)	0·09 (0·08 – 0·1)	0·09 (0·08 – 0·1)	0·09 (0·08 – 0·11)	0·09 (0·08 – 0·11)	-0·1 (-0·44 – 0·23)	921 (779 – 1100)	58·9
Grenada	0·08 (0·07 – 0·1)	0·08 (0·07 – 0·1)	0·08 (0·07 – 0·09)	0·08 (0·07 – 0·09)	0·08 (0·07 – 0·09)	0·08 (0·06 – 0·09)	-0·29 (-0·61 – 0·04)	8 (6 – 9)	4·44
Guyana	0·05 (0·04 – 0·06)	0·05 (0·04 – 0·06)	0·05 (0·04 – 0·06)	0·05 (0·04 – 0·06)	0·05 (0·04 – 0·06)	0·05 (0·04 – 0·06)	-0·06 (-0·43 – 0·25)	33 (28 – 40)	10·7
Haiti	0·06 (0·05 – 0·07)	0·06 (0·05 – 0·07)	0·06 (0·05 – 0·07)	0·06 (0·05 – 0·07)	0·06 (0·05 – 0·07)	0·06 (0·05 – 0·07)	0·27 (-0·04 – 0·62)	600 (507 – 728)	72·98
Jamaica	0·03 (0·02 – 0·03)	0·03 (0·02 – 0·03)	0·03 (0·02 – 0·03)	0·03 (0·02 – 0·04)	0·03 (0·02 – 0·04)	0·03 (0·02 – 0·04)	0·29 (-0·04 – 0·62)	84 (70 – 99)	42·76
Puerto Rico	0·08 (0·06 – 0·09)	0·07 (0·06 – 0·09)	0·07 (0·06 – 0·09)	0·08 (0·06 – 0·09)	0·08 (0·06 – 0·09)	0·08 (0·06 – 0·09)	-0·01 (-0·36 – 0·36)	304 (254 – 361)	17·32
Saint Lucia	0·08 (0·06 – 0·09)	0·07 (0·06 – 0·09)	0·07 (0·06 – 0·09)	0·07 (0·06 – 0·08)	0·07 (0·06 – 0·08)	0·07 (0·06 – 0·08)	-0·27 (-0·6 – 0·09)	13 (11 – 15)	31·27
Saint Vincent and the Grenadines	0·08 (0·06 – 0·09)	0·08 (0·07 – 0·09)	0·08 (0·06 – 0·09)	0·08 (0·06 – 0·09)	0·08 (0·06 – 0·09)	0·08 (0·06 – 0·09)	-0·03 (-0·38 – 0·32)	8 (7 – 10)	‥
Suriname	0·08 (0·07 – 0·09)	0·08 (0·06 – 0·09)	0·07 (0·06 – 0·09)	0·07 (0·06 – 0·09)	0·07 (0·06 – 0·09)	0·07 (0·06 – 0·09)	-0·24 (-0·58 – 0·06)	38 (32 – 46)	34·64
Trinidad and Tobago	0·12 (0·1 – 0·14)	0·12 (0·1 – 0·14)	0·11 (0·09 – 0·13)	0·11 (0·09 – 0·13)	0·11 (0·09 – 0·13)	0·11 (0·09 – 0·13)	-0·23 (-0·55 – 0·11)	152 (127 – 183)	17·95
Virgin Islands U·S·	0·09 (0·07 – 0·11)	0·09 (0·08 – 0·11)	0·09 (0·08 – 0·11)	0·09 (0·08 – 0·11)	0·09 (0·08 – 0·11)	0·09 (0·08 – 0·11)	0·17 (-0·17 – 0·5)	11 (9 – 14)	‥
Central Latin America	0·06 (0·05 – 0·08)	0·06 (0·06 – 0·07)	0·06 (0·06 – 0·08)	0·06 (0·05 – 0·07)	0·06 (0·05 – 0·06)	0·06 (0·05 – 0·07)	-0·33 (-0·54 to -0·12)	14 448 (12 520 – 16 977)	49·33
Colombia	0·06 (0·05 – 0·07)	0·08 (0·07 – 0·09)	0·07 (0·06 – 0·07)	0·06 (0·05 – 0·06)	0·05 (0·04 – 0·05)	0·04 (0·04 – 0·04)	-1·9 (-2·56 to -1·23)	1772 (1669 – 1929)	-2·97
Costa Rica	0·06 (0·05 – 0·07)	0·06 (0·05 – 0·07)	0·06 (0·05 – 0·07)	0·05 (0·04 – 0·06)	0·05 (0·05 – 0·06)	0·05 (0·05 – 0·07)	-0·17 (-0·44 – 0·21)	261 (221 – 318)	65·47
El Salvador	0·03 (0·02 – 0·03)	0·03 (0·02 – 0·03)	0·03 (0·02 – 0·03)	0·02 (0·02 – 0·03)	0·02 (0·02 – 0·02)	0·02 (0·02 – 0·03)	-0·66 (-1·21 to -0·23)	128 (112 – 145)	5·2
Guatemala	0·06 (0·05 – 0·08)	0·06 (0·05 – 0·08)	0·06 (0·05 – 0·06)	0·06 (0·05 – 0·06)	0·06 (0·05 – 0·07)	0·06 (0·05 – 0·07)	-0·24 (-0·55 – 0·09)	887 (739 – 1081)	77·13
Honduras	0·06 (0·05 – 0·07)	0·06 (0·05 – 0·07)	0·06 (0·05 – 0·07)	0·06 (0·05 – 0·07)	0·06 (0·05 – 0·07)	0·06 (0·05 – 0·07)	0·0 (-0·41 – 0·39)	433 (351 – 541)	79·31
Mexico	0·06 (0·05 – 0·08)	0·06 (0·05 – 0·07)	0·06 (0·05 – 0·08)	0·05 (0·05 – 0·06)	0·05 (0·05 – 0·06)	0·06 (0·05 – 0·08)	-0·07 (-0·31 – 0·17)	7813 (6533 – 9529)	55·79
Nicaragua	0·06 (0·05 – 0·07)	0·06 (0·05 – 0·06)	0·06 (0·05 – 0·07)	0·06 (0·05 – 0·07)	0·06 (0·05 – 0·07)	0·06 (0·05 – 0·07)	0·07 (-0·36 – 0·4)	338 (282 – 411)	85·22
Panama	0·1 (0·09 – 0·12)	0·1 (0·09 – 0·12)	0·1 (0·08 – 0·11)	0·1 (0·08 – 0·11)	0·1 (0·09 – 0·12)	0·1 (0·09 – 0·12)	-0·01 (-0·37 – 0·34)	398 (335 – 465)	77·1
Venezuela	0·08 (0·07 – 0·1)	0·08 (0·07 – 0·1)	0·08 (0·07 – 0·1)	0·08 (0·07 – 0·09)	0·09 (0·07 – 0·1)	0·08 (0·07 – 0·1)	-0·04 (-0·47 – 0·41)	2417 (2033 – 2823)	72·12
Tropical Latin America	0·11 (0·09 – 0·14)	0·11 (0·09 – 0·14)	0·1 (0·09 – 0·12)	0·1 (0·09 – 0·12)	0·09 (0·08 – 0·1)	0·09 (0·08 – 0·1)	-0·94 (-1·42 to -0·44)	18 392 (16 286 – 20 965)	14·04
Brazil	0·11 (0·09 – 0·14)	0·11 (0·09 – 0·14)	0·1 (0·09 – 0·12)	0·1 (0·09 – 0·12)	0·09 (0·08 – 0·1)	0·09 (0·08 – 0·1)	-0·95 (-1·45 to -0·44)	17 988 (15 946 – 20 517)	13·25
Paraguay	0·07 (0·06 – 0·08)	0·06 (0·05 – 0·07)	0·06 (0·05 – 0·07)	0·06 (0·06 – 0·07)	0·07 (0·05 – 0·08)	0·07 (0·06 – 0·08)	-0·01 (-0·42 – 0·37)	405 (343 – 485)	64·8
North Africa and Middle East	0·09 (0·08 – 0·11)	0·09 (0·08 – 0·11)	0·09 (0·08 – 0·11)	0·1 (0·09 – 0·11)	0·1 (0·09 – 0·12)	0·11 (0·1 – 0·12)	0·57 (0·44 – 0·72)	56 899 (50 246 – 65 831)	105·51
Afghanistan	0·08 (0·07 – 0·1)	0·09 (0·07 – 0·1)	0·09 (0·08 – 0·11)	0·09 (0·08 – 0·11)	0·07 (0·07 – 0·08)	0·09 (0·08 – 0·11)	0·48 (0·07 – 0·88)	2722 (2236 – 3313)	216
Algeria	0·1 (0·08 – 0·12)	0·1 (0·08 – 0·12)	0·1 (0·08 – 0·12)	0·09 (0·08 – 0·11)	0·09 (0·08 – 0·11)	0·1 (0·08 – 0·12)	-0·12 (-0·6 – 0·32)	3645 (2944 – 4532)	61·17

Bahrain	0·05 (0·04 – 0·06)	0·05 (0·04 – 0·06)	0·05 (0·04 – 0·06)	0·05 (0·04 – 0·06)	0·04 (0·04 – 0·05)	0·05 (0·04 – 0·06)	-0·25 (-0·59 – 0·11)	58 (47 – 73)	166·99
Egypt	0·17 (0·14 – 0·2)	0·17 (0·14 – 0·2)	0·16 (0·14 – 0·18)	0·16 (0·14 – 0·19)	0·16 (0·13 – 0·19)	0·16 (0·14 – 0·19)	-0·13 (-0·51 – 0·24)	13 745 (11 539 – 16 393)	61·54
Iran	0·07 (0·06 – 0·08)	0·07 (0·06 – 0·08)	0·06 (0·06 – 0·08)	0·07 (0·06 – 0·08)	0·07 (0·06 – 0·08)	0·07 (0·06 – 0·08)	-0·11 (-0·49 – 0·34)	4987 (4177 – 6191)	48·07
Iraq	0·09 (0·07 – 0·11)	0·09 (0·07 – 0·11)	0·09 (0·07 – 0·11)	0·09 (0·07 – 0·11)	0·09 (0·07 – 0·11)	0·09 (0·07 – 0·11)	-0·14 (-0·6 – 0·3)	3143 (2529 – 3966)	115·9
Jordan	0·22 (0·18 – 0·26)	0·22 (0·19 – 0·26)	0·2 (0·17 – 0·24)	0·21 (0·19 – 0·24)	0·21 (0·19 – 0·24)	0·22 (0·19 – 0·26)	0·07 (-0·22 – 0·4)	1541 (1280 – 1864)	142·31
Kuwait	0·1 (0·08 – 0·12)	0·1 (0·08 – 0·12)	0·1 (0·08 – 0·12)	0·1 (0·08 – 0·12)	0·1 (0·08 – 0·12)	0·1 (0·08 – 0·12)	-0·14 (-0·54 – 0·31)	337 (265 – 429)	89·01
Lebanon	0·08 (0·06 – 0·09)	0·08 (0·06 – 0·09)	0·07 (0·06 – 0·09)	0·08 (0·07 – 0·1)	0·08 (0·07 – 0·09)	0·08 (0·07 – 0·09)	0·21 (-0·2 – 0·53)	453 (385 – 543)	131·35
Libya	0·11 (0·09 – 0·13)	0·11 (0·09 – 0·13)	0·11 (0·09 – 0·13)	0·11 (0·09 – 0·13)	0·11 (0·09 – 0·13)	0·1 (0·09 – 0·13)	-0·13 (-0·54 – 0·32)	597 (487 – 730)	46·46
Morocco	0·04 (0·03 – 0·04)	0·04 (0·04 – 0·05)	0·04 (0·04 – 0·05)	0·04 (0·04 – 0·05)	0·04 (0·04 – 0·05)	0·04 (0·04 – 0·05)	0·46 (0·06 – 0·83)	1366 (1138 – 1655)	71·58
Oman	0·05 (0·04 – 0·06)	0·05 (0·04 – 0·06)	0·05 (0·04 – 0·06)	0·05 (0·04 – 0·06)	0·05 (0·04 – 0·06)	0·05 (0·04 – 0·06)	-0·22 (-0·66 – 0·21)	192 (158 – 239)	143·33
Palestine	0·09 (0·07 – 0·11)	0·09 (0·07 – 0·11)	0·09 (0·07 – 0·11)	0·09 (0·07 – 0·11)	0·09 (0·07 – 0·11)	0·09 (0·07 – 0·11)	0·01 (-0·41 – 0·43)	409 (326 – 516)	153·59
Qatar	0·09 (0·08 – 0·12)	0·09 (0·07 – 0·11)	0·09 (0·08 – 0·12)	0·1 (0·08 – 0·12)	0·1 (0·08 – 0·12)	0·1 (0·08 – 0·12)	0·21 (-0·34 – 0·66)	195 (150 – 256)	400·09
Saudi Arabia	0·08 (0·07 – 0·1)	0·08 (0·07 – 0·1)	0·08 (0·07 – 0·1)	0·08 (0·07 – 0·09)	0·08 (0·07 – 0·09)	0·08 (0·07 – 0·1)	-0·07 (-0·22 – 0·08)	2419 (2021 – 2907)	93·55
Sudan	0·08 (0·07 – 0·1)	0·08 (0·07 – 0·1)	0·08 (0·07 – 0·1)	0·08 (0·06 – 0·1)	0·08 (0·06 – 0·1)	0·08 (0·07 – 0·1)	-0·21 (-0·64 – 0·21)	2820 (2289 – 3530)	97·38
Syria	0·12 (0·1 – 0·14)	0·12 (0·1 – 0·15)	0·12 (0·1 – 0·14)	0·13 (0·1 – 0·15)	0·13 (0·11 – 0·15)	0·13 (0·1 – 0·15)	0·15 (-0·18 – 0·55)	2064 (1709 – 2494)	58·02
Tunisia	0·08 (0·07 – 0·09)	0·08 (0·07 – 0·09)	0·07 (0·06 – 0·08)	0·07 (0·06 – 0·08)	0·08 (0·07 – 0·09)	0·08 (0·07 – 0·09)	-0·02 (-0·35 – 0·37)	875 (730 – 1048)	‥
Turkey	0·06 (0·06 – 0·06)	0·07 (0·07 – 0·07)	0·08 (0·08 – 0·09)	0·11 (0·1 – 0·11)	0·14 (0·14 – 0·15)	0·16 (0·16 – 0·17)	3·64 (3·52 – 3·76)	12 677 (12 149 – 13 180)	302·87
United Arab Emirates	0·09 (0·08 – 0·11)	0·09 (0·08 – 0·11)	0·08 (0·07 – 0·1)	0·09 (0·08 – 0·1)	0·08 (0·07 – 0·1)	0·08 (0·07 – 0·09)	-0·62 (-0·96 to -0·21)	646 (527 – 803)	348·96
Yemen	0·08 (0·07 – 0·1)	0·08 (0·06 – 0·1)	0·08 (0·06 – 0·09)	0·08 (0·06 – 0·09)	0·08 (0·06 – 0·09)	0·08 (0·06 – 0·1)	-0·11 (-0·49 – 0·39)	1951 (1565 – 2464)	136·23
South Asia	0·07 (0·06 – 0·09)	0·06 (0·05 – 0·07)	0·07 (0·06 – 0·09)	0·08 (0·07 – 0·09)	0·06 (0·05 – 0·07)	0·06 (0·06 – 0·07)	-0·67 (-1·14 to -0·23)	93 190 (82 002 – 105 791)	37·62
Bangladesh	0·06 (0·05 – 0·07)	0·05 (0·05 – 0·07)	0·05 (0·04 – 0·06)	0·05 (0·04 – 0·06)	0·06 (0·05 – 0·07)	0·06 (0·05 – 0·07)	0·48 (0·18 – 0·81)	8811 (7426 – 10 839)	97·87
Bhutan	0·07 (0·06 – 0·09)	0·08 (0·06 – 0·09)	0·08 (0·07 – 0·09)	0·08 (0·07 – 0·09)	0·08 (0·07 – 0·09)	0·08 (0·07 – 0·09)	0·22 (-0·08 – 0·51)	55 (47 – 67)	79
India	0·08 (0·06 – 0·1)	0·06 (0·05 – 0·07)	0·08 (0·06 – 0·09)	0·08 (0·08 – 0·09)	0·06 (0·05 – 0·07)	0·06 (0·06 – 0·07)	-0·89 (-1·46 to -0·35)	72 187 (63 774 – 81 022)	26·83
Nepal	0·05 (0·05 – 0·06)	0·06 (0·05 – 0·07)	0·05 (0·05 – 0·06)	0·06 (0·05 – 0·07)	0·07 (0·06 – 0·08)	0·07 (0·06 – 0·08)	0·95 (0·62 – 1·28)	1764 (1495 – 2120)	121·73
Pakistan	0·06 (0·05 – 0·08)	0·07 (0·06 – 0·08)	0·06 (0·05 – 0·07)	0·06 (0·06 – 0·07)	0·06 (0·05 – 0·08)	0·07 (0·05 – 0·08)	0·03 (-0·28 – 0·35)	10 373 (8698 – 12 822)	87·91
Southeast Asia East Asia and Oceania	0·03 (0·03 – 0·04)	0·03 (0·03 – 0·03)	0·04 (0·04 – 0·04)	0·05 (0·04 – 0·05)	0·07 (0·06 – 0·08)	0·11 (0·1 – 0·12)	4·45 (4·32 – 4·61)	222 805 (206 457 – 242 873)	369
East Asia	0·04 (0·03 – 0·04)	0·03 (0·03 – 0·03)	0·04 (0·04 – 0·04)	0·05 (0·05 – 0·06)	0·09 (0·08 – 0·1)	0·14 (0·13 – 0·15)	5·32 (5·18 – 5·49)	204 282 (189 808 – 221 916)	442·96
China	0·03 (0·03 – 0·03)	0·03 (0·03 – 0·03)	0·04 (0·04 – 0·04)	0·05 (0·05 – 0·05)	0·08 (0·08 – 0·09)	0·14 (0·13 – 0·16)	5·67 (5·5 – 5·87)	198 225 (184 244 – 214 994)	497
North Korea	0·13 (0·11 – 0·15)	0·13 (0·11 – 0·15)	0·13 (0·11 – 0·15)	0·13 (0·11 – 0·15)	0·13 (0·11 – 0·15)	0·13 (0·1 – 0·15)	-0·0 (-0·37 – 0·4)	3343 (2792 – 3961)	33·84
Taiwan	0·1 (0·09 – 0·12)	0·1 (0·09 – 0·12)	0·11 (0·09 – 0·13)	0·11 (0·09 – 0·13)	0·11 (0·09 – 0·13)	0·11 (0·09 – 0·13)	0·2 (-0·16 – 0·62)	2715 (2218 – 3246)	41·19
Oceania	0·08 (0·07 – 0·09)	0·08 (0·07 – 0·09)	0·08 (0·06 – 0·09)	0·08 (0·06 – 0·09)	0·08 (0·06 – 0·09)	0·08 (0·06 – 0·09)	-0·09 (-0·33 – 0·18)	725 (611 – 861)	73·9
American Samoa	0·08 (0·07 – 0·09)	0·08 (0·06 – 0·09)	0·08 (0·06 – 0·09)	0·07 (0·06 – 0·09)	0·07 (0·06 – 0·09)	0·07 (0·06 – 0·09)	-0·22 (-0·5 – 0·11)	5 (4 – 6)	‥
Federated States of Micronesia	0·08 (0·06 – 0·09)	0·08 (0·06 – 0·09)	0·08 (0·06 – 0·09)	0·08 (0·06 – 0·09)	0·08 (0·06 – 0·09)	0·08 (0·06 – 0·09)	-0·04 (-0·37 – 0·26)	6 (5 – 8)	5·4
Fiji	0·07 (0·06 – 0·09)	0·07 (0·06 – 0·09)	0·07 (0·06 – 0·08)	0·07 (0·06 – 0·08)	0·07 (0·06 – 0·08)	0·07 (0·06 – 0·08)	-0·16 (-0·44 – 0·13)	55 (46 – 67)	29·35
Guam	0·08 (0·06 – 0·09)	0·08 (0·07 – 0·09)	0·08 (0·06 – 0·09)	0·08 (0·06 – 0·09)	0·08 (0·06 – 0·09)	0·08 (0·06 – 0·09)	-0·08 (-0·38 – 0·24)	13 (11 – 15)	‥
Kiribati	0·08 (0·06 – 0·09)	0·07 (0·06 – 0·09)	0·07 (0·06 – 0·08)	0·07 (0·06 – 0·08)	0·07 (0·06 – 0·08)	0·07 (0·06 – 0·08)	-0·59 (-0·93 to -0·25)	6 (5 – 8)	31·47
Marshall Islands	0·07 (0·06 – 0·09)	0·07 (0·06 – 0·09)	0·07 (0·06 – 0·08)	0·07 (0·06 – 0·09)	0·07 (0·06 – 0·09)	0·07 (0·06 – 0·09)	-0·0 (-0·28 – 0·27)	5 (4 – 5)	71·91
Northern Mariana Islands	0·08 (0·07 – 0·09)	0·08 (0·07 – 0·09)	0·08 (0·06 – 0·09)	0·08 (0·06 – 0·09)	0·08 (0·06 – 0·09)	0·07 (0·06 – 0·09)	-0·18 (-0·5 – 0·11)	8 (6 – 9)	‥
Papua New Guinea	0·08 (0·07 – 0·09)	0·08 (0·07 – 0·09)	0·08 (0·07 – 0·09)	0·08 (0·07 – 0·09)	0·08 (0·07 – 0·09)	0·08 (0·07 – 0·09)	-0·06 (-0·38 – 0·32)	507 (421 – 610)	90·58
Samoa	0·08 (0·06 – 0·09)	0·07 (0·06 – 0·09)	0·07 (0·06 – 0·08)	0·07 (0·06 – 0·08)	0·07 (0·06 – 0·08)	0·07 (0·06 – 0·08)	-0·34 (-0·68 to -0·03)	12 (10 – 15)	16·66
Solomon Islands	0·07 (0·06 – 0·09)	0·07 (0·06 – 0·09)	0·07 (0·06 – 0·08)	0·07 (0·06 – 0·08)	0·07 (0·06 – 0·08)	0·07 (0·06 – 0·08)	-0·33 (-0·64 to -0·06)	33 (28 – 40)	82·93

Tonga	0·08 (0·06 – 0·09)	0·08 (0·06 – 0·09)	0·07 (0·06 – 0·09)	0·07 (0·06 – 0·09)	0·07 (0·06 – 0·09)	0·07 (0·06 – 0·09)	-0·18 (-0·48 – 0·15)	7 (6 – 8)	10
Vanuatu	0·08 (0·07 – 0·09)	0·08 (0·07 – 0·09)	0·08 (0·06 – 0·09)	0·08 (0·06 – 0·09)	0·08 (0·06 – 0·09)	0·08 (0·06 – 0·09)	-0·26 (-0·58 – 0·05)	18 (15 – 22)	76·34
Southeast Asia	0·03 (0·02 – 0·03)	0·03 (0·02 – 0·03)	0·03 (0·03 – 0·03)	0·04 (0·03 – 0·04)	0·03 (0·03 – 0·04)	0·03 (0·02 – 0·04)	0·39 (0·14 – 0·65)	17 798 (14 633 – 21 670)	88·01
Cambodia	0·03 (0·02 – 0·03)	0·03 (0·02 – 0·03)	0·03 (0·02 – 0·03)	0·03 (0·02 – 0·03)	0·03 (0·02 – 0·03)	0·02 (0·02 – 0·03)	-0·53 (-0·85 to -0·24)	316 (259 – 379)	75·2
Indonesia	0·02 (0·02 – 0·03)	0·02 (0·02 – 0·02)	0·03 (0·02 – 0·03)	0·02 (0·02 – 0·02)	0·03 (0·03 – 0·03)	0·03 (0·03 – 0·04)	1·13 (0·48 – 1·8)	7233 (5761 – 9210)	141·01
Laos	0·03 (0·02 – 0·03)	0·03 (0·02 – 0·03)	0·03 (0·02 – 0·03)	0·03 (0·02 – 0·03)	0·02 (0·02 – 0·03)	0·03 (0·02 – 0·03)	-0·32 (-0·61 – 0·01)	143 (118 – 173)	64·05
Malaysia	0·03 (0·03 – 0·04)	0·03 (0·03 – 0·04)	0·04 (0·03 – 0·04)	0·05 (0·04 – 0·06)	0·05 (0·04 – 0·06)	0·03 (0·03 – 0·04)	-0·03 (-0·25 – 0·22)	885 (726 – 1070)	91·85
Maldives	0·03 (0·02 – 0·03)	0·03 (0·02 – 0·04)	0·03 (0·03 – 0·04)	0·03 (0·03 – 0·04)	0·03 (0·03 – 0·04)	0·03 (0·03 – 0·04)	0·33 (0·11 – 0·57)	10 (8 – 12)	131·16
Mauritius	0·03 (0·03 – 0·04)	0·03 (0·03 – 0·04)	0·04 (0·03 – 0·05)	0·04 (0·03 – 0·05)	0·03 (0·03 – 0·04)	0·03 (0·03 – 0·04)	0·1 (-0·15 – 0·35)	42 (34 – 50)	55·25
Myanmar	0·02 (0·02 – 0·03)	0·02 (0·02 – 0·03)	0·02 (0·02 – 0·02)	0·02 (0·02 – 0·02)	0·02 (0·02 – 0·03)	0·02 (0·02 – 0·03)	0·1 (-0·17 – 0·36)	1094 (894 – 1317)	49·31
Philippines	0·03 (0·02 – 0·03)	0·03 (0·02 – 0·03)	0·03 (0·02 – 0·03)	0·03 (0·02 – 0·03)	0·02 (0·02 – 0·03)	0·02 (0·02 – 0·03)	-0·31 (-0·56 to -0·03)	2062 (1712 – 2496)	71·07
Sri Lanka	0·03 (0·03 – 0·04)	0·03 (0·03 – 0·04)	0·04 (0·03 – 0·05)	0·04 (0·03 – 0·05)	0·03 (0·03 – 0·04)	0·03 (0·03 – 0·04)	0·15 (-0·12 – 0·4)	652 (540 – 789)	49·83
Seychelles	0·03 (0·03 – 0·04)	0·03 (0·03 – 0·04)	0·03 (0·03 – 0·04)	0·03 (0·03 – 0·04)	0·03 (0·03 – 0·04)	0·03 (0·03 – 0·04)	-0·15 (-0·39 – 0·07)	3 (3 – 4)	50·61
Thailand	0·03 (0·03 – 0·04)	0·03 (0·03 – 0·04)	0·04 (0·03 – 0·04)	0·04 (0·04 – 0·05)	0·03 (0·03 – 0·04)	0·03 (0·03 – 0·04)	0·21 (-0·04 – 0·47)	2354 (1927 – 2863)	66·53
Timor-Leste	0·03 (0·03 – 0·04)	0·03 (0·03 – 0·04)	0·03 (0·03 – 0·04)	0·03 (0·02 – 0·03)	0·03 (0·02 – 0·03)	0·03 (0·03 – 0·04)	0·02 (-0·24 – 0·24)	29 (25 – 35)	75·66
Vietnam	0·03 (0·03 – 0·04)	0·03 (0·03 – 0·04)	0·04 (0·03 – 0·05)	0·08 (0·07 – 0·08)	0·04 (0·04 – 0·05)	0·03 (0·03 – 0·04)	-0·13 (-0·38 – 0·13)	2947 (2424 – 3540)	56·29
Sub-Saharan Africa	0·07 (0·06 – 0·09)	0·07 (0·06 – 0·09)	0·07 (0·06 – 0·08)	0·07 (0·06 – 0·08)	0·07 (0·06 – 0·08)	0·07 (0·06 – 0·08)	-0·23 (-0·35 to -0·1)	59 928 (51 183 – 71 314)	92·87
Central sub-Saharan Africa	0·08 (0·07 – 0·1)	0·08 (0·07 – 0·09)	0·08 (0·07 – 0·09)	0·08 (0·07 – 0·09)	0·08 (0·07 – 0·09)	0·08 (0·07 – 0·09)	-0·15 (-0·44 – 0·11)	8739 (7353 – 10 424)	116·45
Angola	0·08 (0·07 – 0·1)	0·08 (0·07 – 0·09)	0·08 (0·07 – 0·09)	0·08 (0·07 – 0·09)	0·08 (0·07 – 0·09)	0·08 (0·07 – 0·09)	-0·11 (-0·49 – 0·25)	1908 (1579 – 2307)	125·85
Central African Republic	0·08 (0·07 – 0·1)	0·08 (0·07 – 0·09)	0·08 (0·07 – 0·09)	0·08 (0·07 – 0·1)	0·08 (0·07 – 0·09)	0·08 (0·07 – 0·09)	-0·05 (-0·43 – 0·34)	373 (308 – 453)	70·91
Congo (Brazzaville)	0·09 (0·08 – 0·11)	0·09 (0·08 – 0·11)	0·09 (0·07 – 0·1)	0·09 (0·07 – 0·1)	0·09 (0·07 – 0·1)	0·09 (0·07 – 0·1)	-0·29 (-0·65 – 0·1)	377 (312 – 450)	82·67
Democratic Republic of the Congo	0·08 (0·07 – 0·1)	0·08 (0·07 – 0·09)	0·08 (0·07 – 0·09)	0·08 (0·07 – 0·09)	0·08 (0·07 – 0·09)	0·08 (0·07 – 0·09)	-0·17 (-0·55 – 0·2)	5856 (4895 – 7067)	120·43
Equatorial Guinea	0·09 (0·07 – 0·1)	0·08 (0·07 – 0·1)	0·08 (0·07 – 0·1)	0·08 (0·07 – 0·1)	0·09 (0·07 – 0·1)	0·09 (0·07 – 0·1)	0·07 (-0·29 – 0·45)	68 (57 – 82)	120·19
Gabon	0·09 (0·08 – 0·11)	0·09 (0·08 – 0·11)	0·09 (0·08 – 0·11)	0·09 (0·08 – 0·11)	0·09 (0·08 – 0·11)	0·09 (0·08 – 0·11)	0·18 (-0·18 – 0·58)	157 (130 – 189)	94·71
Eastern sub-Saharan Africa	0·05 (0·05 – 0·06)	0·05 (0·05 – 0·06)	0·05 (0·05 – 0·06)	0·05 (0·04 – 0·06)	0·05 (0·05 – 0·06)	0·05 (0·05 – 0·06)	0·04 (-0·1 – 0·2)	19 206 (16 523 – 22 700)	113·14
Burundi	0·05 (0·05 – 0·07)	0·05 (0·05 – 0·07)	0·06 (0·05 – 0·07)	0·06 (0·05 – 0·07)	0·06 (0·05 – 0·07)	0·06 (0·05 – 0·07)	0·28 (-0·13 – 0·65)	641 (531 – 776)	123·94
Comoros	0·12 (0·1 – 0·15)	0·12 (0·1 – 0·15)	0·11 (0·1 – 0·13)	0·11 (0·1 – 0·13)	0·12 (0·1 – 0·14)	0·12 (0·1 – 0·14)	-0·17 (-0·55 – 0·18)	85 (70 – 103)	84
Djibouti	0·07 (0·05 – 0·08)	0·06 (0·05 – 0·08)	0·06 (0·05 – 0·07)	0·06 (0·05 – 0·07)	0·06 (0·05 – 0·07)	0·06 (0·05 – 0·07)	-0·37 (-0·7 to -0·0)	56 (47 – 67)	52·23
Eritrea	0·06 (0·05 – 0·07)	0·06 (0·05 – 0·07)	0·06 (0·05 – 0·07)	0·06 (0·05 – 0·07)	0·06 (0·05 – 0·07)	0·06 (0·05 – 0·07)	-0·12 (-0·49 – 0·22)	279 (229 – 341)	65·55
Ethiopia	0·02 (0·02 – 0·03)	0·03 (0·02 – 0·03)	0·02 (0·02 – 0·03)	0·03 (0·02 – 0·03)	0·03 (0·03 – 0·04)	0·03 (0·03 – 0·04)	0·91 (0·55 – 1·31)	2895 (2351 – 3536)	173·67
Kenya	0·05 (0·04 – 0·06)	0·05 (0·04 – 0·06)	0·06 (0·05 – 0·07)	0·04 (0·03 – 0·04)	0·04 (0·03 – 0·04)	0·05 (0·04 – 0·05)	-0·28 (-0·38 to -0·17)	1988 (1680 – 2393)	86·79
Madagascar	0·05 (0·05 – 0·07)	0·05 (0·05 – 0·07)	0·05 (0·04 – 0·06)	0·05 (0·04 – 0·06)	0·05 (0·04 – 0·06)	0·05 (0·04 – 0·06)	-0·31 (-0·67 – 0·02)	1176 (972 – 1430)	96·63
Malawi	0·07 (0·06 – 0·09)	0·07 (0·06 – 0·09)	0·08 (0·06 – 0·09)	0·07 (0·06 – 0·08)	0·05 (0·05 – 0·06)	0·06 (0·05 – 0·07)	-0·83 (-1·45 to -0·32)	941 (803 – 1126)	50·87
Mozambique	0·06 (0·05 – 0·07)	0·05 (0·05 – 0·07)	0·05 (0·05 – 0·07)	0·05 (0·05 – 0·06)	0·05 (0·05 – 0·06)	0·05 (0·05 – 0·07)	-0·13 (-0·5 – 0·32)	1433 (1177 – 1744)	111·38
Rwanda	0·03 (0·02 – 0·03)	0·03 (0·02 – 0·03)	0·03 (0·02 – 0·03)	0·03 (0·02 – 0·03)	0·03 (0·02 – 0·03)	0·03 (0·02 – 0·03)	0·15 (-0·32 – 0·53)	308 (249 – 375)	72·58
Somalia	0·05 (0·05 – 0·07)	0·05 (0·05 – 0·06)	0·05 (0·04 – 0·06)	0·05 (0·05 – 0·06)	0·05 (0·05 – 0·07)	0·05 (0·05 – 0·07)	-0·09 (-0·47 – 0·26)	504 (415 – 617)	61·99
South Sudan	0·06 (0·05 – 0·07)	0·06 (0·05 – 0·07)	0·06 (0·05 – 0·07)	0·06 (0·05 – 0·07)	0·06 (0·05 – 0·07)	0·06 (0·05 – 0·07)	-0·11 (-0·46 – 0·2)	771 (638 – 951)	119·43
Tanzania	0·09 (0·08 – 0·11)	0·09 (0·08 – 0·1)	0·1 (0·09 – 0·12)	0·1 (0·09 – 0·12)	0·11 (0·1 – 0·12)	0·1 (0·08 – 0·12)	0·41 (-0·03 – 0·84)	5099 (4224 – 6159)	148·39
Uganda	0·06 (0·05 – 0·07)	0·06 (0·05 – 0·07)	0·06 (0·05 – 0·07)	0·05 (0·04 – 0·05)	0·05 (0·04 – 0·05)	0·05 (0·05 – 0·05)	-0·74 (-1·27 to -0·21)	1942 (1731 – 2200)	94·69
Zambia	0·07 (0·06 – 0·09)	0·07 (0·06 – 0·09)	0·06 (0·06 – 0·07)	0·06 (0·05 – 0·06)	0·06 (0·06 – 0·06)	0·07 (0·06 – 0·08)	-0·2 (-0·53 – 0·14)	1075 (897 – 1305)	93·65
Southern sub-Saharan Africa	0·12 (0·09 – 0·16)	0·12 (0·09 – 0·16)	0·13 (0·1 – 0·16)	0·13 (0·1 – 0·16)	0·12 (0·1 – 0·15)	0·13 (0·1 – 0·16)	0·21 (-0·1 – 0·54)	8950 (6930 – 11 606)	66·2

Botswana	0·08 (0·07 – 0·09)	0·08 (0·07 – 0·09)	0·08 (0·07 – 0·1)	0·08 (0·07 – 0·1)	0·08 (0·07 – 0·1)	0·08 (0·07 – 0·1)	0·07 (-0·27 – 0·44)	164 (137 – 198)	87·33
Lesotho	0·08 (0·06 – 0·09)	0·08 (0·06 – 0·09)	0·08 (0·06 – 0·09)	0·08 (0·06 – 0·09)	0·08 (0·06 – 0·09)	0·08 (0·06 – 0·09)	-0·02 (-0·37 – 0·31)	139 (114 – 169)	36·81
Namibia	0·1 (0·09 – 0·12)	0·1 (0·09 – 0·12)	0·09 (0·08 – 0·1)	0·09 (0·08 – 0·1)	0·1 (0·09 – 0·12)	0·1 (0·09 – 0·12)	-0·03 (-0·37 – 0·35)	222 (186 – 267)	81·63
South Africa	0·14 (0·1 – 0·19)	0·14 (0·1 – 0·19)	0·15 (0·12 – 0·2)	0·15 (0·12 – 0·19)	0·14 (0·11 – 0·17)	0·15 (0·11 – 0·2)	0·29 (-0·09 – 0·7)	7445 (5626 – 9863)	66·06
Swaziland (eSwatini)	0·04 (0·03 – 0·05)	0·04 (0·03 – 0·05)	0·04 (0·03 – 0·04)	0·04 (0·03 – 0·04)	0·04 (0·03 – 0·05)	0·04 (0·03 – 0·05)	-0·05 (-0·38 – 0·25)	47 (39 – 57)	65·23
Zimbabwe	0·07 (0·06 – 0·08)	0·07 (0·06 – 0·08)	0·06 (0·05 – 0·07)	0·06 (0·06 – 0·07)	0·07 (0·06 – 0·08)	0·07 (0·06 – 0·08)	0·2 (-0·16 – 0·52)	933 (775 – 1135)	66·01
Western sub-Saharan Africa	0·08 (0·07 – 0·09)	0·08 (0·06 – 0·09)	0·07 (0·06 – 0·08)	0·07 (0·06 – 0·08)	0·07 (0·06 – 0·08)	0·07 (0·06 – 0·08)	-0·4 (-0·61 to -0·19)	23 033 (19 701 – 27 438)	82·25
Benin	0·06 (0·05 – 0·07)	0·06 (0·05 – 0·07)	0·06 (0·05 – 0·07)	0·06 (0·05 – 0·07)	0·06 (0·05 – 0·07)	0·06 (0·05 – 0·07)	-0·09 (-0·39 – 0·23)	570 (476 – 695)	125·24
Burkina Faso	0·07 (0·06 – 0·08)	0·07 (0·06 – 0·08)	0·06 (0·06 – 0·07)	0·06 (0·05 – 0·07)	0·07 (0·06 – 0·08)	0·07 (0·06 – 0·08)	-0·03 (-0·36 – 0·33)	1014 (833 – 1227)	107·25
Cameroon	0·08 (0·07 – 0·09)	0·08 (0·07 – 0·09)	0·08 (0·06 – 0·09)	0·07 (0·06 – 0·09)	0·07 (0·06 – 0·09)	0·07 (0·06 – 0·09)	-0·3 (-0·65 to -0·01)	1454 (1214 – 1745)	86·21
Cape Verde	0·08 (0·06 – 0·09)	0·07 (0·06 – 0·09)	0·08 (0·06 – 0·09)	0·08 (0·06 – 0·09)	0·08 (0·07 – 0·09)	0·08 (0·07 – 0·09)	0·09 (-0·22 – 0·44)	38 (32 – 46)	76·61
Chad	0·06 (0·05 – 0·07)	0·06 (0·05 – 0·07)	0·05 (0·04 – 0·06)	0·05 (0·04 – 0·06)	0·06 (0·05 – 0·07)	0·06 (0·05 – 0·07)	-0·14 (-0·41 – 0·21)	665 (548 – 820)	130·63
Cote d'Ivoire	0·08 (0·07 – 0·09)	0·08 (0·06 – 0·09)	0·07 (0·06 – 0·08)	0·07 (0·06 – 0·08)	0·07 (0·06 – 0·09)	0·07 (0·06 – 0·09)	-0·15 (-0·54 – 0·19)	1435 (1204 – 1709)	84·62
The Gambia	0·07 (0·06 – 0·08)	0·07 (0·06 – 0·08)	0·07 (0·06 – 0·08)	0·07 (0·06 – 0·08)	0·07 (0·06 – 0·08)	0·07 (0·06 – 0·08)	-0·04 (-0·35 – 0·26)	114 (95 – 140)	115·6
Ghana	0·05 (0·04 – 0·06)	0·05 (0·04 – 0·05)	0·05 (0·05 – 0·06)	0·05 (0·05 – 0·06)	0·05 (0·04 – 0·05)	0·04 (0·04 – 0·05)	-0·49 (-0·79 to -0·15)	1031 (892 – 1219)	73·3
Guinea	0·07 (0·05 – 0·08)	0·06 (0·05 – 0·08)	0·06 (0·05 – 0·08)	0·06 (0·05 – 0·07)	0·06 (0·05 – 0·07)	0·06 (0·05 – 0·07)	-0·2 (-0·52 – 0·1)	666 (555 – 797)	105·56
Guinea-Bissau	0·07 (0·06 – 0·09)	0·07 (0·06 – 0·09)	0·07 (0·06 – 0·08)	0·07 (0·06 – 0·08)	0·07 (0·06 – 0·08)	0·07 (0·06 – 0·08)	-0·4 (-0·69 to -0·12)	107 (89 – 131)	64·28
Liberia	0·07 (0·06 – 0·08)	0·07 (0·06 – 0·08)	0·07 (0·06 – 0·08)	0·07 (0·06 – 0·08)	0·07 (0·06 – 0·08)	0·07 (0·06 – 0·08)	-0·07 (-0·43 – 0·31)	258 (215 – 315)	113·47
Mali	0·05 (0·04 – 0·06)	0·05 (0·04 – 0·06)	0·04 (0·04 – 0·05)	0·04 (0·04 – 0·05)	0·05 (0·04 – 0·06)	0·05 (0·05 – 0·06)	-0·04 (-0·62 – 0·5)	685 (624 – 758)	89·43
Mauritania	0·08 (0·07 – 0·1)	0·08 (0·07 – 0·1)	0·07 (0·06 – 0·08)	0·07 (0·06 – 0·08)	0·08 (0·07 – 0·09)	0·08 (0·07 – 0·09)	-0·16 (-0·53 – 0·25)	270 (224 – 327)	102·23
Niger	0·07 (0·06 – 0·08)	0·06 (0·05 – 0·07)	0·06 (0·05 – 0·07)	0·07 (0·06 – 0·08)	0·06 (0·05 – 0·07)	0·07 (0·06 – 0·08)	-0·08 (-0·44 – 0·28)	1126 (942 – 1383)	149·04
Nigeria	0·09 (0·08 – 0·11)	0·09 (0·08 – 0·11)	0·08 (0·06 – 0·09)	0·08 (0·07 – 0·09)	0·08 (0·07 – 0·09)	0·08 (0·07 – 0·1)	-0·51 (-0·87 to -0·15)	12 160 (10 149 – 14 678)	70·72
Sao Tome and Principe	0·09 (0·07 – 0·1)	0·08 (0·07 – 0·1)	0·08 (0·07 – 0·1)	0·08 (0·07 – 0·1)	0·08 (0·07 – 0·1)	0·08 (0·07 – 0·1)	-0·17 (-0·51 – 0·15)	14 (11 – 16)	66·19
Senegal	0·06 (0·05 – 0·07)	0·05 (0·05 – 0·06)	0·05 (0·04 – 0·05)	0·05 (0·04 – 0·05)	0·05 (0·04 – 0·06)	0·05 (0·04 – 0·06)	-0·35 (-0·65 – 0·03)	664 (554 – 808)	93·34
Sierra Leone	0·07 (0·06 – 0·08)	0·07 (0·06 – 0·08)	0·06 (0·05 – 0·08)	0·06 (0·05 – 0·08)	0·06 (0·05 – 0·08)	0·06 (0·05 – 0·07)	-0·39 (-0·71 to -0·08)	345 (288 – 418)	53·23
Togo	0·07 (0·06 – 0·09)	0·07 (0·06 – 0·09)	0·07 (0·06 – 0·08)	0·07 (0·06 – 0·08)	0·07 (0·06 – 0·08)	0·07 (0·06 – 0·08)	-0·25 (-0·59 – 0·09)	418 (348 – 504)	86·91

Section 6. Unit cost estimates

6.1. Definition of unit cost

We estimated unit costs as spending per capita on each service divided by utilisation per capita as shown in equation (7):

$$Unit\ cost_y = \frac{THE\ per\ capita \cdot \widehat{share}_y}{\widehat{utilisation\ rate}_y} \quad (7)$$

where y denotes either outpatient visit or inpatient admission. Spending per capita was the product of total health expenditures (THE) per capita in 2016 international dollars, and the share of each service in THE. The shares were estimated with NHA data, which was a mutually exclusive and collectively exhaustive account THE through a health system.

6.2. Data sources

The NHA tracked spending in the health sector using the System of Health Accounts framework.[15] Bui et al did a systematic review to identify and publish data from 872 reports from 1996 to 2010.[16] The GBD Health Financing Collaborator Network updated the review in 2016 and located 178 additional NHAs for a total of 1050.[17–19] We searched ministry of health websites and collected a total of 1285 country-years of NHA data, including some accounts that reported data for several years.

The NHA functional categories have five "one-digit" level categories for personal health-care services and goods, two for collective health-care services, and seven for health-related functions. Each of the one-digit level categories can be reported in more detail at the "two-digit" level, where the two-digit levels for the curative and rehabilitative care categories distinguish outpatient (HC 1.3 and HC 2.3, respectively) and inpatient services (HC 1.1 and HC 2.1, respectively). We included only NHAs that reported curative and rehabilitative services at the two-digit level, which limited the sample to 795 country-years of NHA data.

Half (391 of 795) of the NHAs that met the inclusion criteria were from the High-Income super-region, and 155 (20%) were from Central Europe, Eastern Europe, and Central Asia (Table S12). The most recent NHA was in 2015. Among countries in the High Income super-region, 79% had at least one NHA between 1990 and 2016 that met the inclusion criteria. Among countries in the sub-Saharan Africa super-region, 61% had at least one NHA. In three super-regions, fewer than half of the countries had at least one NHA: Southeast Asia, East Asia, and Oceana (44%), Latin America and Caribbean (34%), and North Africa and the Middle East (29%).

Our definition of outpatient visits per capita included facility-based preventive maternal and child care (HC 6.4), and vaccinations (HC 6.2). Expenditures on these categories were not, however, included in the expenditure shares. Only 146 of 795 NHAs reported these expenditures, which would have limited the sample size as well as the percentage of countries represented. The absence of these expenditures may have underestimated the unit cost of outpatient visits, and was reported in the main text. Among NHAs that reported these expenditures, they accounted for less than 1% of THE in 101 (69%) country-years, which were predominately from high income countries, and more than 3% of THE in 16 (11%) country-years, which were predominately from low and lower-middle income countries.

We did not produce estimates of the shares of day-patient admissions (HC1.2 and HC2.2) and long-term care (HC 3), because data on utilisation and expenditures were not available globally. Only 120 of 795 NHAs reported expenditures on day-patient admissions. Although 720 of the NHAs reported expenditures on long-term care, utilisation data were not systematically reported in the administrative data sources and rarely reported in the survey data sources.

Table S12. Description of the National Health Account sample by GBD super-region and time period, and number of countries represented by GBD super-region

Count of countries by GBD super-region reflects the countries where total health expenditure (THE) per capita values was estimated. Four countries' NHAs didn't report information on the share of THE spent on inpatient services, but did report on share of THE spent on outpatient services. Count of NHAs include these four NHAs. GBD = Global Burden of Disease. NHA = National Health Accounts. THE=Total Health Expenditure.

GBD super-region	Time periods					Total number of NHAs across time periods	Number of Countries in GBD super-region	Number (percentage) of countries with at least one NHA
	1990-1994	1995-1999	2000-2004	2005-2009	2010-2016			
**Central Europe, Eastern Europe, and Central Asia**	0	8	37	59	51	155	29	20 (69%)
**High-income**	0	78	97	104	112	391	33	26 (79%)
**Latin America and Caribbean**	0	12	7	6	8	33	29	10 (34%)
**North Africa and Middle East**	0	1	7	6	8	22	21	6 (29%)
**South Asia**	0	3	5	8	9	25	5	4 (80%)
**Southeast Asia, East Asia, and Oceania**	5	14	20	27	18	84	25	11 (44%)
**Sub-Saharan Africa**	0	1	17	31	36	85	46	28 (61%)
**Total**	5	117	190	241	242	795	188	105 (56%)

6.3. Estimates of expenditures shares

We developed three statistical models to mutually exclusively and collectively exhaustively estimate the share of THE spent on: outpatient services, inpatient services, and all other functions in 184 countries covering 22 years. We estimated the three logit transformed shares of THE using a linear regression model with lag distributed income as the independent variable, where income refers to gross domestic product per capita. Dependent variables were logit transformed to bound predictions between zero and one. The estimated coefficient (standard error) for lag distributed income was 0.184 (0.023) for the share of outpatient expenditures, 0.094 (0.024) for inpatient, and -0.176 (0.029) for all other functions. We used the results of the linear regression model and lag-distributed income to predict the shares for each country-year, and rescaled the estimates to ensure they summed to one.

We also estimated the shares using the ST-GPR method described in Section 4.3 above, where the linear regression results were the first of three steps. To choose between the two methods, we compared the out-of-sample root mean squared error, and the in-sample coverage (Table S13). To generate the out-of-sample root mean square error, we performed a ten-fold cross-validation procedure, in which we portioned our data by country so that the trained model would be evaluated on data from countries that weren't used to estimate the model. In-sample coverage represents the percentage of data points falling within the 95% uncertainty interval. We reported estimates based on

the linear regression model, because the out-of-sample root mean squared error of the two models were almost the same, and in-sample coverage of the linear regression model was higher, reflecting larger uncertainty intervals.

Table S13. Regression model fit statistics.

Root mean square errors were calculated in level space. In-sample coverage is the percentage of data points falling within the 95% uncertainty interval.

Share	Outpatient	Inpatient	All other functions
**Out-of-sample root mean square error**			
**Linear regression**	0.07917	0.08874	0.1273
**Spatio-temporal Gaussian Process Regression**	0.07912	0.08850	0.1284
**In-sample coverage**			
**Linear regression**	95 %	94%	94%
**Spatio-temporal Gaussian Process Regression**	45%	43%	69%

We propagated uncertainty by calculating 1,000 draws for each model at the country-year level. To ensure the estimated shares were collectively exhaustive of THE, at the country-year-draw level, the three shares were rescaled to ensure they summed to one.

6.4. Comparison to WHO-CHOICE estimates

We compared our unit cost estimates to the widely-referenced estimates by researchers on the World Health Organization Choosing Interventions that are Cost Effective (WHO-CHOICE) project. The WHO-CHOICE researchers were the first to produce global estimates of the unit cost of outpatient visits and inpatient bed-days.[20] The estimates were updated to report unit costs for 2007 and 2008 in three currencies: local currency, United States dollars, and international dollars.[21]

We compared our (IHME) 2008 estimates to the WHO-CHOICE estimates of outpatient visits for secondary hospitals and inpatient bed-days for teaching hospitals. WHO-CHOICE estimated costs for three levels of facilities for each service, and we based our comparison on the level with the highest unit costs. To facilitate comparison between the estimates we made two conversions. First, we converted WHO-CHOICE estimates of cost per inpatient per-bed to cost per admission by multiplying each estimate by 9.75, which was the average length of stay secondary and teaching hospitals for every country in the WHO-CHOICE prediction equations. Second, our 2008 unit cost estimates were measured in 2017 international dollars while WHO-CHOICE's 2008 unit cost estimates were measured in 2010 international dollars. We deflated our estimates measured in 2017 dollars to 2010 dollars using GDP deflators provided by the International Monetary Fund.[22]

We ran least squares regressions of our estimates on the WHO-CHOICE estimates and restricted the intercept to equal zero. The slope of this regression for outpatient cost was 2.0267 meaning that our outpatient cost estimates were, on average, 102.67% higher than WHO-CHOICE. Similarly, the slope of the inpatient regression on unit cost was 1.0264 meaning that our estimates were, on average, 2.64% higher than WHO-CHOICE.

6.5. Comparison to other comprehensive, national studies of the unit cost of services

To compare our results to other comprehensive, national cost estimates, we conducted a literature search on Medline with the following strategies: 1) MeSH terms (Direct Service Cost or Hospital Cost) and ("cost per outpatient visit"

OR “cost per inpatient admission”), and 2) MeSH terms (Direct Service Cost or Hospital Cost) and “national costing study” OR “country costing study.” The first strategy resulted in 309 articles, of which 265 were disease-specific, 27 did not report either inpatient or outpatient cost estimates, 16 were not nationally representative, and one reported data before 1990. (Exclusion criteria were applied sequentially.) The second strategy resulted in 55 articles, of which 41 were disease-specific, 12 were not nationally representative, one didn’t address representativeness, and one did not report either inpatient or outpatient cost estimates.

We also searched for national costing studies reported by national governments and other organisations, and identified studies in the United States and Australia that reported comprehensive unit costs at the national level, as reported in the Discussion section of the main text. NHS Improvement reported the reference cost of a hospital admission in the United Kingdom, but excluded services such as intensive care, chemotherapy, and other high cost drugs.[23] The Canadian Institute of Health Information reported the average cost per resource intensity weight for every location except Quebec and Nunavut.[24] The total number of admissions would be similar, but not the same as the total weighted cases, because the average resource intensity weight is not one.

6.6. Country-specific parameters for the calculation of 2016 unit costs

Table S14. National estimates of share of total health expenditure on outpatient and inpatient services, utilisation rates, and unit cost in 2016 in 2017 international dollars

Table displays three sets of national estimates organised by GBD region: 1) THE per capita measured in 2017 international dollars,[25,26] and the estimated share of outpatient visits and inpatient admissions in the national health accounts, 2) utilisation per capita for each service, and 3) cost per outpatient visit and inpatient admission calculated as the ratio of expenditure (THE per capita * share) to utilisation per capita. For each result, the mean of 1,000 draws is reported, and in parenthesis the uncertainty interval defined as the 2.5th and 97.5th percentile of draws. The exchange rate for 2017 international dollars to United Sates dollars is reported to facilitate conversions. GBD= Global Burden of Disease. THE=Total Health Expenditure.

Table S14: National estimates of share of total health expenditure on outpatient and inpatient services, utilisation rates, and unit cost in 2016 in 2017 international dollars

	Exchange rate 2017 PPP to 2017 USD	Total health expenditure per capita measured in 2017 PPP (95% UI)	Outpatient share (95% UI)	Outpatient utilization per capita (95% UI)	Outpatient unit cost measured in 2017 PPP (95% UI)	Inpatient share (95% UI)	Inpatient utilization per capita (95% UI)	Inpatient unit cost measured in 2017 PPP (95% UI)
Global	··	··	··	5·32 (4·79 – 5·9)	··	··	0·1 (0·09 – 0·1)	··
Central Europe, Eastern Europe, and Central Asia	··	··	··	9·04 (8·66 – 9·41)	··	··	0·2 (0·19 – 0·21)	··
Central Asia	··	··	··	6·68 (6·46 – 6·91)	··	··	0·14 (0·14 – 0·15)	··
Armenia	0·4	865 (733 – 1008)	21·8 (20·7 – 22·9)	3·9 (3·77 – 4·03)	48 (40 – 57)	26·0 (24·7 – 27·3)	0·13 (0·13 – 0·14)	1710 (1426 – 2019)
Azerbaijan	0·22	1200 (1059 – 1341)	24·0 (22·9 – 25·1)	4·67 (4·5 – 4·85)	62 (54 – 70)	27·2 (26·1 – 28·5)	0·07 (0·06 – 0·07)	4938 (4342 – 5613)
Georgia	0·36	859 (772 – 953)	22·0 (20·9 – 23·1)	6·06 (5·24 – 7·16)	31 (26 – 38)	26·1 (24·9 – 27·4)	0·09 (0·09 – 0·1)	2408 (2128 – 2720)
Kazakhstan	0·31	1040 (965 – 1115)	25·0 (23·9 – 26·2)	6·67 (6·45 – 6·91)	39 (35 – 43)	27·8 (26·6 – 29·1)	0·15 (0·15 – 0·16)	1872 (1699 – 2046)
Kyrgyzstan	0·3	322 (298 – 355)	19·1 (17·8 – 20·5)	3·48 (3·38 – 3·58)	18 (16 – 20)	24·4 (22·8 – 26·1)	0·15 (0·14 – 0·16)	525 (472 – 595)
Mongolia	0·28	515 (467 – 564)	22·6 (21·6 – 23·7)	5·34 (4·55 – 6·34)	22 (18 – 26)	26·4 (25·3 – 27·7)	0·15 (0·13 – 0·18)	907 (717 – 1126)
Tajikistan	0·25	212 (198 – 230)	18·5 (17·2 – 20·0)	4·55 (4·38 – 4·72)	9 (8 – 10)	24·0 (22·3 – 25·8)	0·12 (0·12 – 0·13)	410 (368 – 459)
Turkmenistan	0·39	1165 (1043 – 1312)	23·8 (22·7 – 24·9)	3·55 (3·42 – 3·68)	78 (68 – 89)	27·1 (26·0 – 28·3)	0·15 (0·12 – 0·18)	2182 (1706 – 2668)
Uzbekistan	0·2	471 (437 – 506)	20·9 (19·8 – 22·1)	9·62 (9·34 – 9·92)	10 (9 – 11)	25·5 (24·2 – 26·9)	0·17 (0·16 – 0·17)	717 (648 – 790)
Central Europe	··	··	··	7·95 (7·66 – 8·23)	··	··	0·19 (0·18 – 0·21)	··
Albania	0·35	891 (823 – 977)	22·6 (21·5 – 23·6)	5·56 (4·72 – 6·49)	36 (30 – 44)	26·4 (25·3 – 27·6)	0·09 (0·09 – 0·1)	2546 (2275 – 2879)
Bosnia and Herzegovina	0·39	1115 (1040 – 1202)	22·4 (21·4 – 23·5)	10·15 (8·98 – 11·85)	25 (21 – 29)	26·3 (25·2 – 27·6)	0·12 (0·1 – 0·14)	2550 (2104 – 3087)
Bulgaria	0·37	1707 (1641 – 1779)	24·1 (23·0 – 25·2)	6·05 (5·68 – 6·41)	68 (63 – 74)	27·3 (26·1 – 28·5)	0·29 (0·28 – 0·31)	1592 (1460 – 1728)
Croatia	0·52	1812 (1680 – 1971)	24·8 (23·6 – 25·9)	6·5 (6·28 – 6·73)	69 (63 – 76)	27·6 (26·5 – 28·9)	0·18 (0·17 – 0·19)	2753 (2489 – 3067)
Czech Republic	0·54	2656 (2537 – 2779)	25·9 (24·6 – 27·2)	10·8 (10·4 – 11·27)	64 (59 – 69)	28·3 (26·9 – 29·6)	0·25 (0·22 – 0·29)	3035 (2546 – 3547)
Hungary	0·49	2085 (1978 – 2194)	25·2 (24·0 – 26·4)	12·71 (12·3 – 13·11)	42 (38 – 45)	27·9 (26·7 – 29·2)	0·21 (0·2 – 0·22)	2764 (2528 – 3013)
Macedonia	0·35	950 (834 – 1105)	23·2 (22·2 – 24·3)	6·74 (6·46 – 7·02)	33 (28 – 38)	26·8 (25·7 – 28·0)	0·12 (0·11 – 0·13)	2081 (1800 – 2521)
Montenegro	0·41	1015 (957 – 1073)	23·6 (22·6 – 24·7)	7·27 (6·87 – 7·68)	33 (30 – 36)	27·0 (25·9 – 28·3)	0·12 (0·12 – 0·13)	2264 (2067 – 2469)
Poland	0·46	1805 (1731 – 1902)	25·2 (24·0 – 26·4)	7·4 (7·15 – 7·66)	62 (57 – 67)	27·9 (26·7 – 29·2)	0·17 (0·16 – 0·18)	2940 (2679 – 3219)
Romania	0·41	1200 (1111 – 1297)	24·3 (23·1 – 25·3)	4·99 (4·8 – 5·17)	58 (53 – 65)	27·4 (26·2 – 28·6)	0·24 (0·22 – 0·26)	1393 (1231 – 1568)
Serbia	0·37	1469 (1352 – 1591)	23·3 (22·2 – 24·3)	7·77 (7·51 – 8·01)	44 (40 – 48)	26·8 (25·7 – 28·0)	0·12 (0·12 – 0·13)	3155 (2844 – 3495)
Slovakia	0·54	2309 (2152 – 2477)	25·6 (24·4 – 26·9)	12·6 (12·18 – 13·0)	47 (43 – 52)	28·1 (26·8 – 29·5)	0·21 (0·19 – 0·22)	3164 (2865 – 3499)
Slovenia	0·67	2876 (2770 – 2992)	25·9 (24·6 – 27·1)	7·47 (7·25 – 7·69)	100 (93 – 107)	28·2 (26·9 – 29·6)	0·19 (0·18 – 0·2)	4347 (3986 – 4707)
Eastern Europe	··	··	··	10·61 (10·09 – 11·14)	··	··	0·23 (0·22 – 0·24)	··
Belarus	0·27	1239 (1179 – 1304)	24·2 (23·1 – 25·3)	12·84 (12·48 – 13·18)	23 (22 – 25)	27·4 (26·2 – 28·6)	0·25 (0·21 – 0·3)	1348 (1122 – 1629)
Estonia	0·61	2027 (1895 – 2156)	25·5 (24·3 – 26·7)	7·83 (7·62 – 8·04)	66 (61 – 72)	28·0 (26·8 – 29·4)	0·19 (0·18 – 0·19)	3052 (2793 – 3329)
Latvia	0·56	1802 (1674 – 1934)	24·9 (23·8 – 26·1)	7·21 (6·92 – 7·5)	62 (57 – 69)	27·7 (26·6 – 29·1)	0·19 (0·19 – 0·2)	2578 (2346 – 2813)
Lithuania	0·51	2005 (1906 – 2107)	25·4 (24·2 – 26·6)	9·68 (9·41 – 9·97)	53 (49 – 56)	28·0 (26·8 – 29·3)	0·26 (0·24 – 0·27)	2186 (1984 – 2401)
Moldova	0·37	568 (519 – 623)	20·1 (18·9 – 21·4)	6·63 (6·42 – 6·84)	17 (15 – 19)	25·0 (23·5 – 26·5)	0·17 (0·16 – 0·17)	855 (760 – 966)
Russia	0·37	1581 (1518 – 1654)	25·1 (23·9 – 26·3)	12·02 (11·48 – 12·55)	33 (31 – 36)	27·8 (26·7 – 29·1)	0·23 (0·22 – 0·24)	1941 (1808 – 2089)
Ukraine	0·25	619 (576 – 663)	22·1 (21·0 – 23·1)	6·3 (5·49 – 7·49)	22 (18 – 26)	26·1 (24·9 – 27·4)	0·23 (0·22 – 0·25)	694 (626 – 768)
High-income	··	··	··	8·63 (7·91 – 9·43)	··	··	0·14 (0·13 – 0·15)	··
Australasia	··	··	··	8·41 (8·13 – 8·67)	··	··	0·17 (0·15 – 0·18)	··

Australia	1·1	4510 (4430 – 4603)	29·1 (28·3 – 29·9)	8·49 (8·24 – 8·74)	154 (148 – 162)	27·6 (26·8 – 28·4)	0·17 (0·15 – 0·19)	7334 (6669 – 8087)
New Zealand	1·02	3717 (3469 – 3967)	28·3 (27·5 – 29·1)	7·97 (7·44 – 8·43)	132 (120 – 146)	27·2 (26·4 – 28·0)	0·16 (0·15 – 0·16)	6479 (5973 – 7007)
High-income Asia-Pacific	‥	‥	‥	16·41 (14·77 – 17·83)	‥	‥	0·13 (0·11 – 0·15)	‥
Brunei	0·4	2037 (1792 – 2284)	30·9 (29·9 – 31·9)	9·31 (7·95 – 11·69)	68 (54 – 83)	28·4 (27·4 – 29·4)	0·11 (0·09 – 0·13)	5260 (4199 – 6551)
Japan	0·89	4350 (4175 – 4542)	28·5 (27·7 – 29·3)	16·38 (14·21 – 18·21)	76 (67 – 88)	27·3 (26·5 – 28·1)	0·12 (0·1 – 0·14)	10 335 (8572 – 12 472)
Singapore	0·6	3715 (3396 – 4049)	31·0 (30·0 – 32·0)	14·56 (12·43 – 17·73)	79 (65 – 95)	28·5 (27·5 – 29·5)	0·12 (0·1 – 0·14)	9132 (7516 – 11 044)
South Korea	0·75	2959 (2841 – 3078)	28·3 (27·5 – 29·1)	16·7 (16·13 – 17·23)	50 (47 – 53)	27·2 (26·5 – 28·0)	0·17 (0·16 – 0·18)	4878 (4492 – 5261)
High-income North America	‥	‥	‥	6·42 (5·69 – 7·2)	‥	‥	0·12 (0·11 – 0·13)	‥
Canada	0·93	5012 (4884 – 5133)	29·1 (28·3 – 29·8)	9·28 (8·82 – 9·71)	157 (148 – 167)	27·6 (26·8 – 28·4)	0·14 (0·11 – 0·16)	10 103 (8448 – 12 088)
Greenland	1·19	‥	‥	7·28 (6·3 – 8·56)	‥	‥	0·12 (0·1 – 0·15)	‥
United States	1	9793 (9572 – 10 055)	29·7 (28·9 – 30·6)	6·1 (5·3 – 6·93)	478 (415 – 548)	27·9 (27·0 – 28·7)	0·12 (0·11 – 0·13)	22 543 (20 547 – 24 392)
Southern Latin America	‥	‥	‥	4·4 (3·94 – 4·97)	‥	‥	0·09 (0·08 – 0·1)	‥
Argentina	0·57	1452 (1348 – 1567)	26·5 (25·7 – 27·4)	4·52 (3·9 – 5·32)	86 (71 – 101)	26·4 (25·5 – 27·3)	0·09 (0·08 – 0·11)	4211 (3507 – 5052)
Chile	0·59	1980 (1915 – 2039)	26·7 (25·9 – 27·5)	4·04 (3·95 – 4·13)	131 (125 – 138)	26·4 (25·6 – 27·4)	0·09 (0·08 – 0·1)	5956 (5252 – 6721)
Uruguay	0·7	2099 (1956 – 2247)	26·4 (25·5 – 27·3)	4·71 (4·1 – 5·45)	118 (100 – 139)	26·3 (25·4 – 27·3)	0·06 (0·05 – 0·07)	9102 (7505 – 10 776)
Western Europe	‥	‥	‥	7·87 (7·28 – 8·56)	‥	‥	0·17 (0·16 – 0·18)	‥
Andorra	0·56	9155 (8439 – 9997)	30·5 (29·6 – 31·4)	7·63 (6·67 – 8·73)	367 (311 – 429)	28·2 (27·3 – 29·2)	0·17 (0·14 – 0·21)	15 054 (12 308 – 18 392)
Austria	0·91	5238 (5144 – 5342)	29·3 (28·4 – 30·1)	8·19 (7·95 – 8·45)	187 (179 – 196)	27·7 (26·9 – 28·5)	0·27 (0·26 – 0·28)	5357 (5044 – 5663)
Belgium	0·91	5009 (4846 – 5185)	29·0 (28·2 – 29·8)	9·91 (9·08 – 10·99)	147 (131 – 162)	27·5 (26·8 – 28·3)	0·18 (0·17 – 0·19)	7869 (7298 – 8435)
Cyprus	0·72	2287 (2163 – 2422)	27·8 (27·0 – 28·6)	5·81 (4·96 – 6·68)	110 (93 – 130)	27·0 (26·2 – 27·8)	0·1 (0·09 – 0·12)	5938 (4956 – 7154)
Denmark	1·09	5228 (5076 – 5386)	29·1 (28·4 – 30·0)	5·69 (5·57 – 5·82)	267 (256 – 281)	27·6 (26·8 – 28·4)	0·16 (0·15 – 0·17)	9136 (8556 – 9721)
Finland	1·02	4296 (4216 – 4375)	28·8 (28·1 – 29·6)	5·41 (5·23 – 5·58)	229 (218 – 240)	27·5 (26·7 – 28·3)	0·18 (0·17 – 0·19)	6627 (6175 – 7104)
France	0·9	4821 (4728 – 4907)	28·7 (27·9 – 29·5)	8·13 (7·91 – 8·38)	170 (163 – 178)	27·4 (26·6 – 28·2)	0·2 (0·19 – 0·22)	6576 (6049 – 7063)
Germany	0·87	5637 (5425 – 5857)	29·2 (28·4 – 29·9)	10·37 (9·43 – 11·41)	159 (143 – 175)	27·6 (26·8 – 28·4)	0·26 (0·25 – 0·27)	6050 (5674 – 6445)
Greece	0·68	2309 (2151 – 2479)	27·5 (26·7 – 28·3)	5·21 (4·48 – 6·15)	122 (102 – 146)	26·8 (26·1 – 27·7)	0·2 (0·16 – 0·23)	3176 (2623 – 3857)
Iceland	1·27	4447 (4260 – 4653)	29·1 (28·2 – 29·8)	7·15 (6·91 – 7·4)	180 (171 – 193)	27·6 (26·8 – 28·4)	0·13 (0·12 – 0·14)	9402 (8699 – 10 124)
Ireland	0·92	5406 (4961 – 5864)	29·5 (28·7 – 30·4)	9·37 (8·83 – 9·86)	170 (153 – 189)	27·8 (27·0 – 28·7)	0·15 (0·14 – 0·16)	10 189 (9131 – 11 362)
Israel	1·04	2613 (2481 – 2767)	28·1 (27·3 – 28·8)	8·6 (7·55 – 10·0)	86 (73 – 99)	27·1 (26·4 – 27·9)	0·17 (0·16 – 0·18)	4183 (3879 – 4499)
Italy	0·82	3505 (3378 – 3632)	28·4 (27·7 – 29·2)	9·69 (8·75 – 10·95)	103 (91 – 115)	27·3 (26·5 – 28·1)	0·13 (0·12 – 0·14)	7259 (6625 – 7897)
Luxembourg	0·99	6708 (6306 – 7217)	31·6 (30·5 – 32·8)	7·74 (7·49 – 7·99)	274 (253 – 299)	28·7 (27·6 – 29·9)	0·16 (0·15 – 0·17)	12 262 (11 100 – 13 522)
Malta	0·66	3769 (3606 – 3928)	28·0 (27·2 – 28·7)	13·78 (11·74 – 15·81)	76 (66 – 90)	27·1 (26·3 – 27·9)	0·14 (0·11 – 0·17)	7422 (6179 – 8997)
Netherlands	0·9	5696 (5422 – 6005)	29·4 (28·6 – 30·2)	5·37 (4·98 – 5·81)	312 (282 – 343)	27·7 (26·9 – 28·6)	0·13 (0·11 – 0·15)	12 313 (10 701 – 14 114)
Norway	1·11	7071 (6791 – 7337)	30·4 (29·5 – 31·4)	5·21 (5·03 – 5·38)	413 (388 – 438)	28·2 (27·3 – 29·1)	0·2 (0·19 – 0·21)	10 060 (9375 – 10 820)
Portugal	0·66	2779 (2650 – 2916)	27·6 (26·8 – 28·4)	5·49 (5·05 – 5·87)	139 (128 – 155)	26·9 (26·1 – 27·7)	0·11 (0·11 – 0·12)	6610 (6121 – 7147)
Spain	0·74	3453 (3359 – 3544)	28·2 (27·5 – 29·0)	7·85 (7·02 – 8·83)	124 (110 – 140)	27·2 (26·4 – 28·0)	0·12 (0·11 – 0·12)	8027 (7567 – 8480)
Sweden	1·02	5786 (5527 – 6046)	29·2 (28·4 – 30·0)	4·21 (3·59 – 4·92)	402 (343 – 467)	27·6 (26·8 – 28·5)	0·14 (0·12 – 0·17)	11 249 (9420 – 13 328)
Switzerland	1·26	7654 (7379 – 7890)	29·9 (29·1 – 30·8)	5·76 (5·42 – 6·13)	398 (369 – 428)	28·0 (27·1 – 28·9)	0·17 (0·16 – 0·18)	12 421 (11 587 – 13 301)
United Kingdom	0·89	4394 (4243 – 4571)	28·4 (27·3 – 30·6)	5·16 (4·47 – 5·89)	243 (210 – 284)	27·3 (26·4 – 28·5)	0·13 (0·12 – 0·15)	9037 (7699 – 10 398)
Latin America and Caribbean	‥	‥	‥	4·71 (4·22 – 5·26)	‥	‥	0·07 (0·06 – 0·08)	‥
Andean Latin America	‥	‥	‥	5·7 (4·85 – 6·96)	‥	‥	0·07 (0·06 – 0·08)	‥
Bolivia	0·44	462 (439 – 486)	28·2 (25·8 – 30·8)	5·36 (4·51 – 6·68)	25 (19 – 30)	23·2 (21·0 – 25·5)	0·07 (0·06 – 0·08)	1576 (1264 – 1899)

Ecuador	0·54	1029 (944 – 1126)	30·2 (27·7 – 32·7)	5·75 (4·82 – 6·91)	54 (43 – 67)	24·1 (21·8 – 26·4)	0·07 (0·07 – 0·08)	3297 (2890 – 3777)
Peru	0·47	712 (672 – 755)	30·4 (27·9 – 32·9)	5·8 (4·95 – 7·15)	38 (29 – 45)	24·2 (21·9 – 26·5)	0·07 (0·06 – 0·08)	2499 (2011 – 3035)
Caribbean	··	··	··	3·34 (2·87 – 3·84)	··	··	0·08 (0·06 – 0·09)	··
Antigua and Barbuda	0·61	1250 (1176 – 1340)	32·7 (30·2 – 35·5)	3·69 (3·11 – 4·31)	111 (92 – 136)	25·2 (22·6 – 27·8)	0·07 (0·06 – 0·08)	4667 (3697 – 5695)
The Bahamas	1·06	1842 (1720 – 1968)	33·1 (30·5 – 35·9)	4·48 (3·8 – 5·23)	136 (112 – 167)	25·3 (22·6 – 28·0)	0·09 (0·08 – 0·12)	4931 (3892 – 6170)
Barbados	0·91	1295 (1234 – 1359)	31·6 (29·0 – 34·2)	2·5 (2·13 – 2·88)	164 (137 – 197)	24·7 (22·3 – 27·2)	0·12 (0·1 – 0·14)	2790 (2236 – 3425)
Belize	0·56	540 (504 – 583)	29·3 (26·8 – 31·8)	2·91 (2·45 – 3·43)	55 (44 – 67)	23·7 (21·4 – 26·0)	0·05 (0·05 – 0·07)	2368 (1883 – 2884)
Bermuda	1·46	··	··	3·85 (3·28 – 4·48)	··	··	0·08 (0·07 – 0·1)	··
Cuba	0·48	1026 (883 – 1189)	31·0 (28·5 – 33·6)	3·54 (3·03 – 4·06)	90 (72 – 113)	24·4 (22·1 – 26·8)	0·09 (0·08 – 0·11)	2760 (2160 – 3595)
Dominica	0·69	613 (577 – 650)	30·2 (27·7 – 32·8)	5·54 (4·68 – 6·47)	33 (27 – 40)	24·1 (21·8 – 26·3)	0·07 (0·06 – 0·09)	1995 (1587 – 2447)
Dominican Republic	0·43	985 (913 – 1056)	30·9 (28·3 – 33·4)	3·29 (2·8 – 3·85)	92 (77 – 112)	24·4 (22·1 – 26·7)	0·09 (0·07 – 0·1)	2760 (2180 – 3365)
Grenada	0·68	717 (671 – 771)	30·6 (28·0 – 33·2)	3·31 (2·82 – 3·88)	66 (54 – 81)	24·2 (22·0 – 26·6)	0·07 (0·06 – 0·09)	2435 (1949 – 2959)
Guyana	0·56	335 (298 – 379)	28·2 (25·8 – 30·7)	4·73 (3·94 – 5·5)	20 (16 – 25)	23·2 (21·0 – 25·4)	0·04 (0·04 – 0·05)	1796 (1393 – 2278)
Haiti	0·37	141 (136 – 147)	23·8 (21·4 – 26·4)	2·44 (2·05 – 2·83)	14 (11 – 17)	21·1 (18·9 – 23·7)	0·05 (0·05 – 0·07)	558 (447 – 679)
Jamaica	0·53	511 (473 – 557)	29·4 (26·9 – 31·9)	2·09 (1·79 – 2·41)	72 (59 – 87)	23·7 (21·4 – 26·0)	0·03 (0·02 – 0·03)	4186 (3349 – 5145)
Puerto Rico	0·94	··	··	6·18 (5·29 – 7·17)	··	··	0·08 (0·07 – 0·1)	··
Saint Lucia	0·69	735 (673 – 795)	30·3 (27·7 – 32·8)	4·24 (3·6 – 4·93)	53 (43 – 64)	24·1 (21·9 – 26·4)	0·07 (0·06 – 0·08)	2565 (2036 – 3158)
Saint Vincent and the Grenadines	0·61	543 (519 – 568)	30·1 (27·6 – 32·6)	3·31 (2·83 – 3·88)	49 (41 – 60)	24·0 (21·8 – 26·4)	0·07 (0·06 – 0·09)	1799 (1457 – 2211)
Suriname	0·35	881 (792 – 968)	31·6 (29·0 – 34·1)	3·35 (2·85 – 3·88)	83 (68 – 101)	24·7 (22·3 – 27·1)	0·07 (0·06 – 0·08)	3128 (2467 – 3886)
Trinidad and Tobago	0·5	1999 (1879 – 2119)	34·0 (31·2 – 37·0)	2·86 (2·44 – 3·33)	239 (197 – 288)	25·7 (23·0 – 28·5)	0·11 (0·09 – 0·14)	4557 (3604 – 5575)
Virgin Islands U·S·	1·75	··	··	4·81 (4·07 – 5·67)	··	··	0·11 (0·09 – 0·13)	··
Central Latin America	··	··	··	4·43 (3·81 – 5·1)	··	··	0·06 (0·05 – 0·07)	··
Colombia	0·41	884 (822 – 948)	30·8 (28·3 – 33·3)	6·6 (5·66 – 7·7)	41 (34 – 50)	24·3 (22·0 – 26·7)	0·04 (0·03 – 0·04)	5897 (5084 – 6673)
Costa Rica	0·68	1383 (1312 – 1459)	31·2 (28·7 – 33·8)	3·2 (2·7 – 3·73)	136 (111 – 166)	24·5 (22·2 – 27·0)	0·05 (0·05 – 0·07)	6305 (5007 – 7685)
El Salvador	0·48	607 (575 – 641)	29·2 (26·7 – 31·7)	2·01 (1·84 – 2·16)	88 (78 – 100)	23·6 (21·4 – 25·9)	0·02 (0·02 – 0·02)	6919 (5851 – 8226)
Guatemala	0·52	502 (478 – 530)	28·8 (26·3 – 31·4)	4·59 (3·81 – 5·56)	32 (25 – 39)	23·5 (21·2 – 25·7)	0·05 (0·04 – 0·07)	2220 (1764 – 2695)
Honduras	0·47	382 (359 – 409)	27·3 (24·9 – 29·8)	4·04 (3·36 – 4·73)	26 (21 – 32)	22·8 (20·7 – 25·1)	0·05 (0·04 – 0·07)	1696 (1311 – 2134)
Mexico	0·45	1113 (1063 – 1164)	31·7 (29·1 – 34·3)	3·71 (3·2 – 4·28)	95 (80 – 112)	24·7 (22·3 – 27·2)	0·06 (0·05 – 0·07)	4577 (3592 – 5576)
Nicaragua	0·36	454 (431 – 484)	27·3 (24·8 – 29·8)	6·44 (5·39 – 7·53)	19 (16 – 24)	22·8 (20·6 – 25·0)	0·05 (0·05 – 0·07)	1907 (1511 – 2342)
Panama	0·59	1645 (1544 – 1745)	32·3 (29·7 – 35·0)	9·93 (8·51 – 11·47)	54 (44 – 65)	25·0 (22·5 – 27·5)	0·1 (0·08 – 0·12)	4095 (3338 – 5056)
Venezuela	1	530 (482 – 590)	31·7 (29·2 – 34·3)	3·66 (3·08 – 4·3)	46 (37 – 58)	24·7 (22·3 – 27·2)	0·08 (0·06 – 0·09)	1717 (1376 – 2124)
Tropical Latin America	··	··	··	5·05 (4·73 – 5·39)	··	··	0·08 (0·08 – 0·1)	··
Brazil	0·58	1408 (1352 – 1467)	31·4 (28·8 – 34·0)	5·09 (4·77 – 5·43)	87 (77 – 97)	24·6 (22·2 – 27·1)	0·09 (0·08 – 0·1)	4054 (3403 – 4761)
Paraguay	0·42	767 (703 – 832)	29·3 (26·7 – 31·8)	3·88 (3·3 – 4·58)	58 (47 – 71)	23·6 (21·4 – 25·9)	0·06 (0·05 – 0·07)	3009 (2402 – 3707)
North Africa and Middle East	··	··	··	5·72 (5·07 – 6·61)	··	··	0·1 (0·09 – 0·11)	··
Afghanistan	0·29	170 (154 – 184)	20·7 (18·0 – 23·5)	5·59 (4·67 – 6·82)	6 (5 – 8)	28·6 (24·7 – 32·7)	0·08 (0·07 – 0·1)	599 (457 – 769)
Algeria	0·27	1038 (978 – 1095)	27·5 (24·5 – 30·7)	4·06 (3·45 – 4·8)	70 (57 – 85)	32·6 (29·0 – 36·3)	0·09 (0·07 – 0·11)	3787 (2864 – 4793)
Bahrain	0·5	2449 (2254 – 2641)	31·4 (27·8 – 34·9)	7·52 (6·24 – 9·37)	103 (79 – 127)	34·5 (30·7 – 38·3)	0·04 (0·03 – 0·05)	20 526 (15 697 – 25 631)
Egypt	0·17	505 (481 – 532)	26·7 (23·9 – 29·7)	7·77 (6·61 – 9·58)	18 (14 – 21)	32·2 (28·6 – 35·8)	0·15 (0·13 – 0·18)	1087 (860 – 1345)
Iran	0·26	1328 (1220 – 1445)	28·2 (25·1 – 31·4)	5·63 (4·85 – 6·59)	67 (54 – 81)	33·0 (29·4 – 36·7)	0·06 (0·05 – 0·08)	7217 (5582 – 9069)
Iraq	0·33	575 (498 – 671)	27·5 (24·5 – 30·7)	2·15 (1·8 – 2·54)	74 (58 – 94)	32·7 (29·0 – 36·2)	0·08 (0·06 – 0·1)	2387 (1762 – 3169)

Jordan	0·44	761 (694 – 837)	26·8 (23·9 – 29·9)	3·82 (3·2 – 4·56)	54 (43 – 67)	32·3 (28·6 – 35·8)	0·2 (0·17 – 0·24)	1240 (962 – 1571)
Kuwait	0·44	2571 (2330 – 2804)	32·9 (29·1 – 36·6)	5·73 (4·83 – 7·03)	149 (117 – 183)	35·1 (31·3 – 39·2)	0·09 (0·07 – 0·11)	10 690 (7952 – 14 003)
Lebanon	0·57	1207 (1106 – 1322)	28·0 (24·9 – 31·2)	4·0 (3·38 – 4·76)	85 (67 – 105)	32·9 (29·3 – 36·5)	0·08 (0·07 – 0·09)	5140 (4048 – 6350)
Libya	0·45	642 (558 – 732)	28·0 (24·9 – 31·1)	5·77 (4·97 – 6·86)	31 (24 – 39)	32·9 (29·2 – 36·6)	0·1 (0·08 – 0·12)	2182 (1628 – 2884)
Morocco	0·36	474 (443 – 509)	25·6 (22·8 – 28·5)	3·57 (2·97 – 4·35)	34 (27 – 42)	31·6 (27·9 – 35·2)	0·04 (0·03 – 0·05)	3715 (2898 – 4670)
Oman	0·42	1678 (1496 – 1877)	31·3 (27·8 – 34·7)	6·58 (5·52 – 8·09)	80 (62 – 101)	34·4 (30·7 – 38·2)	0·04 (0·03 – 0·05)	14 217 (10 795 – 18 105)
Palestine	0·68	247 (222 – 274)	24·0 (21·3 – 26·8)	4·9 (4·1 – 5·83)	12 (10 – 15)	30·7 (26·9 – 34·3)	0·08 (0·06 – 0·1)	964 (721 – 1298)
Qatar	0·54	3276 (2790 – 3782)	34·6 (30·6 – 38·7)	9·68 (7·99 – 11·78)	118 (89 – 151)	35·8 (31·8 – 40·2)	0·09 (0·07 – 0·11)	13 771 (9948 – 18 967)
Saudi Arabia	0·4	2877 (2659 – 3114)	31·8 (28·2 – 35·3)	6·87 (5·95 – 7·94)	134 (110 – 161)	34·6 (30·9 – 38·5)	0·08 (0·06 – 0·09)	13 062 (10 279 – 16 358)
Sudan	0·48	281 (242 – 329)	23·6 (20·9 – 26·5)	4·4 (3·7 – 5·4)	15 (12 – 20)	30·5 (26·7 – 34·2)	0·07 (0·06 – 0·09)	1197 (898 – 1595)
Syria	0·06	246 (216 – 276)	24·6 (21·9 – 27·5)	4·5 (3·84 – 5·37)	14 (11 – 17)	31·1 (27·3 – 34·7)	0·11 (0·09 – 0·14)	673 (521 – 866)
Tunisia	0·29	821 (785 – 857)	26·8 (23·9 – 29·8)	5·51 (4·73 – 6·57)	40 (32 – 48)	32·3 (28·7 – 35·8)	0·08 (0·07 – 0·09)	3401 (2684 – 4247)
Turkey	0·37	1054 (980 – 1138)	28·6 (25·5 – 31·8)	8·82 (8·48 – 9·13)	34 (30 – 39)	33·2 (29·6 – 36·9)	0·16 (0·15 – 0·17)	2190 (1886 – 2518)
United Arab Emirates	0·57	2508 (2342 – 2683)	32·7 (29·0 – 36·4)	6·04 (4·86 – 7·74)	137 (104 – 173)	35·0 (31·2 – 39·0)	0·07 (0·05 – 0·08)	13 130 (10 010 – 16 708)
Yemen	0·47	185 (157 – 217)	23·2 (20·4 – 26·0)	2·41 (2·14 – 2·68)	18 (14 – 23)	30·2 (26·4 – 33·9)	0·07 (0·06 – 0·09)	804 (581 – 1108)
South Asia	··	··	··	3·54 (2·98 – 4·15)	··	··	0·05 (0·05 – 0·06)	··
Bangladesh	0·36	94 (89 – 99)	18·1 (15·9 – 20·3)	3·52 (2·96 – 4·13)	5 (4 – 6)	18·8 (16·5 – 21·5)	0·05 (0·05 – 0·07)	326 (257 – 410)
Bhutan	0·31	287 (264 – 315)	20·6 (18·2 – 23·1)	4·19 (3·56 – 4·91)	14 (11 – 17)	20·2 (17·6 – 23·1)	0·07 (0·06 – 0·08)	842 (667 – 1078)
India	0·26	243 (238 – 250)	19·6 (17·4 – 22·0)	3·41 (2·86 – 4·02)	14 (11 – 17)	19·7 (17·2 – 22·6)	0·05 (0·05 – 0·06)	875 (731 – 1048)
Nepal	0·29	165 (154 – 177)	17·3 (15·2 – 19·4)	2·21 (1·86 – 2·54)	13 (11 – 16)	18·4 (16·1 – 21·0)	0·06 (0·05 – 0·07)	518 (411 – 660)
Pakistan	0·27	145 (135 – 157)	19·3 (17·1 – 21·7)	4·63 (3·91 – 5·41)	6 (5 – 7)	19·5 (17·0 – 22·4)	0·05 (0·05 – 0·07)	525 (399 – 667)
Southeast Asia East Asia and Oceania	··	··	··	5·15 (4·72 – 5·61)	··	··	0·11 (0·1 – 0·12)	··
East Asia	··	··	··	5·62 (5·26 – 5·99)	··	··	0·14 (0·13 – 0·16)	··
China	0·51	839 (798 – 878)	37·7 (35·9 – 39·4)	5·32 (5·0 – 5·64)	59 (54 – 65)	27·8 (26·1 – 29·5)	0·15 (0·13 – 0·16)	1609 (1448 – 1790)
North Korea	0·99	124 (116 – 133)	30·7 (28·6 – 32·9)	7·27 (6·19 – 8·54)	5 (4 – 6)	25·0 (22·9 – 27·1)	0·13 (0·11 – 0·15)	247 (200 – 300)
Taiwan	0·46	2268 (2010 – 2543)	41·8 (39·5 – 43·8)	21·0 (18·05 – 24·23)	45 (37 – 55)	29·3 (27·2 – 31·5)	0·11 (0·09 – 0·14)	5845 (4615 – 7279)
Oceania	··	··	··	5·39 (4·53 – 6·77)	··	··	0·06 (0·05 – 0·08)	··
American Samoa	1	··	··	6·69 (5·64 – 8·02)	··	··	0·06 (0·05 – 0·07)	··
Federated States of Micronesia	0·9	281 (268 – 295)	32·6 (30·6 – 34·5)	5·88 (4·96 – 6·92)	16 (13 – 19)	25·8 (23·9 – 27·7)	0·06 (0·05 – 0·08)	1153 (931 – 1393)
Fiji	0·56	349 (324 – 377)	36·2 (34·6 – 37·9)	6·88 (5·78 – 8·6)	19 (15 – 23)	27·2 (25·5 – 29·0)	0·06 (0·05 – 0·08)	1509 (1208 – 1794)
Guam	1	··	··	7·6 (6·56 – 8·92)	··	··	0·07 (0·06 – 0·09)	··
Kiribati	0·78	201 (177 – 230)	30·4 (28·2 – 32·6)	5·2 (4·33 – 6·21)	12 (9 – 15)	24·8 (22·7 – 27·0)	0·06 (0·05 – 0·07)	894 (702 – 1124)
Marshall Islands	0·94	602 (568 – 634)	33·0 (31·1 – 34·9)	5·81 (4·84 – 7·23)	35 (27 – 42)	25·9 (24·1 – 27·9)	0·06 (0·05 – 0·07)	2591 (2096 – 3121)
Northern Mariana Islands	1	··	··	6·92 (5·71 – 8·7)	··	··	0·06 (0·05 – 0·07)	··
Papua New Guinea	0·67	129 (122 – 137)	31·6 (29·6 – 33·6)	5·09 (4·26 – 6·5)	8 (6 – 10)	25·4 (23·4 – 27·4)	0·06 (0·05 – 0·08)	512 (419 – 626)
Samoa	0·67	339 (313 – 367)	34·4 (32·7 – 36·1)	5·87 (4·89 – 7·09)	20 (16 – 25)	26·5 (24·8 – 28·3)	0·06 (0·05 – 0·07)	1475 (1184 – 1787)
Solomon Islands	0·9	155 (142 – 168)	30·7 (28·6 – 32·9)	5·19 (4·3 – 6·16)	9 (7 – 11)	24·9 (22·9 – 27·1)	0·06 (0·05 – 0·07)	693 (549 – 851)
Tonga	0·67	267 (253 – 282)	34·2 (32·5 – 36·0)	5·92 (5·03 – 7·17)	16 (13 – 19)	26·5 (24·7 – 28·3)	0·06 (0·05 – 0·08)	1111 (915 – 1328)
Vanuatu	0·99	161 (139 – 191)	32·2 (30·3 – 34·2)	5·36 (4·49 – 6·56)	10 (7 – 12)	25·6 (23·8 – 27·6)	0·06 (0·05 – 0·08)	641 (500 – 814)
Southeast Asia	··	··	··	4·13 (3·49 – 4·9)	··	··	0·03 (0·02 – 0·03)	··
Cambodia	0·33	225 (208 – 243)	32·7 (30·8 – 34·7)	3·93 (3·54 – 4·43)	19 (16 – 22)	25·8 (24·0 – 27·8)	0·02 (0·02 – 0·02)	2954 (2356 – 3614)

Indonesia	0·3	404 (370 – 439)	36·9 (35·2 – 38·5)	3·92 (3·21 – 4·85)	38 (30 – 48)	27·5 (25·8 – 29·2)	0·03 (0·02 – 0·04)	3996 (3056 – 5086)
Laos	0·33	185 (168 – 205)	34·4 (32·7 – 36·2)	3·69 (3·11 – 4·36)	17 (14 – 21)	26·5 (24·8 – 28·3)	0·02 (0·02 – 0·02)	2485 (1989 – 3082)
Malaysia	0·33	1122 (1077 – 1166)	39·9 (37·9 – 41·7)	8·44 (7·14 – 9·89)	53 (44 – 63)	28·6 (26·8 – 30·6)	0·03 (0·02 – 0·03)	11 232 (9156 – 13 672)
Maldives	0·62	1965 (1680 – 2262)	38·0 (36·2 – 39·7)	4·28 (3·6 – 5·07)	174 (137 – 218)	27·9 (26·3 – 29·7)	0·03 (0·02 – 0·03)	20 651 (16 123 – 25 683)
Mauritius	0·45	1153 (1073 – 1235)	39·0 (37·1 – 40·7)	5·21 (4·41 – 6·11)	87 (72 – 105)	28·3 (26·6 – 30·1)	0·03 (0·03 – 0·04)	10 051 (8136 – 12 381)
Myanmar	0·2	310 (277 – 348)	34·1 (32·4 – 35·8)	4·13 (3·48 – 4·86)	26 (21 – 32)	26·4 (24·6 – 28·2)	0·02 (0·02 – 0·02)	4124 (3287 – 5176)
Philippines	0·36	351 (330 – 372)	35·4 (33·7 – 37·1)	5·19 (4·37 – 6·05)	24 (20 – 29)	26·9 (25·2 – 28·7)	0·02 (0·02 – 0·02)	4731 (3826 – 5748)
Sri Lanka	0·31	370 (349 – 395)	36·9 (35·2 – 38·5)	4·95 (4·17 – 5·78)	28 (23 – 33)	27·5 (25·8 – 29·2)	0·03 (0·03 – 0·04)	3268 (2639 – 3977)
Seychelles	0·53	1015 (892 – 1141)	40·1 (38·0 – 41·9)	4·1 (3·54 – 4·67)	99 (81 – 120)	28·7 (26·9 – 30·6)	0·03 (0·03 – 0·04)	9062 (7008 – 11 218)
Thailand	0·35	640 (605 – 680)	38·3 (36·5 – 40·0)	2·9 (2·45 – 3·38)	85 (71 – 102)	28·0 (26·4 – 29·8)	0·03 (0·03 – 0·04)	5170 (4124 – 6381)
Timor-Leste	0·52	106 (91 – 122)	35·0 (33·3 – 36·7)	4·99 (4·19 – 6·0)	7 (6 – 9)	26·8 (25·1 – 28·5)	0·03 (0·02 – 0·03)	1114 (874 – 1424)
Vietnam	0·34	348 (326 – 372)	34·7 (33·0 – 36·4)	2·91 (2·46 – 3·37)	41 (35 – 50)	26·6 (24·9 – 28·4)	0·03 (0·03 – 0·04)	2981 (2422 – 3641)
Sub-Saharan Africa	··	··	··	3·74 (3·2 – 4·39)	··	··	0·06 (0·05 – 0·07)	··
Central sub-Saharan Africa	··	··	··	3·65 (3·05 – 4·4)	··	··	0·07 (0·06 – 0·09)	··
Angola	0·54	201 (177 – 226)	32·0 (30·1 – 33·9)	3·85 (3·18 – 4·49)	17 (13 – 21)	24·7 (23·1 – 26·7)	0·07 (0·06 – 0·09)	676 (529 – 864)
Central African Republic	0·54	30 (28 – 32)	24·0 (22·4 – 25·8)	3·56 (2·93 – 4·52)	2 (2 – 3)	21·2 (19·8 – 22·8)	0·07 (0·06 – 0·09)	87 (68 – 106)
Congo (Brazzaville)	0·33	180 (162 – 199)	31·1 (29·3 – 32·9)	4·27 (3·6 – 5·22)	13 (10 – 16)	24·4 (22·8 – 26·2)	0·08 (0·07 – 0·1)	552 (437 – 683)
Democratic Republic of the Congo	0·54	43 (39 – 48)	24·4 (22·9 – 26·2)	3·52 (2·92 – 4·32)	3 (2 – 4)	21·4 (20·0 – 23·0)	0·07 (0·06 – 0·09)	128 (101 – 158)
Equatorial Guinea	0·39	1044 (902 – 1217)	37·7 (34·7 – 40·8)	4·86 (4·07 – 5·87)	81 (64 – 105)	27·1 (24·3 – 29·8)	0·08 (0·07 – 0·1)	3478 (2649 – 4457)
Gabon	0·42	495 (456 – 542)	35·0 (32·5 – 37·6)	4·65 (3·88 – 5·71)	38 (30 – 46)	26·0 (23·8 – 28·3)	0·09 (0·07 – 0·11)	1447 (1170 – 1784)
Eastern sub-Saharan Africa	··	··	··	4·03 (3·46 – 4·7)	··	··	0·05 (0·04 – 0·06)	··
Burundi	0·38	64 (60 – 69)	24·1 (22·5 – 25·9)	6·46 (5·43 – 7·85)	2 (2 – 3)	21·3 (19·8 – 22·9)	0·06 (0·05 – 0·07)	249 (199 – 308)
Comoros	0·49	129 (118 – 140)	26·3 (24·8 – 28·0)	4·44 (3·68 – 5·54)	8 (6 – 9)	22·3 (21·0 – 23·8)	0·11 (0·09 – 0·13)	265 (207 – 329)
Djibouti	0·55	151 (140 – 164)	28·9 (27·3 – 30·6)	4·38 (3·62 – 5·4)	10 (8 – 12)	23·5 (22·1 – 25·0)	0·06 (0·05 – 0·07)	622 (497 – 754)
Eritrea	0·58	40 (36 – 46)	25·3 (23·8 – 27·0)	4·15 (3·46 – 4·91)	2 (2 – 3)	21·9 (20·5 – 23·4)	0·05 (0·04 – 0·06)	170 (131 – 216)
Ethiopia	0·37	84 (77 – 92)	26·2 (24·7 – 27·8)	3·46 (2·86 – 4·08)	6 (5 – 8)	22·2 (20·9 – 23·8)	0·03 (0·02 – 0·03)	667 (531 – 851)
Kenya	0·45	189 (182 – 197)	28·7 (27·2 – 30·4)	4·01 (3·43 – 4·76)	14 (11 – 16)	23·4 (22·0 – 24·9)	0·04 (0·04 – 0·05)	1048 (846 – 1245)
Madagascar	0·27	82 (74 – 91)	26·0 (24·5 – 27·6)	4·17 (3·45 – 5·28)	5 (4 – 6)	22·2 (20·8 – 23·7)	0·05 (0·04 – 0·06)	386 (308 – 490)
Malawi	0·24	143 (139 – 148)	25·3 (23·7 – 26·9)	3·17 (2·63 – 3·79)	11 (10 – 14)	21·8 (20·4 – 23·4)	0·05 (0·04 – 0·06)	601 (489 – 710)
Mozambique	0·3	87 (84 – 91)	25·3 (23·8 – 27·0)	3·92 (3·28 – 4·71)	6 (5 – 7)	21·9 (20·4 – 23·4)	0·05 (0·04 – 0·06)	386 (309 – 472)
Rwanda	0·36	156 (148 – 165)	26·6 (25·1 – 28·3)	5·47 (5·03 – 5·92)	8 (7 – 8)	22·4 (21·1 – 24·0)	0·03 (0·02 – 0·03)	1378 (1106 – 1718)
Somalia	0·49	47 (46 – 48)	23·0 (21·3 – 24·9)	3·77 (3·12 – 4·56)	3 (2 – 4)	20·8 (19·2 – 22·5)	0·05 (0·04 – 0·06)	202 (158 – 251)
South Sudan	0·08	106 (102 – 110)	28·6 (27·0 – 30·3)	3·56 (2·98 – 4·37)	9 (7 – 10)	23·4 (21·9 – 24·9)	0·06 (0·05 – 0·07)	439 (348 – 537)
Tanzania	0·3	166 (151 – 185)	28·0 (26·5 – 29·7)	5·02 (4·17 – 6·03)	9 (8 – 11)	23·1 (21·7 – 24·6)	0·09 (0·08 – 0·11)	415 (328 – 515)
Uganda	0·3	165 (150 – 181)	27·1 (25·6 – 28·7)	4·13 (3·82 – 4·44)	11 (10 – 12)	22·7 (21·3 – 24·2)	0·05 (0·04 – 0·05)	780 (658 – 909)
Zambia	0·33	244 (228 – 261)	29·5 (27·9 – 31·2)	2·83 (2·32 – 3·49)	26 (20 – 32)	23·7 (22·3 – 25·3)	0·06 (0·05 – 0·08)	904 (721 – 1103)
Southern sub-Saharan Africa	··	··	··	3·08 (2·61 – 3·61)	··	··	0·12 (0·09 – 0·15)	··
Botswana	0·41	1038 (951 – 1127)	34·6 (32·2 – 37·1)	3·29 (2·75 – 3·94)	109 (89 – 134)	25·8 (23·7 – 28·2)	0·07 (0·06 – 0·09)	3777 (2935 – 4698)
Lesotho	0·36	272 (261 – 284)	28·2 (26·7 – 30·0)	2·79 (2·32 – 3·35)	28 (22 – 34)	23·2 (21·8 – 24·7)	0·07 (0·05 – 0·08)	974 (787 – 1203)
Namibia	0·44	1046 (965 – 1125)	33·1 (31·0 – 35·3)	3·03 (2·54 – 3·61)	115 (93 – 139)	25·2 (23·4 – 27·3)	0·09 (0·07 – 0·11)	2997 (2395 – 3697)
South Africa	0·42	1121 (1069 – 1179)	33·9 (31·7 – 36·3)	3·24 (2·76 – 3·78)	118 (98 – 140)	25·5 (23·6 – 27·8)	0·14 (0·11 – 0·19)	2060 (1513 – 2739)

Swaziland (eSwatini)	0·36	718<br>(650 – 804)	32·4<br>(30·4 – 34·4)	2·92<br>(2·43 – 3·52)	80<br>(65 – 100)	24·9<br>(23·2 – 27·0)	0·04<br>(0·03 – 0·04)	5141<br>(4059 – 6549)
Zimbabwe	0·51	196<br>(181 – 214)	27·0<br>(25·5 – 28·7)	2·58<br>(2·15 – 3·12)	21<br>(17 – 25)	22·6<br>(21·3 – 24·2)	0·06<br>(0·05 – 0·07)	769<br>(611 – 951)
Western sub-Saharan Africa	‥	‥	‥	3·62<br>(3·06 – 4·3)	‥	‥	0·06<br>(0·05 – 0·07)	‥
Benin	0·36	88<br>(84 – 91)	27·3<br>(25·8 – 29·0)	3·66<br>(3·09 – 4·32)	7<br>(5 – 8)	22·8<br>(21·4 – 24·3)	0·05<br>(0·04 – 0·06)	400<br>(320 – 481)
Burkina Faso	0·35	97<br>(92 – 101)	26·6<br>(25·1 – 28·2)	1·84<br>(1·53 – 2·18)	14<br>(12 – 17)	22·4<br>(21·1 – 24·0)	0·05<br>(0·04 – 0·07)	402<br>(325 – 499)
Cameroon	0·38	156<br>(147 – 169)	28·6<br>(27·1 – 30·3)	3·98<br>(3·28 – 5·03)	11<br>(9 – 14)	23·4<br>(22·0 – 24·9)	0·06<br>(0·05 – 0·07)	608<br>(489 – 735)
Cape Verde	0·46	380<br>(354 – 407)	31·5<br>(29·7 – 33·4)	5·01<br>(4·23 – 6·02)	24<br>(19 – 28)	24·6<br>(22·9 – 26·4)	0·07<br>(0·06 – 0·08)	1360<br>(1090 – 1638)
Chad	0·35	105<br>(96 – 114)	27·7<br>(26·1 – 29·4)	3·45<br>(2·87 – 4·36)	8<br>(7 – 10)	22·9<br>(21·6 – 24·5)	0·05<br>(0·04 – 0·06)	525<br>(412 – 659)
Cote d'Ivoire	0·4	184<br>(168 – 202)	28·9<br>(27·4 – 30·7)	3·84<br>(3·18 – 4·59)	14<br>(11 – 17)	23·5<br>(22·1 – 25·0)	0·06<br>(0·05 – 0·07)	699<br>(571 – 862)
The Gambia	0·26	135<br>(127 – 144)	26·7<br>(25·2 – 28·3)	4·86<br>(4·04 – 5·77)	7<br>(6 – 9)	22·5<br>(21·1 – 24·1)	0·06<br>(0·05 – 0·07)	551<br>(438 – 669)
Ghana	0·32	248<br>(226 – 272)	29·6<br>(27·9 – 31·4)	4·12<br>(3·79 – 4·45)	18<br>(16 – 20)	23·8<br>(22·4 – 25·4)	0·04<br>(0·03 – 0·04)	1635<br>(1311 – 1954)
Guinea	0·35	65<br>(62 – 69)	25·8<br>(24·3 – 27·4)	3·57<br>(2·98 – 4·28)	5<br>(4 – 6)	22·1<br>(20·7 – 23·6)	0·05<br>(0·04 – 0·06)	282<br>(226 – 344)
Guinea-Bissau	0·4	115<br>(107 – 125)	26·1<br>(24·7 – 27·7)	3·65<br>(3·07 – 4·35)	8<br>(7 – 10)	22·2<br>(20·9 – 23·8)	0·06<br>(0·05 – 0·07)	460<br>(366 – 571)
Liberia	13·72	191<br>(185 – 199)	24·3<br>(22·8 – 26·1)	3·62<br>(3·04 – 4·35)	13<br>(10 – 16)	21·4<br>(20·0 – 23·0)	0·06<br>(0·05 – 0·07)	736<br>(591 – 907)
Mali	0·36	114<br>(108 – 120)	27·2<br>(25·8 – 29·0)	2·64<br>(2·34 – 2·96)	12<br>(10 – 14)	22·7<br>(21·4 – 24·3)	0·04<br>(0·03 – 0·04)	680<br>(593 – 771)
Mauritania	0·29	179<br>(165 – 192)	29·6<br>(27·9 – 31·3)	4·12<br>(3·42 – 4·97)	13<br>(10 – 16)	23·8<br>(22·3 – 25·4)	0·07<br>(0·06 – 0·08)	645<br>(514 – 791)
Niger	0·37	69<br>(66 – 72)	24·7<br>(23·2 – 26·4)	3·05<br>(2·49 – 3·78)	6<br>(5 – 7)	21·6<br>(20·1 – 23·2)	0·06<br>(0·05 – 0·07)	268<br>(211 – 327)
Nigeria	0·32	222<br>(200 – 246)	31·0<br>(29·3 – 32·9)	3·67<br>(3·02 – 4·44)	19<br>(15 – 24)	24·4<br>(22·8 – 26·1)	0·07<br>(0·05 – 0·08)	830<br>(650 – 1023)
Sao Tome and Principe	0·53	228<br>(210 – 250)	28·9<br>(27·3 – 30·6)	3·92<br>(3·27 – 4·77)	17<br>(13 – 21)	23·5<br>(22·1 – 25·0)	0·07<br>(0·06 – 0·08)	793<br>(631 – 977)
Senegal	0·37	123<br>(116 – 130)	27·9<br>(26·4 – 29·6)	3·67<br>(3·11 – 4·36)	9<br>(8 – 11)	23·0<br>(21·7 – 24·6)	0·04<br>(0·04 – 0·05)	664<br>(531 – 811)
Sierra Leone	0·3	188<br>(170 – 210)	26·5<br>(25·0 – 28·1)	6·68<br>(5·55 – 8·23)	8<br>(6 – 9)	22·4<br>(21·0 – 24·0)	0·05<br>(0·04 – 0·06)	815<br>(639 – 1009)
Togo	0·37	101<br>(95 – 108)	26·1<br>(24·6 – 27·7)	3·79<br>(3·19 – 4·59)	7<br>(6 – 8)	22·2<br>(20·9 – 23·8)	0·06<br>(0·05 – 0·07)	400<br>(325 – 493)

Section 7. Cost of universal health coverage

7.1. Introduction

We estimated the additional services and cost needed to meet a UHC standard for utilisation. The metric for the units of service needed was based on the 2016 volume of services per disability-adjusted-life-year (DALY) from the GBD 2016. For a given standard of utilisation per DALY, the additional units of service needed to meet the UHC standard was calculated for each country, and multiplied by the unit cost of the service in that country.

$$Cost_{ijkl} = \left(\left(\frac{volume_{Standardjkl}}{DALYs_{Standardkl}}\right) - \left(\frac{volume_{ijkl}}{DALYs_{ikl}}\right) \right) * DALYs_{ikl} * unitcost_{ij} \quad (8)$$

Where *i* refers to country, *j* to the service (outpatient or inpatient), *k* to age category, and *l* to sex.

The cost for a country was the sum of additional costs for each age category and sex that did not meet the outpatient and/or inpatient standard. A service-age-sex was included in the cost estimate if the average utilisation per DALY was less than the standard. The total cost was the sum across all countries that did not meet the standard for at least one service-age-sex category.

Two key steps to perform the calculation were to: 1) address the endogeneity of DALYs, and 2) identify a UHC standard for utilisation. A country's burden of disease is endogenous to health service utilisation in the sense that better access to and quality of care will reduce the burden. Consequently, we standardised the burden of disease across countries by removing the effects of access to and quality of care. We based our UHC standard for utilisation of personal health services on the performance of a current health system and described the methods for selecting it.

7.2. DALYs in the absence of health services

To estimate DALYS in the absence of personal health care, we regressed 2016 DALYs per capita on the SDI and a measure of health access and quality, and then created a counterfactual measure of DALYs using the predictions with the health access and quality measure set to zero. We used the GBD's Health Access and Quality (HAQ) index[27,28] as our measure of health access and quality, with two adaptations. The HAQ index was based on mortality from 32 causes that were amenable to health care; the same 32 causes that were used in the construction of the GBD UHC index. The GBD 2016 HAQ index was constructed with the mortality to incidence ratio for eight neoplasms, and the risk-standardised mortality rates for each the other 24 causes.[27]

The first adaptation was to create the HAQ index by age category using the GBD 2016 mortality results and risk adjustments, rather than using the age-standardised GBD 2016 index. The causes included in the index differed across age categories, for example GBD only reports mortality from neonatal disorders for ages 0-11 months. Consequently, the 2016 GBD HAQ cause weights for each age category were normalised to sum to one. The age-specific HAQ index was not calculated for 75 or more years of age, because the amenable age categories in the GBD 2016 HAQ index were from zero to 74 years.

The second adaptation was to calculate the difference between the observed HAQ index value, and the frontier value of the HAQ index for given SDI.[28] The difference is a measure access and quality after controlling for SDI. We used the ratio of the observed HAQ index value and the frontier for a given SDI in our regression estimates (Table S15). The HAQ index and SDI were highly correlated (correlation coefficient = 0.94). The ratio of the observed HAQ value and the frontier was moderately correlated with SDI (correlation coefficient = 0.18) and produced stable regression estimates. Total number of DALYs averted for each country in 2015 due to the presence of healthcare are reported in the Table S16.

Table S15. Regression results for disability-adjusted-life-year per person as a function of age-specific Health Access and Quality index, and Socio-demographic Index by age and sex, 2016

Table displays coefficient estimates for the regression of Socio-demographic Index (SDI) and HAQ index over HAQ index frontier on disability-adjusted-life-years. The HAQ index frontier represents the highest expected HAQ to be achieved at a given SDI level for a given age-sex group. Results reported in bold text indicate statistical significance at $p<0.05$. SDI = Socio-demographic Index, HAQ = Health Access and Quality, R^2 = R-squared (coefficient of determination).

Age Group	Sex	Intercept	Coefficient of the ratio of observed HAQ index value to HAQ index frontier at the country's SDI	SDI coefficient	R^2
Early Neonatal	Male	**176.45**	**-120.58**	**-35.61**	0.89
Early Neonatal	Female	**132.52**	**-91.47**	**-25.97**	0.89
Late Neonatal	Male	**16.66**	**-11.50**	**-3.20**	0.85
Late Neonatal	Female	**14.88**	**-10.66**	**-2.60**	0.85
Post Neonatal	Male	**4.34**	**-3.26**	**-0.81**	0.77
Post Neonatal	Female	**3.82**	**-2.82**	**-0.75**	0.75
1 to 4	Male	**1.39**	**-1.17**	**-0.18**	0.69
1 to 4	Female	**1.27**	**-1.07**	**-0.17**	0.68
5 to 9	Male	**0.27**	**-0.17**	**-0.04**	0.77
5 to 9	Female	**0.23**	**-0.14**	**-0.04**	0.75
10 to 14	Male	**0.27**	**-0.17**	**-0.03**	0.77
10 to 14	Female	**0.25**	**-0.15**	**-0.02**	0.72
15 to 19	Male	**0.33**	**-0.17**	**-0.04**	0.53
15 to 19	Female	**0.25**	**-0.11**	**-0.03**	0.59
20 to 24	Male	**0.37**	**-0.16**	**-0.05**	0.40
20 to 24	Female	**0.30**	**-0.14**	**-0.04**	0.54
25 to 29	Male	**0.50**	**-0.28**	**-0.06**	0.46
25 to 29	Female	**0.41**	**-0.23**	**-0.05**	0.43
30 to 34	Male	**0.67**	**-0.43**	**-0.07**	0.46
30 to 34	Female	**0.54**	**-0.35**	**-0.06**	0.43
35 to 39	Male	**0.86**	**-0.59**	**-0.09**	0.52
35 to 39	Female	**0.68**	**-0.45**	**-0.07**	0.53
40 to 44	Male	**1.01**	**-0.71**	**-0.10**	0.59
40 to 44	Female	**0.76**	**-0.50**	**-0.08**	0.64
45 to 49	Male	**1.17**	**-0.79**	**-0.11**	0.65
45 to 49	Female	**0.84**	**-0.53**	**-0.10**	0.75
50 to 54	Male	**1.30**	**-0.85**	**-0.12**	0.63
50 to 54	Female	**0.97**	**-0.59**	**-0.12**	0.80
55 to 59	Male	**1.41**	**-0.85**	**-0.13**	0.63
55 to 59	Female	**1.06**	**-0.60**	**-0.14**	0.82
60 to 64	Male	**1.51**	**-0.82**	**-0.15**	0.60
60 to 64	Female	**1.18**	**-0.62**	**-0.18**	0.82
65 to 69	Male	**1.65**	**-0.79**	**-0.18**	0.58

65 to 69	Female	**1.41**	**-0.69**	**-0.22**	0.81
70 to 74	Male	**1.79**	**-0.71**	**-0.22**	0.57
70 to 74	Female	**1.65**	**-0.73**	**-0.28**	0.79
75+	Male	n/a	n/a	n/a	n/a
75+	Female	n/a	n/a	n/a	n/a

Table S16. Total DALYs averted due to the presence of healthcare, 2015

Table presents the difference between the DALYs in the absence of healthcare and the total DALYs observed. Uncertainty intervals represented by the 2.5th and 97.5th percentile of the draws. DALY= Disability adjusted life year.

Table S16: Total DALYs averted due to the presence of healthcare, 2015	
**Location name**	**DALYs averted due to healthcare (in thousands) (95% UI)**
Global	..
Central Europe, Eastern Europe, and Central Asia	..
Central Asia	..
Armenia	1563 (1449 – 1660)
Azerbaijan	4891 (4442 – 5339)
Georgia	2036 (1865 – 2206)
Kazakhstan	8843 (7954 – 9626)
Kyrgyzstan	3377 (3154 – 3592)
Mongolia	1516 (1348 – 1646)
Tajikistan	4406 (3905 – 4833)
Turkmenistan	2358 (2107 – 2592)
Uzbekistan	15 531 (14 038 – 16 903)
Central Europe	..
Albania	1535 (1426 – 1635)
Bosnia and Herzegovina	1952 (1795 – 2098)
Bulgaria	3274 (2967 – 3586)
Croatia	1976 (1811 – 2136)
Czech Republic	4949 (4554 – 5338)
Hungary	4459 (4072 – 4857)
Macedonia	1030 (954 – 1101)
Montenegro	301 (280 – 323)
Poland	16 159 (14 704 – 17 524)
Romania	8761 (7989 – 9506)
Serbia	4326 (3983 – 4633)
Slovakia	2386 (2161 – 2590)
Slovenia	969 (892 – 1048)
Eastern Europe	..
Belarus	4614 (4146 – 5086)
Estonia	560 (507 – 608)
Latvia	860 (778 – 940)

Lithuania	1164 (1050 – 1268)
Moldova	2008 (1837 – 2181)
Russia	64 758 (51 978 – 76 256)
Ukraine	21 788 (18 747 – 24 448)
High-income	··
Australasia	··
Australia	11 707 (10 898 – 12 534)
New Zealand	2250 (2079 – 2405)
High-income Asia-Pacific	··
Brunei	166 (149 – 182)
Japan	58 354 (53 919 – 62 262)
Singapore	1774 (1643 – 1912)
South Korea	22 644 (20 460 – 24 676)
High-income North America	··
Canada	17 139 (15 878 – 18 321)
Greenland	24 (22 – 27)
United States	143 864 (131 186 – 154 913)
Southern Latin America	··
Argentina	21 060 (19 571 – 22 442)
Chile	9205 (8457 – 9915)
Uruguay	1700 (1587 – 1804)
Western Europe	··
Andorra	36 (33 – 39)
Austria	4115 (3800 – 4405)
Belgium	5250 (4841 – 5646)
Cyprus	405 (373 – 434)
Denmark	2623 (2417 – 2833)
Finland	2678 (2468 – 2880)
France	29 913 (27 538 – 31 955)
Germany	37 209 (34 078 – 40 253)
Greece	5298 (4935 – 5641)
Iceland	162 (150 – 174)
Ireland	2276 (2103 – 2428)
Israel	4139 (3843 – 4426)
Italy	29 745 (27 619 – 31 679)
Luxembourg	281 (260 – 300)
Malta	201 (185 – 216)
Netherlands	8344 (7728 – 8977)

Norway	2584<br>(2397 – 2760)
Portugal	5246<br>(4860 – 5585)
Spain	23 294<br>(21 738 – 24 752)
Sweden	4773<br>(4393 – 5127)
Switzerland	4124<br>(3807 – 4447)
United Kingdom	30 874<br>(28 552 – 33 110)
Latin America and Caribbean	..
Andean Latin America	..
Bolivia	4784<br>(4288 – 5240)
Ecuador	8325<br>(7724 – 8856)
Peru	17 251<br>(16 101 – 18 314)
Caribbean	..
Antigua and Barbuda	42<br>(39 – 45)
The Bahamas	172<br>(156 – 187)
Barbados	127<br>(115 – 137)
Belize	189<br>(170 – 205)
Bermuda	31<br>(28 – 33)
Cuba	6362<br>(5963 – 6757)
Dominica	32<br>(29 – 35)
Dominican Republic	5060<br>(4649 – 5430)
Grenada	45<br>(41 – 49)
Guyana	315<br>(283 – 347)
Haiti	4145<br>(3083 – 4979)
Jamaica	1385<br>(1251 – 1510)
Puerto Rico	1609<br>(1478 – 1726)
Saint Lucia	81<br>(74 – 87)
Saint Vincent and the Grenadines	52<br>(47 – 56)
Suriname	232<br>(205 – 257)
Trinidad and Tobago	590<br>(532 – 643)
Virgin Islands U.S.	47<br>(42 – 51)
Central Latin America	..
Colombia	25 865<br>(24 238 – 27 325)
Costa Rica	2563<br>(2414 – 2709)
El Salvador	3151<br>(2930 – 3355)
Guatemala	8156<br>(7444 – 8870)
Honduras	3933<br>(3553 – 4270)
Mexico	65 880<br>(61 936 – 69 498)

Nicaragua	3452<br>(3240 – 3656)
Panama	2000<br>(1854 – 2133)
Venezuela	16 130<br>(14 885 – 17 225)
Tropical Latin America	..
Brazil	88 540<br>(81 458 – 95 429)
Paraguay	3047<br>(2816 – 3280)
North Africa and Middle East	..
Afghanistan	14 701<br>(11 590 – 17 574)
Algeria	19 792<br>(18 148 – 21 307)
Bahrain	716<br>(658 – 770)
Egypt	51 214<br>(47 343 – 55 008)
Iran	43 334<br>(39 055 – 47 087)
Iraq	22 181<br>(19 117 – 25 348)
Jordan	4539<br>(4244 – 4830)
Kuwait	1959<br>(1808 – 2114)
Lebanon	3280<br>(3063 – 3494)
Libya	3007<br>(2761 – 3231)
Morocco	16 217<br>(14 891 – 17 445)
Oman	2492<br>(2309 – 2669)
Palestine	3621<br>(3350 – 3893)
Qatar	1132<br>(1036 – 1223)
Saudi Arabia	14 666<br>(13 395 – 15 720)
Sudan	17 294<br>(13 831 – 19 767)
Syria	9306<br>(6818 – 11 748)
Tunisia	5796<br>(5360 – 6186)
Turkey	39 596<br>(36 406 – 42 426)
United Arab Emirates	4793<br>(4299 – 5223)
Yemen	15 763<br>(13 342 – 18 139)
South Asia	..
Bangladesh	85 049<br>(79 336 – 90 625)
Bhutan	348<br>(315 – 380)
India	512 823<br>(463 012 – 556 191)
Nepal	15 797<br>(14 516 – 17 024)
Pakistan	82 981<br>(73 591 – 91 998)
Southeast Asia East Asia and Oceania	..
East Asia	..
China	734 620<br>(689 037 – 776 947)

North Korea	15 010<br>(13 781 – 16 080)
Taiwan	10 142<br>(9205 – 10 959)
Oceania	..
American Samoa	36<br>(33 – 39)
Federated States of Micronesia	38<br>(32 – 44)
Fiji	329<br>(270 – 385)
Guam	76<br>(68 – 83)
Kiribati	38<br>(28 – 46)
Marshall Islands	34<br>(29 – 38)
Northern Mariana Islands	66<br>(61 – 71)
Papua New Guinea	3320<br>(2553 – 3936)
Samoa	102<br>(93 – 110)
Solomon Islands	273<br>(236 – 307)
Tonga	47<br>(42 – 52)
Vanuatu	121<br>(102 – 137)
Southeast Asia	..
Cambodia	7143<br>(6520 – 7734)
Indonesia	100 690<br>(91 722 – 108 652)
Laos	3021<br>(2046 – 3694)
Malaysia	13 727<br>(12 748 – 14 710)
Maldives	198<br>(185 – 210)
Mauritius	597<br>(544 – 651)
Myanmar	22 284<br>(19 824 – 24 405)
Philippines	46 552<br>(42 667 – 50 337)
Sri Lanka	11 066<br>(10 194 – 11 842)
Seychelles	49<br>(45 – 53)
Thailand	34 515<br>(32 191 – 36 954)
Timor-Leste	563<br>(488 – 630)
Vietnam	48 779<br>(45 501 – 52 008)
Sub-Saharan Africa	..
Central sub-Saharan Africa	..
Angola	13 171<br>(10 504 – 15 534)
Central African Republic	1741<br>(941 – 2375)
Congo (Brazzaville)	1948<br>(1576 – 2292)
Democratic Republic of the Congo	41 529<br>(30 229 – 51 659)
Equatorial Guinea	360<br>(290 – 423)
Gabon	725<br>(617 – 830)

Eastern sub-Saharan Africa	..
Burundi	5951<br>(4351 – 7096)
Comoros	322<br>(283 – 358)
Djibouti	464<br>(380 – 537)
Eritrea	2241<br>(1928 – 2513)
Ethiopia	51 689<br>(47 071 – 56 402)
Kenya	20 881<br>(19 301 – 22 460)
Madagascar	12 367<br>(10 549 – 14 116)
Malawi	9556<br>(8163 – 10 797)
Mozambique	15 142<br>(13 062 – 17 143)
Rwanda	5982<br>(5324 – 6553)
Somalia	3314<br>(2375 – 4121)
South Sudan	7786<br>(6058 – 9295)
Tanzania	26 453<br>(23 593 – 29 131)
Uganda	20 136<br>(17 817 – 22 151)
Zambia	6896<br>(5681 – 7999)
Southern sub-Saharan Africa	..
Botswana	1039<br>(870 – 1222)
Lesotho	780<br>(540 – 994)
Namibia	1095<br>(922 – 1246)
South Africa	20 994<br>(18 434 – 23 295)
Swaziland (eSwatini)	561<br>(443 – 666)
Zimbabwe	6763<br>(5818 – 7632)
Western sub-Saharan Africa	..
Benin	5710<br>(5006 – 6380)
Burkina Faso	9941<br>(7779 – 12 070)
Cameroon	9550<br>(7513 – 11 370)
Cape Verde	306<br>(284 – 327)
Chad	6869<br>(5738 – 7883)
Cote d'Ivoire	9381<br>(7460 – 11 099)
The Gambia	1087<br>(974 – 1193)
Ghana	12 951<br>(11 408 – 14 343)
Guinea	5767<br>(4820 – 6603)
Guinea-Bissau	807<br>(699 – 907)
Liberia	2539<br>(2282 – 2764)
Mali	11 173<br>(9555 – 12 637)

Mauritania	1885<br>(1701 – 2050)
Niger	12 663<br>(10 787 – 14 188)
Nigeria	87 294<br>(73 106 – 100 119)
Sao Tome and Principe	100<br>(91 – 108)
Senegal	7705<br>(7041 – 8341)
Sierra Leone	2920<br>(2483 – 3294)
Togo	3362<br>(2902 – 3784)

7.3. Identifying the UHC standard for utilisation

To identify our UHC standard of utilisation of personal health services, we calculated the total cost of attaining the standard using each country as the standard. When countries with lower utilisation per DALY were the standard, the total cost decreased for two reasons: fewer additional services were needed, and the service-age-sex results in fewer countries did not meet the standard. We created a scatter plot with the GBD 2016 UHC index and logarithm of total cost of attaining the standard (Figure S13). Countries to the left represented lower total cost to meet the UHC standard for a given UHC index score.

Six countries stood out for their combination of low cost for achieving a UHC standard for utilisation and high score on the UHC index: four in the highest quintile of the UHC index (Switzerland, Finland, Sweden, and the Netherlands), and two in the second quintile (Portugal, and Lebanon). Comparing the health system statistics, the ratio of total volume of visits to total counterfactual DALYs ranged from 7.88 in Switzerland to 5.16 in Lebanon, a 35% difference (Table S17). There was a broader range for inpatient admissions; the ratio of total volume of admissions to total counterfactual DALYs ranged from 0.24 in Switzerland to 0.10 in Lebanon, a 58% difference. Among the countries on the frontier, the utilisation of inpatient services distinguished the countries with higher costs for achieving a UHC standard and higher scores on the UHC index from the others.

Figure S13. Total cost to reach universal health coverage using each country as a standard, by Socio-demographic Index quintile

The total cost to reach universal health coverage (UHC) was calculated for all countries using each country as a standard. Countries that were above the standard with respect to outpatient and inpatient utilisation were not included in the total cost calculation for that standard. Results are plotted against UHC index. Countries with relatively low costs to reach UHC for a given UHC index are labelled.

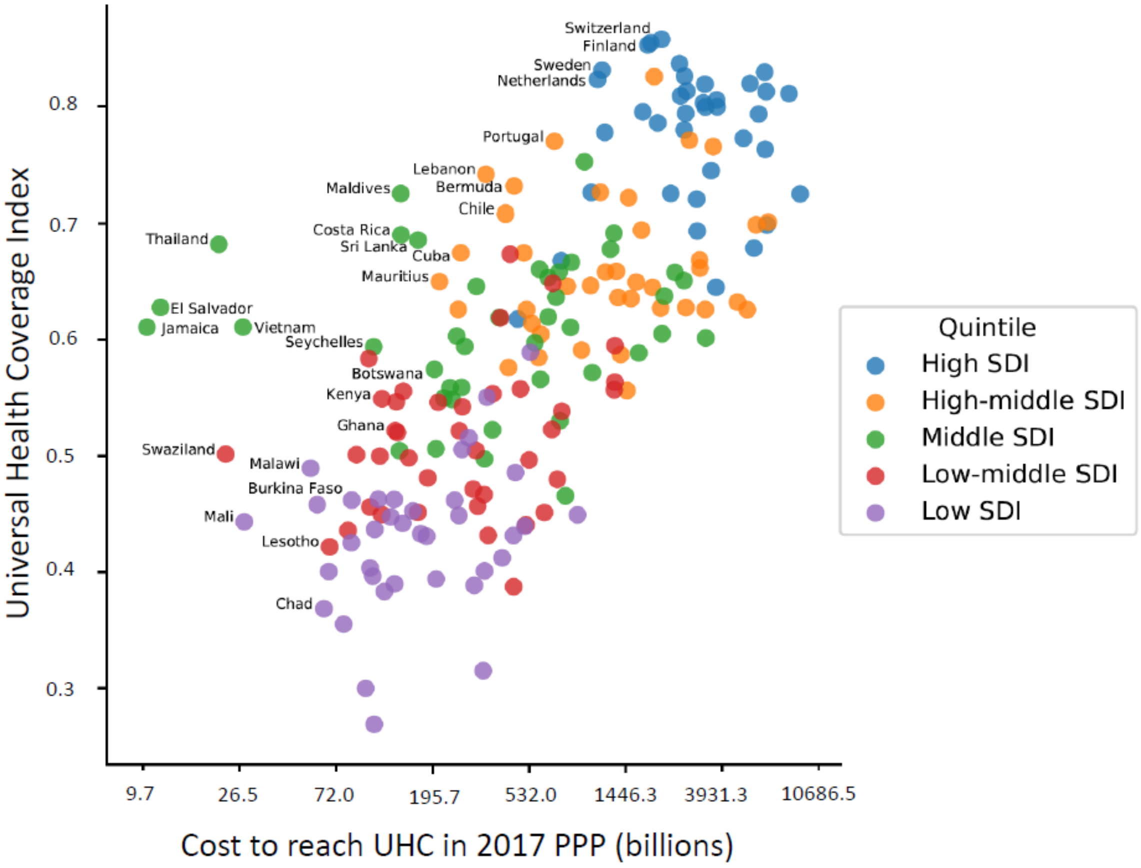

Table S17. Preliminary estimates of funds needed to achieve universal health coverage in 2016 using utilisation per DALY of six countries by World Bank income group in 2017 international dollars

Total additional cost of reaching the UHC standard for 188 countries was calculated with utilisation per DALY of for six countries with a high scores on the UHC index, and low total additional cost estimates. DALY=disability-adjusted-life-year, UHC=Universal Health Coverage

					Total additional cost to meet UHC standard for utilisation in billions of international dollars by World Bank income group				
**Country**	**GBD 2016 UHC index**	**Country rank by GBD 2016 UHC index**	**Ratio of total visits to counterfactual DALYs**	**Ratio of total admis-sions to counterfactual DALYs**	**Low income**	**Lower middle income**	**Upper middle income**	**High income**	**Total**
Switzerland	0.86	1	7.88	0.24	69.01	692.65	757.12	798.23	2317.01
Finland	0.85	3	7.16	0.24	60.39	616.83	632.97	669.45	1979.65
Sweden	0.83	5	5.70	0.19	51.58	488.72	391.77	313.55	1245.62
Netherlands	0.82	9	7.25	0.17	47.09	456.04	408.36	266.21	1177.69
Portugal	0.77	29	7.01	0.14	34.72	336.02	278.14	96.71	745.59
Lebanon	0.74	34	5.16	0.10	17.05	176	105.15	59.41	357.6

Estimates of additional cost of reaching the UHC standard for utilisation among the six countries were ranked from highest (2017 international $2,317 billion) to lowest (I$357 billion). Within this range, much of difference reflected the high unit costs in high income countries, where the cost was 13-fold higher in Switzerland (I$798 billion) than Lebanon (I$59 billion). In low income countries, the cost were four times higher in Switzerland (I$69 billion) than Lebanon (I$18 billion). In estimates using the utilisation per DALY from two countries in the middle of the range (Sweden and Netherlands) the range of costs was narrower; the cost in low-income countries ranged from I$51 to I$47 billion, lower-middle income countries ranged from I$489 to I$456 billion, upper middle income countries from I$408 to I$392 billion, and high income countries from I$314 to I$266 billion.

The Netherlands, in the middle of the range, was selected as the standard with a ratio of 7.25 total visits to total counterfactual DALYs, and 0.17 total admissions to total counterfactual DALYs. The key to our method was to select a standard that represented the performance of a country rather than an ideal. Other countries could arguably have been selected. Portugal in the second quintile of the UHC index would serve as an intermediate UHC standard for utilisation with a smaller ratio of admissions to counterfactual DALYs and lower additional cost of inpatient admissions, and we conducted a sensitivity analysis using it as a standard. Switzerland with the highest score on the UHC index would serve as an extreme UHC standard, implying that only one country with the highest score on the UHC index had universal coverage.

The ratios for each service-age-sex in the Netherlands (Table S18) were used in the UHC calculations, rather than the aggregate ratio:

$$Cost_{ijkl} = \left(\left(\frac{volume_{Netherlandsjkl}}{DALY0s_{Netherlandskl}}\right) - \left(\frac{volume_{ijkl}}{DALY0s_{ikl}}\right) \right) * DALY0s_{ikl} * unitcost_{ij} \quad (9)$$

where *DALY*0 denotes the counterfactual DALYs with health access and quality removed, *i* refers country, *j* to the service (outpatient or inpatient), *k* to age category, and *l* to sex. The ratio of counterfactual DALYs to actual DALYs was highest for children 0 to 4 years of age, meaning that health service access and quality reduced DALYs for these age categories. Conversely the ratio of utilisation to counterfactual DALY was lowest for them, meaning that health services had a large effect on the burden of disease.

The ratio of volume to counterfactual DALYs varied across locations and age categories. Comparing the ratios for the Netherlands to the summary results by GBD super-regions shows the size of the gaps by locations and age categories (Figures S14 and S15). The highest ratios were in the Central Europe, Eastern Europe, and Central Asia, and High Income super-regions, and lowest were in the South Asia and sub-Saharan Africa super-regions. Across age categories, the highest ratios were among children 5 to 9 years of age. The ratio of admissions per counterfactual DALY was also high among women of child-bearing age. Note that countries with relatively high aggregate ratios of total utilisation to counterfactual DALY still had gaps for specific service-age-sex ratios.

Table S18. Inpatient and outpatient utilisation per counterfactual disability-adjusted-life-year by age and sex for the Netherlands

Counterfactual DALYs were estimated using a linear regression model to predict disability-adjusted-life-years (DALYs) in a scenario where the Health Access and Quality index was 0. The linear regression model included two covariates: Socio-demographic Index and HAQ index over HAQ frontier. The HAQ index frontier represents the highest expected HAQ to be achieved at a given SDI level for a given age-sex group. Using these counterfactual DALY estimates, we calculated total outpatient and inpatient visits per counterfactual DALY by age and sex for the Netherlands. We also calculated the ratio of counterfactual DALYs to actual DALYs, or DALYs reported in the Global Burden of Disease 2016. OP = outpatient, IP= inpatient, DALY = disability-adjusted-life-year.

Age Group Name	Sex	OP utilisation per counterfactual DALY	IP utilisation per counterfactual DALY	counterfactual DALYs/actual DALYs
Early neonatal	male	0.039	0.003	13.764
	female	0.068	0.003	12.850
Late neonatal	male	0.401	0.028	11.628
	female	0.579	0.025	12.037
Post neonatal	male	1.473	0.095	27.919
	female	2.301	0.094	29.348
1 to 4	male	3.720	0.147	27.115
	female	5.660	0.139	27.284
5 to 9	male	19.387	0.456	4.838
	female	33.249	0.477	4.469
10 to 14	male	15.357	0.269	3.568
	female	27.057	0.316	3.112
15 to 19	male	12.516	0.222	2.773
	female	26.554	0.376	2.091
20 to 24	male	12.285	0.228	2.454
	female	22.351	0.413	2.148
25 to 29	male	8.162	0.154	3.388
	female	15.765	0.330	2.752
30 to 34	male	5.720	0.116	4.332
	female	11.964	0.245	3.292
35 to 39	male	4.766	0.100	5.098
	female	9.665	0.177	3.698
40 to 44	male	4.595	0.096	5.168
	female	8.944	0.143	3.580
45 to 49	male	4.600	0.099	4.799
	female	8.560	0.123	3.348
50 to 54	male	4.551	0.104	4.215
	female	7.851	0.107	3.227
55 to 59	male	4.607	0.124	3.504
	female	7.742	0.125	2.968
60 to 64	male	4.852	0.143	2.885

	female	7.709	0.155	2.721
65 to 69	male	5.060	0.156	2.398
	female	7.211	0.186	2.610
70 to 74	male	5.440	0.205	1.945
	female	6.959	0.210	2.366
75 to 79	male	8.754	0.411	1.000
	female	13.922	0.460	1.000
80 to 84	male	7.236	0.381	1.000
	female	11.250	0.424	1.000
85 to 89	male	6.442	0.344	1.000
	female	9.044	0.369	1.000
90 to 94	male	5.856	0.332	1.000
	female	7.445	0.336	1.000
95+	male	5.793	0.355	1.000
	female	6.547	0.335	1.000

Figure S14. Global and GBD super-region estimates of the volume of outpatient visits per counterfactual disability-adjusted-life-year by age, sex, 2016

Counterfactual DALYs were estimated using a linear regression model to predict disability-adjusted-life-years (DALYs) in a scenario where the Health Access and Quality index was 0. The linear regression model included two covariates: Socio-demographic Index and HAQ index over HAQ frontier. The HAQ index frontier represents the highest expected HAQ to be achieved at a given SDI level for a given age-sex group. Using these counterfactual DALY estimates, we calculated total outpatient visits per counterfactual DALY for each age-sex group globally and for each GBD super-region. OP = outpatient, DALY = disability-adjusted-life-year.

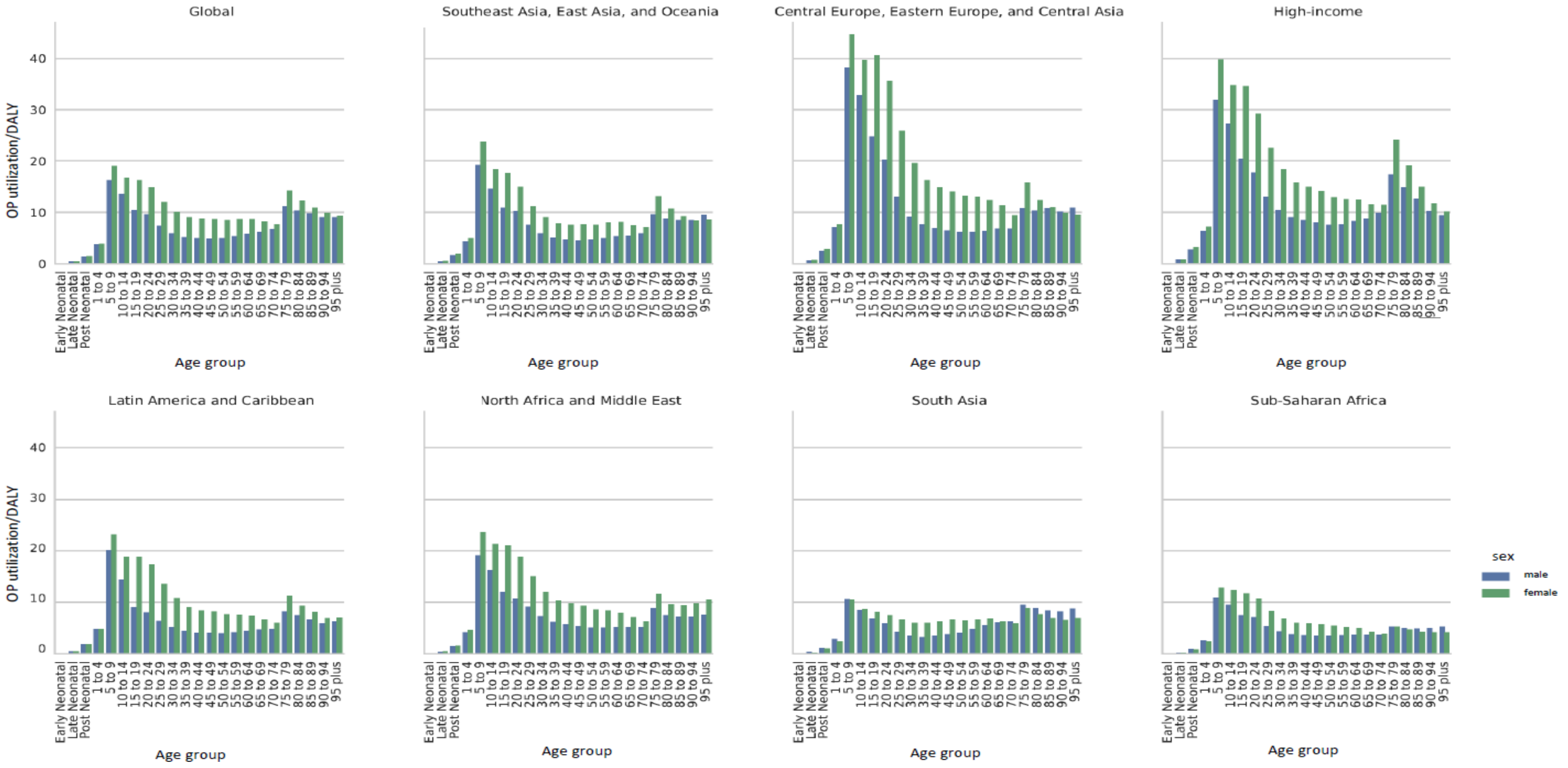

Figure S15. Global and GBD super-region estimates of the volume of outpatient visits per counterfactual disability-adjusted-life-year by age, sex, 2016

Counterfactual DALYs were estimated using a linear regression model to predict disability-adjusted-life-years (DALYs) in a scenario where the Health Access and Quality index was 0. The linear regression model included two covariates: Socio-demographic Index and HAQ index over HAQ frontier. The HAQ index frontier represents the highest expected HAQ to be achieved at a given SDI level for a given age-sex group. Using these counterfactual DALY estimates, we calculated total inpatient admissions per counterfactual DALY for each age-sex group globally and for each GBD super-region. IP = inpatient, DALY = disability-adjusted-life-year.

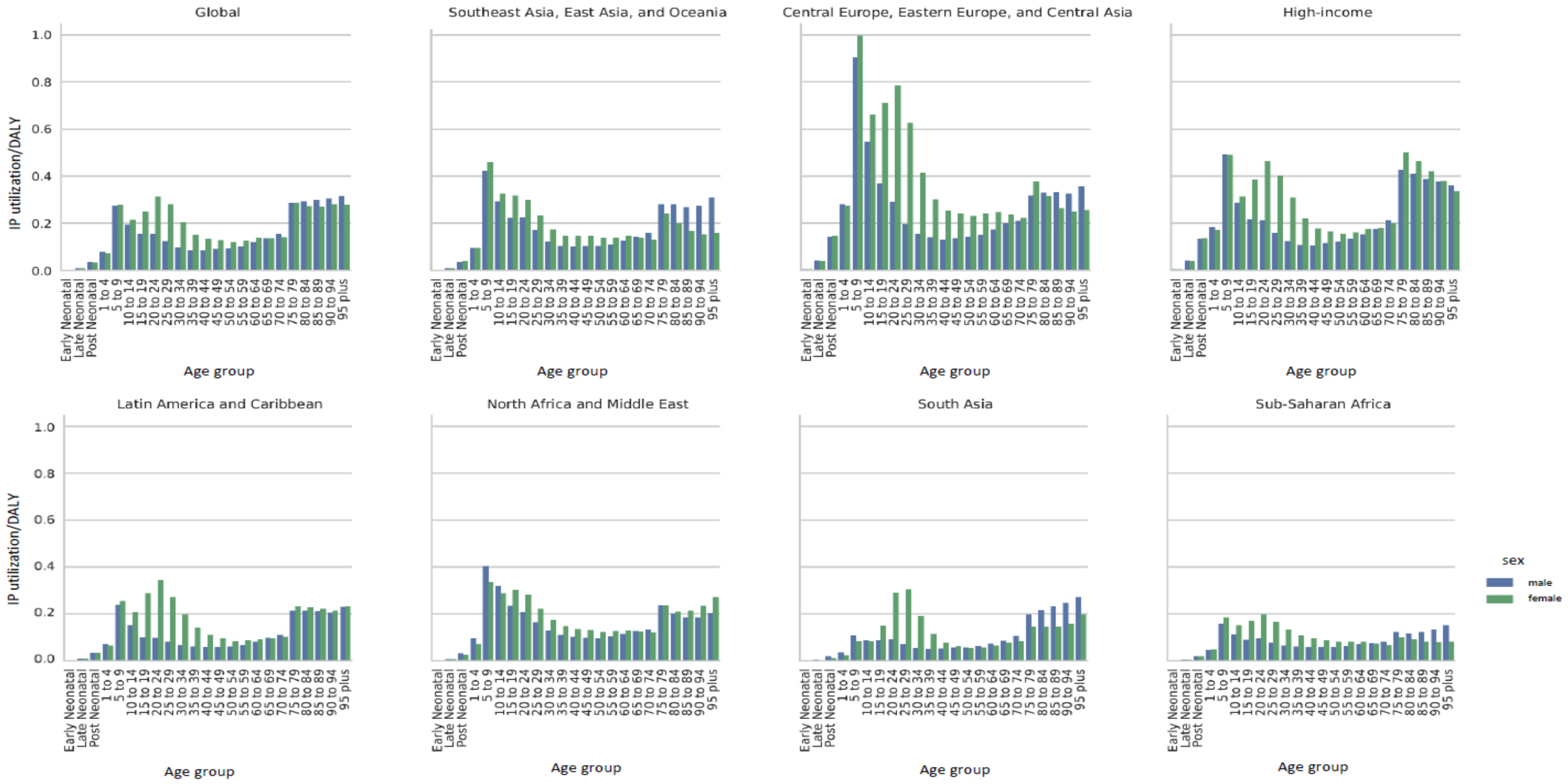

7.4. Main analysis

The costs with the Netherlands as the standard were estimated in international dollars (Table 1) and United States dollars (Table S19). Unit costs that are less for US$0.50 are reported as zero.

Table S19. National unit costs of outpatient visits and inpatient admissions, utilisation per counterfactual disability-adjusted-life-year, and additional visits, admissions, and funds needed to achieve a Universal Health Coverage standard for utilisation in 2016 in 2017 United States dollars

Table displays four sets of national estimates organised by GBD region: 1) cost per outpatient visit and inpatient admission by country in 2016 in 2017 United States dollars, 2) aggregate ratio of total outpatient visits and inpatient admissions to counterfactual DALYs, where the counterfactual DALYs standardised the burden of disease across countries by removing the effects of access and quality of health care, 3) estimates of additional services needed to achieve the UHC standard for utilisation calculated by age and sex category, and 4) total cost of additional services in 2017 United States dollars. The cost of scaling up to the UHC utilisation standard was done at the age-sex level, leading some countries to have a cost of UHC scale-up greater than zero despite their aggregate utilisation per counterfactual DALY being greater than the standard set by the Netherlands. For each result, the mean of 1,000 draws is reported, and in parenthesis the uncertainty interval defined as the 2.5th and 97.5th percentile of draws. DALY = disability-adjusted-life-year. GBD= Global Burden of Disease, UHC = Universal Health Coverage.

Table S19: National unit costs of outpatient visits and inpatient admissions, utilisation per counterfactual disability-adjusted-life-year, and additional visits, admissions, and funds needed to achieve a Universal Health Coverage standard for utilisation in 2016 in 2017 United States dollars

	Outpatient visits			Inpatient admissions			Total additional cost to meet UHC standards in millions 2017 $US (95% UI)
	Unit cost per outpatient visit in 2017 US$ (95% UI)	Outpatient utilization per DALY (95% UI) (Table 1)	Additional outpatient visits to meet UHC standard (95% UI) (in thousands) (Table 1)	Unit cost per inpatient admission in 2017 US$ (95% UI)	Inpatient utilization per DALY (95% UI) (Table 1)	Additional inpatient admissions to meet UHC standard (95% UI) (in thousands) (Table 1)	
Global	‥	6·7 (6·04 – 7·41)	10 415 092 (7 809 849 – 12 742 483)	‥	0·12 (0·11 – 0·13)	348 465 (306 581 – 383 242)	575 567 (413 343 – 739 587)
Low income	‥	‥	1 956 058 (1 552 914 – 2 280 749)	‥	‥	61 106 (54 415 – 66 067)	16 200 (12 767 – 19 362)
Lower middle income	‥	‥	6 224 613 (4 643 059 – 7 683 756)	‥	‥	214 966 (194 755 – 231 539)	141 901 (113 480 – 171 287)
Upper middle income	‥	‥	2 023 229 (1 418 055 – 2 614 556)	‥	‥	59 265 (46 625 – 70 696)	176 598 (135 310 – 216 521)
High income	‥	‥	211 192 (83 151 – 330 668)	‥	‥	13 128 (6499 – 18 933)	240 867 (135 989 – 343 474)
Central Europe, Eastern Europe, and Central Asia	‥	10·82 (10·37 – 11·31)	116 530 (86 387 – 145 714)	‥	0·24 (0·23 – 0·25)	4372 (2820 – 6017)	4534 (3284 – 5949)
Central Asia	‥	8·25 (7·95 – 8·54)	54 694 (48 267 – 61 347)	‥	0·17 (0·17 – 0·18)	1745 (1556 – 1936)	1824 (1616 – 2031)
Armenia	19 (16 – 23)	4·86 (4·67 – 5·05)	5691 (5076 – 6307)	691 (574 – 812)	0·16 (0·16 – 0·17)	63 (51 – 75)	155 (124 – 188)
Azerbaijan	14 (12 – 16)	5·8 (5·53 – 6·08)	9910 (8076 – 11 755)	1093 (960 – 1241)	0·08 (0·08 – 0·09)	581 (539 – 621)	776 (660 – 901)
Georgia	11 (9 – 14)	7·13 (6·13 – 8·43)	1736 (359 – 3125)	880 (775 – 991)	0·11 (0·1 – 0·11)	209 (185 – 232)	206 (165 – 251)
Kazakhstan	12 (11 – 13)	8·27 (7·91 – 8·65)	2937 (1224 – 4814)	579 (526 – 633)	0·19 (0·18 – 0·2)	217 (163 – 277)	162 (118 – 214)
Kyrgyzstan	5 (5 – 6)	4·06 (3·91 – 4·22)	14 137 (12 960 – 15 301)	159 (142 – 179)	0·17 (0·17 – 0·18)	83 (60 – 103)	89 (77 – 103)
Mongolia	6 (5 – 7)	6·39 (5·38 – 7·61)	1669 (388 – 2868)	253 (200 – 314)	0·18 (0·15 – 0·22)	32 (14 – 48)	19 (9 – 29)
Tajikistan	2 (2 – 2)	5·69 (5·44 – 5·96)	8781 (7148 – 10 447)	103 (92 – 115)	0·15 (0·15 – 0·16)	162 (130 – 192)	36 (29 – 43)
Turkmenistan	30 (26 – 35)	4·81 (4·6 – 5·02)	9620 (8440 – 10 757)	847 (661 – 1033)	0·2 (0·16 – 0·24)	44 (16 – 66)	330 (269 – 397)
Uzbekistan	2 (2 – 2)	11·85 (11·37 – 12·32)	212 (-318 – 764)	146 (132 – 161)	0·21 (0·2 – 0·22)	353 (275 – 431)	52 (39 – 65)
Central Europe	‥	10·21 (9·83 – 10·6)	25 786 (19 317 – 32 104)	‥	0·25 (0·23 – 0·27)	737 (344 – 1109)	1439 (919 – 1986)
Albania	13 (11 – 16)	6·98 (5·92 – 8·13)	1189 (-163 – 2405)	906 (807 – 1021)	0·12 (0·11 – 0·12)	129 (110 – 147)	134 (100 – 168)
Bosnia and Herzegovina	10 (8 – 11)	12·28 (10·83 – 14·43)	0 (0 – 0)	1004 (823 – 1208)	0·14 (0·12 – 0·16)	115 (36 – 185)	119 (32 – 220)
Bulgaria	26 (23 – 28)	7·17 (6·7 – 7·63)	3478 (1720 – 5113)	596 (546 – 646)	0·35 (0·33 – 0·37)	0 (0 – 0)	89 (44 – 134)
Croatia	36 (33 – 40)	8·06 (7·74 – 8·42)	965 (417 – 1483)	1434 (1293 – 1593)	0·23 (0·21 – 0·24)	41 (18 – 64)	94 (45 – 144)
Czech Republic	34 (32 – 37)	14·1 (13·48 – 14·84)	0 (0 – 0)	1630 (1368 – 1906)	0·32 (0·28 – 0·38)	0 (0 – 0)	0 (0 – 0)
Hungary	20 (19 – 22)	15·6 (15·03 – 16·24)	0 (0 – 0)	1344 (1230 – 1465)	0·26 (0·24 – 0·28)	24 (-35 – 81)	33 (-45 – 111)
Macedonia	11 (10 – 13)	8·32 (7·93 – 8·72)	469 (321 – 613)	730 (627 – 878)	0·15 (0·14 – 0·16)	57 (38 – 74)	47 (30 – 65)
Montenegro	13 (12 – 15)	9·39 (8·79 – 9·95)	20 (-21 – 60)	919 (838 – 1001)	0·16 (0·15 – 0·17)	14 (10 – 17)	13 (9 – 17)
Poland	28 (26 – 30)	10·21 (9·78 – 10·63)	0 (0 – 0)	1341 (1221 – 1467)	0·24 (0·22 – 0·25)	77 (-95 – 246)	104 (-122 – 333)
Romania	24 (22 – 27)	6·11 (5·83 – 6·38)	19 296 (15 002 – 23 587)	572 (505 – 643)	0·29 (0·27 – 0·32)	10 (-60 – 74)	471 (340 – 607)
Serbia	16 (15 – 18)	9·3 (8·93 – 9·65)	368 (-307 – 987)	1180 (1063 – 1306)	0·15 (0·14 – 0·16)	263 (204 – 320)	318 (239 – 408)
Slovakia	25 (23 – 28)	16·97 (16·28 – 17·65)	0 (0 – 0)	1710 (1547 – 1890)	0·28 (0·26 – 0·29)	5 (-6 – 15)	8 (-11 – 26)
Slovenia	67 (62 – 71)	9·87 (9·49 – 10·25)	0 (0 – 0)	2913 (2668 – 3151)	0·25 (0·23 – 0·26)	3 (-3 – 8)	8 (-8 – 25)
Eastern Europe	‥	12·12 (11·51 – 12·8)	36 050 (12 837 – 57 240)	‥	0·26 (0·25 – 0·27)	1889 (666 – 3266)	1271 (462 – 2180)
Belarus	6 (6 – 7)	14·85 (14·32 – 15·42)	0 (0 – 0)	370 (307 – 446)	0·29 (0·24 – 0·35)	54 (-74 – 149)	20 (-28 – 59)
Estonia	40 (37 – 43)	10·29 (9·92 – 10·66)	0 (0 – 0)	1854 (1697 – 2023)	0·24 (0·23 – 0·26)	11 (5 – 17)	20 (9 – 31)
Latvia	35 (32 – 38)	8·75 (8·34 – 9·17)	737 (485 – 985)	1441 (1310 – 1571)	0·24 (0·23 – 0·25)	16 (6 – 27)	49 (27 – 72)

Lithuania	27 (25 – 29)	12·3 (11·85 – 12·79)	0 (0 – 0)	1112 (1008 – 1220)	0·33 (0·31 – 0·35)	1 (-1 – 2)	1 (-1 – 2)
Moldova	6 (6 – 7)	7·79 (7·48 – 8·11)	1723 (1241 – 2196)	313 (278 – 354)	0·2 (0·19 – 0·21)	70 (56 – 84)	33 (26 – 41)
Russia	12 (11 – 13)	13·75 (13·08 – 14·58)	0 (0 – 0)	710 (661 – 763)	0·26 (0·25 – 0·27)	1237 (248 – 2429)	878 (172 – 1767)
Ukraine	5 (4 – 6)	7·02 (6·07 – 8·37)	33 590 (10 504 – 54 226)	170 (153 – 188)	0·26 (0·24 – 0·27)	501 (143 – 896)	270 (106 – 454)
High-income	..	11·79 (10·78 – 12·88)	249 199 (94 015 – 391 261)	..	0·2 (0·18 – 0·21)	12 493 (5931 – 18 151)	235 481 (131 760 – 339 981)
Australasia	..	11·79 (11·34 – 12·25)	0 (0 – 0)	..	0·24 (0·22 – 0·26)	45 (6 – 78)	307 (21 – 553)
Australia	169 (162 – 177)	11·95 (11·49 – 12·43)	0 (0 – 0)	8050 (7310 – 8865)	0·24 (0·22 – 0·26)	4 (-31 – 33)	34 (-225 – 272)
New Zealand	135 (123 – 149)	10·93 (10·16 – 11·63)	0 (0 – 0)	6628 (6115 – 7173)	0·21 (0·21 – 0·22)	41 (28 – 55)	273 (176 – 362)
High-income Asia-Pacific	..	22·92 (20·6 – 24·92)	0 (0 – 0)	..	0·18 (0·16 – 0·21)	3195 (626 – 5512)	30 556 (4997 – 59 443)
Brunei	27 (22 – 33)	15·62 (13·29 – 19·64)	0 (0 – 0)	2106 (1672 – 2608)	0·19 (0·15 – 0·22)	3 (0 – 5)	6 (-1 – 13)
Japan	68 (60 – 78)	22·42 (19·46 – 24·99)	0 (0 – 0)	9252 (7636 – 11 110)	0·16 (0·13 – 0·19)	3169 (642 – 5453)	30 415 (4991 – 59 048)
Singapore	48 (39 – 57)	22·68 (19·12 – 27·38)	0 (0 – 0)	5463 (4493 – 6602)	0·18 (0·15 – 0·21)	23 (-22 – 60)	135 (-116 – 393)
South Korea	38 (36 – 40)	24·33 (23·24 – 25·44)	0 (0 – 0)	3662 (3371 – 3948)	0·24 (0·23 – 0·26)	0 (0 – 0)	0 (0 – 0)
High-income North America	..	8·79 (7·79 – 9·87)	119 433 (27 473 – 197 248)	..	0·17 (0·15 – 0·18)	5014 (2949 – 6919)	166 715 (92 510 – 240 972)
Canada	146 (137 – 155)	12·99 (12·23 – 13·69)	0 (0 – 0)	9425 (7861 – 11 248)	0·19 (0·16 – 0·23)	302 (97 – 465)	2882 (572 – 5021)
Greenland	..	8·7 (7·52 – 10·28)	15 (-4 – 29)	..	0·15 (0·12 – 0·18)	1 (1 – 1)	0 (0 – 0)
United States	480 (415 – 548)	8·33 (7·25 – 9·52)	119 418 (27 474 – 197 217)	22 529 (20 547 – 24 392)	0·17 (0·15 – 0·18)	4710 (2677 – 6590)	163 833 (90 562 – 237 439)
Southern Latin America	..	5·92 (5·32 – 6·71)	77 103 (38 426 – 104 635)	..	0·12 (0·1 – 0·14)	2402 (1510 – 3199)	12 324 (8276 – 16 356)
Argentina	49 (41 – 58)	6·1 (5·28 – 7·18)	47 282 (12 243 – 72 301)	2412 (2001 – 2883)	0·12 (0·1 – 0·14)	1529 (822 – 2167)	6164 (3279 – 9215)
Chile	77 (73 – 81)	5·48 (5·31 – 5·68)	26 005 (22 813 – 29 141)	3497 (3079 – 3940)	0·12 (0·11 – 0·14)	618 (397 – 806)	4208 (3174 – 5250)
Uruguay	82 (70 – 97)	6·08 (5·29 – 7·06)	3816 (1301 – 5880)	6332 (5234 – 7516)	0·08 (0·07 – 0·09)	255 (216 – 288)	1952 (1366 – 2520)
Western Europe	..	10·65 (9·86 – 11·58)	52 663 (6711 – 97 539)	..	0·24 (0·22 – 0·25)	1837 (233 – 3139)	25 579 (8778 – 43 286)
Andorra	208 (175 – 242)	10·72 (9·35 – 12·3)	0 (0 – 0)	8505 (6936 – 10 364)	0·24 (0·2 – 0·29)	0 (0 – 0)	0 (0 – 0)
Austria	170 (163 – 178)	11·15 (10·71 – 11·61)	29 (-257 – 308)	4876 (4591 – 5155)	0·37 (0·35 – 0·39)	0 (0 – 0)	7 (-52 – 63)
Belgium	133 (119 – 147)	13·54 (12·39 – 15·04)	0 (0 – 0)	7138 (6627 – 7660)	0·24 (0·22 – 0·26)	5 (-15 – 22)	39 (-105 – 158)
Cyprus	79 (67 – 93)	8·51 (7·29 – 9·82)	246 (-39 – 524)	4278 (3551 – 5126)	0·15 (0·13 – 0·18)	16 (-1 – 31)	91 (12 – 178)
Denmark	292 (279 – 307)	7·82 (7·55 – 8·09)	885 (280 – 1498)	9984 (9345 – 10 618)	0·22 (0·21 – 0·23)	2 (-5 – 9)	273 (53 – 505)
Finland	234 (222 – 245)	7·16 (6·84 – 7·47)	1777 (908 – 2645)	6763 (6295 – 7243)	0·24 (0·22 – 0·25)	0 (-1 – 1)	425 (212 – 654)
France	154 (147 – 160)	11·44 (11·01 – 11·89)	0 (0 – 0)	5937 (5456 – 6371)	0·28 (0·26 – 0·31)	0 (0 – 0)	0 (0 – 0)
Germany	138 (124 – 152)	13·86 (12·63 – 15·39)	0 (0 – 0)	5267 (4933 – 5602)	0·34 (0·33 – 0·36)	0 (0 – 0)	0 (0 – 0)
Greece	83 (69 – 99)	6·75 (5·79 – 7·93)	7123 (1258 – 12 279)	2156 (1775 – 2611)	0·25 (0·21 – 0·3)	0 (0 – 0)	614 (90 – 1188)
Iceland	229 (216 – 244)	10·14 (9·69 – 10·57)	0 (0 – 0)	11 911 (11 014 – 12 818)	0·18 (0·17 – 0·2)	2 (1 – 3)	24 (11 – 37)
Ireland	156 (140 – 173)	13·09 (12·21 – 13·88)	0 (0 – 0)	9340 (8358 – 10 401)	0·21 (0·19 – 0·22)	0 (-2 – 3)	4 (-23 – 29)
Israel	89 (76 – 103)	12·32 (10·77 – 14·4)	0 (0 – 0)	4352 (4037 – 4682)	0·24 (0·23 – 0·26)	0 (0 – 0)	0 (0 – 0)
Italy	85 (75 – 94)	12·73 (11·46 – 14·42)	0 (0 – 0)	5967 (5439 – 6484)	0·17 (0·16 – 0·19)	546 (-33 – 1077)	3306 (-104 – 6833)
Luxembourg	272 (251 – 296)	10·79 (10·34 – 11·26)	0 (0 – 0)	12 125 (10 974 – 13 369)	0·22 (0·21 – 0·24)	0 (0 – 0)	0 (-1 – 1)
Malta	51 (44 – 59)	18·82 (16·05 – 21·55)	0 (0 – 0)	4902 (4068 – 5923)	0·19 (0·15 – 0·23)	1 (-3 – 4)	4 (-14 – 20)
Netherlands	282 (254 – 310)	7·25 (6·67 – 7·89)	0 (0 – 0)	11 153 (9659 – 12 740)	0·17 (0·15 – 0·2)	0 (0 – 0)	0 (0 – 0)
Norway	459 (432 – 488)	7·21 (6·92 – 7·51)	1275 (374 – 2084)	11 188 (10 435 – 12 043)	0·27 (0·26 – 0·29)	0 (-2 – 3)	601 (232 – 999)
Portugal	93 (85 – 103)	7·01 (6·43 – 7·52)	4178 (1393 – 7291)	4394 (4066 – 4748)	0·14 (0·14 – 0·15)	291 (215 – 372)	1679 (1178 – 2219)
Spain	92 (81 – 104)	10·6 (9·45 – 11·9)	0 (0 – 0)	5931 (5591 – 6266)	0·16 (0·15 – 0·17)	602 (366 – 842)	3577 (2090 – 5177)

Sweden	413 (351 – 478)	5·7 (4·87 – 6·63)	11 494 (4594 – 17 687)	11 541 (9636 – 13 633)	0·19 (0·16 – 0·23)	61 (-91 – 179)	5598 (1812 – 9541)
Switzerland	502 (465 – 539)	7·88 (7·36 – 8·41)	2280 (458 – 3955)	15 670 (14 607 – 16 767)	0·24 (0·22 – 0·25)	0 (0 – 0)	1159 (249 – 2060)
United Kingdom	218 (189 – 250)	7·01 (6·1 – 8·07)	23 375 (-7052 – 52 671)	8043 (6882 – 9264)	0·18 (0·16 – 0·21)	311 (-547 – 945)	8178 (-1378 – 19 119)
Latin America and Caribbean	‥	6·47 (5·79 – 7·22)	615 322 (410 489 – 803 049)	‥	0·1 (0·09 – 0·11)	28 433 (23 133 – 32 514)	81 228 (61 130 – 100 515)
Andean Latin America	‥	7·78 (6·65 – 9·5)	11 992 (-10962 – 30 524)	‥	0·1 (0·09 – 0·11)	2869 (2402 – 3267)	3895 (2850 – 4898)
Bolivia	11 (8 – 13)	7·42 (6·21 – 9·26)	4481 (-1078 – 9010)	693 (557 – 837)	0·1 (0·08 – 0·12)	542 (432 – 638)	430 (278 – 582)
Ecuador	30 (23 – 36)	7·86 (6·57 – 9·5)	2906 (-4405 – 8852)	1802 (1574 – 2057)	0·1 (0·1 – 0·11)	810 (759 – 863)	1556 (1233 – 1886)
Peru	18 (14 – 21)	7·86 (6·72 – 9·76)	4605 (-8218 – 15 468)	1186 (952 – 1437)	0·09 (0·08 – 0·11)	1518 (1145 – 1831)	1909 (1184 – 2662)
Caribbean	‥	4·26 (3·66 – 4·9)	113 711 (93 323 – 132 567)	‥	0·1 (0·08 – 0·11)	2427 (1909 – 2836)	5893 (4349 – 7552)
Antigua and Barbuda	69 (57 – 84)	5·29 (4·47 – 6·18)	157 (99 – 209)	2862 (2268 – 3494)	0·1 (0·08 – 0·12)	4 (3 – 5)	24 (16 – 32)
The Bahamas	146 (119 – 177)	6·13 (5·18 – 7·21)	423 (194 – 609)	5270 (4138 – 6559)	0·13 (0·11 – 0·16)	12 (8 – 16)	129 (73 – 182)
Barbados	151 (125 – 181)	3·34 (2·85 – 3·86)	887 (770 – 997)	2566 (2046 – 3134)	0·15 (0·13 – 0·18)	6 (2 – 10)	151 (110 – 195)
Belize	31 (24 – 38)	3·81 (3·19 – 4·5)	996 (784 – 1183)	1329 (1056 – 1617)	0·07 (0·06 – 0·09)	25 (20 – 28)	64 (47 – 81)
Bermuda	‥	5·96 (5·06 – 6·97)	88 (49 – 124)	‥	0·13 (0·11 – 0·15)	2 (1 – 3)	0 (0 – 0)
Cuba	44 (35 – 54)	4·29 (3·65 – 4·91)	27 201 (21 141 – 33 629)	1342 (1039 – 1730)	0·11 (0·09 – 0·13)	527 (327 – 704)	1920 (1269 – 2695)
Dominica	23 (19 – 28)	7·58 (6·41 – 8·85)	54 (22 – 86)	1365 (1088 – 1676)	0·1 (0·09 – 0·12)	4 (3 – 5)	6 (4 – 9)
Dominican Republic	40 (33 – 48)	4·5 (3·84 – 5·26)	24 490 (18 527 – 29 940)	1185 (934 – 1442)	0·12 (0·1 – 0·14)	388 (255 – 494)	1454 (982 – 1967)
Grenada	45 (37 – 55)	4·55 (3·88 – 5·35)	248 (185 – 303)	1646 (1316 – 1998)	0·1 (0·08 – 0·12)	6 (4 – 7)	21 (15 – 28)
Guyana	11 (9 – 14)	6·09 (5·08 – 7·13)	1012 (517 – 1479)	1007 (779 – 1274)	0·06 (0·05 – 0·07)	62 (55 – 68)	74 (52 – 99)
Haiti	5 (4 – 6)	2·95 (2·46 – 3·44)	40 783 (35 773 – 45 780)	205 (164 – 249)	0·07 (0·06 – 0·08)	882 (747 – 981)	390 (299 – 485)
Jamaica	38 (31 – 46)	2·75 (2·34 – 3·18)	10 051 (9014 – 11 074)	2218 (1768 – 2717)	0·04 (0·03 – 0·05)	281 (261 – 300)	1014 (821 – 1223)
Puerto Rico	‥	8·71 (7·46 – 10·12)	1528 (-178 – 2836)	‥	0·12 (0·1 – 0·14)	153 (96 – 202)	0 (0 – 0)
Saint Lucia	36 (30 – 44)	5·98 (5·08 – 6·96)	265 (153 – 368)	1763 (1396 – 2166)	0·1 (0·08 – 0·12)	9 (7 – 12)	27 (18 – 36)
Saint Vincent and the Grenadines	30 (25 – 36)	4·24 (3·62 – 4·96)	275 (212 – 329)	1096 (886 – 1345)	0·09 (0·08 – 0·11)	6 (5 – 8)	15 (11 – 20)
Suriname	29 (24 – 35)	4·49 (3·81 – 5·21)	1226 (918 – 1518)	1080 (853 – 1343)	0·09 (0·08 – 0·11)	29 (20 – 35)	67 (46 – 89)
Trinidad and Tobago	121 (99 – 145)	3·73 (3·18 – 4·36)	3899 (3215 – 4531)	2295 (1810 – 2801)	0·15 (0·12 – 0·18)	27 (10 – 40)	537 (377 – 714)
Virgin Islands U·S·	‥	5·98 (5·03 – 7·05)	127 (60 – 184)	‥	0·14 (0·11 – 0·16)	4 (2 – 6)	0 (0 – 0)
Central Latin America	‥	5·94 (5·09 – 6·85)	380 121 (252 615 – 495 549)	‥	0·08 (0·07 – 0·09)	16 581 (14 173 – 18 455)	50 882 (39 397 – 62 178)
Colombia	17 (14 – 21)	8·86 (7·54 – 10·35)	6139 (-14333 – 25 382)	2423 (2089 – 2742)	0·05 (0·05 – 0·05)	4122 (3892 – 4313)	10 120 (8387 – 11 632)
Costa Rica	92 (75 – 112)	4·38 (3·71 – 5·15)	11 198 (8563 – 13 699)	4260 (3380 – 5187)	0·07 (0·06 – 0·09)	316 (258 – 359)	2400 (1755 – 3076)
El Salvador	43 (37 – 48)	2·61 (2·38 – 2·82)	24 501 (23 066 – 25 961)	3353 (2824 – 3971)	0·03 (0·02 – 0·03)	672 (646 – 698)	3299 (2830 – 3794)
Guatemala	17 (13 – 20)	5·98 (4·97 – 7·24)	18 928 (2626 – 31 078)	1159 (923 – 1410)	0·07 (0·06 – 0·08)	1141 (944 – 1303)	1658 (1132 – 2233)
Honduras	12 (10 – 15)	5·59 (4·63 – 6·59)	13 067 (6947 – 18 450)	805 (622 – 1012)	0·07 (0·06 – 0·09)	567 (456 – 657)	625 (411 – 841)
Mexico	43 (36 – 51)	4·99 (4·29 – 5·77)	236 409 (160 140 – 305 056)	2059 (1618 – 2512)	0·08 (0·07 – 0·1)	7645 (5842 – 8954)	26 228 (18 374 – 34 244)
Nicaragua	7 (6 – 9)	8·49 (7·13 – 9·97)	612 (-2376 – 2991)	695 (551 – 854)	0·07 (0·06 – 0·09)	410 (336 – 468)	292 (189 – 396)
Panama	32 (26 – 38)	13·79 (11·76 – 15·98)	0 (0 – 0)	2418 (1960 – 2968)	0·14 (0·12 – 0·16)	72 (18 – 112)	178 (43 – 329)
Venezuela	46 (37 – 57)	4·8 (4·01 – 5·65)	69 267 (52 395 – 84 376)	1725 (1374 – 2122)	0·1 (0·08 – 0·12)	1637 (1408 – 1841)	6083 (4487 – 7889)
Tropical Latin America	‥	7·27 (6·79 – 7·79)	109 498 (63 846 – 156 000)	‥	0·12 (0·11 – 0·14)	6556 (4321 – 8378)	20 558 (13 107 – 27 845)
Brazil	51 (45 – 56)	7·33 (6·85 – 7·85)	98 890 (56 314 – 140 448)	2367 (1985 – 2777)	0·12 (0·11 – 0·14)	6167 (3989 – 7942)	19 796 (12 529 – 26 906)
Paraguay	24 (20 – 30)	5·51 (4·68 – 6·51)	10 607 (5839 – 14 711)	1271 (1010 – 1559)	0·09 (0·07 – 0·1)	389 (304 – 455)	762 (519 – 1013)
North Africa and Middle East	‥	7·13 (6·31 – 8·23)	676 422 (469 769 – 838 873)	‥	0·12 (0·11 – 0·14)	23 918 (17 677 – 28 552)	40 399 (26 699 – 53 437)

Afghanistan	2 (1 – 2)	5·61 (4·67 – 6·9)	63 000 (28 873 – 87 927)	172 (130 – 219)	0·08 (0·07 – 0·1)	2490 (1879 – 2993)	551 (350 – 765)
Algeria	19 (16 – 23)	5·61 (4·74 – 6·66)	47 746 (26 082 – 66 884)	1034 (781 – 1306)	0·12 (0·1 – 0·16)	1108 (502 – 1609)	2121 (1100 – 3193)
Bahrain	52 (40 – 64)	10·69 (8·81 – 13·26)	9 (-11 – 27)	10 273 (7851 – 12 821)	0·06 (0·05 – 0·07)	87 (72 – 98)	905 (567 – 1223)
Egypt	3 (2 – 4)	9·29 (7·89 – 11·49)	1275 (-4466 – 6068)	186 (146 – 228)	0·18 (0·15 – 0·21)	1492 (553 – 2250)	288 (91 – 488)
Iran	17 (14 – 21)	7·23 (6·21 – 8·57)	21 686 (-20514 – 53 980)	1873 (1445 – 2348)	0·08 (0·07 – 0·1)	4995 (3755 – 5906)	9876 (5825 – 14 039)
Iraq	24 (19 – 31)	2·33 (1·94 – 2·77)	159 645 (142 270 – 174 339)	782 (576 – 1036)	0·09 (0·07 – 0·11)	2264 (1420 – 2905)	5719 (4092 – 7440)
Jordan	24 (19 – 30)	4·73 (3·97 – 5·69)	15 825 (9757 – 20 581)	552 (427 – 698)	0·25 (0·2 – 0·3)	28 (11 – 43)	403 (206 – 611)
Kuwait	65 (51 – 80)	8·64 (7·24 – 10·59)	217 (-427 – 747)	4681 (3469 – 6108)	0·13 (0·1 – 0·16)	69 (22 – 108)	353 (89 – 658)
Lebanon	49 (38 – 60)	5·16 (4·34 – 6·15)	12 180 (7591 – 15 958)	2934 (2306 – 3618)	0·1 (0·08 – 0·12)	315 (228 – 388)	1539 (1023 – 2061)
Libya	14 (11 – 18)	7·93 (6·81 – 9·44)	1932 (-1693 – 4475)	999 (738 – 1308)	0·13 (0·11 – 0·16)	158 (64 – 240)	194 (63 – 341)
Morocco	12 (10 – 15)	4·78 (3·97 – 5·83)	77 838 (52 509 – 97 910)	1344 (1044 – 1683)	0·05 (0·04 – 0·07)	2867 (2547 – 3136)	4854 (3602 – 6239)
Oman	34 (26 – 42)	9·14 (7·59 – 11·27)	19 (-75 – 104)	5974 (4508 – 7561)	0·06 (0·05 – 0·07)	300 (250 – 339)	1806 (1136 – 2537)
Palestine	8 (7 – 10)	5·21 (4·37 – 6·19)	4803 (896 – 8395)	662 (492 – 885)	0·08 (0·07 – 0·11)	278 (170 – 361)	230 (113 – 359)
Qatar	64 (48 – 81)	14·61 (11·97 – 17·89)	0 (0 – 0)	7533 (5354 – 10 209)	0·13 (0·1 – 0·17)	29 (5 – 47)	232 (38 – 449)
Saudi Arabia	54 (44 – 65)	10·55 (9·09 – 12·28)	637 (-1938 – 3394)	5260 (4124 – 6563)	0·12 (0·1 – 0·14)	802 (437 – 1111)	4345 (1948 – 6963)
Sudan	7 (6 – 9)	5·5 (4·62 – 6·7)	73 204 (34 725 – 101 941)	580 (429 – 762)	0·09 (0·07 – 0·11)	2331 (1590 – 2886)	1918 (1135 – 2772)
Syria	1 (1 – 1)	5·06 (4·27 – 6·03)	61 729 (46 076 – 74 829)	38 (29 – 48)	0·13 (0·1 – 0·15)	846 (486 – 1160)	79 (50 – 110)
Tunisia	12 (9 – 14)	7·28 (6·22 – 8·71)	3098 (-1641 – 7151)	985 (773 – 1223)	0·1 (0·09 – 0·12)	516 (360 – 649)	556 (327 – 828)
Turkey	13 (11 – 15)	12·18 (11·65 – 12·72)	0 (0 – 0)	819 (704 – 940)	0·22 (0·21 – 0·23)	358 (213 – 499)	295 (166 – 428)
United Arab Emirates	78 (59 – 99)	8·49 (6·81 – 10·95)	402 (-827 – 1492)	7529 (5702 – 9516)	0·09 (0·08 – 0·12)	272 (124 – 384)	2145 (773 – 3653)
Yemen	8 (7 – 11)	2·5 (2·22 – 2·81)	131 176 (121 549 – 140 001)	382 (272 – 518)	0·07 (0·06 – 0·09)	2312 (1786 – 2718)	1992 (1504 – 2638)
South Asia	‥	4·67 (3·93 – 5·48)	4 027 525 (3 113 082 – 4 896 398)	‥	0·07 (0·06 – 0·08)	120 653 (108 685 – 130 918)	38 906 (30 448 – 47 561)
Bangladesh	2 (1 – 2)	4·39 (3·69 – 5·18)	422 808 (323 953 – 514 241)	119 (93 – 148)	0·07 (0·06 – 0·08)	12 253 (10 232 – 13 694)	2218 (1610 – 2822)
Bhutan	4 (4 – 5)	5·91 (5·0 – 6·92)	1100 (605 – 1507)	265 (208 – 336)	0·1 (0·08 – 0·12)	36 (24 – 45)	15 (9 – 20)
India	4 (3 – 5)	4·59 (3·82 – 5·4)	3 217 963 (2 493 450 – 3 913 540)	230 (192 – 275)	0·07 (0·07 – 0·08)	92 448 (84 033 – 100 097)	33 460 (26 322 – 41 004)
Nepal	4 (3 – 5)	2·65 (2·24 – 3·07)	113 559 (102 332 – 124 285)	152 (120 – 192)	0·07 (0·06 – 0·08)	2207 (1843 – 2507)	769 (601 – 958)
Pakistan	2 (1 – 2)	5·76 (4·89 – 6·74)	272 095 (150 396 – 379 250)	144 (109 – 182)	0·07 (0·06 – 0·08)	13 709 (11 176 – 15 491)	2444 (1690 – 3248)
Southeast Asia East Asia and Oceania	‥	6·69 (6·11 – 7·29)	1 709 453 (1 060 336 – 2 339 121)	‥	0·14 (0·13 – 0·15)	71 154 (62 987 – 78 765)	130 584 (102 424 – 160 421)
East Asia	‥	7·1 (6·6 – 7·59)	753 139 (400 129 – 1 114 796)	‥	0·18 (0·17 – 0·2)	12 175 (6354 – 17 677)	33 462 (20 311 – 47 564)
China	30 (28 – 33)	6·73 (6·28 – 7·17)	751 600 (401 537 – 1 113 190)	822 (739 – 914)	0·18 (0·17 – 0·2)	11 360 (5698 – 16 459)	32 656 (19 837 – 46 420)
North Korea	5 (4 – 6)	7·98 (6·79 – 9·38)	1539 (-7288 – 7371)	246 (199 – 299)	0·14 (0·12 – 0·16)	579 (159 – 927)	156 (32 – 286)
Taiwan	20 (17 – 25)	31·05 (26·72 – 36·07)	0 (0 – 0)	2642 (2078 – 3277)	0·17 (0·14 – 0·2)	236 (-13 – 443)	649 (-8 – 1382)
Oceania	‥	6·17 (5·19 – 7·76)	13 061 (2801 – 19 123)	‥	0·07 (0·06 – 0·09)	752 (620 – 860)	411 (279 – 534)
American Samoa	‥	9·87 (8·28 – 11·83)	2 (-2 – 6)	‥	0·09 (0·08 – 0·11)	4 (3 – 5)	0 (0 – 0)
Federated States of Micronesia	14 (12 – 17)	8·2 (6·88 – 9·65)	40 (-18 – 102)	1042 (837 – 1253)	0·09 (0·07 – 0·11)	6 (5 – 7)	7 (4 – 10)
Fiji	10 (8 – 13)	8·78 (7·32 – 10·99)	233 (-83 – 516)	835 (671 – 996)	0·08 (0·07 – 0·1)	58 (47 – 67)	52 (33 – 70)
Guam	‥	10·14 (8·73 – 11·96)	3 (-8 – 12)	‥	0·1 (0·08 – 0·12)	8 (6 – 11)	0 (0 – 0)
Kiribati	9 (7 – 12)	6·55 (5·43 – 7·82)	88 (11 – 160)	697 (544 – 871)	0·07 (0·06 – 0·09)	8 (6 – 9)	6 (4 – 9)
Marshall Islands	33 (26 – 40)	7·12 (5·89 – 8·91)	41 (-9 – 81)	2449 (1980 – 2948)	0·07 (0·06 – 0·09)	5 (4 – 6)	13 (9 – 18)
Northern Mariana Islands	‥	9·87 (8·18 – 12·47)	0 (-1 – 1)	‥	0·09 (0·07 – 0·11)	6 (4 – 7)	0 (0 – 0)
Papua New Guinea	5 (4 – 7)	5·68 (4·77 – 7·26)	11 686 (2535 – 16 966)	346 (282 – 421)	0·07 (0·06 – 0·09)	576 (474 – 664)	267 (177 – 357)

Samoa	14 (11 – 17)	7·61 (6·31 – 9·23)	50 (-36 – 134)	998 (799 – 1205)	0·08 (0·07 – 0·1)	12 (9 – 14)	13 (8 – 18)
Solomon Islands	8 (7 – 10)	6·18 (5·07 – 7·34)	652 (261 – 1022)	629 (496 – 769)	0·07 (0·06 – 0·08)	45 (38 – 51)	34 (23 – 45)
Tonga	10 (8 – 13)	8·09 (6·86 – 9·81)	22 (-32 – 63)	748 (614 – 891)	0·09 (0·07 – 0·1)	6 (5 – 7)	5 (3 – 7)
Vanuatu	10 (7 – 12)	6·37 (5·33 – 7·83)	244 (-5 – 403)	642 (497 – 809)	0·08 (0·06 – 0·09)	18 (14 – 21)	14 (9 – 20)
Southeast Asia	‥	5·73 (4·82 – 6·78)	943 254 (591 879 – 1 254 765)	‥	0·04 (0·03 – 0·05)	58 227 (54 094 – 61 475)	96 711 (75 355 – 119 452)
Cambodia	6 (5 – 7)	5·1 (4·55 – 5·76)	27 443 (19 330 – 34 554)	990 (788 – 1208)	0·03 (0·02 – 0·03)	1640 (1554 – 1723)	1802 (1418 – 2257)
Indonesia	12 (9 – 14)	5·83 (4·7 – 7·24)	295 299 (93 244 – 446 300)	1213 (921 – 1533)	0·04 (0·03 – 0·05)	20 694 (18 605 – 22 305)	28 794 (20 757 – 38 324)
Laos	6 (5 – 7)	4·48 (3·74 – 5·3)	12 330 (7785 – 16 551)	829 (660 – 1023)	0·02 (0·02 – 0·03)	728 (689 – 768)	677 (530 – 850)
Malaysia	18 (15 – 21)	12·69 (10·7 – 14·84)	0 (0 – 0)	3757 (3048 – 4552)	0·04 (0·04 – 0·05)	2447 (2230 – 2638)	9236 (6904 – 11 901)
Maldives	109 (85 – 135)	6·01 (5·04 – 7·11)	459 (192 – 687)	12 829 (9972 – 15 884)	0·04 (0·03 – 0·04)	33 (31 – 35)	474 (349 – 609)
Mauritius	39 (32 – 47)	6·78 (5·73 – 7·96)	851 (-14 – 1571)	4529 (3651 – 5557)	0·04 (0·03 – 0·05)	121 (111 – 129)	582 (434 – 752)
Myanmar	5 (4 – 6)	5·73 (4·82 – 6·77)	69 602 (33 044 – 101 798)	821 (653 – 1028)	0·03 (0·02 – 0·03)	5260 (4990 – 5521)	4699 (3602 – 6028)
Philippines	9 (7 – 10)	6·94 (5·83 – 8·1)	61 728 (-3412 – 114 239)	1697 (1371 – 2060)	0·03 (0·02 – 0·03)	10 025 (9471 – 10 537)	17 573 (13 537 – 22 100)
Sri Lanka	9 (7 – 10)	6·38 (5·41 – 7·46)	19 598 (7286 – 30 838)	1004 (810 – 1221)	0·04 (0·03 – 0·05)	1979 (1825 – 2115)	2166 (1633 – 2746)
Seychelles	53 (43 – 64)	5·22 (4·5 – 5·95)	141 (84 – 196)	4837 (3727 – 5966)	0·04 (0·03 – 0·05)	9 (8 – 10)	51 (38 – 65)
Thailand	30 (25 – 36)	3·7 (3·13 – 4·29)	194 354 (161 103 – 226 590)	1850 (1463 – 2264)	0·04 (0·04 – 0·05)	6255 (5714 – 6751)	17 576 (13 678 – 21 881)
Timor-Leste	4 (3 – 5)	6·53 (5·49 – 7·85)	946 (44 – 1620)	590 (458 – 746)	0·03 (0·03 – 0·04)	111 (103 – 118)	69 (52 – 91)
Vietnam	14 (12 – 17)	3·77 (3·17 – 4·38)	260 504 (214 653 – 306 356)	1032 (834 – 1254)	0·04 (0·03 – 0·05)	8926 (8260 – 9545)	13 011 (10 316 – 15 939)
Sub-Saharan Africa	‥	3·77 (3·22 – 4·42)	3 020 640 (2 414 437 – 3 539 543)	‥	0·06 (0·05 – 0·07)	87 442 (76 512 – 95 734)	44 436 (34 158 – 54 100)
Central sub-Saharan Africa	‥	3·5 (2·92 – 4·23)	393 978 (301 686 – 465 275)	‥	0·07 (0·06 – 0·09)	9590 (7859 – 11 045)	2542 (1802 – 3288)
Angola	9 (7 – 11)	4·07 (3·36 – 4·76)	63 953 (46 328 – 81 532)	369 (286 – 467)	0·08 (0·06 – 0·09)	1717 (1302 – 2081)	1232 (843 – 1675)
Central African Republic	1 (1 – 1)	2·87 (2·36 – 3·66)	25 402 (20 393 – 28 715)	47 (37 – 57)	0·06 (0·05 – 0·07)	589 (507 – 653)	56 (42 – 70)
Congo (Brazzaville)	4 (3 – 5)	4·88 (4·12 – 5·96)	10 362 (6201 – 13 219)	180 (142 – 222)	0·09 (0·08 – 0·11)	282 (203 – 349)	97 (63 – 129)
Democratic Republic of the Congo	2 (1 – 2)	3·26 (2·68 – 4·0)	290 433 (227 403 – 340 120)	69 (54 – 85)	0·07 (0·06 – 0·08)	6881 (5634 – 7924)	959 (696 – 1220)
Equatorial Guinea	32 (25 – 41)	5·67 (4·75 – 6·85)	1471 (691 – 2070)	1367 (1030 – 1733)	0·1 (0·08 – 0·11)	47 (34 – 59)	114 (70 – 162)
Gabon	16 (12 – 19)	5·75 (4·79 – 7·02)	2358 (798 – 3489)	614 (492 – 751)	0·11 (0·09 – 0·13)	75 (43 – 100)	85 (46 – 124)
Eastern sub-Saharan Africa	‥	4·28 (3·67 – 5·01)	996 828 (743 228 – 1 207 013)	‥	0·05 (0·05 – 0·06)	36 696 (33 170 – 39 410)	11 333 (8868 – 13 671)
Burundi	1 (1 – 1)	5·98 (5·02 – 7·3)	11 871 (3913 – 18 542)	94 (75 – 116)	0·05 (0·04 – 0·06)	1158 (1015 – 1274)	121 (86 – 157)
Comoros	4 (3 – 5)	5·91 (4·89 – 7·38)	955 (243 – 1488)	130 (101 – 161)	0·15 (0·12 – 0·18)	15 (4 – 23)	6 (2 – 9)
Djibouti	6 (4 – 7)	4·89 (4·06 – 6·02)	1304 (408 – 1940)	343 (273 – 414)	0·06 (0·05 – 0·08)	70 (57 – 79)	31 (21 – 42)
Eritrea	1 (1 – 2)	5·08 (4·22 – 6·0)	9630 (5819 – 13 329)	98 (76 – 125)	0·06 (0·05 – 0·08)	408 (344 – 462)	55 (37 – 73)
Ethiopia	2 (2 – 3)	3·94 (3·28 – 4·61)	273 222 (206 661 – 334 516)	246 (194 – 311)	0·03 (0·03 – 0·04)	10 888 (10 182 – 11 519)	3339 (2525 – 4242)
Kenya	6 (5 – 7)	4·98 (4·24 – 5·94)	98 404 (62 012 – 127 388)	467 (378 – 556)	0·05 (0·04 – 0·06)	4086 (3634 – 4445)	2520 (1873 – 3132)
Madagascar	1 (1 – 2)	4·19 (3·44 – 5·3)	60 315 (32 355 – 79 371)	103 (82 – 130)	0·05 (0·04 – 0·06)	2491 (2225 – 2719)	343 (246 – 447)
Malawi	3 (2 – 3)	2·91 (2·39 – 3·48)	80 086 (68 235 – 90 326)	145 (118 – 171)	0·05 (0·04 – 0·06)	2061 (1859 – 2214)	523 (414 – 631)
Mozambique	2 (1 – 2)	3·53 (2·93 – 4·27)	111 554 (86 778 – 131 250)	118 (94 – 144)	0·04 (0·04 – 0·05)	3467 (3119 – 3768)	608 (465 – 751)
Rwanda	3 (2 – 3)	6·41 (5·86 – 7·0)	9032 (3988 – 13 555)	494 (395 – 613)	0·03 (0·02 – 0·04)	1267 (1188 – 1341)	653 (495 – 839)
Somalia	1 (1 – 2)	4·21 (3·49 – 5·13)	31 363 (22 963 – 38 164)	105 (81 – 129)	0·05 (0·04 – 0·07)	1007 (886 – 1104)	153 (114 – 194)
South Sudan	1 (1 – 1)	2·81 (2·34 – 3·47)	53 687 (42 287 – 62 028)	37 (29 – 45)	0·04 (0·04 – 0·06)	1594 (1410 – 1743)	98 (74 – 121)
Tanzania	3 (2 – 3)	5·46 (4·54 – 6·56)	82 667 (28 247 – 127 002)	125 (99 – 155)	0·1 (0·08 – 0·12)	2717 (1706 – 3547)	587 (308 – 849)
Uganda	3 (3 – 4)	4·17 (3·85 – 4·51)	104 655 (89 660 – 119 680)	232 (196 – 270)	0·05 (0·04 – 0·06)	4041 (3717 – 4317)	1282 (1057 – 1518)

Zambia	8 (7 – 10)	2·97 (2·42 – 3·68)	68 083 (55 857 – 77 098)	299 (238 – 364)	0·07 (0·06 – 0·08)	1426 (1181 – 1625)	1013 (756 – 1278)
Southern sub-Saharan Africa	..	3·32 (2·82 – 3·9)	315 824 (272 681 – 351 952)	..	0·13 (0·1 – 0·16)	3230 (1217 – 4639)	14 780 (10 718 – 19 024)
Botswana	45 (36 – 55)	3·92 (3·27 – 4·69)	6989 (5418 – 8325)	1552 (1203 – 1925)	0·09 (0·07 – 0·1)	144 (109 – 174)	545 (381 – 712)
Lesotho	10 (8 – 12)	2·42 (2·0 – 2·92)	14 410 (13 144 – 15 473)	348 (280 – 427)	0·06 (0·05 – 0·07)	287 (255 – 315)	244 (194 – 297)
Namibia	51 (41 – 61)	3·57 (3·01 – 4·26)	8217 (6723 – 9470)	1331 (1061 – 1638)	0·1 (0·09 – 0·13)	120 (79 – 155)	584 (406 – 764)
South Africa	50 (41 – 59)	3·53 (2·99 – 4·17)	212 016 (181 378 – 239 044)	871 (634 – 1148)	0·15 (0·12 – 0·2)	1078 (-660 – 2294)	11 610 (8105 – 15 309)
Swaziland (eSwatini)	29 (23 – 35)	2·93 (2·43 – 3·53)	6271 (5439 – 6994)	1840 (1441 – 2325)	0·04 (0·03 – 0·04)	171 (159 – 181)	496 (390 – 619)
Zimbabwe	11 (9 – 13)	2·73 (2·28 – 3·31)	67 921 (59 244 – 75 421)	396 (314 – 489)	0·06 (0·05 – 0·08)	1431 (1214 – 1613)	1301 (987 – 1598)
Western sub-Saharan Africa	..	3·49 (2·96 – 4·14)	1 314 010 (1 052 581 – 1 531 034)	..	0·06 (0·05 – 0·07)	37 925 (33 376 – 41 367)	15 781 (12 131 – 19 252)
Benin	2 (2 – 3)	3·71 (3·13 – 4·39)	32 265 (24 674 – 38 953)	145 (116 – 174)	0·05 (0·04 – 0·06)	1073 (949 – 1179)	234 (176 – 290)
Burkina Faso	5 (4 – 6)	1·6 (1·32 – 1·89)	107 078 (99 758 – 113 840)	141 (113 – 173)	0·05 (0·04 – 0·06)	2156 (1938 – 2353)	831 (680 – 993)
Cameroon	4 (3 – 5)	4·07 (3·32 – 5·14)	67 795 (42 948 – 85 051)	232 (186 – 280)	0·06 (0·05 – 0·07)	2145 (1835 – 2406)	800 (573 – 1027)
Cape Verde	11 (9 – 13)	6·16 (5·2 – 7·44)	465 (23 – 813)	624 (499 – 751)	0·09 (0·07 – 0·1)	32 (23 – 38)	25 (15 – 35)
Chad	3 (2 – 4)	2·96 (2·47 – 3·79)	58 393 (44 688 – 67 045)	186 (145 – 232)	0·04 (0·03 – 0·05)	1769 (1601 – 1900)	506 (381 – 631)
Cote d'Ivoire	6 (5 – 7)	3·92 (3·26 – 4·72)	65 217 (46 680 – 80 859)	283 (230 – 348)	0·06 (0·05 – 0·08)	1959 (1663 – 2225)	929 (671 – 1197)
The Gambia	2 (2 – 2)	5·31 (4·39 – 6·33)	2790 (1133 – 4250)	146 (116 – 177)	0·06 (0·05 – 0·07)	163 (135 – 184)	30 (20 – 39)
Ghana	6 (5 – 6)	4·79 (4·38 – 5·18)	53 317 (44 430 – 62 866)	515 (415 – 618)	0·04 (0·04 – 0·05)	2674 (2465 – 2855)	1686 (1319 – 2056)
Guinea	2 (1 – 2)	3·45 (2·88 – 4·14)	43 783 (34 234 – 52 028)	100 (80 – 122)	0·05 (0·04 – 0·06)	1319 (1174 – 1442)	208 (158 – 256)
Guinea-Bissau	3 (3 – 4)	3·65 (3·05 – 4·35)	5952 (4596 – 7065)	184 (146 – 228)	0·06 (0·05 – 0·07)	176 (153 – 196)	53 (40 – 67)
Liberia	2 (1 – 2)	3·58 (2·99 – 4·36)	15 502 (11 817 – 18 396)	102 (82 – 125)	0·06 (0·05 – 0·07)	446 (388 – 493)	74 (55 – 93)
Mali	4 (4 – 5)	2·08 (1·84 – 2·33)	96 252 (89 929 – 102 622)	245 (214 – 278)	0·03 (0·03 – 0·03)	2571 (2465 – 2671)	1040 (909 – 1171)
Mauritania	4 (3 – 4)	5·25 (4·36 – 6·39)	6893 (3621 – 9457)	186 (147 – 227)	0·08 (0·07 – 0·1)	231 (172 – 279)	70 (47 – 93)
Niger	2 (2 – 3)	2·41 (1·97 – 3·0)	97 816 (83 636 – 109 861)	100 (78 – 122)	0·04 (0·04 – 0·05)	2520 (2253 – 2718)	459 (355 – 563)
Nigeria	6 (5 – 8)	3·54 (2·93 – 4·3)	595 624 (450 474 – 717 983)	269 (210 – 331)	0·06 (0·05 – 0·08)	15 940 (13 191 – 18 072)	8012 (5775 – 10 334)
Sao Tome and Principe	9 (7 – 11)	4·94 (4·12 – 6·03)	315 (156 – 439)	419 (332 – 515)	0·09 (0·07 – 0·11)	11 (7 – 13)	7 (4 – 10)
Senegal	3 (3 – 4)	4·16 (3·51 – 4·94)	36 370 (25 259 – 45 456)	247 (197 – 301)	0·05 (0·04 – 0·06)	1383 (1233 – 1510)	472 (361 – 595)
Sierra Leone	2 (2 – 3)	6·14 (5·1 – 7·57)	7602 (2229 – 11 947)	245 (191 – 301)	0·05 (0·04 – 0·06)	722 (648 – 786)	195 (141 – 252)
Togo	3 (2 – 3)	4·08 (3·43 – 4·93)	20 583 (14 857 – 25 208)	150 (121 – 184)	0·06 (0·05 – 0·07)	637 (539 – 714)	151 (110 – 193)

7.5 Sensitivity analysis – intermediate UHC standard for utilisation

We conducted a sensitivity analyses with an intermediate UHC standard for utilisation. For the main analysis, the additional admissions required was relatively larger than visits. As noted above, Portugal served as an intermediate UHC standard for utilisation with a smaller ratio of admissions to counterfactual DALYs and lower additional cost of inpatient admissions.

$$Cost_{ijkl} = \left(\left(\frac{volume_{Portugaljkl}}{DALY0s_{Portugalkl}}\right) - \left(\frac{volume_{ijkl}}{DALY0s_{ikl}}\right) \right) * DALY0s_{ikl} * unitcost_{ij} \quad (10)$$

where *DALY*0 denotes the counterfactual DALYs with health access and quality removed, *i* refers country, *j* to the service (outpatient or inpatient), *k* to age category, and *l* to sex. As reported in Table S20, 0.23 billion additional admissions were needed, a 33% increase from current volume rather than a 49% increase; 7.67 billion additional visits were needed, a 19% increase from current volume rather than a 26% increase.

7.6 Sensitivity analysis – improvements in quality and types of service

Health systems differed in the quality and type of services they provide, as well as in the volume of services, and the main analysis did not account for these differences. Expenditures for improved quality and types of service would be reported in several NHA health care function categories such as curative care (HC 1), and rehabilitative care (HC 2), which were the topic of our analysis, as well as ancillary services (HC 4), medical goods (HC 5), governance and health system and financing administration (HC 7), and NHA capital account categories such as fixed capital formation (HK 1), and education and training of health personal (HKR.5).

For the recurrent costs of outpatient visits reported in NHA categories HC 1.3 and HC 2.3, improved quality and type of service would be in reflected in longer visits, additional exams and medical goods during the visit, and some aspects of higher salaries. To the extent earnings are foregone during health professional education, which were not counted in category HKR.5, professionals would have higher salaries as a return on their investment. For the recurrent costs of inpatient admissions reported in NHA categories HC 1.1 and HC 1.2, improved quality and type of service would be reflected in more frequent rounds by the medical staff, additional exams and medical goods during the admission, infection control, hygiene, and nutrition, as well as some aspects of higher salaries.

Personnel was a large share of the cost of personal health services. For our sensitivity analysis, we assumed that the cost of personnel would increase as a fixed share of gross domestic product (GDP) per capita for a given level of quality and type of service. To estimate the fixed share, we regressed unit costs on GDP per capita, and the R^2 for the regression was 0.50 for outpatient services and 0.55 for inpatient. Systematic differences above the fixed share would represent an increase in the quality and type of service.

To test the hypothesis that differences in unit cost across countries may be explained by differences in quality and type of service, we regressed unit costs on GDP per capita with splines at each GDP per capita quintile. The R^2 for the regression was 0.60 for outpatient services and 0.63 for inpatient. At the Netherlands' GPD per capita, the ratio of the spline result and the fixed share, was 1.28 for outpatient unit costs and 1.24 for inpatient unit cost. In our sensitivity analysis reported in Table S21, we increased outpatient unit costs by 28% and inpatient unit costs by 24%.

A caveat to this sensitivity analysis is that the unit cost of services could differ across countries for other reasons, such as inefficiency and population density where remote facilities have fewer patients. Our analysis assumes that systematic differences represent only differences in quality and type of service.

Table S20: Sensitivity analysis of national unit costs and funds needed to achieve an intermediate Universal Health Coverage standard for utilisation in 2016 in 2017 international dollars

Table displays four sets of national estimates organised by GBD region: 1) cost per outpatient visit and inpatient admission by country, 2) aggregate ratio of total outpatient visits and inpatient admissions to counterfactual DALYs, where the counterfactual DALYs standardised the burden of disease across countries by removing the effects of access and quality of health care, 3) estimates of additional services needed to achieve an intermediate UHC standard for utilisation calculated by age and sex category, and 4) total cost of additional services in 2017 international dollars and as a percentage of 2016 gross domestic product. The cost of scaling up to the UHC utilisation standard was done at the age-sex level, leading some countries to have a cost of UHC scale-up greater than zero despite their aggregate utilisation per counterfactual DALY being greater than the standard set by the Portugal. For each result, the mean of 1,000 draws is reported, and in parenthesis the uncertainty interval defined as the 2.5th and 97.5th percentile of draws. DALY = disability-adjusted-life-year. GBD= Global Burden of Disease, UHC = Universal Health Coverage.

Table S20: Sensitivity analysis of national unit costs and funds needed to achieve an intermediate Universal Health Coverage standard for utilisation in 2016 in 2017 international dollars

	Outpatient visits			Inpatient admissions			Total additional cost to meet UHC standards in millions 2017 PPP $ (95% UI)
	Unit cost per outpatient visit in 2017 PPP $ (95% UI) (Table S14)	Ratio of total outpatient visits to counterfactual DALYs (95% UI) (Table 1)	Additional outpatient visits to meet UHC standard (95% UI) (in thousands)	Unit cost per inpatient admission in 2017 PPP $ (95% UI) (Table S14)	Ratio of total inpatient admissions to counterfactual DALYs (95% UI) (Table 1)	Additional inpatient admissions to meet UHC standard (95% UI) (in thousands)	
Global	‥	6·7 (6·04 – 7·41)	7 671 882 (5 332 598 – 9 750 543)	‥	0·12 (0·11 – 0·13)	233 699 (202 092 – 260 652)	745 588 (556 421 – 932 086)
Low income	‥	‥	1 344 863 (996 539 – 1 637 357)	‥	‥	45 557 (39 521 – 50 117)	34 717 (26 850 – 42 024)
Lower middle income	‥	‥	4 637 681 (3 109 297 – 6 040 344)	‥	‥	151 005 (133 092 – 166 621)	336 021 (260 758 – 414 015)
Upper middle income	‥	‥	1 575 140 (1 111 949 – 2 005 508)	‥	‥	33 281 (25 194 – 40 082)	278 137 (206 399 – 343 585)
High income	‥	‥	114 197 (19 582 – 197 213)	‥	‥	3856 (1315 – 6041)	96 714 (44 234 – 147 957)
Central Europe, Eastern Europe, and Central Asia	‥	10·82 (10·37 – 11·31)	100 242 (69 304 – 130 366)	‥	0·24 (0·23 – 0·25)	1905 (889 – 3025)	8117 (5807 – 10 548)
Central Asia	‥	8·25 (7·95 – 8·54)	40 050 (34 726 – 45 306)	‥	0·17 (0·17 – 0·18)	1147 (986 – 1295)	4473 (3905 – 5042)
Armenia	48 (40 – 57)	4·86 (4·67 – 5·05)	4740 (4154 – 5342)	1710 (1426 – 2019)	0·16 (0·16 – 0·17)	38 (28 – 47)	295 (233 – 363)
Azerbaijan	62 (54 – 70)	5·8 (5·53 – 6·08)	7950 (6417 – 9565)	4938 (4342 – 5613)	0·08 (0·08 – 0·09)	375 (339 – 411)	2346 (1965 – 2762)
Georgia	31 (26 – 38)	7·13 (6·13 – 8·43)	1563 (70 – 3233)	2408 (2128 – 2720)	0·11 (0·1 – 0·11)	122 (104 – 140)	345 (260 – 444)
Kazakhstan	39 (35 – 43)	8·27 (7·91 – 8·65)	2192 (1056 – 3514)	1872 (1699 – 2046)	0·19 (0·18 – 0·2)	132 (86 – 178)	333 (220 – 463)
Kyrgyzstan	18 (16 – 20)	4·06 (3·91 – 4·22)	10 468 (9314 – 11 486)	525 (472 – 595)	0·17 (0·17 – 0·18)	57 (35 – 76)	216 (184 – 255)
Mongolia	22 (18 – 26)	6·39 (5·38 – 7·61)	1206 (388 – 2037)	907 (717 – 1126)	0·18 (0·15 – 0·22)	22 (9 – 33)	47 (23 – 74)
Tajikistan	9 (8 – 10)	5·69 (5·44 – 5·96)	4578 (3220 – 5907)	410 (368 – 459)	0·15 (0·15 – 0·16)	124 (98 – 148)	91 (71 – 112)
Turkmenistan	78 (68 – 89)	4·81 (4·6 – 5·02)	7068 (6054 – 8116)	2182 (1706 – 2668)	0·2 (0·16 – 0·24)	30 (9 – 44)	619 (497 – 758)
Uzbekistan	10 (9 – 11)	11·85 (11·37 – 12·32)	285 (-50 – 662)	717 (648 – 790)	0·21 (0·2 – 0·22)	248 (181 – 313)	181 (127 – 237)
Central Europe	‥	10·21 (9·83 – 10·6)	22 489 (16 758 – 27 964)	‥	0·25 (0·23 – 0·27)	221 (129 – 301)	1925 (1426 – 2428)
Albania	36 (30 – 44)	6·98 (5·92 – 8·13)	1201 (-257 – 2770)	2546 (2275 – 2879)	0·12 (0·11 – 0·12)	63 (47 – 79)	208 (128 – 294)
Bosnia and Herzegovina	25 (21 – 29)	12·28 (10·83 – 14·43)	0 (0 – 0)	2550 (2104 – 3087)	0·14 (0·12 – 0·16)	37 (-11 – 77)	99 (-25 – 227)
Bulgaria	68 (63 – 74)	7·17 (6·7 – 7·63)	2964 (1306 – 4559)	1592 (1460 – 1728)	0·35 (0·33 – 0·37)	0 (0 – 0)	203 (89 – 323)
Croatia	69 (63 – 76)	8·06 (7·74 – 8·42)	746 (263 – 1237)	2753 (2489 – 3067)	0·23 (0·21 – 0·24)	9 (-7 – 24)	76 (8 – 148)
Czech Republic	64 (59 – 69)	14·1 (13·48 – 14·84)	0 (0 – 0)	3035 (2546 – 3547)	0·32 (0·28 – 0·38)	0 (0 – 0)	0 (0 – 0)
Hungary	42 (38 – 45)	15·6 (15·03 – 16·24)	0 (0 – 0)	2764 (2528 – 3013)	0·26 (0·24 – 0·28)	0 (0 – 0)	0 (0 – 0)
Macedonia	33 (28 – 38)	8·32 (7·93 – 8·72)	434 (288 – 579)	2081 (1800 – 2521)	0·15 (0·14 – 0·16)	21 (9 – 31)	59 (30 – 86)
Montenegro	33 (30 – 36)	9·39 (8·79 – 9·95)	18 (-8 – 42)	2264 (2067 – 2469)	0·16 (0·15 – 0·17)	4 (1 – 6)	9 (3 – 14)
Poland	62 (57 – 67)	10·21 (9·78 – 10·63)	0 (0 – 0)	2940 (2679 – 3219)	0·24 (0·22 – 0·25)	0 (0 – 0)	0 (0 – 0)
Romania	58 (53 – 65)	6·11 (5·83 – 6·38)	16 799 (13 053 – 20 413)	1393 (1231 – 1568)	0·29 (0·27 – 0·32)	0 (0 – 0)	983 (730 – 1251)
Serbia	44 (40 – 48)	9·3 (8·93 – 9·65)	326 (-56 – 689)	3155 (2844 – 3495)	0·15 (0·14 – 0·16)	87 (57 – 115)	288 (182 – 404)
Slovakia	47 (43 – 52)	16·97 (16·28 – 17·65)	0 (0 – 0)	3164 (2865 – 3499)	0·28 (0·26 – 0·29)	0 (0 – 0)	0 (0 – 0)
Slovenia	100 (93 – 107)	9·87 (9·49 – 10·25)	0 (0 – 0)	4347 (3986 – 4707)	0·25 (0·23 – 0·26)	0 (0 – 0)	0 (0 – 0)
Eastern Europe	‥	12·12 (11·51 – 12·8)	37 703 (11 923 – 60 466)	‥	0·26 (0·25 – 0·27)	537 (-378 – 1583)	1719 (-20 – 3699)
Belarus	23 (22 – 25)	14·85 (14·32 – 15·42)	0 (0 – 0)	1348 (1122 – 1629)	0·29 (0·24 – 0·35)	4 (-16 – 21)	6 (-21 – 29)
Estonia	66 (61 – 72)	10·29 (9·92 – 10·66)	3 (-59 – 63)	3052 (2793 – 3329)	0·24 (0·23 – 0·26)	1 (-1 – 4)	4 (-7 – 16)
Latvia	62 (57 – 69)	8·75 (8·34 – 9·17)	761 (480 – 1015)	2578 (2346 – 2813)	0·24 (0·23 – 0·25)	2 (-2 – 6)	53 (29 – 76)
Lithuania	53 (49 – 56)	12·3 (11·85 – 12·79)	0 (0 – 0)	2186 (1984 – 2401)	0·33 (0·31 – 0·35)	0 (0 – 0)	0 (0 – 0)

Moldova	17<br>(15 – 19)	7·79<br>(7·48 – 8·11)	1877<br>(1439 – 2296)	855<br>(760 – 966)	0·2<br>(0·19 – 0·21)	29<br>(18 – 40)	57<br>(43 – 73)
Russia	33<br>(31 – 36)	13·75<br>(13·08 – 14·58)	0<br>(0 – 0)	1941<br>(1808 – 2089)	0·26<br>(0·25 – 0·27)	375<br>(-408 – 1322)	729<br>(-777 – 2588)
Ukraine	22<br>(18 – 26)	7·02<br>(6·07 – 8·37)	35 063<br>(9524 – 57 646)	694<br>(626 – 768)	0·26<br>(0·24 – 0·27)	126<br>(-127 – 428)	870<br>(203 – 1611)
High-income	..	11·79<br>(10·78 – 12·88)	144 294<br>(22 566 – 243 330)	..	0·2<br>(0·18 – 0·21)	3411<br>(817 – 5696)	88 366<br>(35 411 – 140 195)
Australasia	..	11·79<br>(11·34 – 12·25)	0<br>(0 – 0)	..	0·24<br>(0·22 – 0·26)	10<br>(5 – 13)	62<br>(36 – 87)
Australia	154<br>(148 – 162)	11·95<br>(11·49 – 12·43)	0<br>(0 – 0)	7334<br>(6669 – 8087)	0·24<br>(0·22 – 0·26)	0<br>(0 – 0)	0<br>(0 – 0)
New Zealand	132<br>(120 – 146)	10·93<br>(10·16 – 11·63)	0<br>(0 – 0)	6479<br>(5973 – 7007)	0·21<br>(0·21 – 0·22)	10<br>(5 – 13)	62<br>(36 – 87)
High-income Asia-Pacific	..	22·92<br>(20·6 – 24·92)	0<br>(0 – 0)	..	0·18<br>(0·16 – 0·21)	649<br>(-1137 – 2068)	7375<br>(-10193 – 24 970)
Brunei	68<br>(54 – 83)	15·62<br>(13·29 – 19·64)	0<br>(0 – 0)	5260<br>(4199 – 6551)	0·19<br>(0·15 – 0·22)	1<br>(0 – 2)	4<br>(-1 – 8)
Japan	76<br>(67 – 88)	22·42<br>(19·46 – 24·99)	0<br>(0 – 0)	10 335<br>(8572 – 12 472)	0·16<br>(0·13 – 0·19)	646<br>(-1142 – 2064)	7349<br>(-10095 – 24 915)
Singapore	79<br>(65 – 95)	22·68<br>(19·12 – 27·38)	0<br>(0 – 0)	9132<br>(7516 – 11 044)	0·18<br>(0·15 – 0·21)	2<br>(-8 – 9)	22<br>(-65 – 84)
South Korea	50<br>(47 – 53)	24·33<br>(23·24 – 25·44)	0<br>(0 – 0)	4878<br>(4492 – 5261)	0·24<br>(0·23 – 0·26)	0<br>(0 – 0)	0<br>(0 – 0)
High-income North America	..	8·79<br>(7·79 – 9·87)	44 453<br>(-19932 – 98 535)	..	0·17<br>(0·15 – 0·18)	1630<br>(778 – 2388)	57 367<br>(19 395 – 92 458)
Canada	157<br>(148 – 167)	12·99<br>(12·23 – 13·69)	0<br>(0 – 0)	10 103<br>(8448 – 12 088)	0·19<br>(0·16 – 0·23)	110<br>(-58 – 244)	1134<br>(-525 – 2626)
Greenland	..	8·7<br>(7·52 – 10·28)	8<br>(-2 – 15)	..	0·15<br>(0·12 – 0·18)	0<br>(0 – 1)	0<br>(0 – 0)
United States	478<br>(415 – 548)	8·33<br>(7·25 – 9·52)	44 445<br>(-19932 – 98 515)	22 543<br>(20 547 – 24 392)	0·17<br>(0·15 – 0·18)	1520<br>(782 – 2189)	56 233<br>(18 908 – 90 532)
Southern Latin America	..	5·92<br>(5·32 – 6·71)	61 151<br>(30 041 – 85 996)	..	0·12<br>(0·1 – 0·14)	1070<br>(420 – 1670)	12 288<br>(7386 – 17 117)
Argentina	86<br>(71 – 101)	6·1<br>(5·28 – 7·18)	36 716<br>(7876 – 60 261)	4211<br>(3507 – 5052)	0·12<br>(0·1 – 0·14)	649<br>(147 – 1098)	6051<br>(2387 – 9725)
Chile	131<br>(125 – 138)	5·48<br>(5·31 – 5·68)	21 412<br>(18 669 – 24 349)	5956<br>(5252 – 6721)	0·12<br>(0·11 – 0·14)	253<br>(91 – 400)	4338<br>(3142 – 5495)
Uruguay	118<br>(100 – 139)	6·08<br>(5·29 – 7·06)	3023<br>(859 – 4743)	9102<br>(7505 – 10 776)	0·08<br>(0·07 – 0·09)	167<br>(126 – 201)	1899<br>(1237 – 2541)
Western Europe	..	10·65<br>(9·86 – 11·58)	38 690<br>(4608 – 72 088)	..	0·24<br>(0·22 – 0·25)	53<br>(-37 – 140)	11 274<br>(1608 – 22 257)
Andorra	367<br>(311 – 429)	10·72<br>(9·35 – 12·3)	0<br>(0 – 0)	15 054<br>(12 308 – 18 392)	0·24<br>(0·2 – 0·29)	0<br>(0 – 0)	0<br>(0 – 0)
Austria	187<br>(179 – 196)	11·15<br>(10·71 – 11·61)	0<br>(0 – 0)	5357<br>(5044 – 5663)	0·37<br>(0·35 – 0·39)	0<br>(0 – 0)	0<br>(0 – 0)
Belgium	147<br>(131 – 162)	13·54<br>(12·39 – 15·04)	0<br>(0 – 0)	7869<br>(7298 – 8435)	0·24<br>(0·22 – 0·26)	0<br>(0 – 0)	0<br>(0 – 0)
Cyprus	110<br>(93 – 130)	8·51<br>(7·29 – 9·82)	183<br>(-131 – 432)	5938<br>(4956 – 7154)	0·15<br>(0·13 – 0·18)	2<br>(-4 – 7)	35<br>(-15 – 82)
Denmark	267<br>(256 – 281)	7·82<br>(7·55 – 8·09)	108<br>(-32 – 265)	9136<br>(8556 – 9721)	0·22<br>(0·21 – 0·23)	0<br>(0 – 0)	29<br>(-9 – 71)
Finland	229<br>(218 – 240)	7·16<br>(6·84 – 7·47)	1301<br>(506 – 2151)	6627<br>(6175 – 7104)	0·24<br>(0·22 – 0·25)	0<br>(0 – 0)	299<br>(114 – 507)
France	170<br>(163 – 178)	11·44<br>(11·01 – 11·89)	0<br>(0 – 0)	6576<br>(6049 – 7063)	0·28<br>(0·26 – 0·31)	0<br>(0 – 0)	0<br>(0 – 0)
Germany	159<br>(143 – 175)	13·86<br>(12·63 – 15·39)	0<br>(0 – 0)	6050<br>(5674 – 6445)	0·34<br>(0·33 – 0·36)	0<br>(0 – 0)	0<br>(0 – 0)
Greece	122<br>(102 – 146)	6·75<br>(5·79 – 7·93)	6358<br>(1353 – 10 464)	3176<br>(2623 – 3857)	0·25<br>(0·21 – 0·3)	0<br>(0 – 0)	800<br>(144 – 1486)
Iceland	180<br>(171 – 193)	10·14<br>(9·69 – 10·57)	0<br>(0 – 0)	9402<br>(8699 – 10 124)	0·18<br>(0·17 – 0·2)	0<br>(0 – 0)	1<br>(-2 – 4)
Ireland	170<br>(153 – 189)	13·09<br>(12·21 – 13·88)	0<br>(0 – 0)	10 189<br>(9131 – 11 362)	0·21<br>(0·19 – 0·22)	0<br>(0 – 0)	0<br>(0 – 0)
Israel	86<br>(73 – 99)	12·32<br>(10·77 – 14·4)	0<br>(0 – 0)	4183<br>(3879 – 4499)	0·24<br>(0·23 – 0·26)	0<br>(0 – 0)	0<br>(0 – 0)
Italy	103<br>(91 – 115)	12·73<br>(11·46 – 14·42)	0<br>(0 – 0)	7259<br>(6625 – 7897)	0·17<br>(0·16 – 0·19)	0<br>(0 – 0)	0<br>(0 – 0)
Luxembourg	274<br>(253 – 299)	10·79<br>(10·34 – 11·26)	0<br>(0 – 0)	12 262<br>(11 100 – 13 522)	0·22<br>(0·21 – 0·24)	0<br>(0 – 0)	0<br>(0 – 0)
Malta	76<br>(66 – 90)	18·82<br>(16·05 – 21·55)	0<br>(0 – 0)	7422<br>(6179 – 8997)	0·19<br>(0·15 – 0·23)	0<br>(0 – 0)	0<br>(0 – 0)
Netherlands	312<br>(282 – 343)	7·25<br>(6·67 – 7·89)	2394<br>(-976 – 5122)	12 313<br>(10 701 – 14 114)	0·17<br>(0·15 – 0·2)	7<br>(-27 – 33)	851<br>(-259 – 1874)
Norway	413<br>(388 – 438)	7·21<br>(6·92 – 7·51)	719<br>(105 – 1275)	10 060<br>(9375 – 10 820)	0·27<br>(0·26 – 0·29)	0<br>(0 – 0)	298<br>(41 – 544)
Portugal	139<br>(128 – 155)	7·01<br>(6·43 – 7·52)	0<br>(0 – 0)	6610<br>(6121 – 7147)	0·14<br>(0·14 – 0·15)	0<br>(0 – 0)	0<br>(0 – 0)
Spain	124<br>(110 – 140)	10·6<br>(9·45 – 11·9)	0<br>(0 – 0)	8027<br>(7567 – 8480)	0·16<br>(0·15 – 0·17)	43<br>(-32 – 119)	345<br>(-253 – 967)
Sweden	402<br>(343 – 467)	5·7<br>(4·87 – 6·63)	8530<br>(1882 – 14 483)	11 249<br>(9420 – 13 328)	0·19<br>(0·16 – 0·23)	0<br>(0 – 0)	3534<br>(653 – 6769)

Switzerland	398 (369 – 428)	7·88 (7·36 – 8·41)	1623 (-100 – 3412)	12 421 (11 587 – 13 301)	0·24 (0·22 – 0·25)	0 (0 – 0)	655 (-36 – 1417)
United Kingdom	243 (210 – 284)	7·01 (6·1 – 8·07)	17 474 (-2881 – 38 805)	9037 (7699 – 10 398)	0·18 (0·16 – 0·21)	1 (0 – 2)	4426 (-604 – 10 847)
Latin America and Caribbean	‥	6·47 (5·79 – 7·22)	429 733 (266 574 – 564 471)	‥	0·1 (0·09 – 0·11)	17 826 (13 460 – 21 257)	105 081 (76 735 – 132 864)
Andean Latin America	‥	7·78 (6·65 – 9·5)	2826 (-7415 – 10 904)	‥	0·1 (0·09 – 0·11)	1873 (1477 – 2209)	4916 (3505 – 6403)
Bolivia	25 (19 – 30)	7·42 (6·21 – 9·26)	2176 (-2786 – 5953)	1576 (1264 – 1899)	0·1 (0·08 – 0·12)	360 (256 – 449)	629 (355 – 886)
Ecuador	54 (43 – 67)	7·86 (6·57 – 9·5)	329 (-2353 – 2284)	3297 (2890 – 3777)	0·1 (0·1 – 0·11)	541 (496 – 588)	1811 (1503 – 2149)
Peru	38 (29 – 45)	7·86 (6·72 – 9·76)	321 (-5049 – 4640)	2499 (2011 – 3035)	0·09 (0·08 – 0·11)	972 (665 – 1230)	2477 (1435 – 3677)
Caribbean	‥	4·26 (3·66 – 4·9)	93 521 (73 791 – 111 812)	‥	0·1 (0·08 – 0·11)	1454 (1005 – 1836)	9127 (6428 – 12 198)
Antigua and Barbuda	111 (92 – 136)	5·29 (4·47 – 6·18)	125 (71 – 177)	4667 (3697 – 5695)	0·1 (0·08 – 0·12)	2 (1 – 3)	26 (15 – 38)
The Bahamas	136 (112 – 167)	6·13 (5·18 – 7·21)	277 (32 – 489)	4931 (3892 – 6170)	0·13 (0·11 – 0·16)	6 (3 – 9)	70 (26 – 114)
Barbados	164 (137 – 197)	3·34 (2·85 – 3·86)	802 (689 – 916)	2790 (2236 – 3425)	0·15 (0·13 – 0·18)	2 (-1 – 4)	139 (100 – 183)
Belize	55 (44 – 67)	3·81 (3·19 – 4·5)	764 (562 – 944)	2368 (1883 – 2884)	0·07 (0·06 – 0·09)	17 (12 – 20)	82 (57 – 108)
Bermuda	‥	5·96 (5·06 – 6·97)	63 (26 – 97)	‥	0·13 (0·11 – 0·15)	1 (0 – 1)	0 (0 – 0)
Cuba	90 (72 – 113)	4·29 (3·65 – 4·91)	24 042 (17 896 – 30 134)	2760 (2160 – 3595)	0·11 (0·09 – 0·13)	237 (45 – 406)	2884 (1733 – 4277)
Dominica	33 (27 – 40)	7·58 (6·41 – 8·85)	30 (-1 – 59)	1995 (1587 – 2447)	0·1 (0·09 – 0·12)	2 (1 – 3)	5 (2 – 8)
Dominican Republic	92 (77 – 112)	4·5 (3·84 – 5·26)	19 512 (13 665 – 24 658)	2760 (2180 – 3365)	0·12 (0·1 – 0·14)	201 (119 – 271)	2400 (1536 – 3377)
Grenada	66 (54 – 81)	4·55 (3·88 – 5·35)	197 (136 – 251)	2435 (1949 – 2959)	0·1 (0·08 – 0·12)	3 (2 – 4)	21 (14 – 30)
Guyana	20 (16 – 25)	6·09 (5·08 – 7·13)	647 (140 – 1113)	1796 (1393 – 2278)	0·06 (0·05 – 0·07)	44 (37 – 50)	93 (61 – 128)
Haiti	14 (11 – 17)	2·95 (2·46 – 3·44)	32 766 (28 089 – 37 127)	558 (447 – 679)	0·07 (0·06 – 0·08)	621 (487 – 726)	808 (605 – 1021)
Jamaica	72 (59 – 87)	2·75 (2·34 – 3·18)	8719 (7726 – 9666)	4186 (3349 – 5145)	0·04 (0·03 – 0·05)	215 (196 – 232)	1538 (1229 – 1873)
Puerto Rico	‥	8·71 (7·46 – 10·12)	606 (-925 – 1749)	‥	0·12 (0·1 – 0·14)	67 (13 – 114)	0 (0 – 0)
Saint Lucia	53 (43 – 64)	5·98 (5·08 – 6·96)	200 (85 – 305)	2565 (2036 – 3158)	0·1 (0·08 – 0·12)	5 (3 – 7)	24 (13 – 36)
Saint Vincent and the Grenadines	49 (41 – 60)	4·24 (3·62 – 4·96)	224 (162 – 280)	1799 (1457 – 2211)	0·09 (0·08 – 0·11)	4 (2 – 5)	18 (12 – 25)
Suriname	83 (68 – 101)	4·49 (3·81 – 5·21)	992 (698 – 1259)	3128 (2467 – 3886)	0·09 (0·08 – 0·11)	16 (8 – 22)	135 (83 – 189)
Trinidad and Tobago	239 (197 – 288)	3·73 (3·18 – 4·36)	3458 (2805 – 4063)	4557 (3604 – 5575)	0·15 (0·12 – 0·18)	9 (-1 – 18)	881 (599 – 1189)
Virgin Islands U·S·	‥	5·98 (5·03 – 7·05)	97 (27 – 163)	‥	0·14 (0·11 – 0·16)	1 (0 – 2)	0 (0 – 0)
Central Latin America	‥	5·94 (5·09 – 6·85)	288 197 (175 621 – 378 926)	‥	0·08 (0·07 – 0·09)	11 568 (9594 – 13 023)	75 430 (56 982 – 93 597)
Colombia	41 (34 – 50)	8·86 (7·54 – 10·35)	1830 (-5168 – 7560)	5897 (5084 – 6673)	0·05 (0·05 – 0·05)	3028 (2829 – 3190)	17 952 (14 757 – 20 748)
Costa Rica	136 (111 – 166)	4·38 (3·71 – 5·15)	9502 (6907 – 11 800)	6305 (5007 – 7685)	0·07 (0·06 – 0·09)	208 (152 – 250)	2635 (1844 – 3464)
El Salvador	88 (78 – 100)	2·61 (2·38 – 2·82)	21 395 (20 103 – 22 609)	6919 (5851 – 8226)	0·03 (0·02 – 0·03)	522 (497 – 544)	5520 (4741 – 6352)
Guatemala	32 (25 – 39)	5·98 (4·97 – 7·24)	11 135 (-1316 – 20 498)	2220 (1764 – 2695)	0·07 (0·06 – 0·08)	778 (579 – 932)	2110 (1283 – 2974)
Honduras	26 (21 – 32)	5·59 (4·63 – 6·59)	8993 (3415 – 13 725)	1696 (1311 – 2134)	0·07 (0·06 – 0·09)	386 (276 – 470)	904 (538 – 1268)
Mexico	95 (80 – 112)	4·99 (4·29 – 5·77)	177 619 (102 283 – 239 636)	4577 (3592 – 5576)	0·08 (0·07 – 0·1)	5106 (3677 – 6148)	40 779 (27 044 – 54 397)
Nicaragua	19 (16 – 24)	8·49 (7·13 – 9·97)	0 (0 – 0)	1907 (1511 – 2342)	0·07 (0·06 – 0·09)	275 (201 – 333)	529 (312 – 754)
Panama	54 (44 – 65)	13·79 (11·76 – 15·98)	0 (0 – 0)	4095 (3338 – 5056)	0·14 (0·12 – 0·16)	37 (15 – 57)	156 (54 – 270)
Venezuela	46 (37 – 58)	4·8 (4·01 – 5·65)	57 724 (41 471 – 71 072)	1717 (1376 – 2124)	0·1 (0·08 – 0·12)	1229 (1032 – 1385)	4845 (3500 – 6349)
Tropical Latin America	‥	7·27 (6·79 – 7·79)	45 188 (9685 – 80 636)	‥	0·12 (0·11 – 0·14)	2931 (1052 – 4428)	15 609 (6374 – 24 035)
Brazil	87 (77 – 97)	7·33 (6·85 – 7·85)	38 197 (5433 – 71 518)	4054 (3403 – 4761)	0·12 (0·11 – 0·14)	2682 (823 – 4150)	14 434 (5419 – 22 630)
Paraguay	58 (47 – 71)	5·51 (4·68 – 6·51)	6991 (2362 – 11 051)	3009 (2402 – 3707)	0·09 (0·07 – 0·1)	249 (194 – 296)	1175 (755 – 1624)
North Africa and Middle East	‥	7·13 (6·31 – 8·23)	480 071 (325 711 – 604 263)	‥	0·12 (0·11 – 0·14)	15 169 (10 603 – 18 663)	77 601 (50 229 – 104 246)
Afghanistan	6 (5 – 8)	5·61 (4·67 – 6·9)	37 604 (11 272 – 57 369)	599 (457 – 769)	0·08 (0·07 – 0·1)	1600 (1029 – 2083)	1228 (691 – 1800)

Algeria	70 (57 – 85)	5·61 (4·74 – 6·66)	31 065 (16 551 – 43 470)	3787 (2864 – 4793)	0·12 (0·1 – 0·16)	622 (265 – 945)	4655 (2368 – 6967)
Bahrain	103 (79 – 127)	10·69 (8·81 – 13·26)	8 (-11 – 27)	20 526 (15 697 – 25 631)	0·06 (0·05 – 0·07)	60 (45 – 71)	1252 (713 – 1761)
Egypt	18 (14 – 21)	9·29 (7·89 – 11·49)	533 (-3637 – 3872)	1087 (860 – 1345)	0·18 (0·15 – 0·21)	911 (459 – 1275)	1014 (436 – 1567)
Iran	67 (54 – 81)	7·23 (6·21 – 8·57)	7361 (-14925 – 28 503)	7217 (5582 – 9069)	0·08 (0·07 – 0·1)	3235 (2008 – 4095)	24 303 (11 874 – 36 526)
Iraq	74 (58 – 94)	2·33 (1·94 – 2·77)	125 446 (108 570 – 139 641)	2387 (1762 – 3169)	0·09 (0·07 – 0·11)	1437 (771 – 1927)	12 933 (8986 – 17 042)
Jordan	54 (43 – 67)	4·73 (3·97 – 5·69)	10 625 (4837 – 15 140)	1240 (962 – 1571)	0·25 (0·2 – 0·3)	34 (15 – 48)	628 (250 – 1023)
Kuwait	149 (117 – 183)	8·64 (7·24 – 10·59)	30 (-73 – 110)	10 690 (7952 – 14 003)	0·13 (0·1 – 0·16)	36 (6 – 61)	403 (51 – 804)
Lebanon	85 (67 – 105)	5·16 (4·34 – 6·15)	9506 (5086 – 13 213)	5140 (4048 – 6350)	0·1 (0·08 – 0·12)	187 (130 – 240)	1803 (1120 – 2516)
Libya	31 (24 – 39)	7·93 (6·81 – 9·44)	547 (-812 – 1721)	2182 (1628 – 2884)	0·13 (0·11 – 0·16)	75 (21 – 118)	188 (45 – 339)
Morocco	34 (27 – 42)	4·78 (3·97 – 5·83)	62 709 (37 816 – 83 787)	3715 (2898 – 4670)	0·05 (0·04 – 0·07)	2074 (1763 – 2316)	9970 (7170 – 12 970)
Oman	80 (62 – 101)	9·14 (7·59 – 11·27)	3 (-23 – 27)	14 217 (10 795 – 18 105)	0·06 (0·05 – 0·07)	210 (160 – 248)	3029 (1744 – 4399)
Palestine	12 (10 – 15)	5·21 (4·37 – 6·19)	1652 (-187 – 3334)	964 (721 – 1298)	0·08 (0·07 – 0·11)	193 (127 – 244)	211 (111 – 327)
Qatar	118 (89 – 151)	14·61 (11·97 – 17·89)	0 (0 – 0)	13 771 (9948 – 18 967)	0·13 (0·1 – 0·17)	16 (5 – 26)	227 (52 – 440)
Saudi Arabia	134 (110 – 161)	10·55 (9·09 – 12·28)	265 (-91 – 613)	13 062 (10 279 – 16 358)	0·12 (0·1 – 0·14)	440 (253 – 607)	5879 (2843 – 9190)
Sudan	15 (12 – 20)	5·5 (4·62 – 6·7)	43 870 (6945 – 69 732)	1197 (898 – 1595)	0·09 (0·07 – 0·11)	1403 (816 – 1849)	2429 (1259 – 3691)
Syria	14 (11 – 17)	5·06 (4·27 – 6·03)	44 330 (28 732 – 56 973)	673 (521 – 866)	0·13 (0·1 – 0·15)	394 (110 – 618)	886 (484 – 1292)
Tunisia	40 (32 – 48)	7·28 (6·22 – 8·71)	1820 (-1866 – 5231)	3401 (2684 – 4247)	0·1 (0·09 – 0·12)	286 (179 – 379)	1070 (558 – 1676)
Turkey	34 (30 – 39)	12·18 (11·65 – 12·72)	0 (0 – 0)	2190 (1886 – 2518)	0·22 (0·21 – 0·23)	251 (137 – 358)	551 (290 – 806)
United Arab Emirates	137 (104 – 173)	8·49 (6·81 – 10·95)	12 (-8 – 30)	13 130 (10 010 – 16 708)	0·09 (0·08 – 0·12)	131 (22 – 212)	1794 (232 – 3404)
Yemen	18 (14 – 23)	2·5 (2·22 – 2·81)	102 684 (93 336 – 111 066)	804 (581 – 1108)	0·07 (0·06 – 0·09)	1575 (1046 – 1979)	3148 (2298 – 4223)
South Asia	..	4·67 (3·93 – 5·48)	3 099 706 (2 152 185 – 3 975 235)	..	0·07 (0·06 – 0·08)	82 034 (70 568 – 91 590)	103 716 (79 656 – 130 388)
Bangladesh	5 (4 – 6)	4·39 (3·69 – 5·18)	325 237 (230 983 – 412 210)	326 (257 – 410)	0·07 (0·06 – 0·08)	8392 (6403 – 9888)	4379 (2978 – 5721)
Bhutan	14 (11 – 17)	5·91 (5·0 – 6·92)	701 (228 – 1101)	842 (667 – 1078)	0·1 (0·08 – 0·12)	20 (10 – 28)	28 (14 – 41)
India	14 (11 – 17)	4·59 (3·82 – 5·4)	2 524 223 (1 777 704 – 3 221 116)	875 (731 – 1048)	0·07 (0·07 – 0·08)	62 691 (54 733 – 69 966)	91 319 (69 745 – 115 220)
Nepal	13 (11 – 16)	2·65 (2·24 – 3·07)	92 524 (81 292 – 103 467)	518 (411 – 660)	0·07 (0·06 – 0·08)	1515 (1140 – 1809)	2002 (1513 – 2553)
Pakistan	6 (5 – 7)	5·76 (4·89 – 6·74)	157 021 (37 804 – 266 053)	525 (399 – 667)	0·07 (0·06 – 0·08)	9416 (7025 – 11 186)	5988 (3708 – 8358)
Southeast Asia East Asia and Oceania	..	6·69 (6·11 – 7·29)	1 345 835 (823 859 – 1 824 889)	..	0·14 (0·13 – 0·15)	48 720 (43 656 – 53 187)	273 613 (211 011 – 338 345)
East Asia	..	7·1 (6·6 – 7·59)	610 327 (350 490 – 871 210)	..	0·18 (0·17 – 0·2)	3892 (1290 – 6254)	42 736 (24 791 – 61 928)
China	59 (54 – 65)	6·73 (6·28 – 7·17)	609 789 (350 987 – 870 188)	1609 (1448 – 1790)	0·18 (0·17 – 0·2)	3515 (1149 – 5707)	42 091 (24 461 – 61 155)
North Korea	5 (4 – 6)	7·98 (6·79 – 9·38)	539 (-1176 – 1856)	247 (200 – 300)	0·14 (0·12 – 0·16)	282 (5 – 487)	74 (5 – 141)
Taiwan	45 (37 – 55)	31·05 (26·72 – 36·07)	0 (0 – 0)	5845 (4615 – 7279)	0·17 (0·14 – 0·2)	95 (-8 – 182)	570 (-38 – 1242)
Oceania	..	6·17 (5·19 – 7·76)	8902 (1341 – 13 598)	..	0·07 (0·06 – 0·09)	502 (375 – 612)	401 (249 – 544)
American Samoa	..	9·87 (8·28 – 11·83)	2 (-5 – 9)	..	0·09 (0·08 – 0·11)	2 (1 – 3)	0 (0 – 0)
Federated States of Micronesia	16 (13 – 19)	8·2 (6·88 – 9·65)	29 (6 – 48)	1153 (931 – 1393)	0·09 (0·07 – 0·11)	4 (2 – 5)	5 (3 – 7)
Fiji	19 (15 – 23)	8·78 (7·32 – 10·99)	292 (38 – 527)	1509 (1208 – 1794)	0·08 (0·07 – 0·1)	36 (25 – 46)	61 (34 – 87)
Guam	..	10·14 (8·73 – 11·96)	3 (-3 – 9)	..	0·1 (0·08 – 0·12)	4 (2 – 7)	0 (0 – 0)
Kiribati	12 (9 – 15)	6·55 (5·43 – 7·82)	56 (9 – 100)	894 (702 – 1124)	0·07 (0·06 – 0·09)	5 (4 – 6)	5 (3 – 8)
Marshall Islands	35 (27 – 42)	7·12 (5·89 – 8·91)	32 (6 – 57)	2591 (2096 – 3121)	0·07 (0·06 – 0·09)	3 (2 – 4)	10 (6 – 13)
Northern Mariana Islands	..	9·87 (8·18 – 12·47)	0 (0 – 0)	..	0·09 (0·07 – 0·11)	3 (2 – 5)	0 (0 – 0)
Papua New Guinea	8 (6 – 10)	5·68 (4·77 – 7·26)	7878 (896 – 12 049)	512 (419 – 626)	0·07 (0·06 – 0·09)	389 (288 – 477)	268 (166 – 373)
Samoa	20 (16 – 25)	7·61 (6·31 – 9·23)	28 (-2 – 59)	1475 (1184 – 1787)	0·08 (0·07 – 0·1)	8 (5 – 10)	12 (6 – 18)

Solomon Islands	9 (7 – 11)	6·18 (5·07 – 7·34)	410 (98 – 680)	693 (549 – 851)	0·07 (0·06 – 0·08)	31 (25 – 37)	26 (17 – 36)
Tonga	16 (13 – 19)	8·09 (6·86 – 9·81)	17 (-2 – 35)	1111 (915 – 1328)	0·09 (0·07 – 0·1)	4 (3 – 5)	5 (2 – 7)
Vanuatu	10 (7 – 12)	6·37 (5·33 – 7·83)	154 (-9 – 260)	641 (500 – 814)	0·08 (0·06 – 0·09)	12 (8 – 15)	9 (5 – 14)
Southeast Asia	··	5·73 (4·82 – 6·78)	726 606 (392 197 – 1 005 771)	··	0·04 (0·03 – 0·05)	44 326 (40 487 – 47 685)	230 477 (176 187 – 288 696)
Cambodia	19 (16 – 22)	5·1 (4·55 – 5·76)	18 984 (11 757 – 25 161)	2954 (2356 – 3614)	0·03 (0·02 – 0·03)	1301 (1224 – 1373)	4219 (3279 – 5320)
Indonesia	38 (30 – 48)	5·83 (4·7 – 7·24)	218 232 (41 722 – 349 883)	3996 (3056 – 5086)	0·04 (0·03 – 0·05)	15 566 (13 585 – 17 213)	71 776 (49 815 – 96 876)
Laos	17 (14 – 21)	4·48 (3·74 – 5·3)	8440 (3889 – 12 155)	2485 (1989 – 3082)	0·02 (0·02 – 0·03)	585 (547 – 622)	1614 (1239 – 2051)
Malaysia	53 (44 – 63)	12·69 (10·7 – 14·84)	0 (0 – 0)	11 232 (9156 – 13 672)	0·04 (0·04 – 0·05)	1835 (1633 – 2020)	20 808 (15 136 – 27 397)
Maldives	174 (137 – 218)	6·01 (5·04 – 7·11)	320 (66 – 534)	20 651 (16 123 – 25 683)	0·04 (0·03 – 0·04)	25 (23 – 27)	581 (420 – 756)
Mauritius	87 (72 – 105)	6·78 (5·73 – 7·96)	577 (-202 – 1224)	10 051 (8136 – 12 381)	0·04 (0·03 – 0·05)	89 (80 – 98)	960 (691 – 1260)
Myanmar	26 (21 – 32)	5·73 (4·82 – 6·77)	48 508 (12 788 – 79 482)	4124 (3287 – 5176)	0·03 (0·02 – 0·03)	4100 (3831 – 4343)	18 293 (13 745 – 23 713)
Philippines	24 (20 – 29)	6·94 (5·83 – 8·1)	24 186 (-30160 – 68 276)	4731 (3826 – 5748)	0·03 (0·02 – 0·03)	7853 (7338 – 8345)	37 888 (28 799 – 48 340)
Sri Lanka	28 (23 – 33)	6·38 (5·41 – 7·46)	12 851 (-276 – 24 820)	3268 (2639 – 3977)	0·04 (0·03 – 0·05)	1488 (1338 – 1621)	5256 (3823 – 6786)
Seychelles	99 (81 – 120)	5·22 (4·5 – 5·95)	106 (51 – 158)	9062 (7008 – 11 218)	0·04 (0·03 – 0·05)	7 (6 – 7)	73 (54 – 94)
Thailand	85 (71 – 102)	3·7 (3·13 – 4·29)	175 152 (141 624 – 207 443)	5170 (4124 – 6381)	0·04 (0·04 – 0·05)	4626 (4084 – 5097)	39 341 (29 999 – 49 447)
Timor-Leste	7 (6 – 9)	6·53 (5·49 – 7·85)	484 (-228 – 1053)	1114 (874 – 1424)	0·03 (0·03 – 0·04)	86 (80 – 93)	101 (74 – 134)
Vietnam	41 (35 – 50)	3·77 (3·17 – 4·38)	218 766 (171 330 – 263 469)	2981 (2422 – 3641)	0·04 (0·03 – 0·05)	6765 (6116 – 7321)	29 567 (22 860 – 36 602)
Sub-Saharan Africa	··	3·77 (3·22 – 4·42)	2 072 000 (1 474 034 – 2 572 415)	··	0·06 (0·05 – 0·07)	64 634 (55 294 – 71 686)	89 095 (67 313 – 110 054)
Central sub-Saharan Africa	··	3·5 (2·92 – 4·23)	267 423 (181 603 – 336 363)	··	0·07 (0·06 – 0·09)	6694 (5014 – 8036)	3246 (2085 – 4413)
Angola	17 (13 – 21)	4·07 (3·36 – 4·76)	38 311 (22 153 – 53 485)	676 (529 – 864)	0·08 (0·06 – 0·09)	1151 (771 – 1470)	1453 (864 – 2074)
Central African Republic	2 (2 – 3)	2·87 (2·36 – 3·66)	19 579 (14 811 – 22 747)	87 (68 – 106)	0·06 (0·05 – 0·07)	428 (348 – 497)	78 (57 – 100)
Congo (Brazzaville)	13 (10 – 16)	4·88 (4·12 – 5·96)	6574 (2541 – 9301)	552 (437 – 683)	0·09 (0·08 – 0·11)	171 (97 – 231)	185 (104 – 263)
Democratic Republic of the Congo	3 (2 – 4)	3·26 (2·68 – 4·0)	200 863 (136 758 – 249 521)	128 (101 – 158)	0·07 (0·06 – 0·08)	4875 (3657 – 5854)	1251 (848 – 1647)
Equatorial Guinea	81 (64 – 105)	5·67 (4·75 – 6·85)	841 (78 – 1443)	3478 (2649 – 4457)	0·1 (0·08 – 0·11)	27 (15 – 39)	169 (76 – 268)
Gabon	38 (30 – 46)	5·75 (4·79 – 7·02)	1255 (60 – 2065)	1447 (1170 – 1784)	0·11 (0·09 – 0·13)	41 (19 – 58)	110 (49 – 169)
Eastern sub-Saharan Africa	··	4·28 (3·67 – 5·01)	646 310 (424 291 – 834 526)	··	0·05 (0·05 – 0·06)	27 691 (24 498 – 30 109)	24 344 (18 542 – 29 807)
Burundi	2 (2 – 3)	5·98 (5·02 – 7·3)	4575 (-2314 – 10 459)	249 (199 – 308)	0·05 (0·04 – 0·06)	883 (746 – 996)	234 (161 – 317)
Comoros	8 (6 – 9)	5·91 (4·89 – 7·38)	500 (63 – 854)	265 (207 – 329)	0·15 (0·12 – 0·18)	10 (3 – 15)	7 (3 – 11)
Djibouti	10 (8 – 12)	4·89 (4·06 – 6·02)	723 (-31 – 1245)	622 (497 – 754)	0·06 (0·05 – 0·08)	50 (37 – 59)	39 (24 – 54)
Eritrea	2 (2 – 3)	5·08 (4·22 – 6·0)	5428 (2195 – 8501)	170 (131 – 216)	0·06 (0·05 – 0·08)	292 (227 – 343)	64 (40 – 89)
Ethiopia	6 (5 – 8)	3·94 (3·28 – 4·61)	185 627 (124 019 – 239 930)	667 (531 – 851)	0·03 (0·03 – 0·04)	8579 (7851 – 9196)	7004 (5146 – 8996)
Kenya	14 (11 – 16)	4·98 (4·24 – 5·94)	59 818 (25 628 – 85 627)	1048 (846 – 1245)	0·05 (0·04 – 0·06)	3011 (2579 – 3350)	3997 (2811 – 5128)
Madagascar	5 (4 – 6)	4·19 (3·44 – 5·3)	36 344 (8892 – 53 499)	386 (308 – 490)	0·05 (0·04 – 0·06)	1909 (1641 – 2134)	939 (640 – 1258)
Malawi	11 (10 – 14)	2·91 (2·39 – 3·48)	59 245 (47 953 – 69 838)	601 (489 – 710)	0·05 (0·04 – 0·06)	1559 (1373 – 1713)	1630 (1247 – 2008)
Mozambique	6 (5 – 7)	3·53 (2·93 – 4·27)	77 725 (55 009 – 96 600)	386 (309 – 472)	0·04 (0·04 – 0·05)	2657 (2333 – 2928)	1484 (1095 – 1876)
Rwanda	8 (7 – 8)	6·41 (5·86 – 7·0)	2931 (-492 – 6005)	1378 (1106 – 1718)	0·03 (0·02 – 0·04)	1006 (922 – 1080)	1420 (1053 – 1853)
Somalia	3 (2 – 4)	4·21 (3·49 – 5·13)	21 131 (12 835 – 27 870)	202 (158 – 251)	0·05 (0·04 – 0·07)	741 (622 – 840)	214 (150 – 279)
South Sudan	9 (7 – 10)	2·81 (2·34 – 3·47)	37 869 (26 627 – 45 893)	439 (348 – 537)	0·04 (0·04 – 0·06)	1249 (1064 – 1392)	880 (635 – 1115)
Tanzania	9 (8 – 11)	5·46 (4·54 – 6·56)	38 751 (117 – 68 702)	415 (328 – 515)	0·1 (0·08 – 0·12)	1677 (823 – 2266)	1086 (475 – 1617)
Uganda	11 (10 – 12)	4·17 (3·85 – 4·51)	64 696 (52 108 – 77 448)	780 (658 – 909)	0·05 (0·04 – 0·06)	3072 (2756 – 3340)	3110 (2503 – 3749)
Zambia	26 (20 – 32)	2·97 (2·42 – 3·68)	50 948 (39 196 – 59 634)	904 (721 – 1103)	0·07 (0·06 – 0·08)	998 (755 – 1193)	2236 (1576 – 2891)

Southern sub-Saharan Africa	‥	3·32 (2·82 – 3·9)	259 084 (215 283 – 295 104)	‥	0·13 (0·1 – 0·16)	2040 (1337 – 2567)	27 629 (19 791 – 35 882)
Botswana	109 (89 – 134)	3·92 (3·27 – 4·69)	5526 (3990 – 6819)	3777 (2935 – 4698)	0·09 (0·07 – 0·1)	89 (54 – 118)	956 (618 – 1303)
Lesotho	28 (22 – 34)	2·42 (2·0 – 2·92)	12 204 (10 957 – 13 282)	974 (787 – 1203)	0·06 (0·05 – 0·07)	206 (176 – 233)	545 (425 – 672)
Namibia	115 (93 – 139)	3·57 (3·01 – 4·26)	6385 (4896 – 7659)	2997 (2395 – 3697)	0·1 (0·09 – 0·13)	66 (34 – 93)	947 (617 – 1267)
South Africa	118 (98 – 140)	3·53 (2·99 – 4·17)	176 743 (144 847 – 203 084)	2060 (1513 – 2739)	0·15 (0·12 – 0·2)	525 (27 – 872)	22 179 (15 571 – 29 558)
Swaziland (eSwatini)	80 (65 – 100)	2·93 (2·43 – 3·53)	5048 (4243 – 5759)	5141 (4059 – 6549)	0·04 (0·03 – 0·04)	132 (121 – 142)	1096 (851 – 1378)
Zimbabwe	21 (17 – 25)	2·73 (2·28 – 3·31)	53 178 (44 352 – 60 430)	769 (611 – 951)	0·06 (0·05 – 0·08)	1022 (803 – 1193)	1907 (1398 – 2389)
Western sub-Saharan Africa	‥	3·49 (2·96 – 4·14)	899 182 (643 002 – 1 116 079)	‥	0·06 (0·05 – 0·07)	28 210 (23 827 – 31 521)	33 875 (24 832 – 42 419)
Benin	7 (5 – 8)	3·71 (3·13 – 4·39)	20 928 (13 087 – 27 541)	400 (320 – 481)	0·05 (0·04 – 0·06)	811 (686 – 913)	466 (334 – 598)
Burkina Faso	14 (12 – 17)	1·6 (1·32 – 1·89)	83 434 (77 019 – 89 755)	402 (325 – 499)	0·05 (0·04 – 0·06)	1670 (1441 – 1858)	1859 (1497 – 2257)
Cameroon	11 (9 – 14)	4·07 (3·32 – 5·14)	43 742 (20 566 – 59 451)	608 (489 – 735)	0·06 (0·05 – 0·07)	1551 (1256 – 1810)	1463 (966 – 1943)
Cape Verde	24 (19 – 28)	6·16 (5·2 – 7·44)	196 (-222 – 484)	1360 (1090 – 1638)	0·09 (0·07 – 0·1)	20 (12 – 25)	32 (15 – 49)
Chad	8 (7 – 10)	2·96 (2·47 – 3·79)	40 594 (27 140 – 49 276)	525 (412 – 659)	0·04 (0·03 – 0·05)	1397 (1237 – 1524)	1091 (791 – 1385)
Cote d'Ivoire	14 (11 – 17)	3·92 (3·26 – 4·72)	42 076 (24 036 – 56 909)	699 (571 – 862)	0·06 (0·05 – 0·08)	1397 (1116 – 1648)	1587 (1065 – 2123)
The Gambia	7 (6 – 9)	5·31 (4·39 – 6·33)	1180 (39 – 2194)	551 (438 – 669)	0·06 (0·05 – 0·07)	118 (92 – 138)	75 (46 – 103)
Ghana	18 (16 – 20)	4·79 (4·38 – 5·18)	34 030 (25 093 – 43 222)	1635 (1311 – 1954)	0·04 (0·04 – 0·05)	2062 (1858 – 2230)	3977 (3035 – 4935)
Guinea	5 (4 – 6)	3·45 (2·88 – 4·14)	30 386 (21 216 – 38 023)	282 (226 – 344)	0·05 (0·04 – 0·06)	999 (857 – 1117)	431 (314 – 546)
Guinea-Bissau	8 (7 – 10)	3·65 (3·05 – 4·35)	4171 (2852 – 5259)	460 (366 – 571)	0·06 (0·05 – 0·07)	129 (105 – 149)	96 (68 – 124)
Liberia	13 (10 – 16)	3·58 (2·99 – 4·36)	10 962 (7568 – 13 714)	736 (591 – 907)	0·06 (0·05 – 0·07)	331 (273 – 379)	391 (277 – 505)
Mali	12 (10 – 14)	2·08 (1·84 – 2·33)	72 214 (66 273 – 77 810)	680 (593 – 771)	0·03 (0·03 – 0·03)	2081 (1985 – 2176)	2272 (1969 – 2575)
Mauritania	13 (10 – 16)	5·25 (4·36 – 6·39)	4165 (1505 – 6481)	645 (514 – 791)	0·08 (0·07 – 0·1)	144 (90 – 187)	150 (90 – 213)
Niger	6 (5 – 7)	2·41 (1·97 – 3·0)	70 367 (55 784 – 81 752)	268 (211 – 327)	0·04 (0·04 – 0·05)	1981 (1725 – 2176)	937 (703 – 1165)
Nigeria	19 (15 – 24)	3·54 (2·93 – 4·3)	401 807 (256 047 – 525 965)	830 (650 – 1023)	0·06 (0·05 – 0·08)	11 451 (8912 – 13 477)	17 362 (11 607 – 23 177)
Sao Tome and Principe	17 (13 – 21)	4·94 (4·12 – 6·03)	178 (29 – 284)	793 (631 – 977)	0·09 (0·07 – 0·11)	7 (4 – 9)	9 (4 – 13)
Senegal	9 (8 – 11)	4·16 (3·51 – 4·94)	22 971 (12 950 – 31 275)	664 (531 – 811)	0·05 (0·04 – 0·06)	1048 (891 – 1165)	921 (673 – 1191)
Sierra Leone	8 (6 – 9)	6·14 (5·1 – 7·57)	2385 (-2435 – 5885)	815 (639 – 1009)	0·05 (0·04 – 0·06)	553 (474 – 613)	475 (323 – 632)
Togo	7 (6 – 8)	4·08 (3·43 – 4·93)	13 394 (7537 – 18 041)	400 (325 – 493)	0·06 (0·05 – 0·07)	460 (374 – 537)	283 (190 – 376)

Table S21. Sensitivity analysis of national unit costs and expenditures required to achieve Universal Health Coverage standard for utilisation adjusting for quality and types of services in 2016 in 2017 international dollars

Table displays four sets of national estimates organised by GBD region: 1) unit cost per outpatient visit increased by 28% and inpatient admission increased by 24% by country to reflect the cost of improved quality and types of services, 2) aggregate ratio of total outpatient visits and inpatient admissions to counterfactual DALYs, where the counterfactual DALYs standardised the burden of disease across countries by removing the effects of access and quality of health care, 3) estimates of additional services needed to achieve the UHC standard for utilisation calculated by age and sex category, and 4) total cost of additional services in 2017 international dollars including the cost of improve quality and types of services. The cost of scaling up to the UHC utilisation standard was done at the age-sex level, leading some countries to have a cost of UHC scale-up greater than zero despite their aggregate utilisation per counterfactual DALY being greater than the standard set by the Netherlands. For each result, the mean of 1,000 draws is reported, and in parenthesis the uncertainty interval defined as the 2.5th and 97.5th percentile of draws. DALY = disability-adjusted-life-year. GBD= Global Burden of Disease, UHC = Universal Health Coverage.

Table S21: Sensitivity analysis of national unit costs and expenditures required to achieve Universal Health Coverage standard for utilisation adjusting for quality and types of services in 2016 in 2017 international dollars

	Outpatient visits			Inpatient admissions			
	Unit cost per outpatient visit in 2017 PPP $ (95% UI)	Ratio of total outpatient visits to counterfactual DALYs (95% UI) (Table 1)	Additional outpatient visits to meet UHC standard (95% UI) (in thousands) (Table 1)	Unit cost per inpatient admission in 2017 PPP $ (95% UI)	Ratio of total inpatient admissions to counterfactual DALYs (95% UI) (Table 1)	Additional inpatient admissions to meet UHC standard (95% UI) (in thousands) (Table 1)	Total additional cost to meet UHC standards in millions 2017 PPP $ (95% UI)
Global	··	6·7 (6·04 – 7·41)	10 415 092 (7 809 849 – 12 742 483)	··	0·12 (0·11 – 0·13)	348 465 (306 581 – 383 242)	1 474 808 (1 120 782 – 1 824 451)
Low income	··	··	1 956 058 (1 552 914 – 2 280 749)	··	··	61 106 (54 415 – 66 067)	59 034 (46 761 – 70 379)
Lower middle income	··	··	6 224 613 (4 643 059 – 7 683 756)	··	··	214 966 (194 755 – 231 539)	569 875 (457 324 – 689 135)
Upper middle income	··	··	2 023 229 (1 418 055 – 2 614 556)	··	··	59 265 (46 625 – 70 696)	512 699 (394 128 – 626 702)
High income	··	··	211 192 (83 151 – 330 668)	··	··	13 128 (6499 – 18 933)	333 200 (190 419 – 476 728)
Central Europe, Eastern Europe, and Central Asia	··	10·82 (10·37 – 11·31)	116 530 (86 387 – 145 714)	··	0·24 (0·23 – 0·25)	4372 (2820 – 6017)	17 516 (13 237 – 22 049)
Central Asia	··	8·25 (7·95 – 8·54)	54 694 (48 267 – 61 347)	··	0·17 (0·17 – 0·18)	1745 (1556 – 1936)	8267 (7307 – 9219)
Armenia	48 (40 – 57)	4·86 (4·67 – 5·05)	5691 (5076 – 6307)	1710 (1426 – 2019)	0·16 (0·16 – 0·17)	63 (51 – 75)	489 (390 – 594)
Azerbaijan	62 (54 – 70)	5·8 (5·53 – 6·08)	9910 (8076 – 11 755)	4938 (4342 – 5613)	0·08 (0·08 – 0·09)	581 (539 – 621)	4379 (3721 – 5087)
Georgia	31 (26 – 38)	7·13 (6·13 – 8·43)	1736 (359 – 3125)	2408 (2128 – 2720)	0·11 (0·1 – 0·11)	209 (185 – 232)	702 (564 – 857)
Kazakhstan	39 (35 – 43)	8·27 (7·91 – 8·65)	2937 (1224 – 4814)	1872 (1699 – 2046)	0·19 (0·18 – 0·2)	217 (163 – 277)	653 (476 – 867)
Kyrgyzstan	18 (16 – 20)	4·06 (3·91 – 4·22)	14 137 (12 960 – 15 301)	525 (472 – 595)	0·17 (0·17 – 0·18)	83 (60 – 103)	375 (325 – 436)
Mongolia	22 (18 – 26)	6·39 (5·38 – 7·61)	1669 (388 – 2868)	907 (717 – 1126)	0·18 (0·15 – 0·22)	32 (14 – 48)	85 (43 – 133)
Tajikistan	9 (8 – 10)	5·69 (5·44 – 5·96)	8781 (7148 – 10 447)	410 (368 – 459)	0·15 (0·15 – 0·16)	162 (130 – 192)	181 (147 – 219)
Turkmenistan	78 (68 – 89)	4·81 (4·6 – 5·02)	9620 (8440 – 10 757)	2182 (1706 – 2668)	0·2 (0·16 – 0·24)	44 (16 – 66)	1086 (888 – 1309)
Uzbekistan	10 (9 – 11)	11·85 (11·37 – 12·32)	212 (-318 – 764)	717 (648 – 790)	0·21 (0·2 – 0·22)	353 (275 – 431)	316 (239 – 396)
Central Europe	··	10·21 (9·83 – 10·6)	25 786 (19 317 – 32 104)	··	0·25 (0·23 – 0·27)	737 (344 – 1109)	4516 (3001 – 6128)
Albania	36 (30 – 44)	6·98 (5·92 – 8·13)	1189 (-163 – 2405)	2546 (2275 – 2879)	0·12 (0·11 – 0·12)	129 (110 – 147)	470 (351 – 591)
Bosnia and Herzegovina	25 (21 – 29)	12·28 (10·83 – 14·43)	0 (0 – 0)	2550 (2104 – 3087)	0·14 (0·12 – 0·16)	115 (36 – 185)	378 (102 – 697)
Bulgaria	68 (63 – 74)	7·17 (6·7 – 7·63)	3478 (1720 – 5113)	1592 (1460 – 1728)	0·35 (0·33 – 0·37)	0 (0 – 0)	306 (151 – 458)
Croatia	69 (63 – 76)	8·06 (7·74 – 8·42)	965 (417 – 1483)	2753 (2489 – 3067)	0·23 (0·21 – 0·24)	41 (18 – 64)	226 (109 – 348)
Czech Republic	64 (59 – 69)	14·1 (13·48 – 14·84)	0 (0 – 0)	3035 (2546 – 3547)	0·32 (0·28 – 0·38)	0 (0 – 0)	0 (0 – 0)
Hungary	42 (38 – 45)	15·6 (15·03 – 16·24)	0 (0 – 0)	2764 (2528 – 3013)	0·26 (0·24 – 0·28)	24 (-35 – 81)	84 (-114 – 284)
Macedonia	33 (28 – 38)	8·32 (7·93 – 8·72)	469 (321 – 613)	2081 (1800 – 2521)	0·15 (0·14 – 0·16)	57 (38 – 74)	168 (108 – 233)
Montenegro	33 (30 – 36)	9·39 (8·79 – 9·95)	20 (-21 – 60)	2264 (2067 – 2469)	0·16 (0·15 – 0·17)	14 (10 – 17)	40 (27 – 52)
Poland	62 (57 – 67)	10·21 (9·78 – 10·63)	0 (0 – 0)	2940 (2679 – 3219)	0·24 (0·22 – 0·25)	77 (-95 – 246)	283 (-331 – 905)
Romania	58 (53 – 65)	6·11 (5·83 – 6·38)	19 296 (15 002 – 23 587)	1393 (1231 – 1568)	0·29 (0·27 – 0·32)	10 (-60 – 74)	1470 (1063 – 1894)
Serbia	44 (40 – 48)	9·3 (8·93 – 9·65)	368 (-307 – 987)	3155 (2844 – 3495)	0·15 (0·14 – 0·16)	263 (204 – 320)	1057 (792 – 1354)
Slovakia	47 (43 – 52)	16·97 (16·28 – 17·65)	0 (0 – 0)	3164 (2865 – 3499)	0·28 (0·26 – 0·29)	5 (-6 – 15)	18 (-25 – 60)
Slovenia	100 (93 – 107)	9·87 (9·49 – 10·25)	0 (0 – 0)	4347 (3986 – 4707)	0·25 (0·23 – 0·26)	3 (-3 – 8)	15 (-14 – 46)
Eastern Europe	··	12·12 (11·51 – 12·8)	36 050 (12 837 – 57 240)	··	0·26 (0·25 – 0·27)	1889 (666 – 3266)	4733 (1835 – 7930)
Belarus	23 (22 – 25)	14·85 (14·32 – 15·42)	0 (0 – 0)	1348 (1122 – 1629)	0·29 (0·24 – 0·35)	54 (-74 – 149)	92 (-125 – 266)
Estonia	66 (61 – 72)	10·29 (9·92 – 10·66)	0 (0 – 0)	3052 (2793 – 3329)	0·24 (0·23 – 0·26)	11 (5 – 17)	40 (18 – 64)
Latvia	62 (57 – 69)	8·75 (8·34 – 9·17)	737 (485 – 985)	2578 (2346 – 2813)	0·24 (0·23 – 0·25)	16 (6 – 27)	110 (61 – 162)

Lithuania	53 (49 – 56)	12·3 (11·85 – 12·79)	0 (0 – 0)	2186 (1984 – 2401)	0·33 (0·31 – 0·35)	1 (-1 – 2)	2 (-2 – 6)
Moldova	17 (15 – 19)	7·79 (7·48 – 8·11)	1723 (1241 – 2196)	855 (760 – 966)	0·2 (0·19 – 0·21)	70 (56 – 84)	112 (89 – 140)
Russia	33 (31 – 36)	13·75 (13·08 – 14·58)	0 (0 – 0)	1941 (1808 – 2089)	0·26 (0·25 – 0·27)	1237 (248 – 2429)	2980 (583 – 5994)
Ukraine	22 (18 – 26)	7·02 (6·07 – 8·37)	33 590 (10 504 – 54 226)	694 (626 – 768)	0·26 (0·24 – 0·27)	501 (143 – 896)	1396 (546 – 2353)
High-income	..	11·79 (10·78 – 12·88)	249 199 (94 015 – 391 261)	..	0·2 (0·18 – 0·21)	12 493 (5931 – 18 151)	315 274 (176 967 – 454 259)
Australasia	..	11·79 (11·34 – 12·25)	0 (0 – 0)	..	0·24 (0·22 – 0·26)	45 (6 – 78)	370 (44 – 658)
Australia	154 (148 – 162)	11·95 (11·49 – 12·43)	0 (0 – 0)	7334 (6669 – 8087)	0·24 (0·22 – 0·26)	4 (-31 – 33)	39 (-255 – 308)
New Zealand	132 (120 – 146)	10·93 (10·16 – 11·63)	0 (0 – 0)	6479 (5973 – 7007)	0·21 (0·21 – 0·22)	41 (28 – 55)	331 (214 – 439)
High-income Asia-Pacific	..	22·92 (20·6 – 24·92)	0 (0 – 0)	..	0·18 (0·16 – 0·21)	3195 (626 – 5512)	42 636 (6966 – 83 024)
Brunei	68 (54 – 83)	15·62 (13·29 – 19·64)	0 (0 – 0)	5260 (4199 – 6551)	0·19 (0·15 – 0·22)	3 (0 – 5)	19 (-3 – 40)
Japan	76 (67 – 88)	22·42 (19·46 – 24·99)	0 (0 – 0)	10 335 (8572 – 12 472)	0·16 (0·13 – 0·19)	3169 (642 – 5453)	42 337 (6947 – 82 194)
Singapore	79 (65 – 95)	22·68 (19·12 – 27·38)	0 (0 – 0)	9132 (7516 – 11 044)	0·18 (0·15 – 0·21)	23 (-22 – 60)	280 (-241 – 815)
South Korea	50 (47 – 53)	24·33 (23·24 – 25·44)	0 (0 – 0)	4878 (4492 – 5261)	0·24 (0·23 – 0·26)	0 (0 – 0)	0 (0 – 0)
High-income North America	..	8·79 (7·79 – 9·87)	119 433 (27 473 – 197 248)	..	0·17 (0·15 – 0·18)	5014 (2949 – 6919)	209 303 (116 049 – 302 869)
Canada	157 (148 – 167)	12·99 (12·23 – 13·69)	0 (0 – 0)	10 103 (8448 – 12 088)	0·19 (0·16 – 0·23)	302 (97 – 465)	3840 (762 – 6691)
Greenland	..	8·7 (7·52 – 10·28)	15 (-4 – 29)	..	0·15 (0·12 – 0·18)	1 (1 – 1)	0 (0 – 0)
United States	478 (415 – 548)	8·33 (7·25 – 9·52)	119 418 (27 474 – 197 217)	22 543 (20 547 – 24 392)	0·17 (0·15 – 0·18)	4710 (2677 – 6590)	205 463 (113 585 – 298 052)
Southern Latin America	..	5·92 (5·32 – 6·71)	77 103 (38 426 – 104 635)	..	0·12 (0·1 – 0·14)	2402 (1510 – 3199)	26 085 (17 426 – 34 709)
Argentina	86 (71 – 101)	6·1 (5·28 – 7·18)	47 282 (12 243 – 72 301)	4211 (3507 – 5052)	0·12 (0·1 – 0·14)	1529 (822 – 2167)	13 559 (7240 – 20 294)
Chile	131 (125 – 138)	5·48 (5·31 – 5·68)	26 005 (22 813 – 29 141)	5956 (5252 – 6721)	0·12 (0·11 – 0·14)	618 (397 – 806)	9038 (6843 – 11 259)
Uruguay	118 (100 – 139)	6·08 (5·29 – 7·06)	3816 (1301 – 5880)	9102 (7505 – 10 776)	0·08 (0·07 – 0·09)	255 (216 – 288)	3488 (2435 – 4508)
Western Europe	..	10·65 (9·86 – 11·58)	52 663 (6711 – 97 539)	..	0·24 (0·22 – 0·25)	1837 (233 – 3139)	36 880 (13 048 – 62 021)
Andorra	367 (311 – 429)	10·72 (9·35 – 12·3)	0 (0 – 0)	15 054 (12 308 – 18 392)	0·24 (0·2 – 0·29)	0 (0 – 0)	0 (0 – 0)
Austria	187 (179 – 196)	11·15 (10·71 – 11·61)	29 (-257 – 308)	5357 (5044 – 5663)	0·37 (0·35 – 0·39)	0 (0 – 0)	9 (-73 – 89)
Belgium	147 (131 – 162)	13·54 (12·39 – 15·04)	0 (0 – 0)	7869 (7298 – 8435)	0·24 (0·22 – 0·26)	5 (-15 – 22)	53 (-143 – 216)
Cyprus	110 (93 – 130)	8·51 (7·29 – 9·82)	246 (-39 – 524)	5938 (4956 – 7154)	0·15 (0·13 – 0·18)	16 (-1 – 31)	159 (21 – 309)
Denmark	267 (256 – 281)	7·82 (7·55 – 8·09)	885 (280 – 1498)	9136 (8556 – 9721)	0·22 (0·21 – 0·23)	2 (-5 – 9)	319 (62 – 589)
Finland	229 (218 – 240)	7·16 (6·84 – 7·47)	1777 (908 – 2645)	6627 (6175 – 7104)	0·24 (0·22 – 0·25)	0 (-1 – 1)	533 (266 – 821)
France	170 (163 – 178)	11·44 (11·01 – 11·89)	0 (0 – 0)	6576 (6049 – 7063)	0·28 (0·26 – 0·31)	0 (0 – 0)	0 (0 – 0)
Germany	159 (143 – 175)	13·86 (12·63 – 15·39)	0 (0 – 0)	6050 (5674 – 6445)	0·34 (0·33 – 0·36)	0 (0 – 0)	0 (0 – 0)
Greece	122 (102 – 146)	6·75 (5·79 – 7·93)	7123 (1258 – 12 279)	3176 (2623 – 3857)	0·25 (0·21 – 0·3)	0 (0 – 0)	1162 (169 – 2247)
Iceland	180 (171 – 193)	10·14 (9·69 – 10·57)	0 (0 – 0)	9402 (8699 – 10 124)	0·18 (0·17 – 0·2)	2 (1 – 3)	24 (11 – 37)
Ireland	170 (153 – 189)	13·09 (12·21 – 13·88)	0 (0 – 0)	10 189 (9131 – 11 362)	0·21 (0·19 – 0·22)	0 (-2 – 3)	5 (-31 – 39)
Israel	86 (73 – 99)	12·32 (10·77 – 14·4)	0 (0 – 0)	4183 (3879 – 4499)	0·24 (0·23 – 0·26)	0 (0 – 0)	0 (0 – 0)
Italy	103 (91 – 115)	12·73 (11·46 – 14·42)	0 (0 – 0)	7259 (6625 – 7897)	0·17 (0·16 – 0·19)	546 (-33 – 1077)	4993 (-157 – 10 319)
Luxembourg	274 (253 – 299)	10·79 (10·34 – 11·26)	0 (0 – 0)	12 262 (11 100 – 13 522)	0·22 (0·21 – 0·24)	0 (0 – 0)	0 (-1 – 1)
Malta	76 (66 – 90)	18·82 (16·05 – 21·55)	0 (0 – 0)	7422 (6179 – 8997)	0·19 (0·15 – 0·23)	1 (-3 – 4)	8 (-27 – 37)
Netherlands	312 (282 – 343)	7·25 (6·67 – 7·89)	0 (0 – 0)	12 313 (10 701 – 14 114)	0·17 (0·15 – 0·2)	0 (0 – 0)	0 (0 – 0)
Norway	413 (388 – 438)	7·21 (6·92 – 7·51)	1275 (374 – 2084)	10 060 (9375 – 10 820)	0·27 (0·26 – 0·29)	0 (-2 – 3)	691 (268 – 1148)
Portugal	139 (128 – 155)	7·01 (6·43 – 7·52)	4178 (1393 – 7291)	6610 (6121 – 7147)	0·14 (0·14 – 0·15)	291 (215 – 372)	3158 (2209 – 4182)
Spain	124 (110 – 140)	10·6 (9·45 – 11·9)	0 (0 – 0)	8027 (7567 – 8480)	0·16 (0·15 – 0·17)	602 (366 – 842)	6003 (3508 – 8687)

Sweden	402 (343 – 467)	5·7 (4·87 – 6·63)	11 494 (4594 – 17 687)	11 249 (9420 – 13 328)	0·19 (0·16 – 0·23)	61 (-91 – 179)	6976 (2295 – 11 868)
Switzerland	398 (369 – 428)	7·88 (7·36 – 8·41)	2280 (458 – 3955)	12 421 (11 587 – 13 301)	0·24 (0·22 – 0·25)	0 (0 – 0)	1177 (253 – 2092)
United Kingdom	243 (210 – 284)	7·01 (6·1 – 8·07)	23 375 (-7052 – 52 671)	9037 (7699 – 10 398)	0·18 (0·16 – 0·21)	311 (-547 – 945)	11 610 (-2044 – 27 094)
Latin America and Caribbean	··	6·47 (5·79 – 7·22)	615 322 (410 489 – 803 049)	··	0·1 (0·09 – 0·11)	28 433 (23 133 – 32 514)	201 383 (152 282 – 248 921)
Andean Latin America	··	7·78 (6·65 – 9·5)	11 992 (-10962 – 30 524)	··	0·1 (0·09 – 0·11)	2869 (2402 – 3267)	9773 (7049 – 12 430)
Bolivia	25 (19 – 30)	7·42 (6·21 – 9·26)	4481 (-1078 – 9010)	1576 (1264 – 1899)	0·1 (0·08 – 0·12)	542 (432 – 638)	1215 (783 – 1647)
Ecuador	54 (43 – 67)	7·86 (6·57 – 9·5)	2906 (-4405 – 8852)	3297 (2890 – 3777)	0·1 (0·1 – 0·11)	810 (759 – 863)	3549 (2807 – 4316)
Peru	38 (29 – 45)	7·86 (6·72 – 9·76)	4605 (-8218 – 15 468)	2499 (2011 – 3035)	0·09 (0·08 – 0·11)	1518 (1145 – 1831)	5009 (3098 – 6982)
Caribbean	··	4·26 (3·66 – 4·9)	113 711 (93 323 – 132 567)	··	0·1 (0·08 – 0·11)	2427 (1909 – 2836)	15 563 (11 535 – 20 016)
Antigua and Barbuda	111 (92 – 136)	5·29 (4·47 – 6·18)	157 (99 – 209)	4667 (3697 – 5695)	0·1 (0·08 – 0·12)	4 (3 – 5)	48 (32 – 66)
The Bahamas	136 (112 – 167)	6·13 (5·18 – 7·21)	423 (194 – 609)	4931 (3892 – 6170)	0·13 (0·11 – 0·16)	12 (8 – 16)	153 (86 – 216)
Barbados	164 (137 – 197)	3·34 (2·85 – 3·86)	887 (770 – 997)	2790 (2236 – 3425)	0·15 (0·13 – 0·18)	6 (2 – 10)	211 (154 – 272)
Belize	55 (44 – 67)	3·81 (3·19 – 4·5)	996 (784 – 1183)	2368 (1883 – 2884)	0·07 (0·06 – 0·09)	25 (20 – 28)	143 (104 – 182)
Bermuda	··	5·96 (5·06 – 6·97)	88 (49 – 124)	··	0·13 (0·11 – 0·15)	2 (1 – 3)	0 (0 – 0)
Cuba	90 (72 – 113)	4·29 (3·65 – 4·91)	27 201 (21 141 – 33 629)	2760 (2160 – 3595)	0·11 (0·09 – 0·13)	527 (327 – 704)	5049 (3345 – 7074)
Dominica	33 (27 – 40)	7·58 (6·41 – 8·85)	54 (22 – 86)	1995 (1587 – 2447)	0·1 (0·09 – 0·12)	4 (3 – 5)	12 (7 – 17)
Dominican Republic	92 (77 – 112)	4·5 (3·84 – 5·26)	24 490 (18 527 – 29 940)	2760 (2180 – 3365)	0·12 (0·1 – 0·14)	388 (255 – 494)	4300 (2908 – 5829)
Grenada	66 (54 – 81)	4·55 (3·88 – 5·35)	248 (185 – 303)	2435 (1949 – 2959)	0·1 (0·08 – 0·12)	6 (4 – 7)	39 (27 – 52)
Guyana	20 (16 – 25)	6·09 (5·08 – 7·13)	1012 (517 – 1479)	1796 (1393 – 2278)	0·06 (0·05 – 0·07)	62 (55 – 68)	166 (115 – 220)
Haiti	14 (11 – 17)	2·95 (2·46 – 3·44)	40 783 (35 773 – 45 780)	558 (447 – 679)	0·07 (0·06 – 0·08)	882 (747 – 981)	1343 (1033 – 1673)
Jamaica	72 (59 – 87)	2·75 (2·34 – 3·18)	10 051 (9014 – 11 074)	4186 (3349 – 5145)	0·04 (0·03 – 0·05)	281 (261 – 300)	2410 (1948 – 2909)
Puerto Rico	··	8·71 (7·46 – 10·12)	1528 (-178 – 2836)	··	0·12 (0·1 – 0·14)	153 (96 – 202)	0 (0 – 0)
Saint Lucia	53 (43 – 64)	5·98 (5·08 – 6·96)	265 (153 – 368)	2565 (2036 – 3158)	0·1 (0·08 – 0·12)	9 (7 – 12)	49 (32 – 66)
Saint Vincent and the Grenadines	49 (41 – 60)	4·24 (3·62 – 4·96)	275 (212 – 329)	1799 (1457 – 2211)	0·09 (0·08 – 0·11)	6 (5 – 8)	32 (22 – 42)
Suriname	83 (68 – 101)	4·49 (3·81 – 5·21)	1226 (918 – 1518)	3128 (2467 – 3886)	0·09 (0·08 – 0·11)	29 (20 – 35)	244 (168 – 324)
Trinidad and Tobago	239 (197 – 288)	3·73 (3·18 – 4·36)	3899 (3215 – 4531)	4557 (3604 – 5575)	0·15 (0·12 – 0·18)	27 (10 – 40)	1363 (956 – 1814)
Virgin Islands U·S·	··	5·98 (5·03 – 7·05)	127 (60 – 184)	··	0·14 (0·11 – 0·16)	4 (2 – 6)	0 (0 – 0)
Central Latin America	··	5·94 (5·09 – 6·85)	380 121 (252 615 – 495 549)	··	0·08 (0·07 – 0·09)	16 581 (14 173 – 18 455)	131 340 (102 038 – 160 558)
Colombia	41 (34 – 50)	8·86 (7·54 – 10·35)	6139 (-14333 – 25 382)	5897 (5084 – 6673)	0·05 (0·05 – 0·05)	4122 (3892 – 4313)	30 549 (25 295 – 35 106)
Costa Rica	136 (111 – 166)	4·38 (3·71 – 5·15)	11 198 (8563 – 13 699)	6305 (5007 – 7685)	0·07 (0·06 – 0·09)	316 (258 – 359)	4471 (3270 – 5722)
El Salvador	88 (78 – 100)	2·61 (2·38 – 2·82)	24 501 (23 066 – 25 961)	6919 (5851 – 8226)	0·03 (0·02 – 0·03)	672 (646 – 698)	8560 (7348 – 9832)
Guatemala	32 (25 – 39)	5·98 (4·97 – 7·24)	18 928 (2626 – 31 078)	2220 (1764 – 2695)	0·07 (0·06 – 0·08)	1141 (944 – 1303)	3954 (2706 – 5330)
Honduras	26 (21 – 32)	5·59 (4·63 – 6·59)	13 067 (6947 – 18 450)	1696 (1311 – 2134)	0·07 (0·06 – 0·09)	567 (456 – 657)	1650 (1084 – 2212)
Mexico	95 (80 – 112)	4·99 (4·29 – 5·77)	236 409 (160 140 – 305 056)	4577 (3592 – 5576)	0·08 (0·07 – 0·1)	7645 (5842 – 8954)	73 106 (51 184 – 95 345)
Nicaragua	19 (16 – 24)	8·49 (7·13 – 9·97)	612 (-2376 – 2991)	1907 (1511 – 2342)	0·07 (0·06 – 0·09)	410 (336 – 468)	992 (642 – 1347)
Panama	54 (44 – 65)	13·79 (11·76 – 15·98)	0 (0 – 0)	4095 (3338 – 5056)	0·14 (0·12 – 0·16)	72 (18 – 112)	375 (91 – 695)
Venezuela	46 (37 – 58)	4·8 (4·01 – 5·65)	69 267 (52 395 – 84 376)	1717 (1376 – 2124)	0·1 (0·08 – 0·12)	1637 (1408 – 1841)	7682 (5659 – 9985)
Tropical Latin America	··	7·27 (6·79 – 7·79)	109 498 (63 846 – 156 000)	··	0·12 (0·11 – 0·14)	6556 (4321 – 8378)	44 708 (28 670 – 60 373)
Brazil	87 (77 – 97)	7·33 (6·85 – 7·85)	98 890 (56 314 – 140 448)	4054 (3403 – 4761)	0·12 (0·11 – 0·14)	6167 (3989 – 7942)	42 435 (26 910 – 57 578)
Paraguay	58 (47 – 71)	5·51 (4·68 – 6·51)	10 607 (5839 – 14 711)	3009 (2402 – 3707)	0·09 (0·07 – 0·1)	389 (304 – 455)	2273 (1543 – 3032)
North Africa and Middle East	··	7·13 (6·31 – 8·23)	676 422 (469 769 – 838 873)	··	0·12 (0·11 – 0·14)	23 918 (17 677 – 28 552)	148 985 (98 870 – 196 722)

Afghanistan	6 (5 – 8)	5·61 (4·67 – 6·9)	63 000 (28 873 – 87 927)	599 (457 – 769)	0·08 (0·07 – 0·1)	2490 (1879 – 2993)	2410 (1528 – 3342)
Algeria	70 (57 – 85)	5·61 (4·74 – 6·66)	47 746 (26 082 – 66 884)	3787 (2864 – 4793)	0·12 (0·1 – 0·16)	1108 (502 – 1609)	9789 (5124 – 14 669)
Bahrain	103 (79 – 127)	10·69 (8·81 – 13·26)	9 (-11 – 27)	20 526 (15 697 – 25 631)	0·06 (0·05 – 0·07)	87 (72 – 98)	2243 (1406 – 3031)
Egypt	18 (14 – 21)	9·29 (7·89 – 11·49)	1275 (-4466 – 6068)	1087 (860 – 1345)	0·18 (0·15 – 0·21)	1492 (553 – 2250)	2105 (660 – 3562)
Iran	67 (54 – 81)	7·23 (6·21 – 8·57)	21 686 (-20514 – 53 980)	7217 (5582 – 9069)	0·08 (0·07 – 0·1)	4995 (3755 – 5906)	47 357 (27 852 – 67 283)
Iraq	74 (58 – 94)	2·33 (1·94 – 2·77)	159 645 (142 270 – 174 339)	2387 (1762 – 3169)	0·09 (0·07 – 0·11)	2264 (1420 – 2905)	22 178 (15 909 – 28 796)
Jordan	54 (43 – 67)	4·73 (3·97 – 5·69)	15 825 (9757 – 20 581)	1240 (962 – 1571)	0·25 (0·2 – 0·3)	28 (11 – 43)	1159 (593 – 1759)
Kuwait	149 (117 – 183)	8·64 (7·24 – 10·59)	217 (-427 – 747)	10 690 (7952 – 14 003)	0·13 (0·1 – 0·16)	69 (22 – 108)	1005 (255 – 1871)
Lebanon	85 (67 – 105)	5·16 (4·34 – 6·15)	12 180 (7591 – 15 958)	5140 (4048 – 6350)	0·1 (0·08 – 0·12)	315 (228 – 388)	3392 (2250 – 4542)
Libya	31 (24 – 39)	7·93 (6·81 – 9·44)	1932 (-1693 – 4475)	2182 (1628 – 2884)	0·13 (0·11 – 0·16)	158 (64 – 240)	532 (177 – 935)
Morocco	34 (27 – 42)	4·78 (3·97 – 5·83)	77 838 (52 509 – 97 910)	3715 (2898 – 4670)	0·05 (0·04 – 0·07)	2867 (2547 – 3136)	16 808 (12 491 – 21 569)
Oman	80 (62 – 101)	9·14 (7·59 – 11·27)	19 (-75 – 104)	14 217 (10 795 – 18 105)	0·06 (0·05 – 0·07)	300 (250 – 339)	5361 (3375 – 7533)
Palestine	12 (10 – 15)	5·21 (4·37 – 6·19)	4803 (896 – 8395)	964 (721 – 1298)	0·08 (0·07 – 0·11)	278 (170 – 361)	420 (209 – 655)
Qatar	118 (89 – 151)	14·61 (11·97 – 17·89)	0 (0 – 0)	13 771 (9948 – 18 967)	0·13 (0·1 – 0·17)	29 (5 – 47)	535 (88 – 1034)
Saudi Arabia	134 (110 – 161)	10·55 (9·09 – 12·28)	637 (-1938 – 3394)	13 062 (10 279 – 16 358)	0·12 (0·1 – 0·14)	802 (437 – 1111)	13 432 (6028 – 21 525)
Sudan	15 (12 – 20)	5·5 (4·62 – 6·7)	73 204 (34 725 – 101 941)	1197 (898 – 1595)	0·09 (0·07 – 0·11)	2331 (1590 – 2886)	5022 (2977 – 7257)
Syria	14 (11 – 17)	5·06 (4·27 – 6·03)	61 729 (46 076 – 74 829)	673 (521 – 866)	0·13 (0·1 – 0·15)	846 (486 – 1160)	1814 (1141 – 2515)
Tunisia	40 (32 – 48)	7·28 (6·22 – 8·71)	3098 (-1641 – 7151)	3401 (2684 – 4247)	0·1 (0·09 – 0·12)	516 (360 – 649)	2399 (1409 – 3571)
Turkey	34 (30 – 39)	12·18 (11·65 – 12·72)	0 (0 – 0)	2190 (1886 – 2518)	0·22 (0·21 – 0·23)	358 (213 – 499)	980 (551 – 1423)
United Arab Emirates	137 (104 – 173)	8·49 (6·81 – 10·95)	402 (-827 – 1492)	13 130 (10 010 – 16 708)	0·09 (0·08 – 0·12)	272 (124 – 384)	4672 (1681 – 7953)
Yemen	18 (14 – 23)	2·5 (2·22 – 2·81)	131 176 (121 549 – 140 001)	804 (581 – 1108)	0·07 (0·06 – 0·09)	2312 (1786 – 2718)	5373 (4057 – 7092)
South Asia	··	4·67 (3·93 – 5·48)	4 027 525 (3 113 082 – 4 896 398)	··	0·07 (0·06 – 0·08)	120 653 (108 685 – 130 918)	182 221 (142 748 – 223 356)
Bangladesh	5 (4 – 6)	4·39 (3·69 – 5·18)	422 808 (323 953 – 514 241)	326 (257 – 410)	0·07 (0·06 – 0·08)	12 253 (10 232 – 13 694)	7683 (5583 – 9776)
Bhutan	14 (11 – 17)	5·91 (5·0 – 6·92)	1100 (605 – 1507)	842 (667 – 1078)	0·1 (0·08 – 0·12)	36 (24 – 45)	59 (36 – 82)
India	14 (11 – 17)	4·59 (3·82 – 5·4)	3 217 963 (2 493 450 – 3 913 540)	875 (731 – 1048)	0·07 (0·07 – 0·08)	92 448 (84 033 – 100 097)	159 993 (125 772 – 196 196)
Nepal	13 (11 – 16)	2·65 (2·24 – 3·07)	113 559 (102 332 – 124 285)	518 (411 – 660)	0·07 (0·06 – 0·08)	2207 (1843 – 2507)	3328 (2606 – 4151)
Pakistan	6 (5 – 7)	5·76 (4·89 – 6·74)	272 095 (150 396 – 379 250)	525 (399 – 667)	0·07 (0·06 – 0·08)	13 709 (11 176 – 15 491)	11 158 (7731 – 14 809)
Southeast Asia East Asia and Oceania	··	6·69 (6·11 – 7·29)	1 709 453 (1 060 336 – 2 339 121)	··	0·14 (0·13 – 0·15)	71 154 (62 987 – 78 765)	457 716 (359 683 – 562 325)
East Asia	··	7·1 (6·6 – 7·59)	753 139 (400 129 – 1 114 796)	··	0·18 (0·17 – 0·2)	12 175 (6354 – 17 677)	83 136 (50 065 – 118 027)
China	59 (54 – 65)	6·73 (6·28 – 7·17)	751 600 (401 537 – 1 113 190)	1609 (1448 – 1790)	0·18 (0·17 – 0·2)	11 360 (5698 – 16 459)	81 153 (49 400 – 115 644)
North Korea	5 (4 – 6)	7·98 (6·79 – 9·38)	1539 (-7288 – 7371)	247 (200 – 300)	0·14 (0·12 – 0·16)	579 (159 – 927)	195 (39 – 356)
Taiwan	45 (37 – 55)	31·05 (26·72 – 36·07)	0 (0 – 0)	5845 (4615 – 7279)	0·17 (0·14 – 0·2)	236 (-13 – 443)	1788 (-23 – 3806)
Oceania	··	6·17 (5·19 – 7·76)	13 061 (2801 – 19 123)	··	0·07 (0·06 – 0·09)	752 (620 – 860)	746 (505 – 967)
American Samoa	··	9·87 (8·28 – 11·83)	2 (-2 – 6)	··	0·09 (0·08 – 0·11)	4 (3 – 5)	0 (0 – 0)
Federated States of Micronesia	16 (13 – 19)	8·2 (6·88 – 9·65)	40 (-18 – 102)	1153 (931 – 1393)	0·09 (0·07 – 0·11)	6 (5 – 7)	10 (6 – 13)
Fiji	19 (15 – 23)	8·78 (7·32 – 10·99)	233 (-83 – 516)	1509 (1208 – 1794)	0·08 (0·07 – 0·1)	58 (47 – 67)	116 (74 – 156)
Guam	··	10·14 (8·73 – 11·96)	3 (-8 – 12)	··	0·1 (0·08 – 0·12)	8 (6 – 11)	0 (0 – 0)
Kiribati	12 (9 – 15)	6·55 (5·43 – 7·82)	88 (11 – 160)	894 (702 – 1124)	0·07 (0·06 – 0·09)	8 (6 – 9)	10 (7 – 14)
Marshall Islands	35 (27 – 42)	7·12 (5·89 – 8·91)	41 (-9 – 81)	2591 (2096 – 3121)	0·07 (0·06 – 0·09)	5 (4 – 6)	18 (11 – 24)
Northern Mariana Islands	··	9·87 (8·18 – 12·47)	0 (-1 – 1)	··	0·09 (0·07 – 0·11)	6 (4 – 7)	0 (0 – 0)
Papua New Guinea	8 (6 – 10)	5·68 (4·77 – 7·26)	11 686 (2535 – 16 966)	512 (419 – 626)	0·07 (0·06 – 0·09)	576 (474 – 664)	496 (328 – 660)

Samoa	20 (16 – 25)	7·61 (6·31 – 9·23)	50 (-36 – 134)	1475 (1184 – 1787)	0·08 (0·07 – 0·1)	12 (9 – 14)	23 (14 – 33)
Solomon Islands	9 (7 – 11)	6·18 (5·07 – 7·34)	652 (261 – 1022)	693 (549 – 851)	0·07 (0·06 – 0·08)	45 (38 – 51)	47 (32 – 63)
Tonga	16 (13 – 19)	8·09 (6·86 – 9·81)	22 (-32 – 63)	1111 (915 – 1328)	0·09 (0·07 – 0·1)	6 (5 – 7)	9 (6 – 13)
Vanuatu	10 (7 – 12)	6·37 (5·33 – 7·83)	244 (-5 – 403)	641 (500 – 814)	0·08 (0·06 – 0·09)	18 (14 – 21)	18 (11 – 25)
Southeast Asia	..	5·73 (4·82 – 6·78)	943 254 (591 879 – 1 254 765)	..	0·04 (0·03 – 0·05)	58 227 (54 094 – 61 475)	373 834 (291 350 – 461 503)
Cambodia	19 (16 – 22)	5·1 (4·55 – 5·76)	27 443 (19 330 – 34 554)	2954 (2356 – 3614)	0·03 (0·02 – 0·03)	1640 (1554 – 1723)	6705 (5282 – 8398)
Indonesia	38 (30 – 48)	5·83 (4·7 – 7·24)	295 299 (93 244 – 446 300)	3996 (3056 – 5086)	0·04 (0·03 – 0·05)	20 694 (18 605 – 22 305)	118 920 (85 727 – 158 392)
Laos	17 (14 – 21)	4·48 (3·74 – 5·3)	12 330 (7785 – 16 551)	2485 (1989 – 3082)	0·02 (0·02 – 0·03)	728 (689 – 768)	2536 (1987 – 3183)
Malaysia	53 (44 – 63)	12·69 (10·7 – 14·84)	0 (0 – 0)	11 232 (9156 – 13 672)	0·04 (0·04 – 0·05)	2447 (2230 – 2638)	34 399 (25 713 – 44 327)
Maldives	174 (137 – 218)	6·01 (5·04 – 7·11)	459 (192 – 687)	20 651 (16 123 – 25 683)	0·04 (0·03 – 0·04)	33 (31 – 35)	954 (701 – 1226)
Mauritius	87 (72 – 105)	6·78 (5·73 – 7·96)	851 (-14 – 1571)	10 051 (8136 – 12 381)	0·04 (0·03 – 0·05)	121 (111 – 129)	1611 (1201 – 2082)
Myanmar	26 (21 – 32)	5·73 (4·82 – 6·77)	69 602 (33 044 – 101 798)	4124 (3287 – 5176)	0·03 (0·02 – 0·03)	5260 (4990 – 5521)	29 417 (22 526 – 37 712)
Philippines	24 (20 – 29)	6·94 (5·83 – 8·1)	61 728 (-3412 – 114 239)	4731 (3826 – 5748)	0·03 (0·02 – 0·03)	10 025 (9471 – 10 537)	60 860 (46 878 – 76 519)
Sri Lanka	28 (23 – 33)	6·38 (5·41 – 7·46)	19 598 (7286 – 30 838)	3268 (2639 – 3977)	0·04 (0·03 – 0·05)	1979 (1825 – 2115)	8769 (6614 – 11 105)
Seychelles	99 (81 – 120)	5·22 (4·5 – 5·95)	141 (84 – 196)	9062 (7008 – 11 218)	0·04 (0·03 – 0·05)	9 (8 – 10)	120 (90 – 153)
Thailand	85 (71 – 102)	3·7 (3·13 – 4·29)	194 354 (161 103 – 226 590)	5170 (4124 – 6381)	0·04 (0·04 – 0·05)	6255 (5714 – 6751)	62 090 (48 310 – 77 068)
Timor-Leste	7 (6 – 9)	6·53 (5·49 – 7·85)	946 (44 – 1620)	1114 (874 – 1424)	0·03 (0·03 – 0·04)	111 (103 – 118)	165 (123 – 215)
Vietnam	41 (35 – 50)	3·77 (3·17 – 4·38)	260 504 (214 653 – 306 356)	2981 (2422 – 3641)	0·04 (0·03 – 0·05)	8926 (8260 – 9545)	47 288 (37 451 – 57 870)
Sub-Saharan Africa	..	3·77 (3·22 – 4·42)	3 020 640 (2 414 437 – 3 539 543)	..	0·06 (0·05 – 0·07)	87 442 (76 512 – 95 734)	151 714 (116 765 – 184 262)
Central sub-Saharan Africa	..	3·5 (2·92 – 4·23)	393 978 (301 686 – 465 275)	..	0·07 (0·06 – 0·09)	9590 (7859 – 11 045)	6246 (4399 – 8114)
Angola	17 (13 – 21)	4·07 (3·36 – 4·76)	63 953 (46 328 – 81 532)	676 (529 – 864)	0·08 (0·06 – 0·09)	1717 (1302 – 2081)	2868 (1956 – 3907)
Central African Republic	2 (2 – 3)	2·87 (2·36 – 3·66)	25 402 (20 393 – 28 715)	87 (68 – 106)	0·06 (0·05 – 0·07)	589 (507 – 653)	131 (98 – 164)
Congo (Brazzaville)	13 (10 – 16)	4·88 (4·12 – 5·96)	10 362 (6201 – 13 219)	552 (437 – 683)	0·09 (0·08 – 0·11)	282 (203 – 349)	374 (242 – 498)
Democratic Republic of the Congo	3 (2 – 4)	3·26 (2·68 – 4·0)	290 433 (227 403 – 340 120)	128 (101 – 158)	0·07 (0·06 – 0·08)	6881 (5634 – 7924)	2250 (1632 – 2867)
Equatorial Guinea	81 (64 – 105)	5·67 (4·75 – 6·85)	1471 (691 – 2070)	3478 (2649 – 4457)	0·1 (0·08 – 0·11)	47 (34 – 59)	368 (226 – 524)
Gabon	38 (30 – 46)	5·75 (4·79 – 7·02)	2358 (798 – 3489)	1447 (1170 – 1784)	0·11 (0·09 – 0·13)	75 (43 – 100)	255 (136 – 369)
Eastern sub-Saharan Africa	..	4·28 (3·67 – 5·01)	996 828 (743 228 – 1 207 013)	..	0·05 (0·05 – 0·06)	36 696 (33 170 – 39 410)	41 737 (32 773 – 50 268)
Burundi	2 (2 – 3)	5·98 (5·02 – 7·3)	11 871 (3913 – 18 542)	249 (199 – 308)	0·05 (0·04 – 0·06)	1158 (1015 – 1274)	400 (287 – 521)
Comoros	8 (6 – 9)	5·91 (4·89 – 7·38)	955 (243 – 1488)	265 (207 – 329)	0·15 (0·12 – 0·18)	15 (4 – 23)	15 (6 – 23)
Djibouti	10 (8 – 12)	4·89 (4·06 – 6·02)	1304 (408 – 1940)	622 (497 – 754)	0·06 (0·05 – 0·08)	70 (57 – 79)	71 (48 – 95)
Eritrea	2 (2 – 3)	5·08 (4·22 – 6·0)	9630 (5819 – 13 329)	170 (131 – 216)	0·06 (0·05 – 0·08)	408 (344 – 462)	118 (80 – 158)
Ethiopia	6 (5 – 8)	3·94 (3·28 – 4·61)	273 222 (206 661 – 334 516)	667 (531 – 851)	0·03 (0·03 – 0·04)	10 888 (10 182 – 11 519)	11 402 (8628 – 14 483)
Kenya	14 (11 – 16)	4·98 (4·24 – 5·94)	98 404 (62 012 – 127 388)	1048 (846 – 1245)	0·05 (0·04 – 0·06)	4086 (3634 – 4445)	7052 (5246 – 8748)
Madagascar	5 (4 – 6)	4·19 (3·44 – 5·3)	60 315 (32 355 – 79 371)	386 (308 – 490)	0·05 (0·04 – 0·06)	2491 (2225 – 2719)	1613 (1154 – 2105)
Malawi	11 (10 – 14)	2·91 (2·39 – 3·48)	80 086 (68 235 – 90 326)	601 (489 – 710)	0·05 (0·04 – 0·06)	2061 (1859 – 2214)	2734 (2161 – 3304)
Mozambique	6 (5 – 7)	3·53 (2·93 – 4·27)	111 554 (86 778 – 131 250)	386 (309 – 472)	0·04 (0·04 – 0·05)	3467 (3119 – 3768)	2497 (1912 – 3077)
Rwanda	8 (7 – 8)	6·41 (5·86 – 7·0)	9032 (3988 – 13 555)	1378 (1106 – 1718)	0·03 (0·02 – 0·04)	1267 (1188 – 1341)	2272 (1726 – 2919)
Somalia	3 (2 – 4)	4·21 (3·49 – 5·13)	31 363 (22 963 – 38 164)	202 (158 – 251)	0·05 (0·04 – 0·07)	1007 (886 – 1104)	373 (276 – 472)
South Sudan	9 (7 – 10)	2·81 (2·34 – 3·47)	53 687 (42 287 – 62 028)	439 (348 – 537)	0·04 (0·04 – 0·06)	1594 (1410 – 1743)	1467 (1109 – 1808)
Tanzania	9 (8 – 11)	5·46 (4·54 – 6·56)	82 667 (28 247 – 127 002)	415 (328 – 515)	0·1 (0·08 – 0·12)	2717 (1706 – 3547)	2449 (1278 – 3550)
Uganda	11 (10 – 12)	4·17 (3·85 – 4·51)	104 655 (89 660 – 119 680)	780 (658 – 909)	0·05 (0·04 – 0·06)	4041 (3717 – 4317)	5393 (4451 – 6380)

Zambia	26 (20 – 32)	2·97 (2·42 – 3·68)	68 083 (55 857 – 77 098)	904 (721 – 1103)	0·07 (0·06 – 0·08)	1426 (1181 – 1625)	3881 (2887 – 4887)
Southern sub-Saharan Africa	..	3·32 (2·82 – 3·9)	315 824 (272 681 – 351 952)	..	0·13 (0·1 – 0·16)	3230 (1217 – 4639)	44 537 (32 407 – 57 462)
Botswana	109 (89 – 134)	3·92 (3·27 – 4·69)	6989 (5418 – 8325)	3777 (2935 – 4698)	0·09 (0·07 – 0·1)	144 (109 – 174)	1680 (1174 – 2200)
Lesotho	28 (22 – 34)	2·42 (2·0 – 2·92)	14 410 (13 144 – 15 473)	974 (787 – 1203)	0·06 (0·05 – 0·07)	287 (255 – 315)	867 (692 – 1055)
Namibia	115 (93 – 139)	3·57 (3·01 – 4·26)	8217 (6723 – 9470)	2997 (2395 – 3697)	0·1 (0·09 – 0·13)	120 (79 – 155)	1673 (1168 – 2190)
South Africa	118 (98 – 140)	3·53 (2·99 – 4·17)	212 016 (181 378 – 239 044)	2060 (1513 – 2739)	0·15 (0·12 – 0·2)	1078 (-660 – 2294)	35 366 (24 782 – 46 588)
Swaziland (eSwatini)	80 (65 – 100)	2·93 (2·43 – 3·53)	6271 (5439 – 6994)	5141 (4059 – 6549)	0·04 (0·03 – 0·04)	171 (159 – 181)	1753 (1377 – 2181)
Zimbabwe	21 (17 – 25)	2·73 (2·28 – 3·31)	67 921 (59 244 – 75 421)	769 (611 – 951)	0·06 (0·05 – 0·08)	1431 (1214 – 1613)	3197 (2424 – 3924)
Western sub-Saharan Africa	..	3·49 (2·96 – 4·14)	1 314 010 (1 052 581 – 1 531 034)	..	0·06 (0·05 – 0·07)	37 925 (33 376 – 41 367)	59 195 (45 386 – 72 398)
Benin	7 (5 – 8)	3·71 (3·13 – 4·39)	32 265 (24 674 – 38 953)	400 (320 – 481)	0·05 (0·04 – 0·06)	1073 (949 – 1179)	809 (610 – 1006)
Burkina Faso	14 (12 – 17)	1·6 (1·32 – 1·89)	107 078 (99 758 – 113 840)	402 (325 – 499)	0·05 (0·04 – 0·06)	2156 (1938 – 2353)	3024 (2475 – 3607)
Cameroon	11 (9 – 14)	4·07 (3·32 – 5·14)	67 795 (42 948 – 85 051)	608 (489 – 735)	0·06 (0·05 – 0·07)	2145 (1835 – 2406)	2637 (1887 – 3388)
Cape Verde	24 (19 – 28)	6·16 (5·2 – 7·44)	465 (23 – 813)	1360 (1090 – 1638)	0·09 (0·07 – 0·1)	32 (23 – 38)	69 (41 – 94)
Chad	8 (7 – 10)	2·96 (2·47 – 3·79)	58 393 (44 688 – 67 045)	525 (412 – 659)	0·04 (0·03 – 0·05)	1769 (1601 – 1900)	1806 (1358 – 2249)
Cote d'Ivoire	14 (11 – 17)	3·92 (3·26 – 4·72)	65 217 (46 680 – 80 859)	699 (571 – 862)	0·06 (0·05 – 0·08)	1959 (1663 – 2225)	2893 (2089 – 3721)
The Gambia	7 (6 – 9)	5·31 (4·39 – 6·33)	2790 (1133 – 4250)	551 (438 – 669)	0·06 (0·05 – 0·07)	163 (135 – 184)	140 (96 – 185)
Ghana	18 (16 – 20)	4·79 (4·38 – 5·18)	53 317 (44 430 – 62 866)	1635 (1311 – 1954)	0·04 (0·04 – 0·05)	2674 (2465 – 2855)	6648 (5206 – 8094)
Guinea	5 (4 – 6)	3·45 (2·88 – 4·14)	43 783 (34 234 – 52 028)	282 (226 – 344)	0·05 (0·04 – 0·06)	1319 (1174 – 1442)	736 (561 – 908)
Guinea-Bissau	8 (7 – 10)	3·65 (3·05 – 4·35)	5952 (4596 – 7065)	460 (366 – 571)	0·06 (0·05 – 0·07)	176 (153 – 196)	166 (125 – 210)
Liberia	13 (10 – 16)	3·58 (2·99 – 4·36)	15 502 (11 817 – 18 396)	736 (591 – 907)	0·06 (0·05 – 0·07)	446 (388 – 493)	673 (502 – 848)
Mali	12 (10 – 14)	2·08 (1·84 – 2·33)	96 252 (89 929 – 102 622)	680 (593 – 771)	0·03 (0·03 – 0·03)	2571 (2465 – 2671)	3628 (3169 – 4085)
Mauritania	13 (10 – 16)	5·25 (4·36 – 6·39)	6893 (3621 – 9457)	645 (514 – 791)	0·08 (0·07 – 0·1)	231 (172 – 279)	305 (206 – 408)
Niger	6 (5 – 7)	2·41 (1·97 – 3·0)	97 816 (83 636 – 109 861)	268 (211 – 327)	0·04 (0·04 – 0·05)	2520 (2253 – 2718)	1556 (1204 – 1907)
Nigeria	19 (15 – 24)	3·54 (2·93 – 4·3)	595 624 (450 474 – 717 983)	830 (650 – 1023)	0·06 (0·05 – 0·08)	15 940 (13 191 – 18 072)	31 179 (22 410 – 40 156)
Sao Tome and Principe	17 (13 – 21)	4·94 (4·12 – 6·03)	315 (156 – 439)	793 (631 – 977)	0·09 (0·07 – 0·11)	11 (7 – 13)	18 (11 – 24)
Senegal	9 (8 – 11)	4·16 (3·51 – 4·94)	36 370 (25 259 – 45 456)	664 (531 – 811)	0·05 (0·04 – 0·06)	1383 (1233 – 1510)	1591 (1216 – 2007)
Sierra Leone	8 (6 – 9)	6·14 (5·1 – 7·57)	7602 (2229 – 11 947)	815 (639 – 1009)	0·05 (0·04 – 0·06)	722 (648 – 786)	811 (586 – 1046)
Togo	7 (6 – 8)	4·08 (3·43 – 4·93)	20 583 (14 857 – 25 208)	400 (325 – 493)	0·06 (0·05 – 0·07)	637 (539 – 714)	508 (370 – 649)

References

1 GBD 2016 SDG Collaborators. Measuring progress and projecting attainment on the basis of past trends of the health-related Sustainable Development Goals in 188 countries: an analysis from the Global Burden of Disease Study 2016. *Lancet Lond Engl* 2017; **390**: 1423–59.

2 United States Census Bureau. Computer Assisted Personal Interviewing manual for National Health Interview Survey field representatives. 2018; published online Jan. ftp://ftp.cdc.gov/pub/Health_Statistics/NCHS/Survey_Questionnaires/NHIS/2018/frmanual.pdf.

3 Lavado RF, Brooks BPC, Hanlon M. Estimating health expenditure shares from household surveys. *World Health Organ Bull World Health Organ Geneva* 2013; **91**: 519-24C.

4 Ngandu NK, Manda S, Besada D, Rohde S, Oliphant NP, Doherty T. Does adjusting for recall in trend analysis affect coverage estimates for maternal and child health indicators? An analysis of DHS and MICS survey data. *Glob Health Action* 2016; **9**: 32408.

5 Lu C, Chin B, Li G, Murray CJ. Limitations of methods for measuring out-of-pocket and catastrophic private health expenditures. *Bull World Health Organ* 2009; **87**: 238–44.

6 Flaxman AD, Vos T, Murray CJL, Kiyono P, editors. An Integrative Metaregression Framework for Descriptive Epidemiology, 1 edition. Seattle: University of Washington Press, 2015.

7 GBD 2016 Disease and Injury Incidence and Prevalence Collaborators. Global, regional, and national incidence, prevalence, and years lived with disability for 328 diseases and injuries for 195 countries, 1990-2016: a systematic analysis for the Global Burden of Disease Study 2016. *Lancet Lond Engl* 2017; **390**: 1211–59.

8 Getzen TE. Health care is an individual necessity and a national luxury: applying multilevel decision models to the analysis of health care expenditures. *J Health Econ* 2000; **19**: 259–70.

9 Fletcher JM, Frisvold DE. Higher Education and Health Investments: Does More Schooling Affect Preventive Health Care Use? *J Hum Cap* 2009; **3**: 144–76.

10 GBD 2015 Mortality and Causes of Death Collaborators. Global, regional, and national life expectancy, all-cause mortality, and cause-specific mortality for 249 causes of death, 1980-2015: a systematic analysis for the Global Burden of Disease Study 2015. *Lancet Lond Engl* 2016; **388**: 1459–544.

11 GBD 2016 Mortality Collaborators. Global, regional, and national under-5 mortality, adult mortality, age-specific mortality, and life expectancy, 1970-2016: a systematic analysis for the Global Burden of Disease Study 2016. *Lancet Lond Engl* 2017; **390**: 1084–150.

12 World Development Indicators | The World Bank. http://wdi.worldbank.org/table/2.12 (accessed May 5, 2017).

13 GBD 2016 Risk Factors Collaborators. Global, regional, and national comparative risk assessment of 84 behavioural, environmental and occupational, and metabolic risks or clusters of risks, 1990-2016: a systematic analysis for the Global Burden of Disease Study 2016. *Lancet Lond Engl* 2017; **390**: 1345–422.

14 GBD 2013 Mortality and Causes of Death Collaborators. Global, regional, and national age-sex specific all-cause and cause-specific mortality for 240 causes of death, 1990-2013: a systematic analysis for the Global Burden of Disease Study 2013. *Lancet Lond Engl* 2015; **385**: 117–71.

15 OECD, WHO, Eurostat. A System of Health Accounts. Paris: Organisation for Economic Co-operation and Development, 2011 http://www.oecd-ilibrary.org/content/book/9789264116016-en (accessed April 29, 2017).

16 Bui AL, Lavado RF, Johnson EK, *et al.* National health accounts data from 1996 to 2010: a systematic review. *Bull World Health Organ* 2015; **93**: 566-576D.

17 Dieleman J, Campbell M, Chapin A, *et al.* Evolution and patterns of global health financing 1995–2014: development assistance for health, and government, prepaid private, and out-of-pocket health spending in 184 countries. *The Lancet* 2017; **389**: 1981–2004.

18 Dieleman JL, Haakenstad A, Micah A, *et al.* Spending on health and HIV/AIDS: domestic health spending and development assistance in 188 countries, 1995–2015. *The Lancet* 2018; **391**: 1799–829.

19 Dieleman JL, Sadat N, Chang AY, *et al.* Trends in future health financing and coverage: future health spending and universal health coverage in 188 countries, 2016–40. *The Lancet* 2018; **391**: 1783–98.

20 Stenberg K, Lauer JA, Gkountouras G, Fitzpatrick C, Stanciole A. Econometric estimation of WHO-CHOICE country-specific costs for inpatient and outpatient health service delivery. *Cost Eff Resour Alloc* 2018; **16**: 11.

21 WHO | Health service delivery costs. WHO. http://www.who.int/choice/cost-effectiveness/inputs/health_service/en/ (accessed July 15, 2018).

22 World Economic Outlook Database April 2017. https://www.imf.org/external/pubs/ft/weo/2017/01/weodata/index.aspx (accessed Sept 6, 2017).

23 Reference costs | NHS Improvement. https://improvement.nhs.uk/resources/reference-costs/ (accessed July 26, 2018).

24 Understanding Variability in the Cost of a Standard Hospital Stay. 2017 https://apps.uqo.ca/LoginSigparb/LoginPourRessources.aspx?url=http://www.deslibris.ca/ID/10096045 (accessed July 17, 2018).

25 Dieleman J, Financing Global Health 2017 Collaborator network. Tracking spending on health and HIV/AIDS: 188 countries, 1995-2015. *Forthcom Lancet*.

26 Dieleman J, Financing Global Health 2017 Collaborator network. Estimating future health spending and universal health coverage, 2016-2040. *Forthcom Lancet.*

27 GBD 2016 Healthcare Access and Quality Collaborators. Measuring performance on the Healthcare Access and Quality Index for 195 countries and territories and selected subnational locations: a systematic analysis from the Global Burden of Disease Study 2016. *Lancet Lond Engl* 2018; **391**: 2236–71.

28 Barber RM, Fullman N, Sorensen RJD, *et al.* Healthcare Access and Quality Index based on mortality from causes amenable to personal health care in 195 countries and territories, 1990–2015: a novel analysis from the Global Burden of Disease Study 2015. *The Lancet* 2017; **0**. DOI:10.1016/S0140-6736(17)30818-8.

Table S5. Data sources used in the utilisation estimates and their attributes by country and years

Table S5: Data sources used in the utilisation estimates and their attributes by country and years									
**Location**	**Year**	**Citation**	**Data source used in outpatient model**	**Outpatient recall period in weeks**	**Data source used in inpatient model**	**Inpatient recall period in weeks**	**Data source used as reference data**	**Age range**	**Sexes**
Afghanistan	2006	Indian Institute of Health Management Research (IIHMR), Johns Hopkins University (JHU), Ministry of Public Health (Afghanistan). Afghanistan Health Survey 2006.	X	4.3				All ages	Both
Afghanistan	2010	Central Statistics Organization (Afghanistan), ICF Macro, Indian Institute of Health Management Research (IIHMR), Ministry of Public Health (Afghanistan), World Health Organization Regional Office for the Eastern Mediterranean (EMRO-WHO). Afghanistan Special Demographic and Health Survey 2010. Calverton, United States: ICF Macro.	X	4.3	X	52		All ages	Both
Albania	1993	Ministry of Health (Albania). Albania Inpatient Care Discharges per 100 1993.			X		X	All ages	Both
Albania	1994	Ministry of Health (Albania). Albania Inpatient Care Discharges per 100 1994.			X		X	All ages	Both
Albania	1995	Ministry of Health (Albania). Albania Inpatient Care Discharges per 100 1995.			X		X	All ages	Both
Albania	1996	Ministry of Health (Albania). Albania Inpatient Care Discharges per 100 1996.			X		X	All ages	Both
Albania	1997	Ministry of Health (Albania). Albania Inpatient Care Discharges per 100 1997.			X		X	All ages	Both
Albania	1998	Ministry of Health (Albania). Albania Inpatient Care Discharges per 100 1998.			X		X	All ages	Both
Albania	1999	Ministry of Health (Albania). Albania Inpatient Care Discharges per 100 1999.			X		X	All ages	Both
Albania	2000	Ministry of Health (Albania). Albania Inpatient Care Discharges per 100 2000.			X		X	All ages	Both
Albania	2001	Ministry of Health (Albania). Albania Inpatient Care Discharges per 100 2001.			X		X	All ages	Both
Albania	2002	National Institute of Statistics (Albania), World Bank (WB). Albania Living Standards Measurement Survey 2002. Washington DC, United States: World Bank (WB).	X	4	X	52		All ages	Both
Albania	2002	Ministry of Health (Albania). Albania Inpatient Care Discharges per 100 2002.			X		X	All ages	Both
Albania	2003	Ministry of Health (Albania). Albania Inpatient Care Discharges per 100 2003.			X		X	All ages	Both
Albania	2004	National Institute of Statistics (Albania), World Bank (WB). Albania Living Standards Measurement Survey 2004. Washington DC, United States: World Bank (WB).	X	4				All ages	Both
Albania	2004	Ministry of Health (Albania). Albania Inpatient Care Discharges per 100 2004.			X		X	All ages	Both
Albania	2005	National Institute of Statistics (Albania), World Bank (WB). Albania Living Standards Measurement Survey 2005. Washington DC, United States: World Bank (WB).			X	52		All ages	Both
Albania	2005	Ministry of Health (Albania). Albania Inpatient Care Discharges per 100 2005.			X		X	All ages	Both
Albania	2006	Ministry of Health (Albania). Albania Inpatient Care Discharges per 100 2006.			X		X	All ages	Both
Albania	2007	Ministry of Health (Albania). Albania Inpatient Care Discharges per 100 2007.			X		X	All ages	Both
Albania	2008	Ministry of Health (Albania). Albania Inpatient Care Discharges per 100 2008.			X		X	All ages	Both
Albania	2009	Ministry of Health (Albania). Albania Inpatient Care Discharges per 100 2009.			X		X	All ages	Both
Albania	2010	Ministry of Health (Albania). Albania Inpatient Care Discharges per 100 2010.			X		X	All ages	Both
Albania	2011	Ministry of Health (Albania). Albania Inpatient Care Discharges per 100 2011.			X		X	All ages	Both
Albania	2012	Ministry of Health (Albania). Albania Inpatient Care Discharges per 100 2012.			X		X	All ages	Both
Albania	2013	Ministry of Health (Albania). Albania Inpatient Care Discharges per 100 2013.			X		X	All ages	Both
Algeria	2005	National Institute of Public Health, Ministry of Health, Population, and Hospital Reform (Algeria). Algeria National Health Survey 2005.	X	12.9				All ages	Both
Angola	2008	National Institute of Statistics (Angola), Oxford Policy Management, United Nations Children's Fund (UNICEF). Angola Integrated Inquiry into People's Well-Being 2008-2009.	X	4.3				All ages	Both
Argentina	2000	Gallup Europe, World Health Organization (WHO). Argentina WHO Multi-country Survey Study on Health and Health System Responsiveness 2000-2001. Geneva, Switzerland: World Health Organization (WHO).	X	4.3	X	4.3		15-95	Both
Argentina	2005	Center for the Study of State and Society (CEDES), Ministry of Health and Environment (Argentina), National Institute of Statistics and Censuses (Argentina). Argentina National Survey of Risk Factors 2005.	X	52				15-100+	Male
Argentina	2009	Ministry of Health (Argentina), National Institute of Statistics and Censuses (Argentina). Argentina National Survey of Risk Factors 2009.	X	52				15-100+	Both
Armenia	1980	Ministry of Health (Armenia). Armenia Inpatient Care Discharges per 100 1980.			X		X	All ages	Both
Armenia	1981	Ministry of Health (Armenia). Armenia Inpatient Care Discharges per 100 1981.			X		X	All ages	Both
Armenia	1982	Ministry of Health (Armenia). Armenia Inpatient Care Discharges per 100 1982.			X		X	All ages	Both

Armenia	1983	Ministry of Health (Armenia). Armenia Inpatient Care Discharges per 100 1983.			X		X	All ages	Both
Armenia	1984	Ministry of Health (Armenia). Armenia Inpatient Care Discharges per 100 1984.			X		X	All ages	Both
Armenia	1985	Ministry of Health (Armenia). Armenia Inpatient Care Discharges per 100 1985.			X		X	All ages	Both
Armenia	1986	Ministry of Health (Armenia). Armenia Inpatient Care Discharges per 100 1986.			X		X	All ages	Both
Armenia	1987	Ministry of Health (Armenia). Armenia Inpatient Care Discharges per 100 1987.			X		X	All ages	Both
Armenia	1988	Ministry of Health (Armenia). Armenia Inpatient Care Discharges per 100 1988.			X		X	All ages	Both
Armenia	1989	Ministry of Health (Armenia). Armenia Inpatient Care Discharges per 100 1989.			X		X	All ages	Both
Armenia	1990	Ministry of Health (Armenia). Armenia Inpatient Care Discharges per 100 1990.			X		X	All ages	Both
Armenia	1991	Ministry of Health (Armenia). Armenia Inpatient Care Discharges per 100 1991.			X		X	All ages	Both
Armenia	1992	Ministry of Health (Armenia). Armenia Inpatient Care Discharges per 100 1992.			X		X	All ages	Both
Armenia	1993	Ministry of Health (Armenia). Armenia Inpatient Care Discharges per 100 1993.			X		X	All ages	Both
Armenia	1994	Ministry of Health (Armenia). Armenia Inpatient Care Discharges per 100 1994.			X		X	All ages	Both
Armenia	1995	Ministry of Health (Armenia). Armenia Inpatient Care Discharges per 100 1995.			X		X	All ages	Both
Armenia	1996	Ministry of Health (Armenia). Armenia Inpatient Care Discharges per 100 1996.			X		X	All ages	Both
Armenia	1997	Ministry of Health (Armenia). Armenia Inpatient Care Discharges per 100 1997.			X		X	All ages	Both
Armenia	1998	Ministry of Health (Armenia). Armenia Inpatient Care Discharges per 100 1998.			X		X	All ages	Both
Armenia	1998	Ministry of Health (Armenia). Armenia Outpatient Contacts Per Person Per Year 1998.	X				X	All ages	Both
Armenia	1999	Ministry of Health (Armenia). Armenia Inpatient Care Discharges per 100 1999.			X		X	All ages	Both
Armenia	1999	Ministry of Health (Armenia). Armenia Outpatient Contacts Per Person Per Year 1999.	X				X	All ages	Both
Armenia	2000	Ministry of Health (Armenia). Armenia Inpatient Care Discharges per 100 2000.			X		X	All ages	Both
Armenia	2000	Ministry of Health (Armenia). Armenia Outpatient Contacts Per Person Per Year 2000.	X				X	All ages	Both
Armenia	2001	Ministry of Health (Armenia). Armenia Inpatient Care Discharges per 100 2001.			X		X	All ages	Both
Armenia	2001	Ministry of Health (Armenia). Armenia Outpatient Contacts Per Person Per Year 2001.	X				X	All ages	Both
Armenia	2002	Ministry of Health (Armenia). Armenia Inpatient Care Discharges per 100 2002.			X		X	All ages	Both
Armenia	2002	Ministry of Health (Armenia). Armenia Outpatient Contacts Per Person Per Year 2002.	X				X	All ages	Both
Armenia	2003	Ministry of Health (Armenia). Armenia Inpatient Care Discharges per 100 2003.			X		X	All ages	Both
Armenia	2003	Ministry of Health (Armenia). Armenia Outpatient Contacts Per Person Per Year 2003.	X				X	All ages	Both
Armenia	2004	Ministry of Health (Armenia). Armenia Inpatient Care Discharges per 100 2004.			X		X	All ages	Both
Armenia	2004	Ministry of Health (Armenia). Armenia Outpatient Contacts Per Person Per Year 2004.	X				X	All ages	Both
Armenia	2005	Ministry of Health (Armenia). Armenia Inpatient Care Discharges per 100 2005.			X		X	All ages	Both
Armenia	2005	Ministry of Health (Armenia). Armenia Outpatient Contacts Per Person Per Year 2005.	X				X	All ages	Both
Armenia	2006	Ministry of Health (Armenia). Armenia Inpatient Care Discharges per 100 2006.			X		X	All ages	Both
Armenia	2006	Ministry of Health (Armenia). Armenia Outpatient Contacts Per Person Per Year 2006.	X				X	All ages	Both
Armenia	2007	Ministry of Health (Armenia). Armenia Inpatient Care Discharges per 100 2007.			X		X	All ages	Both
Armenia	2007	Ministry of Health (Armenia). Armenia Outpatient Contacts Per Person Per Year 2007.	X				X	All ages	Both
Armenia	2008	Ministry of Health (Armenia). Armenia Inpatient Care Discharges per 100 2008.			X		X	All ages	Both
Armenia	2008	Ministry of Health (Armenia). Armenia Outpatient Contacts Per Person Per Year 2008.	X				X	All ages	Both
Armenia	2009	Ministry of Health (Armenia). Armenia Inpatient Care Discharges per 100 2009.			X		X	All ages	Both
Armenia	2009	Ministry of Health (Armenia). Armenia Outpatient Contacts Per Person Per Year 2009.	X				X	All ages	Both
Armenia	2010	Armenian Sociological Association, Concluzia-Prim Center for Survey Methodology (Moldova), Institute for Advanced Studies (Austria), London School of Hygiene and Tropical Medicine, University of Aberdeen. Armenia Health in Times of Transition Household Survey 2010.			X	52		15-85	Both
Armenia	2010	Ministry of Health (Armenia). Armenia Inpatient Care Discharges per 100 2010.			X		X	All ages	Both
Armenia	2010	Ministry of Health (Armenia). Armenia Outpatient Contacts Per Person Per Year 2010.	X				X	All ages	Both

Armenia	2011	Ministry of Health (Armenia). Armenia Inpatient Care Discharges per 100 2011.			X		X	All ages	Both
Armenia	2011	Ministry of Health (Armenia). Armenia Outpatient Contacts Per Person Per Year 2011.	X				X	All ages	Both
Armenia	2012	Ministry of Health (Armenia). Armenia Inpatient Care Discharges per 100 2012.			X		X	All ages	Both
Armenia	2012	Ministry of Health (Armenia). Armenia Outpatient Contacts Per Person Per Year 2012.	X				X	All ages	Both
Armenia	2013	Ministry of Health (Armenia). Armenia Inpatient Care Discharges per 100 2013.			X		X	All ages	Both
Armenia	2013	Ministry of Health (Armenia). Armenia Outpatient Contacts Per Person Per Year 2013.	X				X	All ages	Both
Armenia	2014	Ministry of Health (Armenia). Armenia Inpatient Care Discharges per 100 2014.			X		X	All ages	Both
Armenia	2014	Ministry of Health (Armenia). Armenia Outpatient Contacts Per Person Per Year 2014.	X				X	All ages	Both
Australia	1995	Australian Bureau of Statistics. Australia National Health Survey 1995. Canberra, Australia: Australian Bureau of Statistics.	X	2	X	2		All ages	Both
Australia	2000	TQA Research, World Health Organization (WHO). Australia WHO Multi-country Survey Study on Health and Health System Responsiveness 2000-2001. Geneva, Switzerland: World Health Organization (WHO).	X	4.3	X	4.3		15-100+	Both
Australia	2001, 2002, 2003, 2004, 2005, 2006, 2007, 2008, 2009, 2010, 2011, 2012, 2013, 2014, 2015, 2016	Organization for Economic Co-operation and Development (OECD). OECD Health Statistics. Paris, France: Organization for Economic Co-operation and Development (OECD).	X				X	All ages	Both
Australia	2005	Centre for Health Promotion Studies, National University of Ireland, Galway, Health Promotion Unit, Department of Health and Children (Ireland). Ireland Survey of Lifestyle Attitudes and Nutrition 1998. Dublin, Ireland: Health Promotion Unit, Department of Health and Children (Ireland).	X	2	X	2		All ages	Both
Australia	2011	ISSP Research Group (2009): International Social Survey Programme: Health and Health Care - ISSP 2011. GESIS Data Archive, Cologne. ZA5800 Data file version 3.0.0, doi:10.4232/1.12252.	X	52				All ages	Both
Austria	1989, 1990, 1991, 1992, 1993, 1994, 1995, 1996, 1997, 1998, 1999, 2001, 2002, 2003, 2004, 2005, 2006, 2007, 2009, 2010, 2011	World Health Organization Regional Office for Europe (WHO/Europe). European Hospital Morbidity Database 1999-2007. Copenhagen, Denmark: World Health Organization Regional Office for Europe (WHO/Europe).			X		X	All ages	Both
Austria	1989	Federal Ministry of Health (Austria), Statistics Austria. Austria Hospital Inpatient Discharges 1989. Vienna, Austria: Statistics Austria.			X		X	All ages	Both
Austria	1999		X				X	All ages	Both
Austria	2000	Statistics Austria, World Health Organization (WHO). Austria WHO Multi-country Survey Study on Health and Health System Responsiveness 2000-2001. Geneva, Switzerland: World Health Organization (WHO).	X	4.3	X	4.3		All ages	Both
Austria	2000		X				X	All ages	Both
Austria	2001, 2002, 2003, 2004, 2005, 2006, 2007, 2008, 2009, 2010, 2011, 2012, 2013,	Organization for Economic Co-operation and Development (OECD). OECD Health Statistics. Paris, France: Organization for Economic Co-operation and Development (OECD).	X				X	All ages	Both

	2014, 2015								
Austria	2003	World Health Organization (WHO). Austria World Health Survey 2003. Geneva, Switzerland: World Health Organization (WHO), 2005.			X	52		0-95	Both
Austria	2006	Börsch-Supan, A. (2013). Survey of Health, Ageing and Retirement in Europe (SHARE) Wave 1. Release version: 2.6.0. SHARE-ERIC. Data set. DOI: 10.6103/SHARE.w1.260	X	52	X	52		All ages	Both
Austria	2010	Börsch-Supan, A. (2013). Survey of Health, Ageing and Retirement in Europe (SHARE) Wave 2. Release version: 2.6.0. SHARE-ERIC. Data set. DOI: 10.6103/SHARE.w2.260	X	52	X	52		35-100+	Both
Austria	2012	Börsch-Supan, A. (2013). Survey of Health, Ageing and Retirement in Europe (SHARE) Wave 4. Release version: 1.1.1. SHARE-ERIC. Data set. DOI: 10.6103/SHARE.w4.111	X	52	X	52		35-100+	Both
Austria	2013	Federal Ministry of Health (Austria), Statistics Austria. Austria Hospital Inpatient Discharges 2013. Vienna, Austria: Statistics Austria.			X		X	All ages	Both
Austria	2013	Börsch-Supan, A. (2015). Survey of Health, Ageing and Retirement in Europe (SHARE) Wave 5. Release version: 1.0.0. SHARE-ERIC. Data set. DOI: 10.6103/SHARE.w5.100	X	52	X	52		All ages	Both
Austria	2014	Federal Ministry of Health (Austria), Statistics Austria. Austria Hospital Inpatient Discharges 2014. Vienna, Austria: Statistics Austria.			X		X	All ages	Both
Azerbaijan	1981, 1986, 1987, 1988, 1989, 1990, 1991, 1992, 1993, 1994, 1995, 1997, 1998, 1999, 2000, 2001, 2002, 2003, 2004, 2005, 2006, 2007, 2008, 2009, 2010, 2011, 2012, 2013, 2014, 2015, 2016	World Health Organization Regional Office for Europe (WHO/Europe). European Health for All Database - Inpatient Care Discharges Per 100. Copenhagen, Denmark: World Health Organization Regional Office for Europe (WHO/Europe).			X		X	All ages	Both
Azerbaijan	1999, 2000, 2001, 2002, 2003, 2004, 2005, 2006, 2007, 2008, 2009, 2010, 2011, 2012, 2013, 2014	World Health Organization Regional Office for Europe (WHO/Europe). European Health for All Database - Outpatient Contacts Per Person Per Year. Copenhagen, Denmark: World Health Organization Regional Office for Europe (WHO/Europe).	X				X	All ages	Both
Azerbaijan	2010	Concluzia-Prim Center for Survey Methodology (Moldova), Institute for Advanced Studies (Austria), London School of Hygiene and Tropical Medicine, SIAR Research and Consulting (Azerbaijan), University of Aberdeen. Azerbaijan Health in Times of Transition Household Survey 2010.			X	52		15-85	Both
Bahrain	1995	Ministry of Health (Bahrain), Council of Health Ministers of GCC States. Bahrain Family Health Survey 1995. Manama, Bahrain: Ministry of Health (Bahrain).	X	2				All ages	Both
Bahrain	2000	Gallup Europe, World Health Organization (WHO). Bahrain WHO Multi-country Survey Study on Health and Health System Responsiveness 2000-2001. Geneva, Switzerland: World Health Organization (WHO).	X	4.3	X	4.3		15-95	Both
Bangladesh	2003	World Health Organization (WHO). Bangladesh World Health Survey 2003. Geneva, Switzerland: World Health Organization (WHO), 2005.			X	52		All ages	Both

Barbados	1999	Pan American Health Organization (PAHO), Center for Demography and Ecology, University of Wisconsin-Madison, Inter-University Consortium for Political and Social Research (ICPSR), Chronic Disease Research Centre (CDRC), University of the West Indies. Barbados - Bridgetown Survey on Health, Well-Being, and Aging in Latin America and the Caribbean 1999-2000. Ann Arbor, United States: Inter-University Consortium for Political and Social Research (ICPSR).	X	17.2	X	17.2		60-100+	Both
Belarus	1999, 2000, 2001, 2002, 2003, 2004, 2005, 2006, 2007, 2008, 2009, 2010, 2011, 2012, 2013, 2014, 2015	World Health Organization Regional Office for Europe (WHO/Europe). European Health for All Database - Outpatient Contacts Per Person Per Year. Copenhagen, Denmark: World Health Organization Regional Office for Europe (WHO/Europe).	X				X	All ages	Both
Belarus	2010	Belarusian State University, Concluzia-Prim Center for Survey Methodology (Moldova), Institute for Advanced Studies (Austria), London School of Hygiene and Tropical Medicine, University of Aberdeen. Belarus Health in Times of Transition Household Survey 2010.			X	52		15-90	Both
Belgium	1998	National Institute for Health and Disability Insurance (Belgium). Belgium Outpatient Contacts Per Person Per Year 1998.	X				X	All ages	Both
Belgium	1999	National Institute for Health and Disability Insurance (Belgium). Belgium Outpatient Contacts Per Person Per Year 1999.	X				X	All ages	Both
Belgium	2000	International Research Associates (INRA) Europe, World Health Organization (WHO). Belgium WHO Multi-country Survey Study on Health and Health System Responsiveness 2000-2001. Geneva, Switzerland: World Health Organization (WHO).	X	4.3	X	4.3		15-95	Both
Belgium	2000	Federal Public Service Health, Food Chain Safety, and Environment (Belgium). Belgium Minimum Clinical Summary 2000.			X		X	All ages	Both
Belgium	2001, 2002, 2003, 2004, 2005, 2006, 2007, 2008, 2009, 2010, 2011, 2012	Organization for Economic Co-operation and Development (OECD). OECD Health Statistics. Paris, France: Organization for Economic Co-operation and Development (OECD).	X				X	All ages	Both
Belgium	2001	Federal Public Service Health, Food Chain Safety, and Environment (Belgium). Belgium Minimum Clinical Summary 2001.			X		X	All ages	Both
Belgium	2002, 2003, 2007	World Health Organization Regional Office for Europe (WHO/Europe). European Hospital Morbidity Database 1999-2007. Copenhagen, Denmark: World Health Organization Regional Office for Europe (WHO/Europe).			X		X	All ages	Both
Belgium	2002	World Health Organization (WHO). Belgium World Health Survey 2002. Geneva, Switzerland: World Health Organization (WHO), 2005.			X	52		All ages	Both
Belgium	2002	Federal Public Service Health, Food Chain Safety, and Environment (Belgium). Belgium Minimum Clinical Summary 2002.			X		X	All ages	Both
Belgium	2005	Federal Public Service Health, Food Chain Safety, and Environment (Belgium). Belgium Minimum Clinical Summary 2005.			X		X	All ages	Both
Belgium	2006	Börsch-Supan, A. (2013). Survey of Health, Ageing and Retirement in Europe (SHARE) Wave 1. Release version: 2.6.0. SHARE-ERIC. Data set. DOI: 10.6103/SHARE.w1.260	X	52	X	52		All ages	Both
Belgium	2006	Federal Public Service Health, Food Chain Safety, and Environment (Belgium). Belgium Minimum Clinical Summary 2006.			X		X	All ages	Both
Belgium	2007	Federal Public Service Health, Food Chain Safety, and Environment (Belgium). Belgium Minimum Clinical Summary 2007.			X		X	All ages	Both
Belgium	2010	Börsch-Supan, A. (2013). Survey of Health, Ageing and Retirement in Europe (SHARE) Wave 2. Release version: 2.6.0. SHARE-ERIC. Data set. DOI: 10.6103/SHARE.w2.260	X	52	X	52		30-100+	Both
Belgium	2010	Federal Public Service Health, Food Chain Safety, and Environment (Belgium). Belgium Minimum Hospital Summary 2010.			X		X	All ages	Both
Belgium	2011	ISSP Research Group (2009): International Social Survey Programme: Health and Health Care - ISSP 2011. GESIS Data Archive, Cologne. ZA5800 Data file version 3.0.0, doi:10.4232/1.12252.	X	52				0-95	Both
Belgium	2011	Federal Public Service Health, Food Chain Safety, and Environment (Belgium). Belgium Minimum Hospital Summary 2011.			X		X	All ages	Both

Belgium	2012	Börsch-Supan, A. (2013). Survey of Health, Ageing and Retirement in Europe (SHARE) Wave 4. Release version: 1.1.1. SHARE-ERIC. Data set. DOI: 10.6103/SHARE.w4.111	X	52	X	52		40-100+	Both
Belgium	2012	Federal Public Service Health, Food Chain Safety, and Environment (Belgium). Belgium Minimum Hospital Summary 2012.			X		X	All ages	Both
Belgium	2013	Börsch-Supan, A. (2015). Survey of Health, Ageing and Retirement in Europe (SHARE) Wave 5. Release version: 1.0.0. SHARE-ERIC. Data set. DOI: 10.6103/SHARE.w5.100	X	52	X	52		All ages	Both
Belgium	2013	Federal Public Service Health, Food Chain Safety, and Environment (Belgium). Belgium Minimum Hospital Summary 2013.			X		X	All ages	Both
Bhutan	2003	National Statistics Bureau (Bhutan). Bhutan Living Standards Survey 2003.	X	4				All ages	Both
Bolivia	1992	National Institute of Statistics (Bolivia). Bolivia Integrated Household Survey 1992. La Paz, Bolivia: National Institute of Statistics (Bolivia).	X	4.3				All ages	Both
Bolivia	1994	National Institute of Statistics (Bolivia). Bolivia Integrated Household Survey 1994. La Paz, Bolivia: National Institute of Statistics (Bolivia).	X	4.3				All ages	Both
Bolivia	1999	National Institute of Statistics (Bolivia), World Bank (WB), Inter-American Development Bank (IDB), United Nations Economic Commission for Latin America and the Caribbean (CEPAL). Bolivia Household Survey 1999. La Paz, Bolivia: National Institute of Statistics (Bolivia).	X	4				All ages	Both
Bolivia	2001	National Institute of Statistics (Bolivia), World Bank (WB), Inter-American Development Bank (IDB), United Nations Economic Commission for Latin America and the Caribbean (CEPAL). Bolivia Household Survey 2001. La Paz, Bolivia: National Institute of Statistics (Bolivia).	X	4				All ages	Both
Bolivia	2002	National Institute of Statistics (Bolivia), World Bank (WB). Bolivia Household Survey 2002. La Paz, Bolivia: National Institute of Statistics (Bolivia).	X	4				All ages	Both
Bolivia	2003	National Institute of Statistics (Bolivia). Bolivia Household Survey 2003-2004. La Paz, Bolivia: National Institute of Statistics (Bolivia).	X	4				All ages	Both
Bolivia	2009	National Institute of Statistics (Bolivia). Bolivia Household Survey 2009. La Paz, Bolivia: National Institute of Statistics (Bolivia).	X	4				All ages	Both
Bosnia and Herzegovina	2001	Agency for Statistics (Bosnia and Herzegovina), Institute of Statistics (Republic of Srpska), Federal Office of Statistics (Bosnia and Herzegovina), Swedish International Development Agency (SIDA), UK Department for International Development (DFID), United Nations Development Programme (UNDP), European Commission (EC), Government of Japan, World Bank (WB). Bosnia and Herzegovina Living Standards Measurement Survey 2001. Washington, DC, United States: World Bank (WB).	X	4	X	52		All ages	Both
Bosnia and Herzegovina	2002	Agency for Statistics (Bosnia and Herzegovina), Institute of Statistics (Republic of Srpska), Federal Office of Statistics (Bosnia and Herzegovina), Independent Bureau for Humanitarian Issues (IBHI), Birks Sinclair and Associates, LTD, Institute for Social and Economic Research, University of Essex. Bosnia and Herzegovina Living Standards Measurement Survey 2002. Washington, DC, United States: World Bank (WB).	X	52	X	52		1-100+	Both
Bosnia and Herzegovina	2003	World Health Organization (WHO). Bosnia and Herzegovina World Health Survey 2003. Geneva, Switzerland: World Health Organization (WHO), 2005.			X	52		15-85	Both
Bosnia and Herzegovina	2004	Agency for Statistics (Bosnia and Herzegovina), Birks Sinclair and Associates, LTD, Federal Office of Statistics (Federation of Bosnia and Herzegovina), Independent Bureau for Humanitarian Issues (IBHI), Institute for Social and Economic Research, University of Essex, Institute of Statistics (Republic of Srpska). Bosnia and Herzegovina Living Standards Measurement Survey 2004-2005.	X	52				15-100+	Both
Brazil	1996	Brazilian Institute of Geography and Statistics (IBGE), World Bank (WB). Brazil Living Standards Measurement Survey 1996-1997. Washington DC, United States: World Bank (WB).	X	4.3				All ages	Both
Brazil	2001, 2002, 2003, 2004, 2005, 2006, 2007, 2008, 2009, 2010, 2011, 2012, 2013, 2014	Organization for Economic Co-operation and Development (OECD). OECD Health Statistics. Paris, France: Organization for Economic Co-operation and Development (OECD).	X				X	All ages	Both
Brazil	2003	Center for Scientific and Technological Information, Oswaldo Cruz Foundation and World Health Organization (WHO). Brazil World Health Survey 2003. Geneva, Switzerland: World Health Organization (WHO), 2005.			X	52		15-100+	Both
Brazil	2003, 2008	Health Institute (São Paulo, Brazil), State University of Campinas, São Paulo Municipal Health Department, São Paulo State University, University of São Paulo. Brazil - São Paulo Health Survey 2008-2009.			X	52		All ages	Both

Brazil	2013	Brazilian Institute of Geography and Statistics (IBGE), Ministry of Health (Brazil), Ministry of Planning, Budget, and Management (Brazil). Brazil National Health Survey 2013. Rio de Janeiro, Brazil: Brazilian Institute of Geography and Statistics (IBGE).	X	52	X	52		All ages	Both
Brunei	2001	Department of Economic Planning and Development (Brunei Darussalam). Brunei Population and Housing Census 2001.	X	4.3				All ages	Both
Bulgaria	1981, 1982, 1983, 1984, 1985, 1986, 1987, 1988, 1989, 1990, 1991, 1992, 1993, 1994, 1995, 1996, 1997, 1998, 1999, 2000, 2001, 2002, 2003, 2004, 2005, 2006, 2007, 2008, 2009, 2010, 2011, 2012, 2013, 2014, 2015	World Health Organization Regional Office for Europe (WHO/Europe). European Health for All Database - Inpatient Care Discharges Per 100. Copenhagen, Denmark: World Health Organization Regional Office for Europe (WHO/Europe).			X		X	All ages	Both
Bulgaria	1995	Gallup International, World Bank (WB). Bulgaria Living Standards Measurement Survey 1995. Washington, DC, United States: World Bank (WB).	X	4				All ages	Both
Bulgaria	1997	Gallup International, World Bank (WB). Bulgaria Living Standards Measurement Survey 1997. Washington, DC, United States: World Bank (WB).	X	4				All ages	Both
Bulgaria	2000	International Research Associates (INRA) Europe, World Health Organization (WHO). Bulgaria WHO Multi-country Survey Study on Health and Health System Responsiveness 2000-2001. Geneva, Switzerland: World Health Organization (WHO).	X	4.3	X	4.3		15-90	Both
Bulgaria	2001	TNS BBSS, World Bank. Bulgaria Living Standards Measurement Survey 2001. Washington DC, United States: World Bank.	X	4	X	4		All ages	Both
Bulgaria	2003	National Statistical Institute of Bulgaria. Bulgaria Living Standards Measurement Survey 2003. Washington DC, United States: World Bank.	X	52	X	52		All ages	Both
Bulgaria	2007	Ministry of Labor and Social Policy (Bulgaria), National Statistical Institute of Bulgaria, TNS Gallup, World Bank. Bulgaria Multitopic Household Survey 2007. Washington DC, United States: World Bank.	X	52	X	52		All ages	Both
Bulgaria	2011	ISSP Research Group (2009): International Social Survey Programme: Health and Health Care - ISSP 2011. GESIS Data Archive, Cologne. ZA5800 Data file version 3.0.0, doi:10.4232/1.12252.	X	52				15-95	Both
Burkina Faso	2002	World Health Organization (WHO). Burkina Faso World Health Survey 2002-2003. Geneva, Switzerland: World Health Organization (WHO), 2005.			X	52		All ages	Both
Burkina Faso	2003	National Institute of Statistics and Demography (Burkina Faso), World Bank. Burkina Faso Core Welfare Indicators Questionnaire Survey 2003. Ouagadougou, Burkina Faso: National Institute of Statistics and Demography (Burkina Faso).	X	4				All ages	Both
Burkina Faso	2014	National Institute of Statistics and Demography (Burkina Faso), World Bank. Burkina Faso Continuous Multisectoral Survey 2014. Washington DC, United States: World Bank.	X	4				All ages	Both
Burundi	1998	Burundi Institute of Statistics and Economic Studies. Burundi Priority Survey 1998-1999.	X	2				All ages	Both
Burundi	2002	Burundi Institute of Statistics and Economic Studies. Burundi Survey on Indicators of Development 2002.	X	4				All ages	Both
Cambodia	2003	National Institute of Statistics (Cambodia), Statistics Sweden. Cambodia Socio-Economic Survey 2003-2005. Phnom Penh, Cambodia: National Institute of Statistics (Cambodia).	X	4	X	4		All ages	Both
Cambodia	2006	National Institute of Statistics (Cambodia), Statistics Sweden. Cambodia Socio-Economic Survey 2006-2007. Phnom Penh, Cambodia: National Institute of Statistics (Cambodia).	X	4	X	4		All ages	Both
Cambodia	2007	National Institute of Statistics (Cambodia), Statistics Sweden. Cambodia Socio-Economic Survey 2007-2008. Phnom Penh, Cambodia: National Institute of Statistics (Cambodia).	X	4.3	X	4.3		All ages	Both

Cambodia	2009	National Institute of Statistics (Cambodia), Statistics Sweden. Cambodia Socio-Economic Survey 2009. Phnom Penh, Cambodia: National Institute of Statistics (Cambodia).	X	4.3				All ages	Both
Cambodia	2010	National Institute of Statistics (Cambodia), Statistics Sweden. Cambodia Socio-Economic Survey 2010. Phnom Penh, Cambodia: National Institute of Statistics (Cambodia).	X	4.3				0-95	Both
Cambodia	2011	National Institute of Statistics (Cambodia), Statistics Sweden. Cambodia Socio-Economic Survey 2011. Phnom Penh, Cambodia: National Institute of Statistics (Cambodia).	X	4.3				0-95	Both
Cambodia	2012	Ministry of Planning (Cambodia), National Institute of Statistics (Cambodia). Cambodia Socio-Economic Survey 2012. Phnom Penh, Cambodia: National Institute of Statistics (Cambodia).	X	4.3				All ages	Both
Cambodia	2013	National Institute of Statistics (Cambodia), Statistics Sweden. Cambodia Socio-Economic Survey 2013. Phnom Penh, Cambodia: National Institute of Statistics (Cambodia).	X	4.3				0-20	Both
Cambodia	2014	National Institute of Statistics (Cambodia), Statistics Sweden. Cambodia Socio-Economic Survey 2014. Phnom Penh, Cambodia: National Institute of Statistics (Cambodia).	X	4.3				All ages	Both
Canada	2000	Statistics Canada. Canada Community Health Survey 2000-2001. Ottawa, Canada: Statistics Canada, 2003.			X	52		10-85	Both
Canada	2000	Environics Research Group, World Health Organization (WHO). Canada WHO Multi-country Survey Study on Health and Health System Responsiveness 2000-2001. Geneva, Switzerland: World Health Organization (WHO).	X	4.3	X	4.3		0-95	Both
Canada	2001, 2002, 2003, 2004, 2005, 2006, 2007, 2008, 2009, 2010, 2011, 2012, 2013, 2014	Organization for Economic Co-operation and Development (OECD). OECD Health Statistics. Paris, France: Organization for Economic Co-operation and Development (OECD).	X				X	All ages	Both
Canada	2005	Statistics Canada. Canada Community Health Survey 2005. Ottawa, Canada: Statistics Canada.			X	52		0-85	Both
Canada	2007	Statistics Canada. Canada Community Health Survey 2007-2008. Ottawa, Canada: Statistics Canada.			X	52		0-85	Both
Cape Verde	2007	National Institute of Statistics (INE) (Cape Verde) and World Bank (WB), Cape Verde Core Welfare Indicator Questionnaire Survey 2007. Praia, Cape Verde: National Institute of Statistics (INE) (Cape Verde).	X	4				All ages	Both
Chad	2003	World Health Organization (WHO). Chad World Health Survey 2003. Geneva, Switzerland: World Health Organization (WHO), 2005.			X	52		All ages	Both
Chile	1990	Department of Economics, University of Chile, Ministry of Planning (Chile). Chile National Socioeconomic Characterization Survey 1990.	X	12.9				All ages	Both
Chile	1992	Department of Economics, University of Chile, Ministry of Planning (Chile). Chile National Socioeconomic Characterization Survey 1992.	X	12.9	X	12.9		All ages	Both
Chile	1994	Department of Economics, University of Chile, Ministry of Planning (Chile). Chile National Socioeconomic Characterization Survey 1994. Santiago, Chile: Ministry of Social Development (Chile).	X	12.9	X	12.9		All ages	Both
Chile	1996	Department of Economics, University of Chile, Ministry of Planning (Chile). Chile National Socioeconomic Characterization Survey 1996. Santiago, Chile: Ministry of Social Development (Chile).	X	12.9	X	12.9		All ages	Both
Chile	1998	Department of Economics, University of Chile, Ministry of Planning (Chile). Chile National Socioeconomic Characterization Survey 1998.	X	12.9	X	12.9		All ages	Both
Chile	1999	Pan American Health Organization (PAHO), Center for Demography and Ecology, University of Wisconsin-Madison, Inter-University Consortium for Political and Social Research (ICPSR), Institute of Nutrition and Food Technology (INTA), University of Chile, Center for Geriatrics and Gerontology, Pontifical Catholic University of Chile. Chile - Santiago Survey on Health, Well-Being, and Aging in Latin America and the Caribbean 1999-2000. Ann Arbor, United States: Inter-University Consortium for Political and Social Research (ICPSR).	X	17.2	X	17.2		60-100+	Both
Chile	2000	Department of Economics, University of Chile, Ministry of Planning (Chile). Chile National Socioeconomic Characterization Survey 2000. Santiago, Chile: Ministry of Social Development (Chile).	X	12.9	X	52		All ages	Both
Chile	2000	Ministry of Health (Chile), National Institute of Statistics (Chile). Chile National Quality of Life and Health Survey 2000. Santiago, Chile: Ministry of Health (Chile).	X	4.3				All ages	Both
Chile	2000	University of Concepcion (Chile), World Health Organization (WHO). Chile WHO Multi-country Survey Study on Health and Health System Responsiveness 2000-2001. Geneva, Switzerland: World Health Organization (WHO).	X	4.3	X	4.3		All ages	Both

Chile	2001, 2002, 2003, 2004, 2005, 2006, 2007, 2008, 2009, 2010, 2011, 2012, 2013, 2014	Organization for Economic Co-operation and Development (OECD). OECD Health Statistics. Paris, France: Organization for Economic Co-operation and Development (OECD).	X				X	All ages	Both
Chile	2001	Ministry of Health (Chile). Chile Hospital Discharges 2001. Santiago, Chile: Ministry of Health (Chile).			X		X	0-99	Both
Chile	2002	Ministry of Health (Chile). Chile Hospital Discharges 2002. Santiago, Chile: Ministry of Health (Chile).			X		X	0-99	Both
Chile	2003	Department of Economics, University of Chile, Ministry of Planning (Chile). Chile National Socioeconomic Characterization Survey 2003. Santiago, Chile: Ministry of Social Development (Chile).	X	12.9	X	52		All ages	Both
Chile	2003	Ministry of Health (Chile). Chile Hospital Discharges 2003. Santiago, Chile: Ministry of Health (Chile).			X		X	0-99	Both
Chile	2004	Ministry of Health (Chile). Chile Hospital Discharges 2004. Santiago, Chile: Ministry of Health (Chile).			X		X	0-99	Both
Chile	2005	Ministry of Health (Chile). Chile Hospital Discharges 2005. Santiago, Chile: Ministry of Health (Chile).			X		X	0-99	Both
Chile	2006	Department of Economics, University of Chile, Ministry of Planning (Chile). Chile National Socioeconomic Characterization Survey 2006. Santiago, Chile: Ministry of Social Development (Chile).	X	12.9	X	52		All ages	Both
Chile	2006	Ministry of Health (Chile). Chile Hospital Discharges 2006. Santiago, Chile: Ministry of Health (Chile).			X		X	0-99	Both
Chile	2007	Ministry of Health (Chile). Chile Hospital Discharges 2007. Santiago, Chile: Ministry of Health (Chile).			X		X	0-99	Both
Chile	2008	Ministry of Health (Chile). Chile Hospital Discharges 2008. Santiago, Chile: Ministry of Health (Chile).			X		X	0-99	Both
Chile	2009	Ministry of Health (Chile). Chile Hospital Discharges 2009. Santiago, Chile: Ministry of Health (Chile).			X		X	0-99	Both
Chile	2009	Ministry of Planning (Chile), Social Observatory, Alberto Hurtado University. Chile National Socioeconomic Characterization Survey 2009. Santiago, Chile: Ministry of Social Development (Chile).	X	12.9	X	52		All ages	Both
Chile	2010	Ministry of Health (Chile). Chile Hospital Discharges 2010. Santiago, Chile: Ministry of Health (Chile).			X		X	0-99	Both
Chile	2011	Ministry of Health (Chile). Chile Hospital Discharges 2011. Santiago, Chile: Ministry of Health (Chile).			X		X	0-99	Both
Chile	2011	ISSP Research Group (2009): International Social Survey Programme: Health and Health Care - ISSP 2011. GESIS Data Archive, Cologne. ZA5800 Data file version 3.0.0, doi:10.4232/1.12252.	X	52				15-100+	Both
Chile	2012	Ministry of Health (Chile). Chile Hospital Discharges 2012. Santiago, Chile: Ministry of Health (Chile).			X		X	0-99	Both
Chile	2015	Microdata Center, Department of Economics, University of Chile, Ministry of Social Development (Chile), National Institute of Statistics (Chile). Chile National Socioeconomic Characterization Survey 2015-2016. Santiago, Chile: Ministry of Social Development (Chile).	X	12.9				All ages	Both
Chile	2015	National Office of Statistics (Cuba). Cuba Statistical Yearbook 2012. Havana, Cuba: National Office of Statistics (Cuba).			X	52		All ages	Both
China	1990, 1991, 1992, 1993, 1994, 1995, 1996, 1997, 1998, 1999, 2000, 2001, 2002, 2003, 2004, 2005, 2006, 2007, 2008, 2009, 2010, 2011, 2012, 2013, 2014	Organization for Economic Co-operation and Development (OECD). OECD Health Statistics. Paris, France: Organization for Economic Co-operation and Development (OECD).			X		X	All ages	Both
China	1991, 1993, 1997, 2000,	Carolina Population Center, University of North Carolina at Chapel Hill, Chinese Center for Disease Control and Prevention (CCDC). China Health and Nutrition Survey. Chapel Hill,	X	4	X	4		All ages	Both

	2004, 2006, 2009	United States: Carolina Population Center, University of North Carolina at Chapel Hill.							
China	1996	National Bureau of Statistics of China. China Statistical Yearbook 1996. Beijing, China: National Bureau of Statistics of China.	X				X	All ages	Both
China	1997	National Bureau of Statistics of China. China Statistical Yearbook 1997. Beijing, China: National Bureau of Statistics of China.	X				X	All ages	Both
China	1998	National Bureau of Statistics of China. China Statistical Yearbook 2003. Beijing, China: National Bureau of Statistics of China.	X				X	All ages	Both
China	1999	National Bureau of Statistics of China. China Statistical Yearbook 2003. Beijing, China: National Bureau of Statistics of China.	X				X	All ages	Both
China	2000	National Bureau of Statistics of China. China Statistical Yearbook 2003. Beijing, China: National Bureau of Statistics of China.	X				X	All ages	Both
China	2000	Institute of Social Medicine and Health Policy, Shandong University, Shandong University School of Medicine, World Health Organization (WHO). China WHO Multi-country Survey Study on Health and Health System Responsiveness 2000-2001.	X	4.3	X	4.3		0-95	Both
China	2001	National Bureau of Statistics of China. China Statistical Yearbook 2003. Beijing, China: National Bureau of Statistics of China.	X				X	All ages	Both
China	2002	World Health Organization (WHO). China World Health Survey 2002. Geneva, Switzerland: World Health Organization (WHO), 2005.			X	52		15-100+	Both
China	2002	National Bureau of Statistics of China. China Statistical Yearbook 2003. Beijing, China: National Bureau of Statistics of China.	X				X	All ages	Both
China	2003	National Bureau of Statistics of China. China Statistical Yearbook 2003. Beijing, China: National Bureau of Statistics of China.	X				X	All ages	Both
China	2005	National Bureau of Statistics of China. China Statistical Yearbook 2005. Beijing, China: National Bureau of Statistics of China.	X				X	All ages	Both
China	2007	Ministry of Health (China), National Center for Chronic and Noncommunicable Disease Control and Prevention (China), World Health Organization (WHO). China WHO Study on Global AGEing and Adult Health 2007-2010.	X	52				All ages	Both
China	2007	National Bureau of Statistics of China. China Statistical Yearbook 2007. Beijing, China: National Bureau of Statistics of China.	X				X	All ages	Both
China	2008	National Bureau of Statistics of China. China Statistical Yearbook 2003. Beijing, China: National Bureau of Statistics of China.	X				X	All ages	Both
China	2008	China Center for Economic Research, Peking University. China Health and Retirement Longitudinal Study 2008. Beijing, China: China Center for Economic Research, Peking University.	X	4.3				30-95	Both
China	2012	China Center for Economic Research, Peking University. China Health and Retirement Longitudinal Study Pilot Resurvey 2012. Beijing, China: China Center for Economic Research, Peking University.	X	4.3	X	52		All ages	Both
China	2015	Institute for Health Metrics and Evaluation (IHME). United States Expenditure by Disease Cost to Payment Estimates 1996-2012.	X				X	All ages	Both
China	2015	National Bureau of Statistics of China. China Statistical Yearbook 2015. Beijing, China: National Bureau of Statistics of China.			X		X	All ages	Both
Colombia	1997	National Administrative Department of Statistics (Colombia). Colombia National Quality of Life Survey 1997. Bogotá, Colombia: National Administrative Department of Statistics (Colombia).			X	52		All ages	Both
Colombia	2000	Pontificia Universidad Javeriana (Colombia), World Health Organization (WHO). Colombia WHO Multi-country Survey Study on Health and Health System Responsiveness 2000-2001. Geneva, Switzerland: World Health Organization (WHO).	X	4.3	X	4.3		15-100+	Both
Colombia	2007	Administrative Department of Science, Techonology, and Innovation (Colombia), Center for Development Projects, Pontifical Xavierian University, Ministry of Social Protection (Colombia), Specialized Information Systems. Colombia National Health Survey 2007-2008.	X	4.3	X	52		5-70	Both
Colombia	2008	National Administrative Department of Statistics (Colombia). Colombia National Quality of Life Survey 2008. Bogotá, Colombia: National Administrative Department of Statistics (Colombia).			X	52		All ages	Both
Colombia	2009, 2010, 2011, 2012, 2013	Organization for Economic Co-operation and Development (OECD). OECD Health Statistics. Paris, France: Organization for Economic Co-operation and Development (OECD).			X		X	All ages	Both
Colombia	2010	National Administrative Department of Statistics (Colombia). Colombia National Quality of Life Survey 2010. Bogotá, Colombia: National Administrative Department of Statistics (Colombia), 2012.	X	4.3	X	52		All ages	Both
Comoros	2003	World Health Organization (WHO). Comoros World Health Survey 2003. Geneva, Switzerland: World Health Organization (WHO), 2005.			X	52		15-95	Both

Costa Rica	2000	Gallup Europe, World Health Organization (WHO). Costa Rica WHO Multi-country Survey Study on Health and Health System Responsiveness 2000-2001. Geneva, Switzerland: World Health Organization (WHO).	X	4.3	X	4.3		15-95	Both
Costa Rica	2006	Central American Population Center, University of Costa Rica. Costa Rica Survey of Family Health Services and Expenses 2008. San José, Costa Rica: Central American Population Center, University of Costa Rica.	X	52	X	52		All ages	Both
Cote d'Ivoire	2003	World Health Organization (WHO). Côte d'Ivoire World Health Survey 2003. Geneva, Switzerland: World Health Organization (WHO), 2005.			X	52		All ages	Both
Croatia	1981, 1982, 1983, 1984, 1985, 1986, 1987, 1988, 1989, 1990, 1991, 1992, 1993, 1994, 1995, 1996, 1997, 1998, 1999, 2000, 2001, 2002, 2003, 2004, 2005, 2006, 2007, 2008, 2009, 2010, 2011, 2012, 2013, 2014, 2015	World Health Organization Regional Office for Europe (WHO/Europe). European Health for All Database - Inpatient Care Discharges Per 100. Copenhagen, Denmark: World Health Organization Regional Office for Europe (WHO/Europe).			X		X	All ages	Both
Croatia	2000	Market, Media, and Public Opinion Research (Croatia), World Health Organization (WHO). Croatia WHO Multi-country Survey Study on Health and Health System Responsiveness 2000-2001. Geneva, Switzerland: World Health Organization (WHO).	X	4.3	X	4.3		15-95	Both
Croatia	2003, 2004, 2005, 2006, 2007	World Health Organization Regional Office for Europe (WHO/Europe). European Hospital Morbidity Database 1999-2007. Copenhagen, Denmark: World Health Organization Regional Office for Europe (WHO/Europe).			X		X	All ages	Both
Croatia	2003	World Health Organization (WHO). Croatia World Health Survey 2003. Geneva, Switzerland: World Health Organization (WHO), 2005.			X	52		15-95	Both
Croatia	2011	ISSP Research Group (2009): International Social Survey Programme: Health and Health Care - ISSP 2011. GESIS Data Archive, Cologne. ZA5800 Data file version 3.0.0, doi:10.4232/1.12252.	X	52				0-90	Both
Cuba	1999	Pan American Health Organization (PAHO), Center for Demography and Ecology, University of Wisconsin-Madison, Inter-University Consortium for Political and Social Research (ICPSR), Centre for Population and Development Studies, National Statistics Office (Cuba), Iberoamerican Center for the Third Age. Cuba - Havana Survey on Health, Well-Being, and Aging in Latin America and the Caribbean 1999-2000. Ann Arbor, United States: Inter-University Consortium for Political and Social Research (ICPSR).	X	17.2				60-100+	Both
Cyprus	2000	MEMRB International, World Health Organization (WHO). Cyprus WHO Multi-country Survey Study on Health and Health System Responsiveness 2000-2001. Geneva, Switzerland: World Health Organization (WHO).	X	4.3	X	4.3		All ages	Both
Cyprus	2011	World Health Organization Regional Office for Europe (WHO/Europe). European Hospital Morbidity Database 1999-2007. Copenhagen, Denmark: World Health Organization Regional Office for Europe (WHO/Europe).			X		X	All ages	Both
Czech Republic	1998	Institute of Health Information and Statistics of the Czech Republic. Czech Republic Surveys on Activity of Health Establishments 1998.	X				X	All ages	Both
Czech Republic	1999, 2001, 2002, 2003, 2004, 2005, 2006, 2007, 2009,	World Health Organization Regional Office for Europe (WHO/Europe). European Hospital Morbidity Database 1999-2007. Copenhagen, Denmark: World Health Organization Regional Office for Europe (WHO/Europe).			X		X	All ages	Both

	2010, 2011								
Czech Republic	1999	Institute of Health Information and Statistics of the Czech Republic. Czech Republic Surveys on Activity of Health Establishments 1999.	X				X	All ages	Both
Czech Republic	2000	Institute of Health Information and Statistics of the Czech Republic, International Research Associates (INRA) Europe, World Health Organization (WHO). Czech Republic WHO Multi-country Survey Study on Health and Health System Responsiveness 2000-2001. Geneva, Switzerland: World Health Organization (WHO).	X	4.3	X	4.3		15-90	Both
Czech Republic	2000	Institute of Health Information and Statistics of the Czech Republic. Czech Republic Surveys on Activity of Health Establishments 2000.	X				X	All ages	Both
Czech Republic	2001	Institute of Health Information and Statistics of the Czech Republic. Czech Republic Surveys on Activity of Health Establishments 2001.	X				X	All ages	Both
Czech Republic	2002	World Health Organization (WHO). Czech Republic World Health Survey 2002-2003. Geneva, Switzerland: World Health Organization (WHO), 2005.			X	52		15-95	Both
Czech Republic	2002	Institute of Health Information and Statistics of the Czech Republic. Czech Republic Surveys on Activity of Health Establishments 2002.	X				X	All ages	Both
Czech Republic	2003	Institute of Health Information and Statistics of the Czech Republic. Czech Republic Surveys on Activity of Health Establishments 2003.	X				X	All ages	Both
Czech Republic	2004	Institute of Health Information and Statistics of the Czech Republic. Czech Republic Surveys on Activity of Health Establishments 2004.	X				X	All ages	Both
Czech Republic	2005	Institute of Health Information and Statistics of the Czech Republic. Czech Republic Surveys on Activity of Health Establishments 2005.	X				X	All ages	Both
Czech Republic	2006	Institute of Health Information and Statistics of the Czech Republic. Czech Republic Surveys on Activity of Health Establishments 2006.	X				X	All ages	Both
Czech Republic	2007	Institute of Health Information and Statistics of the Czech Republic. Czech Republic Surveys on Activity of Health Establishments 2007.	X				X	All ages	Both
Czech Republic	2008	Institute of Health Information and Statistics of the Czech Republic. Czech Republic Surveys on Activity of Health Establishments 2008.	X				X	All ages	Both
Czech Republic	2009	Institute of Health Information and Statistics of the Czech Republic. Czech Republic Surveys on Activity of Health Establishments 2009.	X				X	All ages	Both
Czech Republic	2010	Börsch-Supan, A. (2013). Survey of Health, Ageing and Retirement in Europe (SHARE) Wave 2. Release version: 2.6.0. SHARE-ERIC. Data set. DOI: 10.6103/SHARE.w2.260	X	52	X	52		35-100+	Both
Czech Republic	2010	Institute of Health Information and Statistics of the Czech Republic. Czech Republic Surveys on Activity of Health Establishments 2010.	X				X	All ages	Both
Czech Republic	2011	ISSP Research Group (2009): International Social Survey Programme: Health and Health Care - ISSP 2011. GESIS Data Archive, Cologne. ZA5800 Data file version 3.0.0, doi:10.4232/1.12252.	X	52				15-95	Both
Czech Republic	2011	Institute of Health Information and Statistics of the Czech Republic. Czech Republic Surveys on Activity of Health Establishments 2011.	X				X	All ages	Both
Czech Republic	2012	Börsch-Supan, A. (2013). Survey of Health, Ageing and Retirement in Europe (SHARE) Wave 4. Release version: 1.1.1. SHARE-ERIC. Data set. DOI: 10.6103/SHARE.w4.111	X	52	X	52		All ages	Both
Czech Republic	2012	Institute of Health Information and Statistics of the Czech Republic. Czech Republic Surveys on Activity of Health Establishments 2012.	X				X	All ages	Both
Czech Republic	2013	Börsch-Supan, A. (2015). Survey of Health, Ageing and Retirement in Europe (SHARE) Wave 5. Release version: 1.0.0. SHARE-ERIC. Data set. DOI: 10.6103/SHARE.w5.100	X	52	X	52		All ages	Both
Czech Republic	2013	Institute of Health Information and Statistics of the Czech Republic. Czech Republic Surveys on Activity of Health Establishments 2013.	X				X	All ages	Both
Denmark	1979	Danish Health and Medicines Authority. Denmark National Patient Registry 1979.			X		X	All ages	Both
Denmark	1980	Danish Health and Medicines Authority. Denmark National Patient Registry 1980.			X		X	All ages	Both
Denmark	1981	Danish Health and Medicines Authority. Denmark National Patient Registry 1981.			X		X	All ages	Both
Denmark	1982	Danish Health and Medicines Authority. Denmark National Patient Registry 1982.			X		X	All ages	Both
Denmark	1983	Danish Health and Medicines Authority. Denmark National Patient Registry 1983.			X		X	All ages	Both
Denmark	1984	Danish Health and Medicines Authority. Denmark National Patient Registry 1984.			X		X	All ages	Both
Denmark	1985	Danish Health and Medicines Authority. Denmark National Patient Registry 1985.			X		X	All ages	Both
Denmark	1986	Danish Health and Medicines Authority. Denmark National Patient Registry 1986.			X		X	All ages	Both
Denmark	1987	Danish Health and Medicines Authority. Denmark National Patient Registry 1987.			X		X	All ages	Both

Denmark	1988	Danish Health and Medicines Authority. Denmark National Patient Registry 1988.			X		X	All ages	Both
Denmark	1989	Danish Health and Medicines Authority. Denmark National Patient Registry 1989.			X		X	All ages	Both
Denmark	1990	Danish Health and Medicines Authority. Denmark National Patient Registry 1990.			X		X	All ages	Both
Denmark	1991	Danish Health and Medicines Authority. Denmark National Patient Registry 1991.			X		X	All ages	Both
Denmark	1992	Danish Health and Medicines Authority. Denmark National Patient Registry 1992.			X		X	All ages	Both
Denmark	1993	Danish Health and Medicines Authority. Denmark National Patient Registry 1993.			X		X	All ages	Both
Denmark	1994	Danish Health and Medicines Authority. Denmark National Patient Registry 1994.			X		X	All ages	Both
Denmark	1995	Danish Health and Medicines Authority. Denmark National Patient Registry 1995.			X		X	All ages	Both
Denmark	1996	Danish Health and Medicines Authority. Denmark National Patient Registry 1996.			X		X	All ages	Both
Denmark	1997	Danish Health and Medicines Authority. Denmark National Patient Registry 1997.			X		X	All ages	Both
Denmark	1998	Danish Health and Medicines Authority. Denmark National Patient Registry 1998.			X		X	All ages	Both
Denmark	1999, 2000	World Health Organization Regional Office for Europe (WHO/Europe). European Health for All Database - Outpatient Contacts Per Person Per Year. Copenhagen, Denmark: World Health Organization Regional Office for Europe (WHO/Europe).	X				X	All ages	Both
Denmark	1999	Danish Health and Medicines Authority. Denmark National Patient Registry 1999.			X		X	All ages	Both
Denmark	2000	Statistics Denmark, World Health Organization (WHO). Denmark WHO Multi-country Survey Study on Health and Health System Responsiveness 2000-2001. Geneva, Switzerland: World Health Organization (WHO).	X	4.3	X	4.3		All ages	Both
Denmark	2000	Danish Health and Medicines Authority. Denmark National Patient Registry 2000.			X		X	All ages	Both
Denmark	2001, 2002, 2003, 2004, 2005, 2006, 2007, 2008, 2009, 2010, 2011, 2012, 2013, 2014, 2015, 2016	Organization for Economic Co-operation and Development (OECD). OECD Health Statistics. Paris, France: Organization for Economic Co-operation and Development (OECD).	X				X	All ages	Both
Denmark	2001	Danish Health and Medicines Authority. Denmark National Patient Registry 2001.			X		X	All ages	Both
Denmark	2002	Danish Health and Medicines Authority. Denmark National Patient Registry 2002.			X		X	All ages	Both
Denmark	2003	World Health Organization (WHO). Denmark World Health Survey 2003. Geneva, Switzerland: World Health Organization (WHO), 2005.			X	52		15-95	Both
Denmark	2003	Danish Health and Medicines Authority. Denmark National Patient Registry 2003.			X		X	All ages	Both
Denmark	2004	Danish Health and Medicines Authority. Denmark National Patient Registry 2004.			X		X	All ages	Both
Denmark	2005	Danish Health and Medicines Authority. Denmark National Patient Registry 2005.			X		X	All ages	Both
Denmark	2006	Börsch-Supan, A. (2013). Survey of Health, Ageing and Retirement in Europe (SHARE) Wave 1. Release version: 2.6.0. SHARE-ERIC. Data set. DOI: 10.6103/SHARE.w1.260	X	52	X	52		All ages	Both
Denmark	2006	Danish Health and Medicines Authority. Denmark National Patient Registry 2006.			X		X	All ages	Both
Denmark	2007	Danish Health and Medicines Authority. Denmark National Patient Registry 2007.			X		X	All ages	Both
Denmark	2008	Danish Health and Medicines Authority. Denmark National Patient Registry 2008.			X		X	All ages	Both
Denmark	2009	Danish Health and Medicines Authority. Denmark National Patient Registry 2009.			X		X	All ages	Both
Denmark	2010	Börsch-Supan, A. (2013). Survey of Health, Ageing and Retirement in Europe (SHARE) Wave 2. Release version: 2.6.0. SHARE-ERIC. Data set. DOI: 10.6103/SHARE.w2.260	X	52	X	52		30-100+	Both
Denmark	2010	Danish Health and Medicines Authority. Denmark National Patient Registry 2010.			X		X	All ages	Both
Denmark	2011	Danish Health and Medicines Authority. Denmark National Patient Registry 2011.			X		X	All ages	Both
Denmark	2012	Börsch-Supan, A. (2013). Survey of Health, Ageing and Retirement in Europe (SHARE) Wave 4. Release version: 1.1.1. SHARE-ERIC. Data set. DOI: 10.6103/SHARE.w4.111	X	52	X	52		30-100+	Both
Denmark	2012	Danish Health and Medicines Authority. Denmark National Patient Registry 2012.			X		X	All ages	Both
Denmark	2013	ISSP Research Group (2009): International Social Survey Programme: Health and Health Care - ISSP 2011. GESIS Data Archive, Cologne. ZA5800 Data file version 3.0.0, doi:10.4232/1.12252.	X	52				15-95	Both

Denmark	2013	Börsch-Supan, A. (2015). Survey of Health, Ageing and Retirement in Europe (SHARE) Wave 5. Release version: 1.0.0. SHARE-ERIC. Data set. DOI: 10.6103/SHARE.w5.100	X	52	X	52		All ages	Both
Denmark	2013	Danish Health and Medicines Authority. Denmark National Patient Registry 2013.			X		X	All ages	Both
Dominica	2001	Canadian International Development Agency (CIDA), Caribbean Community (CARICOM) Secretariat. Dominica Population and Housing Census 2001.	X	4.3				All ages	Both
Dominican Republic	2003	World Health Organization (WHO). Dominican Republic World Health Survey 2003. Geneva, Switzerland: World Health Organization (WHO), 2005.			X	52		15-100+	Both
Ecuador	1997	National Institute of Statistics and Censuses (Ecuador). Ecuador Hospital Inpatient Discharges 1997. Quito, Ecuador: National Institute of Statistics and Censuses (Ecuador).			X		X	0-99	Both
Ecuador	1998	National Institute of Statistics and Censuses (Ecuador). Ecuador Hospital Inpatient Discharges 1998. Quito, Ecuador: National Institute of Statistics and Censuses (Ecuador).			X		X	0-99	Both
Ecuador	1999	National Institute of Statistics and Censuses (Ecuador). Ecuador Hospital Inpatient Discharges 1999. Quito, Ecuador: National Institute of Statistics and Censuses (Ecuador).			X		X	0-99	Both
Ecuador	2000	National Institute of Statistics and Censuses (Ecuador). Ecuador Hospital Inpatient Discharges 2000. Quito, Ecuador: National Institute of Statistics and Censuses (Ecuador).			X		X	0-99	Both
Ecuador	2001	National Institute of Statistics and Censuses (Ecuador). Ecuador Hospital Inpatient Discharges 2001. Quito, Ecuador: National Institute of Statistics and Censuses (Ecuador).			X		X	0-99	Both
Ecuador	2002	National Institute of Statistics and Censuses (Ecuador). Ecuador Hospital Inpatient Discharges 2002. Quito, Ecuador: National Institute of Statistics and Censuses (Ecuador).			X		X	0-99	Both
Ecuador	2003	World Health Organization (WHO). Ecuador World Health Survey 2003. Geneva, Switzerland: World Health Organization (WHO), 2005.			X	52		All ages	Both
Ecuador	2003	National Institute of Statistics and Censuses (Ecuador). Ecuador Hospital Inpatient Discharges 2003. Quito, Ecuador: National Institute of Statistics and Censuses (Ecuador).			X		X	0-99	Both
Ecuador	2004	National Institute of Statistics and Censuses (Ecuador). Ecuador Hospital Inpatient Discharges 2004. Quito, Ecuador: National Institute of Statistics and Censuses (Ecuador).			X		X	0-99	Both
Ecuador	2005	National Institute of Statistics and Censuses (Ecuador). Ecuador Hospital Inpatient Discharges 2005. Quito, Ecuador: National Institute of Statistics and Censuses (Ecuador).			X		X	0-99	Both
Ecuador	2006	National Institute of Statistics and Censuses (Ecuador). Ecuador Hospital Inpatient Discharges 2006. Quito, Ecuador: National Institute of Statistics and Censuses (Ecuador).			X		X	0-99	Both
Ecuador	2007	National Institute of Statistics and Censuses (Ecuador). Ecuador Hospital Inpatient Discharges 2007. Quito, Ecuador: National Institute of Statistics and Censuses (Ecuador).			X		X	0-99	Both
Ecuador	2008	National Institute of Statistics and Censuses (Ecuador). Ecuador Hospital Inpatient Discharges 2008. Quito, Ecuador: National Institute of Statistics and Censuses (Ecuador).			X		X	0-99	Both
Ecuador	2009	National Institute of Statistics and Censuses (Ecuador). Ecuador Hospital Inpatient Discharges 2009. Quito, Ecuador: National Institute of Statistics and Censuses (Ecuador).			X		X	0-99	Both
Ecuador	2010	National Institute of Statistics and Censuses (Ecuador). Ecuador Hospital Inpatient Discharges 2010. Quito, Ecuador: National Institute of Statistics and Censuses (Ecuador).			X		X	0-99	Both
Ecuador	2011	National Institute of Statistics and Censuses (Ecuador). Ecuador Hospital Inpatient Discharges 2011. Quito, Ecuador: National Institute of Statistics and Censuses (Ecuador).			X		X	0-99	Both
Ecuador	2012	National Institute of Statistics and Censuses (Ecuador). Ecuador Hospital Inpatient Discharges 2012. Quito, Ecuador: National Institute of Statistics and Censuses (Ecuador), 2013.			X		X	0-99	Both
Ecuador	2013	National Institute of Statistics and Censuses (Ecuador). Ecuador Hospital Inpatient Discharges 2013. Quito, Ecuador: National Institute of Statistics and Censuses (Ecuador).			X		X	0-99	Both
Ecuador	2014	National Institute of Statistics and Censuses (Ecuador). Ecuador Hospital Inpatient Discharges 2014. Quito, Ecuador: National Institute of Statistics and Censuses (Ecuador).			X		X	0-99	Both
Egypt	2000	Health Care International, World Health Organization (WHO). Egypt WHO Multi-country Survey Study on Health and Health System Responsiveness 2000-2001. Geneva, Switzerland: World Health Organization (WHO).	X	4.3	X	4.3		All ages	Both
Egypt	2009	Information and Decision Support Center (IDSC) (Egypt), Population Council. Egypt Survey of Young People 2009. New York City , United States: Population Council.	X	26				10-29	Both
El Salvador	2013	General Administration of Statistics and Censuses (El Salvador), Ministry of Economy (El Salvador). El Salvador Multipurpose Household Survey 2013. San Salvador, El Salvador: General Administration of Statistics and Censuses (El Salvador).	X	4.3	X	4.3		All ages	Both
El Salvador	2014	General Administration of Statistics and Censuses (El Salvador), Ministry of Economy (El Salvador). El Salvador Multipurpose Household Survey 2014. San Salvador, El Salvador: General Administration of Statistics and Censuses (El Salvador).	X	4.3	X	4.3		All ages	Both

England	1999, 2001, 2002, 2003, 2004, 2006, 2007, 2009, 2010	World Health Organization Regional Office for Europe (WHO/Europe). European Hospital Morbidity Database 1999-2007. Copenhagen, Denmark: World Health Organization Regional Office for Europe (WHO/Europe).			X		X	All ages	Both
England	2000, 2001, 2002, 2003, 2004, 2005, 2006, 2007, 2008, 2009, 2010, 2011, 2012, 2013, 2014	Organization for Economic Co-operation and Development (OECD). OECD Health Statistics. Paris, France: Organization for Economic Co-operation and Development (OECD).			X		X	All ages	Both
England	2000	National Centre for Social Research (NatCen), World Health Organization (WHO). United Kingdom WHO Multi-country Survey Study on Health and Health System Responsiveness 2000-2001. Geneva, Switzerland: World Health Organization (WHO).	X	4.3	X	4.3		All ages	Both
England	2004	World Health Organization (WHO). United Kingdom World Health Survey 2004. Geneva, Switzerland: World Health Organization (WHO), 2005.			X	52		All ages	Both
England	2006	Department of Health, Social Services and Public Safety (Northern Ireland), Information Centre for Health and Social Care, NHS, NHS England, NHS Health Scotland, NHS Wales. United Kingdom Hospital Patient and Discharge Data 2006.			X		X	All ages	Both
England	2012	Department of Health, Social Services and Public Safety (Northern Ireland), Information Centre for Health and Social Care, NHS, NHS England, NHS Health Scotland, NHS Wales. United Kingdom Hospital Patient and Discharge Data 2012.			X		X	All ages	Both
England	2013	Department of Health, Social Services and Public Safety (Northern Ireland), Information Centre for Health and Social Care, NHS, NHS England, NHS Health Scotland, NHS Wales. United Kingdom Hospital Patient and Discharge Data 2013.			X		X	All ages	Both
England	2014	Department of Health, Social Services and Public Safety (Northern Ireland), Information Centre for Health and Social Care, NHS, NHS England, NHS Health Scotland, NHS Wales. United Kingdom Hospital Patient and Discharge Data 2014.			X		X	All ages	Both
Estonia	1980	National Institute for Health Development (Estonia). Estonia Hospital Inpatient Discharges 1980.			X		X	All ages	Both
Estonia	1985	National Institute for Health Development (Estonia). Estonia Hospital Inpatient Discharges 1985.			X		X	All ages	Both
Estonia	1986	National Institute for Health Development (Estonia). Estonia Hospital Inpatient Discharges 1986.			X		X	All ages	Both
Estonia	1987	National Institute for Health Development (Estonia). Estonia Hospital Inpatient Discharges 1987.			X		X	All ages	Both
Estonia	1988	National Institute for Health Development (Estonia). Estonia Hospital Inpatient Discharges 1988.			X		X	All ages	Both
Estonia	1989	National Institute for Health Development (Estonia). Estonia Hospital Inpatient Discharges 1989.			X		X	All ages	Both
Estonia	1990	National Institute for Health Development (Estonia). Estonia Hospital Inpatient Discharges 1990.			X		X	All ages	Both
Estonia	1991	National Institute for Health Development (Estonia). Estonia Hospital Inpatient Discharges 1991.			X		X	All ages	Both
Estonia	1992	National Institute for Health Development (Estonia). Estonia Hospital Inpatient Discharges 1992.			X		X	All ages	Both
Estonia	1993	National Institute for Health Development (Estonia). Estonia Hospital Inpatient Discharges 1993.			X		X	All ages	Both
Estonia	1994	National Institute for Health Development (Estonia). Estonia Hospital Inpatient Discharges 1994.			X		X	All ages	Both
Estonia	1995	National Institute for Health Development (Estonia). Estonia Hospital Inpatient Discharges 1995.			X		X	All ages	Both
Estonia	1996	National Institute for Health Development (Estonia). Estonia Hospital Inpatient Discharges 1996.			X		X	All ages	Both
Estonia	1997	National Institute for Health Development (Estonia). Estonia Hospital Inpatient Discharges 1997.			X		X	All ages	Both
Estonia	1998	National Institute for Health Development (Estonia). Estonia Hospital Inpatient Discharges 1998.			X		X	All ages	Both
Estonia	1998	National Institute for Health Development (Estonia). Estonia Outpatient Contacts Per Person Per Year 1998.	X				X	All ages	Both
Estonia	1999, 2001	World Health Organization Regional Office for Europe (WHO/Europe). European Hospital Morbidity Database 1999-2007. Copenhagen, Denmark: World Health Organization Regional Office for Europe (WHO/Europe).			X		X	All ages	Both
Estonia	1999	National Institute for Health Development (Estonia). Estonia Hospital Inpatient Discharges 1999.			X		X	All ages	Both
Estonia	1999	National Institute for Health Development (Estonia). Estonia Outpatient Contacts Per Person Per Year 1999.	X				X	All ages	Both

Estonia	2000	International Research Associates (INRA) Europe, World Health Organization (WHO). Estonia WHO Multi-country Survey Study on Health and Health System Responsiveness 2000-2001. Geneva, Switzerland: World Health Organization (WHO).	X	4.3	X	4.3		15-90	Both
Estonia	2001, 2002, 2003, 2004, 2005, 2006, 2007, 2008, 2009, 2010, 2011, 2012, 2013, 2014, 2015	Organization for Economic Co-operation and Development (OECD). OECD Health Statistics. Paris, France: Organization for Economic Co-operation and Development (OECD).	X				X	All ages	Both
Estonia	2003	World Health Organization (WHO). Estonia World Health Survey 2003. Geneva, Switzerland: World Health Organization (WHO), 2005.			X	52		0-95	Both
Estonia	2003	Ministry of Social Affairs (Estonia). Estonia Hospital Inpatient Discharges 2003.			X		X	All ages	Both
Estonia	2004	Ministry of Social Affairs (Estonia). Estonia Hospital Inpatient Discharges 2004.			X		X	All ages	Both
Estonia	2005	Ministry of Social Affairs (Estonia). Estonia Hospital Inpatient Discharges 2005.			X		X	All ages	Both
Estonia	2006	Ministry of Social Affairs (Estonia). Estonia Hospital Inpatient Discharges 2006.			X		X	All ages	Both
Estonia	2007	Ministry of Social Affairs (Estonia). Estonia Hospital Inpatient Discharges 2007.			X		X	All ages	Both
Estonia	2008	Ministry of Social Affairs (Estonia). Estonia Hospital Inpatient Discharges 2008.			X		X	All ages	Both
Estonia	2009	Ministry of Social Affairs (Estonia). Estonia Hospital Inpatient Discharges 2009.			X		X	All ages	Both
Estonia	2010	Ministry of Social Affairs (Estonia). Estonia Hospital Inpatient Discharges 2010.			X		X	All ages	Both
Estonia	2011	Ministry of Social Affairs (Estonia). Estonia Hospital Inpatient Discharges 2011.			X		X	All ages	Both
Estonia	2012	Börsch-Supan, A. (2013). Survey of Health, Ageing and Retirement in Europe (SHARE) Wave 4. Release version: 1.1.1. SHARE-ERIC. Data set. DOI: 10.6103/SHARE.w4.111	X	52	X	52		25-100+	Both
Estonia	2012	National Institute for Health Development (Estonia). Estonia Hospital Inpatient Discharges 2012.			X		X	All ages	Both
Estonia	2013	Börsch-Supan, A. (2015). Survey of Health, Ageing and Retirement in Europe (SHARE) Wave 5. Release version: 1.0.0. SHARE-ERIC. Data set. DOI: 10.6103/SHARE.w5.100	X	52	X	52		All ages	Both
Estonia	2013	National Institute for Health Development (Estonia). Estonia Hospital Inpatient Discharges 2013.			X		X	All ages	Both
Estonia	2014	National Institute for Health Development (Estonia). Estonia Hospital Inpatient Discharges 2014.			X		X	All ages	Both
Ethiopia	1996	Central Statistical Agency (Ethiopia). Ethiopia Welfare Monitoring Survey 1996.	X	8.6				All ages	Both
Ethiopia	1999	Central Statistical Agency (Ethiopia). Ethiopia Welfare Monitoring and Income Expenditure Surveys 1999-2000. Addis Ababa, Ethiopia: Central Statistical Agency (Ethiopia).	X	8.6				All ages	Both
Ethiopia	2000	Central Statistical Agency (Ethiopia). Ethiopia Welfare Monitoring Survey 2000.	X	8.6				All ages	Both
Ethiopia	2003	World Health Organization (WHO). Ethiopia World Health Survey 2003. Geneva, Switzerland: World Health Organization (WHO), 2005.			X	52		All ages	Both
Ethiopia	2004	Central Statistical Agency (Ethiopia). Ethiopia Welfare Monitoring Survey 2004.	X	8.6				All ages	Both
Ethiopia	2011	Central Statistical Agency (Ethiopia), World Bank. Ethiopia Living Standards Measurement Study - Integrated Survey on Agriculture 2011-2012. Washington DC, United States: World Bank.	X	8.6				All ages	Both
Ethiopia	2013	Central Statistical Agency (Ethiopia), World Bank. Ethiopia Living Standards Measurement Study - Integrated Survey on Agriculture 2013-2014. Washington DC, United States: World Bank, 2015.	X	52				0-90	Both
Finland	1988	National Institute for Health and Welfare (THL) (Finland). Finland Hospital Discharge Register 1988.			X		X	All ages	Both
Finland	1989	National Institute for Health and Welfare (THL) (Finland). Finland Hospital Discharge Register 1989.			X		X	All ages	Both
Finland	1990	National Institute for Health and Welfare (THL) (Finland). Finland Hospital Discharge Register 1990.			X		X	All ages	Both
Finland	1991	National Institute for Health and Welfare (THL) (Finland). Finland Hospital Discharge Register 1991.			X		X	All ages	Both
Finland	1992	National Institute for Health and Welfare (THL) (Finland). Finland Hospital Discharge Register 1992.			X		X	All ages	Both
Finland	1993	National Institute for Health and Welfare (THL) (Finland). Finland Hospital Discharge Register 1993.			X		X	All ages	Both
Finland	1994	National Institute for Health and Welfare (THL) (Finland). Finland Hospital Discharge Register 1994.			X		X	All ages	Both
Finland	1995	National Institute for Health and Welfare (THL) (Finland). Finland Hospital Discharge Register 1995.			X		X	All ages	Both

Finland	1996	National Institute for Health and Welfare (THL) (Finland). Finland Hospital Discharge Register 1996.			X		X	All ages	Both
Finland	1997	National Institute for Health and Welfare (THL) (Finland). Finland Hospital Discharge Register 1997.			X		X	All ages	Both
Finland	1998	National Institute for Health and Welfare (THL) (Finland). Finland Hospital Discharge Register 1998.			X		X	All ages	Both
Finland	1998	National Institute for Health and Welfare (THL) (Finland). Finland Outpatient Contacts Per Person Per Year 1998.	X				X	All ages	Both
Finland	1999, 2001, 2002, 2003, 2004, 2005, 2006, 2007, 2009, 2010, 2011	World Health Organization Regional Office for Europe (WHO/Europe). European Hospital Morbidity Database 1999-2007. Copenhagen, Denmark: World Health Organization Regional Office for Europe (WHO/Europe).			X		X	All ages	Both
Finland	1999	National Institute for Health and Welfare (THL) (Finland). Finland Hospital Discharge Register 1999.			X		X	All ages	Both
Finland	1999	National Institute for Health and Welfare (THL) (Finland). Finland Outpatient Contacts Per Person Per Year 1999.	X				X	All ages	Both
Finland	2000	International Research Associates (INRA) Europe, National Research and Development Center for Welfare and Health (STAKES) (Finland), World Health Organization (WHO). Finland WHO Multi-country Survey Study on Health and Health System Responsiveness 2000-2001. Geneva, Switzerland: World Health Organization (WHO).	X	4.3	X	4.3		All ages	Both
Finland	2001, 2002, 2003, 2004, 2005, 2006, 2007, 2008, 2009, 2010, 2011, 2012, 2013, 2014, 2015	Organization for Economic Co-operation and Development (OECD). OECD Health Statistics. Paris, France: Organization for Economic Co-operation and Development (OECD).	X				X	All ages	Both
Finland	2004	World Health Organization (WHO). Finland World Health Survey 2004. Geneva, Switzerland: World Health Organization (WHO), 2005.			X	52		15-95	Both
Finland	2011	ISSP Research Group (2009): International Social Survey Programme: Health and Health Care - ISSP 2011. GESIS Data Archive, Cologne. ZA5800 Data file version 3.0.0, doi:10.4232/1.12252.	X	52				15-80	Both
Finland	2013	National Institute for Health and Welfare (THL) (Finland). Finland Hospital Discharge Register 2013.			X		X	All ages	Both
Finland	2014	National Institute for Health and Welfare (THL) (Finland). Finland Health Behavior and Health Among the Adult Population 2014.	X	52				All ages	Both
Finland	2014	National Institute for Health and Welfare (THL) (Finland). Finland Hospital Discharge Register 2014.			X		X	All ages	Both
France	1974, 1975, 1976, 1977, 1978, 1979, 1980, 1981, 1982, 1983, 1984, 1985, 1986, 1987, 1988, 1989, 1990, 1991, 1992, 1993, 1994, 1995, 1996, 1997, 1998, 1999, 2000, 2001, 2002, 2003, 2004, 2005, 2006,	Organization for Economic Co-operation and Development (OECD). OECD Health Statistics. Paris, France: Organization for Economic Co-operation and Development (OECD).			X		X	All ages	Both

	2007, 2008, 2009, 2010, 2011, 2012, 2013, 2014, 2015								
France	1999, 2000	World Health Organization Regional Office for Europe (WHO/Europe). European Health for All Database - Outpatient Contacts Per Person Per Year. Copenhagen, Denmark: World Health Organization Regional Office for Europe (WHO/Europe).	X				X	All ages	Both
France	2000	Erik Consulting, International Research Associates (INRA) Europe, World Health Organization (WHO). France WHO Multi-country Survey Study on Health and Health System Responsiveness 2000-2001. Geneva, Switzerland: World Health Organization (WHO).	X	4.3	X	4.3		All ages	Both
France	2003	World Health Organization (WHO). France World Health Survey 2003. Geneva, Switzerland: World Health Organization (WHO), 2005.			X	52		0-95	Both
France	2006	Börsch-Supan, A. (2013). Survey of Health, Ageing and Retirement in Europe (SHARE) Wave 1. Release version: 2.6.0. SHARE-ERIC. Data set. DOI: 10.6103/SHARE.w1.260	X	52	X	52		All ages	Both
France	2010	Börsch-Supan, A. (2013). Survey of Health, Ageing and Retirement in Europe (SHARE) Wave 2. Release version: 2.6.0. SHARE-ERIC. Data set. DOI: 10.6103/SHARE.w2.260	X	52	X	52		35-100+	Both
France	2011	ISSP Research Group (2009): International Social Survey Programme: Health and Health Care - ISSP 2011. GESIS Data Archive, Cologne. ZA5800 Data file version 3.0.0, doi:10.4232/1.12252.	X	52				15-100+	Both
France	2012	Börsch-Supan, A. (2013). Survey of Health, Ageing and Retirement in Europe (SHARE) Wave 4. Release version: 1.1.1. SHARE-ERIC. Data set. DOI: 10.6103/SHARE.w4.111	X	52	X	52		40-100+	Both
France	2013	Börsch-Supan, A. (2015). Survey of Health, Ageing and Retirement in Europe (SHARE) Wave 5. Release version: 1.0.0. SHARE-ERIC. Data set. DOI: 10.6103/SHARE.w5.100	X	52	X	52		All ages	Both
Georgia	1980	National Center for Disease Control and Public Health (Georgia). Georgia Inpatient Care Discharges per 100 1980.			X		X	All ages	Both
Georgia	1985	National Center for Disease Control and Public Health (Georgia). Georgia Inpatient Care Discharges per 100 1985.			X		X	All ages	Both
Georgia	1986	National Center for Disease Control and Public Health (Georgia). Georgia Inpatient Care Discharges per 100 1986.			X		X	All ages	Both
Georgia	1987	National Center for Disease Control and Public Health (Georgia). Georgia Inpatient Care Discharges per 100 1987.			X		X	All ages	Both
Georgia	1988	National Center for Disease Control and Public Health (Georgia). Georgia Inpatient Care Discharges per 100 1988.			X		X	All ages	Both
Georgia	1989	National Center for Disease Control and Public Health (Georgia). Georgia Inpatient Care Discharges per 100 1989.			X		X	All ages	Both
Georgia	1990	National Center for Disease Control and Public Health (Georgia). Georgia Inpatient Care Discharges per 100 1990.			X		X	All ages	Both
Georgia	1991	National Center for Disease Control and Public Health (Georgia). Georgia Inpatient Care Discharges per 100 1991.			X		X	All ages	Both
Georgia	1992	National Center for Disease Control and Public Health (Georgia). Georgia Inpatient Care Discharges per 100 1992.			X		X	All ages	Both
Georgia	1993	National Center for Disease Control and Public Health (Georgia). Georgia Inpatient Care Discharges per 100 1993.			X		X	All ages	Both
Georgia	1994	National Center for Disease Control and Public Health (Georgia). Georgia Inpatient Care Discharges per 100 1994.			X		X	All ages	Both
Georgia	1995	National Center for Disease Control and Public Health (Georgia). Georgia Inpatient Care Discharges per 100 1995.			X		X	All ages	Both
Georgia	1996	National Center for Disease Control and Public Health (Georgia). Georgia Inpatient Care Discharges per 100 1996.			X		X	All ages	Both
Georgia	1997	National Center for Disease Control and Public Health (Georgia). Georgia Inpatient Care Discharges per 100 1997.			X		X	All ages	Both
Georgia	1998	National Center for Disease Control and Public Health (Georgia). Georgia Inpatient Care Discharges per 100 1998.			X		X	All ages	Both
Georgia	1999	National Center for Disease Control and Public Health (Georgia). Georgia Inpatient Care Discharges per 100 1999.			X		X	All ages	Both
Georgia	2000	Institute for Polling and Marketing (Georgia), World Health Organization (WHO). Georgia WHO Multi-country Survey Study on Health and Health System Responsiveness 2000-2001.			X	4.3		All ages	Both

Georgia	2000	National Center for Disease Control and Public Health (Georgia). Georgia Inpatient Care Discharges per 100 2000.			X		X	All ages	Both
Georgia	2001	National Center for Disease Control and Public Health (Georgia). Georgia Inpatient Care Discharges per 100 2001.			X		X	All ages	Both
Georgia	2002	National Center for Disease Control and Public Health (Georgia). Georgia Inpatient Care Discharges per 100 2002.			X		X	All ages	Both
Georgia	2003	World Health Organization (WHO). Georgia World Health Survey 2003. Geneva, Switzerland: World Health Organization (WHO), 2005.			X	52		15-100+	Both
Georgia	2003	National Center for Disease Control and Public Health (Georgia). Georgia Inpatient Care Discharges per 100 2003.			X		X	All ages	Both
Georgia	2004	National Center for Disease Control and Public Health (Georgia). Georgia Inpatient Care Discharges per 100 2004.			X		X	All ages	Both
Georgia	2005	National Center for Disease Control and Public Health (Georgia). Georgia Inpatient Care Discharges per 100 2005.			X		X	All ages	Both
Georgia	2006	National Center for Disease Control and Public Health (Georgia). Georgia Inpatient Care Discharges per 100 2006.			X		X	All ages	Both
Georgia	2007	National Center for Disease Control and Public Health (Georgia). Georgia Inpatient Care Discharges per 100 2007.			X		X	All ages	Both
Georgia	2008	National Center for Disease Control and Public Health (Georgia). Georgia Inpatient Care Discharges per 100 2008.			X		X	All ages	Both
Georgia	2009	National Center for Disease Control and Public Health (Georgia). Georgia Inpatient Care Discharges per 100 2009.			X		X	All ages	Both
Georgia	2010	Concluzia-Prim Center for Survey Methodology (Moldova), Georgia Opinion Research Business International (GORBI), Institute for Advanced Studies (Austria), London School of Hygiene and Tropical Medicine, University of Aberdeen. Georgia Health in Times of Transition Household Survey 2010.			X	52		15-95	Both
Georgia	2010	National Center for Disease Control and Public Health (Georgia). Georgia Inpatient Care Discharges per 100 2010.			X		X	All ages	Both
Georgia	2011	National Center for Disease Control and Public Health (Georgia). Georgia Inpatient Care Discharges per 100 2011.			X		X	All ages	Both
Georgia	2012	National Center for Disease Control and Public Health (Georgia). Georgia Inpatient Care Discharges per 100 2012.			X		X	All ages	Both
Georgia	2013	National Center for Disease Control and Public Health (Georgia). Georgia Inpatient Care Discharges per 100 2013.			X		X	All ages	Both
Georgia	2014	National Center for Disease Control and Public Health (Georgia). Georgia Inpatient Care Discharges per 100 2014.			X		X	All ages	Both
Germany	1971, 1972, 1973, 1974, 1975, 1976, 1977, 1978, 1979, 1980, 1981, 1982, 1983, 1984, 1985, 1986, 1987, 1988, 1989, 1990	World Health Organization Regional Office for Europe (WHO/Europe). European Health for All Database - Inpatient Care Discharges Per 100. Copenhagen, Denmark: World Health Organization Regional Office for Europe (WHO/Europe).			X		X	All ages	Both
Germany	1990	Federal Statistical Office (Germany). Germany Federal Health Reporting Hospital Discharges 1990.			X		X	All ages	Both
Germany	1991	Federal Statistical Office (Germany). Germany Federal Health Reporting Hospital Discharges 1991.			X		X	All ages	Both
Germany	1992	Federal Statistical Office (Germany). Germany Federal Health Reporting Hospital Discharges 1992.			X		X	All ages	Both
Germany	1993	Federal Statistical Office (Germany). Germany Federal Health Reporting Hospital Discharges 1993.			X		X	All ages	Both
Germany	1994	Federal Statistical Office (Germany). Germany Federal Health Reporting Hospital Discharges 1994.			X		X	All ages	Both
Germany	1995	Federal Statistical Office (Germany). Germany Federal Health Reporting Hospital Discharges 1995.			X		X	All ages	Both
Germany	1996	Federal Statistical Office (Germany). Germany Federal Health Reporting Hospital Discharges 1996.			X		X	All ages	Both
Germany	1997	Federal Environment Agency (Germany), Federal Institute for Drugs and Medical Devices (Germany), Max Planck Institute of Psychiatry, Robert Koch Institute. Germany National Health Interview and Examination Survey 1997-1999. Berlin, Germany: Robert Koch Institute, 2000.	X	52	X	4		15-80	Both
Germany	1997	Federal Statistical Office (Germany). Germany Federal Health Reporting Hospital Discharges 1997.			X		X	All ages	Both
Germany	1998	Federal Statistical Office (Germany). Germany Federal Health Reporting Hospital Discharges 1998.			X		X	All ages	Both
Germany	1999	Federal Statistical Office (Germany). Germany Federal Health Reporting Hospital Discharges 1999.			X		X	All ages	Both

Germany	2000	International Research Associates (INRA) Europe, World Health Organization (WHO). Germany WHO Multi-country Survey Study on Health and Health System Responsiveness 2000-2001. Geneva, Switzerland: World Health Organization (WHO).	X	4.3	X	4.3		15-100+	Both
Germany	2000	Federal Statistical Office (Germany). Germany Federal Health Reporting Hospital Discharges 2000.			X		X	All ages	Both
Germany	2001	Federal Statistical Office (Germany). Germany Federal Health Reporting Hospital Discharges 2001.			X		X	All ages	Both
Germany	2002, 2003, 2004, 2005, 2006, 2007, 2009, 2010, 2011	World Health Organization Regional Office for Europe (WHO/Europe). European Hospital Morbidity Database 1999-2007. Copenhagen, Denmark: World Health Organization Regional Office for Europe (WHO/Europe).			X		X	All ages	Both
Germany	2002	Federal Statistical Office (Germany). Germany Federal Health Reporting Hospital Discharges 2002.			X		X	All ages	Both
Germany	2003	World Health Organization (WHO). Germany World Health Survey 2004. Geneva, Switzerland: World Health Organization (WHO), 2005.			X	52		15-95	Both
Germany	2006	Börsch-Supan, A. (2013). Survey of Health, Ageing and Retirement in Europe (SHARE) Wave 1. Release version: 2.6.0. SHARE-ERIC. Data set. DOI: 10.6103/SHARE.w1.260	X	52	X	52		All ages	Both
Germany	2009	Robert Koch Institute. Germany Health Update 2009-2010. Berlin, Germany: Robert Koch Institute.	X	52	X	52		5-20	Both
Germany	2010	Börsch-Supan, A. (2013). Survey of Health, Ageing and Retirement in Europe (SHARE) Wave 2. Release version: 2.6.0. SHARE-ERIC. Data set. DOI: 10.6103/SHARE.w2.260	X	52	X	52		30-100+	Both
Germany	2011	ISSP Research Group (2009): International Social Survey Programme: Health and Health Care - ISSP 2011. GESIS Data Archive, Cologne. ZA5800 Data file version 3.0.0, doi:10.4232/1.12252.	X	52				All ages	Both
Germany	2012	Börsch-Supan, A. (2013). Survey of Health, Ageing and Retirement in Europe (SHARE) Wave 4. Release version: 1.1.1. SHARE-ERIC. Data set. DOI: 10.6103/SHARE.w4.111	X	52	X	52		40-100+	Both
Germany	2013	Börsch-Supan, A. (2015). Survey of Health, Ageing and Retirement in Europe (SHARE) Wave 5. Release version: 1.0.0. SHARE-ERIC. Data set. DOI: 10.6103/SHARE.w5.100	X	52	X	52		All ages	Both
Germany	2013	Federal Statistical Office (Germany). Germany Federal Health Reporting Hospital Discharges 2013.			X		X	All ages	Both
Germany	2014	Federal Statistical Office (Germany). Germany Federal Health Reporting Hospital Discharges 2014.			X		X	All ages	Both
Ghana	1991	Ghana Statistical Service. Ghana Living Standards Measurement Survey 1991-1992. Accra, Ghana: Ghana Statistical Service.	X	2	X	2		All ages	Both
Ghana	1997, 2004	Ghana Statistical Service, World Bank. Ghana Core Welfare Indicators Survey 1997.	X	4				All ages	Both
Ghana	1998	Ghana Statistical Service. Ghana Living Standards Survey 1998-1999.	X	2	X	2		5-100+	Both
Ghana	2003	World Health Organization (WHO). Ghana World Health Survey 2003. Geneva, Switzerland: World Health Organization (WHO), 2005.			X	52		All ages	Both
Ghana	2005	Ghana Statistical Service. Ghana Living Standards Measurement Survey 2005-2006. Accra, Ghana: Ghana Statistical Service.	X	2	X	2		1-100+	Both
Ghana	2005	World Health Organization (2005). Study on Global Ageing and Adult Health (SAGE), Pilot Study, 2005 (Data Set 27-28, Cunningham, Shayna.) [machine-readable data file and documentation]. Geneva, Switzerland: World Health Organization (Producer). Los Altos, CA: Sociometrics Corporation, Data Archive of Social Research on Aging (Producer & Distributor).	X	52				All ages	Both
Ghana	2007	Ghana Health Service, Ministry of Health (Ghana), University of Ghana, World Health Organization (WHO). Ghana WHO Study on Global AGEing and Adult Health 2007-2008.	X	52				15-100+	Both
Ghana	2012	Ghana Statistical Service, World Bank. Ghana Living Standards Measurement Survey 2012-2013. Accra, Ghana: Ghana Statistical Service.	X	2	X	2		All ages	Both

Greece	1971, 1972, 1973, 1974, 1975, 1976, 1977, 1978, 1979, 1980, 1981, 1982, 1983, 1984, 1985, 1986, 1987, 1988, 1989, 1990, 1991, 1992, 1993, 1994, 1995, 1996, 1997, 1998, 1999, 2000, 2001, 2002, 2003, 2004, 2005, 2006, 2007, 2008, 2009, 2010, 2011, 2012	World Health Organization Regional Office for Europe (WHO/Europe). European Health for All Database - Inpatient Care Discharges Per 100. Copenhagen, Denmark: World Health Organization Regional Office for Europe (WHO/Europe).			X		X	All ages	Both
Greece	1999, 2000, 2001, 2002, 2003, 2004, 2005, 2006, 2007	World Health Organization Regional Office for Europe (WHO/Europe). European Health for All Database - Outpatient Contacts Per Person Per Year. Copenhagen, Denmark: World Health Organization Regional Office for Europe (WHO/Europe).	X				X	All ages	Both
Greece	2000	National School of Public Health (Greece), World Health Organization (WHO). Greece WHO Multi-country Survey Study on Health and Health System Responsiveness 2000-2001.	X	4.3	X	4.3		0-95	Both
Greece	2003	World Health Organization (WHO). Greece World Health Survey 2003. Geneva, Switzerland: World Health Organization (WHO), 2005.			X	52		15-100+	Both
Greece	2006	Börsch-Supan, A. (2013). Survey of Health, Ageing and Retirement in Europe (SHARE) Wave 1. Release version: 2.6.0. SHARE-ERIC. Data set. DOI: 10.6103/SHARE.w1.260	X	52	X	52		All ages	Both
Greece	2010	Börsch-Supan, A. (2013). Survey of Health, Ageing and Retirement in Europe (SHARE) Wave 2. Release version: 2.6.0. SHARE-ERIC. Data set. DOI: 10.6103/SHARE.w2.260	X	52	X	52		35-100+	Both
Guatemala	2003	World Health Organization (WHO). Guatemala World Health Survey 2003. Geneva, Switzerland: World Health Organization (WHO), 2005.			X	52		All ages	Both
Guatemala	2008	Guatemala Ministry of Health and Social Assistance, University of Valle and Division of Reproductive Health-Centers for Disease Control and Prevention (CDC). Guatemala Reproductive Health Survey 2008-2009. Atlanta, United States: Centers for Disease Control and Prevention (CDC).	X	4				0-50	Female
Guyana	1992	Bureau of Statistics (Guyana), World Bank. Guyana Living Standards Measurement Survey 1992-1993.	X	4.3	X	4.3		All ages	Both
Haiti	2001	Haitian Institute of Statistics and Informatics, The Fafo Research Foundation. Haiti Living Condition Survey 2001, Haiti: Haitian Institute of Statistics and Informatics.	X	2				15-100+	Both
Hungary	1991				X		X	All ages	Both
Hungary	1992				X		X	All ages	Both
Hungary	1993				X		X	All ages	Both
Hungary	1994				X		X	All ages	Both
Hungary	1995				X		X	All ages	Both
Hungary	1996				X		X	All ages	Both
Hungary	1997				X		X	All ages	Both

Hungary	1998				X		X	All ages	Both
Hungary	1999, 2000	World Health Organization Regional Office for Europe (WHO/Europe). European Health for All Database - Outpatient Contacts Per Person Per Year. Copenhagen, Denmark: World Health Organization Regional Office for Europe (WHO/Europe).	X				X	All ages	Both
Hungary	1999				X		X	All ages	Both
Hungary	2000	Health Promotion Research Institute (Hungary), Hungarian Gallup Institute. Hungary National Population Health Survey 2000.	X	52	X	52		15-100+	Both
Hungary	2000	Szonda Ipsos, World Health Organization (WHO). Hungary WHO Multi-country Survey Study on Health and Health System Responsiveness 2000-2001. Geneva, Switzerland: World Health Organization (WHO).	X	4.3	X	4.3		All ages	Both
Hungary	2000				X		X	All ages	Both
Hungary	2001, 2002, 2003, 2004, 2005, 2006, 2007, 2008, 2009, 2010, 2011, 2012, 2013, 2014, 2015	Organization for Economic Co-operation and Development (OECD). OECD Health Statistics. Paris, France: Organization for Economic Co-operation and Development (OECD).	X				X	All ages	Both
Hungary	2001				X		X	All ages	Both
Hungary	2002				X		X	All ages	Both
Hungary	2003, 2004, 2005, 2006, 2007, 2009, 2010, 2011	World Health Organization Regional Office for Europe (WHO/Europe). European Hospital Morbidity Database 1999-2007. Copenhagen, Denmark: World Health Organization Regional Office for Europe (WHO/Europe).			X		X	All ages	Both
Hungary	2003	World Health Organization (WHO). Hungary World Health Survey 2003. Geneva, Switzerland: World Health Organization (WHO), 2005.			X	52		15-100+	Both
Hungary	2003				X		X	All ages	Both
Hungary	2004				X		X	All ages	Both
Hungary	2012	Börsch-Supan, A. (2013). Survey of Health, Ageing and Retirement in Europe (SHARE) Wave 4. Release version: 1.1.1. SHARE-ERIC. Data set. DOI: 10.6103/SHARE.w4.111	X	52	X	52		25-100+	Both
Hungary	2014	National Institute for Strategic Health Research (Hungary). Hungary Hospital Inpatient Discharges 2012.			X		X	All ages	Both
Hungary	2015	National Institute for Strategic Health Research (Hungary). Hungary Hospital Inpatient Discharges 2012.			X		X	All ages	Both
Iceland	1988	Directorate of Health (Iceland). Iceland Hospital Data Registry 1988.			X		X	All ages	Both
Iceland	1989	Directorate of Health (Iceland). Iceland Hospital Data Registry 1989.			X		X	All ages	Both
Iceland	1990	Directorate of Health (Iceland). Iceland Hospital Data Registry 1990.			X		X	All ages	Both
Iceland	1991	Directorate of Health (Iceland). Iceland Hospital Data Registry 1991.			X		X	All ages	Both
Iceland	1992	Directorate of Health (Iceland). Iceland Hospital Data Registry 1992.			X		X	All ages	Both
Iceland	1993	Directorate of Health (Iceland). Iceland Hospital Data Registry 1993.			X		X	All ages	Both
Iceland	1994	Directorate of Health (Iceland). Iceland Hospital Data Registry 1994.			X		X	All ages	Both
Iceland	1995	Directorate of Health (Iceland). Iceland Hospital Data Registry 1995.			X		X	All ages	Both
Iceland	1998	Directorate of Health (Iceland). Iceland Hospital Data Registry 1998.	X				X	All ages	Both
Iceland	1999, 2001, 2002, 2003, 2004, 2005, 2006, 2007	World Health Organization Regional Office for Europe (WHO/Europe). European Hospital Morbidity Database 1999-2007. Copenhagen, Denmark: World Health Organization Regional Office for Europe (WHO/Europe).			X		X	All ages	Both
Iceland	1999	Directorate of Health (Iceland). Iceland Hospital Data Registry 1999.	X		X		X	All ages	Both
Iceland	2000	International Research Associates (INRA) Europe, World Health Organization (WHO). Iceland WHO Multi-country Survey Study on Health and Health System Responsiveness 2000-2001. Geneva, Switzerland: World Health Organization (WHO).	X	4.3	X	4.3		15-85	Both

Iceland	2001, 2002, 2003, 2004, 2005, 2006, 2007, 2008, 2009, 2010, 2011, 2012, 2013, 2014, 2015	Organization for Economic Co-operation and Development (OECD). OECD Health Statistics. Paris, France: Organization for Economic Co-operation and Development (OECD).	X				X	All ages	Both
Iceland	2008	Directorate of Health (Iceland). Iceland Hospital Data Registry 2008.			X		X	All ages	Both
Iceland	2010	Directorate of Health (Iceland). Iceland Hospital Data Registry 2010.			X		X	All ages	Both
Iceland	2011	Directorate of Health (Iceland). Iceland Hospital Data Registry 2011.			X		X	All ages	Both
Iceland	2012	Directorate of Health (Iceland). Iceland Hospital Data Registry 2012.			X		X	All ages	Both
Iceland	2013	Directorate of Health (Iceland). Iceland Hospital Data Registry 2013.			X		X	All ages	Both
Iceland	2014	Directorate of Health (Iceland). Iceland Hospital Data Registry 2014.			X		X	All ages	Both
India	1995	Ministry of Statistics and Programme Implementation (India). India National Sample Survey Round 52 1995-1996. New Delhi, India: Ministry of Statistics and Programme Implementation (India).	X	2.1	X	52		All ages	Both
India	1997	World Bank. India - Uttar Pradesh and Bihar Survey of Living Conditions 1997-1998. Washington DC, United States: World Bank.	X	4.3				All ages	Both
India	1998	International Institute for Population Sciences (IIPS) (India), Ministry of Health and Family Welfare (India). India District Level Household Survey 1998-1999 (DLHS). Mumbai, India: International Institute for Population Sciences (IIPS) (India).	X	12.9				0-45	Female
India	2000	Institute of Health Systems (India), World Health Organization (WHO). India - Andhra Pradesh WHO Multi-country Survey Study on Health and Health System Responsiveness 2000-2001.	X	4.3	X	4.3		All ages	Both
India	2003	International Institute for Population Sciences (India), World Health Organization (WHO). India World Health Survey 2003. Geneva, Switzerland: World Health Organization (WHO), 2005.			X	52		All ages	Both
India	2004	Ministry of Statistics and Programme Implementation (India). India National Sample Survey Round 60 2004. New Delhi, India: Ministry of Statistics and Programme Implementation (India).	X	2.1	X	52		All ages	Both
India	2005	World Health Organization (2005). Study on Global Ageing and Adult Health (SAGE), Pilot Study, 2005 (Data Set 27-28, Cunningham, Shayna.) [machine-readable data file and documentation]. Geneva, Switzerland: World Health Organization (Producer). Los Altos, CA: Sociometrics Corporation, Data Archive of Social Research on Aging (Producer & Distributor).	X	52				20-100+	Both
India	2007	International Institute for Population Sciences (India), World Health Organization (WHO). India WHO Study on Global Ageing and Adult Health 2007. Geneva, Switzerland: World Health Organization (WHO), 2007.	X	52	X	52		15-100+	Both
India	2014	Government of India, Ministry of Statistics and Programme Implementation (India). India National Sample Survey Round 71 2014. New Delhi, India: Ministry of Statistics and Programme Implementation (India).	X	2.1	X	52		All ages	Both
Indonesia	1992	Central Bureau of Statistics (Indonesia). Indonesia National Socioeconomic Survey 1992.	X	4.3	X	4.3		All ages	Both
Indonesia	1993	RAND Corporation, University of Indonesia. Indonesia Family Life Survey 1993-1994. Santa Monica, United States: RAND Corporation.	X	4	X	52		10-100+	Both
Indonesia	1993	Central Bureau of Statistics (Indonesia). Indonesia National Socioeconomic Survey 1993.	X	4.3	X	4.3		All ages	Both
Indonesia	1994	Central Bureau of Statistics (Indonesia). Indonesia National Socioeconomic Survey 1994.	X	4.3	X	4.3		All ages	Both
Indonesia	1995	Central Bureau of Statistics (Indonesia), Ministry of Health (Indonesia), United Nations Children's Fund (UNICEF). Indonesia National Socioeconomic Survey 1995.	X	4.3	X	4.3		All ages	Both
Indonesia	1996	Central Bureau of Statistics (Indonesia), Ministry of Health (Indonesia), United Nations Children's Fund (UNICEF). Indonesia National Socioeconomic Survey 1996.	X	4.3	X	4.3		All ages	Both
Indonesia	1997	Central Bureau of Statistics (Indonesia), Ministry of Health (Indonesia), United Nations Children's Fund (UNICEF). Indonesia National Socioeconomic Survey 1997.	X	4.3	X	4.3		All ages	Both
Indonesia	1998	Central Bureau of Statistics (Indonesia), Ministry of Health (Indonesia), World Bank. Indonesia National Socioeconomic Survey 1998.	X	4.3				All ages	Both
Indonesia	1999	Central Bureau of Statistics (Indonesia), Ministry of Health (Indonesia), World Bank. Indonesia National Socioeconomic Survey 1999.	X	4.3				All ages	Both
Indonesia	2000	Central Bureau of Statistics (Indonesia), Ministry of Health (Indonesia), World Bank. Indonesia National Socioeconomic Survey 2000.	X	4.3	X	4.3		All ages	Both

Indonesia	2000	Institute of Health Systems (India), World Health Organization (WHO). India - Andhra Pradesh WHO Multi-country Survey Study on Health and Health System Responsiveness 2000-2001.	X	4.3	X	4.3		All ages	Both
Indonesia	2001	Central Bureau of Statistics (Indonesia), Ministry of Health (Indonesia), World Bank. Indonesia National Socioeconomic Survey 2001.	X	4.3	X	4.3		All ages	Both
Indonesia	2002	Statistics Indonesia. Indonesia National Socioeconomic Survey 2002.	X	4.3	X	52		All ages	Both
Indonesia	2004	Statistics Indonesia. Indonesia National Socioeconomic Survey 2004.	X	4	X	52		All ages	Both
Indonesia	2005	Statistics Indonesia. Indonesia National Socioeconomic Survey 2005.	X	4	X	52		All ages	Both
Indonesia	2006	Statistics Indonesia. Indonesia National Socioeconomic Survey - Poverty Program Evaluation 2006. Jakarta, Indonesia: Statistics Indonesia.	X	4	X	52		All ages	Both
Indonesia	2007	Center for Population and Policy Studies, Gadjah Mada University (Indonesia), RAND Corporation, SurveyMETER. Indonesia Family Life Survey 2007-2008. Santa Monica, United States: RAND Corporation.	X	4				15-100+	Both
Indonesia	2007	National Institute of Health Research and Development (NIHRD), Ministry of Health (Indonesia). Indonesia Basic Health Research 2007-2008.	X	52				All ages	Both
Indonesia	2007	Family Health International, Ministry of Health (Indonesia), National AIDS Commission (KPA), Statistics Indonesia. Indonesia Behavioral Surveillance Survey 2007.	X	4	X	52		All ages	Both
Indonesia	2008	Statistics Indonesia. Indonesia National Socioeconomic Survey 2008.	X	4	X	52		All ages	Both
Indonesia	2008	Statistics Indonesia. Indonesia National Socioeconomic Survey - Poverty Program Evaluation 2008-2009. Jakarta, Indonesia: Statistics Indonesia.	X	4	X	52		All ages	Both
Indonesia	2010	Statistics Indonesia. Indonesia National Socioeconomic Survey 2010.	X	4	X	52		All ages	Both
Indonesia	2011	Statistics Indonesia. Indonesia National Socioeconomic Survey 2011.	X	4.3	X	52		All ages	Both
Iran	2000	Ministry of Health and Medical Education (Iran), World Health Organization (WHO). Iran WHO Multi-country Survey Study on Health and Health System Responsiveness 2000-2001. Geneva, Switzerland: World Health Organization (WHO).	X	4.3	X	4.3		All ages	Both
Iraq	2004	Central Organization for Statistics and Information Technology (Iraq), The Fafo Research Foundation. Iraq Multiple Indicator Rapid Assessment 2004.	X	2				All ages	Both
Iraq	2006	Central Organization for Statistics and Information Technology (Iraq), Kurdistan Regional Statistics Office, World Bank. Iraq Household Socioeconomic Survey 2006-2007. Washington DC, United States: World Bank.	X	4.3				All ages	Both
Ireland	1980	Department of Health (Ireland), Department of Health and Children (Ireland), Economic and Social Research Institute (ESRI) (Ireland). Ireland Hospital Inpatient Enquiry 1980.			X		X	All ages	Both
Ireland	1981	Department of Health (Ireland), Department of Health and Children (Ireland), Economic and Social Research Institute (ESRI) (Ireland). Ireland Hospital Inpatient Enquiry 1981.			X		X	All ages	Both
Ireland	1982	Department of Health (Ireland), Department of Health and Children (Ireland), Economic and Social Research Institute (ESRI) (Ireland). Ireland Hospital Inpatient Enquiry 1982.			X		X	All ages	Both
Ireland	1983	Department of Health and Children (Ireland), Economic and Social Research Institute (ESRI) (Ireland). Ireland Hospital Inpatient Enquiry 1983.			X		X	All ages	Both
Ireland	1984	Department of Health and Children (Ireland), Economic and Social Research Institute (ESRI) (Ireland). Ireland Hospital Inpatient Enquiry 1984.			X		X	All ages	Both
Ireland	1985	Department of Health and Children (Ireland), Economic and Social Research Institute (ESRI) (Ireland). Ireland Hospital Inpatient Enquiry 1985.			X		X	All ages	Both
Ireland	1986	Department of Health and Children (Ireland), Economic and Social Research Institute (ESRI) (Ireland). Ireland Hospital Inpatient Enquiry 1986.			X		X	All ages	Both
Ireland	1987	Department of Health and Children (Ireland), Economic and Social Research Institute (ESRI) (Ireland). Ireland Hospital Inpatient Enquiry 1987.			X		X	All ages	Both
Ireland	1988	Department of Health and Children (Ireland), Economic and Social Research Institute (ESRI) (Ireland). Ireland Hospital Inpatient Enquiry 1988.			X		X	All ages	Both
Ireland	1989	Department of Health and Children (Ireland), Economic and Social Research Institute (ESRI) (Ireland). Ireland Hospital Inpatient Enquiry 1989.			X		X	All ages	Both
Ireland	1990	Department of Health and Children (Ireland), Economic and Social Research Institute (ESRI) (Ireland). Ireland Hospital Inpatient Enquiry 1990.			X		X	All ages	Both
Ireland	1991	Department of Health and Children (Ireland), Economic and Social Research Institute (ESRI) (Ireland). Ireland Hospital Inpatient Enquiry 1991.			X		X	All ages	Both
Ireland	1992	Department of Health and Children (Ireland), Economic and Social Research Institute (ESRI) (Ireland). Ireland Hospital Inpatient Enquiry 1992.			X		X	All ages	Both
Ireland	1993	Department of Health and Children (Ireland), Economic and Social Research Institute (ESRI) (Ireland). Ireland Hospital Inpatient Enquiry 1993.			X		X	All ages	Both
Ireland	1994	Department of Health and Children (Ireland), Economic and Social Research Institute (ESRI) (Ireland). Ireland Hospital Inpatient Enquiry 1994.			X		X	All ages	Both

Ireland	1995	Department of Health and Children (Ireland), Economic and Social Research Institute (ESRI) (Ireland). Ireland Hospital Inpatient Enquiry 1995.			X		X	All ages	Both
Ireland	1996	Department of Health and Children (Ireland), Economic and Social Research Institute (ESRI) (Ireland). Ireland Hospital Inpatient Enquiry 1996.			X		X	All ages	Both
Ireland	1997	Department of Health and Children (Ireland), Economic and Social Research Institute (ESRI) (Ireland). Ireland Hospital Inpatient Enquiry 1997.			X		X	All ages	Both
Ireland	1998	Department of Health and Children (Ireland), Economic and Social Research Institute (ESRI) (Ireland). Ireland Hospital Inpatient Enquiry 1998.			X		X	All ages	Both
Ireland	1999, 2001, 2002, 2003, 2004, 2005, 2006, 2007, 2009, 2010, 2011	World Health Organization Regional Office for Europe (WHO/Europe). European Hospital Morbidity Database 1999-2007. Copenhagen, Denmark: World Health Organization Regional Office for Europe (WHO/Europe).			X		X	All ages	Both
Ireland	1999	Department of Health and Children (Ireland), Economic and Social Research Institute (ESRI) (Ireland). Ireland Hospital Inpatient Enquiry 1999.			X		X	All ages	Both
Ireland	2000	International Research Associates (INRA) Europe, World Health Organization (WHO). Ireland WHO Multi-country Survey Study on Health and Health System Responsiveness 2000-2001. Geneva, Switzerland: World Health Organization (WHO).	X	4.3	X	4.3		15-90	Both
Ireland	2000	Department of Health (Ireland), Economic and Social Research Institute (ESRI) (Ireland). Ireland Hospital Inpatient Enquiry 2000.			X		X	All ages	Both
Ireland	2002	Centre for Health Promotion Studies, National University of Ireland, Galway, Department of Public Health Medicine and Epidemiology, University College Dublin, Health Promotion Unit, Department of Health and Children (Ireland). Ireland Survey of Lifestyle Attitudes and Nutrition 2002. Dublin, Ireland: Health Promotion Unit, Department of Health and Children (Ireland).	X	52				0-90	Both
Ireland	2003	World Health Organization (WHO). Ireland World Health Survey 2003. Geneva, Switzerland: World Health Organization (WHO), 2005.			X	52		0-95	Both
Ireland	2007	Economic and Social Research Institute (ESRI) (Ireland), Health Promotion Unit, Department of Health and Children (Ireland), National University of Ireland, Galway, Royal College of Surgeons in Ireland (RCSI), University College Cork. Ireland Survey of Lifestyle Attitudes and Nutrition 2007. Dublin, Ireland: Health Promotion Unit, Department of Health and Children (Ireland).	X	52				All ages	Both
Ireland	2008, 2011, 2016	World Health Organization Regional Office for Europe (WHO/Europe). European Health for All Database - Outpatient Contacts Per Person Per Year. Copenhagen, Denmark: World Health Organization Regional Office for Europe (WHO/Europe).	X				X	All ages	Both
Ireland	2010	Börsch-Supan, A. (2013). Survey of Health, Ageing and Retirement in Europe (SHARE) Wave 2. Release version: 2.6.0. SHARE-ERIC. Data set. DOI: 10.6103/SHARE.w2.260	X	52	X	52		30-100+	Both
Ireland	2013	Department of Health and Children (Ireland), Economic and Social Research Institute (ESRI) (Ireland), Health Service Executive (HSE) (Ireland). Ireland Hospital Inpatient Enquiry 2013.			X		X	All ages	Both
Ireland	2014	Department of Health and Children (Ireland), Economic and Social Research Institute (ESRI) (Ireland), Health Service Executive (HSE) (Ireland). Ireland Hospital Inpatient Enquiry 2014.			X		X	All ages	Both
Israel	1999, 2001, 2002, 2003, 2004, 2005, 2006, 2007, 2009	World Health Organization Regional Office for Europe (WHO/Europe). European Hospital Morbidity Database 1999-2007. Copenhagen, Denmark: World Health Organization Regional Office for Europe (WHO/Europe).			X		X	All ages	Both
Israel	2001, 2010	Organization for Economic Co-operation and Development (OECD). OECD Health Statistics. Paris, France: Organization for Economic Co-operation and Development (OECD).	X				X	All ages	Both
Israel	2003	World Health Organization (WHO). Israel World Health Survey 2003. Geneva, Switzerland: World Health Organization (WHO), 2005.			X	52		0-95	Both
Israel	2006	Börsch-Supan, A. (2013). Survey of Health, Ageing and Retirement in Europe (SHARE) Wave 1. Release version: 2.6.0. SHARE-ERIC. Data set. DOI: 10.6103/SHARE.w1.260	X	52	X	52		0-95	Both
Israel	2010	Börsch-Supan, A. (2013). Survey of Health, Ageing and Retirement in Europe (SHARE) Wave 2. Release version: 2.6.0. SHARE-ERIC. Data set. DOI: 10.6103/SHARE.w2.260	X	52	X	52		35-100+	Both
Israel	2011	ISSP Research Group (2009): International Social Survey Programme: Health and Health Care - ISSP 2011. GESIS Data Archive, Cologne. ZA5800 Data file version 3.0.0, doi:10.4232/1.12252.	X	52				0-95	Both

Israel	2011	Ministry of Health (Israel). Israel National Hospital Discharge Database 2011.			X		X	All ages	Both
Israel	2012	Ministry of Health (Israel). Israel National Hospital Discharge Database 2012.			X		X	All ages	Both
Israel	2013	Börsch-Supan, A. (2015). Survey of Health, Ageing and Retirement in Europe (SHARE) Wave 5. Release version: 1.0.0. SHARE-ERIC. Data set. DOI: 10.6103/SHARE.w5.100	X	52	X	52		All ages	Both
Israel	2013	Ministry of Health (Israel). Israel National Hospital Discharge Database 2013.			X		X	All ages	Both
Israel	2014	Ministry of Health (Israel). Israel National Hospital Discharge Database 2014.			X		X	All ages	Both
Italy	1970	Ministry of Health (Italy). Italy National Hospital Discharge Database 1970.			X		X	All ages	Both
Italy	1971	Ministry of Health (Italy). Italy National Hospital Discharge Database 1971.			X		X	All ages	Both
Italy	1972	Ministry of Health (Italy). Italy National Hospital Discharge Database 1972.			X		X	All ages	Both
Italy	1973	Ministry of Health (Italy). Italy National Hospital Discharge Database 1973.			X		X	All ages	Both
Italy	1974	Ministry of Health (Italy). Italy National Hospital Discharge Database 1974.			X		X	All ages	Both
Italy	1975	Ministry of Health (Italy). Italy National Hospital Discharge Database 1975.			X		X	All ages	Both
Italy	1976	Ministry of Health (Italy). Italy National Hospital Discharge Database 1976.			X		X	All ages	Both
Italy	1977	Ministry of Health (Italy). Italy National Hospital Discharge Database 1977.			X		X	All ages	Both
Italy	1978	Ministry of Health (Italy). Italy National Hospital Discharge Database 1978.			X		X	All ages	Both
Italy	1979	Ministry of Health (Italy). Italy National Hospital Discharge Database 1979.			X		X	All ages	Both
Italy	1980	Ministry of Health (Italy). Italy National Hospital Discharge Database 1980.			X		X	All ages	Both
Italy	1981	Ministry of Health (Italy). Italy National Hospital Discharge Database 1981.			X		X	All ages	Both
Italy	1982	Ministry of Health (Italy). Italy National Hospital Discharge Database 1982.			X		X	All ages	Both
Italy	1983	Ministry of Health (Italy). Italy National Hospital Discharge Database 1983.			X		X	All ages	Both
Italy	1984	Ministry of Health (Italy). Italy National Hospital Discharge Database 1984.			X		X	All ages	Both
Italy	1985	Ministry of Health (Italy). Italy National Hospital Discharge Database 1985.			X		X	All ages	Both
Italy	1986	Ministry of Health (Italy). Italy National Hospital Discharge Database 1986.			X		X	All ages	Both
Italy	1987	Ministry of Health (Italy). Italy National Hospital Discharge Database 1987.			X		X	All ages	Both
Italy	1988	Ministry of Health (Italy). Italy National Hospital Discharge Database 1988.			X		X	All ages	Both
Italy	1989	Ministry of Health (Italy). Italy National Hospital Discharge Database 1989.			X		X	All ages	Both
Italy	1990	Ministry of Health (Italy). Italy National Hospital Discharge Database 1990.			X		X	All ages	Both
Italy	1991	Ministry of Health (Italy). Italy National Hospital Discharge Database 1991.			X		X	All ages	Both
Italy	1992	Ministry of Health (Italy). Italy National Hospital Discharge Database 1992.			X		X	All ages	Both
Italy	1993	Ministry of Health (Italy). Italy National Hospital Discharge Database 1993.			X		X	All ages	Both
Italy	1994	Ministry of Health (Italy). Italy National Hospital Discharge Database 1994.			X		X	All ages	Both
Italy	1995	Ministry of Health (Italy). Italy National Hospital Discharge Database 1995.			X		X	All ages	Both
Italy	1996	Ministry of Health (Italy). Italy National Hospital Discharge Database 1996.			X		X	All ages	Both
Italy	1997	Ministry of Health (Italy). Italy National Hospital Discharge Database 1997.			X		X	All ages	Both
Italy	1998	Ministry of Health (Italy). Italy National Hospital Discharge Database 1998.			X		X	All ages	Both
Italy	1999, 2001, 2002, 2003, 2004, 2005, 2006, 2007, 2009, 2010	World Health Organization Regional Office for Europe (WHO/Europe). European Hospital Morbidity Database 1999-2007. Copenhagen, Denmark: World Health Organization Regional Office for Europe (WHO/Europe).			X		X	All ages	Both
Italy	1999	Ministry of Health (Italy). Italy National Hospital Discharge Database 1999.			X		X	All ages	Both
Italy	2000	International Research Associates (INRA) Europe, World Health Organization (WHO). Italy WHO Multi-country Survey Study on Health and Health System Responsiveness 2000-2001. Geneva, Switzerland: World Health Organization (WHO).	X	4.3	X	4.3		15-95	Both
Italy	2000	Ministry of Health (Italy). Italy National Hospital Discharge Database 2000.			X		X	All ages	Both

Italy	2003	World Health Organization (WHO). Italy World Health Survey 2003. Geneva, Switzerland: World Health Organization (WHO), 2005.			X	52		All ages	Both
Italy	2006	Börsch-Supan, A. (2013). Survey of Health, Ageing and Retirement in Europe (SHARE) Wave 1. Release version: 2.6.0. SHARE-ERIC. Data set. DOI: 10.6103/SHARE.w1.260	X	52	X	52		All ages	Both
Italy	2010	Börsch-Supan, A. (2013). Survey of Health, Ageing and Retirement in Europe (SHARE) Wave 2. Release version: 2.6.0. SHARE-ERIC. Data set. DOI: 10.6103/SHARE.w2.260	X	52	X	52		35-100+	Both
Italy	2012	Börsch-Supan, A. (2013). Survey of Health, Ageing and Retirement in Europe (SHARE) Wave 4. Release version: 1.1.1. SHARE-ERIC. Data set. DOI: 10.6103/SHARE.w4.111	X	52	X	52		35-100+	Both
Italy	2012	Ministry of Health (Italy). Italy National Hospital Discharge Database 2012.			X		X	All ages	Both
Italy	2013	Börsch-Supan, A. (2015). Survey of Health, Ageing and Retirement in Europe (SHARE) Wave 5. Release version: 1.0.0. SHARE-ERIC. Data set. DOI: 10.6103/SHARE.w5.100	X	52	X	52		All ages	Both
Italy	2013	Ministry of Health (Italy). Italy National Hospital Discharge Database 2013.			X		X	All ages	Both
Italy	2014	Ministry of Health (Italy). Italy National Hospital Discharge Database 2014.			X		X	All ages	Both
Jamaica	1990	Statistical Institute of Jamaica. Jamaica Survey of Living Conditions 1990. Washington DC, United States: World Bank.	X	4	X	4		All ages	Both
Jamaica	1991	Planning Institute of Jamaica, Statistical Institute of Jamaica. Jamaica Survey of Living Conditions 1991.	X	4	X	4		All ages	Both
Jamaica	1992	Planning Institute of Jamaica, Statistical Institute of Jamaica. Jamaica Survey of Living Conditions 1992.	X	4	X	4		All ages	Both
Jamaica	1993	Planning Institute of Jamaica, Statistical Institute of Jamaica. Jamaica Survey of Living Conditions 1993.	X	4	X	4		All ages	Both
Jamaica	1994	Planning Institute of Jamaica, Statistical Institute of Jamaica. Jamaica Survey of Living Conditions 1994.	X	4	X	4		All ages	Both
Jamaica	1995	Planning Institute of Jamaica, Statistical Institute of Jamaica. Jamaica Survey of Living Conditions 1995.	X	4	X	4		0-70	Both
Jamaica	1996	Planning Institute of Jamaica, Statistical Institute of Jamaica. Jamaica Survey of Living Conditions 1996.	X	4	X	4		All ages	Both
Jamaica	1997	Planning Institute of Jamaica, Statistical Institute of Jamaica. Jamaica Survey of Living Conditions 1997.	X	4	X	4		All ages	Both
Jamaica	1998	Planning Institute of Jamaica, Statistical Institute of Jamaica. Jamaica Survey of Living Conditions 1988. Kingston, Jamaica: Planning Institute of Jamaica.	X	4	X	4		All ages	Both
Jamaica	1999	Planning Institute of Jamaica, Statistical Institute of Jamaica. Jamaica Survey of Living Conditions 1999.	X	4	X	4		All ages	Both
Jamaica	2000	Planning Institute of Jamaica, Statistical Institute of Jamaica. Jamaica Survey of Living Conditions 2000.	X	4	X	4		All ages	Both
Japan	1975, 1978, 1981, 1984, 1987, 1990, 1992, 1993, 1994, 1995, 1996, 1997, 1998, 1999, 2000, 2001, 2002, 2003, 2004, 2005, 2006, 2007, 2008, 2009, 2010, 2011, 2012, 2013, 2014	Organization for Economic Co-operation and Development (OECD). OECD Health Statistics. Paris, France: Organization for Economic Co-operation and Development (OECD).			X		X	All ages	Both
Jordan	2000	Gallup Europe, World Health Organization (WHO). Jordan WHO Multi-country Survey Study on Health and Health System Responsiveness 2000-2001. Geneva, Switzerland: World Health Organization (WHO).	X	4.3	X	4.3		15-90	Both
Jordan	2007	Centers for Disease Control and Prevention (CDC), Ministry of Health (Jordan), World Health Organization (WHO). Jordan STEPS Noncommunicable Disease Risk Factors Survey 2007.			X	52		15-100+	Both

Kazakhstan	1981, 1982, 1983, 1984, 1985, 1986, 1987, 1988, 1989, 1990, 1991, 1992, 1993, 1994, 1995, 1996, 1997, 1998, 1999, 2000, 2001, 2002, 2003, 2004, 2005, 2006, 2007, 2008, 2009, 2010, 2011, 2012, 2013, 2014, 2015, 2016	World Health Organization Regional Office for Europe (WHO/Europe). European Health for All Database - Inpatient Care Discharges Per 100. Copenhagen, Denmark: World Health Organization Regional Office for Europe (WHO/Europe).			X		X	All ages	Both
Kazakhstan	1996	Agency of the Republic of Kazakhstan on Statistics, World Bank. Kazakhstan Living Standards Measurement Survey 1996. Washington DC, United States: World Bank.			X	4.3		All ages	Both
Kazakhstan	1999, 2000, 2001, 2002, 2003, 2004, 2005, 2006, 2007, 2008, 2009, 2010, 2011, 2012, 2013, 2014, 2015, 2016	World Health Organization Regional Office for Europe (WHO/Europe). European Health for All Database - Outpatient Contacts Per Person Per Year. Copenhagen, Denmark: World Health Organization Regional Office for Europe (WHO/Europe).	X				X	All ages	Both
Kazakhstan	2002	World Health Organization (WHO). Kazakhstan World Health Survey 2002-2003. Geneva, Switzerland: World Health Organization (WHO), 2005.			X	52		0-90	Both
Kazakhstan	2010	Center for Study of Public Opinion (Kazakhstan), Concluzia-Prim Center for Survey Methodology (Moldova), Institute for Advanced Studies (Austria), London School of Hygiene and Tropical Medicine, University of Aberdeen. Kazakhstan Health in Times of Transition Household Survey 2010.			X	52		15-100+	Both
Kenya	1997	Central Bureau of Statistics (Kenya). Kenya Welfare Monitoring Survey III 1997. Nairobi, Kenya: Kenya National Bureau of Statistics.	X	4.3				All ages	Both
Kenya	2004	World Health Organization (WHO). Kenya World Health Survey 2004. Geneva, Switzerland: World Health Organization (WHO), 2005.			X	52		All ages	Both
Kenya	2007	Centers for Disease Control and Prevention (CDC), Kenya Medical Research Institute (KEMRI), Kenya National Bureau of Statistics, Ministry of Public Health and Sanitation (Kenya), National AIDS Control Council (Kenya), National AIDS and STI Control Program (Kenya), National Coordinating Agency for Population and Development (Kenya), National Public Health Laboratory Services, Ministry of Public Health and Sanitation (Kenya), USAID. Kenya AIDS Indicator Survey 2007. Nairobi, Kenya: Kenya National Bureau of Statistics.	X	4	X	26		All ages	Both
Kenya	2007	Abt Associates Inc., Kenya National Bureau of Statistics, Ministry of Health (Kenya). Kenya Household Health Expenditure and Utilization Survey 2007. Nairobi, Kenya: Kenya National Bureau of Statistics.	X	4	X	52		All ages	Both
Kenya	2009, 2010, 2011, 2012	Action Africa Help International (AAH-I), Institute for Health Metrics and Evaluation (IHME), Ministry of Medical Services (Kenya), Ministry of Public Health and Sanitation (Kenya). Access, Bottlenecks, Costs, and Equity (ABCE) project in Kenya, 2012. Seattle, United States: Institute for Health Metrics and Evaluation (IHME), 2015.			X		X	All ages	Both

Kenya	2012	Kenya National Bureau of Statistics, Ministry of Devolution and Planning (Kenya), Ministry of Health (Kenya), National AIDS and STI Control Program (Kenya). Kenya AIDS Indicator Survey 2012-2013. Nairobi, Kenya: Kenya National Bureau of Statistics.	X	52				10-65	Both
Kyrgyzstan	1981, 1982, 1983, 1984, 1985, 1986, 1987, 1988, 1989, 1990, 1991, 1992, 1993, 1994, 1995, 1996, 1997, 1998, 1999, 2000, 2001, 2002, 2003, 2004, 2005, 2006, 2007, 2008, 2009, 2010, 2011, 2012, 2013, 2014, 2015, 2016	World Health Organization Regional Office for Europe (WHO/Europe). European Health for All Database - Inpatient Care Discharges Per 100. Copenhagen, Denmark: World Health Organization Regional Office for Europe (WHO/Europe).			X		X	All ages	Both
Kyrgyzstan	1993	Institute of Sociology, Russian Academy of Sciences, Paragon Research, University of North Carolina, World Bank. Kyrgyzstan Living Standards Measurement Survey 1993. Washington DC, United States: World Bank.			X	4.3		All ages	Both
Kyrgyzstan	1997	National Statistical Committee of the Kyrgyz Republic, Research Triangle Institute, Inc. (RTI), World Bank. Kyrgyzstan Living Standards Measurement Survey 1997. Washington DC, United States: World Bank.			X	52		All ages	Both
Kyrgyzstan	1998	National Statistical Committee of the Kyrgyz Republic, Research Triangle Institute, Inc. (RTI), World Bank. Kyrgyzstan Living Standards Measurement Survey 1998. Washington DC, United States: World Bank.			X	52		All ages	Both
Kyrgyzstan	1999, 2000, 2001, 2002, 2003, 2004, 2005, 2006, 2007, 2008, 2009, 2010, 2011, 2012, 2013, 2014, 2015, 2016	World Health Organization Regional Office for Europe (WHO/Europe). European Health for All Database - Outpatient Contacts Per Person Per Year. Copenhagen, Denmark: World Health Organization Regional Office for Europe (WHO/Europe).	X				X	All ages	Both
Kyrgyzstan	2000	National Statistical Committee of the Kyrgyz Republic, SIAR Research and Consulting (Kyrgyzstan), World Health Organization (WHO). Kyrgyzstan WHO Multi-country Survey Study on Health and Health System Responsiveness 2000-2001. Geneva, Switzerland: World Health Organization (WHO).			X	4.3		0-95	Both
Kyrgyzstan	2010	Concluzia-Prim Center for Survey Methodology (Moldova), Institute for Advanced Studies (Austria), International Centre for Sociological, Political and Social Psychological Research (Kyrgyzstan), London School of Hygiene and Tropical Medicine, University of Aberdeen. Kyrgyzstan Health in Times of Transition Household Survey 2011.			X	52		15-90	Both
Laos	2003	World Health Organization (WHO). Laos World Health Survey 2003.			X	52		15-100+	Both
Latvia	1980	Ministry of Health of the Republic of Latvia, National Health Service (Latvia). Latvia Hospital Inpatient Discharges 1980.			X		X	All ages	Both
Latvia	1981	Ministry of Health of the Republic of Latvia, National Health Service (Latvia). Latvia Hospital Inpatient Discharges 1981.			X		X	All ages	Both
Latvia	1982	Ministry of Health of the Republic of Latvia, National Health Service (Latvia). Latvia Hospital Inpatient Discharges 1982.			X		X	All ages	Both
Latvia	1983	Ministry of Health of the Republic of Latvia, National Health Service (Latvia). Latvia Hospital Inpatient Discharges 1983.			X		X	All ages	Both

Latvia	1984	Ministry of Health of the Republic of Latvia, National Health Service (Latvia). Latvia Hospital Inpatient Discharges 1984.			X		X	All ages	Both
Latvia	1985	Ministry of Health of the Republic of Latvia, National Health Service (Latvia). Latvia Hospital Inpatient Discharges 1985.			X		X	All ages	Both
Latvia	1986	Ministry of Health of the Republic of Latvia, National Health Service (Latvia). Latvia Hospital Inpatient Discharges 1986.			X		X	All ages	Both
Latvia	1987	Ministry of Health of the Republic of Latvia, National Health Service (Latvia). Latvia Hospital Inpatient Discharges 1987.			X		X	All ages	Both
Latvia	1988	Ministry of Health of the Republic of Latvia, National Health Service (Latvia). Latvia Hospital Inpatient Discharges 1988.			X		X	All ages	Both
Latvia	1989	Ministry of Health of the Republic of Latvia, National Health Service (Latvia). Latvia Hospital Inpatient Discharges 1989.			X		X	All ages	Both
Latvia	1990	Ministry of Health of the Republic of Latvia, National Health Service (Latvia). Latvia Hospital Inpatient Discharges 1990.			X		X	All ages	Both
Latvia	1991	Ministry of Health of the Republic of Latvia, National Health Service (Latvia). Latvia Hospital Inpatient Discharges 1991.			X		X	All ages	Both
Latvia	1992	Ministry of Health of the Republic of Latvia, National Health Service (Latvia). Latvia Hospital Inpatient Discharges 1992.			X		X	All ages	Both
Latvia	1993	Ministry of Health of the Republic of Latvia, National Health Service (Latvia). Latvia Hospital Inpatient Discharges 1993.			X		X	All ages	Both
Latvia	1994	Ministry of Health of the Republic of Latvia, National Health Service (Latvia). Latvia Hospital Inpatient Discharges 1994.			X		X	All ages	Both
Latvia	1995	Ministry of Health of the Republic of Latvia, National Health Service (Latvia). Latvia Hospital Inpatient Discharges 1995.			X		X	All ages	Both
Latvia	1996	Ministry of Health of the Republic of Latvia, National Health Service (Latvia). Latvia Hospital Inpatient Discharges 1996.			X		X	All ages	Both
Latvia	1997	Ministry of Health of the Republic of Latvia, National Health Service (Latvia). Latvia Hospital Inpatient Discharges 1997.			X		X	All ages	Both
Latvia	1998	Ministry of Health of the Republic of Latvia, National Health Service (Latvia). Latvia Hospital Inpatient Discharges 1998.			X		X	All ages	Both
Latvia	1999, 2000	World Health Organization Regional Office for Europe (WHO/Europe). European Health for All Database - Outpatient Contacts Per Person Per Year. Copenhagen, Denmark: World Health Organization Regional Office for Europe (WHO/Europe).	X				X	All ages	Both
Latvia	1999	Ministry of Health of the Republic of Latvia, National Health Service (Latvia). Latvia Hospital Inpatient Discharges 1999.			X		X	All ages	Both
Latvia	2000	Gallup Europe, World Health Organization (WHO). Latvia WHO Multi-country Survey Study on Health and Health System Responsiveness 2000-2001. Geneva, Switzerland: World Health Organization (WHO).	X	4.3	X	4.3		15-100+	Both
Latvia	2000	Ministry of Health of the Republic of Latvia, National Health Service (Latvia). Latvia Hospital Inpatient Discharges 2000.			X		X	All ages	Both
Latvia	2001, 2002, 2003, 2004, 2005, 2006, 2007, 2008, 2009, 2010, 2011, 2012, 2013, 2014, 2015	Organization for Economic Co-operation and Development (OECD). OECD Health Statistics. Paris, France: Organization for Economic Co-operation and Development (OECD).	X				X	All ages	Both
Latvia	2001	Ministry of Health of the Republic of Latvia, National Health Service (Latvia). Latvia Hospital Inpatient Discharges 2001.			X		X	All ages	Both
Latvia	2002	Ministry of Health of the Republic of Latvia, National Health Service (Latvia). Latvia Hospital Inpatient Discharges 2002.			X		X	All ages	Both
Latvia	2003	World Health Organization (WHO). Latvia World Health Survey 2003. Geneva, Switzerland: World Health Organization (WHO), 2005.			X	52		0-90	Both
Latvia	2003	Ministry of Health of the Republic of Latvia, National Health Service (Latvia). Latvia Hospital Inpatient Discharges 2003.			X		X	All ages	Both
Latvia	2004, 2007	World Health Organization Regional Office for Europe (WHO/Europe). European Hospital Morbidity Database 1999-2007. Copenhagen, Denmark: World Health Organization Regional Office for Europe (WHO/Europe).			X		X	All ages	Both
Latvia	2004	Ministry of Health of the Republic of Latvia, National Health Service (Latvia). Latvia Hospital Inpatient Discharges 2004.			X		X	All ages	Both
Latvia	2006	Ministry of Health of the Republic of Latvia, National Health Service (Latvia). Latvia Hospital Inpatient Discharges 2006.			X		X	All ages	Both
Latvia	2007	Ministry of Health of the Republic of Latvia, National Health Service (Latvia). Latvia Hospital Inpatient Discharges 2007.			X		X	All ages	Both
Latvia	2008	Ministry of Health of the Republic of Latvia, National Health Service (Latvia). Latvia Hospital Inpatient Discharges 2008.			X		X	All ages	Both
Latvia	2010	Ministry of Health of the Republic of Latvia, National Health Service (Latvia). Latvia Hospital Inpatient Discharges 2010.			X		X	All ages	Both
Latvia	2011	Ministry of Health of the Republic of Latvia, National Health Service (Latvia). Latvia Hospital Inpatient Discharges 2011.			X		X	All ages	Both
Latvia	2012	Ministry of Health of the Republic of Latvia, National Health Service (Latvia). Latvia Hospital Inpatient Discharges 2012.			X		X	All ages	Both
Latvia	2013	Ministry of Health of the Republic of Latvia, National Health Service (Latvia). Latvia Hospital Inpatient Discharges 2013.			X		X	All ages	Both

Latvia	2014	Centre for Disease Prevention and Control (Latvia), Riga Stradiņš University. Latvia Health Behavior Among the Adult Population 2014.	X	52	X	52		15-64	Both
Latvia	2014	Ministry of Health of the Republic of Latvia, National Health Service (Latvia). Latvia Hospital Inpatient Discharges 2014.			X		X	All ages	Both
Lebanon	2000	Ministry of Public Health (Lebanon), World Health Organization (WHO). Lebanon WHO Multi-country Survey Study on Health and Health System Responsiveness 2000-2001.	X	4.3	X	4.3		All ages	Both
Lithuania	1971, 1972, 1973, 1974, 1975, 1976, 1977, 1978, 1979, 1980, 1981, 1982, 1983, 1984, 1985, 1986, 1987, 1988, 1989, 1990, 1991, 1992, 1993, 1994, 1995, 1996, 1997, 1998, 1999, 2000, 2001, 2002, 2003, 2004, 2005, 2006, 2007, 2008, 2009, 2010, 2011, 2012, 2013, 2014, 2015	Organization for Economic Co-operation and Development (OECD). OECD Health Statistics. Paris, France: Organization for Economic Co-operation and Development (OECD).			X		X	All ages	Both
Lithuania	1998	Health Information Centre, Ministry of Health (Lithuania). Lithuania Annual Report Data for Outpatient Contacts Per Person Per Year 1998.	X				X	All ages	Both
Lithuania	1999, 2001, 2002, 2003, 2004, 2005, 2006, 2007, 2009, 2010, 2011	World Health Organization Regional Office for Europe (WHO/Europe). European Hospital Morbidity Database 1999-2007. Copenhagen, Denmark: World Health Organization Regional Office for Europe (WHO/Europe).			X		X	All ages	Both
Lithuania	1999	Health Information Centre, Ministry of Health (Lithuania). Lithuania Annual Report Data for Outpatient Contacts Per Person Per Year 1999.	X				X	All ages	Both
Lithuania	2000	Biomedical Engineering Institute, Kaunas University of Technology, Statistics Lithuania, World Health Organization (WHO). Lithuania WHO Multi-country Survey Study on Health and Health System Responsiveness 2000-2001. Geneva, Switzerland: World Health Organization (WHO).	X	4.3	X	4.3		All ages	Both
Lithuania	2011	ISSP Research Group (2009): International Social Survey Programme: Health and Health Care - ISSP 2011. GESIS Data Archive, Cologne. ZA5800 Data file version 3.0.0, doi:10.4232/1.12252.	X	52				0-90	Both
Luxembourg	2000	International Research Associates (INRA) Europe, World Health Organization (WHO). Luxembourg WHO Multi-country Survey Study on Health and Health System Responsiveness 2000-2001. Geneva, Switzerland: World Health Organization (WHO).	X	4.3	X	4.3		15-85	Both
Luxembourg	2001, 2002, 2003, 2004, 2005, 2006, 2007, 2009,	World Health Organization Regional Office for Europe (WHO/Europe). European Hospital Morbidity Database 1999-2007. Copenhagen, Denmark: World Health Organization Regional Office for Europe (WHO/Europe).			X		X	All ages	Both

	2010, 2011								
Luxembourg	2003	World Health Organization (WHO). Luxembourg World Health Survey 2003. Geneva, Switzerland: World Health Organization (WHO), 2005.			X	52		15-95	Both
Luxembourg	2004, 2005, 2006, 2007, 2008, 2009, 2010, 2011, 2012, 2013, 2014, 2015	Organization for Economic Co-operation and Development (OECD). OECD Health Statistics. Paris, France: Organization for Economic Co-operation and Development (OECD).	X				X	All ages	Both
Luxembourg	2013	Börsch-Supan, A. (2015). Survey of Health, Ageing and Retirement in Europe (SHARE) Wave 5. Release version: 1.0.0. SHARE-ERIC. Data set. DOI: 10.6103/SHARE.w5.100	X	52	X	52		All ages	Both
Luxembourg	2014, 2015	World Health Organization Regional Office for Europe (WHO/Europe). European Health for All Database - Inpatient Care Discharges Per 100. Copenhagen, Denmark: World Health Organization Regional Office for Europe (WHO/Europe).			X		X	All ages	Both
Macedonia	1980	Institute of Public Health (Macedonia). Macedonia Hospital Inpatient Discharges 1980.			X		X	All ages	Both
Macedonia	1982	Institute of Public Health (Macedonia). Macedonia Hospital Inpatient Discharges 1982.			X		X	All ages	Both
Macedonia	1984	Institute of Public Health (Macedonia). Macedonia Hospital Inpatient Discharges 1984.			X		X	All ages	Both
Macedonia	1985	Institute of Public Health (Macedonia). Macedonia Hospital Inpatient Discharges 1985.			X		X	All ages	Both
Macedonia	1986	Institute of Public Health (Macedonia). Macedonia Hospital Inpatient Discharges 1986.			X		X	All ages	Both
Macedonia	1987	Institute of Public Health (Macedonia). Macedonia Hospital Inpatient Discharges 1987.			X		X	All ages	Both
Macedonia	1989	Institute of Public Health (Macedonia). Macedonia Hospital Inpatient Discharges 1989.			X		X	All ages	Both
Macedonia	1990	Institute of Public Health (Macedonia). Macedonia Hospital Inpatient Discharges 1990.			X		X	All ages	Both
Macedonia	1991	Institute of Public Health (Macedonia). Macedonia Hospital Inpatient Discharges 1991.			X		X	All ages	Both
Macedonia	1992	Institute of Public Health (Macedonia). Macedonia Hospital Inpatient Discharges 1992.			X		X	All ages	Both
Macedonia	1993	Institute of Public Health (Macedonia). Macedonia Hospital Inpatient Discharges 1993.			X		X	All ages	Both
Macedonia	1995	Institute of Public Health (Macedonia). Macedonia Hospital Inpatient Discharges 1995.			X		X	All ages	Both
Macedonia	1996	Institute of Public Health (Macedonia). Macedonia Hospital Inpatient Discharges 1996.			X		X	All ages	Both
Macedonia	1997	Institute of Public Health (Macedonia). Macedonia Hospital Inpatient Discharges 1997.			X		X	All ages	Both
Macedonia	1999, 2000, 2001, 2002, 2003, 2004, 2005, 2006, 2007, 2011, 2012, 2013, 2014	World Health Organization Regional Office for Europe (WHO/Europe). European Health for All Database - Outpatient Contacts Per Person Per Year. Copenhagen, Denmark: World Health Organization Regional Office for Europe (WHO/Europe).	X				X	All ages	Both
Macedonia	2000	Centers for Public Health (Macedonia). Macedonia Hospital Inpatient Discharges 2000.			X		X	All ages	Both
Macedonia	2001	Centers for Public Health (Macedonia). Macedonia Hospital Inpatient Discharges 2001.			X		X	All ages	Both
Macedonia	2002	Centers for Public Health (Macedonia). Macedonia Hospital Inpatient Discharges 2002.			X		X	All ages	Both
Macedonia	2004	Centers for Public Health (Macedonia). Macedonia Hospital Inpatient Discharges 2004.			X		X	All ages	Both
Macedonia	2012	Institute of Public Health (Macedonia). Macedonia Hospital Inpatient Discharges 2012.			X		X	All ages	Both
Malawi	2003	World Health Organization (WHO). Malawi World Health Survey 2003. Geneva, Switzerland: World Health Organization (WHO), 2005.			X	52		All ages	Both
Malawi	2004	National Statistical Office of Malawi, World Bank. Malawi Living Standards Measurement Survey 2004-2005.	X	2	X	52		All ages	Both
Malawi	2010	National Statistical Office of Malawi, World Bank. Malawi Living Standards Measurement Survey 2010-2011. Washington DC, United States: World Bank.	X	2	X	52		All ages	Both

Malawi	2013	National Statistical Office of Malawi. Malawi Living Standards Measurement Survey 2013. Washington DC, United States: World Bank, 2015.			X	52		All ages	Both
Malaysia	2003	World Health Organization (WHO). Malaysia World Health Survey 2003. Geneva, Switzerland: World Health Organization (WHO), 2005.			X	52		All ages	Both
Malaysia	2006	Institute for Public Health, Ministry of Health (Malaysia). Malaysia National Health And Morbidity Survey 2006. Kuala Lumpur, Malaysia: Institute for Public Health, Ministry of Health (Malaysia).			X	52		All ages	Both
Malaysia	2011	Institute for Public Health, Ministry of Health (Malaysia). Malaysia National Health and Morbidity Survey 2011.	X	52	X	52		All ages	Both
Mali	2003	World Health Organization (WHO). Mali World Health Survey 2003. Geneva, Switzerland: World Health Organization (WHO), 2005.			X	52		All ages	Both
Mali	2014	Ministry of Rural Development (Mali), National Institute of Statistics (INSTAT) (Mali), World Bank. Mali Living Standards Measurement Study - Integrated Survey on Agriculture 2014-2015. Washington DC, United States: World Bank.	X	4.3	X	52		All ages	Both
Malta	2000	International Research Associates (INRA) Europe, World Health Organization (WHO). Malta WHO Multi-country Survey Study on Health and Health System Responsiveness 2000-2001. Geneva, Switzerland: World Health Organization (WHO).	X	4.3	X	4.3		15-95	Both
Malta	2005, 2006, 2007, 2009, 2011	World Health Organization Regional Office for Europe (WHO/Europe). European Hospital Morbidity Database 1999-2007. Copenhagen, Denmark: World Health Organization Regional Office for Europe (WHO/Europe).			X		X	All ages	Both
Mauritania	2003	World Health Organization (WHO). Mauritania World Health Survey 2003. Geneva, Switzerland: World Health Organization (WHO), 2005.			X	52		All ages	Both
Mauritius	2003	World Health Organization (WHO). Mauritius World Health Survey 2003. Geneva, Switzerland: World Health Organization (WHO), 2005.			X	52		15-95	Both
Mexico	1995, 1996, 1997, 1998, 1999, 2000, 2001, 2002, 2003, 2004, 2005, 2006, 2007, 2008, 2009, 2010, 2011, 2012, 2013, 2014, 2015	Organization for Economic Co-operation and Development (OECD). OECD Health Statistics. Paris, France: Organization for Economic Co-operation and Development (OECD).			X		X	All ages	Both
Mexico	1999	National Institute of Public Health (Mexico). Mexico National Health Survey 1999-2000.	X	52	X	52		All ages	Both
Mexico	1999	Pan American Health Organization (PAHO), Center for Demography and Ecology, University of Wisconsin-Madison, Inter-University Consortium for Political and Social Research (ICPSR), College of the Northern Border (COLEF), Research in Health and Demographics (INSAD), National Institute of Medical Sciences and Nutrition Salvador Zubirán. Mexico - Mexico City Survey on Health, Well-Being, and Aging in Latin America and the Caribbean 1999-2000. Ann Arbor, United States: Inter-University Consortium for Political and Social Research (ICPSR).	X	17.2	X	26		50-100+	Both
Mexico	2000	National Institute of Public Health (Mexico), World Health Organization (WHO). Mexico WHO Multi-country Survey Study on Health and Health System Responsiveness 2000-2001. Geneva, Switzerland: World Health Organization (WHO).	X	4.3	X	4.3		All ages	Both
Mexico	2002	Center for Research and Teaching in Economics (CIDE) (Mexico), National Institute of Perinatology (Mexico), National Institute of Statistics and Geography (INEGI) (Mexico), Universidad Iberoamericana. Mexico Family Life Survey 2002.	X	4	X	4		All ages	Male
Mexico	2002	World Health Organization (WHO). Mexico World Health Survey 2002-2003. Geneva, Switzerland: World Health Organization (WHO), 2005.			X	52		All ages	Both
Mexico	2005	National Institute of Public Health (Mexico). Mexico National Survey of Health and Nutrition 2005-2006. Cuernavaca, Mexico: National Institute of Public Health (Mexico).	X	2	X	52		All ages	Both
Mexico	2005	California Center for Population Research (CCPR), University of California Los Angeles (UCLA), Center for Research and Teaching in Economics (CIDE) (Mexico), National Institute of Public Health (Mexico), Universidad Iberoamericana. Mexico Family Life Survey 2005-2006.	X	4	X	52		All ages	Both
Mexico	2008	Center for Research and Teaching in Economics (CIDE) (Mexico), Duke University, National Institute of Public Health (Mexico), Universidad Iberoamericana, University of California, Los Angeles (UCLA). Mexico Family Life Survey 2008-2013.	X	4	X	52		All ages	Both

Mexico	2008	Field Operatives Corporation (Mexico), Ministry of Health (Mexico), National Institute of Public Health (Mexico). Mexico National Survey of Health Spending 2008. Mexico City, Mexico: Ministry of Health (Mexico).	X	12.9				All ages	Both
Mexico	2009	National Institute of Public Health (Mexico), World Health Organization (WHO). Mexico WHO Study on Global AGEing and Adult Health 2009-2010. Geneva, Switzerland: World Health Organization (WHO), 2011.	X	52				All ages	Both
Mexico	2010	National Institute of Statistics and Geography (INEGI) (Mexico). Mexico Household Income and Expenditure Survey 2010. Mexico City, Mexico: National Institute of Statistics and Geography (INEGI) (Mexico).	X	12.9				All ages	Both
Mexico	2011	National Institute of Public Health (Mexico). Mexico National Survey of Health and Nutrition 2011-2012. Cuernavaca, Mexico: National Institute of Public Health (Mexico).	X	2	X	52		All ages	Both
Mexico	2012	National Institute of Statistics, Geography, and Informatics (Mexico), Population Studies Center, University of Pennsylvania, University of Maryland, University of Wisconsin. Mexico Health and Aging Study 2012. Mexico City, México: National Institute of Statistics, Geography, and Informatics (Mexico).	X	52				35-100+	Both
Moldova	1981, 1982, 1983, 1984, 1985, 1986, 1987, 1988, 1989, 1990, 1991, 1992, 1993, 1994, 1995, 1996, 1997, 1998, 1999, 2000, 2001, 2002, 2003, 2004, 2005, 2006, 2007, 2008, 2009, 2010, 2011, 2012, 2013, 2014, 2015	World Health Organization Regional Office for Europe (WHO/Europe). European Health for All Database - Inpatient Care Discharges Per 100. Copenhagen, Denmark: World Health Organization Regional Office for Europe (WHO/Europe).			X		X	All ages	Both
Moldova	1999, 2000, 2001, 2002, 2003, 2004, 2005, 2006, 2007, 2008, 2009, 2010, 2011, 2012, 2013, 2014, 2015	World Health Organization Regional Office for Europe (WHO/Europe). European Health for All Database - Outpatient Contacts Per Person Per Year. Copenhagen, Denmark: World Health Organization Regional Office for Europe (WHO/Europe).	X				X	All ages	Both
Moldova	2010	Concluzia-Prim Center for Survey Methodology (Moldova), Independent Sociology and Information Service (OPINIA) (Moldova), Institute for Advanced Studies (Austria), London School of Hygiene and Tropical Medicine, University of Aberdeen. Moldova Health in Times of Transition Household Survey 2010.			X	52		15-95	Both
Montenegro	2006, 2007, 2008, 2009, 2010, 2011, 2012, 2013, 2014, 2015	World Health Organization Regional Office for Europe (WHO/Europe). European Health for All Database - Inpatient Care Discharges Per 100. Copenhagen, Denmark: World Health Organization Regional Office for Europe (WHO/Europe).			X		X	All ages	Both
Montenegro	2015	World Health Organization Regional Office for Europe (WHO/Europe). European Health for All Database - Outpatient Contacts Per Person Per Year. Copenhagen, Denmark: World Health Organization Regional Office for Europe (WHO/Europe).	X				X	All ages	Both

Morocco	1990	Directorate of Statistics of the High Commission for Planning (Morocco), World Bank. Morocco Living Standards Measurement Survey 1990-1991.	X	4	X	52		All ages	Both
Morocco	2000	Gallup Europe, World Health Organization (WHO). Morocco WHO Multi-country Survey Study on Health and Health System Responsiveness 2000-2001. Geneva, Switzerland: World Health Organization (WHO).	X	4.3	X	4.3		15-90	Both
Morocco	2003	World Health Organization (WHO). Morocco World Health Survey 2003. Geneva, Switzerland: World Health Organization (WHO), 2005.			X	52		15-100+	Both
Myanmar	2003	World Health Organization (WHO). Myanmar World Health Survey 2003. Geneva, Switzerland: World Health Organization (WHO), 2005.			X	52		15-100+	Both
Namibia	2003	World Health Organization (WHO). Namibia World Health Survey 2003. Geneva, Switzerland: World Health Organization (WHO), 2005.			X	52		All ages	Both
Nepal	2003	World Health Organization (WHO). Nepal World Health Survey 2003. Geneva, Switzerland: World Health Organization (WHO), 2005.			X	52		15-100+	Both
Nepal	2003	Central Bureau of Statistics (Nepal), World Bank. Nepal Living Standards Measurement Survey 2003-2004. Kathmandu, Nepal: Central Bureau of Statistics (Nepal).	X	4.3				All ages	Both
Netherlands	1990	Statistics Netherlands. Netherlands National Medical Registry 1990.			X		X	All ages	Both
Netherlands	1991	Statistics Netherlands. Netherlands National Medical Registry 1991.			X		X	All ages	Both
Netherlands	1992	Statistics Netherlands. Netherlands National Medical Registry 1992.			X		X	All ages	Both
Netherlands	1993	Statistics Netherlands. Netherlands National Medical Registry 1993.			X		X	All ages	Both
Netherlands	1994	Statistics Netherlands. Netherlands National Medical Registry 1994.			X		X	All ages	Both
Netherlands	1995	Statistics Netherlands. Netherlands National Medical Registry 1995.			X		X	All ages	Both
Netherlands	1996	Statistics Netherlands. Netherlands National Medical Registry 1996.			X		X	All ages	Both
Netherlands	1997	Statistics Netherlands. Netherlands National Medical Registry 1997.			X		X	All ages	Both
Netherlands	1998	Dutch Hospital Data. Netherlands National Medical Registry 1998.			X		X	All ages	Both
Netherlands	1999	Dutch Hospital Data. Netherlands National Medical Registry 1999.			X		X	All ages	Both
Netherlands	2000	Dutch Hospital Data. Netherlands National Medical Registry 2000.			X		X	All ages	Both
Netherlands	2000	International Research Associates (INRA) Europe, Netherlands Organisation for Applied Scientific Research (TNO), World Health Organization (WHO). Netherlands WHO Multi-country Survey Study on Health and Health System Responsiveness 2000-2001. Geneva, Switzerland: World Health Organization (WHO).	X	4.3	X	4.3		15-95	Both
Netherlands	2001	Dutch Hospital Data. Netherlands National Medical Registry 2001.			X		X	All ages	Both
Netherlands	2002	Dutch Hospital Data. Netherlands National Medical Registry 2002.			X		X	All ages	Both
Netherlands	2002	Statistics Netherlands. Netherlands Permanent Quality of Life Survey 2002.	X	2				0-90	Both
Netherlands	2003	Dutch Hospital Data. Netherlands National Medical Registry 2003.			X		X	All ages	Both
Netherlands	2004, 2007, 2010, 2011	World Health Organization Regional Office for Europe (WHO/Europe). European Hospital Morbidity Database 1999-2007. Copenhagen, Denmark: World Health Organization Regional Office for Europe (WHO/Europe).			X		X	All ages	Both
Netherlands	2004	World Health Organization (WHO). Netherlands World Health Survey 2004. Geneva, Switzerland: World Health Organization (WHO), 2005.			X	52		15-85	Both
Netherlands	2004	Dutch Hospital Data. Netherlands National Medical Registry 2004.			X		X	All ages	Both
Netherlands	2004	Statistics Netherlands. Netherlands Permanent Quality of Life Survey 2004.	X	2				0-90	Both
Netherlands	2006	Börsch-Supan, A. (2013). Survey of Health, Ageing and Retirement in Europe (SHARE) Wave 1. Release version: 2.6.0. SHARE-ERIC. Data set. DOI: 10.6103/SHARE.w1.260	X	52	X	52		All ages	Both
Netherlands	2006	Dutch Hospital Data. Netherlands National Medical Registry 2006.			X		X	All ages	Both
Netherlands	2007	CentERdata. Netherlands Dutch National Bank Household Survey 2007. Tillburg, Netherlands: CentERdata.	X	26				15-95	Both
Netherlands	2007	Dutch Hospital Data. Netherlands National Medical Registry 2007.			X		X	All ages	Both
Netherlands	2008	CentERdata. Netherlands Dutch National Bank Household Survey 2008. Tillburg, Netherlands: CentERdata.	X	26				15-95	Both
Netherlands	2008	Statistics Netherlands. Netherlands Permanent Quality of Life Survey 2008.	X	2				0-90	Both
Netherlands	2009	CentERdata. Netherlands Dutch National Bank Household Survey 2009. Tillburg, Netherlands: CentERdata.	X	26				15-95	Both
Netherlands	2010	Börsch-Supan, A. (2013). Survey of Health, Ageing and Retirement in Europe (SHARE) Wave 2. Release version: 2.6.0. SHARE-ERIC. Data set. DOI: 10.6103/SHARE.w2.260	X	52	X	52		25-100+	Both
Netherlands	2010	Dutch Hospital Data. Netherlands National Medical Registry 2010.			X		X	All ages	Both

Netherlands	2010	CentERdata. Netherlands Dutch National Bank Household Survey 2010. Tilburg, Netherlands: CentERdata.	X	26				0-90	Both
Netherlands	2011	ISSP Research Group (2009): International Social Survey Programme: Health and Health Care - ISSP 2011. GESIS Data Archive, Cologne. ZA5800 Data file version 3.0.0, doi:10.4232/1.12252.	X	52				15-100+	Both
Netherlands	2011	CentERdata. Netherlands Dutch National Bank Household Survey 2011. Tilburg, Netherlands: CentERdata.	X	26				0-90	Both
Netherlands	2012	Börsch-Supan, A. (2013). Survey of Health, Ageing and Retirement in Europe (SHARE) Wave 4. Release version: 1.1.1. SHARE-ERIC. Data set. DOI: 10.6103/SHARE.w4.111	X	52	X	52		35-100+	Both
Netherlands	2012	CentERdata. Netherlands Dutch National Bank Household Survey 2012. Tilburg, Netherlands: CentERdata.	X	26				0-95	Both
Netherlands	2013	CentERdata. Netherlands Dutch National Bank Household Survey 2013. Tilburg, Netherlands: CentERdata.	X	26				0-95	Both
Netherlands	2013	Börsch-Supan, A. (2015). Survey of Health, Ageing and Retirement in Europe (SHARE) Wave 5. Release version: 1.0.0. SHARE-ERIC. Data set. DOI: 10.6103/SHARE.w5.100	X	52	X	52		All ages	Both
Netherlands	2014	CentERdata. Netherlands Dutch National Bank Household Survey 2014.	X	26				0-95	Both
New Zealand	2000	University of Otago (New Zealand), World Health Organization (WHO). New Zealand WHO Multi-country Survey Study on Health and Health System Responsiveness 2000-2001. Geneva, Switzerland: World Health Organization (WHO).	X	4.3	X	4.3		All ages	Both
New Zealand	2000	Ministry of Health (New Zealand). New Zealand National Minimum Dataset 2000.			X		X	0-99	Both
New Zealand	2001	Ministry of Health (New Zealand). New Zealand National Minimum Dataset 2001.			X		X	0-99	Both
New Zealand	2002	Ministry of Health (New Zealand). New Zealand National Minimum Dataset 2002.			X		X	0-99	Both
New Zealand	2003	Ministry of Health (New Zealand). New Zealand National Minimum Dataset 2003.			X		X	0-99	Both
New Zealand	2004, 2008, 2013	Organization for Economic Co-operation and Development (OECD). OECD Health Statistics. Paris, France: Organization for Economic Co-operation and Development (OECD).	X				X	All ages	Both
New Zealand	2004	Ministry of Health (New Zealand). New Zealand National Minimum Dataset 2004.			X		X	0-99	Both
New Zealand	2005	Ministry of Health (New Zealand). New Zealand National Minimum Dataset 2005.			X		X	0-99	Both
New Zealand	2006	Ministry of Health (New Zealand). New Zealand National Minimum Dataset 2006.			X		X	0-99	Both
New Zealand	2007	Ministry of Health (New Zealand). New Zealand National Minimum Dataset 2007.			X		X	0-99	Both
New Zealand	2008	Ministry of Health (New Zealand). New Zealand National Minimum Dataset 2008.			X		X	0-99	Both
New Zealand	2009	Ministry of Health (New Zealand). New Zealand National Minimum Dataset 2009.			X		X	0-99	Both
New Zealand	2010	Ministry of Health (New Zealand). New Zealand National Minimum Dataset 2010.			X		X	0-99	Both
New Zealand	2011	Ministry of Health (New Zealand). New Zealand National Minimum Dataset 2011.			X		X	0-99	Both
New Zealand	2012	Ministry of Health (New Zealand). New Zealand National Minimum Dataset 2012.			X		X	0-99	Both
New Zealand	2013	Ministry of Health (New Zealand). New Zealand National Minimum Dataset 2013.			X		X	0-99	Both
New Zealand	2014	Ministry of Health (New Zealand). New Zealand National Minimum Dataset 2014.			X		X	0-99	Both
New Zealand	2015	Ministry of Health (New Zealand). New Zealand National Minimum Dataset 2015.			X		X	0-99	Both
Nicaragua	1993	National Institute of Statistics and Censuses (Nicaragua), World Bank. Nicaragua Living Standards Measurement Survey 1993.	X	4.3	X	4.3		All ages	Both
Nicaragua	1998	National Institute of Statistics and Censuses (Nicaragua), World Bank. Nicaragua Living Standards Measurement Survey 1998-1999.	X	4	X	4		All ages	Both
Nicaragua	2001	National Institute of Statistics and Censuses (Nicaragua), World Bank. Nicaragua Living Standards Measurement Survey 2001.	X	4				All ages	Both
Nicaragua	2011	Ministry of Health (Nicaragua), National Institute for Development Information (Nicaragua). Nicaragua National Demographic and Health Survey 2011-2012. Managua, Nicaragua: National Institute for Development Information (Nicaragua).	X	4.3	X	4.3		All ages	Both
Niger	2011	National Institute of Statistics (Niger), World Bank. Niger Living Standards Measurement Study - Integrated Survey on Agriculture 2011-2012.	X	4	X	52		0-70	Both
Nigeria	2000	University of Ibadan (Nigeria), World Health Organization (WHO). Nigeria WHO Multi-country Survey Study on Health and Health System Responsiveness 2000-2001.	X	4.3	X	4.3		All ages	Both
Nigeria	2003	Federal Office of Statistics (Nigeria). Nigeria Living Standards Survey 2003-2004.	X	2				All ages	Both
Nigeria	2007	Central Bank of Nigeria, National Bureau of Statistics (Nigeria), Nigerian Communications Commission (NCC). Nigeria General Household Survey 2007. Abuja, Nigeria: National Bureau of Statistics (Nigeria).	X	4				All ages	Both
Nigeria	2008	National Bureau of Statistics (Nigeria). Nigeria Living Standards Survey 2008-2010. Abuja, Nigeria: National Bureau of Statistics (Nigeria).	X	2	X	4		All ages	Both

Northern Ireland	1999, 2001, 2002, 2003, 2004, 2006, 2007, 2009, 2010	World Health Organization Regional Office for Europe (WHO/Europe). European Hospital Morbidity Database 1999-2007. Copenhagen, Denmark: World Health Organization Regional Office for Europe (WHO/Europe).			X		X	All ages	Both
Northern Ireland	2006	Department of Health, Social Services and Public Safety (Northern Ireland), Information Centre for Health and Social Care, NHS, NHS England, NHS Health Scotland, NHS Wales. United Kingdom Hospital Patient and Discharge Data 2006.			X		X	All ages	Both
Northern Ireland	2012	Department of Health, Social Services and Public Safety (Northern Ireland), Information Centre for Health and Social Care, NHS, NHS England, NHS Health Scotland, NHS Wales. United Kingdom Hospital Patient and Discharge Data 2012.			X		X	All ages	Both
Northern Ireland	2013	Trinity College Dublin. Ireland Longitudinal Study on Ageing 2009-2011. Dublin, Ireland: Irish Social Science Data Archive, University College Dublin.			X	52		0-85	Both
Northern Ireland	2013	Department of Health, Social Services and Public Safety (Northern Ireland), Information Centre for Health and Social Care, NHS, NHS England, NHS Health Scotland, NHS Wales. United Kingdom Hospital Patient and Discharge Data 2013.			X		X	All ages	Both
Northern Ireland	2014	Department of Health, Social Services and Public Safety (Northern Ireland), Information Centre for Health and Social Care, NHS, NHS England, NHS Health Scotland, NHS Wales. United Kingdom Hospital Patient and Discharge Data 2014.			X		X	All ages	Both
Norway	1973	Norwegian Directorate of Health. Norway Patient Register 1973.			X		X	All ages	Both
Norway	1975	Norwegian Directorate of Health. Norway Patient Register 1975.			X		X	All ages	Both
Norway	1977	Norwegian Directorate of Health. Norway Patient Register 1977.			X		X	All ages	Both
Norway	1978	Norwegian Directorate of Health. Norway Patient Register 1978.			X		X	All ages	Both
Norway	1979	Norwegian Directorate of Health. Norway Patient Register 1979.			X		X	All ages	Both
Norway	1980	Norwegian Directorate of Health. Norway Patient Register 1980.			X		X	All ages	Both
Norway	1981	Norwegian Directorate of Health. Norway Patient Register 1981.			X		X	All ages	Both
Norway	1982	Norwegian Directorate of Health. Norway Patient Register 1982.			X		X	All ages	Both
Norway	1983	Norwegian Directorate of Health. Norway Patient Register 1983.			X		X	All ages	Both
Norway	1984	Norwegian Directorate of Health. Norway Patient Register 1984.			X		X	All ages	Both
Norway	1985	Norwegian Directorate of Health. Norway Patient Register 1985.			X		X	All ages	Both
Norway	1986	Norwegian Directorate of Health. Norway Patient Register 1986.			X		X	All ages	Both
Norway	1987	Norwegian Directorate of Health. Norway Patient Register 1987.			X		X	All ages	Both
Norway	1988	Norwegian Directorate of Health. Norway Patient Register 1988.			X		X	All ages	Both
Norway	1989	Norwegian Directorate of Health. Norway Patient Register 1989.			X		X	All ages	Both
Norway	1990	Norwegian Directorate of Health. Norway Patient Register 1990.			X		X	All ages	Both
Norway	1991	Norwegian Directorate of Health. Norway Patient Register 1991.			X		X	All ages	Both
Norway	1992	Norwegian Directorate of Health. Norway Patient Register 1992.			X		X	All ages	Both
Norway	1993	Norwegian Directorate of Health. Norway Patient Register 1993.			X		X	All ages	Both
Norway	1994	Norwegian Directorate of Health. Norway Patient Register 1994.			X		X	All ages	Both
Norway	1995	Norwegian Directorate of Health. Norway Patient Register 1995.			X		X	All ages	Both
Norway	1996	Norwegian Directorate of Health. Norway Patient Register 1996.			X		X	All ages	Both
Norway	1997	Norwegian Directorate of Health. Norway Patient Register 1997.			X		X	All ages	Both
Norway	1998	Norwegian Directorate of Health. Norway Patient Register 1998.			X		X	All ages	Both
Norway	1999	Norwegian Directorate of Health. Norway Patient Register 1999.			X		X	All ages	Both
Norway	2000	Norwegian Directorate of Health. Norway Patient Register 2000.			X		X	All ages	Both
Norway	2001	Norwegian Directorate of Health. Norway Patient Register 2001.			X		X	All ages	Both
Norway	2002	Norwegian Directorate of Health. Norway Patient Register 2002.			X		X	All ages	Both
Norway	2003, 2004, 2005, 2006, 2007	World Health Organization Regional Office for Europe (WHO/Europe). European Hospital Morbidity Database 1999-2007. Copenhagen, Denmark: World Health Organization Regional Office for Europe (WHO/Europe).			X		X	All ages	Both

Norway	2003	World Health Organization (WHO). Norway World Health Survey 2003. Geneva, Switzerland: World Health Organization (WHO), 2005.			X	52		15-95	Both
Norway	2003	Norwegian Directorate of Health. Norway Patient Register 2003.			X		X	All ages	Both
Norway	2007, 2008, 2009, 2010, 2011, 2012, 2013, 2014, 2015	Organization for Economic Co-operation and Development (OECD). OECD Health Statistics. Paris, France: Organization for Economic Co-operation and Development (OECD).	X				X	All ages	Both
Norway	2009	Norwegian Directorate of Health. Norway Patient Register 2009.			X		X	All ages	Both
Norway	2010	Norwegian Directorate of Health. Norway Patient Register 2010.			X		X	All ages	Both
Norway	2011	ISSP Research Group (2009): International Social Survey Programme: Health and Health Care - ISSP 2011. GESIS Data Archive, Cologne. ZA5800 Data file version 3.0.0, doi:10.4232/1.12252.	X	52				15-80	Both
Norway	2011	Norwegian Directorate of Health. Norway Patient Register 2011.			X		X	All ages	Both
Norway	2012	Norwegian Directorate of Health. Norway Patient Register 2012.			X		X	All ages	Both
Norway	2013	Norwegian Directorate of Health. Norway Patient Register 2013.			X		X	All ages	Both
Norway	2014	Norwegian Directorate of Health. Norway Patient Register 2014.			X		X	All ages	Both
Oman	1995	Health Ministers' Council for GCC States, Ministry of Health (Oman). Oman Family Health Survey 1995.	X	2				All ages	Both
Oman	2000	Gallup Europe, World Health Organization (WHO). Oman WHO Multi-country Survey Study on Health and Health System Responsiveness 2000-2001. Geneva, Switzerland: World Health Organization (WHO).	X	4.3	X	4.3		15-75	Both
Pakistan	1991	Federal Bureau of Statistics (Pakistan) and World Bank. Pakistan Living Standards Measurement Survey 1991. Islamabad, Pakistan: Federal Bureau of Statistics (Pakistan).	X	4.3				All ages	Both
Pakistan	2003	World Health Organization (WHO). Pakistan World Health Survey 2003-2004. Geneva, Switzerland: World Health Organization (WHO), 2005.			X	52		All ages	Both
Pakistan	2008	Pakistan Bureau of Statistics. Pakistan Social and Living Standards Measurement Survey 2008-2009.	X	2				All ages	Both
Palestine	2004	Palestinian Central Bureau of Statistics. Palestine Demographic and Health Survey 2004.	X	2	X	2		15-55	Female
Panama	1997	Ministry of Planning and Economic Policy (Panama), World Bank. Panama Living Standards Measurement Survey 1997. Washington DC, United States: World Bank.	X	4.3				All ages	Both
Panama	2003	Census and Statistics Directorate (Panama), Ministry of Economy and Finance (Panama), World Bank. Panama Living Standard Measurement Survey 2003. Washington DC, United States: World Bank.	X	4.3	X	4.3		5-100+	Both
Panama	2008	Census and Statistics Directorate (Panama), Ministry of Economy and Finance (Panama), World Bank. Panama Living Standard Measurement Survey 2008. Washington DC, United States: World Bank.	X	4.3				5-100+	Both
Papua New Guinea	1996	Papua New Guinea Institute of National Affairs, Unisearch PNG, World Bank. Papau New Guinea Living Standards Measurement Survey 1996. Washington DC, United States: World Bank.	X	4.3				0-90	Both
Paraguay	1997	Department of Statistics, Surveys and Censuses (Paraguay). Paraguay Integrated Household Survey 1997-1998. Asunción, Paraguay: Department of Statistics, Surveys and Censuses (Paraguay).	X	12.9				All ages	Both
Paraguay	2000	Department of Statistics, Surveys and Censuses (Paraguay). Paraguay Integrated Household Survey 2000-2001. Asunción, Paraguay: Department of Statistics, Surveys and Censuses (Paraguay).	X	12.9				All ages	Both
Paraguay	2002	World Health Organization (WHO). Paraguay World Health Survey 2002-2003. Geneva, Switzerland: World Health Organization (WHO), 2005.			X	52		All ages	Both
Paraguay	2003	Department of Statistics, Surveys and Censuses (Paraguay). Paraguay Permanent Household Survey 2003. Asunción, Paraguay: Department of Statistics, Surveys and Censuses (Paraguay).	X	12.9				All ages	Both
Paraguay	2004	Department of Statistics, Surveys and Censuses (Paraguay). Paraguay Permanent Household Survey 2004. Asunción, Paraguay: Department of Statistics, Surveys and Censuses (Paraguay).	X	12.9				All ages	Both
Paraguay	2005	Department of Statistics, Surveys and Censuses (Paraguay). Paraguay Permanent Household Survey 2005. Asunción, Paraguay: Department of Statistics, Surveys and Censuses (Paraguay).	X	12.9				All ages	Both
Paraguay	2006	Department of Statistics, Surveys and Censuses (Paraguay). Paraguay Permanent Household Survey 2006. Asunción, Paraguay: Department of Statistics, Surveys and Censuses (Paraguay).	X	12.9				All ages	Both
Paraguay	2007	Department of Statistics, Surveys and Censuses (Paraguay). Paraguay Permanent Household Survey 2007. Asunción, Paraguay: Department of Statistics, Surveys and Censuses (Paraguay).	X	12.9				All ages	Both

Paraguay	2008	Department of Statistics, Surveys and Censuses (Paraguay). Paraguay Permanent Household Survey 2008. Asunción, Paraguay: Department of Statistics, Surveys and Censuses (Paraguay).	X	12.9				All ages	Both
Paraguay	2009	Department of Statistics, Surveys and Censuses (Paraguay). Paraguay Permanent Household Survey 2009. Asunción, Paraguay: Department of Statistics, Surveys and Censuses (Paraguay)	X	12.9				All ages	Both
Peru	1990	National Institute of Statistics and Informatics (Peru), World Bank (WB). Peru Living Standards Measurement Survey 1990.	X	4	X	4		0-95	Both
Peru	1991	National Institute of Statistics and Informatics (INEI) (Peru), World Bank. Peru Living Standards Measurement Survey 1991. Washington DC, United States: World Bank.	X	4.3	X	4.3		All ages	Both
Peru	1994	National Institute of Statistics and Informatics (Peru), World Bank (WB). Peru Living Standards Measurement Survey 1994.	X	4	X	4		All ages	Both
Peru	1998	National Institute of Statistics and Informatics (INEI) (Peru). Peru National Household Survey, Second Quarter 1998. Lima, Peru: National Institute of Statistics and Informatics (INEI) (Peru).	X	12.9	X	12.9		All ages	Both
Peru	1999	National Institute of Statistics and Informatics (INEI) (Peru). Peru National Household Survey, Second Quarter 1999. Lima, Peru: National Institute of Statistics and Informatics (INEI) (Peru).	X	12.9	X	12.9		All ages	Both
Peru	2000	Instituto Cuánto. Peru National Living Standards Measurement Survey 2000. Lima, Peru: Instituto Cuánto.	X	4				All ages	Both
Peru	2000	National Institute of Statistics and Informatics (INEI) (Peru). Peru National Household Survey, Second Quarter 2000. Lima, Peru: National Institute of Statistics and Informatics (INEI) (Peru).	X	12.9	X	12.9		All ages	Both
Peru	2005	National Institute of Statistics and Informatics (INEI) (Peru). Peru National Household Survey 2005. Lima, Peru: National Institute of Statistics and Informatics (INEI) (Peru).	X	4	X	52		All ages	Both
Peru	2006	National Institute of Statistics and Informatics (INEI) (Peru). Peru National Household Survey 2006. Lima, Peru: National Institute of Statistics and Informatics (INEI) (Peru).	X	4	X	52		All ages	Both
Peru	2007	National Institute of Statistics and Informatics (INEI) (Peru), United Nations Economic Commission for Latin America and the Caribbean (CEPAL), Institute of Research for Development (France). Peru National Household Survey 2007. Lima, Peru: National Institute of Statistics and Informatics (INEI) (Peru).	X	4	X	52		All ages	Both
Peru	2008	National Institute of Statistics and Informatics (INEI) (Peru), United Nations Economic Commission for Latin America and the Caribbean (CEPAL), Institute of Research for Development (France). Peru National Household Survey 2008. Lima, Peru: National Institute of Statistics and Informatics (INEI) (Peru).	X	4	X	52		All ages	Both
Peru	2009	National Institute of Statistics and Informatics (INEI) (Peru). Peru National Household Survey 2009. Lima, Peru: National Institute of Statistics and Informatics (INEI) (Peru).	X	4	X	52		All ages	Both
Peru	2010	National Institute of Statistics and Informatics (INEI) (Peru). Peru National Household Survey 2010. Lima, Peru: National Institute of Statistics and Informatics (INEI) (Peru).	X	4	X	52		All ages	Both
Philippines	2003	World Health Organization (WHO). Philippines World Health Survey 2003. Geneva, Switzerland: World Health Organization (WHO), 2005.			X	52		All ages	Both
Philippines	2011	ISSP Research Group (2009): International Social Survey Programme: Health and Health Care - ISSP 2011. GESIS Data Archive, Cologne. ZA5800 Data file version 3.0.0, doi:10.4232/1.12252.	X	52				15-90	Both
Poland	1980	Ministry of Health (Poland). Poland Hospital Inpatient Discharges 1980.			X		X	All ages	Both
Poland	1981	Ministry of Health (Poland). Poland Hospital Inpatient Discharges 1981.			X		X	All ages	Both
Poland	1982	Ministry of Health (Poland). Poland Hospital Inpatient Discharges 1982.			X		X	All ages	Both
Poland	1983	Ministry of Health (Poland). Poland Hospital Inpatient Discharges 1983.			X		X	All ages	Both
Poland	1984	Ministry of Health (Poland). Poland Hospital Inpatient Discharges 1984.			X		X	All ages	Both
Poland	1985	Ministry of Health (Poland). Poland Hospital Inpatient Discharges 1985.			X		X	All ages	Both
Poland	1986	Ministry of Health (Poland). Poland Hospital Inpatient Discharges 1986.			X		X	All ages	Both
Poland	1987	Ministry of Health (Poland). Poland Hospital Inpatient Discharges 1987.			X		X	All ages	Both
Poland	1988	Ministry of Health (Poland). Poland Hospital Inpatient Discharges 1988.			X		X	All ages	Both
Poland	1989	Ministry of Health (Poland). Poland Hospital Inpatient Discharges 1989.			X		X	All ages	Both
Poland	1990	Ministry of Health (Poland). Poland Hospital Inpatient Discharges 1990.			X		X	All ages	Both
Poland	1991	Ministry of Health (Poland). Poland Hospital Inpatient Discharges 1991.			X		X	All ages	Both
Poland	1992	Ministry of Health (Poland). Poland Hospital Inpatient Discharges 1992.			X		X	All ages	Both
Poland	1993	Ministry of Health (Poland). Poland Hospital Inpatient Discharges 1993.			X		X	All ages	Both
Poland	1994	Ministry of Health (Poland). Poland Hospital Inpatient Discharges 1994.			X		X	All ages	Both
Poland	1995	Ministry of Health (Poland). Poland Hospital Inpatient Discharges 1995.			X		X	All ages	Both

Poland	1996	Ministry of Health (Poland). Poland Hospital Inpatient Discharges 1996.			X		X	All ages	Both
Poland	1997	Ministry of Health (Poland). Poland Hospital Inpatient Discharges 1997.			X		X	All ages	Both
Poland	1998	Ministry of Health (Poland). Poland Hospital Inpatient Discharges 1998.			X		X	All ages	Both
Poland	1998	Ministry of Justice (Poland). Poland Outpatient Contacts Per Person Per Year 1998.	X				X	All ages	Both
Poland	1999	Ministry of Health (Poland). Poland Hospital Inpatient Discharges 1999.			X		X	All ages	Both
Poland	1999	Ministry of Justice (Poland). Poland Outpatient Contacts Per Person Per Year 1999.	X				X	All ages	Both
Poland	2000	Public Opinion Research Center (CBOS) (Poland), World Health Organization (WHO). Poland WHO Multi-country Survey Study on Health and Health System Responsiveness 2000-2001. Geneva, Switzerland: World Health Organization (WHO).	X	4.3	X	4.3		0-95	Both
Poland	2000	Ministry of Health (Poland). Poland Hospital Inpatient Discharges 2000.			X		X	All ages	Both
Poland	2001, 2002, 2003, 2004, 2005, 2006, 2007, 2008, 2009, 2010, 2011, 2012, 2013, 2014, 2015	Organization for Economic Co-operation and Development (OECD). OECD Health Statistics. Paris, France: Organization for Economic Co-operation and Development (OECD).	X				X	All ages	Both
Poland	2001	Ministry of Health (Poland). Poland Hospital Inpatient Discharges 2001.			X		X	All ages	Both
Poland	2002	Ministry of Health (Poland). Poland Hospital Inpatient Discharges 2002.			X		X	All ages	Both
Poland	2004, 2005, 2006, 2007, 2009, 2010, 2011	World Health Organization Regional Office for Europe (WHO/Europe). European Hospital Morbidity Database 1999-2007. Copenhagen, Denmark: World Health Organization Regional Office for Europe (WHO/Europe).			X		X	All ages	Both
Poland	2004				X		X	All ages	Both
Poland	2005				X		X	All ages	Both
Poland	2010	Börsch-Supan, A. (2013). Survey of Health, Ageing and Retirement in Europe (SHARE) Wave 2. Release version: 2.6.0. SHARE-ERIC. Data set. DOI: 10.6103/SHARE.w2.260	X	52	X	52		35-100+	Both
Poland	2012	Börsch-Supan, A. (2013). Survey of Health, Ageing and Retirement in Europe (SHARE) Wave 4. Release version: 1.1.1. SHARE-ERIC. Data set. DOI: 10.6103/SHARE.w4.111	X	52	X	52		35-100+	Both
Poland	2013	ISSP Research Group (2009): International Social Survey Programme: Health and Health Care - ISSP 2011. GESIS Data Archive, Cologne. ZA5800 Data file version 3.0.0, doi:10.4232/1.12252.	X	52				15-90	Both
Poland	2013	Ministry of Health (Poland). Poland Hospital Inpatient Discharges 2013.			X		X	All ages	Both
Poland	2014	Ministry of Health (Poland). Poland Hospital Inpatient Discharges 2014.			X		X	All ages	Both
Portugal	1985	Statistics Portugal. Portugal Hospital Inpatient Discharges 1985.			X		X	All ages	Both
Portugal	1986	Statistics Portugal. Portugal Hospital Inpatient Discharges 1986.			X		X	All ages	Both
Portugal	1987	Statistics Portugal. Portugal Hospital Inpatient Discharges 1987.			X		X	All ages	Both
Portugal	1988	Statistics Portugal. Portugal Hospital Inpatient Discharges 1988.			X		X	All ages	Both
Portugal	1989	Statistics Portugal. Portugal Hospital Inpatient Discharges 1989.			X		X	All ages	Both
Portugal	1990	Statistics Portugal. Portugal Hospital Inpatient Discharges 1990.			X		X	All ages	Both
Portugal	1991	Statistics Portugal. Portugal Hospital Inpatient Discharges 1991.			X		X	All ages	Both
Portugal	1992	Statistics Portugal. Portugal Hospital Inpatient Discharges 1992.			X		X	All ages	Both
Portugal	1993	Statistics Portugal. Portugal Hospital Inpatient Discharges 1993.			X		X	All ages	Both
Portugal	1994	Statistics Portugal. Portugal Hospital Inpatient Discharges 1994.			X		X	All ages	Both
Portugal	1995	Statistics Portugal. Portugal Hospital Inpatient Discharges 1995.			X		X	All ages	Both
Portugal	1996	Statistics Portugal. Portugal Hospital Inpatient Discharges 1996.			X		X	All ages	Both
Portugal	1997	Statistics Portugal. Portugal Hospital Inpatient Discharges 1997.			X		X	All ages	Both
Portugal	1998	Statistics Portugal. Portugal Hospital Inpatient Discharges 1998.			X		X	All ages	Both

Portugal	1998	Statistics Portugal. Portugal Outpatient Contacts Per Person Per Year 1998.	X				X	All ages	Both
Portugal	1999	Statistics Portugal. Portugal Hospital Inpatient Discharges 1999.			X		X	All ages	Both
Portugal	1999	Statistics Portugal. Portugal Outpatient Contacts Per Person Per Year 1999.	X				X	All ages	Both
Portugal	2000	International Research Associates (INRA) Europe, World Health Organization (WHO). Portugal WHO Multi-country Survey Study on Health and Health System Responsiveness 2000-2001. Geneva, Switzerland: World Health Organization (WHO).	X	4.3	X	4.3		15-90	Both
Portugal	2000	Statistics Portugal. Portugal Hospital Inpatient Discharges 2000.			X		X	All ages	Both
Portugal	2001, 2002, 2003, 2004, 2005, 2006, 2007, 2008, 2009, 2010, 2011, 2012, 2013	Organization for Economic Co-operation and Development (OECD). OECD Health Statistics. Paris, France: Organization for Economic Co-operation and Development (OECD).	X				X	All ages	Both
Portugal	2001	Statistics Portugal. Portugal Hospital Inpatient Discharges 2001.			X		X	All ages	Both
Portugal	2002	Statistics Portugal. Portugal Hospital Inpatient Discharges 2002.			X		X	All ages	Both
Portugal	2003	World Health Organization (WHO). Portugal World Health Survey 2003. Geneva, Switzerland: World Health Organization (WHO), 2006.			X	52		15-95	Both
Portugal	2003	Statistics Portugal. Portugal Hospital Inpatient Discharges 2003.			X		X	All ages	Both
Portugal	2004	Statistics Portugal. Portugal Hospital Inpatient Discharges 2004.			X		X	All ages	Both
Portugal	2005	Statistics Portugal. Portugal Hospital Inpatient Discharges 2005.			X		X	All ages	Both
Portugal	2006	Statistics Portugal. Portugal Hospital Inpatient Discharges 2006.			X		X	All ages	Both
Portugal	2007	Statistics Portugal. Portugal Hospital Inpatient Discharges 2007.			X		X	All ages	Both
Portugal	2008	Statistics Portugal. Portugal Hospital Inpatient Discharges 2008.			X		X	All ages	Both
Portugal	2009	Statistics Portugal. Portugal Hospital Inpatient Discharges 2009.			X		X	All ages	Both
Portugal	2010	Statistics Portugal. Portugal Hospital Inpatient Discharges 2010.			X		X	All ages	Both
Portugal	2011	Statistics Portugal. Portugal Hospital Inpatient Discharges 2011.			X		X	All ages	Both
Portugal	2012	Börsch-Supan, A. (2013). Survey of Health, Ageing and Retirement in Europe (SHARE) Wave 4. Release version: 1.1.1. SHARE-ERIC. Data set. DOI: 10.6103/SHARE.w4.111	X	52	X	52		35-100+	Both
Portugal	2012	Statistics Portugal. Portugal Hospital Inpatient Discharges 2012.			X		X	All ages	Both
Portugal	2013	ISSP Research Group (2009): International Social Survey Programme: Health and Health Care - ISSP 2011. GESIS Data Archive, Cologne. ZA5800 Data file version 3.0.0, doi:10.4232/1.12252.	X	52				0-95	Both
Portugal	2013	Statistics Portugal. Portugal Hospital Inpatient Discharges 2013.			X		X	All ages	Both
Portugal	2014	Statistics Portugal. Portugal Hospital Inpatient Discharges 2014.			X		X	All ages	Both
Puerto Rico	2006	University of Puerto Rico, University of Wisconsin - Madison (UW - Madison). Puerto Rican Elderly: Health Conditions, Wave 2 2006-2007. San Juan, Puerto Rico: University of Puerto Rico.	X	52				65-100+	Both
Qatar	1998	Arab Fund for Economic and Social Development (AFESD), Arab Gulf Program for Development (AGFUND), Central Statistical Organization (Qatar), Health Ministers' Council for GCC States, Ministry of Public Health (Qatar), United Nations Children's Fund (UNICEF), United Nations Population Fund (UNFPA), United Nations Statistics Division (UNSD), World Health Organization (WHO). Qatar Family Health Survey 1998.	X	2				All ages	Both
Romania	1980	Ministry of Health (Romania). Romania Hospital Inpatient Discharges 1980.			X		X	All ages	Both
Romania	1985	Ministry of Health (Romania). Romania Hospital Inpatient Discharges 1985.			X		X	All ages	Both
Romania	1986	Ministry of Health (Romania). Romania Hospital Inpatient Discharges 1986.			X		X	All ages	Both
Romania	1987	Ministry of Health (Romania). Romania Hospital Inpatient Discharges 1987.			X		X	All ages	Both
Romania	1988	Ministry of Health (Romania). Romania Hospital Inpatient Discharges 1988.			X		X	All ages	Both
Romania	1989	Ministry of Health (Romania). Romania Hospital Inpatient Discharges 1989.			X		X	All ages	Both
Romania	1990	Ministry of Health (Romania). Romania Hospital Inpatient Discharges 1990.			X		X	All ages	Both
Romania	1991	Ministry of Health (Romania). Romania Hospital Inpatient Discharges 1991.			X		X	All ages	Both

Romania	1992	Ministry of Health (Romania). Romania Hospital Inpatient Discharges 1992.			X		X	All ages	Both
Romania	1993	Ministry of Health (Romania). Romania Hospital Inpatient Discharges 1993.			X		X	All ages	Both
Romania	1994	Ministry of Health (Romania). Romania Hospital Inpatient Discharges 1994.			X		X	All ages	Both
Romania	1995	Ministry of Health (Romania). Romania Hospital Inpatient Discharges 1995.			X		X	All ages	Both
Romania	1996	Ministry of Health (Romania). Romania Hospital Inpatient Discharges 1996.			X		X	All ages	Both
Romania	1997	Ministry of Health (Romania). Romania Hospital Inpatient Discharges 1997.			X		X	All ages	Both
Romania	1998	Ministry of Health (Romania). Romania Hospital Inpatient Discharges 1998.			X		X	All ages	Both
Romania	1999, 2000, 2001, 2002, 2003, 2004, 2005, 2006, 2007, 2008, 2009, 2010, 2011, 2012, 2013, 2014	World Health Organization Regional Office for Europe (WHO/Europe). European Health for All Database - Outpatient Contacts Per Person Per Year. Copenhagen, Denmark: World Health Organization Regional Office for Europe (WHO/Europe).	X				X	All ages	Both
Romania	1999	Ministry of Health (Romania). Romania Hospital Inpatient Discharges 1999.			X		X	All ages	Both
Romania	2000	International Research Associates (INRA) Europe, World Health Organization (WHO). Romania WHO Multi-country Survey Study on Health and Health System Responsiveness 2000-2001. Geneva, Switzerland: World Health Organization (WHO).	X	4.3	X	4.3		15-95	Both
Romania	2000	Ministry of Health (Romania). Romania Hospital Inpatient Discharges 2000.			X		X	All ages	Both
Romania	2001	Ministry of Health (Romania). Romania Hospital Inpatient Discharges 2001.			X		X	All ages	Both
Romania	2002	Ministry of Health (Romania). Romania Hospital Inpatient Discharges 2002.			X		X	All ages	Both
Romania	2003	Ministry of Health (Romania). Romania Hospital Inpatient Discharges 2003.			X		X	All ages	Both
Romania	2004	Ministry of Health (Romania). Romania Hospital Inpatient Discharges 2004.			X		X	All ages	Both
Romania	2005, 2006, 2007, 2009, 2010, 2011	World Health Organization Regional Office for Europe (WHO/Europe). European Hospital Morbidity Database 1999-2007. Copenhagen, Denmark: World Health Organization Regional Office for Europe (WHO/Europe).			X		X	All ages	Both
Romania	2005	Ministry of Health (Romania). Romania Hospital Inpatient Discharges 2005.			X		X	All ages	Both
Romania	2013	Ministry of Health (Romania). Romania Hospital Inpatient Discharges 2013.			X		X	All ages	Both
Russia	1987, 1988, 1989, 1990, 1991, 1992, 1993, 1994, 1995, 1996, 1997, 1998, 1999, 2000, 2001, 2002, 2003, 2004, 2005, 2006, 2007, 2009, 2010, 2011, 2012, 2013, 2014	World Health Organization Regional Office for Europe (WHO/Europe). European Health for All Database - Inpatient Care Discharges Per 100. Copenhagen, Denmark: World Health Organization Regional Office for Europe (WHO/Europe).			X		X	All ages	Both
Russia	1992	Carolina Population Center, University of North Carolina at Chapel Hill, Institute of Sociology, Russian Academy of Sciences, National Research University Higher School of Economics (Russia), ZAO Demoscope. Russia Longitudinal Monitoring Survey of HSE, Round II 1992-1993.	X	2	X	4.3		All ages	Both

Russia	1993	Carolina Population Center, University of North Carolina at Chapel Hill, Institute of Sociology, Russian Academy of Sciences, National Research University Higher School of Economics (Russia), ZAO Demoscope. Russia Longitudinal Monitoring Survey of HSE, Round IV 1993-1994.	X	2	X	4.3		All ages	Both
Russia	1994	Russia Longitudinal Monitoring Survey (RLMS-HSE), Round V 1994. National Research University Higher School of Economics, ZAO Demoscope, Carolina Population Center, Univeristy of North Carolina at Chapel Hill, Institute of Sociology, Russian Academy of Sciences.	X	2	X	12.9		10-100+	Both
Russia	1995	Russia Longitudinal Monitoring Survey (RLMS-HSE), Round VI 1995. National Research University Higher School of Economics, ZAO Demoscope, Carolina Population Center, Univeristy of North Carolina at Chapel Hill, Institute of Sociology, Russian Academy of Sciences.	X	2	X	12.9		10-100+	Both
Russia	1996	Russia Longitudinal Monitoring Survey (RLMS-HSE), Round VII 1996. National Research University Higher School of Economics, ZAO Demoscope, Carolina Population Center, Univeristy of North Carolina at Chapel Hill, Institute of Sociology, Russian Academy of Sciences.	X	2	X	12.9		All ages	Both
Russia	1998	Russia Longitudinal Monitoring Survey (RLMS-HSE), Round VIII 1998-1999. National Research University Higher School of Economics, ZAO Demoscope, Carolina Population Center, Univeristy of North Carolina at Chapel Hill, Institute of Sociology, Russian Academy of Sciences.	X	2	X	12.9		All ages	Both
Russia	1999, 2000	World Health Organization Regional Office for Europe (WHO/Europe). European Health for All Database - Outpatient Contacts Per Person Per Year. Copenhagen, Denmark: World Health Organization Regional Office for Europe (WHO/Europe).	X				X	All ages	Both
Russia	2000	Russia Longitudinal Monitoring Survey (RLMS-HSE), Round IX 2000. National Research University Higher School of Economics, ZAO Demoscope, Carolina Population Center, Univeristy of North Carolina at Chapel Hill, Institute of Sociology, Russian Academy of Sciences.	X	2				All ages	Both
Russia	2000	International Research Associates (INRA) Europe, World Health Organization (WHO). Russia WHO Multi-country Survey Study on Health and Health System Responsiveness 2000-2001. Geneva, Switzerland: World Health Organization (WHO).	X	4.3	X	4.3		15-95	Both
Russia	2001	Russia Longitudinal Monitoring Survey (RLMS-HSE), Round X 2001. National Research University Higher School of Economics, ZAO Demoscope, Carolina Population Center, Univeristy of North Carolina at Chapel Hill, Institute of Sociology, Russian Academy of Sciences.	X	2	X	4.3		All ages	Both
Russia	2001, 2002, 2003, 2004, 2005, 2006, 2007, 2008, 2009, 2010, 2011, 2012, 2013, 2014, 2015	Organization for Economic Co-operation and Development (OECD). OECD Health Statistics. Paris, France: Organization for Economic Co-operation and Development (OECD).	X				X	All ages	Both
Russia	2002	Russia Longitudinal Monitoring Survey (RLMS-HSE), Round XI 2002. National Research University Higher School of Economics, ZAO Demoscope, Carolina Population Center, Univeristy of North Carolina at Chapel Hill, Institute of Sociology, Russian Academy of Sciences.	X	2	X	12.9		All ages	Both
Russia	2003	Russia Longitudinal Monitoring Survey (RLMS-HSE), Round XII 2003. National Research University Higher School of Economics, ZAO Demoscope, Carolina Population Center, Univeristy of North Carolina at Chapel Hill, Institute of Sociology, Russian Academy of Sciences.	X	2	X	12.9		All ages	Both
Russia	2003	World Health Organization (WHO). Russia World Health Survey 2003. Geneva, Switzerland: World Health Organization (WHO), 2005.			X	52		0-95	Both
Russia	2004	Russia Longitudinal Monitoring Survey (RLMS-HSE), Round XIII 2004. National Research University Higher School of Economics, ZAO Demoscope, Carolina Population Center, Univeristy of North Carolina at Chapel Hill, Institute of Sociology, Russian Academy of Sciences.	X	4.3	X	12.9		All ages	Both
Russia	2005	Russia Longitudinal Monitoring Survey (RLMS-HSE), Round XIV 2005. National Research University Higher School of Economics, ZAO Demoscope, Carolina Population Center, Univeristy of North Carolina at Chapel Hill, Institute of Sociology, Russian Academy of Sciences.	X	4.3	X	8.6		10-100+	Both
Russia	2006	Russia Longitudinal Monitoring Survey (RLMS-HSE), Round XV 2006. National Research University Higher School of Economics, ZAO Demoscope, Carolina Population Center, Univeristy of North Carolina at Chapel Hill, Institute of Sociology, Russian Academy of Sciences.			X	12.9		All ages	Both
Russia	2007	Russian Academy of Medical Science, World Health Organization (WHO). Russia WHO Study on Global AGEing and Adult Health 2007-2010.	X	52				15-100+	Both

Russia	2007	Russia Longitudinal Monitoring Survey (RLMS-HSE), Round XVI 2007. National Research University Higher School of Economics, ZAO Demoscope, Carolina Population Center, Univeristy of North Carolina at Chapel Hill, Institute of Sociology, Russian Academy of Sciences.	X	2	X	12.9		10-100+	Both
Russia	2008	Russia Longitudinal Monitoring Survey (RLMS-HSE), Round XVII 2008. National Research University Higher School of Economics, ZAO Demoscope, Carolina Population Center, Univeristy of North Carolina at Chapel Hill, Institute of Sociology, Russian Academy of Sciences.	X	2	X	12.9		All ages	Both
Russia	2009	Russia Longitudinal Monitoring Survey (RLMS-HSE), Round XVIII 2009. National Research University Higher School of Economics, ZAO Demoscope, Carolina Population Center, Univeristy of North Carolina at Chapel Hill, Institute of Sociology, Russian Academy of Sciences.	X	2	X	12.9		10-100+	Both
Russia	2010	Center for Sociological Studies, Lomonosov Moscow State University, Concluzia-Prim Center for Survey Methodology (Moldova), Institute for Advanced Studies (Austria), London School of Hygiene and Tropical Medicine, University of Aberdeen. Russia Health in Times of Transition Household Survey 2010.			X	52		15-100+	Both
Russia	2011	Russia Longitudinal Monitoring Survey (RLMS-HSE), Round XX 2011-2012. National Research University Higher School of Economics, ZAO Demoscope, Carolina Population Center, Univeristy of North Carolina at Chapel Hill, Institute of Sociology, Russian Academy of Sciences.	X	2				10-100+	Both
Rwanda	2001	Ministry of Finance and Economic Planning (Rwanda), World Bank. Rwanda Core Welfare Indicators Questionnaire Survey 2001.	X	4				All ages	Both
Rwanda	2010	National Institute of Statistics of Rwanda. Rwanda Integrated Household Living Conditions Survey 2010-2011. Kigali, Rwanda: National Institute of Statistics of Rwanda.	X	2	X	2		All ages	Both
Saint Lucia	2004, 2010	Central Statistical Office of Saint Lucia. Saint Lucia Core Welfare Indicators Questionnaire Survey 2004. Saint Lucia: Central Statistical Office of Saint Lucia.	X	4				All ages	Both
Saudi Arabia	1995	Health Ministers' Council for GCC States, Ministry of Health (United Arab Emirates). United Arab Emirates Family Health Survey 1995.	X	2				All ages	Both
Scotland	1999, 2001, 2002, 2003, 2004, 2006, 2007, 2009, 2010	World Health Organization Regional Office for Europe (WHO/Europe). European Hospital Morbidity Database 1999-2007. Copenhagen, Denmark: World Health Organization Regional Office for Europe (WHO/Europe).			X		X	All ages	Both
Scotland	2006	Department of Health, Social Services and Public Safety (Northern Ireland), Information Centre for Health and Social Care, NHS, NHS England, NHS Health Scotland, NHS Wales. United Kingdom Hospital Patient and Discharge Data 2006.			X		X	All ages	Both
Scotland	2011	Trinity College Dublin. Ireland Longitudinal Study on Ageing 2012-2013. Dublin, Ireland: Irish Social Science Data Archive, University College Dublin.			X	52		50-85	Both
Scotland	2012	Department of Health, Social Services and Public Safety (Northern Ireland), Information Centre for Health and Social Care, NHS, NHS England, NHS Health Scotland, NHS Wales. United Kingdom Hospital Patient and Discharge Data 2012.			X		X	All ages	Both
Scotland	2013	Department of Health, Social Services and Public Safety (Northern Ireland), Information Centre for Health and Social Care, NHS, NHS England, NHS Health Scotland, NHS Wales. United Kingdom Hospital Patient and Discharge Data 2013.			X		X	All ages	Both
Scotland	2014	Department of Health, Social Services and Public Safety (Northern Ireland), Information Centre for Health and Social Care, NHS, NHS England, NHS Health Scotland, NHS Wales. United Kingdom Hospital Patient and Discharge Data 2014.			X		X	All ages	Both
Senegal	2003	World Health Organization (WHO). Senegal World Health Survey 2003. Geneva, Switzerland: World Health Organization (WHO), 2005.			X	52		All ages	Both
Serbia	2002	Ministry of Social Affairs (Serbia), World Bank. Yugoslavia, Federal Republic - Serbia Living Standards Measurement Survey 2002. Washington DC, United States: World Bank.	X	4	X	52		All ages	Both
Serbia	2003	Ministry of Social Affairs (Serbia), World Bank. Serbia and Montenegro - Serbia Living Standards Measurement Survey 2003. Washington DC, United States: World Bank.	X	4	X	52		All ages	Both
Serbia	2006	Institute of Public Health of Serbia. Serbia Outpatient Contacts Per Person Per Year 2006.	X				X	All ages	Both
Serbia	2007	Ministry of Social Affairs (Serbia), World Bank. Serbia Living Standards Measurement Survey 2007. Washington DC, United States: World Bank.	X	4.3	X	52		All ages	Both
Serbia	2007	Institute of Public Health of Serbia. Serbia Outpatient Contacts Per Person Per Year 2007.	X				X	All ages	Both
Serbia	2008	Institute of Public Health of Serbia. Serbia Outpatient Contacts Per Person Per Year 2008.	X				X	All ages	Both
Serbia	2009	Institute of Public Health of Serbia. Serbia Outpatient Contacts Per Person Per Year 2009.	X				X	All ages	Both
Serbia	2010	Institute of Public Health of Serbia. Serbia Inpatient Care Discharges Per 100 2010.			X		X	All ages	Both
Serbia	2010	Institute of Public Health of Serbia. Serbia Outpatient Contacts Per Person Per Year 2010.	X				X	All ages	Both

Serbia	2011	Institute of Public Health of Serbia. Serbia Inpatient Care Discharges Per 100 2011.			X		X	All ages	Both
Serbia	2011	Institute of Public Health of Serbia. Serbia Outpatient Contacts Per Person Per Year 2011.	X				X	All ages	Both
Serbia	2012	Institute of Public Health of Serbia. Serbia National Hospital Discharge Database 2012.			X		X	All ages	Both
Serbia	2012	Institute of Public Health of Serbia. Serbia Outpatient Contacts Per Person Per Year 2012.	X				X	All ages	Both
Serbia	2013	Institute of Public Health of Serbia. Serbia Inpatient Care Discharges Per 100 2013.			X		X	All ages	Both
Serbia	2013	Institute of Public Health of Serbia. Serbia Outpatient Contacts Per Person Per Year 2013.	X				X	All ages	Both
Serbia	2014	Institute of Public Health of Serbia. Serbia Inpatient Care Discharges Per 100 2014.			X		X	All ages	Both
Serbia	2014	Institute of Public Health of Serbia. Serbia Outpatient Contacts Per Person Per Year 2014.	X				X	All ages	Both
Seychelles	2013	Ministry of Health (Seychelles), World Health Organization (WHO). Seychelles STEPS Noncommunicable Disease Risk Factors Survey 2013-2014.	X	52				25-64	Both
Sierra Leone	2003	Statistics Sierra Leone, World Bank (WB). Sierra Leone Integrated Household Survey 2003-2004. Sierra Leone: Statistics Sierra Leone.	X	2				All ages	Both
Sierra Leone	2007	Statistics Sierra Leone, World Bank. Sierra Leone Core Welfare Indicators Questionnaire Survey 2007.	X	4				All ages	Both
Slovakia	1981, 1982, 1983, 1984, 1985, 1986, 1987, 1988, 1989, 1990, 1991, 1992, 1993, 1994, 1995, 1996, 1997, 1998, 1999, 2000, 2001, 2002, 2003, 2004, 2005, 2006, 2007, 2008, 2009, 2010, 2011, 2012, 2013, 2015	World Health Organization Regional Office for Europe (WHO/Europe). European Health for All Database - Inpatient Care Discharges Per 100. Copenhagen, Denmark: World Health Organization Regional Office for Europe (WHO/Europe).			X		X	All ages	Both
Slovakia	1995, 1998, 1999, 2000	World Health Organization Regional Office for Europe (WHO/Europe). European Health for All Database - Outpatient Contacts Per Person Per Year. Copenhagen, Denmark: World Health Organization Regional Office for Europe (WHO/Europe).	X				X	All ages	Both
Slovakia	2000	Public Health Authority of the Slovak Republic, World Health Organization (WHO). Slovakia WHO Multi-country Survey Study on Health and Health System Responsiveness 2000.	X	4.3	X	4.3		0-90	Both
Slovakia	2001, 2002, 2003, 2004, 2005, 2006, 2007, 2008, 2009, 2010, 2011, 2012, 2013, 2014, 2015	Organization for Economic Co-operation and Development (OECD). OECD Health Statistics. Paris, France: Organization for Economic Co-operation and Development (OECD).	X				X	All ages	Both
Slovakia	2003	World Health Organization (WHO). Slovakia World Health Survey 2003. Geneva, Switzerland: World Health Organization (WHO), 2005.			X	52		All ages	Both
Slovakia	2011	ISSP Research Group (2009): International Social Survey Programme: Health and Health Care - ISSP 2011. GESIS Data Archive, Cologne. ZA5800 Data file version 3.0.0, doi:10.4232/1.12252.	X	52				15-95	Both
Slovenia	1980	National Institute of Public Health (Slovenia). Slovenia National Hospital Health Care Statistics Database 1980.			X		X	All ages	Both
Slovenia	1985	National Institute of Public Health (Slovenia). Slovenia National Hospital Health Care Statistics Database 1985.			X		X	All ages	Both

Slovenia	1986	National Institute of Public Health (Slovenia). Slovenia National Hospital Health Care Statistics Database 1986.			X		X	All ages	Both
Slovenia	1987	National Institute of Public Health (Slovenia). Slovenia National Hospital Health Care Statistics Database 1987.			X		X	All ages	Both
Slovenia	1988	National Institute of Public Health (Slovenia). Slovenia National Hospital Health Care Statistics Database 1988.			X		X	All ages	Both
Slovenia	1989	National Institute of Public Health (Slovenia). Slovenia National Hospital Health Care Statistics Database 1989.			X		X	All ages	Both
Slovenia	1990	National Institute of Public Health (Slovenia). Slovenia National Hospital Health Care Statistics Database 1990.			X		X	All ages	Both
Slovenia	1991	National Institute of Public Health (Slovenia). Slovenia National Hospital Health Care Statistics Database 1991.			X		X	All ages	Both
Slovenia	1992	National Institute of Public Health (Slovenia). Slovenia National Hospital Health Care Statistics Database 1992.			X		X	All ages	Both
Slovenia	1993	National Institute of Public Health (Slovenia). Slovenia National Hospital Health Care Statistics Database 1993.			X		X	All ages	Both
Slovenia	1994	National Institute of Public Health (Slovenia). Slovenia National Hospital Health Care Statistics Database 1994.			X		X	All ages	Both
Slovenia	1995	National Institute of Public Health (Slovenia). Slovenia National Hospital Health Care Statistics Database 1995.			X		X	All ages	Both
Slovenia	1996	National Institute of Public Health (Slovenia). Slovenia National Hospital Health Care Statistics Database 1996.			X		X	All ages	Both
Slovenia	1997	National Institute of Public Health (Slovenia). Slovenia National Hospital Health Care Statistics Database 1997.			X		X	All ages	Both
Slovenia	1998	National Institute of Public Health (Slovenia). Slovenia National Hospital Health Care Statistics Database 1998.			X		X	All ages	Both
Slovenia	1999	National Institute of Public Health (Slovenia). Slovenia National Hospital Health Care Statistics Database 1999.			X		X	All ages	Both
Slovenia	2000	National Institute of Public Health (Slovenia). Slovenia National Hospital Health Care Statistics Database 2000.			X		X	All ages	Both
Slovenia	2001	National Institute of Public Health (Slovenia). Slovenia National Hospital Health Care Statistics Database 2001.			X		X	All ages	Both
Slovenia	2002	National Institute of Public Health (Slovenia). Slovenia National Hospital Health Care Statistics Database 2002.			X		X	All ages	Both
Slovenia	2003	World Health Organization (WHO). Slovenia World Health Survey 2003. Geneva, Switzerland: World Health Organization (WHO), 2005.			X	52		15-95	Both
Slovenia	2003	National Institute of Public Health (Slovenia). Slovenia National Hospital Health Care Statistics Database 2003.			X		X	All ages	Both
Slovenia	2004	National Institute of Public Health (Slovenia). Slovenia National Hospital Health Care Statistics Database 2004.			X		X	All ages	Both
Slovenia	2005	National Institute of Public Health (Slovenia). Slovenia National Hospital Health Care Statistics Database 2005.			X		X	All ages	Both
Slovenia	2006	National Institute of Public Health (Slovenia). Slovenia National Hospital Health Care Statistics Database 2006.			X		X	All ages	Both
Slovenia	2007, 2009, 2010, 2011	World Health Organization Regional Office for Europe (WHO/Europe). European Hospital Morbidity Database 1999-2007. Copenhagen, Denmark: World Health Organization Regional Office for Europe (WHO/Europe).			X		X	All ages	Both
Slovenia	2007, 2008, 2009, 2010, 2011, 2012, 2013, 2014, 2015	Organization for Economic Co-operation and Development (OECD). OECD Health Statistics. Paris, France: Organization for Economic Co-operation and Development (OECD).	X				X	All ages	Both
Slovenia	2007	National Institute of Public Health (Slovenia). Slovenia National Hospital Health Care Statistics Database 2007.			X		X	All ages	Both
Slovenia	2008	National Institute of Public Health (Slovenia). Slovenia National Hospital Health Care Statistics Database 2008.			X		X	All ages	Both
Slovenia	2011	ISSP Research Group (2009): International Social Survey Programme: Health and Health Care - ISSP 2011. GESIS Data Archive, Cologne. ZA5800 Data file version 3.0.0, doi:10.4232/1.12252.	X	52				15-100+	Both
Slovenia	2012	Börsch-Supan, A. (2013). Survey of Health, Ageing and Retirement in Europe (SHARE) Wave 4. Release version: 1.1.1. SHARE-ERIC. Data set. DOI: 10.6103/SHARE.w4.111	X	52	X	52		35-100+	Both
Slovenia	2013	Börsch-Supan, A. (2015). Survey of Health, Ageing and Retirement in Europe (SHARE) Wave 5. Release version: 1.0.0. SHARE-ERIC. Data set. DOI: 10.6103/SHARE.w5.100	X	52	X	52		All ages	Both
Slovenia	2013	National Institute of Public Health (Slovenia). Slovenia National Hospital Health Care Statistics Database 2013.			X		X	All ages	Both
Slovenia	2014	National Institute of Public Health (Slovenia). Slovenia National Hospital Health Care Statistics Database 2014.			X		X	All ages	Both
South Africa	1993	Southern Africa Labour Development Research Unit (SALDRU), University of Cape Town, World Bank. South Africa Living Standards Measurement Study 1993. Washington DC, United States: World Bank.	X	4				All ages	Both
South Africa	1994	Central Statistical Service (South Africa). South Africa October Household Survey 1994.			X	4.3		All ages	Both
South Africa	1995	Central Statistical Service (South Africa). South Africa October Household Survey 1995.	X	4.3				All ages	Both

South Africa	1996	Central Statistical Service (South Africa). South Africa October Household Survey 1996.	X	4.3	X	4.3		All ages	Both
South Africa	2001, 2002, 2003, 2004, 2005, 2006, 2007, 2008, 2009, 2010, 2011, 2012, 2013	Organization for Economic Co-operation and Development (OECD). OECD Health Statistics. Paris, France: Organization for Economic Co-operation and Development (OECD).	X				X	All ages	Both
South Africa	2002	Statistics South Africa. South Africa General Household Survey 2002. Pretoria, South Africa: Statistics South Africa.	X	4.3				All ages	Both
South Africa	2003	Statistics South Africa. South Africa General Household Survey 2003. Pretoria, South Africa: Statistics South Africa.	X	4.3				All ages	Both
South Africa	2004	Statistics South Africa. South Africa General Household Survey 2004. Pretoria, South Africa: Statistics South Africa.	X	4.3				All ages	Both
South Africa	2005	Statistics South Africa. South Africa General Household Survey 2005. Pretoria, South Africa: Statistics South Africa.	X	4.3				All ages	Both
South Africa	2006	Statistics South Africa. South Africa General Household Survey 2006. Pretoria, South Africa: Statistics South Africa.	X	4.3				All ages	Both
South Africa	2007	Statistics South Africa. South Africa General Household Survey 2007. Cape Town, South Africa: DataFirst.	X	4.3				All ages	Both
South Africa	2007	Department of Health (South Africa), Human Sciences Research Council, World Health Organization (WHO). South Africa WHO Study on Global AGEing and Adult Health 2007-2008. Geneva, Switzerland: World Health Organization (WHO).	X	52				15-100+	Both
South Africa	2008	University of Cape Town, Southern Africa Labour and Development Research Unit. National Income Dynamics Study (NIDS) Wave 1 [computer files]. Cape Town: Southern Africa Labour and Development Research Unit [producer], 2009. Cape Town: DataFirst [distributor], 2009	X	4.3				0-20	Both
South Africa	2008	Statistics South Africa. South Africa General Household Survey 2008. Pretoria, South Africa: Statistics South Africa.	X	4.3				All ages	Both
South Africa	2008	Statistics South Africa. South Africa Living Conditions Survey 2008-2009. Pretoria, South Africa: Statistics South Africa.	X	4.3				All ages	Both
South Africa	2009	Statistics South Africa. South Africa General Household Survey 2009. Pretoria, South Africa: Statistics South Africa.	X	4.3				All ages	Both
South Africa	2010	Statistics South Africa. South Africa General Household Survey 2010. Pretoria, South Africa: Statistics South Africa.	X	4.3				All ages	Both
South Africa	2010	Statistics South Africa. South Africa Income and Expenditure Survey 2010-2011. Pretoria, South Africa: Statistics South Africa.	X	4.3				All ages	Both
South Africa	2011	ISSP Research Group (2009): International Social Survey Programme: Health and Health Care - ISSP 2011. GESIS Data Archive, Cologne. ZA5800 Data file version 3.0.0, doi:10.4232/1.12252.	X	52				All ages	Both
South Africa	2012	Southern Africa Labour and Development Research Unit. National Income Dynamics Study 2012, Wave 3 [dataset]. Version 1.2. Cape Town: Southern Africa Labour and Development Research Unit [producer], 2013. Cape Town: DataFirst [distributor], 2013	X	4.3				All ages	Both
South Africa	2012	Statistics South Africa. South Africa General Household Survey 2012. Pretoria, South Africa: Statistics South Africa, 2013.	X	4.3				All ages	Both
South Africa	2012	Human Sciences Research Council, South African Medical Research Council. South Africa National Health and Nutrition Examination Survey 2012.			X	52		15-100+	Both
South Korea	1996, 1999, 2002, 2003, 2005, 2006, 2008, 2009, 2010, 2011, 2012, 2013, 2014, 2015	Organization for Economic Co-operation and Development (OECD). OECD Health Statistics. Paris, France: Organization for Economic Co-operation and Development (OECD).			X		X	All ages	Both
South Korea	2000	Graduate School of Public Health, Seoul National University, World Health Organization (WHO). South Korea WHO Multi-country Survey Study on Health and Health System Responsiveness 2000-2001. Geneva, Switzerland: World Health Organization (WHO).	X	4.3	X	4.3		All ages	Both
Spain	1972	Ministry of Health, Social Services and Equality (Spain), National Statistics Institute (Spain). Spain Statistics on Health Establishments Providing Inpatient Care 1972.			X		X	All ages	Both
Spain	1973	Ministry of Health, Social Services and Equality (Spain), National Statistics Institute (Spain). Spain Statistics on Health Establishments Providing Inpatient Care 1973.			X		X	All ages	Both
Spain	1974	Ministry of Health, Social Services and Equality (Spain), National Statistics Institute (Spain). Spain Statistics on Health Establishments Providing Inpatient Care 1974.			X		X	All ages	Both

Spain	1975	Ministry of Health, Social Services and Equality (Spain), National Statistics Institute (Spain). Spain Statistics on Health Establishments Providing Inpatient Care 1975.			X		X	All ages	Both
Spain	1976	Ministry of Health, Social Services and Equality (Spain), National Statistics Institute (Spain). Spain Statistics on Health Establishments Providing Inpatient Care 1976.			X		X	All ages	Both
Spain	1977	Ministry of Health, Social Services and Equality (Spain), National Statistics Institute (Spain). Spain Statistics on Health Establishments Providing Inpatient Care 1977.			X		X	All ages	Both
Spain	1978	Ministry of Health, Social Services and Equality (Spain), National Statistics Institute (Spain). Spain Statistics on Health Establishments Providing Inpatient Care 1978.			X		X	All ages	Both
Spain	1979	Ministry of Health, Social Services and Equality (Spain), National Statistics Institute (Spain). Spain Statistics on Health Establishments Providing Inpatient Care 1979.			X		X	All ages	Both
Spain	1980	Ministry of Health, Social Services and Equality (Spain), National Statistics Institute (Spain). Spain Statistics on Health Establishments Providing Inpatient Care 1980.			X		X	All ages	Both
Spain	1981	Ministry of Health, Social Services and Equality (Spain), National Statistics Institute (Spain). Spain Statistics on Health Establishments Providing Inpatient Care 1981.			X		X	All ages	Both
Spain	1982	Ministry of Health, Social Services and Equality (Spain), National Statistics Institute (Spain). Spain Statistics on Health Establishments Providing Inpatient Care 1982.			X		X	All ages	Both
Spain	1983	Ministry of Health, Social Services and Equality (Spain), National Statistics Institute (Spain). Spain Statistics on Health Establishments Providing Inpatient Care 1983.			X		X	All ages	Both
Spain	1984	Ministry of Health, Social Services and Equality (Spain), National Statistics Institute (Spain). Spain Statistics on Health Establishments Providing Inpatient Care 1984.			X		X	All ages	Both
Spain	1985	Ministry of Health, Social Services and Equality (Spain), National Statistics Institute (Spain). Spain Statistics on Health Establishments Providing Inpatient Care 1985.			X		X	All ages	Both
Spain	1986	Ministry of Health, Social Services and Equality (Spain), National Statistics Institute (Spain). Spain Statistics on Health Establishments Providing Inpatient Care 1986.			X		X	All ages	Both
Spain	1987	Ministry of Health, Social Services and Equality (Spain), National Statistics Institute (Spain). Spain Statistics on Health Establishments Providing Inpatient Care 1987.			X		X	All ages	Both
Spain	1988	Ministry of Health, Social Services and Equality (Spain), National Statistics Institute (Spain). Spain Statistics on Health Establishments Providing Inpatient Care 1988.			X		X	All ages	Both
Spain	1989	Ministry of Health, Social Services and Equality (Spain), National Statistics Institute (Spain). Spain Statistics on Health Establishments Providing Inpatient Care 1989.			X		X	All ages	Both
Spain	1990	Ministry of Health, Social Services and Equality (Spain), National Statistics Institute (Spain). Spain Statistics on Health Establishments Providing Inpatient Care 1990.			X		X	All ages	Both
Spain	1991	Ministry of Health, Social Services and Equality (Spain), National Statistics Institute (Spain). Spain Statistics on Health Establishments Providing Inpatient Care 1991.			X		X	All ages	Both
Spain	1992	Ministry of Health, Social Services and Equality (Spain), National Statistics Institute (Spain). Spain Statistics on Health Establishments Providing Inpatient Care 1992.			X		X	All ages	Both
Spain	1993	Ministry of Health, Social Services and Equality (Spain), National Statistics Institute (Spain). Spain Statistics on Health Establishments Providing Inpatient Care 1993.			X		X	All ages	Both
Spain	1994	Ministry of Health, Social Services and Equality (Spain), National Statistics Institute (Spain). Spain Statistics on Health Establishments Providing Inpatient Care 1994.			X		X	All ages	Both
Spain	1995	Ministry of Health, Social Services and Equality (Spain), National Statistics Institute (Spain). Spain Statistics on Health Establishments Providing Inpatient Care 1995.			X		X	All ages	Both
Spain	1996	Ministry of Health, Social Services and Equality (Spain), National Statistics Institute (Spain). Spain Statistics on Health Establishments Providing Inpatient Care 1996.			X		X	All ages	Both
Spain	1997	Ministry of Health, Social Services and Equality (Spain). Spain Statistics on Health Establishments Providing Inpatient Care 1997.			X		X	All ages	Both
Spain	1998	Ministry of Health, Social Services and Equality (Spain). Spain Statistics on Health Establishments Providing Inpatient Care 1998.			X		X	All ages	Both
Spain	1999	Ministry of Health, Social Services and Equality (Spain). Spain Statistics on Health Establishments Providing Inpatient Care 1999.			X		X	All ages	Both
Spain	2000	International Research Associates (INRA) Europe, World Health Organization (WHO). Spain WHO Multi-country Survey Study on Health and Health System Responsiveness 2000-2001. Geneva, Switzerland: World Health Organization (WHO).	X	4.3	X	4.3		15-95	Both
Spain	2000	Ministry of Health, Social Services and Equality (Spain). Spain Statistics on Health Establishments Providing Inpatient Care 2000.			X		X	All ages	Both
Spain	2001	Ministry of Health, Social Services and Equality (Spain). Spain Statistics on Health Establishments Providing Inpatient Care 2001.			X		X	All ages	Both
Spain	2002	World Health Organization (WHO). Spain World Health Survey 2002-2003. Geneva, Switzerland: World Health Organization (WHO), 2005.			X	52		All ages	Both
Spain	2002	Ministry of Health, Social Services and Equality (Spain). Spain Statistics on Health Establishments Providing Inpatient Care 2002.			X		X	All ages	Both

Spain	2003	Ministry of Health, Social Services and Equality (Spain). Spain Statistics on Health Establishments Providing Inpatient Care 2003.			X		X	All ages	Both
Spain	2004	Ministry of Health, Social Services and Equality (Spain), Sociological Research Center (Spain). Spain Health Barometer Survey 2004. Madrid, Spain: Ministry of Health, Social Services and Equality (Spain), 2004.	X	52	X	52		All ages	Both
Spain	2004	Ministry of Health, Social Services and Equality (Spain). Spain Statistics on Health Establishments Providing Inpatient Care 2004.			X		X	All ages	Both
Spain	2005	Ministry of Health, Social Services and Equality (Spain). Spain Statistics on Health Establishments Providing Inpatient Care 2005.			X		X	All ages	Both
Spain	2006	Ministry of Health, Social Services and Equality (Spain), National Statistics Institute (Spain). Spain National Health Survey 2006-2007.	X	4	X	52		15-100+	Both
Spain	2006	Börsch-Supan, A. (2013). Survey of Health, Ageing and Retirement in Europe (SHARE) Wave 1. Release version: 2.6.0. SHARE-ERIC. Data set. DOI: 10.6103/SHARE.w1.260	X	52	X	52		All ages	Both
Spain	2006	Ministry of Health, Social Services and Equality (Spain). Spain Statistics on Health Establishments Providing Inpatient Care 2006.			X		X	All ages	Both
Spain	2007	Ministry of Health, Social Services and Equality (Spain). Spain Statistics on Health Establishments Providing Inpatient Care 2007.			X		X	All ages	Both
Spain	2008	Ministry of Health, Social Services and Equality (Spain). Spain Statistics on Health Establishments Providing Inpatient Care 2008.			X		X	All ages	Both
Spain	2009	Ministry of Health, Social Services and Equality (Spain). Spain Statistics on Health Establishments Providing Inpatient Care 2009.			X		X	All ages	Both
Spain	2010	Börsch-Supan, A. (2013). Survey of Health, Ageing and Retirement in Europe (SHARE) Wave 2. Release version: 2.6.0. SHARE-ERIC. Data set. DOI: 10.6103/SHARE.w2.260	X	52	X	52		35-100+	Both
Spain	2010	Ministry of Health, Social Services and Equality (Spain). Spain Statistics on Health Establishments Providing Inpatient Care 2010.			X		X	All ages	Both
Spain	2011	Ministry of Health, Social Services and Equality (Spain). Spain Statistics on Health Establishments Providing Inpatient Care 2011.			X		X	All ages	Both
Spain	2012	Börsch-Supan, A. (2013). Survey of Health, Ageing and Retirement in Europe (SHARE) Wave 4. Release version: 1.1.1. SHARE-ERIC. Data set. DOI: 10.6103/SHARE.w4.111	X	52	X	52		25-100+	Both
Spain	2012	Ministry of Health, Social Services and Equality (Spain). Spain Statistics on Health Establishments Providing Inpatient Care 2012.			X		X	All ages	Both
Spain	2013	Börsch-Supan, A. (2015). Survey of Health, Ageing and Retirement in Europe (SHARE) Wave 5. Release version: 1.0.0. SHARE-ERIC. Data set. DOI: 10.6103/SHARE.w5.100	X	52	X	52		All ages	Both
Spain	2013	Ministry of Health, Social Services and Equality (Spain). Spain Statistics on Health Establishments Providing Inpatient Care 2013.			X		X	All ages	Both
Spain	2014	Ministry of Health, Social Services and Equality (Spain). Spain Statistics on Health Establishments Providing Inpatient Care 2014.			X		X	All ages	Both
Sri Lanka	2003	World Health Organization (WHO). Sri Lanka World Health Survey 2003. Geneva, Switzerland: World Health Organization (WHO), 2005.			X	52		15-100+	Both
Swaziland	2003	World Health Organization (WHO). Swaziland World Health Survey 2003. Geneva, Switzerland: World Health Organization (WHO), 2005.			X	52		All ages	Both
Sweden	1987	National Board of Health and Welfare (Sweden). Sweden National Patient Register 1987.			X		X	All ages	Both
Sweden	1988	National Board of Health and Welfare (Sweden). Sweden National Patient Register 1988.			X		X	All ages	Both
Sweden	1989	National Board of Health and Welfare (Sweden). Sweden National Patient Register 1989.			X		X	All ages	Both
Sweden	1990	National Board of Health and Welfare (Sweden). Sweden National Patient Register 1990.			X		X	All ages	Both
Sweden	1991	National Board of Health and Welfare (Sweden). Sweden National Patient Register 1991.			X		X	All ages	Both
Sweden	1992	National Board of Health and Welfare (Sweden). Sweden National Patient Register 1992.			X		X	All ages	Both
Sweden	1993	National Board of Health and Welfare (Sweden). Sweden National Patient Register 1993.			X		X	All ages	Both
Sweden	1994	National Board of Health and Welfare (Sweden). Sweden National Patient Register 1994.			X		X	All ages	Both
Sweden	1995	National Board of Health and Welfare (Sweden). Sweden National Patient Register 1995.			X		X	All ages	Both
Sweden	1996	National Board of Health and Welfare (Sweden). Sweden National Patient Register 1996.			X		X	All ages	Both
Sweden	1997	National Board of Health and Welfare (Sweden). Sweden National Patient Register 1997.			X		X	All ages	Both
Sweden	1998	National Board of Health and Welfare (Sweden). Sweden National Patient Register 1998. Stockholm, Sweden: National Board of Health and Welfare (Sweden).			X		X	All ages	Both

Sweden	1999, 2001, 2002, 2003, 2004, 2005, 2006, 2007, 2009	World Health Organization Regional Office for Europe (WHO/Europe). European Hospital Morbidity Database 1999-2007. Copenhagen, Denmark: World Health Organization Regional Office for Europe (WHO/Europe).			X		X	All ages	Both
Sweden	1999	National Board of Health and Welfare (Sweden). Sweden National Patient Register 1999. Stockholm, Sweden: National Board of Health and Welfare (Sweden).			X		X	All ages	Both
Sweden	2000	International Research Associates (INRA) Europe, World Health Organization (WHO). Sweden WHO Multi-country Survey Study on Health and Health System Responsiveness 2000-2001. Geneva, Switzerland: World Health Organization (WHO).	X	4.3	X	4.3		15-95	Both
Sweden	2002, 2003, 2004, 2005, 2006, 2007, 2008, 2009, 2010, 2011, 2012, 2013, 2014, 2015	Organization for Economic Co-operation and Development (OECD). OECD Health Statistics. Paris, France: Organization for Economic Co-operation and Development (OECD).	X				X	All ages	Both
Sweden	2003	World Health Organization (WHO). Sweden World Health Survey 2003. Geneva, Switzerland: World Health Organization (WHO), 2005.			X	52		15-95	Both
Sweden	2004	Statistics Sweden, Swedish National Institute of Public Health. Sweden National Survey of Public Health 2004.			X	12.9		16-84	Both
Sweden	2005	Statistics Sweden, Swedish National Institute of Public Health. Sweden National Survey of Public Health 2005.	X	12.9	X	12.9		16-84	Both
Sweden	2006, 2007	Statistics Sweden, Swedish National Institute of Public Health. Sweden National Survey of Public Health 2007.	X	12.9	X	12.9		16-84	Both
Sweden	2006	Börsch-Supan, A. (2013). Survey of Health, Ageing and Retirement in Europe (SHARE) Wave 1. Release version: 2.6.0. SHARE-ERIC. Data set. DOI: 10.6103/SHARE.w1.260	X	52	X	52		All ages	Both
Sweden	2008	Statistics Sweden, Swedish National Institute of Public Health. Sweden National Survey of Public Health 2008.	X	12.9	X	12.9		16-84	Both
Sweden	2009	Statistics Sweden, Swedish National Institute of Public Health. Sweden National Survey of Public Health 2009.	X	12.9	X	12.9		16-84	Both
Sweden	2010	Börsch-Supan, A. (2013). Survey of Health, Ageing and Retirement in Europe (SHARE) Wave 2. Release version: 2.6.0. SHARE-ERIC. Data set. DOI: 10.6103/SHARE.w2.260	X	52	X	52		25-100+	Both
Sweden	2010	Statistics Sweden, Swedish National Institute of Public Health. Sweden National Survey of Public Health 2010.	X	12.9	X	12.9		16-84	Both
Sweden	2011	National Board of Health and Welfare (Sweden). Sweden National Patient Register 2011. Stockholm, Sweden: National Board of Health and Welfare (Sweden).			X		X	All ages	Both
Sweden	2011	Statistics Sweden, Swedish National Institute of Public Health. Sweden National Survey of Public Health 2011.	X	12.9	X	12.9		16-84	Both
Sweden	2011	ISSP Research Group (2009): International Social Survey Programme: Health and Health Care - ISSP 2011. GESIS Data Archive, Cologne. ZA5800 Data file version 3.0.0, doi:10.4232/1.12252.	X	52				15-85	Both
Sweden	2012	National Board of Health and Welfare (Sweden). Sweden National Patient Register 2012. Stockholm, Sweden: National Board of Health and Welfare (Sweden).			X		X	All ages	Both
Sweden	2012	Statistics Sweden, Swedish National Institute of Public Health. Sweden National Survey of Public Health 2012.	X	12.9	X	12.9		16-84	Both
Sweden	2012	Börsch-Supan, A. (2013). Survey of Health, Ageing and Retirement in Europe (SHARE) Wave 4. Release version: 1.1.1. SHARE-ERIC. Data set. DOI: 10.6103/SHARE.w4.111	X	52	X	52		30-100+	Both
Sweden	2013	Statistics Sweden, Swedish National Institute of Public Health. Sweden National Survey of Public Health 2013.	X	12.9	X	12.9		16-84	Both
Sweden	2013	Börsch-Supan, A. (2015). Survey of Health, Ageing and Retirement in Europe (SHARE) Wave 5. Release version: 1.0.0. SHARE-ERIC. Data set. DOI: 10.6103/SHARE.w5.100	X	52	X	52		All ages	Both
Sweden	2013	National Board of Health and Welfare (Sweden). Sweden National Patient Register 2013.			X		X	All ages	Both
Sweden	2014	Public Health Agency of Sweden, Statistics Sweden. Sweden National Survey of Public Health 2014.	X	12.9	X	12.9		16-84	Both
Sweden	2014	National Board of Health and Welfare (Sweden). Sweden National Patient Register 2014.			X		X	All ages	Both
Switzerland	1997	Federal Statistical Office (Switzerland). Switzerland Medical Statistics of Hospitals 1997.			X		X	All ages	Both
Switzerland	1998	Federal Statistical Office (Switzerland). Switzerland Medical Statistics of Hospitals 1998.			X		X	All ages	Both
Switzerland	2000	LINK Institute for Market and Social Research (Switzerland), World Health Organization (WHO). Switzerland WHO Multi-country Survey Study on Health and Health System Responsiveness 2000-2001. Geneva, Switzerland: World Health Organization (WHO).	X	4.3	X	4.3		0-95	Both

Switzerland	2001, 2002, 2003, 2004, 2005, 2006, 2007, 2009, 2010, 2011	World Health Organization Regional Office for Europe (WHO/Europe). European Hospital Morbidity Database 1999-2007. Copenhagen, Denmark: World Health Organization Regional Office for Europe (WHO/Europe).			X		X	All ages	Both
Switzerland	2003, 2008, 2013	Organization for Economic Co-operation and Development (OECD). OECD Health Statistics. Paris, France: Organization for Economic Co-operation and Development (OECD).	X				X	All ages	Both
Switzerland	2006	Börsch-Supan, A. (2013). Survey of Health, Ageing and Retirement in Europe (SHARE) Wave 1. Release version: 2.6.0. SHARE-ERIC. Data set. DOI: 10.6103/SHARE.w1.260	X	52	X	52		All ages	Both
Switzerland	2010	Börsch-Supan, A. (2013). Survey of Health, Ageing and Retirement in Europe (SHARE) Wave 2. Release version: 2.6.0. SHARE-ERIC. Data set. DOI: 10.6103/SHARE.w2.260	X	52	X	52		35-100+	Both
Switzerland	2011	ISSP Research Group (2009): International Social Survey Programme: Health and Health Care - ISSP 2011. GESIS Data Archive, Cologne. ZA5800 Data file version 3.0.0, doi:10.4232/1.12252.	X	52				15-100+	Both
Switzerland	2012	Börsch-Supan, A. (2013). Survey of Health, Ageing and Retirement in Europe (SHARE) Wave 4. Release version: 1.1.1. SHARE-ERIC. Data set. DOI: 10.6103/SHARE.w4.111	X	52	X	52		30-100+	Both
Switzerland	2013	Börsch-Supan, A. (2015). Survey of Health, Ageing and Retirement in Europe (SHARE) Wave 5. Release version: 1.0.0. SHARE-ERIC. Data set. DOI: 10.6103/SHARE.w5.100	X	52	X	52		All ages	Both
Switzerland	2013	Federal Statistical Office (Switzerland). Switzerland Medical Statistics of Hospitals 2013.			X		X	All ages	Both
Switzerland	2014	Federal Statistical Office (Switzerland). Switzerland Medical Statistics of Hospitals 2014.			X		X	All ages	Both
Syria	2000	Ministry of Health (Syria), World Health Organization (WHO). Syria WHO Multi-country Survey Study on Health and Health System Responsiveness 2000-2001.	X	4.3	X	4.3		All ages	Both
Taiwan	1995, 1996, 1997, 1998, 1999, 2000, 2001, 2002, 2003, 2004, 2005	Ho Chan WS. Taiwan's healthcare report 2010. EPMA J. 2010; 1(4): 563–85.	X				X	All ages	Both
Tajikistan	1986, 1987, 1988, 1989, 1990, 1991, 1992, 1993, 1994, 1995, 1996, 1997, 1998, 1999, 2000, 2001, 2002, 2003, 2004, 2005, 2006, 2007, 2008, 2009, 2010, 2011, 2012, 2013, 2014, 2015, 2016	World Health Organization Regional Office for Europe (WHO/Europe). European Health for All Database - Inpatient Care Discharges Per 100. Copenhagen, Denmark: World Health Organization Regional Office for Europe (WHO/Europe).			X		X	All ages	Both
Tajikistan	1999, 2000, 2001, 2002, 2003, 2004, 2005, 2006, 2007, 2008, 2009, 2010, 2011,	World Health Organization Regional Office for Europe (WHO/Europe). European Health for All Database - Outpatient Contacts Per Person Per Year. Copenhagen, Denmark: World Health Organization Regional Office for Europe (WHO/Europe).	X				X	All ages	Both

	2012, 2013, 2014, 2015, 2016									
Tajikistan	2003	National State Statistical Agency (Tajikistan), World Bank. Tajikistan Living Standards Measurement Survey 2003.			X	52		All ages	Both
Tajikistan	2007	National State Statistical Agency (Tajikistan), World Bank. Tajikistan Living Standards Measurement Survey 2007.			X	4		All ages	Both
Tajikistan	2009	National State Statistical Agency (Tajikistan), World Bank. Tajikistan Living Standards Measurement Survey 2009.	X	4	X	52		All ages	Both
Tanzania	1993	Planning Commission (Tanzania), University of Dar es Salaam, World Bank. Tanzania Living Standards Measurement Study 1993-1994. Washington DC, United States: World Bank.			X	4		All ages	Both
Tanzania	1994	Bureau of Statistics (Tanzania), Macro International, Inc, United States Agency for International Development (USAID). Tanzania Knowledge, Attitudes, and Practices Survey 1994. Fairfax, United States: ICF International.	X	52				15-50	Female
Tanzania	2000	Bureau of Statistics (Tanzania), Oxford Policy Management. Tanzania Household Budget Survey 2000-2001. Dar es Salaam, Tanzania: Bureau of Statistics (Tanzania).	X	4				All ages	Both
Tanzania	2004	University of Dar es Salaam, World Bank. Tanzania - Kagera Living Standards Measurement Study 2004.	X	4				All ages	Both
Tanzania	2005	Economic Development Initiatives (EDI), World Bank (WB). Tanzania Core Welfare Indicators Questionnaire Survey 2005. Bukoba, Tanzania: Economic Development Initiatives (EDI).	X	4				All ages	Both
Tanzania	2006	Economic Development Initiatives (EDI), World Bank (WB). Tanzania Core Welfare Indicators Questionnaire Survey 2006-2007. Bukoba, Tanzania: Economic Development Initiatives (EDI).	X	4.3				All ages	Both
Tanzania	2007	National Bureau of Statistics (Tanzania). Tanzania Household Budget Survey 2007. Dar es Salaam, Tanzania: National Bureau of Statistics (Tanzania).	X	4				All ages	Both
Tanzania	2010, 2011	National Bureau of Statistics (Tanzania). Tanzania Living Standards Measurement Study - Integrated Surveys on Agriculture 2010-2011. Dar es Salaam, Tanzania: National Bureau of Statistics (Tanzania).	X	4				All ages	Both
Thailand	2000	Mahidol University, World Health Organization (WHO). Thailand WHO Multi-country Survey Study on Health and Health System Responsiveness 2000-2001. Geneva, Switzerland: World Health Organization (WHO).	X	4.3	X	4.3		15-90	Both
Thailand	2004	Ministry of Public Health (Thailand). Thailand National Health and Examination Survey 2003-2004.	X	4.3	X	52		All ages	Both
Thailand	2007	Ministry of Public Health (Thailand). Thailand Noncommunicable Disease and Injury Behavior Risk Surveillance Survey 2007.	X	52				15-74	Both
The Bahamas	2001	Bahamas Department of Statistics, Ministry of Health (The Bahamas). Bahamas Living Conditions Survey 2001. Nassau, The Bahamas: Bahamas Department of Statistics.	X	4	X	52		All ages	Both
The Gambia	1992	Central Statistics Department, Ministry of Finance and Economic Affairs (Gambia), Carl Bro International. Gambia Household Economic Survey 1992-1993. Banjul, Gambia: Central Statistics Department, Ministry of Finance and Economic Affairs (Gambia).	X	2				All ages	Both
The Gambia	1992	Central Statistics Department (Gambia), Department of State for Finance and Economic Affairs (Gambia). Gambia Social Dimensions of Adjustment Priority Survey I 1992.	X	2				All ages	Both
The Gambia	1993	Central Statistics Department (Gambia). Gambia Household Education and Health Survey 1993-1994. Banjul, The Gambia: Central Statistics Department (Gambia).	X	2				All ages	Both
Timor-Leste	2001	National Statistics Directorate (Timor-Leste), World Bank. Timor-Leste Living Standards and Measurement Survey 2001. Washington DC, United States: World Bank.	X	4.3	X	4.3		All ages	Both
Timor-Leste	2007	National Statistics Directorate (Timor-Leste), World Bank. Timor-Leste Living Standards and Measurement Survey 2007-2008. Washington DC, United States: World Bank.	X	4	X	52		All ages	Both
Trinidad and Tobago	2000	University of the West Indies, World Health Organization (WHO). Trinidad and Tobago WHO Multi-country Survey Study on Health and Health System Responsiveness 2000-2001. Geneva, Switzerland: World Health Organization (WHO).	X	4.3	X	4.3		0-95	Both
Tunisia	2003	World Health Organization (WHO). Tunisia World Health Survey 2003. Geneva, Switzerland: World Health Organization (WHO), 2005.			X	52		15-100+	Both
Turkey	1980	General Directorate of Curative Services, Ministry of Health (Turkey). Turkey Inpatient care discharges per 100 1980.			X		X	All ages	Both
Turkey	1981	General Directorate of Curative Services, Ministry of Health (Turkey). Turkey Inpatient care discharges per 100 1981.			X		X	All ages	Both
Turkey	1982	General Directorate of Curative Services, Ministry of Health (Turkey). Turkey Inpatient care discharges per 100 1982.			X		X	All ages	Both
Turkey	1983	General Directorate of Curative Services, Ministry of Health (Turkey). Turkey Inpatient care discharges per 100 1983.			X		X	All ages	Both

Turkey	1984	General Directorate of Curative Services, Ministry of Health (Turkey). Turkey Inpatient care discharges per 100 1984.			X		X	All ages	Both
Turkey	1985	General Directorate of Curative Services, Ministry of Health (Turkey). Turkey Inpatient care discharges per 100 1985.			X		X	All ages	Both
Turkey	1986	General Directorate of Curative Services, Ministry of Health (Turkey). Turkey Inpatient care discharges per 100 1986.			X		X	All ages	Both
Turkey	1987	General Directorate of Curative Services, Ministry of Health (Turkey). Turkey Inpatient care discharges per 100 1987.			X		X	All ages	Both
Turkey	1988	General Directorate of Curative Services, Ministry of Health (Turkey). Turkey Inpatient care discharges per 100 1988.			X		X	All ages	Both
Turkey	1989	General Directorate of Curative Services, Ministry of Health (Turkey). Turkey Inpatient care discharges per 100 1989.			X		X	All ages	Both
Turkey	1990	General Directorate of Curative Services, Ministry of Health (Turkey). Turkey Inpatient care discharges per 100 1990.			X		X	All ages	Both
Turkey	1991	General Directorate of Curative Services, Ministry of Health (Turkey). Turkey Inpatient care discharges per 100 1991.			X		X	All ages	Both
Turkey	1992	General Directorate of Curative Services, Ministry of Health (Turkey). Turkey Inpatient care discharges per 100 1992.			X		X	All ages	Both
Turkey	1993	General Directorate of Curative Services, Ministry of Health (Turkey). Turkey Inpatient care discharges per 100 1993.			X		X	All ages	Both
Turkey	1994	General Directorate of Curative Services, Ministry of Health (Turkey). Turkey Inpatient care discharges per 100 1994.			X		X	All ages	Both
Turkey	1995	General Directorate of Curative Services, Ministry of Health (Turkey). Turkey Inpatient care discharges per 100 1995.			X		X	All ages	Both
Turkey	1996	General Directorate of Curative Services, Ministry of Health (Turkey). Turkey Inpatient care discharges per 100 1996.			X		X	All ages	Both
Turkey	1997	General Directorate of Curative Services, Ministry of Health (Turkey). Turkey Inpatient care discharges per 100 1997.			X		X	All ages	Both
Turkey	1998	General Directorate of Curative Services, Ministry of Health (Turkey). Turkey Inpatient care discharges per 100 1998.			X		X	All ages	Both
Turkey	1999	General Directorate of Curative Services, Ministry of Health (Turkey). Turkey Inpatient care discharges per 100 1999.			X		X	All ages	Both
Turkey	2000	AMATEM (Turkey), Plaza Ltd. Research, World Health Organization (WHO). Turkey WHO Multi-country Survey Study on Health and Health System Responsiveness 2000-2001. Geneva, Switzerland: World Health Organization (WHO).	X	4.3	X	4.3		0-95	Both
Turkey	2000	General Directorate of Curative Services, Ministry of Health (Turkey). Turkey Inpatient care discharges per 100 2000.			X		X	All ages	Both
Turkey	2001, 2002, 2003, 2004, 2005, 2006, 2007, 2008, 2009, 2010, 2011, 2012, 2013, 2014, 2015	Organization for Economic Co-operation and Development (OECD). OECD Health Statistics. Paris, France: Organization for Economic Co-operation and Development (OECD).	X				X	All ages	Both
Turkey	2001	General Directorate of Curative Services, Ministry of Health (Turkey). Turkey Inpatient care discharges per 100 2001.			X		X	All ages	Both
Turkey	2002	General Directorate of Curative Services, Ministry of Health (Turkey). Turkey Inpatient care discharges per 100 2002.			X		X	All ages	Both
Turkey	2003	General Directorate of Curative Services, Ministry of Health (Turkey). Turkey Inpatient care discharges per 100 2003.			X		X	All ages	Both
Turkey	2004	General Directorate of Curative Services, Ministry of Health (Turkey). Turkey Inpatient care discharges per 100 2004.			X		X	All ages	Both
Turkey	2005	General Directorate of Curative Services, Ministry of Health (Turkey). Turkey Inpatient care discharges per 100 2005.			X		X	All ages	Both
Turkey	2006	General Directorate of Curative Services, Ministry of Health (Turkey). Turkey Inpatient care discharges per 100 2006.			X		X	All ages	Both
Turkey	2007	General Directorate of Curative Services, Ministry of Health (Turkey). Turkey Hospital Inpatient Discharges 2007.			X		X	All ages	Both
Turkey	2008	Turkish Statistical Institute. Turkey Health Interview Survey 2008. Ankara, Turkey: Turkish Statistical Institute.	X	52				0-80	Both
Turkey	2008	General Directorate of Curative Services, Ministry of Health (Turkey). Turkey Hospital Inpatient Discharges 2008.			X		X	All ages	Both
Turkey	2009	General Directorate of Curative Services, Ministry of Health (Turkey). Turkey Hospital Inpatient Discharges 2009.			X		X	All ages	Both
Turkey	2010	Turkish Statistical Institute. Turkey Health Interview Survey 2010. Ankara, Turkey: Turkish Statistical Institute.	X	52	X	52		15-80	Both
Turkey	2010	General Directorate of Curative Services, Ministry of Health (Turkey). Turkey Hospital Inpatient Discharges 2010.			X		X	All ages	Both
Turkey	2011	ISSP Research Group (2009): International Social Survey Programme: Health and Health Care - ISSP 2011. GESIS Data Archive, Cologne. ZA5800 Data file version 3.0.0, doi:10.4232/1.12252.	X	52				0-95	Both
Turkey	2011	General Directorate of Curative Services, Ministry of Health (Turkey). Turkey Hospital Inpatient Discharges 2011.			X		X	All ages	Both
Turkey	2012	General Directorate of Curative Services, Ministry of Health (Turkey). Turkey Inpatient care discharges per 100 2012.			X		X	All ages	Both

Turkey	2013	General Directorate of Curative Services, Ministry of Health (Turkey). Turkey Inpatient care discharges per 100 2013.			X		X	All ages	Both
Turkey	2014	General Directorate of Curative Services, Ministry of Health (Turkey). Turkey Inpatient care discharges per 100 2014.			X		X	All ages	Both
Turkmenistan	2000	Ministry of Health and Medical Industry (Turkmenistan). Turkmenistan Outpatient Contacts Per Person Per Year 2000.	X				X	All ages	Both
Turkmenistan	2001	Ministry of Health and Medical Industry (Turkmenistan). Turkmenistan Outpatient Contacts Per Person Per Year 2001.	X				X	All ages	Both
Turkmenistan	2002	Ministry of Health and Medical Industry (Turkmenistan). Turkmenistan Outpatient Contacts Per Person Per Year 2002.	X				X	All ages	Both
Turkmenistan	2003	Ministry of Health and Medical Industry (Turkmenistan). Turkmenistan Outpatient Contacts Per Person Per Year 2003.	X				X	All ages	Both
Turkmenistan	2004	Ministry of Health and Medical Industry (Turkmenistan). Turkmenistan Outpatient Contacts Per Person Per Year 2004.	X				X	All ages	Both
Turkmenistan	2005	Ministry of Health and Medical Industry (Turkmenistan). Turkmenistan Outpatient Contacts Per Person Per Year 2005.	X				X	All ages	Both
Turkmenistan	2006	Ministry of Health and Medical Industry (Turkmenistan). Turkmenistan Outpatient Contacts Per Person Per Year 2006.	X				X	All ages	Both
Turkmenistan	2007	Ministry of Health and Medical Industry (Turkmenistan). Turkmenistan Outpatient Contacts Per Person Per Year 2007.	X				X	All ages	Both
Turkmenistan	2008	Ministry of Health and Medical Industry (Turkmenistan). Turkmenistan Outpatient Contacts Per Person Per Year 2008.	X				X	All ages	Both
Turkmenistan	2009	Ministry of Health and Medical Industry (Turkmenistan). Turkmenistan Outpatient Contacts Per Person Per Year 2009.	X				X	All ages	Both
Turkmenistan	2010	Ministry of Health and Medical Industry (Turkmenistan). Turkmenistan Outpatient Contacts Per Person Per Year 2010.	X				X	All ages	Both
Turkmenistan	2011	Ministry of Health and Medical Industry (Turkmenistan). Turkmenistan Outpatient Contacts Per Person Per Year 2011.	X				X	All ages	Both
Turkmenistan	2012	Ministry of Health and Medical Industry (Turkmenistan). Turkmenistan Outpatient Contacts Per Person Per Year 2012.	X				X	All ages	Both
Turkmenistan	2013	Ministry of Health and Medical Industry (Turkmenistan). Turkmenistan Outpatient Contacts Per Person Per Year 2013.	X				X	All ages	Both
Turkmenistan	2014	Ministry of Health and Medical Industry (Turkmenistan). Turkmenistan Outpatient Contacts Per Person Per Year 2014.	X				X	All ages	Both
Turkmenistan	2015	Ministry of Health and Medical Industry (Turkmenistan). Turkmenistan Outpatient Contacts Per Person Per Year 2015.	X				X	All ages	Both
Uganda	2002	Economic Policy Research Centre (Uganda), Uganda Bureau of Statistics. Uganda Household Survey 2002-2003.	X	8.6				All ages	Both
Uganda	2009	Uganda Bureau of Statistics. Uganda Living Standards Measurement Survey - Integrated Survey on Agriculture 2009-2010. Washington DC, United States: World Bank.	X	8.6				0-70	Both
Uganda	2009, 2010, 2011, 2012	Infectious Diseases Research Collaboration (IDRC), Institute for Health Metrics and Evaluation (IHME), Makerere University, Ministry of Health (Uganda). Access, Bottlenecks, Costs, and Equity (ABCE) project in Uganda, 2012. Seattle, United States: Institute for Health Metrics and Evaluation (IHME), 2015.			X		X	All ages	Both
Uganda	2012	Uganda Bureau of Statistics. Uganda National Household Survey 2012-2013. Kampala, Uganda: Uganda Bureau of Statistics.	X	8.6				All ages	Both
Ukraine	1980	Centre of Health Statistics, Ministry of Health (Ukraine). Ukraine Inpatient Care Discharges per 100 1980.			X		X	All ages	Both
Ukraine	1981	Centre of Health Statistics, Ministry of Health (Ukraine). Ukraine Inpatient Care Discharges per 100 1981.			X		X	All ages	Both
Ukraine	1982	Centre of Health Statistics, Ministry of Health (Ukraine). Ukraine Inpatient Care Discharges per 100 1982.			X		X	All ages	Both
Ukraine	1983	Centre of Health Statistics, Ministry of Health (Ukraine). Ukraine Inpatient Care Discharges per 100 1983.			X		X	All ages	Both
Ukraine	1984	Centre of Health Statistics, Ministry of Health (Ukraine). Ukraine Inpatient Care Discharges per 100 1984.			X		X	All ages	Both
Ukraine	1985	Centre of Health Statistics, Ministry of Health (Ukraine). Ukraine Inpatient Care Discharges per 100 1985.			X		X	All ages	Both
Ukraine	1986	Centre of Health Statistics, Ministry of Health (Ukraine). Ukraine Inpatient Care Discharges per 100 1986.			X		X	All ages	Both
Ukraine	1987	Centre of Health Statistics, Ministry of Health (Ukraine). Ukraine Inpatient Care Discharges per 100 1987.			X		X	All ages	Both
Ukraine	1988	Centre of Health Statistics, Ministry of Health (Ukraine). Ukraine Inpatient Care Discharges per 100 1988.			X		X	All ages	Both
Ukraine	1989	Centre of Health Statistics, Ministry of Health (Ukraine). Ukraine Inpatient Care Discharges per 100 1989.			X		X	All ages	Both
Ukraine	1990	Centre of Health Statistics, Ministry of Health (Ukraine). Ukraine Inpatient Care Discharges per 100 1990.			X		X	All ages	Both
Ukraine	1991	Centre of Health Statistics, Ministry of Health (Ukraine). Ukraine Inpatient Care Discharges per 100 1991.			X		X	All ages	Both
Ukraine	1992	Centre of Health Statistics, Ministry of Health (Ukraine). Ukraine Inpatient Care Discharges per 100 1992.			X		X	All ages	Both
Ukraine	1993	Centre of Health Statistics, Ministry of Health (Ukraine). Ukraine Inpatient Care Discharges per 100 1993.			X		X	All ages	Both
Ukraine	1994	Centre of Health Statistics, Ministry of Health (Ukraine). Ukraine Inpatient Care Discharges per 100 1994.			X		X	All ages	Both
Ukraine	1995	Centre of Health Statistics, Ministry of Health (Ukraine). Ukraine Inpatient Care Discharges per 100 1995.			X		X	All ages	Both
Ukraine	1996	Kiev International Institute of Sociology, World Bank. Ukraine Household Income and Expenditures Survey 1996.	X	4.3				10-95	Both

Ukraine	1996	Centre of Health Statistics, Ministry of Health (Ukraine). Ukraine Inpatient Care Discharges per 100 1996.			X		X	All ages	Both
Ukraine	1997	Centre of Health Statistics, Ministry of Health (Ukraine). Ukraine Inpatient Care Discharges per 100 1997.			X		X	All ages	Both
Ukraine	1998	Centre of Health Statistics, Ministry of Health (Ukraine). Ukraine Inpatient Care Discharges per 100 1998.			X		X	All ages	Both
Ukraine	1999	Centre of Health Statistics, Ministry of Health (Ukraine). Ukraine Inpatient Care Discharges per 100 1999.			X		X	All ages	Both
Ukraine	2000	Kiev International Institute of Sociology, World Health Organization (WHO). Ukraine WHO Multi-country Survey Study on Health and Health System Responsiveness 2000-2001. Geneva, Switzerland: World Health Organization (WHO).	X	4.3	X	4.3		0-95	Both
Ukraine	2000	Centre of Health Statistics, Ministry of Health (Ukraine). Ukraine Inpatient Care Discharges per 100 2000.			X		X	All ages	Both
Ukraine	2001	Centre of Health Statistics, Ministry of Health (Ukraine). Ukraine Inpatient Care Discharges per 100 2001.			X		X	All ages	Both
Ukraine	2002	World Health Organization (WHO). Ukraine World Health Survey 2002-2003. Geneva, Switzerland: World Health Organization (WHO), 2005.			X	52		All ages	Both
Ukraine	2002	Centre of Health Statistics, Ministry of Health (Ukraine). Ukraine Inpatient Care Discharges per 100 2002.			X		X	All ages	Both
Ukraine	2003	Centre of Health Statistics, Ministry of Health (Ukraine). Ukraine Inpatient Care Discharges per 100 2003.			X		X	All ages	Both
Ukraine	2004	Centre of Health Statistics, Ministry of Health (Ukraine). Ukraine Inpatient Care Discharges per 100 2004.			X		X	All ages	Both
Ukraine	2005	Centre of Health Statistics, Ministry of Health (Ukraine). Ukraine Inpatient Care Discharges per 100 2005.			X		X	All ages	Both
Ukraine	2006	Centre of Health Statistics, Ministry of Health (Ukraine). Ukraine Inpatient Care Discharges per 100 2006.			X		X	All ages	Both
Ukraine	2007	Centre of Health Statistics, Ministry of Health (Ukraine). Ukraine Inpatient Care Discharges per 100 2007.			X		X	All ages	Both
Ukraine	2008	Centre of Health Statistics, Ministry of Health (Ukraine). Ukraine Inpatient Care Discharges per 100 2008.			X		X	All ages	Both
Ukraine	2009	Centre of Health Statistics, Ministry of Health (Ukraine). Ukraine Inpatient Care Discharges per 100 2009.			X		X	All ages	Both
Ukraine	2010	Concluzia-Prim Center for Survey Methodology (Moldova), East-Ukranian Foundation For Social Research, Institute for Advanced Studies (Austria), London School of Hygiene and Tropical Medicine, University of Aberdeen. Ukraine Health in Times of Transition Household Survey 2010.			X	52		15-90	Both
Ukraine	2010	Centre of Health Statistics, Ministry of Health (Ukraine). Ukraine Inpatient Care Discharges per 100 2010.			X		X	All ages	Both
Ukraine	2011	Centre of Health Statistics, Ministry of Health (Ukraine). Ukraine Inpatient Care Discharges per 100 2011.			X		X	All ages	Both
Ukraine	2012	Centre of Health Statistics, Ministry of Health (Ukraine). Ukraine Inpatient Care Discharges per 100 2012.			X		X	All ages	Both
Ukraine	2013	Centre of Health Statistics, Ministry of Health (Ukraine). Ukraine Inpatient Care Discharges per 100 2013.			X		X	All ages	Both
United Arab Emirates	1995	Health Ministers' Council for GCC States, Ministry of Health (United Arab Emirates). United Arab Emirates Family Health Survey 1995.	X	2				All ages	Both
United Arab Emirates	2000	Gallup Europe, World Health Organization (WHO). United Arab Emirates WHO Multi-country Survey Study on Health and Health System Responsiveness 2000-2001. Geneva, Switzerland: World Health Organization (WHO).	X	4.3	X	4.3		15-80	Both
United Arab Emirates	2003	World Health Organization (WHO). United Arab Emirates World Health Survey 2003. Geneva, Switzerland: World Health Organization (WHO), 2005.			X	52		15-95	Both
United States	1979	National Center for Health Statistics (NCHS), Centers for Disease Control and Prevention (CDC), United States Census Bureau. United States National Hospital Discharge Survey 1979. Hyattsville, United States: National Center for Health Statistics (NCHS), Centers for Disease Control and Prevention (CDC).			X		X	0-99	Both
United States	1980	National Center for Health Statistics (NCHS), Centers for Disease Control and Prevention (CDC), United States Census Bureau. United States National Hospital Discharge Survey 1980. Hyattsville, United States: National Center for Health Statistics (NCHS), Centers for Disease Control and Prevention (CDC).			X		X	0-99	Both
United States	1981	National Center for Health Statistics (NCHS), Centers for Disease Control and Prevention (CDC), United States Census Bureau. United States National Hospital Discharge Survey 1981. Hyattsville, United States: National Center for Health Statistics (NCHS), Centers for Disease Control and Prevention (CDC).			X		X	0-99	Both
United States	1982	National Center for Health Statistics (NCHS), Centers for Disease Control and Prevention (CDC), United States Census Bureau. United States National Hospital Discharge Survey 1982. Hyattsville, United States: National Center for Health Statistics (NCHS), Centers for Disease Control and Prevention (CDC).			X		X	0-99	Both
United States	1983	National Center for Health Statistics (NCHS), Centers for Disease Control and Prevention (CDC), United States Census Bureau. United States National Hospital Discharge Survey 1983. Hyattsville, United States: National Center for Health Statistics (NCHS), Centers for Disease Control and Prevention (CDC).			X		X	0-99	Both
United States	1984	National Center for Health Statistics (NCHS), Centers for Disease Control and Prevention (CDC), United States Census Bureau. United States National Hospital Discharge Survey 1984. Hyattsville, United States: National Center for Health Statistics (NCHS), Centers for Disease Control and Prevention (CDC).			X		X	0-99	Both

United States	1985	National Center for Health Statistics (NCHS), Centers for Disease Control and Prevention (CDC), United States Census Bureau. United States National Hospital Discharge Survey 1985. Hyattsville, United States: National Center for Health Statistics (NCHS), Centers for Disease Control and Prevention (CDC).			X		X	0-99	Both
United States	1986	National Center for Health Statistics (NCHS), Centers for Disease Control and Prevention (CDC), United States Census Bureau. United States National Hospital Discharge Survey 1986. Hyattsville, United States: National Center for Health Statistics (NCHS), Centers for Disease Control and Prevention (CDC).			X		X	0-99	Both
United States	1987	National Center for Health Statistics (NCHS), Centers for Disease Control and Prevention (CDC), United States Census Bureau. United States National Hospital Discharge Survey 1987. Hyattsville, United States: National Center for Health Statistics (NCHS), Centers for Disease Control and Prevention (CDC).			X		X	0-99	Both
United States	1988	National Center for Health Statistics (NCHS), Centers for Disease Control and Prevention (CDC). United States National Health and Nutrition Examination Survey 1988-1994. Hyattsville, United States: National Center for Health Statistics (NCHS), Centers for Disease Control and Prevention (CDC).	X	52	X	52		15-95	Both
United States	1988	National Center for Health Statistics (NCHS), Centers for Disease Control and Prevention (CDC), United States Census Bureau. United States National Hospital Discharge Survey 1988. Hyattsville, United States: National Center for Health Statistics (NCHS), Centers for Disease Control and Prevention (CDC).			X		X	0-99	Both
United States	1989	National Center for Health Statistics (NCHS), Centers for Disease Control and Prevention (CDC), United States Census Bureau. United States National Hospital Discharge Survey 1989. Hyattsville, United States: National Center for Health Statistics (NCHS), Centers for Disease Control and Prevention (CDC).			X		X	0-99	Both
United States	1990	United States Department of Health and Human Services. Centers for Disease Control and Prevention. National Center for Health Statistics. National Health Interview Survey, 1994: Second Longitudinal Study on Aging, Wave 2, 1997. ICPSR03526-v2. Ann Arbor, MI: Inter-university Consortium for Political and Social Research [distributor], 2007-03-01. http://doi.org/10.3886/ICPSR03526.v2	X	52	X	52		70-100+	Both
United States	1990	National Center for Health Statistics (NCHS), Centers for Disease Control and Prevention (CDC), US Census Bureau. United States National Health Interview Survey 1990. Hyattsville, United States: National Center for Health Statistics (NCHS), Centers for Disease Control and Prevention (CDC).	X	52	X	52		All ages	Both
United States	1990	National Center for Health Statistics (NCHS), Centers for Disease Control and Prevention (CDC), United States Census Bureau. United States National Hospital Discharge Survey 1990. Hyattsville, United States: National Center for Health Statistics (NCHS), Centers for Disease Control and Prevention (CDC).			X		X	0-99	Both
United States	1991	National Center for Health Statistics (NCHS), Centers for Disease Control and Prevention (CDC), US Census Bureau. United States National Health Interview Survey 1991. Hyattsville, United States: National Center for Health Statistics (NCHS), Centers for Disease Control and Prevention (CDC).	X	2	X	52		All ages	Both
United States	1991	National Center for Health Statistics (NCHS), Centers for Disease Control and Prevention (CDC), United States Census Bureau. United States National Hospital Discharge Survey 1991. Hyattsville, United States: National Center for Health Statistics (NCHS), Centers for Disease Control and Prevention (CDC).			X		X	0-99	Both
United States	1992	National Center for Health Statistics (NCHS), Centers for Disease Control and Prevention (CDC), US Census Bureau. United States National Health Interview Survey 1992. Hyattsville, United States: National Center for Health Statistics (NCHS), Centers for Disease Control and Prevention (CDC).	X	2	X	52		All ages	Both
United States	1992	National Center for Health Statistics (NCHS), Centers for Disease Control and Prevention (CDC), United States Census Bureau. United States National Hospital Discharge Survey 1992. Hyattsville, United States: National Center for Health Statistics (NCHS), Centers for Disease Control and Prevention (CDC).			X		X	0-99	Both
United States	1993	National Center for Health Statistics (NCHS), Centers for Disease Control and Prevention (CDC), US Census Bureau. United States National Health Interview Survey 1993. Hyattville, United States: National Center for Health Statistics (NCHS), Centers for Disease Control and Prevention (CDC).	X	52	X	52		All ages	Both
United States	1993	National Center for Health Statistics (NCHS), Centers for Disease Control and Prevention (CDC), United States Census Bureau. United States National Hospital Discharge Survey 1993. Hyattsville, United States: National Center for Health Statistics (NCHS), Centers for Disease Control and Prevention (CDC).			X		X	0-99	Both
United States	1994	National Center for Health Statistics (NCHS), Centers for Disease Control and Prevention (CDC), United States Census Bureau. United States National Hospital Discharge Survey 1994. Hyattsville, United States: National Center for Health Statistics (NCHS), Centers for Disease Control and Prevention (CDC).			X		X	0-99	Both
United States	1994	United States Department of Health and Human Services. Centers for Disease Control and Prevention. National Center for Health Statistics. National Health Interview Survey, 1994: Second Supplement on Aging. ICPSR02563-v3. Ann Arbor, MI: Inter-university Consortium for Political and Social Research [distributor], 2007-02-12. http://doi.org/10.3886/ICPSR02563.v3	X	52	X	26		65-100+	Both
United States	1995	National Center for Health Statistics (NCHS), Centers for Disease Control and Prevention (CDC), United States Census Bureau. United States National Hospital Discharge Survey 1995. Hyattsville, United States: National Center for Health Statistics (NCHS), Centers for Disease Control and Prevention (CDC).			X		X	0-99	Both

United States	1996	National Center for Health Statistics (NCHS), Centers for Disease Control and Prevention (CDC), United States Census Bureau. United States National Hospital Discharge Survey 1996. Hyattsville, United States: National Center for Health Statistics (NCHS), Centers for Disease Control and Prevention (CDC).			X		X	0-99	Both
United States	1996	Louis Harris and Associates. United States Commonwealth Fund Survey of the Health of Adolescent Girls and Boys 1996-1997. New York, United States: Commonwealth Fund, 1997.	X	52				8-18	Both
United States	1997, 1998, 1999, 2000, 2001, 2002, 2003, 2004, 2005, 2006, 2007, 2009, 2010, 2011, 2012, 2013, 2014	Institute for Health Metrics and Evaluation (IHME). United States Personal Health Care and Public Health Spending 1996-2013. Seattle, United States: Institute for Health Metrics and Evaluation (IHME), 2016.	X				X	All ages	Both
United States	1997	National Center for Health Statistics (NCHS), Centers for Disease Control and Prevention (CDC), US Census Bureau. United States National Health Interview Survey 1997. Hyattsville, United States: National Center for Health Statistics (NCHS), Centers for Disease Control and Prevention (CDC).	X	2	X	52		0-90	Both
United States	1997	National Center for Health Statistics (NCHS), Centers for Disease Control and Prevention (CDC), United States Census Bureau. United States National Hospital Discharge Survey 1997. Hyattsville, United States: National Center for Health Statistics (NCHS), Centers for Disease Control and Prevention (CDC).			X		X	0-99	Both
United States	1998	National Center for Health Statistics (NCHS), Centers for Disease Control and Prevention (CDC), US Census Bureau. United States National Health Interview Survey 1998. Hyattsville, United States: National Center for Health Statistics (NCHS), Centers for Disease Control and Prevention (CDC).	X	2	X	52		0-90	Both
United States	1998	National Center for Health Statistics (NCHS), Centers for Disease Control and Prevention (CDC), United States Census Bureau. United States National Hospital Discharge Survey 1998. Hyattsville, United States: National Center for Health Statistics (NCHS), Centers for Disease Control and Prevention (CDC).			X		X	0-99	Both
United States	1998	Louis Harris and Associates. United States Commonwealth Fund Survey of Women's Health 1998. New York, United States: Commonwealth Fund, 1999.	X	52				18-64	Female
United States	1999	National Center for Health Statistics (NCHS), Centers for Disease Control and Prevention (CDC), US Census Bureau. United States National Health Interview Survey 1999. Hyattsville, United States: National Center for Health Statistics (NCHS), Centers for Disease Control and Prevention (CDC).	X	2	X	52		0-90	Both
United States	1999	National Center for Health Statistics (NCHS), Centers for Disease Control and Prevention (CDC). United States National Health and Nutrition Examination Survey 1999-2000. Hyattsville, United States: National Center for Health Statistics (NCHS), Centers for Disease Control and Prevention (CDC).	X	52	X	52		0-90	Both
United States	1999	National Center for Health Statistics (NCHS), Centers for Disease Control and Prevention (CDC), United States Census Bureau. United States National Hospital Discharge Survey 1999. Hyattsville, United States: National Center for Health Statistics (NCHS), Centers for Disease Control and Prevention (CDC).			X		X	0-99	Both
United States	2000	National Center for Health Statistics (NCHS), Centers for Disease Control and Prevention (CDC), US Census Bureau. United States National Health Interview Survey 2000. Hyattsville, United States: National Center for Health Statistics (NCHS), Centers for Disease Control and Prevention (CDC).	X	2	X	52		0-90	Both
United States	2000	National Center for Health Statistics (NCHS), Centers for Disease Control and Prevention (CDC), United States Census Bureau. United States National Hospital Discharge Survey 2000. Hyattsville, United States: National Center for Health Statistics (NCHS), Centers for Disease Control and Prevention (CDC).			X		X	0-99	Both
United States	2000	Washington State University, World Health Organization (WHO). United States WHO Multi-country Survey Study on Health and Health System Responsiveness 2000-2001. Geneva, Switzerland: World Health Organization (WHO).	X	4.3	X	4.3		All ages	Both
United States	2001	National Center for Health Statistics (NCHS), Centers for Disease Control and Prevention (CDC), US Census Bureau. United States National Health Interview Survey 2001. Hyattsville, United States: National Center for Health Statistics (NCHS), Centers for Disease Control and Prevention (CDC).	X	2	X	52		0-90	Both
United States	2001	National Center for Health Statistics (NCHS), Centers for Disease Control and Prevention (CDC). United States National Health and Nutrition Examination Survey 2001-2002. Hyattsville, United States: National Center for Health Statistics (NCHS), Centers for Disease Control and Prevention (CDC).	X	52	X	52		0-90	Both
United States	2001	National Center for Health Statistics (NCHS), Centers for Disease Control and Prevention (CDC), United States Census Bureau. United States National Hospital Discharge Survey 2001. Hyattsville, United States: National Center for Health Statistics (NCHS), Centers for Disease Control and Prevention (CDC).			X		X	0-99	Both

United States	2002	National Center for Health Statistics (NCHS), Centers for Disease Control and Prevention (CDC), US Census Bureau. United States National Health Interview Survey 2002. Hyattsville, United States: National Center for Health Statistics (NCHS), Centers for Disease Control and Prevention (CDC).	X	2	X	52		0-90	Both
United States	2002	National Center for Health Statistics (NCHS), Centers for Disease Control and Prevention (CDC), United States Census Bureau. United States National Hospital Discharge Survey 2002. Hyattsville, United States: National Center for Health Statistics (NCHS), Centers for Disease Control and Prevention (CDC).			X		X	0-99	Both
United States	2003	National Center for Health Statistics (NCHS), Centers for Disease Control and Prevention (CDC), US Census Bureau. United States National Health Interview Survey 2003. Hyattsville, United States: National Center for Health Statistics (NCHS), Centers for Disease Control and Prevention (CDC).	X	2	X	52		0-90	Both
United States	2003	National Center for Health Statistics (NCHS), Centers for Disease Control and Prevention (CDC). United States National Health and Nutrition Examination Survey 2003-2004. Hyattsville, United States: National Center for Health Statistics (NCHS), Centers for Disease Control and Prevention (CDC).	X	52	X	52		0-90	Both
United States	2003	National Center for Health Statistics (NCHS), Centers for Disease Control and Prevention (CDC), United States Census Bureau. United States National Hospital Discharge Survey 2003. Hyattsville, United States: National Center for Health Statistics (NCHS), Centers for Disease Control and Prevention (CDC).			X		X	0-99	Both
United States	2003	Healthcare Cost and Utilization Project (HCUP), Agency for Healthcare Research and Quality (AHRQ). United States State Inpatient Databases 2003. Rockville, United States: Healthcare Cost and Utilization Project (HCUP), Agency for Healthcare Research and Quality (AHRQ).			X		X	0-99	Both
United States	2004	National Center for Health Statistics (NCHS), Centers for Disease Control and Prevention (CDC), US Census Bureau. United States National Health Interview Survey 2004. Hyattsville, United States: National Center for Health Statistics (NCHS), Centers for Disease Control and Prevention (CDC).	X	2	X	52		0-90	Both
United States	2004	National Center for Health Statistics (NCHS), Centers for Disease Control and Prevention (CDC), United States Census Bureau. United States National Hospital Discharge Survey 2004. Hyattsville, United States: National Center for Health Statistics (NCHS), Centers for Disease Control and Prevention (CDC).			X		X	0-99	Both
United States	2004	Healthcare Cost and Utilization Project (HCUP), Agency for Healthcare Research and Quality (AHRQ). United States State Inpatient Databases 2004. Rockville, United States: Healthcare Cost and Utilization Project (HCUP), Agency for Healthcare Research and Quality (AHRQ).			X		X	0-99	Both
United States	2005	National Center for Health Statistics (NCHS), Centers for Disease Control and Prevention (CDC). United States National Health and Nutrition Examination Survey 2005-2006. Hyattsville, United States: National Center for Health Statistics (NCHS), Centers for Disease Control and Prevention (CDC), 2007.	X	52	X	52		0-90	Both
United States	2005	Panel Study of Income Dynamics, 2005 public use dataset. Produced and distributed by the University of Michigan with primary funding from the National Science Foundation, the National Institute of Aging, and the National Institute of Child Health and Human Development. Ann Arbor, MI, (2011).			X	52		15-100+	Both
United States	2005	National Center for Health Statistics (NCHS), Centers for Disease Control and Prevention (CDC), United States Census Bureau. United States National Hospital Discharge Survey 2005. Hyattsville, United States: National Center for Health Statistics (NCHS), Centers for Disease Control and Prevention (CDC).			X		X	0-99	Both
United States	2005	Healthcare Cost and Utilization Project (HCUP), Agency for Healthcare Research and Quality (AHRQ). United States State Inpatient Databases 2005. Rockville, United States: Healthcare Cost and Utilization Project (HCUP), Agency for Healthcare Research and Quality (AHRQ).			X		X	0-99	Both
United States	2006	National Center for Health Statistics (NCHS), Centers for Disease Control and Prevention (CDC), US Census Bureau. United States National Health Interview Survey 2006. Hyattsville, United States: National Center for Health Statistics (NCHS), Centers for Disease Control and Prevention (CDC).	X	2	X	52		0-90	Both
United States	2006	National Center for Health Statistics (NCHS), Centers for Disease Control and Prevention (CDC), United States Census Bureau. United States National Hospital Discharge Survey 2006. Hyattsville, United States: National Center for Health Statistics (NCHS), Centers for Disease Control and Prevention (CDC).			X		X	0-99	Both
United States	2006	Healthcare Cost and Utilization Project (HCUP), Agency for Healthcare Research and Quality (AHRQ). United States State Inpatient Databases 2006. Rockville, United States: Healthcare Cost and Utilization Project (HCUP), Agency for Healthcare Research and Quality (AHRQ).			X		X	0-99	Both
United States	2007	National Center for Health Statistics (NCHS), Centers for Disease Control and Prevention (CDC). United States National Health and Nutrition Examination Survey 2007-2008. Hyattsville, United States: National Center for Health Statistics (NCHS), Centers for Disease Control and Prevention (CDC), 2009.	X	52	X	52		0-85	Both
United States	2007	National Center for Health Statistics (NCHS), Centers for Disease Control and Prevention (CDC), US Census Bureau. United States National Health Interview Survey 2007. Hyattsville, United States: National Center for Health Statistics (NCHS), Centers for Disease Control and Prevention (CDC).	X	2	X	52		0-90	Both

United States	2007	Panel Study of Income Dynamics, 2007 public use dataset. Produced and distributed by the University of Michigan with primary funding from the National Science Foundation, the National Institute of Aging, and the National Institute of Child Health and Human Development. Ann Arbor, MI, (2011).			X	52		All ages	Both
United States	2007	National Center for Health Statistics (NCHS), Centers for Disease Control and Prevention (CDC), United States Census Bureau. United States National Hospital Discharge Survey 2007. Hyattsville, United States: National Center for Health Statistics (NCHS), Centers for Disease Control and Prevention (CDC).			X		X	0-99	Both
United States	2007	Healthcare Cost and Utilization Project (HCUP), Agency for Healthcare Research and Quality (AHRQ). United States State Inpatient Databases 2007. Rockville, United States: Healthcare Cost and Utilization Project (HCUP), Agency for Healthcare Research and Quality (AHRQ).			X		X	0-99	Both
United States	2008	National Center for Health Statistics (NCHS), Centers for Disease Control and Prevention (CDC), US Census Bureau. United States National Health Interview Survey 2008. Hyattsville, United States: National Center for Health Statistics (NCHS), Centers for Disease Control and Prevention (CDC).	X	2	X	52		0-90	Both
United States	2008	National Center for Health Statistics (NCHS), Centers for Disease Control and Prevention (CDC), United States Census Bureau. United States National Hospital Discharge Survey 2008. Hyattsville, United States: National Center for Health Statistics (NCHS), Centers for Disease Control and Prevention (CDC).			X		X	0-94	Both
United States	2008	Healthcare Cost and Utilization Project (HCUP), Agency for Healthcare Research and Quality (AHRQ). United States State Inpatient Databases 2008. Rockville, United States: Healthcare Cost and Utilization Project (HCUP), Agency for Healthcare Research and Quality (AHRQ).			X		X	0-99	Both
United States	2009	National Center for Health Statistics (NCHS), Centers for Disease Control and Prevention (CDC), US Census Bureau. United States National Health Interview Survey 2009. Hyattsville, United States: National Center for Health Statistics (NCHS), Centers for Disease Control and Prevention (CDC).	X	2	X	52		0-90	Both
United States	2009	National Center for Health Statistics (NCHS), Centers for Disease Control and Prevention (CDC). United States National Health and Nutrition Examination Survey 2009-2010. Hyattsville, United States: National Center for Health Statistics (NCHS), Centers for Disease Control and Prevention (CDC), 2011.	X	52	X	52		0-85	Both
United States	2009	National Center for Health Statistics (NCHS), Centers for Disease Control and Prevention (CDC), United States Census Bureau. United States National Hospital Discharge Survey 2009. Hyattsville, United States: National Center for Health Statistics (NCHS), Centers for Disease Control and Prevention (CDC).			X		X	0-94	Both
United States	2009	Healthcare Cost and Utilization Project (HCUP), Agency for Healthcare Research and Quality (AHRQ). United States State Inpatient Databases 2009. Rockville, United States: Healthcare Cost and Utilization Project (HCUP), Agency for Healthcare Research and Quality (AHRQ).			X		X	0-99	Both
United States	2010	National Center for Health Statistics (NCHS), Centers for Disease Control and Prevention (CDC), US Census Bureau. United States National Health Interview Survey 2010. Hyattsville, United States: National Center for Health Statistics (NCHS), Centers for Disease Control and Prevention (CDC).	X	2	X	52		0-90	Both
United States	2010	National Center for Health Statistics (NCHS), Centers for Disease Control and Prevention (CDC), United States Census Bureau. United States National Hospital Discharge Survey 2010. Hyattsville, United States: National Center for Health Statistics (NCHS), Centers for Disease Control and Prevention (CDC).			X		X	0-94	Both
United States	2011	National Center for Health Statistics (NCHS), Centers for Disease Control and Prevention (CDC), US Census Bureau. United States National Health Interview Survey 2011. Hyattsville, United States: National Center for Health Statistics (NCHS), Centers for Disease Control and Prevention (CDC).	X	2	X	52		0-90	Both
United States	2011	National Center for Health Statistics (NCHS), Centers for Disease Control and Prevention (CDC). United States National Health and Nutrition Examination Survey 2011-2012. Hyattsville, United States: National Center for Health Statistics (NCHS), Centers for Disease Control and Prevention (CDC), 2013.	X	52	X	52		0-85	Both
United States	2011	ISSP Research Group (2009): International Social Survey Programme: Health and Health Care - ISSP 2011. GESIS Data Archive, Cologne. ZA5800 Data file version 3.0.0, doi:10.4232/1.12252.	X	52				0-90	Both
United States	2011	Healthcare Cost and Utilization Project (HCUP), Agency for Healthcare Research and Quality (AHRQ). United States State Inpatient Databases 2011. Rockville, United States: Healthcare Cost and Utilization Project (HCUP), Agency for Healthcare Research and Quality (AHRQ).			X		X	All ages	Both
United States	2012	National Center for Health Statistics (NCHS), Centers for Disease Control and Prevention (CDC), United States Census Bureau. United States National Health Interview Survey 2012. Hyattsville, United States: National Center for Health Statistics (NCHS), Centers for Disease Control and Prevention (CDC), 2013.	X	2	X	52		0-90	Both
United States	2012	Healthcare Cost and Utilization Project (HCUP), Agency for Healthcare Research and Quality (AHRQ). United States State Inpatient Databases 2012. Rockville, United States: Healthcare Cost and Utilization Project (HCUP), Agency for Healthcare Research and Quality (AHRQ).			X		X	All ages	Both

United States	2013	National Center for Health Statistics (NCHS), Centers for Disease Control and Prevention (CDC), United States Census Bureau. United States National Health Interview Survey 2013. Hyattsville, United States: National Center for Health Statistics (NCHS), Centers for Disease Control and Prevention (CDC), 2014.	X	2	X	52		0-90	Both
United States	2013	National Center for Health Statistics (NCHS), Centers for Disease Control and Prevention (CDC). United States National Health and Nutrition Examination Survey 2013-2014. Hyattsville, United States: National Center for Health Statistics (NCHS), Centers for Disease Control and Prevention (CDC).			X	52		0-85	Both
United States	2013	Healthcare Cost and Utilization Project (HCUP), Agency for Healthcare Research and Quality (AHRQ). United States Nationwide Inpatient Sample 2013. Rockville, United States: Healthcare Cost and Utilization Project (HCUP), Agency for Healthcare Research and Quality (AHRQ).			X		X	All ages	Both
United States	2013	Healthcare Cost and Utilization Project (HCUP), Agency for Healthcare Research and Quality (AHRQ). United States State Inpatient Databases 2013. Rockville, United States: Healthcare Cost and Utilization Project (HCUP), Agency for Healthcare Research and Quality (AHRQ).			X		X	All ages	Both
United States	2014	National Center for Health Statistics (NCHS), Centers for Disease Control and Prevention (CDC). United States National Health Interview Survey 2014. Hyattsville, United States: National Center for Health Statistics (NCHS), Centers for Disease Control and Prevention (CDC), 2015.	X	2	X	52		0-90	Both
United States	2014	Healthcare Cost and Utilization Project (HCUP), Agency for Healthcare Research and Quality (AHRQ). United States Nationwide Inpatient Sample 2014. Rockville, United States: Healthcare Cost and Utilization Project (HCUP), Agency for Healthcare Research and Quality (AHRQ).			X		X	All ages	Both
United States	2014	Healthcare Cost and Utilization Project (HCUP), Agency for Healthcare Research and Quality (AHRQ). United States State Inpatient Databases 2014. Rockville, United States: Healthcare Cost and Utilization Project (HCUP), Agency for Healthcare Research and Quality (AHRQ).			X		X	All ages	Both
United States	2015	Healthcare Cost and Utilization Project (HCUP), Agency for Healthcare Research and Quality (AHRQ). United States Nationwide Inpatient Sample 2015. Rockville, United States: Healthcare Cost and Utilization Project (HCUP), Agency for Healthcare Research and Quality (AHRQ).			X		X	All ages	Both
Uruguay	2002	World Health Organization (WHO). Uruguay World Health Survey 2002-2003. Geneva, Switzerland: World Health Organization (WHO), 2005.			X	52		15-95	Both
Uzbekistan	1981, 1982, 1983, 1995, 1996, 1997, 1998, 1999, 2000, 2001, 2002, 2003, 2004, 2005, 2006, 2007, 2008, 2009, 2010, 2011, 2012, 2013, 2014, 2015, 2016	World Health Organization Regional Office for Europe (WHO/Europe). European Health for All Database - Inpatient Care Discharges Per 100. Copenhagen, Denmark: World Health Organization Regional Office for Europe (WHO/Europe).			X		X	All ages	Both
Uzbekistan	1999, 2000, 2001, 2002, 2003, 2004, 2005, 2006, 2007, 2008, 2009, 2010, 2011, 2012, 2013, 2014, 2015, 2016	World Health Organization Regional Office for Europe (WHO/Europe). European Health for All Database - Outpatient Contacts Per Person Per Year. Copenhagen, Denmark: World Health Organization Regional Office for Europe (WHO/Europe).	X				X	All ages	Both
Uzbekistan	2002	Analytical and Information Center of the Ministry of Health of Uzbekistan, Macro International, Inc, Ministry of Macroeconomics and Statistics (Uzbekistan). Uzbekistan Special Demographic and Health Survey 2002. Calverton, United States: Macro International, Inc.			X	52		15-60	Both

Venezuela	2000	Gallup Europe, World Health Organization (WHO). Venezuela WHO Multi-country Survey Study on Health and Health System Responsiveness 2000-2001. Geneva, Switzerland: World Health Organization (WHO).	X	4.3	X	4.3			0-60	Both
Vietnam	2002	World Health Organization (WHO). Vietnam World Health Survey 2002-2003. Geneva, Switzerland: World Health Organization (WHO), 2005.			X	52			All ages	Both
Vietnam	2006	General Statistics Office (Viet Nam), United Nations Development Programme (UNDP), World Bank. Vietnam Living Standards Measurement Survey 2006.	X	52	X	52			All ages	Both
Vietnam	2008	General Statistics Office (Viet Nam), United Nations Development Programme (UNDP), World Bank (WB). Viet Nam Living Standards Measurement Survey 2008. Ha Nội, Viet Nam: General Statistics Office (Viet Nam).	X	52	X	52			All ages	Both
Wales	1999, 2001, 2002, 2003, 2004, 2006, 2007, 2009, 2010	World Health Organization Regional Office for Europe (WHO/Europe). European Hospital Morbidity Database 1999-2007. Copenhagen, Denmark: World Health Organization Regional Office for Europe (WHO/Europe).			X		X		All ages	Both
Wales	2006	Department of Health, Social Services and Public Safety (Northern Ireland), Information Centre for Health and Social Care, NHS, NHS England, NHS Health Scotland, NHS Wales. United Kingdom Hospital Patient and Discharge Data 2006.			X		X		All ages	Both
Wales	2012	Department of Health, Social Services and Public Safety (Northern Ireland), Information Centre for Health and Social Care, NHS, NHS England, NHS Health Scotland, NHS Wales. United Kingdom Hospital Patient and Discharge Data 2012.			X		X		All ages	Both
Wales	2013	Department of Health, Social Services and Public Safety (Northern Ireland), Information Centre for Health and Social Care, NHS, NHS England, NHS Health Scotland, NHS Wales. United Kingdom Hospital Patient and Discharge Data 2013.			X		X		All ages	Both
Wales	2014	Department of Health, Social Services and Public Safety (Northern Ireland), Information Centre for Health and Social Care, NHS, NHS England, NHS Health Scotland, NHS Wales. United Kingdom Hospital Patient and Discharge Data 2014.			X		X		All ages	Both
Yemen	2005	Central Statistical Organization (Yemen). Yemen Household Budget Survey 2005-2006. Sana'a, Yemen: Central Statistical Organization (Yemen).	X	4.3					All ages	Both
Yemen	2012	Ministry of Planning and International Cooperation (Yemen), International Policy Center for Inclusive Growth, Interaction in Development (Yemen), UNICEF Yemen. Yemen National Social Protection Monitoring Survey 2012-2013.	X	2					All ages	Both
Zambia	1991	Central Bureau of Statistics (Norway), Central Statistical Office (Zambia), World Bank. Zambia Priority Survey 1991.	X	12.9					All ages	Both
Zambia	1993	Central Bureau of Statistics (Norway), Central Statistical Office (Zambia). Zambia Priority Survey 1993.	X	12.9					0-9	Both
Zambia	1996	Central Statistical Office (Zambia). Zambia Living Conditions Monitoring Survey 1996. Lusaka, Zambia: Central Statistical Office (Zambia).	X	4.3					0-95	Both
Zambia	1998	Central Statistical Office (Zambia), London School of Hygiene and Tropical Medicine. Zambia Living Conditions Monitoring Survey 1998. Lusaka, Zambia: Central Statistical Office (Zambia).	X	2					All ages	Both
Zambia	2002	Central Statistical Office (Zambia). Zambia Living Conditions Monitoring Survey 2002-2003. Lusaka, Zambia: Central Statistical Office (Zambia).	X	2	X	2			All ages	Both
Zambia	2003	World Health Organization (WHO). Zambia World Health Survey 2003. Geneva, Switzerland: World Health Organization (WHO), 2005.			X	52			All ages	Both
Zambia	2004	Central Statistical Office (Zambia). Zambia Living Conditions Monitoring Survey 2004-2005. Lusaka, Zambia: Central Statistical Office (Zambia).	X	2	X	2			All ages	Both
Zambia	2006	Central Statistical Office (Zambia). Zambia Living Conditions Monitoring Survey 2006.	X	2					All ages	Both
Zambia	2008, 2009, 2010, 2011	Central Statistical Office (Zambia), Churches Health Association of Zambia (CHAZ), Clinton Health Access Initiative (CHAI), Institute for Health Metrics and Evaluation (IHME), Ministry of Health (Zambia), University of Zambia. Access, Bottlenecks, Costs, and Equity (ABCE) project in Zambia, 2011-2012. Seattle, United States: Institute for Health Metrics and Evaluation (IHME), 2015.			X		X		All ages	Both
Zambia	2010	Central Statistical Office (Zambia). Zambia Living Conditions Monitoring Survey 2010.	X	2					All ages	Both
Zimbabwe	2003	World Health Organization (WHO). Zimbabwe World Health Survey 2003. Geneva, Switzerland: World Health Organization (WHO), 2005.			X	52			All ages	Both
